# Asymmetric Suzuki-Miyaura coupling of heterocycles via Rhodium-catalysed allylic arylation of racemates

**DOI:** 10.1038/ncomms15762

**Published:** 2017-06-13

**Authors:** Philipp Schäfer, Thomas Palacin, Mireia Sidera, Stephen P. Fletcher

**Affiliations:** 1 Chemistry Research Laboratory, Department of Chemistry, University of Oxford, 12 Mansfield Road, Oxford OX1 3TA, UK

## Abstract

Using asymmetric catalysis to simultaneously form carbon–carbon bonds and generate single isomer products is strategically important. Suzuki-Miyaura cross-coupling is widely used in the academic and industrial sectors to synthesize drugs, agrochemicals and biologically active and advanced materials. However, widely applicable enantioselective Suzuki-Miyaura variations to provide 3D molecules remain elusive. Here we report a rhodium-catalysed asymmetric Suzuki-Miyaura reaction with important partners including aryls, vinyls, heteroaromatics and heterocycles. The method can be used to couple two heterocyclic species so the highly enantioenriched products have a wide array of cores. We show that pyridine boronic acids are unsuitable, but they can be halogen-modified at the 2-position to undergo reaction, and this halogen can then be removed or used to facilitate further reactions. The method is used to synthesize isoanabasine, preclamol, and niraparib—an anticancer agent in several clinical trials. We anticipate this method will be a useful tool in drug synthesis and discovery.

## Abstract (web summary)

Asymmetric Suzuki-Miyaura procedures often have difficulty incorporating heterocyclic reagents, despite the importance of these in the pharmaceutical industry. Here the authors report a rhodium catalysed cross-coupling that tolerates a wide variety of nucleophiles including a range of heterocycles.

The ability to construct new bonds is important in chemical synthesis. A widely used approach is the metal catalysed cross-coupling of two simple molecules to create a more complex product^1^. In particular the Suzuki-Miyaura reaction, which involves cross-coupling between an sp^2^-hybridized boronic acid and an sp^2^-hybridized halide, is robust and highly tolerant of varying the reaction partners. These features have made it arguably the most strategically important carbon–carbon bond forming reaction, and it is one of the top transformations used in drug discovery^2^. A key feature of Suzuki-Miyaura reactions is the ability to use heteroaromatic units as coupling partners. These are more difficult to work with than simple aromatics like benzene, but are essential components of many drugs and agrochemicals (Fig. 1a)^3^. It is now widely accepted that the exceptional reliability of Suzuki-Miyaura procedures to form Csp^2^–Csp^2^ bonds (Fig. 1b)^4^ has skewed drug development disproportionately toward materials which are essentially flat or planer and lack three-dimensionality—however, a major recent trend within drug discovery is to explicitly shift toward 3D molecules^2^,^5^.

Efforts to extend Suzuki-Miyaura reactions to the preparation of enantiomerically pure compounds^6^ has led to a variety of enantiospecific methods using single-enantiomer alkylboron reagents^7^,^8^,^9^,^10^,^11^,^12^ or halide partners^13^. In terms of asymmetric methods, very few generally useful procedures have emerged, but desymmetrization strategies using *meso*-compounds are known^14^,^15^. Enantioselective Csp^2^–Csp^2^ coupling to form axially chiral biaryls is also well established^16^ and the Fu group pioneered stereoconvergent nickel-catalysed cross-coupling procedures between various nucleophiles and secondary coupling partners^17^,^18^. Despite this progress, relevant asymmetric procedures favour products with structural characteristics far from those typically seen in drugs and are generally restricted to aromatic or aliphatic partners. Methods that tolerate heterocycles have not been developed despite how heavily these feature in biologically active molecules^3^,^5^. As Suzuki-Miyaura coupling is the most widely used method for C–C bond forming heteroaryl elaboration^19^, the ability to use heterocycles is likely necessary for an asymmetric variations to become widely used^20^,^21^,^22^. Even in the absence of stereochemical questions, regio- and chemoselectivity issues with heteroaryls in sp^2^–sp^2^ coupling are of tremendous interest because of the inherent reactivity differences across heteroaromatics, and the possibility of performing multiple couplings on polyhalogenated heteroaromatics^3^,^23^.

Previous asymmetric reactions with sp^2^-hybridized boronic acid derivatives include 1,4- (ref. 24) and 1,2-additions^25^, allylic arylations^26^, allylic vinylations^27^, desymmetrizing allylic additions^28^,^29^, and allylic arylations to racemic substrates^30^. These work well with carbocyclic benzene derivatives, but the vast range of vinyl and heteroaromatic possibilities cannot generally be used^20^,^27^,^31^. Many vinylboronic acids cannot be used in asymmetric additions because they are unstable and readily undergo protodeboronation and polymerization^32^,^33^. We previously reported catalytic asymmetric addition of nucleophiles to racemic allylic halide starting materials^34^,^35^,^36^,^37^ including a system for boronic acid addition^30^ involving [Rh(cod)(OH)]_2_ and (*S*)-Xyl-P-PHOS in the presence of Cs_2_CO_3_. Although this system was suitable for an array of carbocyclic nucleophiles and a few electron rich styrenyl-boronic acids, it was not useful with other vinyl or heteroaromatic species.

Here we present a rhodium-catalysed asymmetric Suzuki-Miyaura cross-coupling that allows variability in both coupling partners (Fig. 1c). A variety of aryl, vinyl and heteroaryl boronic acids react with carbo- and heterocyclic allyl halides. The method gives diverse pharmacologically important core structures bearing useful functional groups. The functional groups allow transformations including cross-coupling and addition reactions to rapidly elaborate the products. High enantioselectivity is observed, even when both coupling partners are heterocycles. We show that a class of cross-coupling partners (pyridine boronic acids) that do not undergo reaction can be coerced into cross-coupling by introducing a halogen to the core. If desired the halogen can be removed after asymmetric Suzuki-Miyaura, or used in further cross-coupling reactions. The method is used to synthesize the pyridine-containing natural alkaloid isoanabasine, whose only previous asymmetric synthesis relied on separation of the racemic mixture of enantiomers^38^. This asymmetric Suzuki-Miyaura reaction is also used in the key step of two syntheses of the antipsychotic drug preclamol, and in three syntheses of niraparib (ZEJULA), an anticancer agent in many late-stage clinical trials.

## Results

### Asymmetric reaction development

**Figure 2:**
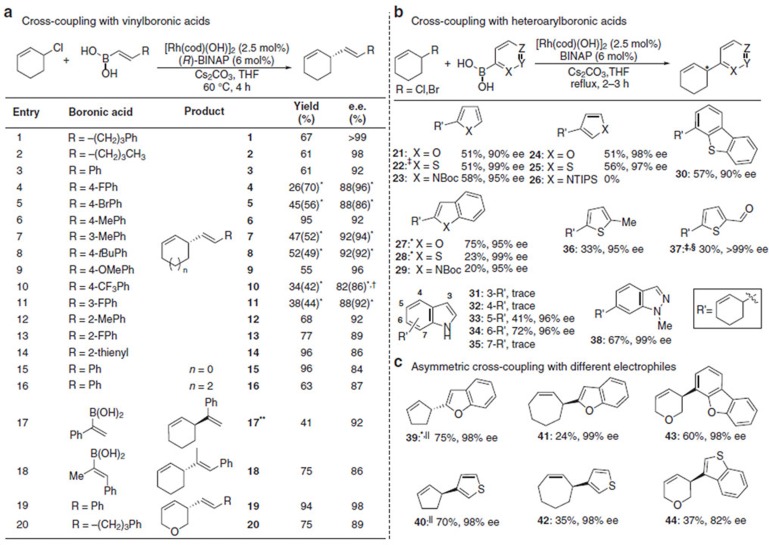
Asymmetric Suzuki-Miyaura coupling using vinyl- and heteroaryl boronic acids. (**a**) Cross-coupling vinylboronic acids to cyclic allyl chlorides. Conditions: 0.4 mmol of allyl chloride, 0.8 mmol of vinylboronic acid, [Rh(cod)(OH)]_2_ (2.5 mol%), ligand (6 mol%), Cs_2_CO_3_ (1.0 eq) in THF at 60 °C. (**b**) Testing various heteroaromatic boronic acids in asymmetric cross-coupling. (**c**) Hetereoaryl boronic acids used in combination with different allyl chlorides. Conditions: 0.4 mmol of allyl chloride, 1.2 mmol of heteroaryl boronic acid, [Rh(cod)(OH)]_2_ (2.5 mol%), ligand (6 mol%), Cs_2_CO_3_ (1.00 eq) in THF at reflux. *In these experiments Xyl-P-PHOS was used instead of BINAP. ^†^It is difficult to determine the ee of product **10** and the ee values here have an estimated error of ±10%. ^‡^Allyl bromide was used instead of allyl chloride. ^§^The reaction was performed at r.t. for 16 h. ^‖^In these experiments 5 mol% [Rh(cod)(OH)]_2_ and 12 mol% ligand was used and the reaction stirred for four hours at reflux while protected from light. All yields are isolated yields. Enantiomeric excesses determined by HPLC, GC or SFC using a chiral non-racemic stationary phase. **Reaction using (*S*)-BINAP and stirred at room temperature 48 h.

With the aim of developing a broadly useful asymmetric variant of Suzuki-Miyaura coupling with building blocks that go beyond simple bench-mark substrates, we extensively explored different catalytic systems by changing reaction parameters. Ultimately, we uncovered a variety of conditions that provide comparable (Supplementary Fig. 1) and complementary (shown below) results. We first sought useful conditions for vinylboronic acid coupling, and after thoroughly screening parameters found that BINAP generally gave better results which allowed us to use a variety of styrene derivatives with high yields and enantioselectivities (Fig. 2a, entries 1–13), although in some cases Xyl-P-PHOS was a more effective ligand (entries 4, 5, 7, 8, 10 and 11). We found that when using 1-phenyl-1-vinylboronic acid, the major product formed was the regioisomer 3 in 75% yield and 91% ee. However, when the reaction was stirred at room temperature for 48 h, the major isomer was **17** in a 4.5:1 ratio. **17** was isolated in 41% yield and 92% ee. The method is suitable for trisubstituted boronic acids as well as additions to 5-,7- and oxygen-containing rings (**15**, **16**, **19** and **20**).

Although thousands of heteroaryl boronic acids are known, for practical reasons we examined a representative unsubstituted set to judge whether these are suitable for asymmetric Suzuki-Miyaura coupling. BINAP was used, >2 equiv. of boronic acid and heating to reflux gave good results (Fig. 2b). At the 2-position of 5-membered O, S and N (Boc-protected) heterocycles, asymmetric addition was observed in >50% yield and very high ee (90–99%, **21**–**23**). At the 3-position, furan **24** (98% ee) and thiophene **25** (97% ee) derivatives also worked well, but products from a protected 3-pyrrole **26** were never isolated, likely due to rapid base-mediated protodeboronation^39^. 2-Boronic acid substituted 5-membered heterocycles fused to a benzene ring gave excellent levels of ee (all >95%) with high yield in the case of benzofuran (**27**, 75%) but low yields with benzothiophene **28** (23%) and protected indole **29** (20%). Dibenzothiophene (to give **30**, 57% 90% ee) and dibenzofuran could be added successfully, but the product from the furan derivative could not be satisfactorily separated from protoboronated dibenzofuran. For indole addition, reaction at the 5- and 6-position gave good results with excellent ee’s (**33** and **34**, both 96% ee) in sharp contrast 3-, 4- and 7-isomers, which could only be obtained in very small amounts due to the stability of the boronic acids during synthesis or under the reaction conditions. Because they were readily available we also examined 5-substituted thiophenes, which produced **36** and **37** with very high enantioselectivities (95–99% ee) and indazolyl to give **38** in 67% yield with 99% ee. A variety of different electrophiles were also successfully employed using heterocyclic boronic acids (Fig. 2c).

### Coupling with pyridylboronic acid derivatives

**Figure 3:**
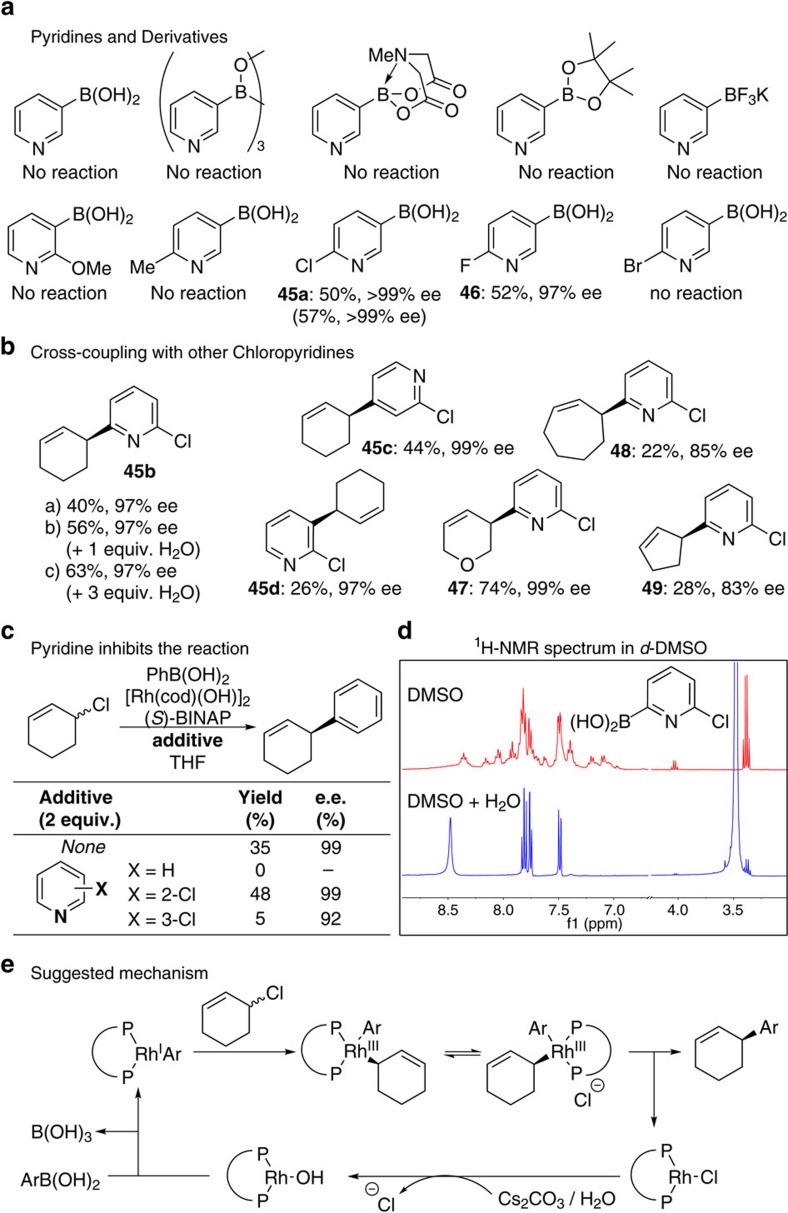
Asymmetric Suzuki-Miyaura coupling with pyridine-derived boronic acids. (**a**) Examination of pyridine boronic acid derivatives and core-modified pyridyl boronic acids. (**b**) Cross-coupling of various chloropyridineboronic acids. (**c**) Pyridine inhibits an asymmetric coupling reaction while 2-Cl-pyridine does not, suggesting that the role of the 2-Cl-unit is to make the pyridine-based partner less Lewis basic and bind less effectively to Rh-species. (**d**) Addition of water to the 2-Cl-pyridinyl boronic acid simplifies its NMR spectra in DMSO suggesting it breaks aggregates into monomers. (**e**) Tentative reaction mechanism for the Rh-catalyzed cross-coupling. All yields are isolated yields.

The most valuable heterocycles are arguably pyridine derivatives, which are important in catalysis, drug design, molecular recognition and feature in many natural products. Experiments with pyridine boronic acids were unsuccessful, as were extensive experiments using boronic acid derivatives under various conditions (Fig. 3a). We decided to modulate the pyridine electronics by introducing various substituents and found that 2-chloro and 2-fluoro pyridines could be used with respectable (>50%) yield and excellent (>97% ee) enantioselectivity. Upon examining all regioisomers of 2-chloropyridine boronic acids we observe uniformly high enantioselectivity (97–99% ee, **45a**–**45d**) when coupling to 6-membered rings although the yield with 2-chloro-3-boronic acid-pyridine **45d** was low (26%) and couplings to 5- and 7-membered rings gave poor ee’s (80–85% ee). These results show that coupling success is highly dependent on the heterocycle, and in unsuccessful cases heterocycle modifications can be made to make the asymmetric Suzuki-Miyaura viable. Later we show that the 2-Cl-group can be readily removed or become part of synthesis design to allow subsequent transformations.

In an attempt to understand the pyridine-based boronic acid behaviour we performed asymmetric coupling of phenylboronic acid and cyclohexenyl chloride using heteroaryl-optimized conditions in the presence of additives (Fig. 3c)^40^. With no additive the reaction provided the carbocyclic coupling product (35%, 99% ee), but if pyridine is added the reaction does not work. Conversely, in the presence of 2 equivalents of 2-Cl-pyridine the coupling proceeds similarly to the unspiked reaction (48% yield, 99% ee). With 3-Cl-pyridine, only a very small amount of product is obtained with lower (92%) ee. Although we suspect the poor results with 3-pyrrole and indole boronic acids (Fig. 2) are due to competitive protodeboronation^39^, in the case of pyridines it is likely that the *N*-lone pairs interfere by binding to rhodium and shutting down a key step in the mechanistic cycle. The role of the 2-F or 2-Cl substituent would be to remove electron density, and hence Lewis basicity, from the pyridine ring. And the observed trend is consistent with their measured and calculated p*K*_a_ values (pyr ∼5.2, 3-Cl-pyr ∼2.8 and 2-Cl-pyr ∼0.7)^41^.

During studies on **45b** we established that the addition of water considerably impacts performance, and we demonstrate that in the presence of three equivalents of water the yield improves from 40 to 63% over dry conditions (Fig. 3b). The water may break up boron-aggregates into acid monomers as suggested by NMR spectroscopy experiments where water is added to **45b** in DMSO (Fig. 3c) and/or facilitate regeneration of L*RhOH species for the catalytic cycle (Fig. 3e). A plausible reaction mechanism, which we believe is consistent with our experiments, is proposed. Key steps involve transmetallation of boronic acids from B to Rh, which is likely to involve L*RhOH, and oxidative addition of (hetero)aryl-Rh species with the allyl chloride. The resulting Rh(III) intermediate may undergo suprafacial 1,3-isomerization between two different allyl species, and if reductive elimination is rate-determining this would provide a mechanism for enantioselection. These proposed steps are all tentative and elucidation of the actual mechanism will require careful further study.

### Use of piperidine substrates

**Figure 4:**
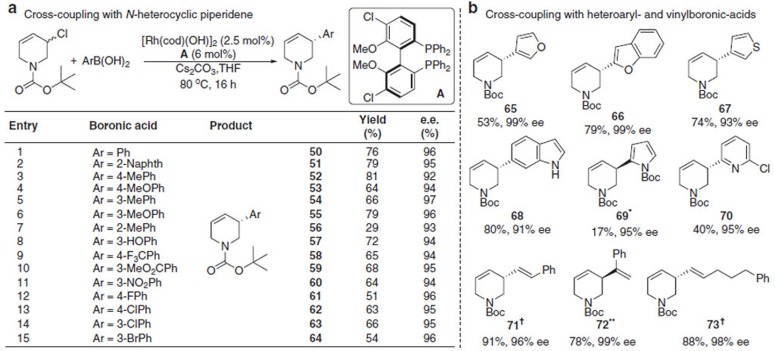
Coupling piperidenyl derivatives. (**a**) Addition of arylboronic acids to *N*-Boc-3-chloro-4-piperidene. Conditions: piperidinyl chloride (1.0 equiv.), boronic acid (2.0 equiv.), [Rh(cod)(OH)]_2_ (2.5 mol%), **A** (6 mol%), Cs_2_CO_3_ (1.0 equiv.) in THF at 80 °C with stirring for 16 h. Enantiomeric excess determined by HPLC using a chiral non-racemic stationary phase. (**b**) Addition of heteroaromatic- and vinylboronic acids to *N*-Boc-3-chloro-4-piperidene. *(*S*)-BINAP was used instead of (*R*)-**A**. ^†^(*R*)-BINAP was used instead of (*R*)-**A**. All yields are isolated yields. **Reaction using (*S*)-A run and stirred at room temperature 72 h.

Using an *N-*heterocyclic allyl chloride partner, which gives products of great interest to synthesis and biology, *tert*-butyloxycarbonyl (Boc) was found to be a suitable and easily removable *N-*protecting group. Asymmetric Suzuki-Miyaura cross-coupling to provide dehydropiperidine products are scarce, with the most relevant method being enantiospecific coupling of enantiomerically enriched allylic boronates^42^. With phenylboronic acid the conditions above gave lower ee’s (Supplementary Fig. 52), but we found that Cl-OMe-BIPHEP **A** gave excellent results (76%, 96% ee, Fig. 4a, entry 1). Modified conditions are required but they simply involve heating the reaction mixture in a septum-sealed round-bottomed flask to 80 °C under an inert atmosphere. For reasons that are unclear the use of a reflux condenser reproducibly gives inferior results. Alternatively we found the reaction can be accomplished by heating in a sealed microwave-vial overnight, or in <1 h using microwave heating set at 80 °C. A variety of substituted arylboronic acids could be coupled in this manner with uniformly high ee’s (92–97% ee), and good yields (>50%), except in the case of *ortho*-substitution (entry 7) which, likely for steric reasons, gave **56** in 29% yield.

### Asymmetric Suzuki-Miyaura coupling of two heterocycles

We next examined the challenging scenario where both coupling partners are heterocycles (Fig. 4b). 3-Furan, 2-benzofuran, 3-thiophene and 6-indole boronic acids performed well with the *N*-heterocycle under these conditions. Boc-protected 2-pyrrole boronic acid gave **69** in low yield, again likely due to sterics, but excellent (95%) ee. Modified-pyridine and vinylboronic acids also couple to the piperidene to rapidly give complex products with very high (93–99%) ee. As we observed in the earlier example, the addition of 1-phenyl-1-vinylboronic acid at 60 °C yields **71** as the major regioisomer. If the reaction is performed at room temperature and stirred 72 h, then **72** is the major product in a 7:1 ratio. **72** was obtained in 78% yield and 99% ee.

### Synthetic potential and applications

**Figure 5:**
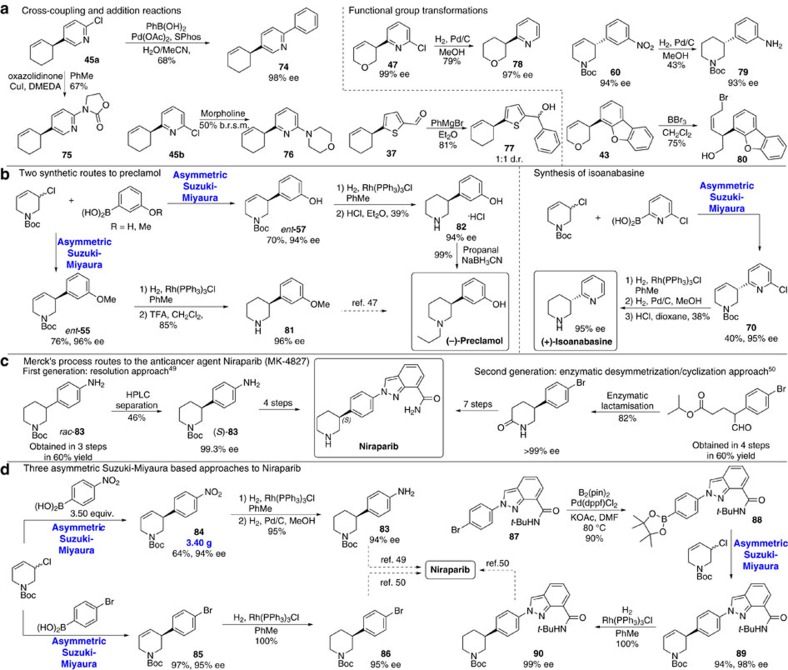
Further transformation of asymmetric Suzuki-Miyaura products and applications in drug and natural product synthesis. (**a**) Representative transformations of different asymmetric coupling products: cross-coupling and S_N_Ar reactions with 2-pyridine derivatives, nucleophile addition to an aldehyde, dehalogenation/hydrogenation, chemoselective ring opening with BBr_3_ and nitro-group reduction/hydrogentation. (**b**) Application of the method to the synthesis of the drug (−)-preclamol and the natural product (+)-isoanabasine. (**c**) Syntheses of the anticancer agent niraparib by Merck. Merck’s first process route involved resolution of racemic starting material by HPLC. Their second-generation route used an enzymatic lactamization approach to provide enantiomerically pure material. (**d**) To highlight the robustness and flexibility of asymmetric Suzuki-Miyaura coupling we used it as the key step in three different approaches to niraparib. All three syntheses intercept an intermediate reported by Merck and show high overall yields and levels of enantioselectivity. All yields are isolated yields.

After establishing that diverse coupling partners can be used in asymmetric Suzuki-Miyaura coupling we explored if the products could be used in further transformations (Fig. 5a). The 2-Cl-pyr-unit, necessary for successful reaction with pyridines, allows Pd-catalysed Suzuki-Miyaura to give **74** and Cu-catalysed Buchwald-Hartwig coupling to give **75**. Similarly, **45b** can be converted to **76** via S_N_Ar addition. As shown above (Fig. 2, **37**) sensitive aldehyde groups tolerate asymmetric coupling, providing a convenient handle for elaboration and we demonstrate addition of the prototypical nucleophile PhMgBr (**37**–**77**). We demonstrate that the 2-Cl-unit can be readily dechlorinated; here, hydrogenation of the double bond also occurs using H_2_ and Pd/C. Double nitro- and olefin-group reduction (**60**–**79**) can be accomplished using the same conditions. In these Pd-catalysed hydrogenations little racemization occurs, but in some later experiments hydrogenations were performed using Wilkinson’s catalyst to avoid racemization. Furthermore, the allyl units generated in the reaction can mask highly functionalized building blocks for synthesis, and we show how regiospecific BBr_3_-mediated ring opening (**43**–**80**) generates an enantiomerically pure heterocycle bearing both a *cis*-allyl bromide and an alcohol.

Many chiral drugs administered in enantiopure form are prepared as racemic mixtures and resolved via classical or chromatographic processes^43^ and the ability to use an asymmetric Suzuki-Miyaura coupling during molecular construction would be a highly attractive alternative. To demonstrate the method can be used to synthesize valuable molecules we prepared the antipsychotic drug preclamol^44^ whose asymmetric synthesis has recently been reported using powerful methods^45^,^46^. Using standard conditions for piperidene derivatives, **81** which had been previously converted to preclamol^47^, was prepared from 3-MeO-Ph boronic acid in three steps in 64% overall yield and 96% ee (Fig. 5b). Alternatively, unprotected 3-phenol boronic acid afforded *ent*-**57** (70%, 94% ee) and gives preclamol via a novel route involving hydrogenation and deprotection (39% yield) followed by reductive amination (99% yield).

We demonstrate that the asymmetric cross-coupling of two heterocyclic species can be used to synthesize the natural product isoanabasine, which bears considerable resemblance to common central nervous system activators. The only previous asymmetric synthesis of isoanabasine relied on preparation of the racemate followed by resolution using stoichiometric BINOL^38^. Here we used 2-Cl-pyridine boronic acid to obtain **70** in 40% yield and 95% ee. Isoanabasine was obtained after sequential hydrogenation using Wilkinson’s catalyst to reduce the double bond and then Pd/C to dehalogenate the pyridinyl moiety, followed by Boc deprotection (38% yield over three steps).

Niraparib (MK-4827) is a poly (ADP-ribose) polymerase inhibitor with potential antineoplastic activity that is currently in at least 8 different clinical trials for various cancers including those of the breast, ovarian, lung and prostate^48^. The medicinal chemistry approach to niraparib involved the tartaric acid salt of a 3-aryl piperidine and inefficient resolution via multiple crystallizations (20% yield, 80–90% ee), followed by chiral HPLC purification at a later stage^49^. For the large scale synthesis Merck first developed an approach based on chiral HPLC separation of *rac*-**83** (46% yield, 94% ee) capable of 0.27 Kg separated product per Kg stationary phase per day (Fig. 5c)^49^. A second-generation route involves a novel transaminase-mediated dynamic kinetic resolution which overcame limitations of the previous approach and demonstrates the enabling potential of advances in catalysis technology on chemical process development^50^.

We demonstrate the power of our approach by describing three different catalytic asymmetric syntheses of niraparib (Fig. 5d). First we intercepted intermediate **83** from Merck’s resolution route by coupling piperidene chloride with 4-nitrobenzeneboronic acid to give **84** (64% yield, 94% ee). To test the robustness of the method we performed a larger-scale reaction to give 3.4 grams of product. Two-stage reduction, first using Wilkinson’s catalyst to hydrogenate the double bond, then Pd/C catalysed nitro-reduction, gave **83** in 95% yield (94% ee). Alternatively, asymmetric coupling using 4-bromobenzene boronic acid provides heterocycle **85** (97%, 95% ee) which can easily be hydrogenated to Boc-protected piperidine **86** (100% yield) a key intermediate in Merck’s second generation synthesis. Finally, we envisaged that the synthesis could be made more convergent by using a complex boronic acid in the asymmetric coupling. We prepared boronate ester **88** as the corresponding boronic acid was poorly behaved making it difficult to isolate and unsuitable for asymmetric coupling. However, use of boronate ester **88** directly afforded **89** (94% yield, 98% ee) and quantitative reduction gave piperidene **90**. Previously, **90** was prepared in nine steps and then doubly deprotected to niraparib.

It is anticipated that the ability to couple boronic acids with allylic halides in an asymmetric Suzuki-Miyaura reaction, particularly when two heterocyclic partners are coupled, will become a widely used transformation in drug discovery and synthesis. In pyridine derivatives, halogen-modification allows coupling to occur, suggesting that many additional heterocycles will be compatible with this approach as it develops. The halogen modifications also facilitate subsequent transformations and enable efficient synthesis design as well as strategies for producing libraries of compounds for screening. The short syntheses of preclamol, isoanabasine and niraparib highlight the flexibility and utility of the method.

## Methods

### General Methods

For synthetic details and analytical data for all reaction products see Supplementary Methods.

### Data availability

All data supporting the findings of this study are available within the article and its accompanying Supplementary Information file, which are both free of charge to access. For NMR spectra and HPLC, GC or UPLC traces see Supplementary Figs 2–51 and 53–99.

## Additional information

**How to cite this article:** Schäfer, P. *et al*. Asymmetric Suzuki-Miyaura coupling of heterocycles via Rhodium-catalysed allylic arylation of racemates. *Nat. Commun.* **8,** 15762 doi: 10.1038/ncomms15762 (2017).

**Publisher’s note**: Springer Nature remains neutral with regard to jurisdictional claims in published maps and institutional affiliations.

## Supplementary Material

Supplementary Information Supplementary figures, supplementary methods and supplementary references.

## Figures and Tables

**Figure 1:**
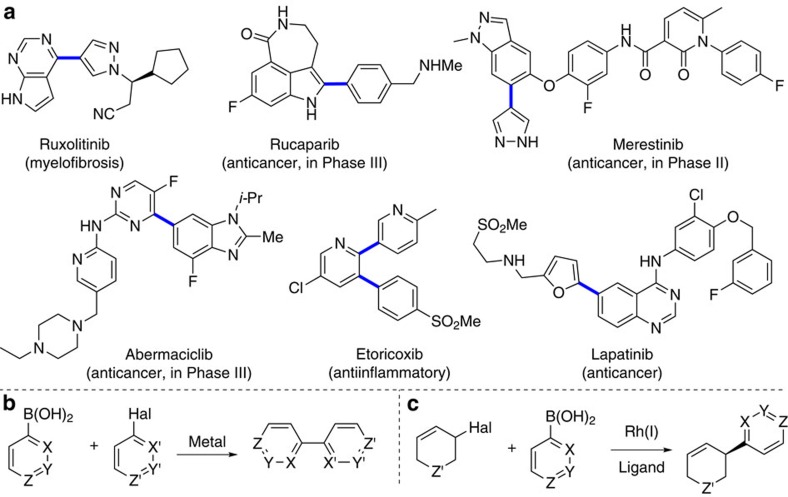
Suzuki-Miyaura cross-coupling. (**a**) Examples of drugs and late-stage drug candidates where Suzuki-Miyaura coupling is used to form key carbon–carbon bonds (shown in blue) with heterocyclic coupling partners. (**b**) The Suzuki-Miyaura reaction is strategically powerful in drug synthesis and design due to its robustness and tolerance of heterocycles. (**c**) This work: asymmetric Suzuki-Miyaura coupling to form Csp^3^–Csp^2^ bonds using heterocycles where X, Y or Z≠CH.
