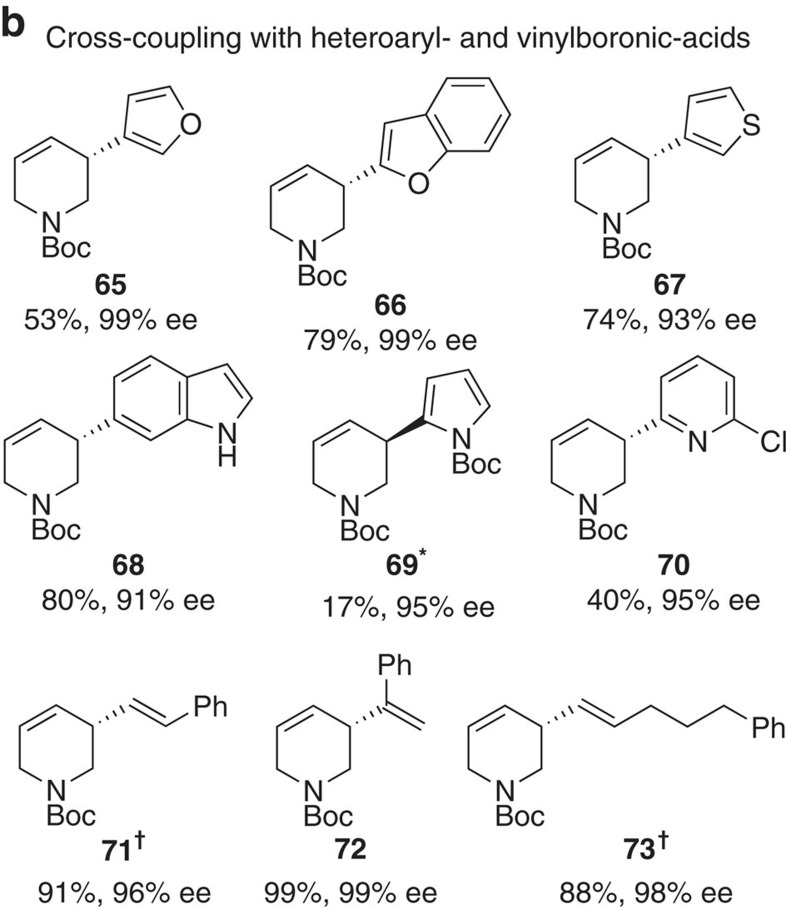# Author Correction: Asymmetric Suzuki-Miyaura coupling of heterocycles via Rhodium-catalysed allylic arylation of racemates

**DOI:** 10.1038/ncomms16216

**Published:** 2018-05-25

**Authors:** Philipp Schäfer, Thomas Palacin, Mireia Sidera, Stephen P. Fletcher

Nature Communications 8: Article number: 15762; DOI: 10.1038/ncomms15762 (2017); Published 06 13 2017; Updated 05 25 2018.

The authors became aware of an error in the original version of this Article, in that two products, **17** and **72**, were incorrectly assigned as the 1,1-alkenylated products, but the products obtained in these experiments were in fact 1,2-alkenylated isomers. The 1,1-disubstituted isomers **17** and **72** can be obtained as the major products if the Suzuki-Miyaura reactions are run at room temperature. As a result of this, the following changes have been made to the original version of this Article:

The fourth sentence of the first paragraph of the ‘Asymmetric reaction development’ section of the Results originally stated ‘The method is suitable for germinal disubstituted (to give **17**, 91% ee, entry **17**) and trisubstituted boronic acids as well as additions to 5-,7- and oxygen-containing rings (**15**, **16**, **19** and **20**).’ The updated version replaces this with ‘We found that when using 1-phenyl-1-vinylboronic acid, the major product formed was the regioisomer 3 in 75% yield and 91% ee. However, when the reaction was stirred at room temperature for 48 h, the major isomer was **17** in a 4.5:1 ratio. **17** was isolated in 41% yield and 92% ee. The method is suitable for trisubstituted boronic acids as well as additions to 5-,7- and oxygen-containing rings (**15**, **16**, **19** and **20**).’

At the end of the ‘Asymmetric Suzuki-Miyaura coupling of two heterocycles’ section of the Results, the following paragraph has been added: ‘As we observed in the earlier example, the addition of 1-phenyl-1-vinylboronic acid at 60 °C yields **71** as the major regioisomer. If the reaction is performed at room temperature and stirred 72 h, then **72** is the major product in a 7:1 ratio. **72** was obtained in 78% yield and 99% ee.’

In Fig. 2a, the ‘Product’ associated with ‘17**’ (previously ‘**17**’) has been updated, and the associated ‘Yield’ and ‘e.e.’ have been changed from ‘75’ and ‘91’ to ‘41’ and ‘92’, respectively. The correct version of Fig. 2 appears below as figure 1

which replaces the previous incorrect version, given below as Figure 2.

At the end of the legend for Fig. 2, the following sentence has been added: ‘**Reaction using (*S*)-BINAP and stirred at room temperature 48 h.’

In Fig. 4b, the chemical structure associated with ‘72**’ (previously ‘**72**’) has been updated, and the associated yield and e.e. changed from ‘99%, 99% ee’ to ‘78%, 99% ee’. The correct version of Fig. 4 which appears below as figure 3

which replaces the previous incorrect version, given below as Figure 4.

At the end of the legend for Fig. 4, the following sentence has been added: ‘**Reaction using (*S*)-A run and stirred at room temperature 72 h.’

This has been corrected in both the PDF and HTML versions of the Article.

The original version of the Supplementary Information associated with this Article has been revised to reflect these changes. Specifically, the previous section ‘(+)-(R)-(1-(Cyclohex-2-en-1-yl)vinyl)benzene (17)’ on page 15 has been replaced by the section ‘(−)-(*S*)-(1-(Cyclohex-2-en-1-yl)vinyl)benzene (**17**)’ on page 14, while the section ‘(+)-(R)-N-tert-Butoxycarbonyl-5-(1-phenylvinyl)-3-piperidene (72)’ on pages 57 and 58 has been replaced by the section ‘(−)-(*S*)-*N*-*tert*-Butoxycarbonyl-5-(1-phenylvinyl)-3-piperidene (**72**) ’ on page 57. Supplementary Figure 18 and Supplementary Fig. 75 have all been replaced. The previous versions of these sections and figures appear now as strikethrough text and figures after the corresponding correct versions in the updated Supplementary Information.

## Figures and Tables

**Figure 1:**
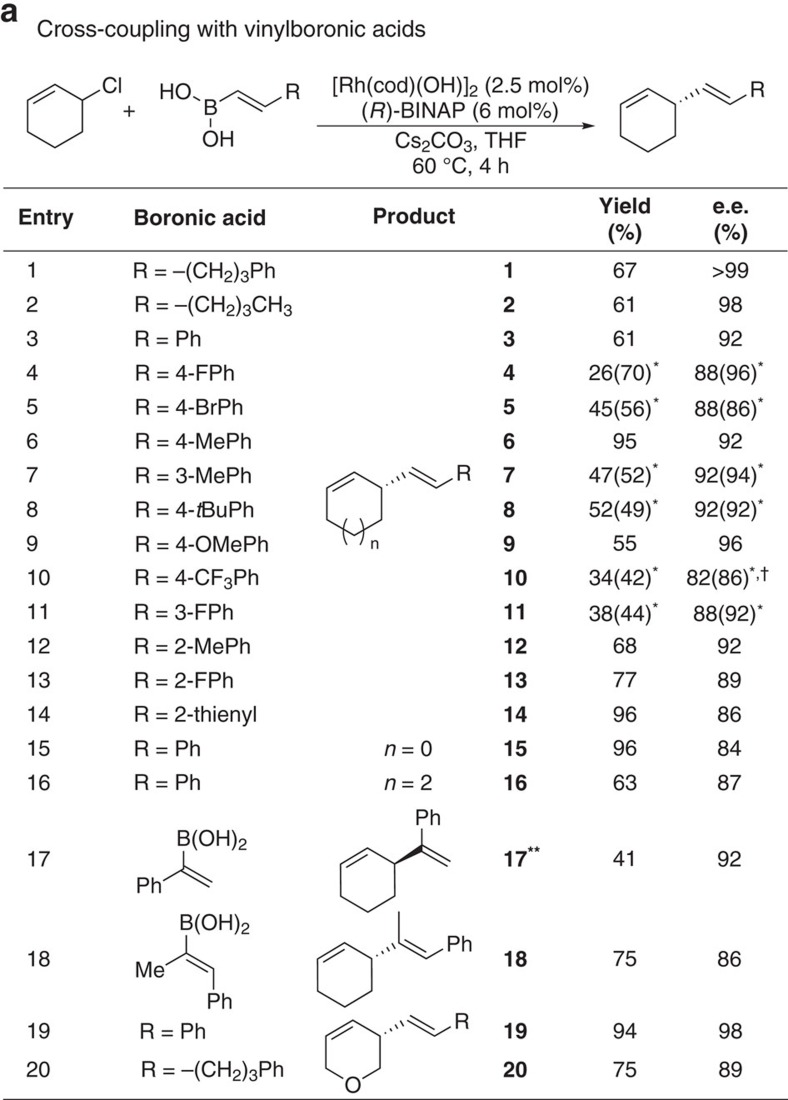


**Figure 2:**
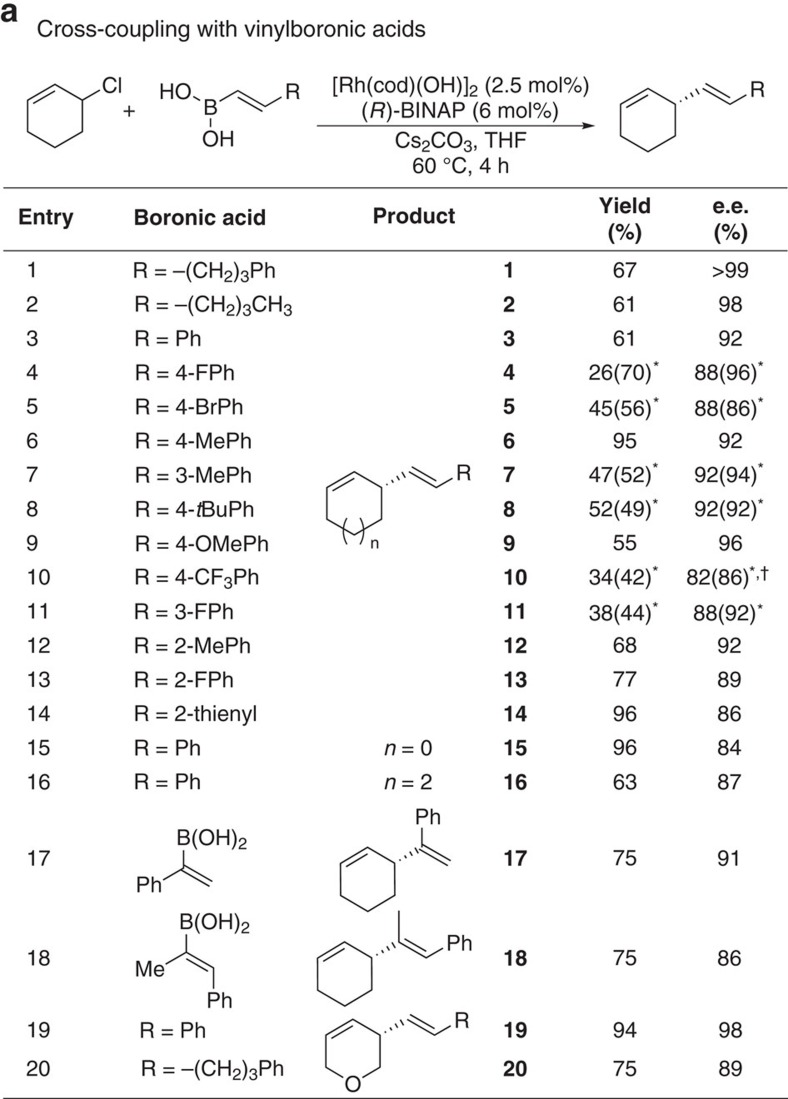


**Figure 3:**
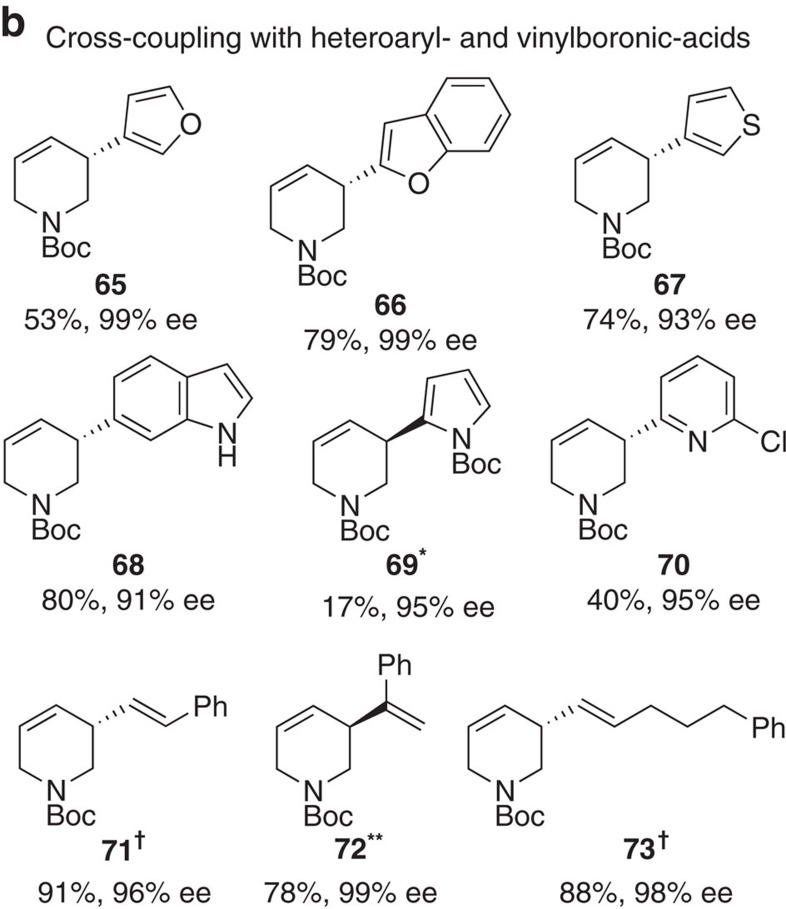


**Figure 4:**