# Supplementary material for: Asymmetric Suzuki-Miyaura coupling of heterocycles via Rhodium-catalysed allylic arylation of racemates
Source: Nat Commun. 2017 Jun 13;8:15762. doi: 10.1038/ncomms15762 (PMC5474734; doi:10.1038/ncomms15762)
Supplement: Supplementary Information — Supplementary figures, supplementary methods and supplementary references. [file ncomms15762-s1.pdf]

Supplementary methods

1. General Information

Procedures using oxygen- and/or moisture-sensitive materials were performed with anhydrous solvents (*vide infra*) under an atmosphere of anhydrous argon in flame-dried flasks, using standard Schlenk techniques. Analytical thin-layer chromatography was performed on precoated glass-backed plates (Silica Gel 60 F254; Merck) and visualised using a combination of UV light (254 nm) and aqueous ceric ammonium molybdate (CAM), aqueous basic potassium permanganate stains or vanillin solution. Flash column chromatography was carried out using Apollo Scientific silica gel 60 (0.040 – 0.063 nm), Merck 60 Å silica gel, VWR (40-63 µm) silica gel and Sigma Aldrich silica gel. Pressure was applied at the column head via a flow of nitrogen with the solvent system used in parentheses.

Reactions at 0 °C were performed using an ice-water bath, which was covered with cotton and foil if overnight stirring is required. Other temperatures were obtained using a Julabo FT902 immersion cooler or the heating plate of the stirrer.

Unless stated otherwise, solution NMR spectra were recorded at room temperature; ^{1}H and ^{13}C NMR experiments were carried out using Bruker AVG-400 (400/100 MHz), AVH-400 (400/100 MHz), AVB-400 (400/100 MHz) or AVC-500 (500/125 MHz) spectrometers. Chemical shifts are reported in ppm from the residual solvent peak. Chemical shifts (δ) are given in ppm and coupling constants (J) are quoted in hertz (Hz). Resonances are described as s (singlet), d (doublet), t (triplet), q (quartet) and m (multiplet). Assignments were made with the assistance of gCOSY, gHSQC, gHMBC or NOESY NMR spectra.

Chiral HPLC separations were achieved using an Agilent 1230 Infinity series normal phase HPLC unit and HP Chemstation software. Chiralpak® columns (250 × 4.6 mm), fitted with matching Chiralpak® Guard Cartridges (10 × 4 mm), were used as specified in the text. Solvents used were of HPLC grade (Fisher Scientific, Sigma Alrich or Rathburn); all eluent systems were isocratic.
Chiral GC measurements were conducted on a HP6890 (H_2 as vector gas) or HP6850 (H_2 as vector gas) with the stated column in the characterization. Temperature programs are described as follows: initial temperature (°C) - initial time (min) - temperature gradient (°C/min) –[certain temperature – holding time - temperature gradient (°C/min)]- final temperature (°C) – holding time. Retention times (t_R) are given in min.

Low-resolution mass spectra were recorded using a Walters LCT premier XE. High-resolution mass spectra (EI and ESI) were recorded using a Bruker MicroTOF spectrometer by the internal service at the University of Oxford.

Infrared measurements (neat, thin film) were carried out using a Bruker Tensor 27 FT-IR with internal calibration in the range 600-4000 cm^{-1}.

Optical rotations were recorded on a Perkin-Elmer 241 polarimeter at 20 °C in a 10 cm cell in the stated solvent; $[\alpha]_D$ values are given in 10^{-1} deg.cm^2 g^{-1} (concentration c given as g/100 mL).

2. General chemicals:

Dry THF and CH_2Cl_2 were collected fresh from an mBraun SPS-800 solvent purification system having been passed through anhydrous alumina columns. Dry 1,2-dichloroethane ether was purchased from Acros with an AcroSeal® respectively.

Unless stated otherwise, commercially available reagents were purchased from Sigma-Aldrich, Fisher Scientific, Apollo Scientific, Acros Organics, Strem Chemicals, Alfa Aesar or TCI UK and were used without purification. Deuterated solvents were purchased from Sigma-Aldrich (DMSO-d_6, CD_2Cl_2, $CDCl_3$).

Trans-2-phenylvinylboronic was purchased from Alfa Aesar and used without further purification. The cyclic allylic chlorides[1], 3-chloro-3,6-dihydro-2*H*-pyran[2], *N*-tert-butoxycarbonyl-5-hydroxy-3-piperidene[3] and 6-Chloropyridinylboronic acid[4] were prepared according to reported methods. 3-Bromocyclohex-1-ene was purchased from ACROS and used without further purification.

3. General procedure for the preparation of vinylic boronic acids

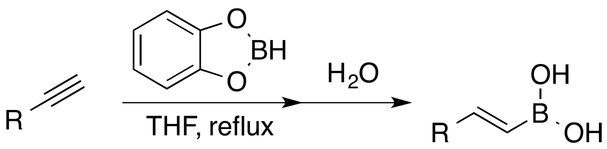

The corresponding alkyne (3.26 mmol, 1.00 eq) and catecholborane (0.4 mL, 3.91 mmol, 1.20 eq) were dissolved in THF (1.2 mL) and the mixture was refluxed for 18 h. The solvent was evaporated and then H_2O (3 mL) was added. The suspension was vigorously stirred for 4 h at room temperature. The solid was filtered and recrystallized with water. The resulting vinylboronic acid was then filtered and dried *in vacuo.*

4. Optimisation of the reaction conditions

Along with the previously reported conditions[5] which used: 1 eq of allyl chloride, 2 eq of boronic acid, 1 eq of Cs_2CO_3, 6 mol% of the ligand (*S*)-Xyl-P-PHOS and 2.5 mol% of $[Rh(cod)(OH)]_2$ in 4 mL of THF; other ligands were found to give comparable results (Scheme 1). The results depicted in blue refer to reactions performed with allyl chloride and the results in red represent the results obtained when using the allyl bromide.

5. Analytical data for figure 2

(+)-(*R,E*)-3-(5-Phenylpent-1-en-1-yl)cyclohex-2-ene (1)

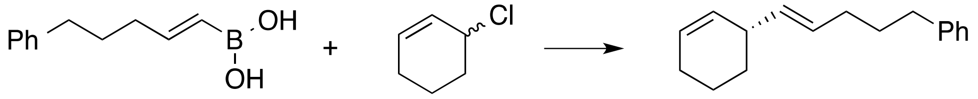

In a 10 mL round bottomed flask $[Rh(cod)(OH)]_2$ (4.6 mg, 0.01 mmol, 0.025 eq), (*R*)-BINAP (14.9 mg, 0.024 mmol, 0.06 eq) and Cs_2CO_3 (130.3 mg, 0.40 mmol, 1.00 eq) were stirred in THF (2 mL) at 60 °C for 30 min. A solution of (*E*)-(5-phenylpent-1-en-1-yl)boronic acid (152.0 mg, 0.80 mmol, 2.00 eq) and the allyl chloride (45 µL, 0.40 mmol, 1.00 eq) in THF (1.5 mL) was then added *via* syringe and the flask rinsed with THF (0.5 mL). The resulting mixture was then stirred for 4 h at 60 °C. SiO_2 (20 mg) was added and the solvent was then carefully evaporated. The resulting solid was directly loaded onto a chromatographic column and eluted with pentane to afford (+)-(*R,E*)-3-(5-phenylpent-1-en-1-yl)cyclohex-2-ene in 67% yield (60.6 mg, 0.27 mmol).

Enantiomeric excess of >99% was determined by SFC [Chiralpak® IG-3; 1500 psi, 30 °C, flow: 1.5 mL/min; 1% to 30% MeOH in 8 min; λ = 210 nm; minor enantiomer t_R = 1.88 min; major enantiomer t_R = 1.93 min].

^{1}H NMR (400 MHz, $CDCl_3$) δ 7.26 – 7.12 (m, 2H), 7.12 – 7.05 (m, 3H), 5.69 – 5.59 (m, 1H), 5.53 – 5.43 (m, 1H), 5.42 – 5.26 (m, 2H), 2.74 – 2.61 (m, 1H), 2.59 – 2.49 (m, 2H), 2.07 – 1.85 (m, 4H), 1.77 – 1.65 (m, 1H), 1.65 – 1.55 (m, 3H), 1.53 – 1.39 (m, 1H), 1.39 – 1.25 (m, 1H).

^{13}C NMR (100 MHz, $CDCl_3$) δ 142.7, 134.8, 130.4, 129.2, 128.5 (2C), 128.3 (2C), 127.4, 125.6, 38.4, 35.4, 32.1, 31.3, 29.5, 25.1, 20.6.

IR (ν_{max}/cm^{-1}): 1472, 2854, 2929.

HRMS (EI) *m/z* calc. for $C_{17}H_{22}$ $[M]^+$: 226.1721, found: 226.1723.

$[\alpha]^{20}_{589}$ = +116.3° (c 0.66, $CHCl_3$) for >99% ee.

(+)-(*R,E*)-1-(3-Cyclohexenyl)-1-hexene (2)

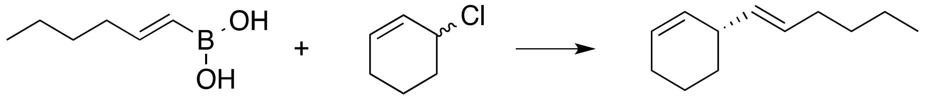

In a 10 mL round bottomed flask $[Rh(cod)(OH)]_2$ (4.6 mg, 0.01 mmol, 0.025 eq), (*R*)-BINAP (14.9 mg, 0.024 mmol, 0.06 eq) and Cs_2CO_3 (130.3 mg, 0.40 mmol, 1.00 eq) were stirred in THF (2 mL) at 60 °C for 30 min. A solution of (*E*)-hex-1-en-1-ylboronic acid (102.4 mg, 0.80 mmol, 2.00 eq) and the allyl chloride (45 µL, 0.40 mmol, 1.00 eq) in THF (1.5 mL) was then added *via* syringe and the flask rinsed with THF (0.5 mL). The resulting mixture was then stirred for 4 h at 60 °C. SiO_2 (20 mg) was added and the solvent was then carefully evaporated. The resulting solid was directly loaded onto a chromatographic column and

eluted with pentane to obtain (+)-(*R*,*E*)-1-(3-cyclohexenyl)-1-hexene in 61% yield (40.1 mg, 0.24 mmol).

Enantiomeric excess of 98% was determined by GC [Hydrodex® β-3P 60 °C 0 min then 1 °C/min to 160 °C. Minor enantiomer t_R = 35.0 min; major enantiomer t_R = 38.5 min].

^{1}H NMR (400 MHz, $CDCl_3$) δ 5.71 (dq, *J* = 9.8, 3.7, 1H), 5.62 – 5.49 (m, 1H), 5.46 – 5.32 (m, 2H), 2.72 (m, 1H), 2.11 – 1.90 (m, 5H), 1.86 – 1.63 (m, 2H), 1.59 – 1.40 (m, 1H), 1.41 – 1.24 (m, 4H), 0.89 (t, *J* = 7.0 Hz, 3H).

^{13}C NMR (101 MHz, $CDCl_3$) δ 134.3, 130.7, 129.9, 127.3, 38.5, 32.4, 31.9, 29.7, 25.2, 22.3, 20.8, 14.1.

IR (ν_{max}/cm^{-1}): 1541, 2859, 2957, 3011.

HRMS (EI) *m/z* calc. for $C_{12}H_{20}$ $[M]^+$: 164.1565, found: 164.1564.

$[\alpha]^{20}_{589}$ = +93.6° (c 1.18, $CHCl_3$) for 98% ee.

(+)-(*R*,*E*)-(2-(Cyclohex-2-en-1-yl)vinyl)benzene (3)

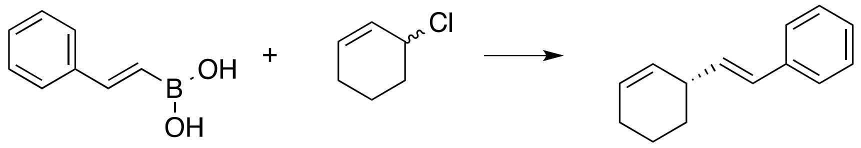

In a 10 mL round bottomed flask $[Rh(cod)(OH)]_2$ (4.6 mg, 0.01 mmol, 0.025 eq), (*R*)-BINAP (14.9 mg, 0.024 mmol, 0.06 eq) and Cs_2CO_3 (130.3 mg, 0.40 mmol, 1.00 eq) were stirred in THF (2 mL) at 60 °C for 30 min. A solution of (*E*)-styrylboronic acid (118.4 mg, 0.80 mmol, 2.00 eq) and the allyl chloride (45 µL, 0.40 mmol, 1.00 eq) in THF (1.5 mL) was then added *via* syringe and the flask rinsed with THF (0.5 mL). The resulting mixture was then stirred for 4 h at 60 °C. SiO_2 (20 mg) was added and the solvent was then carefully evaporated. The resulting solid was directly loaded onto a chromatographic column and eluted with pentane to obtain (+)-(*R*,*E*)-(2-(cyclohex-2-en-1-yl)vinyl)benzene in 61% yield (45.2 mg, 0.24 mmol).

Enantiomeric excess of 92% was determined by SFC [Chiralpak® IG-3; 1500 psi, 30°C; flow: 1.5 mL/min; from 1% to 30% MeOH in 5 min ; λ = 249 nm; major enantiomer t_R = 1.87 min; minor enantiomer t_R = 1.98 min].

^{1}H NMR (400 MHz, $CDCl_3$) δ 7.30 (d, *J* = 1.6 Hz, 1H), 7.28 (s, 1H), 7.22 (t, *J* = 7.7 Hz, 2H), 7.16 – 7.07 (m, 1H), 6.31 (d, *J* = 15.8 Hz, 1H), 6.12 (dd, *J* = 15.8, 7.4 Hz, 1H), 5.78 – 5.68 (m, 1H), 5.57 (m, 1H), 2.89 (m, 1H), 1.95 (m, 2H), 1.81 (m, 1H), 1.74 – 1.61 (m, 1H), 1.59 – 1.46 (m, 2H).

^{13}C NMR (101 MHz, $CDCl_3$) δ 137.9, 134.8, 129.6, 129.2, 128.6 (2C), 128.2, 127.0, 126.2 (2C), 38.8, 29.4, 25.2, 20.7.

IR (ATR) ν (cm^{-1}, $CHCl_3$): 1171, 1542, 2867, 3058.

HRMS (EI) *m/z* calc. for $C_{14}H_{16}$ $[M]^+$: 184.1252, found: 184.1261.

$[\alpha]^{20}_{589}$ = +18.8° (c 0.65, $CHCl_3$) for 92% ee.

(+)-(*R*,*E*)-1-(2-(Cyclohex-2-en-1-yl)vinyl)-4-fluorobenzene (4)

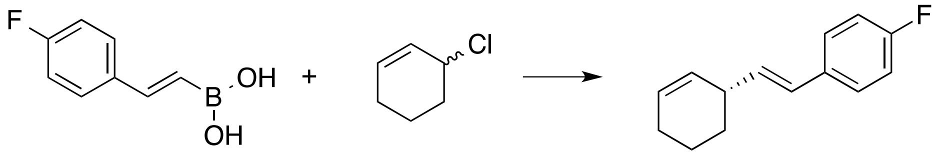

In a 10 mL round bottomed flask $[Rh(cod)(OH)]_2$ (4.6 mg, 0.01 mmol, 0.025 eq), (*R*)-Xyl-PPHOS (18.2 mg, 0.024 mmol, 0.06 eq) and Cs_2CO_3 (130.3 mg, 0.40 mmol, 1.00 eq) were stirred in THF (2 mL) at 60 °C for 30 min. A solution of (*E*)-(4-fluorostyryl)boronic acid (132.8 mg, 0.80 mmol, 2.00 eq) and the allyl chloride (45 µL, 0.40 mmol, 1.00 eq) in THF (1.5 mL) was then added *via* syringe and the flask rinsed with THF (0.5 mL). The resulting mixture was then stirred for 4 h at 60 °C. SiO_2 (20 mg) was added and the solvent was then carefully evaporated. The resulting solid was directly loaded onto a chromatographic column and eluted with pentane to obtain (+)-(*R*,*E*)-1-(2-(cyclohex-2-en-1-yl)vinyl)-4-fluorobenzene in 70% yield (57.5 mg, 0.28 mmol).

Enantiomeric excess of 88% was determined by SFC [Chiralpak® IG-3, 1500 psi, 1.5 mL/min, 30 °C; 1% to 30% MeOH in 8 min; major enantiomer t_R = 1.64 min; minor enantiomer t_R = 1.70 min].

^{1}H NMR (400 MHz, $CDCl_3$) δ 7.37 – 7.27 (m, 2H), 6.98 (t, *J* = 8.7 Hz, 2H), 6.35 (d, *J* = 15.9 Hz, 1H), 6.10 (dd, *J* = 15.9, 7.4 Hz, 1H), 5.81 (dq, *J* = 9.8, 3.3 Hz, 1H), 5.63 (dq, *J* = 10.1, 2.7 Hz, 1H), 2.95 (m, 1H), 2.03 (m, 2H), 1.94 – 1.82 (m, 1H), 1.75 (m, 1H), 1.68 – 1.45 (m, 2H).

^{13}C NMR (101 MHz, $CDCl_3$) δ 162.1 (d, *J* = 245.6 Hz), 134.6 (d, *J* = 2.2 Hz), 134.1 (d, *J* = 3.3 Hz), 129.5, 128.3, 128.0, 127.6 (d, *J* = 7.8 Hz, 2C), 115.4 (d, *J* = 21.5 Hz, 2C), 38.7, 29.4, 25.2, 20.7.

IR (ν_{max}/cm^{-1}): 1422, 2868, 3037.

HRMS (EI) *m/z* calc. for $C_{14}H_{15}F$ $[M]^+$: 202.1158, found: 202.1158.

$[\alpha]^{20}_{589}$ = +124.6° (c 2.29, $CHCl_3$) for 88% ee.

(+)-(*R*,*E*)-1-(2-(Cyclohex-2-en-1-yl)vinyl)-4-bromobenzene (5)

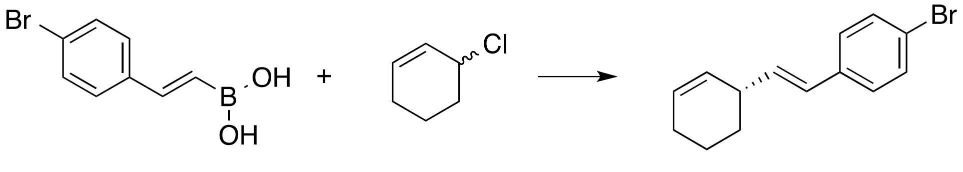

In a 10 mL round bottomed flask $[Rh(cod)(OH)]_2$ (4.6 mg, 0.01 mmol, 0.025 eq), (*R*)-BINAP (14.9 mg, 0.024 mmol, 0.06 eq) and Cs_2CO_3 (130.3 mg, 0.40 mmol, 1.00 eq) were stirred in THF (2 mL) at 60 °C for 30 min. A solution of (*E*)-(4-bromostyryl)boronic acid (181.4 mg,

0.80 mmol, 2.00 eq) and the allyl chloride (45 µL, 0.40 mmol, 1.00 eq) in THF (1.5 mL) was then added *via* syringe and the flask rinsed with THF (0.5 mL). The resulting mixture was then stirred for 4 h at 60 °C. SiO_2 (20 mg) was added and the solvent was then carefully evaporated. The resulting solid was directly loaded onto a chromatographic column and eluted with pentane to obtain (+)-(*R*,*E*)-1-(2-(cyclohex-2-en-1-yl)vinyl)-4-bromobenzene in 45% yield (47.3 mg, 0.18 mmol).

Enantiomeric excess of 88% was determined by SFC [Chiralpak® IG-3; 1500 psi, 30 °C; flow: 1.5 mL/min; 1% to 10% ACN in CO_2 in 4 min; λ = 254 nm; major enantiomer t_R = 3.50 min; minor enantiomer t_R = 3.60 min].

^{1}H NMR (400 MHz, $CDCl_3$) δ 7.41 (d, *J* = 8.4 Hz, 2H), 7.22 (d, *J* = 8.5 Hz, 2H), 6.32 (d, *J* = 16.0 Hz, 1H), 6.18 (dd, *J* = 15.9, 7.3 Hz, 1H), 5.81 (dq, *J* = 9.8, 2.2 Hz, 1H), 5.62 (dq, *J* = 10.1, 2.5 Hz, 1H), 3.00 – 2.89 (m, 1H), 2.03 (m, 2H), 1.87 (m, 1H), 1.73 (m, 1H), 1.69 – 1.44 (m, 2H).

^{13}C NMR (101 MHz, $CDCl_3$) δ 136.9, 135.7, 131.7 (2C), 129.3, 128.5, 128.1, 127.7 (2C), 120.7, 38.8, 29.3, 25.2, 20.6.

IR (ν_{max}/cm^{-1}): 1550, 2861, 2927, 3022.

HRMS (EI) *m/z* calc. for $C_{14}H_{15}Br$ $[M]^+$: 262.0357, found: 262.0352.

$[\alpha]^{20}_{589}$ = +72.4° (c 1.53, $CHCl_3$) for 84% ee.

(+)-(*R*,*E*)-1-(2-(Cyclohex-2-en-1-yl)vinyl)-4-methylbenzene (6)

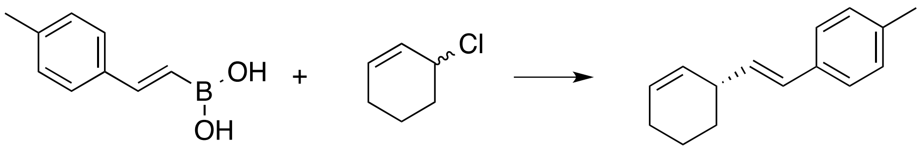

In a 10 mL round bottomed flask $[Rh(cod)(OH)]_2$ (4.6 mg, 0.01 mmol, 0.025 eq), (*R*)-BINAP (14.9 mg, 0.024 mmol, 0.06 eq) and Cs_2CO_3 (130.3 mg, 0.40 mmol, 1.00 eq) were stirred in THF (2 mL) at 60 °C for 30 min. A solution of (*E*)-(4-methylstyryl)boronic acid (129.6 mg, 0.80 mmol, 2.00 eq) and the allyl chloride (45 µL, 0.40 mmol, 1.00 eq) in THF (1.5 mL) was then added *via* syringe and the flask rinsed with THF (0.5 mL). The resulting mixture was then stirred for 4 h at 60 °C. SiO_2 (20 mg) was added and the solvent was then carefully evaporated. The resulting solid was directly loaded onto a chromatographic column and eluted with pentane to obtain (+)-(*R*,*E*)-1-(2-(cyclohex-2-en-1-yl)vinyl)-4-methylbenzene in 95% yield (75.0 mg, 0.38 mmol).

Enantiomeric excess of 92% was determined by SFC [Chiralpak® IG-3; 1500 psi, 30 °C; flow: 1.5 mL/min; 1% to 30% MeOH in CO_2 in 11 min; λ = 254 nm; major enantiomer t_R = 2.27 min; minor enantiomer t_R = 2.35 min].

^{1}H NMR (400 MHz, $CDCl_3$) δ 7.21 – 7.14 (m, 2H), 7.02 (d, *J* = 7.7 Hz, 2H), 6.27 (d, *J* = 15.9 Hz, 1H), 6.10 – 6.00 (m, 1H), 5.76 – 5.66 (m, 1H), 5.56 (m, 1H), 2.87 (m, 1H), 2.24 (s, 3H), 1.94 (m, 2H), 1.86 – 1.73 (m, 1H), 1.66 (m, 1H), 1.47 (m, 2H).

^{13}**C NMR** (101 MHz, $CDCl_3$) δ 136.7, 135.2, 133.8, 129.8, 129.3 (2C), 129.0, 128.1, 126.1 (2C), 38.8, 29.5, 25.3, 21.3, 20.7.

IR ($ν_{max}/cm^{-1}$): 1541, 2864, 2925, 3017.

HRMS (EI) *m/z* calc. for $C_{15}H_{18}$ $[M]^+$: 198.1409, found: 198.1413.

$[\alpha]^{20}_{589}$ = +91.6° (c 1.03, $CHCl_3$) for 88% ee.

(+)-(*R*,*E*)-1-(2-(Cyclohex-2-en-1-yl)vinyl)-3-methylbenzene (7)

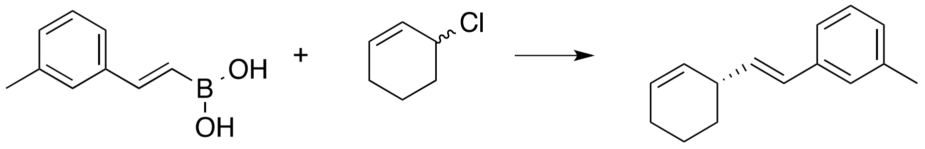

In a 10 mL round bottomed flask $[Rh(cod)(OH)]_2$ (4.6 mg, 0.01 mmol, 0.025 eq), (*R*)-BINAP (14.9 mg, 0.024 mmol, 0.06 eq) and Cs_2CO_3 (130.3 mg, 0.40 mmol, 1.00 eq) were stirred in THF (2.0 mL) at 60 °C for 30 min. A solution of (*E*)-(3-methylstyryl)boronic acid (129.6 mg, 0.80 mmol, 2.00 eq) and the allyl chloride (45 µL, 0.40 mmol, 1.00 eq) in THF (1.5 mL) was then added *via* syringe and the flask rinsed with THF (0.5 mL). The resulting mixture was then stirred for 4 h at 60 °C. SiO_2 (20 mg) was added and the solvent was then carefully evaporated. The resulting solid was directly loaded onto a chromatographic column and eluted with pentane to obtain (+)-(*R*,*E*)-1-(2-(cyclohex-2-en-1-yl)vinyl)-3-methylbenzene in 47% yield (35.3 mg, 0.19 mmol).

Enantiomeric excess of 92% was determined by SFC [Chiralpak® IG-3; 1.5mL/min; 1500psi, 30°C, 1% to 30% MeOH in CO_2 in 5 min; λ = 249 nm; major enantiomer t_R = 1.99 min; minor enantiomer t_R = 2.12 min].

^{1}**H NMR** (400 MHz, $CDCl_3$) δ 7.26 – 7.13 (m, 3H), 7.08 – 6.99 (m, 1H), 6.37 (d, *J* = 15.9 Hz, 1H), 6.19 (dd, *J* = 15.9, 7.4 Hz, 1H), 5.81 (m, 1H), 5.70 – 5.60 (m, 1H), 2.96 (m, 1H), 2.35 (s, 3H), 2.04 (m, 3H), 1.95 – 1.70 (m, 1H), 1.68 – 1.46 (m, 2H).

^{13}**C NMR** (101 MHz, $CDCl_3$) δ 138.1, 137.9, 134.7, 129.7, 129.2, 128.5, 128.2, 127.8, 126.9, 123.4, 38.8, 29.4, 25.3, 21.6, 20.7.

IR ($ν_{max}/cm^{-1}$): 1510, 2859, 2926, 3036.

HRMS (EI) *m/z* calc. for $C_{15}H_{18}$ $[M]^+$: 198.1409, found: 198.1412.

$[\alpha]^{20}_{589}$ = +99.0° (c 0.60, $CHCl_3$) for 90% ee.

(+)-(*R*,*E*)-1-(*tert*-Butyl)-4-(2-(cyclohex-2-en-1-yl)vinyl)benzene (8)

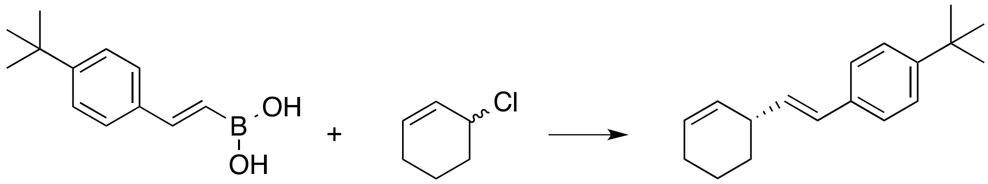

In a 10 mL round bottomed flask $[Rh(cod)(OH)]_2$ (4.6 mg, 0.01 mmol, 0.025 eq), (*R*)-Xyl-PPHOS (18.2 mg, 0.024 mmol, 0.06 eq) and Cs_2CO_3 (130.3 mg, 0.40 mmol, 1.00 eq) were stirred in THF (2.0 mL) at 60 °C for 30 min. A solution of (*E*)-(4-(*tert*-butyl)styryl)boronic acid (163.3 mg, 0.80 mmol, 2.00 eq) and the allyl chloride (45 µL, 0.40 mmol, 1.00 eq) in THF (1.5 mL) was then added *via* syringe and the flask rinsed with THF (0.5 mL). The resulting mixture was then stirred for 4 h at 60 °C. SiO_2 (20 mg) was added and the solvent was then carefully evaporated. The resulting solid was directly loaded onto a chromatographic column and eluted with pentane to obtain (+)-(*R*,*E*)-1-(*tert*-butyl)-4-(2-(cyclohex-2-en-1-yl)vinyl)benzene in 49% yield (46.9 mg, 0.20 mmol).

Enantiomeric excess of 92% was determined by HPLC [Chiralpak® IA; flow: 0.7 mL/min; hexane; λ = 210 nm; major enantiomer t_R = 8.8 min; minor enantiomer t_R = 10.1 min].

^{1}H NMR (400 MHz, $CDCl_3$) δ 7.41 – 7.28 (m, 4H), 6.39 (dd, *J* = 15.9, 1.1 Hz, 1H), 6.17 (dd, *J* = 15.9, 7.4 Hz, 1H), 5.93 – 5.75 (m, 1H), 5.66 (m, 1H), 2.97 (d, *J* = 7.6 Hz, 1H), 2.04 (dt, *J* = 6.1, 3.1 Hz, 2H), 1.97 – 1.84 (m, 1H), 1.76 (t, *J* = 11.9 Hz, 1H), 1.66 – 1.47 (m, 2H), 1.34 (s, 9H).

^{13}C NMR (101 MHz, $CDCl_3$) δ 150.1, 135.2, 134.1, 129.8, 128.9, 128.1, 125.9 (2C), 125.7 (2C), 125.5, 38.8, 34.7, 31.5, 31.4, 29.5, 25.3, 20.7.

IR (ν_{max}/cm^{-1}): 1541, 2859, 2926, 3034.

HRMS (EI) *m/z* calc. for $C_{18}H_{24}$ $[M]^+$: 240.1878, found: 240.1877.

$[\alpha]^{20}_{589}$ = +58.0° (c 0.80, $CHCl_3$) for 84% ee.

(+)-(*R*,*E*)-1-(2-(Cyclohex-2-en-1-yl)vinyl)-4-methoxybenzene (9)

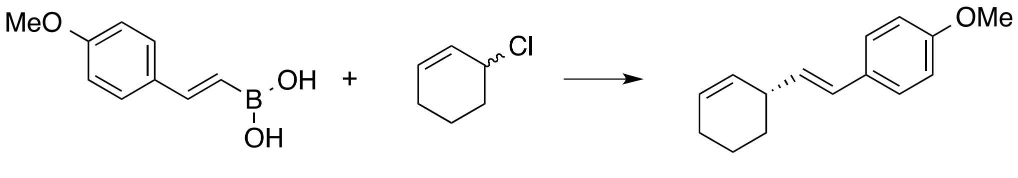

In a 10 mL round bottomed flask $[Rh(cod)(OH)]_2$ (4.6 mg, 0.01 mmol, 0.025 eq), (*R*)-BINAP (14.9 mg, 0.024 mmol, 0.06 eq) and Cs_2CO_3 (130.3 mg, 0.40 mmol, 1.00 eq) were stirred in THF (2.0 mL) at 60 °C for 30 min. A solution of (*E*)-(4-methoxystyryl)boronic acid (142.4 mg, 0.80 mmol, 2.00 eq) and the allyl chloride (45 µL, 0.40 mmol, 1.00 eq) in THF (1.5 mL) was then added *via* syringe and the flask rinsed with THF (0.5 mL). The resulting mixture was then stirred for 4 h at 60 °C. SiO_2 (20 mg) was added and the solvent was then carefully evaporated. The resulting solid was directly loaded onto a chromatographic column and

eluted with pentane to obtain (+)-(*R*,*E*)-1-(2-(cyclohex-2-en-1-yl)vinyl)-4-methoxybenzene in 55% yield (47.1 mg, 0.22 mmol).

Enantiomeric excess of 96% was determined by SFC [Chiralpak® IG-3; 1500 psi, 30 °C, flow: 1.0 mL/min; 100% CO_2; λ = 254 nm; major enantiomer t_R = 14.4 min; minor enantiomer t_R = 17.9 min].

^{1}H NMR (500 MHz, $CDCl_3$) δ 7.24 – 7.19 (m, 2H), 6.76 (d, *J* = 8.7 Hz, 2H), 6.25 (dd, *J* = 15.9, 1.1 Hz, 1H), 5.97 (m, 1H), 5.70 (m, 1H), 5.57 (d, *J* = 2.6 Hz, 1H), 3.72 (s, 3H), 2.85 (m, 1H), 1.94 (m, 2H), 1.80 (m, 1H), 1.72 – 1.59 (m, 1H), 1.62 – 1.55 (m, 1H), 1.57 – 1.37 (m, 1H).

^{13}C NMR (126 MHz, $CDCl_3$) δ 158.9, 132.7, 130.8, 129.9, 128.5, 128.1, 127.3 (2C), 114.1 (2C), 55.4, 38.8, 29.6, 25.3, 20.7.

IR ($ν_{max}/cm^{-1}$): 1541, 2834, 2927, 3016.

HRMS (EI) *m/z* calc. for $C_{15}H_{18}O$ $[M]^+$: 214.1358, found: 214.1353.

$[α]^{20}_{589}$ = +67.4° (c 0.60, $CHCl_3$) for 96% ee.

(+)-(*R*,*E*)-1-(2-(Cyclohex-2-en-1-yl)vinyl)-4-(trifluoromethyl)benzene (10)

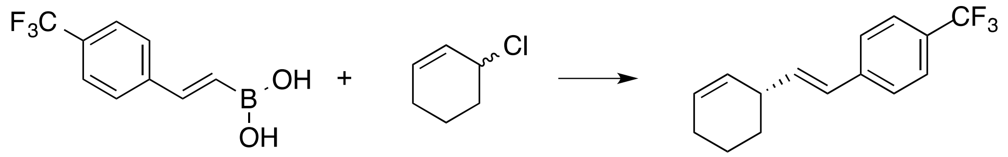

In a 10 mL round bottomed flask $[Rh(cod)(OH)]_2$ (4.6 mg, 0.01 mmol, 0.025 eq), (*R*)-BINAP (14.9 mg, 0.024 mmol, 0.06 eq) and Cs_2CO_3 (130.3 mg, 0.40 mmol, 1.00 eq) were stirred in THF (2.0 mL) at 60 °C for 30 min. A solution of (*E*)-(4-(trifluoromethyl)styryl)boronic acid (172.8 mg, 0.80 mmol, 2.00 eq) and the allyl chloride (45 µL, 0.40 mmol, 1.00 eq) in THF (1.5 mL) was then added *via* syringe and the flask rinsed with THF (0.5 mL). The resulting mixture was then stirred for 4 h at 60 °C. SiO_2 (20 mg) was added and the solvent was then carefully evaporated. The resulting solid was directly loaded onto a chromatographic column and eluted with pentane to obtain (+)-(*R*,*E*)-1-(2-(cyclohex-2-en-1-yl)vinyl)-4-(trifluoromethyl)-benzene in 34% yield (34.3 mg, 0.14 mmol).

Enantiomeric excess of about 82% (±10% ee) was determined by HPLC [Chiralpak® ID; flow: 0.2 mL/min; hexane; λ = 210 nm; major enantiomer t_R = 24.3 min; minor enantiomer t_R = 25.7 min].

^{1}H NMR (400 MHz, $CDCl_3$) δ 7.54 (d, *J* = 8.2 Hz, 2H), 7.44 (d, *J* = 8.1 Hz, 2H), 6.41 (d, *J* = 15.9 Hz, 1H), 6.29 (dd, *J* = 15.9, 7.2 Hz, 1H), 5.83 (m, 1H), 5.63 (m, 1H), 2.99 (m, 1H), 2.04 (m, 2H), 1.90 (m, 1H), 1.75 (m, 1H), 1.69 – 1.46 (m, 2H).

^{13}C NMR (101 MHz, $CDCl_3$) δ 141.5, 137.6, 129.0, 128.7, 128.1, 126.3 (2C), 125.56 (q, *J* = 3.9 Hz, 2C), 124.5 (d, *J* = 271.9 Hz, 1C), 123.0, 38.8, 29.2, 25.2, 20.6.

IR (ν_{max}/cm^{-1}): 1541, 2859, 2926, 3033.

HRMS (EI) *m/z* calc. for $C_{15}H_{15}F_3$ $[M]^+$: 252.1126, found: 252.1158.

$[\alpha]^{20}_{589}$ = +112.7° (c 0.80, $CHCl_3$) for 82% (±10%) ee.

(+)-(*R*,*E*)-1-(2-(Cyclohex-2-en-1-yl)vinyl)-3-fluorobenzene (11)

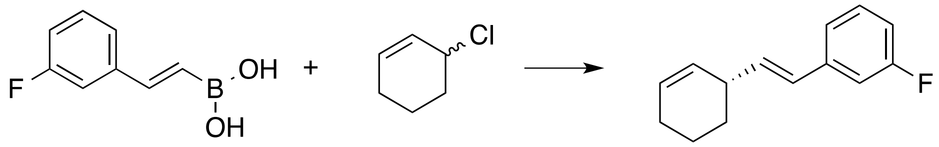

In a 10 mL round bottomed flask $[Rh(cod)(OH)]_2$ (4.6 mg, 0.01 mmol, 0.025 eq), (*R*)-BINAP (14.9 mg, 0.024 mmol, 0.06 eq) and Cs_2CO_3 (130.3 mg, 0.40 mmol, 1.00 eq) were stirred in THF (2.0 mL) at 60 °C for 30 min. A solution of (*E*)-(3-fluorostyryl)boronic acid (132.8 mg, 0.80 mmol, 2.00 eq) and the allyl chloride (45 µL, 0.40 mmol, 1.00 eq) in THF (1.5 mL) was then added *via* syringe and the flask rinsed with THF (0.5 mL). The resulting mixture was then stirred for 4 h at 60 °C. SiO_2 (20 mg) was added and the solvent was then carefully evaporated. The resulting solid was directly loaded onto a chromatographic column and eluted with pentane to obtain (+)-(*R*,*E*)-1-(2-(cyclohex-2-en-1-yl)vinyl)-3-fluorobenzene in 38% yield (30 mg, 0.15 mmol).

Enantiomeric excess of 88% was determined by SFC [Chiralpak® IG-3; 1500 psi, 30 °C; flow: 1.5 mL/min; 1% to 30% MeOH in CO_2 in 5 min; λ = 254 nm; major enantiomer t_R = 1.73 min; minor enantiomer t_R = 1.79 min].

^{1}H NMR (500 MHz, $CDCl_3$) δ 7.17 (td, *J* = 7.9, 6.0 Hz, 1H), 7.06 – 6.95 (m, 2H), 6.81 (td, *J* = 8.4, 2.5 Hz, 1H), 6.27 (d, *J* = 15.8 Hz, 1H), 6.13 (dd, *J* = 15.9, 7.3 Hz, 1H), 5.74 (m, 1H), 5.55 (m, 1H), 2.88 (m, 1H), 1.96 (m, 2H), 1.81 (m, 1H), 1.66 (m, 1H), 1.58 – 1.38 (m, 2H).

^{13}C NMR (126 MHz, $CDCl_3$) 163.3 (d, *J* = 244.8 Hz), 140.4 (d, *J* = 7.7 Hz), 136.3, 130.0 (d, *J* = 8.6 Hz), 129.2, 128.5, 128.2 (d, *J* = 2.6 Hz), 122.1 (d, *J* = 2.7 Hz), 113.8 (d, *J* = 21.4 Hz), 112.6 (d, *J* = 21.7 Hz), 38.7, 29.3, 25.2, 20.6.

IR (ν_{max}/cm^{-1}): 1584, 2860, 2927, 3018.

HRMS (EI) *m/z* calc. for $C_{14}H_{15}F$ $[M]^+$: 202.1158, found: 202.1151.

$[\alpha]^{20}_{589}$ = +93.0° (c 1.24, $CHCl_3$) for 88% ee.

(+)-(*R*,*E*)-1-(2-(Cyclohex-2-en-1-yl)vinyl)-2-methylbenzene (12)

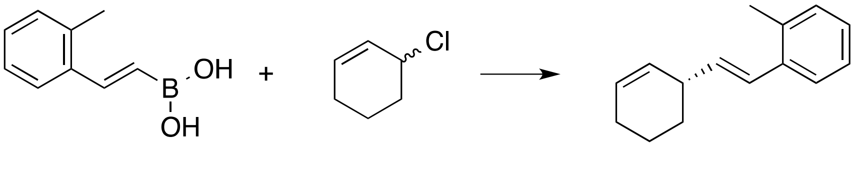

In a 10 mL round bottomed flask $[Rh(cod)(OH)]_2$ (4.6 mg, 0.01 mmol, 0.025 eq), (*R*)-BINAP (14.9 mg, 0.024 mmol, 0.06 eq) and Cs_2CO_3 (130.3 mg, 0.40 mmol, 1.00 eq) were stirred in THF (2.0 mL) at 60 °C for 30 min. A solution of (2-methylstyryl)boronic acid (129.6 mg, 0.80 mmol, 2.00 eq) and the allyl chloride (45 µL, 0.40 mmol, 1.00 eq) in THF (1.5 mL) was then added *via* syringe and the flask rinsed with THF (0.5 mL). The resulting mixture was then stirred for 4 h at 60 °C. SiO_2 (20 mg) was added and the solvent was then carefully evaporated. The resulting solid was directly loaded onto a chromatographic column and eluted with pentane to obtain (+)-(*R*,*E*)-1-(2-(cyclohex-2-en-1-yl)vinyl)-2-methylbenzene in 68% yield (54.0 mg, 0.27 mmol).

Enantiomeric excess of 92% was determined by SFC [Chiralpak® IA-3; 2000 psi, 30°C, flow: 0.3 mL/min; 100% CO_2; λ = 254 nm; major enantiomer t_R = 14.9 min; minor enantiomer t_R = 17.20 min].

^{1}H NMR (400 MHz, $CDCl_3$) δ 7.17 – 7.04 (m, 3H), 6.99 – 6.90 (m, 1H), 6.27 (dd, *J* = 15.9, 1.1 Hz, 1H), 6.10 (dd, *J* = 15.8, 7.4 Hz, 1H), 5.72 (dtd, *J* = 9.8, 3.7, 2.1 Hz, 1H), 5.56 (dq, *J* = 10.1, 2.4 Hz, 1H), 2.87 (tt, *J* = 7.0, 3.5 Hz, 1H), 2.25 (s, 3H), 1.95 (tq, *J* = 5.8, 2.7 Hz, 2H), 1.86 – 1.74 (m, 1H), 1.67 (m, 1H), 1.47 (m, 2H).

^{13}C NMR (101 MHz, $CDCl_3$) δ 138.1, 137.9, 134.6, 129.7, 129.2, 128.5, 128.2, 127.8, 126.9, 123.3, 38.8, 29.4, 25.2, 21.6, 20.7.

IR (ν_{max}/cm^{-1}): 1446, 1488, 1584, 1603, 2858, 2926, 3019.

HRMS (EI) *m/z* calc. for $C_{15}H_{18}$ $[M]^+$: 198.1409, found: 198.1412.

$[\alpha]^{25}_{589}$ = +208.1° (c 2.08, $CHCl_3$) for 92% ee.

(+)-(*R*,*E*)-1-(2-(Cyclohex-2-en-1-yl)vinyl)-2-fluorobenzene (13)

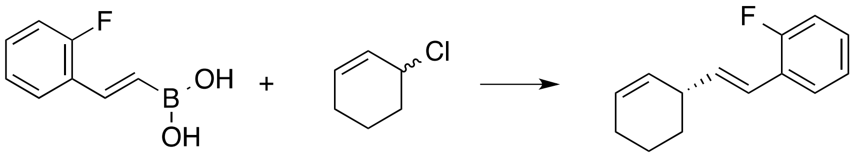

In a 10 mL round bottomed flask $[Rh(cod)(OH)]_2$ (4.6 mg, 0.01 mmol, 0.025 eq), (*R*)-BINAP (14.9 mg, 0.024 mmol, 0.06 eq) and Cs_2CO_3 (130.3 mg, 0.40 mmol, 1.00 eq) were stirred in THF (2.0 mL) at 60 °C for 30 min. A solution of (2-fluorostyryl)boronic acid (129.6 mg, 0.80 mmol, 2.00 eq) and the allyl chloride (45 µL, 0.40 mmol, 1.00 eq) in THF (1.5 mL) was then added *via* syringe and the flask rinsed with THF (0.5 mL). The resulting mixture was then stirred for 4 h at 60 °C. SiO_2 (20 mg) was added and the solvent was then carefully evaporated. The resulting solid was directly loaded onto a chromatographic column and eluted with pentane to obtain (+)-(*R*,*E*)-1-(2-(cyclohex-2-en-1-yl)vinyl)-2-fluorobenzene in 77% yield (61.2 mg, 0.31 mmol).

Enantiomeric excess of 89% was determined by SFC [Chiralpak® IG-3; 1500 psi, 30 °C; flow: 1.5 mL/min; 1% to 30% MeOH in CO_2 in 8 min; λ = 254 nm; major enantiomer t_R = 1.52 min; minor enantiomer t_R = 1.57 min].

^{1}H NMR (400 MHz, $CDCl_3$) δ 7.45 (td, *J* = 7.7, 1.8 Hz, 1H), 7.16 (tdd, *J* = 7.3, 5.1, 1.8 Hz, 1H), 7.11 – 6.96 (m, 2H), 6.55 (d, *J* = 16.0 Hz, 1H), 6.27 (ddd, *J* = 16.0, 7.5, 1.6 Hz, 1H), 5.82 (m, 1H), 5.65 (m, 1H), 2.99 (m, 1H), 2.04 (m, 2H), 1.96 – 1.84 (m, 1H), 1.76 (m, 1H), 1.68 – 1.46 (m, 2H).

^{13}C NMR (101 MHz, $CDCl_3$) δ 160.1 (d, *J* = 248.3 Hz), 137.4 (d, *J* = 4.3 Hz), 129.4, 128.4, 128.2 (d, *J* = 8.4 Hz), 127.2 (d, , *J* = 3.5 Hz), 125.7 (d, *J* = 12.3 Hz), 124.1 (d, *J* = 3.5 Hz), 121.5 (d, *J* = 3.8 Hz), 115.7 (d, *J* = 22.3 Hz), 39.2, 29.3, 25.2, 20.7.

IR (ν_{max}/cm^{-1}): 1455, 1487, 1579, 2859, 2930, 3020.

HRMS (EI) *m/z* calc. for $C_{14}H_{15}F$ $[M]^+$: 202.1158, found: 202.1156.

$[\alpha]^{25}_{589}$ = +175.6° (c 1.47, $CHCl_3$) for 89% ee.

(+)-(*R*,*E*)-2-(2-(Cyclohex-2-en-1-yl)vinyl)thiophene (14)

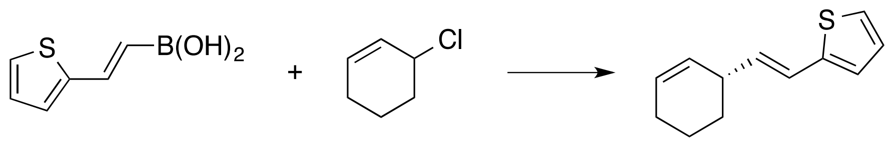

In a 10 mL round bottomed flask $[Rh(cod)(OH)]_2$ (2.3 mg, 0.005 mmol, 0.025 eq), (*R*)-BINAP (7.5 mg, 0.012 mmol, 0.06 eq) and Cs_2CO_3 (65.2 mg, 0.20 mmol, 1.00 eq) were stirred in THF (1.0 mL) at 60 °C for 30 min. A solution of (*E*)-(2-(thiophen-2-yl)vinyl)boronic acid (61.6 mg, 0.40 mmol, 2.00 eq) and the allyl chloride (23 µL, 0.20 mmol, 1.00 eq) in THF (0.5 mL) was then added *via* syringe and the flask rinsed with THF (0.5 mL). The resulting mixture was then stirred for 4 h at 60 °C. SiO_2 (20 mg) was added and the solvent was then carefully evaporated. The resulting solid was directly loaded onto a chromatographic column and eluted with pentane to obtain (+)-(*R*,*E*)-2-(2-(cyclohex-2-en-1-yl)vinyl)thiophene in 96% yield (37.0 mg, 0.19 mmol).

Enantiomeric excess of 86% was determined by SFC [Chiralpak® IG-3; 1500 psi, 30 °C; flow: 1.5 mL/min; 1% to 50% MeOH in CO_2 in 9 min; λ = 254 nm; major enantiomer t_R = 2.05 min; minor enantiomer t_R = 2.15 min].

^{1}H NMR (400 MHz, $CDCl_3$) δ 7.09 (d, *J* = 5.0 Hz, 1H), 6.98 – 6.85 (m, 2H), 6.50 (dd, *J* = 15.7, 1.2 Hz, 1H), 6.04 (dd, *J* = 15.7, 7.4 Hz, 1H), 5.80 (dq, *J* = 9.8, 2.1 Hz, 1H), 5.62 (dq, *J* = 10.0, 2.4 Hz, 1H), 2.96 – 2.88 (m, 1H), 2.01 (m, 2H), 1.92 – 1.80 (m, 1H), 1.72 (m, 1H), 1.54 (m, 2H).

^{13}C NMR (101 MHz, $CDCl_3$) δ 143.2, 134.7, 129.2, 128.5, 127.4, 124.6, 123.4, 122.6, 38.5, 29.2, 25.2, 20.5.

IR (ν_{max}/cm^{-1}): 1487, 1580, 1722, 2866, 2936, 3019.

HRMS (EI) *m/z* calc. for $C_{12}H_{14}S$ $[M]^+$: 190.0816, found: 190.0809.

$[\alpha]^{25}_{589}$ = + 62.9° (c 0.34, $CHCl_3$) for 86% ee.

(+)-(*R*,*E*)-(2-(Cyclopent-2-en-1-yl)vinyl)benzene (15)

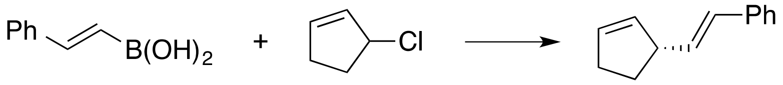

In a 10 mL round bottomed flask $[Rh(cod)(OH)]_2$ (4.6 mg, 0.01 mmol, 0.025 eq), (*R*)-BINAP (14.9 mg, 0.024 mmol, 0.06 eq) and Cs_2CO_3 (130.3 mg, 0.40 mmol, 1.00 eq) were stirred in THF (2.0 mL) at 60 °C for 30 min. A solution of (*E*)-styrylboronic acid (118.4 mg, 0.80 mmol, 2.00 eq) and the allyl chloride (41.0 mg, 0.40 mmol, 1.00 eq) in THF (1.0 mL) was then added *via* syringe and the flask rinsed with THF (1.0 mL). The resulting mixture was then stirred for 4 h at 60 °C. SiO_2 (20 mg) was added and the solvent was then carefully evaporated. The resulting solid was directly loaded onto a chromatographic column and eluted with pentane to obtain (+)-(*R*,*E*)-(2-(cyclopent-2-en-1-yl)vinyl)benzene in 96% yield (65.2 mg, 0.38 mmol).

Enantiomeric excess of 84% was determined by SFC [Chiralpak® IG-3; 1500 psi, 30 °C; flow: 1.5 mL/min; 15 to 50% MeOH in 11 min; λ = 254 nm; major enantiomer t_R = 1.45 min; minor enantiomer t_R = 1.51 min].

^{1}H NMR (400 MHz, $CDCl_3$) δ 7.31 – 7.16 (m, 4H), 7.16 – 7.05 (m, 1H), 6.30 (d, *J* = 15.8 Hz, 1H), 6.08 (dd, *J* = 15.8, 8.0 Hz, 1H), 5.77 (dq, *J* = 4.7, 2.3 Hz, 1H), 5.60 (dq, *J* = 5.8, 2.1 Hz, 1H), 3.46 – 3.34 (m, 1H), 2.43 – 2.19 (m, 2H), 2.18 – 2.04 (m, 1H), 1.65 – 1.52 (m, 1H).

^{13}C NMR (101 MHz, $CDCl_3$) δ 137.9, 134.6, 133.7, 131.9, 128.6 (2C), 128.5, 127.0, 126.2 (2C), 49.0, 32.4, 30.9.

IR (ν_{max}/cm^{-1}): 1449, 1494, 2851, 2936, 3028.

HRMS (EI) *m/z* calc. for $C_{13}H_{14}$ $[M]^+$: 170.1096, found: 170.1087.

$[\alpha]^{25}_{589}$ = +46.3° (c 1.37, $CHCl_3$) for 84% ee.

(+)-(*R*,*E*)-(2-(Cyclohept-2-en-1-yl)vinyl)benzene (16)

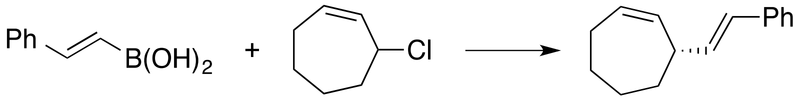

In a 10 mL round bottomed flask $[Rh(cod)(OH)]_2$ (4.6 mg, 0.01 mmol, 0.025 eq), (*R*)-BINAP (14.9 mg, 0.024 mmol, 0.06 eq) and Cs_2CO_3 (130.3 mg, 0.40 mmol, 1.00 eq) were stirred in THF (2.0 mL) at 60 °C for 30 min. A solution of (*E*)-styrylboronic acid (118.4 mg, 0.80 mmol, 2.00 eq) and the allyl chloride (52.2 mg, 0.40 mmol, 1.00 eq) in THF (1.0 mL) was then added *via* syringe and the flask rinsed with THF (1.0 mL). The resulting mixture was then stirred for 4 h at 60 °C. SiO_2 (20 mg) was added and the solvent was then carefully evaporated. The resulting solid was directly loaded onto a chromatographic column and eluted with pentane to obtain (+)-(*R*,*E*)-(2-(cyclohept-2-en-1-yl)vinyl)benzene in 63% yield (50.2 mg, 0.25 mmol).

Enantiomeric excess of 87% was determined by HPLC [Chiralpak® ID; flow: 0.4 mL/min; hexane; λ = 210 nm; minor enantiomer t_R = 11.9 min; major enantiomer t_R = 13.1 min].

^{1}H NMR (400 MHz, $CDCl_3$) δ 7.32 – 7.17 (m, 4H), 7.19 – 7.05 (m, 1H), 6.32 (d, *J* = 15.9 Hz, 1H), 6.20 (dd, *J* = 15.9, 7.5 Hz, 1H), 5.83 – 5.72 (m, 1H), 5.62 (dd, *J* = 11.7, 5.7 Hz, 1H), 3.11 – 3.01 (m, 1H), 2.09 (m, 2H), 1.92 – 1.79 (m, 1H), 1.76 – 1.64 (m, 1H), 1.64 – 1.51 (m, 2H), 1.54 – 1.43 (m, 1H), 1.45 – 1.31 (m, 1H).

^{13}C NMR (101 MHz, $CDCl_3$) δ 138.0, 135.2, 135.1, 132.0, 128.6 (2C), 128.6, 127.0, 126.2 (2C), 43.7, 34.0, 29.6, 28.9, 27.2.

IR (ν_{max}/cm^{-1}): 1262, 1449, 2922, 2993.

HRMS (EI) *m/z* calc. for $C_{15}H_{18}$ $[M]^+$: 198.1409, found: 198.1397.

$[\alpha]^{25}_{589}$ = +51.2° (c 0.53, $CHCl_3$) for 87% ee.

(–)-(*S*)-(1-(Cyclohex-2-en-1-yl)vinyl)benzene (17)

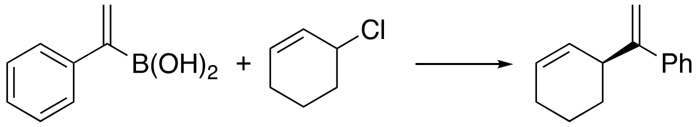

In a 10 mL round bottomed flask $[Rh(cod)(OH)]_2$ (4.6 mg, 0.01 mmol, 0.025 eq), (*S*)-BINAP (14.9 mg, 0.024 mmol, 0.06 eq) and Cs_2CO_3 (130.3 mg, 0.40 mmol, 1.00 eq) were stirred in THF (2.0 mL) at 60 °C for 30 min. Then the mixture was allow to cool to room temperature before adding a solution of (1-phenylvinyl)boronic acid (118.4 mg, 0.80 mmol, 2.00 eq) and the allyl chloride (45 µL, 0.40 mmol, 1.00 eq) in THF (1.5 mL) *via* syringe and the flask rinsed with THF (0.5 mL). The resulting mixture was then stirred for 48 h at room temperature (about 22 °C). SiO_2 (20 mg) was added and the solvent was then carefully evaporated. The resulting solid was directly loaded onto a chromatographic column and eluted with pentane to obtain (+)-(*S*)-(1-(cyclohex-2-en-1-yl)vinyl)benzene in 41% yield (28.0 mg, 0.16 mmol).

Enantiomeric excess of 92% was determined by SFC [Chiralpak® IF-3; 1500 psi, 30 °C; flow: 1.5 mL/min; 1% to 30% MeOH in CO_2 in 5 min; λ = 254 nm; minor enantiomer t_R = 1.40 min; major enantiomer t_R = 1.50 min].

^{1}H NMR (400 MHz, $CDCl_3$) δ 7.36 – 7.27 (m, 2H), 7.27 – 7.15 (m, 3H), 5.77 (m, 1H), 5.64 (m, 1H), 5.22 (s, 1H), 4.98 (t, *J* = 1.4 Hz, 1H), 3.30 (m, 1H), 2.03 – 1.89 (m, 2H), 1.79 – 1.68 (m, 1H), 1.68 – 1.32 (m, 3H).

^{13}C NMR (101 MHz, $CDCl_3$) δ 152.5, 142.0, 129.7, 128.3, 128.2 (2C), 127.2, 126.6 (2C), 113.1, 39.9, 28.4, 25.3, 20.3.

IR (ν_{max}/cm^{-1}): 1574, 1624, 2858, 2928, 3055.

HRMS (EI) *m/z* calc. for $C_{14}H_{16}$ $[M]^+$: 184.1252, found: 184.1253.

$[\alpha]^{25}_{589}$ = = –63.18° (c 0.78, $CHCl_3$) for 92% ee.

(+)-(*R*)-(1-(Cyclohex-2-en-1-yl)vinyl)benzene (17)

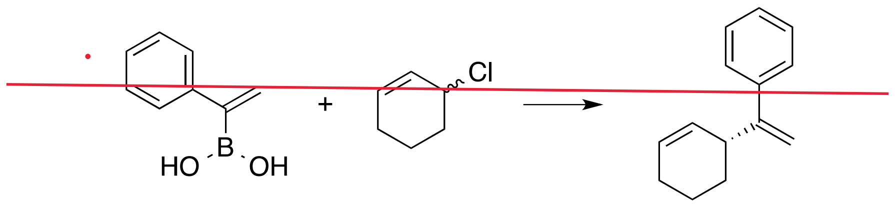

In a 10 mL round bottomed flask $[Rh(cod)(OH)]_2$ (4.6 mg, 0.01 mmol, 0.025 eq), (*R*)-BINAP (14.9 mg, 0.024 mmol, 0.06 eq) and Cs_2CO_3 (130.3 mg, 0.40 mmol, 1.00 eq) were stirred in THF (2.0 mL) at 60 °C for 30 min. A solution of (1-phenylvinyl)boronic acid (118.4 mg, 0.80 mmol, 2.00 eq) and the allyl chloride (45 µL, 0.40 mmol, 1.00 eq) in THF (1.5 mL) was then added *via* syringe and the flask rinsed with THF (0.5 mL). The resulting mixture was then stirred for 4 h at 60 °C. SiO_2 (20 mg) was added and the solvent was then carefully evaporated. The resulting solid was directly loaded onto a chromatographic column and eluted with pentane to obtain (+)-(*R*)-(1-(cyclohex-2-en-1-yl)vinyl)benzene in 75% yield (55.3 mg, 0.30 mmol).

Enantiomeric excess of 95% was determined by SFC [Chiralpak® IG-3; 1500 psi, 30 °C; flow: 1.5 mL/min; 1% to 50% MeOH in CO_2 in 11 min; λ = 254 nm; major enantiomer t_R = 1.92 min; minor enantiomer t_R = 2.06 min].

^{1}H NMR (400 MHz, $CDCl_3$) δ 7.32 – 7.27 (m, 2H), 7.27 – 7.15 (m, 2H), 7.15 – 7.05 (m, 1H), 6.35 – 6.26 (d, *J* = 15.9 Hz, 1H), 6.12 (dd, *J* = 15.9, 7.4 Hz, 1H), 5.80 – 5.68 (m, 1H), 5.57 (dd, *J* = 10.1, 2.6 Hz, 1H), 2.94 – 2.83 (m, 1H), 1.95 (dq, *J* = 5.9, 3.1 Hz, 2H), 1.89 – 1.75 (m, 1H), 1.75 – 1.60 (m, 1H), 1.60 – 1.35 (m, 2H).

^{13}C NMR (101 MHz, $CDCl_3$) δ 137.9, 134.8, 129.6, 129.2, 128.6, 128.2 (2C), 127.0, 126.2 (2C), 38.8, 29.4, 25.2, 20.7.

IR (ν_{max}/cm^{-1}): 1447, 1495, 1598, 2858, 2929, 3022.

HRMS (EI) *m/z* calc. for $C_{14}H_{16}$ $[M]^+$: 184.1252, found: 184.1250.

$[\alpha]^{25}_{589}$ = +252.4° (c 1.10, $CHCl_3$) for 91% ee.

(+)-(*R*,*E*)-(2-(Cyclohex-2-en-1-yl)prop-1-en-1-yl)benzene (18)

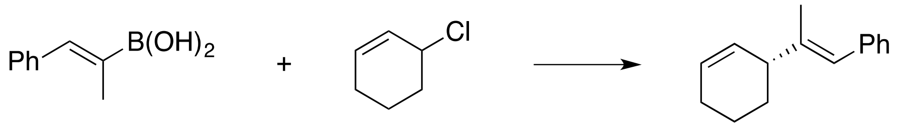

In a 10 mL round bottomed flask $[Rh(cod)(OH)]_2$ (3.4 mg, 0.007 mmol, 0.025 eq), (*R*)-BINAP (11.2 mg, 0.018 mmol, 0.06 eq) and Cs_2CO_3 (97.7 mg, 0.30 mmol, 1.00 eq) were stirred in THF (1.5 mL) at 60 °C for 30 min. A solution of (*Z*)-(1-phenylprop-1-en-2-yl)boronic acid (97.2 mg, 0.60 mmol, 2.00 eq) and the allyl bromide (35 µL, 0.30 mmol, 1.00 eq) in THF (1.0 mL) was then added *via* syringe and the flask rinsed with THF (0.5 mL). The resulting mixture was then stirred for 4 h at 60 °C. SiO_2 (20 mg) was added and the solvent was then

carefully evaporated. The resulting solid was directly loaded onto a chromatographic column and eluted with pentane to obtain (+)-(*R*,*E*)-(2-(cyclohex-2-en-1-yl)prop-1-en-1-yl)benzene in 75% yield (44.9 mg, 0.23 mmol).

Enantiomeric excess of 86% was determined by HPLC [Chiralpak® IB; flow: 1.0 mL/min; hexane; λ = 210 nm; major enantiomer t_R = 4.9 min; minor enantiomer t_R = 5.4 min].

^{1}H NMR (400 MHz, $CDCl_3$) δ 7.39 – 7.23 (m, 4H), 7.22 – 7.09 (m, 1H), 6.30 (s, 1H), 5.89 – 5.74 (m, 1H), 5.74 – 5.59 (m, 1H), 2.94 – 2.84 (m, 1H), 2.08 – 2.00 (m, 2H), 1.85 (s, 3H), 1.81 – 1.70 (m, 1H), 1.68 – 1.48 (m, 3H).

^{13}C NMR (101 MHz, $CDCl_3$) δ 142.5, 138.9, 130.1, 129.0, 128.6, 128.1, 128.0, 126.1, 126.0, 125.4, 45.6, 28.3, 25.3, 21.2, 16.7.

IR (ν_{max}/cm^{-1}): 1446, 1493, 1599, 2858, 2930.

HRMS (EI) *m/z* calc. for $C_{15}H_{18}$ $[M]^+$: 198.1409, found: 198.1412.

$[\alpha]^{25}_{589}$ = +98.1° (c 0.84, $CHCl_3$) for 86% ee.

(+)-(*S*,*E*)-3-Styryl-3,6-dihydro-2*H*-pyran (19)

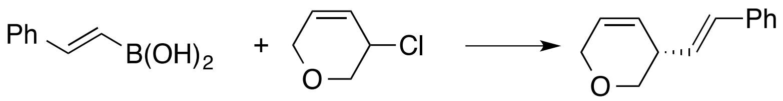

In a 10 mL round bottomed flask $[Rh(cod)(OH)]_2$ (4.6 mg, 0.01 mmol, 0.025 eq), (*R*)-BINAP (14.9 mg, 0.024 mmol, 0.06 eq) and Cs_2CO_3 (130.3 mg, 0.40 mmol, 1.00 eq) were stirred in THF (2.0 mL) at 60 °C for 30 min. A solution of (*E*)-styrylboronic acid (118.4 mg, 0.80 mmol, 2.00 eq) and the allyl chloride (46 µL, 0.40 mmol, 1.00 eq) in THF (1.0 mL) was then added *via* syringe and the flask rinsed with THF (1.0 mL). The resulting mixture was then stirred for 4 h at 60 °C. SiO_2 (20 mg) was added and the solvent was then carefully evaporated. The resulting solid was directly loaded onto a chromatographic column and eluted with pentane to obtain (+)-(*S*,*E*)-3-styryl-3,6-dihydro-2*H*-pyran in 94% yield (85.7 mg, 0.38 mmol).

Enantiomeric excess of 98% was determined by HPLC [Chiralpak® IB; flow: 1.0 mL/min; hexane:iPA 99:1; λ = 210 nm; major enantiomer t_R = 6.5 min; minor enantiomer t_R = 7.9 min].

^{1}H NMR (400 MHz, $CDCl_3$) δ 7.32 – 7.26 (m, 2H), 7.26 – 7.19 (m, 2H), 7.18 – 7.09 (m, 1H), 6.39 (d, *J* = 16.1 Hz, 1H), 6.08 (dd, *J* = 15.9, 8.1 Hz, 1H), 5.76 (s, 2H), 4.09 (d, *J* = 2.7 Hz, 2H), 3.88 (dd, *J* = 11.1, 4.9 Hz, 1H), 3.51 (dd, *J* = 11.1, 6.4 Hz, 1H), 3.07 – 2.95 (m, 1H).

^{13}C NMR (101 MHz, $CDCl_3$) δ 137.3, 131.3, 129.8, 128.7 (2C), 127.4, 127.4, 126.8, 126.3 (2C), 69.2, 65.5, 38.9.

IR (ν_{max}/cm^{-1}): 1262, 1449, 2922, 2993.

HRMS (EI) *m/z* calc. for $C_{13}H_{14}O$ $[M]^+$: 186.1045, found: 186.1038.

$[\alpha]^{25}_{589}$ = +51.2° (c 0.53, $CHCl_3$) for 87% ee.

(+)-(*S*,*E*)-3-(5-Phenylpent-1-en-1-yl)-3,6-dihydro-2*H*-pyran (20)

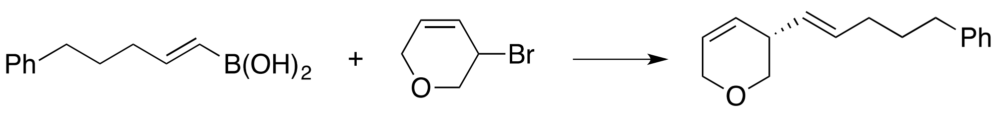

In a 10 mL round bottomed flask $[Rh(cod)(OH)]_2$ (3.4 mg, 0.007 mmol, 0.025 eq), (*R*)-Cl-OMe-BIPHEP **A** (11.7 mg, 0.018 mmol, 0.06 eq) and Cs_2CO_3 (97.7 mg, 0.30 mmol, 1.00 eq) were stirred in THF (1.5 mL) at 60 °C for 30 min. A solution of ((*E*)-(5-phenylpent-1-en-1-yl)boronic acid (114.3 mg, 0.60 mmol, 2.00 eq) and the 3-bromo-3,6-dihydro-2*H*-pyran (48.9 mg, 0.30 mmol, 1.00 eq) in THF (1.0 mL) was then added *via* syringe and the flask rinsed with THF (0.5 mL). The resulting mixture was then stirred for 4 h at 60 °C. SiO_2 (20 mg) was added and the solvent was then carefully evaporated. The resulting solid was directly loaded onto a chromatographic column and eluted with pentane to obtain (+)-(*E*)-3-(5-phenylpent-1-en-1-yl)-3,6-dihydro-2*H*-pyran in 75% yield (51.3 mg, 0.23 mmol).

Enantiomeric excess of 89% was determined by HPLC [Chiralpak® IB; flow: 0.8 mL/min; hexane:iPA 99 :1; λ = 210 nm; major enantiomer t_R = 6.1 min; minor enantiomer t_R = 6.3 min].

^{1}H NMR (400 MHz, $CDCl_3$) δ 7.24 – 7.17 (m, 2H), 7.14 – 7.06 (m, 3H), 5.72 – 5.60 (m, 2H), 5.46 (dt, *J* = 15.6, 6.6 Hz, 1H), 5.27 (dd, *J* = 15.3, 7.8 Hz, 1H), 4.08 – 4.00 (m, 2H), 3.79 (dd, *J* = 11.0, 5.0 Hz, 1H), 3.36 (dd, *J* = 11.0, 6.8 Hz, 1H), 2.86 – 2.76 (m, 1H), 2.54 (d, *J* = 7.5 Hz, 2H), 1.98 (d, *J* = 7.6 Hz, 2H), 1.63 (d, *J* = 7.5 Hz, 2H).

^{13}C NMR (101 MHz, $CDCl_3$) δ 142.6, 132.0, 130.1, 128.6, 128.5, 128.5, 128.4, 128.2, 126.2, 125.8, 69.5, 65.4, 38.5, 35.5, 32.2, 31.2.

IR (ν_{max}/cm^{-1}): 1455, 1496, 2854, 2930, 3027.

HRMS (EI) *m/z* calc. for $C_{16}H_{20}O$ $[M]^+$: 228.1514, found: 228.1501.

$[\alpha]^{25}_{589}$ = +77.2° (c 0.55, $CHCl_3$) for 81% ee.

(+)-(*R*)-2-(Cyclohex-2-en-1-yl)furan (21)

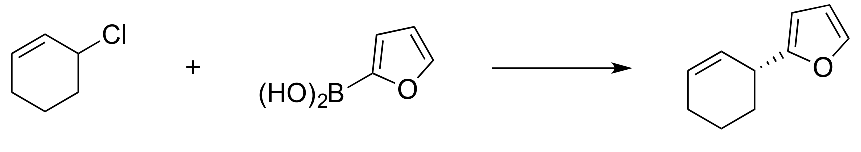

In a 10 mL round bottomed flask $[Rh(cod)(OH)]_2$ (4.6 mg, 0.01 mmol, 0.025 eq), (*R*)-BINAP (14.9 mg, 0.024 mmol, 0.06 eq) and Cs_2CO_3 (130.3 mg, 0.40 mmol, 1.00 eq) were stirred in THF (2.0 mL) at reflux for 30 min. A solution of 3-chlorocyclohexene (45 µL, 0.4 mmol, 1.00 eq) and 2-furanylboronic acid (134.3 mg, 1.20 mmol, 3.00 eq) in THF (1.5 mL) was then added *via* syringe and the flask rinsed with additional THF (0.5 mL). The reaction mixture was refluxed for 2 h. SiO_2 (20 mg) was added and the solvent was then carefully evaporated.

The resulting solid was directly loaded onto a chromatographic column and eluted with pentane to obtain (+)-(*R*)-2-(cyclohex-2-en-1-yl)furan in 51% yield (30.4 mg, 0.20 mmol).

GC analysis indicated an enantiomeric excess of 90% [Hydrodex® *β*-3 P; flow: 3.6 mL/min; 10 min at 60 °C, then 1 °C/min to 85 °C, hold it for 15 min; major enantiomer t_R = 41.3 min; minor enantiomer t_R = 42.1 min].

^{1}H-NMR (400 MHz, $CDCl_3$) δ 1.58 – 1.81 (m, 3 H), 1.92 – 2.13 (m, 3 H), 3.37 – 3.57 (m, 1 H), 5.72 – 5.81 (m, 1 H), 5.81 – 5.91 (m, 1 H), 5.99 (d, *J* = 3.1 Hz, 1 H), 6.28 (dd, *J* = 3.2, 1.9 Hz, 1 H), 7.32 (d, *J* = 1.8 Hz, 1 H).

^{13}C-NMR (100 MHz, $CDCl_3$) δ 20.6, 25.1, 28.4, 35.2, 104.6, 110.1, 127.3, 128.9, 141.1, 159.1.

IR (ATR) ν_{max} /cm^{-1} = 2937l, 2868s, 1646m, 1347m, 1156s, 1072m, 1008l, 884s, 742l.

HRMS (EI/FI): *m/z* calc. for $C_{10}H_{12}O$ $[M]^+$: 148.0888, found: 148.0890.

$[\alpha]^{25}_{589}$ = +167.9° (*c* 1.00, $CHCl_3$).

(+)-(*R*)-2-(Cyclohex-2-en-1-yl)thiophene (22)

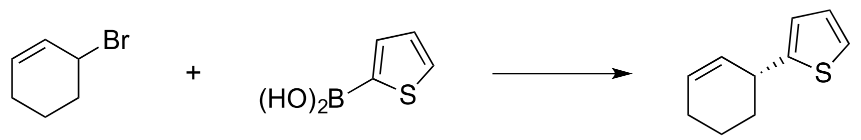

In a 10 mL round bottomed flask $[Rh(cod)(OH)]_2$ (4.6 mg, 0.01 mmol, 0.025 eq), (*R*)-BINAP (14.9 mg, 0.024 mmol, 0.06 eq) and Cs_2CO_3 (130.3 mg, 0.40 mmol, 1.00 eq) were stirred in THF (2.0 mL) at reflux for 30 min. A solution of 3-bromocyclohexene (46 µL, 0.4 mmol, 1.00 eq) and 2-thienylboronic acid (153.6 mg, 1.20 mmol, 3.00 eq) in THF (1.5 mL) was then added *via* syringe and the flask rinsed with additional THF (0.5 mL). The reaction mixture was refluxed for 1.5 h. SiO_2 (20 mg) was added and the solvent was then carefully evaporated. The resulting solid was directly loaded onto a chromatographic column and eluted with pentane to obtain (+)-(*R*)-2-(cyclohex-2-en-1-yl)thiophene in 51% yield (33 mg, 0.20 mmol).

HPLC analysis indicated an enantiomeric excess of 99% [Chiralpak® ID; flow: 1.0 mL/min; hexane/*i*-PrOH 99.9:0.1; *λ* = 210 nm; minor enantiomer t_R = 5.6 min; major enantiomer t_R = 5.9 min].

^{1}H-NMR (400 MHz, $CDCl_3$) δ 1.57 – 1.69 (m, 1 H), 1.69 – 1.83 (m, 2 H), 1.98 – 2.16 (m, 3 H), 3.64 – 3.75 (m, 1 H), 5.74 – 5.82 (m, 1 H), 5.83 – 5.93 (m, 1 H), 6.83 (d, *J* = 3.4 Hz, 1 H), 6.94 (dd, *J* = 5.1, 3.4 Hz, 1 H), 7.14 (d, *J* = 5.1 Hz, 1 H).

^{13}C-NMR (100 MHz, $CDCl_3$) δ 20.7, 24.9, 32.5, 36.7, 122.9, 123.3, 126.5, 128.3, 129.7, 150.5.

IR (ATR) ν_{max} /cm^{-1} = 3021s, 2931m, 2858s, 2835s, 1455s, 850s, 821s, 758s, 723s, 692l, 652m.

HRMS (EI/FI): *m/z* calc. for $C_{10}H_{12}S$ $[M]^+$: 164.0660, found: 164.0661.

$[\alpha]^{25}_{589}$ = +77.4° (*c* 0.50, $CHCl_3$).

(*S*)-*N*-(*tert*-Butoxycarbonyl)-2-(cyclohex-2-en-1-yl)-1*H*-pyrrole (23)

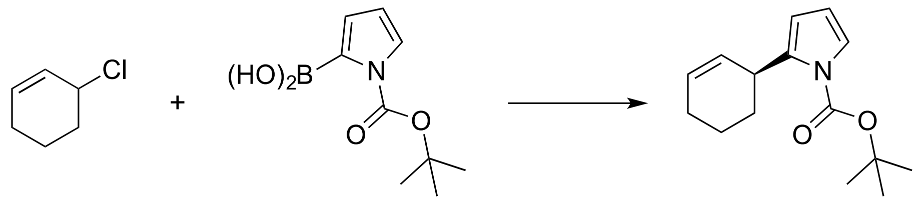

In a 10 mL round bottomed flask $[Rh(cod)(OH)]_2$ (4.6 mg, 0.01 mmol, 0.025 eq), (*S*)-BINAP (14.9 mg, 0.024 mmol, 0.06 eq) and Cs_2CO_3 (130.3 mg, 0.40 mmol, 1.00 eq) were stirred in THF (2.0 mL) at reflux for 30 min. A solution of 3-chlorocyclohexene (45 µL, 0.4 mmol, 1.00 eq) and *N*-(*tert*-butoxycarbonyl)-pyrrole-2-boronic acid (253.2 mg, 1.20 mmol, 3.00 eq) in THF (1.5 mL) was then added *via* syringe and the flask rinsed with additional THF (0.5 mL). The reaction mixture was refluxed for 12 h. SiO_2 (20 mg) was added and the solvent was then carefully evaporated. The resulting solid was directly loaded onto a chromatographic column and eluted with petrol ether and ethyl acetate (97:3). The product could not be separated in this step from impurities.

^{1}H-NMR (400 MHz, $CDCl_3$) δ 1.55 –1.67 (m, 4 H), 1.59 (s, 9 H), 1.93 – 2.08 (m, 3 H), 4.06 (ddd, *J* = 5.6, 2.8, 2.8 Hz, 1 H), 5.68 – 5.76 (m, 1 H), 5.79 – 5.85 (m, 1 H), 5.98 (ddd, *J* = 3.3, 1.9, 0.9 Hz, 1 H), 6.08 (dd, *J* = 3.3, 3.3 Hz, 1 H), 7.23 (dd, *J* = 3.4, 1.9 Hz, 1 H).

^{13}C-NMR (100 MHz, $CDCl_3$) δ 20.1, 25.3, 28.2 (3C), 29.6, 34.1, 83.5, 109.9, 111.9, 121.4, 128.1, 129.3, 139.5, 149.6.

IR (ATR) ν_{max} /cm^{-1} = 2930s, 1740s, 1401s, 1369s, 1324l, 1163m, 1119m, 1062s, 722s.

HRMS (ESI): *m*/*z* calc. for $C_{15}H_{22}NO_2$ $[M+H]^+$: 248.1645, found: 248.1646.

(–)-(*S*)-2-(Cyclohex-2-en-1-yl)-1*H*-pyrrole (23a)

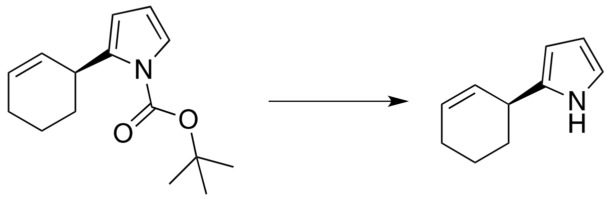

A solution of (*S*)-*N*-(*tert*-Butoxycarbonyl)-2-(cyclohex-2-en-1-yl)-1*H*-pyrrole (42.0 mg, 0.17 mmol) and K_2CO_3 (117 mg, 0.85 mmol) in methanol (1.7 mL) was stirred for 3 h at 60 °C. The reaction was diluted with EtOAc. Al_2O_3 (20 mg) was added and the solvent was then carefully evaporated. The resulting solid was directly loaded onto a flash chromatography column and eluted with petrol ether and ethyl acetate (97:3) to obtain (–)-(*S*)-2-(cyclohex-2-en-1-yl)-1*H*-pyrrole in 58% yield over 2 steps (17 mg, 0.12 mmol).

HPLC analysis indicated an enantiomeric excess of 95% [Chiralpak® IB; flow: 1.0 mL/min; hexane/*i*-PrOH 99:1; λ = 210 nm; major enantiomer t_R = 7.4 min; minor enantiomer t_R = 7.8 min].

^{1}H-NMR (400 MHz, $CDCl_3$) δ 1.56 – 1.77 (m, 3 H), 1.94 – 2.04 (m, 1 H), 2.04 – 2.15 (m, 2 H), 3.50 (m, 1 H), 5.73 – 5.83 (m, 1 H), 5.89 (dddd, *J* = 9.6, 3.2, 3.2, 2.9 Hz, 1 H), 5.93 – 6.00 (m, 1 H), 6.12 – 6.23 (m, 1 H), 6.63 – 6.78 (m, 1 H), 8.00 (br s, 1 H, NH).

^{13}C-NMR (100 MHz, $CDCl_3$) δ 20.7, 25.0, 30.9, 34.5, 104.5, 108.3, 116.1, 128.4, 128.9, 136.1.

IR (ATR) ν_{max} /cm^{-1} = 3384m, 2930m, 2858s, 2836s, 1431s, 1094s, 1026s, 791s, 765s, 714l.

HRMS (ESI): *m/z* calc. for $C_{10}H_{14}N$ $[M+H]^+$: 148.1121, found: 148.1121.

$[\alpha]^{25}_{589}$ = –128.9° (*c* 0.70, $CHCl_3$).

(+)-(*R*)-3-(Cyclohex-2-en-1-yl)furan (24)

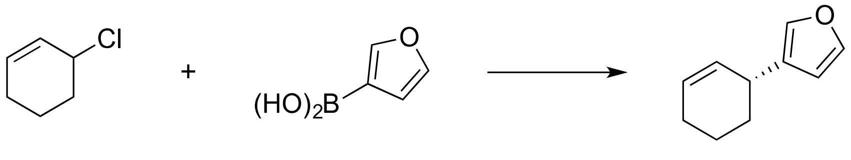

In a 10 mL round bottomed flask $[Rh(cod)(OH)]_2$ (4.6 mg, 0.01 mmol, 0.025 eq), (*R*)-BINAP (14.9 mg, 0.024 mmol, 0.06 eq) and Cs_2CO_3 (130.3 mg, 0.40 mmol, 1.00 eq) were stirred in THF (2.0 mL) at reflux for 30 min. A solution of 3-chlorocyclohexene (45 µL, 0.4 mmol, 1.00 eq) and 3-furanylboronic acid (134.3 mg, 1.20 mmol, 3.00 eq) in THF (1.5 mL) was then added *via* syringe and the flask rinsed with additional THF (0.5 mL). The reaction mixture was refluxed for 2 h. SiO_2 (20 mg) was added and the solvent was then carefully evaporated. The resulting solid was directly loaded onto a chromatographic column and eluted with pentane to obtain (+)-(*R*)-3-(cyclohex-2-en-1-yl)furan in 51% yield (30.4 mg, 0.20 mmol).

^{1}H-NMR (400 MHz, $CDCl_3$) δ 1.53 – 1.66 (m, 2 H), 1.67 – 1.78 (m, 1 H), 1.91 – 2.00 (m, 1 H), 2.01 – 2.08 (m, 2 H), 3.18 – 3.38 (m, 1 H), 5.64 – 5.75 (m, 1 H), 5.75 – 5.83 (m, 1 H), 6.30 (d, *J* = 1.8 Hz, 1 H), 7.20 (s, 1 H), 7.36 (d, *J* = 1.7 Hz, 1 H).

^{13}C-NMR (100 MHz, $CDCl_3$) δ 20.8, 25.2, 30.6, 32.3, 110.2, 128.0, 129.7, 129.9, 138.9, 142.9.

IR (ATR) ν_{max} /cm^{-1} = 2930m, 1158m, 1068m, 1024m, 872l, 785l, 760l, 724m, 660m.

HRMS (EI/FI): *m/z* calc. for $C_{10}H_{12}O$ $[M]^+$: 148.0888, found: 148.0889.

$[\alpha]^{25}_{589}$ = +136.4° (*c* 1.00, $CHCl_3$).

For ee-determination

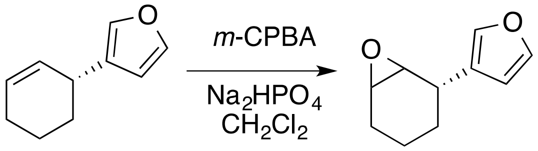

24 (1.00 eq) was dissolved in CH_2Cl_2 (2 mL, for 0.15 mmol scale reaction) under an argon atmosphere. *m*-CPBA (2.00 eq) and Na_2HPO_4 (3.00 eq) were added at room temperature and the reaction mixture was stirred for 2 h. The reaction mixture was diluted with Et_2O (4 mL) and quenched with an aqueous solution of saturated $Na_2S_2O_3$ (4 mL). The organic layer was washed with NaOH (1 M aq., 3 × 5 mL), dried over Mg_2SO_4, filtered and concentrated *in vacuo*. The crude mixture of diastereoisomeric epoxides was directly analyzed by GC chromatography using a chiral non-racemic stationary phase.

GC analysis indicated an enantiomeric excess of 98% [Hydrodex *β*-3 P; flow: 3.6 mL/min; 10 min at 60 °C, then 2 °C/min to 150 °C; major diasteriomer, t_R = 47.5 min/48.3 min; minor diasteriomer, t_R = 47.3 min/48.8 min].

(+)-(*R*)-3-(Cyclohex-2-en-1-yl)thiophene (25)

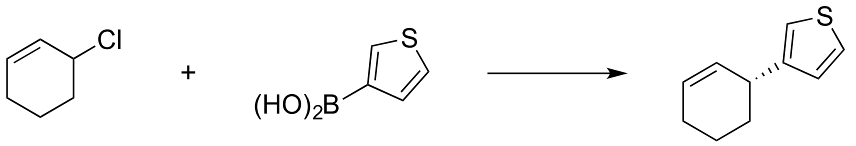

In a 10 mL round bottomed flask $[Rh(cod)(OH)]_2$ (4.6 mg, 0.01 mmol, 0.025 eq), (*R*)-BINAP (14.9 mg, 0.024 mmol, 0.06 eq) and Cs_2CO_3 (130.3 mg, 0.40 mmol, 1.00 eq) were stirred in THF (2.0 mL) at reflux for 30 min. A solution of 3-chlorocyclohexene (45 µL, 0.4 mmol, 1.00 eq) and 3-thienylboronic acid (153.6 mg, 1.20 mmol, 3.00 eq) in THF (1.5 mL) was then added *via* syringe and the flask rinsed with additional THF (0.5 mL). The reaction mixture was refluxed for 1.5 h. SiO_2 (20 mg) was added and the solvent was then carefully evaporated. The resulting solid was directly loaded onto a chromatographic column and eluted with pentane to obtain (+)-(*R*)-3-(cyclohex-2-en-1-yl)thiophene in 56% yield (37.0 mg, 0.22 mmol.

HPLC analysis indicated an enantiomeric excess of 97% [Chiralpak® ID; flow: 1.0 mL/min; hexane/*i*-PrOH 99.9:0.1; *λ* = 210 nm; major enantiomer t_R = 5.4 min; minor enantiomer t_R = 5.8 min].

^{1}H-NMR (400 MHz, $CDCl_3$) δ 1.59 – 1.70 (m, 2 H), 1.71 – 1.81 (m, 1 H), 1.99 – 2.06 (m, 1 H), 2.07 – 2.16 (m, 2 H), 3.46 – 3.61 (m, 1 H), 5.76 – 5.84 (m, 1 H), 5.84 – 5.93 (m, 1 H), 7.00 (d, *J* = 2.9 Hz, 1 H), 7.02 (d, *J* = 5.0 Hz, 1 H), 7.29 (dd, *J* = 5.0, 2.9 Hz, 1 H).

^{13}C-NMR (100 MHz, $CDCl_3$) δ 21.0, 25.2, 31.3, 37.1, 120.0, 125.3, 127.6, 128.0, 130.1, 147.3.

IR (ATR) ν_{max} /cm^{-1} = 2931m, 2858s, 2835s, 854s, 839s, 777l, 745s, 736s, 651m, 637s.

HRMS (EI/FI): *m/z* calc. for $C_{10}H_{12}S$ $[M]^+$: 164.0660, found: 164.0653.

$[\alpha]^{25}{}_{589}$ = +46.5° (*c* 1.00, $CHCl_3$).

(+)-(*R*)-2-(Cyclohex-2-en-1-yl)benzofuran (27)

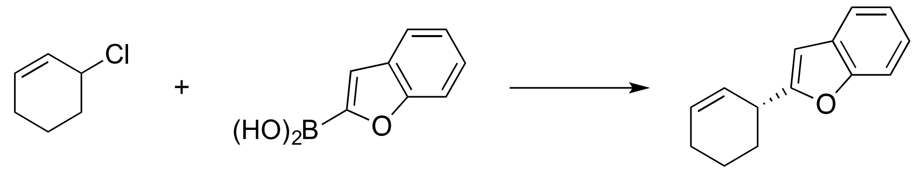

In a 10 mL round bottomed flask $[Rh(cod)(OH)]_2$ (4.6 mg, 0.01 mmol, 0.025 eq), (*R*)-Xyl-P-Phos (18.2 mg, 0.024 mmol, 0.06 eq) and Cs_2CO_3 (130.3 mg, 0.40 mmol, 1.00 eq) were stirred in THF (2.0 mL) at reflux for 30 min. A solution of 3-chlorocyclohexene (45 µL, 0.4 mmol, 1.00 eq) and 2-benzofuranylboronic acid (129.6 mg, 0.8 mmol, 2.00 eq) in THF (1.5 mL) was then added *via* syringe and the flask rinsed with additional THF (0.5 mL). The reaction mixture was refluxed for 1.5 h. SiO_2 (20 mg) was added and the solvent was then carefully evaporated. The resulting solid was directly loaded onto a chromatographic column and eluted with pentane to obtain (+)-(*R*)-2-(cyclohex-2-en-1-yl)benzofuran in 75% yield (57.0 mg, 0.30 mmol).

HPLC analysis indicated an enantiomeric excess of 95% [Chiralpak® IB; flow: 1.0 mL/min; hexane/*i*-PrOH 99.9:0.1; λ = 210 nm; major enantiomer t_R = 5.9 min; minor enantiomer t_R = 6.4 min].

^{1}H-NMR (400 MHz, $CDCl_3$) δ 1.59 – 1.71 (m, 1 H), 1.72 – 1.83 (m, 1 H), 1.90 (dddd, *J* = 12.8, 9.6, 7.0, 2.9 Hz, 1 H), 2.02 – 2.15 (m, 3 H), 3.55 – 3.68 (m, 1 H), 5.81 – 5.90 (m, 1 H), 5.90 – 5.99 (m, 1 H), 6.40 (s, 1 H), 7.20 (m, 2 H), 7.43 (d, *J* = 7.8 Hz, 1 H), 7.47 – 7.53 (m, 1H).

^{13}C-NMR (100 MHz, $CDCl_3$) δ 20.3, 25.0, 27.9, 35.4, 101.7, 110.8, 120.3, 122.4, 123.2, 126.4, 128.8, 129.5, 154.7, 162.1.

IR (ATR) ν_{max} /cm^{-1} = 2933s, 1584s, 1454l, 1254l, 1164m, 949s, 873s, 795m, 748l, 725s.

HRMS (EI): *m/z* calc. for $C_{14}H_{14}O$ $[M]^+$: 198.1045, found: 198.1047.

$[\alpha]^{25}{}_{589}$ = +167.5° (*c* 1.00, $CHCl_3$).

(+)-(*R*)-2-(Cyclohex-2-en-1-yl)benzo[*b*]thiophene (28)

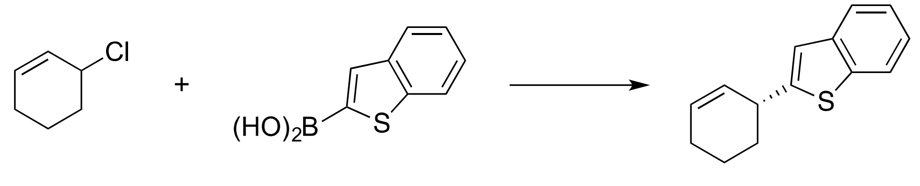

In a 10 mL round bottomed flask $[Rh(cod)(OH)]_2$ (4.6 mg, 0.01 mmol, 0.025 eq), (*R*)-Xyl-P-Phos (18.2 mg, 0.024 mmol, 0.06 eq) and Cs_2CO_3 (130.3 mg, 0.40 mmol, 1.00 eq) were stirred in THF (2.0 mL) at reflux for 30 min. A solution of 3-chlorocyclohexene (45 µL, 0.4 mmol, 1.00 eq) and benzo[*b*]thien-2-ylboronic acid (142.4 mg, 0.8 mmol, 2.00 eq) in THF (1.5 mL) was then added *via* syringe and the flask rinsed with additional THF (0.5 mL). The reaction mixture was refluxed for 2 h. SiO_2 (20 mg) was added and the solvent was then carefully evaporated. The resulting solid was directly loaded onto a chromatographic column and eluted with pentane to obtain (+)-(*R*)-2-(cyclohex-2-en-1-yl)benzo[*b*]thiophene in 23% yield (18.8 mg, 0.09 mmol).

HPLC analysis indicated an enantiomeric excess of 99% [Chiralpak® IB; flow: 1.0 mL/min; hexane/*i*-PrOH 99.9:0.1; λ = 210 nm; major enantiomer t_R = 8.5 min; minor enantiomer t_R = 9.5 min].

^{1}H-NMR (500 MHz, $CDCl_3$) δ 1.58 – 1.73 (m, 1 H), 1.74 – 1.88 (m, 2 H), 1.99 – 2.17 (m, 3 H), 3.69 – 3.81 (m, 1 H), 5.83 – 5.89 (m, 1 H), 5.91 (ddd, *J* = 10.0, 5.8, 3.5 Hz, 1 H), 7.05 (s, 1 H), 7.25 (dd, *J* = 8.2, 7.1 Hz, 1 H), 7.30 (dd, *J* = 8.2, 7.2 Hz, 1 H), 7.67 (d, *J* = 7.6 Hz, 1 H), 7.77 (d, *J* = 8.2 Hz, 1 H).

^{13}C-NMR (126 MHz, $CDCl_3$) δ 20.7, 25.1, 32.1, 37.5, 120.2, 122.4, 123.0, 123.6, 124.2, 129.0, 129.1, 139.4, 140.2, 151.4.

IR (ATR) ν_{max} /cm^{-1} = 2931m, 2858s, 2835l, 1457s, 1435m, 1068s, 856s, 825s, 745l, 726l.

HRMS (EI): *m/z* calc. for $C_{14}H_{14}S$ $[M+H]^+$: 214.0816, found: 214.0819.

$[\alpha]^{25}_{589}$ = –159.0° (*c* 1.00, $CHCl_3$);

(–)-(*S*)-*N*-(*tert*-Butoxycarbonyl)-2-(cyclohex-2-en-1-yl)-1*H*-indole (29)

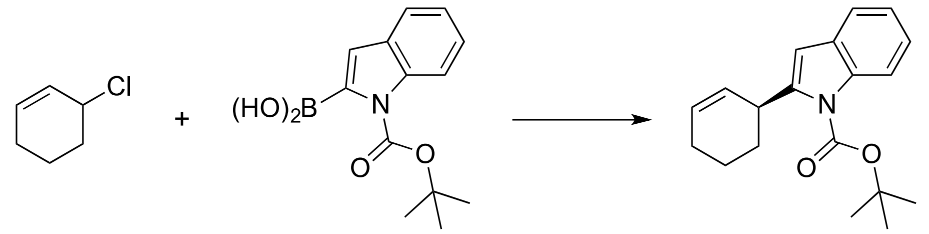

In a 10 mL round bottomed flask $[Rh(cod)(OH)]_2$ (4.6 mg, 0.01 mmol, 0.025 eq), (*S*)-BINAP (14.9 mg, 0.024 mmol, 0.06 eq) and Cs_2CO_3 (130.3 mg, 0.40 mmol, 1.00 eq) were stirred in THF (2.0 mL) at reflux for 30 min. A solution of 3-chlorocyclohexene (45 µL, 0.4 mmol, 1.00 eq) and *N*-(*tert*-butoxycarbonyl)-indole-2-boronic acid (313.3 mg, 1.20 mmol, 3.00 eq) in THF (1.5 mL) was then added *via* syringe and the flask rinsed with additional THF (0.5 mL). The reaction mixture was refluxed for 20 h. SiO_2 (20 mg) was added and the solvent was then carefully evaporated. The resulting solid was directly loaded onto a chromatographic column and eluted with petrol ether and ethyl acetate (97:3) to obtain (–)-(*S*)-*N*-(*tert*-butoxycarbonyl)-2-(cyclohex-2-en-1-yl)-1*H*-indole in 20% yield (24.0 mg, 0.08 mmol).

HPLC analysis indicated an enantiomeric excess of 95% [Chiralpak® IA; flow: 1.0 mL/min; hexane/*i*-PrOH 99:1; λ = 210 nm; minor enantiomer t_R = 4.45 min; minor enantiomer t_R = 5.26 min].

^{1}H-NMR (400 MHz, $CDCl_3$) δ 1.56 – 1.78 (m, 3 H), 1.69, (s, 9 H), 2.01 – 2.14 (m, 3 H), 4.17 – 4.29 (m, 1 H), 5.78 – 5.87 (m, 1 H), 5.87 – 5.94 (m, 1 H), 6.39 (s, 1 H), 7.18 (ddd, *J* = 7.4, 7.3, 1.3 Hz, 1 H), 7.21 – 7.26 (m, 1 H), 7.45 (d, *J* = 7.3 Hz, 1 H), 8.12 (d, *J* = 8.3 Hz, 1 H).

^{13}C-NMR (100 MHz, $CDCl_3$) δ 19.9, 25.4, 28.4 (3C), 29.6, 34.8, 83.9, 108.3, 115.9, 120.0, 122.7, 123.5, 128.6, 128.9, 129.3, 137.1, 145.4, 150.7.

IR (ATR) ν_{max} /cm^{-1} = 2930s, 1731l, 1454s, 1370m, 1329l, 1162m, 1116s, 1086s, 745s.

HRMS (ESI): *m/z* calc. for $C_{19}H_{23}NO_2Na$ $[M+Na]^+$: 320.1621, found: 320.1621.

$[\alpha]^{25}_{589}$ = –33.6° (*c* 0.50, $CHCl_3$).

(–)-(*R*)-4-(Cyclohex-2-en-1-yl)dibenzo[*b,d*]thiophene (30)

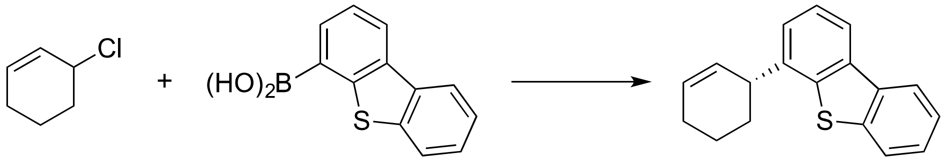

In a 10 mL round bottomed flask $[Rh(cod)(OH)]_2$ (4.6 mg, 0.01 mmol, 0.025 eq), (*R*)-BINAP (14.9 mg, 0.024 mmol, 0.06 eq) and Cs_2CO_3 (130.3 mg, 0.40 mmol, 1.00 eq) were stirred in THF (2.0 mL) at reflux for 30 min. 4-Dibenzothienylboronic acid (274 mg, 1.20 mmol, 3.00 eq) and a solution of 3-chlorocyclohexene (45 µL, 0.4 mmol, 1.00 eq) in THF (1.5 mL) was then added *via* syringe and the flask rinsed with additional THF (0.5 mL). The reaction mixture was refluxed for 2.5 h. SiO_2 (20 mg) was added and the solvent was then carefully evaporated. The resulting solid was directly loaded onto a chromatographic column and eluted with pentane to obtain (–)-(*R*)-4-(cyclohex-2-en-1-yl)dibenzo[*b,d*]thiophene in 57% yield (58.5 mg, 0.23 mmol).

HPLC analysis indicated an enantiomeric excess of 90% [Chiralpak® IC; flow: 1.0 mL/min; hexane/*i*-PrOH 99.7:0.3; λ = 230 nm; major enantiomer t_R = 12.3 min; minor enantiomer t_R = 13.3 min].

^{1}H-NMR (500 MHz, $CDCl_3$) δ 1.65 – 1.89 (m, 3H), 2.11 – 2.26 (m, 3H), 3.73 – 3.81 (m, 1H), 5.79 – 5.88 (m, 1H), 6.04 (dtd, *J* = 9.8, 3.7, 3.6, 2.3 Hz, 1H), 7.34 (dd, *J* = 7.4, 1.1 Hz, 1H), 7.41 – 7.50 (m, 3H), 7.83 – 7.90 (m, 1H), 8.04 (dd, *J* = 7.8, 1.2 Hz, 1H), 8.12 – 8.20 (m, 1H).

^{13}C-NMR (126 MHz, $CDCl_3$) δ 21.3, 25.2, 29.5, 41.4, 119.6, 121.8, 122.9, 124.4, 124.9, 125.6, 126.7, 129.0, 129.6, 136.0, 136.3, 138.7, 139.3, 140.7.

IR (ATR) ν_{max} /cm^{-1} = 3059s, 3021s, 2928m, 2859s, 1443m, 1399s, 1048s, 797s, 749l, 718s.

HRMS (ESI): *m/z* calc. for $C_{18}H_{17}S$ $[M+H]^+$: 265.1040, found: 265.1045.

$[\alpha]^{25}_{589}$ = –1.0° (*c* 1.00, $CHCl_3$).

(–)-(*R*)-5-(Cyclohex-2-en-1-yl)-1*H*-indole (33)

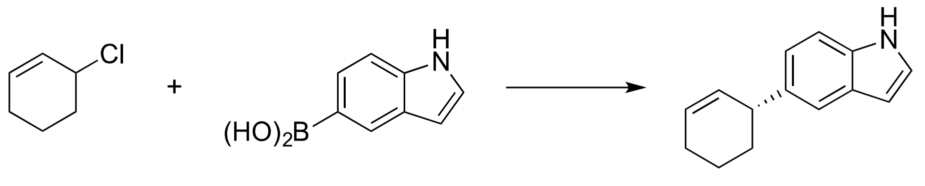

In a 10 mL round bottomed flask $[Rh(cod)(OH)]_2$ (4.6 mg, 0.01 mmol, 0.025 eq), (*R*)-BINAP (14.9 mg, 0.024 mmol, 0.06 eq) and Cs_2CO_3 (130.3 mg, 0.40 mmol, 1.00 eq) were stirred in THF (2.0 mL) at reflux for 30 min. A solution of 3-chlorocyclohexene (45 µL, 0.4 mmol, 1.00 eq) and 5-indolylboronic acid (193.2 mg, 1.20 mmol, 3.00 eq) in THF (1.5 mL) was then added *via* syringe and the flask rinsed with additional THF (0.5 mL). The reaction mixture was refluxed for 1.5 h. SiO_2 (20 mg) was added and the solvent was then carefully evaporated. The resulting solid was directly loaded onto a chromatographic column and eluted with petrol ether and ethyl acetate (9:1, 1% Et_3N) to obtain (–)-(*R*)-5-(cyclohex-2-en-1-yl)-1*H*-indole in 41% yield (33 mg, 0.16 mmol).

HPLC analysis indicated an enantiomeric excess of 96% [Chiralpak® IC; flow: 1.0 mL/min; hexane/*i*-PrOH 97:3; λ = 210 nm; major enantiomer t_R = 9.1 min; minor enantiomer t_R = 9.8 min]

^{1}H-NMR (400 MHz, $CDCl_3$) δ 1.59 – 1.73 (m, 2 H), 1.74 – 1.86 (m, 1 H), 2.02 – 2.19 (m, 3 H), 3.53 (m, 1 H), 5.76 – 5.87 (m, 1 H), 5.87 – 5.96 (m, 1 H), 6.53 (dd, *J* = 2.5, 2.5 Hz, 1 H), 7.10 (dd, *J* = 8.4, 1.6 Hz, 1 H), 7.18 (d, *J* = 2.8 Hz, 1 H), 7.33 (d, *J* = 8.3 Hz, 1 H), 7.51 (d, *J* = 1.6 Hz, 1 H), 8.03 (br s, 1 H, NH).
^{13}C-NMR (100 MHz, $CDCl_3$) δ 21.5, 25.3, 33.4, 42.1, 102.6, 110.9, 119.4, 122.6, 124.4, 127.9, 128.1, 131.4, 134.6, 138.3.

IR (ATR) ν_{max} /cm^{-1} = 3410m, 2926m, 1474s, 1453s, 1415s, 1336s, 895s, 806s, 764m, 722l.

HRMS (EI/FI): *m/z* calc. for $C_{14}H_{15}N$ $[M]^+$: 197.1204, found: 197.1212.

$[\alpha]^{25}_{589}$ = –161.4° (*c* 1.00, $CHCl_3$).

(–)-(*S*)-6-(Cyclohex-2-en-1-yl)-1*H*-indole (34)

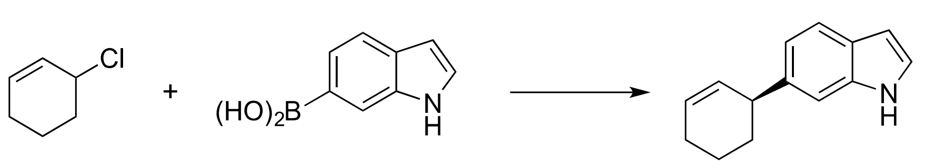

In a 10 mL round bottomed flask $[Rh(cod)(OH)]_2$ (4.6 mg, 0.01 mmol, 0.025 eq), (*S*)-BINAP (14.9 mg, 0.024 mmol, 0.06 eq) and Cs_2CO_3 (130.3 mg, 0.40 mmol, 1.00 eq) were stirred in THF (2.0 mL) at reflux for 30 min. A solution of 3-chlorocyclohexene (45 µL, 0.4 mmol, 1.00 eq) and 6-indolylboronic acid (193.2 mg, 1.20 mmol, 3.00 eq) in THF (1.5 mL) was then added to the flask *via* syringe and rinsed with additional THF (0.5 mL). The reaction mixture was refluxed for 1.5 h. SiO_2 (20 mg) was added and the solvent was then carefully evaporated. The resulting solid was directly loaded onto a chromatographic column and eluted with petrol ether and ethyl acetate (9:1) to obtain (–)-(*S*)-6-(cyclohex-2-en-1-yl)-1*H*-indole in 72% yield (57.0 mg, 0.29 mmol).

HPLC analysis indicated an enantiomeric excess of 96% [Chiralpak® IB; flow: 1.0 mL/min; hexane/*i*-PrOH 98:2; $\lambda = 210$ nm; minor enantiomer $t_R = 14.2$ min; major enantiomer $t_R = 15.9$ min].

^{1}H-NMR (400 MHz, $CDCl_3$) δ 1.62 – 1.72 (m, 2 H), 1.75 – 1.83 (m, 1 H), 2.05 – 2.16 (m, 3 H), 3.54 (dddd, *J* = 8.3, 5.6, 2.8, 2.8 Hz, 1 H), 5.77 – 5.87 (m, 1 H), 5.93 (dddd, *J* = 9.8, 3.6, 3.5, 2.3 Hz, 1 H), 6.53 (ddd, *J* = 3.1, 2.1, 1.0 Hz, 1 H), 7.04 (dd, *J* = 8.1, 1.5 Hz, 1 H), 7.16 (dd, *J* = 3.2, 2.4 Hz, 1 H), 7.23 – 7.28 (m, 1 H), 7.59 (d, *J* = 8.2 Hz, 1 H), 8.02 (br, 1 H, NH).

^{13}C-NMR (100 MHz, $CDCl_3$) δ 21.4, 25.3, 33.2, 42.2, 102.5, 109.9, 120.5, 120.6, 123.9, 126.3, 128.2, 131.1, 136.2, 141.0.

IR (ATR) ν_{max} /cm^{-1} = 3412l, 3019s, 2926m, 2851s, 1450m, 1341m, 1090s, 813m, 766s, 724m.

HRMS (Ammonia CI): *m/z* calc. for $C_{14}H_{16}N$ $[M+H]^+$: 198.1277, found: 198.1282.

$[\alpha]^{25}_{589}$ = –119.2° (*c* 1.00, $CHCl_3$).

(–)-(*R*)-2-(Cyclohex-2-en-1-yl)-5-methylthiophene (36)

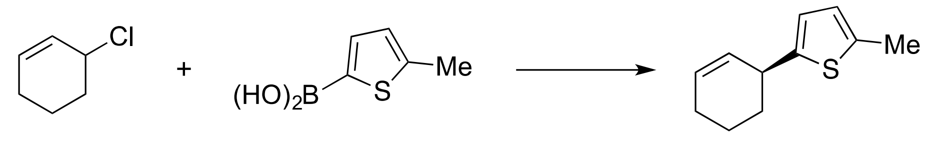

In a 10 mL round bottomed flask $[Rh(cod)(OH)]_2$ (4.6 mg, 0.01 mmol, 0.025 eq), (*S*)-BINAP (14.9 mg, 0.024 mmol, 0.06 eq) and Cs_2CO_3 (130.3 mg, 0.40 mmol, 1.00 eq) were stirred in THF (2.0 mL) at 60 °C for 30 min. A solution of 3-chlorocyclohexene (45 µL, 0.4 mmol, 1.00 eq) and 5-methyl-2-thienylboronic acid (170.4 mg, 1.20 mmol, 3.00 eq) in THF (1.5 mL) was then added at room temperature *via* syringe and the flask rinsed with additional THF (0.5 mL). The reaction mixture was stirred for 12 h at room temperature. SiO_2 (20 mg) was added and the solvent was then carefully evaporated. The resulting solid was directly loaded onto a chromatographic column and eluted with pentane to obtain (–)-(*R*)-2-(cyclohex-2-en-1-yl)-5-methylthiophene in 33% yield (32.5 mg, 0.13 mmol).

HPLC analysis indicated an enantiomeric excess of 95% [Chiralpak® AY-H; flow: 0.4 mL/min; hexane; $\lambda = 210$ nm; minor enantiomer $t_R = 11.7$ min; major enantiomer $t_R = 12.2$ min].

^{1}H-NMR (400 MHz, $CDCl_3$) δ 1.56 – 1.82 (m, 3 H), 2.00 – 2.09 (m, 3 H), 2.44 (s, 3 H), 3.52 – 3.66 (m, 1 H), 5.74 – 5.79 (m, 1 H), 5.80 – 5.85 (m, 1 H), 6.57 (d, *J* = 3.5 Hz, 1 H), 6.59 (d, *J* = 3.5 Hz, 1 H).

^{13}C-NMR (100 MHz, $CDCl_3$) δ 15.5, 20.8, 25.1, 32.5, 37.0, 123.2, 124.7, 128.3, 123.0, 137.4, 148.3.

IR (ATR) ν_{max} /cm^{-1} = 3021s, 2931m, 2859s, 1445s, 1232s, 1045s, 876s, 796l, 760s, 723s.

HRMS (EI/FI): *m/z* calc. for $C_{11}H_{14}S$ $[M]^+$: 178.0816, found: 178.0821.

$[\alpha]^{25}_{589}$ = –68.4° (*c* 0.50, $CHCl_3$).

(–)-(*S*)-5-(Cyclohex-2-en-1-yl)thiophene-2-carbaldehyde (37)

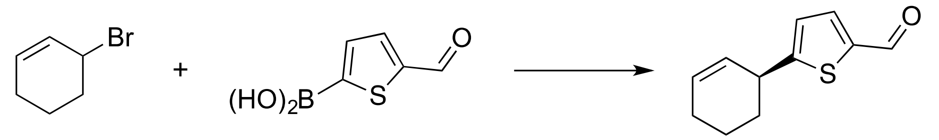

In a 10 mL round bottomed flask $[Rh(cod)(OH)]_2$ (4.6 mg, 0.01 mmol, 0.025 eq), (*S*)-BINAP (14.9 mg, 0.024 mmol, 0.06 eq) and Cs_2CO_3 (130.3 mg, 0.40 mmol, 1.00 eq) were stirred in THF (2.0 mL) at reflux for 30 min. A solution of 3-bromocyclohexene (46 µL, 0.4 mmol, 1.00 eq) and 5-formyl-2-thienylboronic acid (187.2 mg, 1.20 mmol, 3.00 eq) in THF (1.5 mL) was then added *via* syringe and the flask rinsed with additional THF (0.5 mL). The reaction mixture was refluxed for 2 h. SiO_2 (20 mg) was added and the solvent was then carefully evaporated. The resulting solid was directly loaded onto a chromatographic column and eluted with petrol ether and ethyl acetate (95:5) to obtain (–)-(*S*)-5-(cyclohex-2-en-1-yl)thiophene-2-carbaldehyde in 30% yield (23.1 mg, 0.12 mmol).

HPLC analysis indicated an enantiomeric excess of greater than 99% [Chiralpak® IB; flow: 1.4 mL/min; hexane/*i*-PrOH 99.5:0.5; λ = 210 nm; major enantiomer t_R = 19.1 min; minor enantiomer t_R = 22.2 min].

^{1}H-NMR (400 MHz, $CDCl_3$) δ 1.58 – 1.80 (m, 3 H), 2.02 – 2.15 (m, 3 H), 3.67 – 3.76 (m, 1 H), 5.69 – 5.82 (m, 1 H), 5.85 – 5.97 (m, 1 H), 6.95 (d, *J* = 3.8 Hz, 1 H), 7.62 (d, *J* = 3.8 Hz, 1 H), 9.82 (s, 1 H).

^{13}C-NMR (100 MHz, $CDCl_3$) δ 20.5, 24.9, 32.2, 37.6, 125.3, 128.2, 129.8, 136.9, 141.7, 162.4, 182.8.

IR (ATR) ν_{max} /cm^{-1} = 2932s, 2858s, 1665l, 1445m, 1227s, 1050s, 811s, 773l, 724s, 669s.

HRMS (ESI): *m/z* calc. for $C_{11}H_{13}OS$ $[M+H]^+$: 193.0682, found: 193.0683.

$[\alpha]^{25}_{589}$ = –209.5° (*c* 1.00, $CHCl_3$).

(–)-(*S*)-6-(Cyclohex-2-en-1-yl)-1-methyl-1*H*-indazole (38)

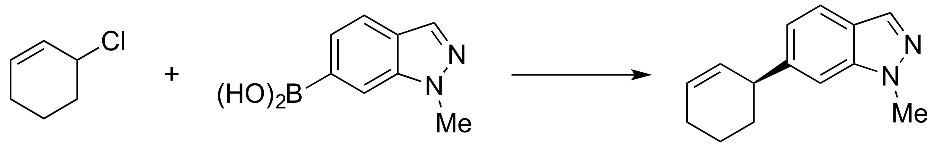

In a 10 mL round bottomed flask $[Rh(cod)(OH)]_2$ (4.6 mg, 0.01 mmol, 0.025 eq), (*S*)-BINAP (14.9 mg, 0.024 mmol, 0.06 eq) and Cs_2CO_3 (130.3 mg, 0.40 mmol, 1.00 eq) were stirred in THF (2.0 mL) at reflux for 30 min. A solution of 3-chlorocyclohexene (45 µL, 0.4 mmol, 1.00 eq) and 1-methyl-1*H*-indazole-6-boronic acid (211.2 mg, 1.20 mmol, 3.00 eq) in THF (1.5 mL) was then added to the flask *via* syringe and rinsed with additional THF (0.5 mL). The reaction mixture was refluxed for 2 h. SiO_2 (20 mg) was added and the solvent was then carefully evaporated. The resulting solid was directly loaded onto a chromatographic column and eluted with petrol ether and ethyl acetate (6:1) to obtain (–)-(*S*)-6-(cyclohex-2-en-1-yl)-1-methyl-1*H*-indazole in 67% yield (57.0 mg, 0.27 mmol) as an off white solid.

HPLC analysis indicated an enantiomeric excess of 99% [Chiralpak® IB; flow: 1.0 mL/min; hexane/*i*-PrOH 95:5; λ = 210 nm; minor enantiomer t_R = 10.8 min; major enantiomer t_R = 11.6 min].

^{1}H-NMR (400 MHz, $CDCl_3$) δ 1.49 – 1.64 (m, 2 H), 1.69 (m, 1 H), 1.94 – 2.11 (m, 3 H), 3.49 (m, 1 H), 3.98 (s, 3 H), 5.71 (dd, *J* = 10.2, 2.4 Hz, 1 H), 5.82 – 5.95 (m, 1 H), 6.96 (dd, *J* = 8.4, 1.3 Hz, 1 H), 7.12 (d, *J* = 1.5 Hz, 1 H), 7.56 (d, *J* = 8.3 Hz, 1 H), 7.84 (s, 1 H).

^{13}C-NMR (100 MHz, $CDCl_3$) δ 21.2, 25.1, 32.8, 35.4, 42.2, 107.2, 120.7, 121.5, 122.6, 128.8, 130.0, 132.4, 140.4, 145.4.

IR (ATR) ν_{max} /cm^{-1} = 2930l, 2859s, 1622m, 1473m, 1440s, 1373s, 1223m, 947s, 839m, 769s.

HRMS (EI/FI): *m/z* calc. for $C_{14}H_{16}N_2$ $[M]^+$: 212.1313, found: 212.1310.

$[\alpha]^{25}_{589}$ = –166.7° (*c* 1.00, $CHCl_3$).

***T*$_{mp}$** = 82 °C.

(–)-(*S*)-2-(Cyclohept-2-en-1-yl)benzofuran (39)

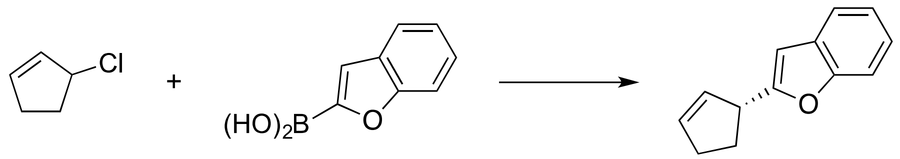

In a 10 mL round bottomed flask $[Rh(cod)(OH)]_2$ (9.2 mg, 0.02 mmol, 0.05 eq), (*R*)-Xyl-P-PHOS (36.4 mg, 0.048 mmol, 0.12 eq) and Cs_2CO_3 (130.3 mg, 0.40 mmol, 1.00 eq) were stirred in THF (2.0 mL) at reflux for 30 min. A solution of 3-chlorocyclopentene (1 M in THF, 0.4 mL, 0.4 mmol, 1.00 eq) and 2-benzofuranylboronic acid (259.2 mg, 1.6 mmol, 4.00 eq) in THF (1.1 mL) was then added *via* syringe and the flask rinsed with additional THF (0.5 mL). The reaction mixture was refluxed for 4 h protected from light. SiO_2 (20 mg) was added and the solvent was then carefully evaporated. The resulting solid was directly loaded onto a chromatographic column and eluted with pentane to obtain (–)-(*S*)-2-(cyclohept-2-en-1-yl)benzofuran in 75% yield (55.3 mg, 0.30 mmol).

HPLC analysis indicated an enantiomeric excess of 99% [Chiralpak® IE; flow: 0.7 mL/min; hexane; λ = 210 nm; minor enantiomer t_R = 11.5 min; major enantiomer t_R = 11.8 min].

^{1}H-NMR (400 MHz, $CDCl_3$) δ 1.96 – 2.11 (m, 1 H), 2.32 – 2.50 (m, 2 H), 2.50 – 2.61 (m, 1 H), 4.04 – 4.12 (m, 1 H), 5.82 – 5.89 (m, 1 H), 5.95 – 6.03 (m, 1 H), 6.38 (s, 1 H), 7.13 – 7.24 (m, 2 H), 7.43 (ddd, *J* = 7.2, 1.7, 0.8 Hz, 1 H), 7.45 – 7.50 (m, 1 H).

^{13}C-NMR (100 MHz, $CDCl_3$) δ 29.9, 32.3, 44.9, 100.9, 111.0, 120.5, 122.5, 123.3, 129.0, 130.8, 133.4, 154.9, 162.1.

IR (ATR) ν_{max} /cm^{-1} = 2944s, 2851s, 1585s, 1454m, 1253m, 1165s, 1010s, 951s, 798s, 742l.

HRMS (ESI): *m/z* calc. for $C_{13}H_{13}O$ $[M+H]^+$: 193.0682, found: 193.0683.

$[\alpha]^{25}_{589}$ = –223.7° (*c* 1.00, $CHCl_3$).

(–)-(*S*)-3-(Cyclopent-2-en-1-yl)thiophene (40)

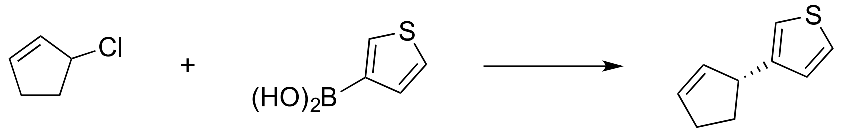

In a 10 mL round bottomed flask [Rh(cod)(OH)]$_2$ (4.6 mg, 0.01 mmol, 0.025 eq), (*S*)-BINAP (14.9 mg, 0.024 mmol, 0.06 eq) and Cs_2CO_3 (130.3 mg, 0.40 mmol, 1.00 eq) were stirred in THF (2.0 mL) at reflux for 30 min. A solution of 3-chlorocyclopentene (1 M in THF, 0.4 mL, 0.4 mmol, 1.00 eq) and 3-thienylboronic acid (153.6 mg, 1.20 mmol, 3.00 eq) in THF (1.1 mL) was then added *via* syringe and the flask rinsed with additional THF (0.5 mL). The reaction mixture was refluxed for 4 h protected from light. SiO_2 (20 mg) was added and the solvent was then carefully evaporated. The resulting solid was directly loaded onto a chromatographic column and eluted with pentane to obtain (–)-(*S*)-3-(cyclopent-2-en-1-yl)thiophene in 70% yield (44.0 mg, 0.28 mmol).

HPLC analysis indicated an enantiomeric excess of 98% [Chiralpak® AY-H; flow: 0.6 mL/min; hexane/*i*-PrOH 99.9:0.1; λ = 210 nm; minor enantiomer t_R = 8.33 min; major enantiomer t_R = 8.75 min].

^{1}H-NMR (400 MHz, $CDCl_3$) δ = 1.64 – 1.76 (m, 1 H), 2.22 – 2.36 (m, 2 H), 2.36 – 2.46 (m, 1 H), 3.91 (m, 1 H), 5.67 – 5.78 (m, 1 H), 5.78 – 5.86 (m, 1 H), 6.81 – 6.92 (m, 2 H), 7.18 (dd, *J* = 4.7, 3.2 Hz, 1 H).

^{13}C-NMR (100 MHz, $CDCl_3$) δ = 32.2, 32.6, 46.3, 119.1, 125.5, 127.3, 131.6, 133.9, 147.1.

IR (ATR) ν_{max} /cm^{-1} = 2943s, 1583s, 1564s, 1451l, 1387s, 1137s, 1105m, 1023s, 830s, 742s, 698.

HRMS (EI): *m/z* calc. for $C_9H_{10}S$ [M]$^+$: 150.0503, found: 150.0494.

$[\alpha]^{25}_{589}$ = –153.9° (*c* 1.00, $CHCl_3$).

(–)-(*S*)-2-(Cyclohept-2-en-1-yl)benzofuran (41)

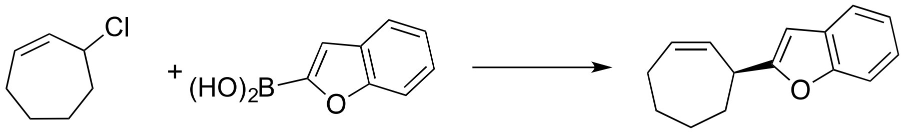

In a 10 mL round bottomed flask [Rh(cod)(OH)]$_2$ (4.6 mg, 0.01 mmol, 0.025 eq), (*S*)-BINAP (14.9 mg, 0.024 mmol, 0.06 eq) and Cs_2CO_3 (130.3 mg, 0.40 mmol, 1.00 eq) were stirred in THF (2.0 mL) at reflux for 30 min. A solution of 3-chlorocycloheptene (52 µL, 0.4 mmol, 1.00 eq) and 2-benzofuranylboronic acid (194.3 mg, 1.20 mmol, 3.00 eq) in THF (1.5 mL) was then added *via* syringe and the flask rinsed with additional THF (0.5 mL). The reaction mixture was refluxed for 2 h. SiO_2 (20 mg) was added and the solvent was then carefully evaporated. The resulting solid was directly loaded onto a chromatographic column and eluted with pentane to obtain (–)-(*S*)-2-(cyclohept-2-en-1-yl)benzofuran in 24% yield (20.4 mg, 0.10 mmol).

HPLC analysis indicated an enantiomeric excess of 99% [Chiralpak® IB; flow: 1.0 mL/min; hexane/*i*-PrOH 99.9:0.1; $\lambda = 210$ nm; minor enantiomer $t_R = 7.4$ min; major enantiomer $t_R = 8.1$ min].

^{1}H-NMR (400 MHz, $CDCl_3$) δ 1.43 – 1.56 (m, 1 H), 1.65 – 1.80 (m, 2 H), 1.82 – 2.00 (m, 2 H), 2.01 – 2.12 (m, 1 H), 2.18 – 2.30 (m, 2 H), 3.70 – 3.86 (m, 1 H), 5.89 – 6.02 (m, 2 H), 6.44 (s, 1 H), 7.13 – 7.25 (m, 2 H), 7.40 – 7.46 (m, 1 H), 7.47 – 7.54 (m, 1 H).

^{13}C-NMR (100 MHz, $CDCl_3$) δ 27.0, 28.8, 29.9, 32.5, 40.5, 101.4, 111.0, 120.5, 122.5, 123.3, 129.0, 132.7, 133.3, 154.8, 162.4.

IR (ATR) ν_{max} /cm^{-1} = 2924l, 2853s, 1584s, 1454l, 1254l, 1167s, 799s, 746l, 688s.

HRMS (EI): *m/z* calc. for $C_{15}H_{16}O$ $[M]^+$: 212.1201, found: 212.1194.

$[\alpha]^{25}_{589}$ = –70.4° (*c* 1.00, $CHCl_3$).

(–)-(*S*)-3-(Cyclohept-2-en-1-yl)thiophene (42)

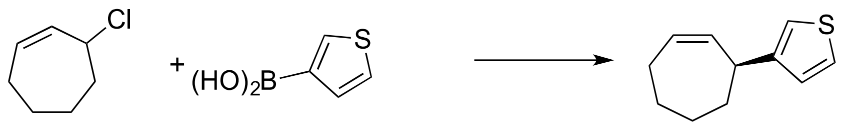

In a 10 mL round bottomed flask $[Rh(cod)(OH)]_2$ (4.6 mg, 0.01 mmol, 0.025 eq), (*S*)-BINAP (14.9 mg, 0.024 mmol, 0.06 eq) and Cs_2CO_3 (130.3 mg, 0.40 mmol, 1.00 eq) were stirred in THF (2.0 mL) at reflux for 30 min. A solution of 3-chlorocycloheptene (52 µL, 0.4 mmol, 1.00 eq) and 3-thienylboronic acid (153.6 mg, 1.20 mmol, 3.00 eq) in THF (1.5 mL) was then added *via* syringe and the flask rinsed with additional THF (0.5 mL). The reaction mixture was refluxed for 2.5 h. SiO_2 (20 mg) was added and the solvent was then carefully evaporated. The resulting solid was directly loaded onto a chromatographic column and eluted with pentane to obtain (–)-(*S*)-3-(cyclohept-2-en-1-yl)thiophene in 35% yield (17.0 mg, 0.14 mmol).

HPLC analysis indicated an enantiomeric excess of 98% [Chiralpak® IB; flow: 0.6 mL/min; hexane/*i*-PrOH 99.9:0.1; $\lambda = 210$ nm; minor enantiomer $t_R = 8.2$ min; major enantiomer $t_R = 8.7$ min].

^{1}H-NMR (400 MHz, $CDCl_3$) δ 1.39 – 1.52 (m, 1 H), 1.61 – 1.83 (m, 3 H), 1.85 – 1.98 (m, 2 H), 2.13 – 2.29 (m, 2 H), 3.63 – 3.74 (m, 1 H), 5.74 – 5.92 (m, 2 H), 6.95 – 7.02 (m, 2 H), 7.23 – 7.29 (m, 1 H).

^{13}C-NMR (100 MHz, $CDCl_3$) δ 27.0, 28.8, 29.9, 35.1, 41.9, 119.2, 125.4, 127.3, 131.6, 136.5, 147.8.
IR (ATR) ν_{max} /cm^{-1} = 3017s, 2920l, 2851s, 1443s, 857s, 842s, 771l, 687s, 648s.

HRMS (EI): *m/z* calc. for $C_{11}H_{14}S$ $[M]^+$: 178.0816, found: 178.0813.

$[\alpha]^{25}_{589}$ = –63.4° (*c* 1.00, $CHCl_3$).

(–)-(*S*)-4-(3,6-Dihydro-2*H*-pyran-3-yl)dibenzofuran (43)

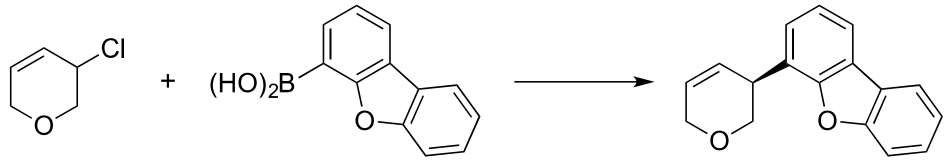

In a 10 mL round bottomed flask $[Rh(cod)(OH)]_2$ (4.6 mg, 0.01 mmol, 0.025 eq), (*S*)-BINAP (14.9 mg, 0.024 mmol, 0.06 eq) and Cs_2CO_3 (130.3 mg, 0.40 mmol, 1.00 eq) were stirred in THF (2.0 mL) at reflux for 30 min. A solution of 3-chloro-3,6-dihydro-2*H*-pyran (45 µL, 0.4 mmol, 1.00 eq) and 4-(dibenzofuranyl)boronic acid (254.4 mg, 1.20 mmol, 3.00 eq) in THF (1.5 mL) was then added *via* syringe and the flask rinsed with additional THF (0.5 mL). The resulting mixture was then stirred for 2 h at reflux before the addition of NaOH (2 M, aq. 0.2 mL). The aqueous phase was extracted with Et_2O (2 × 0.5 mL). The combined organic extracts were washed with water (0.5 mL), dried with $MgSO_4$ and filtered. SiO_2 (20 mg) was added and the solvent was then carefully evaporated. The resulting solid was directly loaded onto a chromatographic column and eluted with petrol ether and ethyl acetate (6:1) to obtain (–)-(*S*)-4-(3,6-dihydro-2*H*-pyran-3-yl)dibenzofuran in 60% yield (59.3mg, 0.24 mmol).

HPLC analysis indicated an enantiomeric excess of 98% [Chiralpak® IB; flow: 1.0 mL/min; hexane/*i*-PrOH 95:5; λ = 210 nm; major enantiomer t_R =6.9 min; minor enantiomer t_R = 7.7 min].

^{1}H-NMR (400 MHz, $CDCl_3$) δ 3.85 (dd, *J* = 10.5, 5.4 Hz, 1 H), 4.21 – 4.34 (m, 4 H), 6.02 – 6.10 (m, 2 H), 7.35 (m, 2 H), 7.41 (dd, *J* = 7.6, 1.4 Hz, 1 H), 7.47 (ddd, *J* = 8.3, 7.8, 1.4 Hz, 1 H), 7.60 (d, *J* = 8.2 Hz, 1 H), 7.85 (dd, *J* = 7.6, 1.4 Hz, 1 H), 7.96 (d, *J* = 7.7 Hz, 1 H).

^{13}C-NMR (100 MHz, $CDCl_3$) δ 35.1, 65.6, 69.6, 111.8, 119.1, 120.8, 122.8, 123.1, 124.0, 124.6, 126.0, 126.4, 126.9, 127.2, 127.8, 154.4, 156.1.

IR (ATR) ν_{max} /cm^{-1} = 2924l, 2853s, 1584s, 1454l, 1254l, 1167s, 799s, 746l, 688s.

HRMS (ESI): *m/z* calc. for $C_{17}H_{15}O_2$ $[M+H]^+$: 251.1068, found: 251.1067.

$[\alpha]^{25}_{589}$ = –70.4° (*c* 1.00, $CHCl_3$).

(–)-(*S*)-3-(Benzo[*b*]thiophen-3-yl)-3,6-dihydro-2*H*-pyran (44)

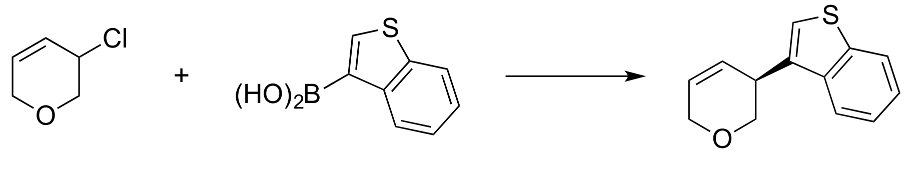

In a 10 mL round bottomed flask $[Rh(cod)(OH)]_2$ (4.6 mg, 0.01 mmol, 0.025 eq), (*S*)-BINAP (14.9 mg, 0.024 mmol, 0.06 eq) and Cs_2CO_3 (130.3 mg, 0.40 mmol, 1.00 eq) were stirred in THF (2.0 mL) at reflux for 30 min. A solution of 3-chloro-3,6-dihydro-2*H*-pyran (45 µL, 0.4 mmol, 1.00 eq) and benzo[*b*]thien-3-ylboronic acid (214 mg, 1.20 mmol, 3.00 eq) in THF (1.5 mL) was added *via* syringe and the flask rinsed with additional THF (0.5 mL). The resulting mixture was then stirred for 2 h at reflux before the addition of NaOH (2 M, aq.

0.2 mL). The aqueous phase was extracted with Et_2O (2 × 0.5 mL). The combined organic extracts were washed with water (0.5 mL), dried with $MgSO_4$ and filtered. SiO_2 (20 mg) was added and the solvent was then carefully evaporated. The resulting solid was directly loaded onto a chromatographic column and eluted with pentane and diethyl ether (95:5) to obtain (–)-(*S*)-3-(benzo[*b*]thiophen-3-yl)-3,6-dihydro-2*H*-pyran in 37% yield (32.0 mg, 0.15 mmol).

HPLC analysis indicated an enantiomeric excess of 82% [Chiralpak® IA; flow: 1.0 mL/min; hexane/*i*-PrOH 99:1; λ = 210 nm; major enantiomer t_R = 6.8 min; minor enantiomer t_R = 7.5 min].

^{1}H-NMR (400 MHz, $CDCl_3$) δ 3.76 (dd, *J* = 11.0, 6.4 Hz, 1 H), 3.93 – 4.01 (m, 1 H), 4.16 (dd, *J* = 10.9, 4.8 Hz, 1 H), 4.24 – 4.30 (m, 2 H), 5.98 (m, 1 H), 6.05 (m, 1 H), 7.22 (s, 1 H), 7.32 – 7.43 (m, 2 H), 7.77 – 7.84 (m, 1 H), 7.84 – 7.92 (m, 1 H).

^{13}C-NMR (100 MHz, $CDCl_3$) δ 35.3, 65.7, 69.4, 121.6, 123.1, 123.2, 124.1, 124.4, 127.2, 127.4, 136.5, 138.3, 140.9.

IR (ATR) ν_{max} /cm^{-1} = 2924s, 2846l, 1457l, 1429m, 1187s, 1102m, 1081m, 765l, 729s, 703s.

HRMS (Ammonia CI): *m*/*z* calc. for $C_{13}H_{13}OS$ $[M+H]^+$: 217.0682, found: 217.0684.

$[\alpha]^{25}_{589}$ = –4.6° (*c* 0.50, $CHCl_3$).

6. Analytical data for figure 3

(–)-(*S*)-2-Chloro-5-(cyclohex-2-en-1-yl)-pyridine (45a)

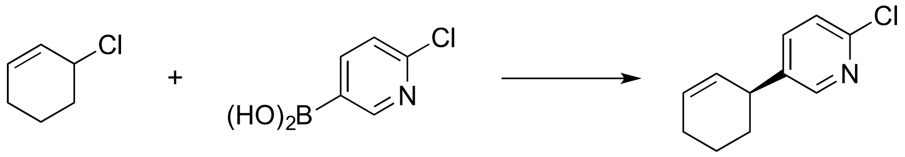

In a 10 mL round bottomed flask [Rh(cod)(OH)]$_2$ (4.6 mg, 0.01 mmol, 0.025 eq), (*S*)-BINAP (14.9 mg, 0.024 mmol, 0.06 eq) and Cs_2CO_3 (390.9 mg, 1.20 mmol, 3.00 eq) were stirred in THF (2.0 mL) at reflux for 30 min. A solution of 3-chlorocyclohexene (45 µL, 0.4 mmol, 1.00 eq) and 6-chloro-3-pyridinylboronic acid (188.8 mg, 1.20 mmol, 3.00 eq) in THF (1.5 mL) was then added *via* syringe and the flask rinsed with additional THF (0.5 mL). The reaction mixture was refluxed for 2 h. SiO_2 (20 mg) was added and the solvent was then carefully evaporated. The resulting solid was directly loaded onto a chromatographic column and eluted with petrol ether and ethyl acetate (97:3) to obtain (–)-(*S*)-2-chloro-5-(cyclohex-2-en-1-yl)-pyridine in 57% yield (44.4 mg, 0.23 mmol).

HPLC analysis indicated an enantiomeric excess of greater than 99% [Chiralpak® AY-H; flow: 1.5 mL/min; hexane/*i*-PrOH 99.4:0.6; λ = 210 nm; minor enantiomer t_R = 13.4 min; major enantiomer, t_R = 16.2 min].

^{1}H-NMR (400 MHz, $CDCl_3$) δ 1.49 (dddd, *J* = 13.1, 10.2, 8.1, 3.1 Hz, 1 H), 1.56 – 1.76 (m, 2 H), 1.95 – 2.06 (m, 1 H), 2.06 – 2.14 (m, 2 H), 3.42 (m, 1 H), 5.58 – 5.67 (m, 1 H), 5.91 – 6.00 (m, 1 H), 7.24 (d, *J* = 8.2 Hz, 1 H), 7.49 (dd, *J* = 8.2, 2.6 Hz, 1 H), 8.23 (d, *J* = 2.6 Hz, 1 H).

^{13}C-NMR (100 MHz, $CDCl_3$) δ 20.8, 24.9, 32.4, 38.7, 124.0, 128.3, 130.0, 138.2, 140.8, 149.2, 149.4.

IR (ATR) ν_{max} /cm^{-1} = 2930m, 2861s, 1564s, 1455l, 1379s, 1137s, 1105l, 833s, 629s.

HRMS (ESI): *m/z* calc. for $C_{11}H_{13}ClN$ $[M+H]^+$: 194.0731, found: 194.0733.

$[\alpha]^{25}_{589}$ = –116.2° (*c* 1.00, $CHCl_3$).

(–)-(*S*)-2-Chloro-6-(cyclohex-2-en-1-yl)-pyridine (45b)

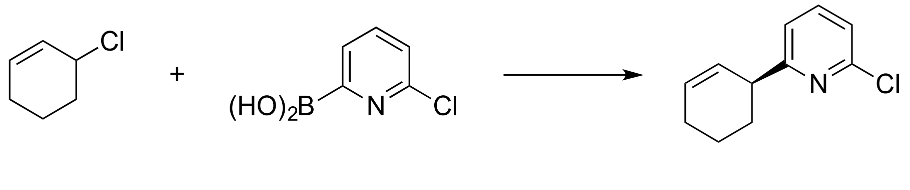

6-Chloropyridinylboronic acid was prepared according to the literature.[4] In a 25 mL round bottomed flask [Rh(cod)(OH)]$_2$ (11.5 mg, 0.025 mmol, 0.0125 eq), (*S*)-BINAP (37.3 mg, 0.06 mmol, 0.03 eq) and Cs_2CO_3 (1.95 g, 6.0 mmol, 3.00 eq) were stirred in THF (5.0 mL) at reflux for 30 min. 3-Chlorocyclohexene (225 µL, 2.0 mmol, 1.00 eq) was added to a sonicated solution of 6-chloro-2-pyridinylboronic acid (944 mg, 6.0 mmol, 3.00 eq) and water (108 µL, 6.0 mmol, 3.00 eq) in THF (8 mL). The resulting mixture was added *via* syringe and the flask

rinsed with additional THF (2 mL). The reaction mixture was refluxed for 12 h. SiO_2 (20 mg) was added and the solvent was then carefully evaporated. The resulting solid was directly loaded onto a chromatographic column and eluted with petrol ether and ethyl acetate (97:3) to obtain (–)-(*S*)-2-chloro-6-(cyclohex-2-en-1-yl)-pyridine in 63% yield (245 mg, 1.27 mmol).

HPLC analysis indicated an enantiomeric excess of 97% [Chiralpak® ID; flow: 1.0 mL/min; hexane/*i*-PrOH 99.7:0.3; λ = 210 nm; major enantiomer t_R = 6.8 min; minor enantiomer t_R = 8.0 min].

^{1}H-NMR (400 MHz, $CDCl_3$) δ 1.59 – 1.75 (m, 3 H), 2.03 – 2.13 (m, 3 H), 3.56 (m, 1 H), 5.71 – 5.79 (m, 1 H), 5.93 (dtd, *J* = 9.8, 3.6, 3.6, 2.3 Hz, 1 H), 7.11 (dd, *J* = 7.6, 0.9 Hz, 1 H), 7.14 (dd, *J* = 7.9, 0.9 Hz, 1 H), 7.56 (dd, *J* = 7.9, 7.6 Hz, 1 H).

^{13}C-NMR (100 MHz, $CDCl_3$) δ 20.9, 25.1, 30.5, 43.7, 120.2, 121.7, 128.0, 129.7, 139.0, 150.8, 166.7.

IR (ATR) ν_{max} /cm^{-1} = 2931s, 1581m, 1557m, 1435l, 1156s, 1133m, 791m, 727m, 663s.

HRMS (ESI): *m/z* calc. for $C_{11}H_{13}ClN$ [M+H]$^+$: 194.0731, found: 194.0733.

$[\alpha]^{25}_{589}$ = –72.0° (*c* 1.00, $CHCl_3$).

(–)-(*S*)-2-Chloro-4-(cyclohex-2-en-1-yl)-pyridine (45c)

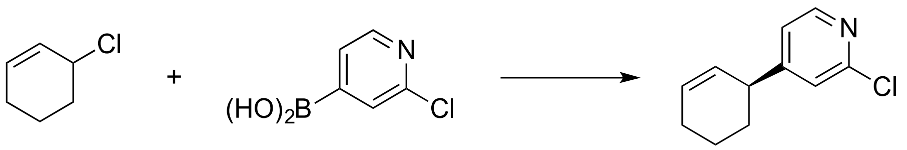

In a 10 mL round bottomed flask $[Rh(cod)(OH)]_2$ (4.6 mg, 0.01 mmol, 0.025 eq), (*S*)-BINAP (14.9 mg, 0.024 mmol, 0.06 eq) and Cs_2CO_3 (130.3 mg, 0.40 mmol, 1.00 eq) were stirred in THF (2.0 mL) at reflux for 30 min. A solution of 3-chlorocyclohexene (45 µL, 0.4 mmol, 1.00 eq) and 2-chloro-4-pyridinylboronic acid (188.8 mg, 1.20 mmol, 3.00 eq) in THF (1.5 mL) was then added *via* syringe and the flask rinsed with additional THF (0.5 mL). The reaction mixture was refluxed for 12 h. SiO_2 (20 mg) was added and the solvent was then carefully evaporated. The resulting solid was directly loaded onto a chromatographic column and eluted with petrol ether and ethyl acetate (97:3) to obtain (–)-(*S*)-2-chloro-4-(cyclohex-2-en-1-yl)-pyridine in 44% yield (34.3 mg, 0.18 mmol).

HPLC analysis indicated an enantiomeric excess of 99% [Chiralpak® IA; flow: 1.0 mL/min; hexane/*i*-PrOH 99.4:0.6; λ = 210 nm; minor enantiomer (+)-(*R*)-2-chloro-4-(cyclohex-2-en-1-yl)-pyridine, t_R = 8.4 min; major enantiomer (–)-(*S*)-2-chloro-4-(cyclohex-2-en-1-yl)-pyridine, t_R = 9.2 min].

^{1}H-NMR (400 MHz, $CDCl_3$) δ 1.44 – 1.80 (m, 3 H), 1.93 – 2.05 (m, 1 H), 2.05 – 2.14 (m, 2 H), 3.29 – 3.44 (m, 1 H), 5.62 (dt, *J* = 10.1, 2.5, 2.5 Hz, 1 H), 5.90 – 6.02 (m, 1 H), 7.06 (dd, *J* = 5.2, 1.6 Hz, 1 H), 7.17 (d, *J* = 1.6 Hz, 1 H), 8.26 (d, *J* = 5.1 Hz, 1 H).

^{13}C-NMR (100 MHz, $CDCl_3$) δ 20.8, 24.9, 31.7, 41.0, 122.1, 123.6, 127.4, 130.4, 149.6, 151.7, 159.0.

IR (ATR) ν_{max} /cm^{-1} = 2931s, 1590l, 1544m, 1380m, 1121s, 1184m, 911s, 733m, 722m, 679s;

HRMS (ESI): *m/z* calc. for $C_{11}H_{13}ClN$ $[M+H]^+$: 194.0731, found: 194.0732.

$[\alpha]^{25}_{589}$ = –117.3° (*c* 1.00, $CHCl_3$).

(–)-(*S*)-2-Chloro-3-(cyclohex-2-en-1-yl)-pyridine (45d)

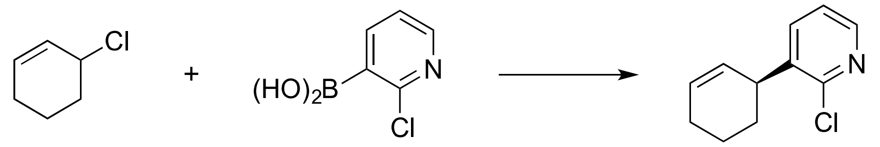

In a 10 mL round bottomed flask $[Rh(cod)(OH)]_2$ (4.6 mg, 0.01 mmol, 0.025 eq), (*S*)-BINAP (14.9 mg, 0.024 mmol, 0.06 eq) and Cs_2CO_3 (130.3 mg, 0.40 mmol, 1.00 eq) were stirred in THF (2.0 mL) at reflux for 30 min. A solution of 3-chlorocyclohexene (45 µL, 0.4 mmol, 1.00 eq) and 2-chloro-3-pyridinylboronic acid (188.8 mg, 1.20 mmol, 3.00 eq) in THF (1.5 mL) was then added *via* syringe and the flask rinsed with additional THF (0.5 mL). The reaction mixture was refluxed for 12 h. SiO_2 (20 mg) was added and the solvent was then carefully evaporated. The resulting solid was directly loaded onto a chromatographic column and eluted with petrol ether and ethyl acetate (97:3) to obtain (–)-(*S*)-2-chloro-3-(cyclohex-2-en-1-yl)-pyridine in 26% yield (20.1 mg, 0.10 mmol).

HPLC analysis indicated an enantiomeric excess of 97% [Chiralpak® IB; flow: 1.0 mL/min; hexane/*i*-PrOH 99.4:0.6; λ = 210 nm; minor enantiomer t_R = 6.7 min; major enantiomer t_R = 7.2 min].

^{1}H-NMR (400 MHz, $CDCl_3$) δ 1.44 – 1.55 (m, 1 H), 1.60 – 1.68 (m, 2 H), 2.04 – 2.19 (m, 3 H), 3.82 (m, 1 H), 5.60 (ddt, *J* = 10.1, 2.5, 2.4, 2.4 Hz, 1 H), 6.00 (dtd, *J* = 9.9, 3.7, 3.7, 2.2 Hz, 1 H), 7.19 (dd, *J* = 7.6, 4.7 Hz, 1 H), 7.59 (dd, *J* = 7.6, 2.0 Hz, 1 H), 8.23 (dd, *J* = 4.7, 1.9 Hz, 1 H).

^{13}C-NMR (100 MHz, $CDCl_3$) δ 20.5, 25.0, 29.6, 37.9, 122.7, 128.0, 130.4, 138.0, 140.0, 147.2, 151.2.

IR (ATR) ν_{max} /cm^{-1} = 2929s, 1561m, 1405l, 1071m, 1060m, 806s, 789m, 723m, 652m.
HRMS (ESI): *m/z* calc. for $C_{11}H_{13}ClN$ $[M+H]^+$: 194.0731, found: 194.0733.

$[\alpha]^{25}_{589}$ = –17.2° (*c* 1.00, $CHCl_3$).

(–)-(*S*)-2-Fluoro-5-(cyclohex-2-en-1-yl)-pyridine (46)

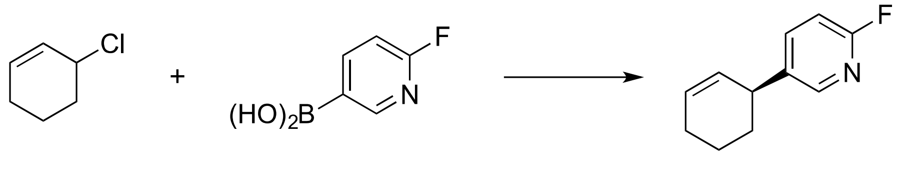

In a 10 mL round bottomed flask $[Rh(cod)(OH)]_2$ (4.6 mg, 0.01 mmol, 0.025 eq), (*S*)-BINAP (14.9 mg, 0.024 mmol, 0.06 eq) and Cs_2CO_3 (130.3 mg, 0.40 mmol, 1.00 eq) were stirred in THF (2.0 mL) at reflux for 30 min. A solution of 3-chlorocyclohexene (45 µL, 0.4 mmol, 1.00 eq) and 6-fluoro-3-pyridinylboronic acid (134.3 mg, 1.20 mmol, 3.00 eq) in THF (1.5 mL)

was then added *via* syringe and the flask rinsed with additional THF (0.5 mL). The reaction mixture was refluxed for 2 h. SiO_2 (20 mg) was added and the solvent was then carefully evaporated. The resulting solid was directly loaded onto a chromatographic column and eluted with petrol ether and ethyl acetate (97:3) to afford in 52% yield (30.4 mg, 0.21 mmol).

HPLC analysis indicated an enantiomeric excess of 97% [Chiralpak® ID; flow: 1.0 mL/min; hexane/*i*-PrOH 99.7:0.3; λ = 210 nm; minor enantiomer t_R = 15.5 min; major enantiomer t_R = 17.2 min].

^{1}H-NMR (400 MHz, $CDCl_3$) δ 1.44 (dddd, J = 13.1, 10.2, 8.1, 3.1 Hz, 1 H), 1.49 – 1.62 (m, 1 H), 1.58 – 1.72 (m, 1 H), 1.89 – 2.01 (m, 1 H), 1.98 – 2.08 (m, 2 H), 3.37 (m, 1 H), 5.50 – 5.65 (m, 1 H), 5.88 (dtd, J = 9.9, 3.7, 3.7, 2.3 Hz, 1 H), 6.79 (dd, J = 8.4 Hz, $J_{H\text{-}F}$ = 3.0 Hz, 1 H), 7.55 (td, J = 8.1, 8.1, 2.6 Hz, 1 H), 7.98 (d, J = 2.5 Hz, 1 H).

^{13}C-NMR (100 MHz, $CDCl_3$) δ 20.7, 24.8, 32.4, 38.5, 109.0 (d, $J_{C\text{-}F}$ = 37.3 Hz), 128.6, 129.6, 139.3 (d, $J_{C\text{-}F}$ = 4.5 Hz), 140.4 (d, $J_{C\text{-}F}$ = 7.7 Hz), 146.7 (d, $J_{C\text{-}F}$ = 14.3 Hz), 162.4 (d, $J_{C\text{-}F}$=236.9 Hz).
^{19}F-NMR (376 MHz, $CDCl_3$) δ = -72.18 (d, J = 8.1 Hz).

IR (ATR) ν_{max} /cm^{-1} = 2931s, 1670s, 1593m, 1483l, 1385s, 1250s, 829s, 656s.

HRMS (ESI): *m/z* calc. for $C_{11}H_{13}FN$ $[M+H]^+$: 178.1027, found: 178.1027.

$[\alpha]^{25}_{589}$ = –66.4° (*c* 1.00, $CHCl_3$).

(–)-(*S*)-2-Chloro-6-(3,6-dihydro-2*H*-pyran-3-yl)pyridine (47)

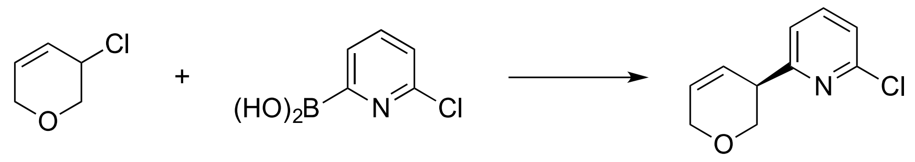

In a 10 mL round bottomed flask $[Rh(cod)(OH)]_2$ (4.6 mg, 0.01 mmol, 0.025 eq), (*S*)-BINAP (14.9 mg, 0.024 mmol, 0.06 eq) and Cs_2CO_3 (390.9 mg, 1.20 mmol, 3.00 eq) were stirred in THF (2 mL) at reflux for 30 min. 3-Chloro-3,6-dihydro-2*H*-pyran (45 µL, 0.4 mmol, 1.00 eq) was added to a sonicated solution of 6-chloro-2-pyridinylboronic acid (189 mg, 1.20 mmol, 3.00 eq) and water (22 µL, 1.20 mmol, 3.00 eq) in THF (1.5 mL). The resulting mixture was added *via* syringe and the flask rinsed with additional THF (0.5 mL). The reaction mixture was refluxed for 2 h. SiO_2 (20 mg) was added and the solvent was then carefully evaporated. The resulting solid was directly loaded onto a chromatographic column and eluted with petrol ether and ethyl acetate (9:1) to obtain (–)-(*S*)-2-chloro-6-(3,6-dihydro-2*H*-pyran-3-yl)pyridine in 74% yield (58.1 mg, 0.30 mmol).

HPLC analysis indicated an enantiomeric excess of 99% [Chiralpak® IA; flow: 1.2 mL/min; hexane/*i*-PrOH 99.2:0.8; λ = 210 nm; major enantiomer t_R = 7.8 min; minor enantiomer t_R = 11.0 min].

^{1}H-NMR (400 MHz, $CDCl_3$) δ 3.56 – 3.65 (m, 1H), 3.88 (dd, J = 11.2, 4.7 Hz, 1H), 4.04 (dd, J = 11.2, 4.7 Hz, 1H), 4.19 – 4.23 (m, 2H), 5.92 – 6.03 (m, 2H), 7.18 (dd, J = 7.9, 0.9 Hz, 1H), 7.23 (dd, J = 7.6, 0.9 Hz, 1H), 7.59 (t, J = 7.7, 7.7 Hz, 1H).

^{13}C-NMR (100 MHz, $CDCl_3$) δ 43.1, 65.6, 69.3, 121.0, 122.3, 125.6, 128.3, 139.2, 150.9, 162.9.

IR (ATR) ν_{max} /cm^{-1} = 1582l, 1559l, 1436l, 1411m, 1157m, 1135l, 1114m, 1088l, 795ls, 744m.

HRMS (ESI): *m*/*z* calc. for $C_{10}H_{11}ClNO$ [M+H]$^+$: 196.0524, found: 196.0524.

$[\alpha]^{25}_{589}$ = –97.4° (*c* 1.00, $CHCl_3$).

(+)-(*S*)-2-Chloro-6-(cyclohept-2-en-1-yl)-pyridine (48)

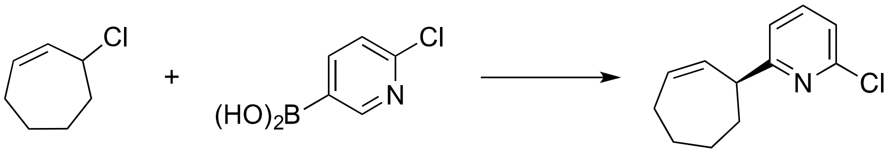

In a 10 mL round bottomed flask $[Rh(cod)(OH)]_2$ (9.2 mg, 0.02 mmol, 0.05 eq), (*S*)-BINAP (29.8 mg, 0.048 mmol, 0.12 eq) and Cs_2CO_3 (390.9 mg, 1.20 mmol, 3.00 eq) were stirred in THF (2 mL) at reflux for 30 min. 3-Chlorocycloheptene (52 µL, 0.4 mmol, 1.00 eq) was added to a sonicated solution of 6-chloro-2-pyridinylboronic acid (189 mg, 1.20 mmol, 3.00 eq) and water (22 µL, 1.20 mmol, 3.00 eq) in THF (1.5 mL). The resulting mixture was added *via* syringe and the flask rinsed with additional THF (0.5 mL). The reaction mixture was refluxed for 2 h. SiO_2 (20 mg) was added and the solvent was then carefully evaporated. The resulting solid was directly loaded onto a chromatographic column and eluted with petrol ether and ethyl acetate (97:3) to obtain (+)-(*S*)-2-chloro-6-(cyclohept-2-en-1-yl)-pyridine in 22% yield (18.3 mg, 0.09 mmol).

HPLC analysis indicated an enantiomeric excess of 85% [Chiralpak® ID; flow: 1.0 mL/min; hexane/*i*-PrOH 99.7:0.3; λ = 210 nm; major enantiomer t_R = 6.6 min; minor enantiomer t_R = 7.4 min].

^{1}H-NMR (400 MHz, $CDCl_3$) δ 1.37 – 1.50 (m, 1H), 1.63 – 1.88 (m, 4H), 1.89 – 1.99 (m, 1H), 2.16 – 2.28 (m, 2H), 3.66 – 3.76 (m, 1H), 5.75 – 5.84 (m, 1H), 5.91 (dddd, *J* = 11.8, 6.5, 5.3, 2.3 Hz, 1 H), 7.10 – 7.17 (m, 2H), 7.57 (t, *J* = 7.7, 7.7 Hz, 1H).

^{13}C-NMR (100 MHz, $CDCl_3$) δ 26.9, 28.9, 30.4, 34.8, 48.9, 120.0, 121.7, 132.9, 134.4, 139.2, 150.7, 167.5.

IR (ATR) ν_{max} /cm^{-1} = 2923l, 2851s 1583l, 1559l, 1439l, 1416m, 1157m, 1134m, 795m, 721s.

HRMS (CI): *m*/*z* calc. for $C_{12}H_{15}ClN$ [M+H]$^+$: 208.0888, found: 208.0890.

$[\alpha]^{25}_{589}$ = +1.9° (*c* 1.00, $CHCl_3$)

(–)-(*S*)-2-Chloro-6-(cyclopent-2-en-1-yl)-pyridine (49)

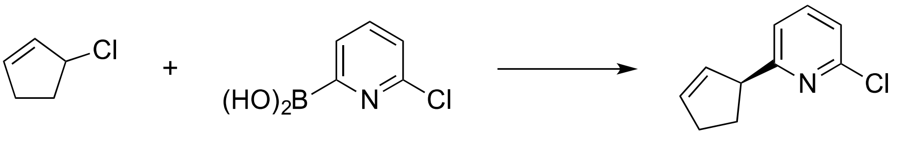

In a 10 mL round bottomed flask $[Rh(cod)(OH)]_2$ (9.2 mg, 0.02 mmol, 0.05 eq), (*S*)-BINAP (29.8 mg, 0.048 mmol, 0.12 eq) and Cs_2CO_3 (390.9 mg, 1.20 mmol, 3.00 eq) were stirred in THF (2 mL) at 60 °C for 30 min. 3-Chloropentene (1 M in THF, 0.4 mL, 0.4 mmol, 1.00 eq) was added to a sonicated solution of 6-chloro-2-pyridinylboronic acid (189 mg, 1.20 mmol, 3.00 eq) and water (22 µL, 1.20 mmol, 3.00 eq) in THF (1.5 mL). The resulting mixture was added *via* syringe and the flask rinsed with additional THF (0.5 mL). The reaction mixture was refluxed for 2 h. SiO_2 (20 mg) was added and the solvent was then carefully evaporated. The resulting solid was directly loaded onto a chromatographic column and eluted with petrol ether and ethyl acetate (97:3) to obtain (–)-(*S*)-2-chloro-6-(cyclopent-2-en-1-yl)-pyridine in 28% yield (20.1 mg, 0.11 mmol).

HPLC analysis indicated an enantiomeric excess of 83% [Chiralpak® ID; flow: 1.0 mL/min; hexane/*i*-PrOH 99.7:0.3; λ = 210 nm; major enantiomer t_R = 8.0 min; minor enantiomer t_R = 8.8 min].

^{1}H-NMR (400 MHz, $CDCl_3$) δ 1.79 – 1.96 (m, 1H), 2.31 – 2.60 (m, 3H), 3.96 – 4.14 (m, 1H), 5.75 – 5.86 (m, 1H), 5.92 – 6.04 (m, 1H), 7.06 (d, *J* = 7.6 Hz, 1H), 7.14 (dd, *J* = 7.8, 1.0 Hz, 1H), 7.55 (dd, *J* = 7.7, 7.7 Hz, 1H).

^{13}C-NMR (100 MHz, $CDCl_3$) δ 32.0, 32.6, 53.5, 119.7, 121.7, 132.4, 133.6, 139.2, 150.7, 166.9.

IR (ATR) ν_{max} /cm^{-1} = 2941s, 1582l, 1558l, 1437l, 1409m, 1159s, 1133m, 795m, 745m, 729m.

HRMS (EI/FI): *m/z* calc. for $C_{10}H_{10}ClN$ $[M]^+$: 179.0502, found: 179.0489.

$[\alpha]^{25}_{589}$ = –117.5° (*c* 1.00, $CHCl_3$).

7. Analytical data for figure 4

***N-tert*-Butoxycarbonyl-5-chloro-3-piperidene:**

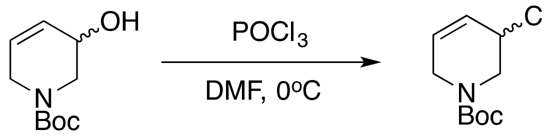

$POCl_3$ (9.7 mL, 104 mmol, 2.20 eq) was added dropwise at 0 °C to a solution of *N-tert*-butoxycarbonyl-5-hydroxy-3-piperidene (9.4 g, 47 mmol, 1.00 eq) in DMF (97 mL). The reaction mixture was stirred overnight at room temperature before the addition of NaOH (2 M, aq, 30 mL) at 0 °C. The aqueous phase was extracted with EtOAc and the combined organic layers were dried over $MgSO_4$, filtered, concentrated *in vacuo* and purified by flash column chromatography. The column was eluted with pentane:Et_2O (9:1) to obtain *N-tert*-butoxycarbonyl-5-chloro-3-piperidene in 67% yield (6.9 g, 31 mmol).

^{1}H NMR (400 MHz, Chloroform-*d*) δ 1.47 (s, 9H), 3.55 - 4.17 (rotameric m, 4H), 4.50 (s, 1H), 5.76 – 5.95 (m, 2H).

^{13}C NMR (101 MHz, Chloroform-*d*) δ 28.4 (3C), 42.4 and 43.1 (rotameric, 1C), 47.0 and 48.3 (rotameric, 1C), 51.7, 80.3, 126.7, 128.4, 154.5.

IR (ATR) ν_{max} /cm^{-1} = 2977 (s), 1698 (l), 1418 (m), 1368 (m), 1336 (s), 1243 (m), 1170 (m), 1123 (m), 1061 (s), 1010 (s), 859 (s), 824 (s), 766 (s), 739 (m), 648 (s).

HRMS (CI): *m/z* calc. for $C_{10}H_{17}O_2NCl$ $[M+H]^+$: 218.0942, found: 218.0942.

(+)-(R)-*N*-tert-Butoxycarbonyl-5-phenyl-3-piperidene (50)

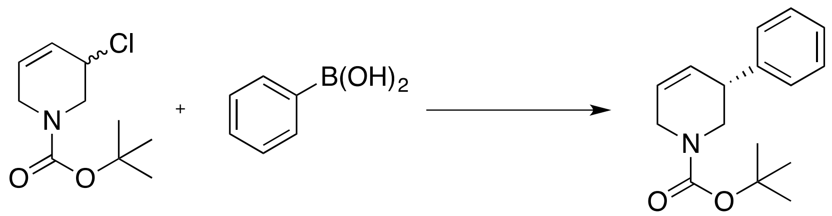

In a 10 mL round bottomed flask $[Rh(cod)(OH)]_2$ (4.6 mg, 0.01 mmol, 0.025 eq), (*R*)-(+)-5,5′-dichloro-2,2′-bis(diphenylphosphino)-6,6′-dimethoxy-1,1′-biphenyl (15.6 mg, 0.024 mmol, 0.06 eq) and Cs_2CO_3 (130.3 mg, 0.40 mmol, 1.00 eq) were stirred in THF (2 mL) at 60 °C for 30 min. A solution of *N-tert*-butoxycarbonyl-5-chloro-3-piperidene (86.8 mg, 0.40 mmol, 1.00 eq) in THF (0.75 mL) and a solution of phenylboronic acid (97.5 mg, 0.80 mmol, 2.00 eq) in THF (0.75 mL) were sequentially added *via* syringe and both flasks were rinsed with THF (0.25 mL each). The resulting mixture was then stirred for 16 h at 80 °C in a sealed flask. SiO_2 (20 mg) was added and the solvent was then carefully evaporated. The resulting solid was directly loaded onto a chromatographic column and eluted with pentane:Et_2O (9:1) to afford (+)-(R)-*N*-tert-butoxycarbonyl-5-phenyl-3-piperidene in 76% yield (79.1 mg, 0.31 mmol).

Enantiomeric excess of 96% was determined by HPLC [Chiralpak® IA; flow: 1.0 mL/min; hexane/*i*-PrOH: 99.4: 0.6; λ = 210 nm; major enantiomer t_R = 7.5 min; minor enantiomer t_R = 8.1 min].

^{1}H NMR (400 MHz, Chloroform-*d*) δ 1.19 – 1.63 (m, 9H), 3.01, 3.33 – 3.46, 3.70 – 3.79 and 4.07 (rotameric m, 2H), 3.46 – 3.62 (m, 1H), 3.82 and 4.07 (rotameric m, 2H), 5.89 (br s, 2H), 7.19 – 7.27 (m, 3H), 7.31 (m, 2H).

^{13}C NMR (101 MHz, Chloroform-*d*) δ 28.3 (3C), 41.5, 42.9 and 43.6 (rotameric, 1C), 47.3 and 48.5 (rotameric, 1C), 79.5, 125.9, 126.7, 127.9 (2C), 128.2, 128.5 (2C), 142.2, 154.7.

IR (ATR) ν_{max} /cm^{-1} = 2975 (s), 1696 (l), 1452 (m), 1420 (m), 1366 (s), 1299 (m), 1237 (l), 1168 (s), 1113 (m).

HRMS (ESI): *m/z* calc. for $C_{16}H_{21}O_2NNa^+$ $[M+Na]^+$: 282.1465, found: 282.1466.

$[\alpha]^{25}_{589}$ = +112.9° (*c* 1.00, $CHCl_3$).

(+)-(*R*)-*N-tert*-Butoxycarbonyl-5-(naphthalen-2-yl)-3-piperidene (51)

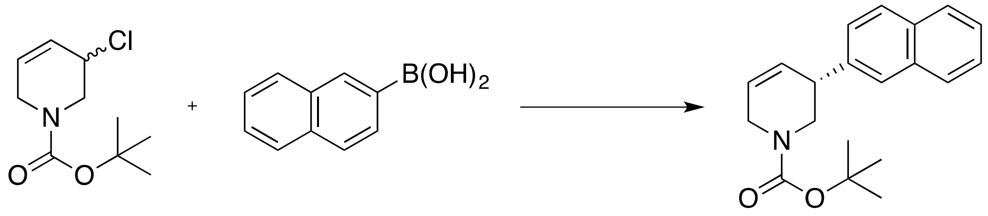

In a 10 mL round bottomed flask $[Rh(cod)(OH)]_2$ (4.6 mg, 0.01 mmol, 0.025 eq), (*R*)-(+)-5,5′-dichloro-2,2′-bis(diphenylphosphino)-6,6′-dimethoxy-1,1′-biphenyl (15.6 mg, 0.024 mmol, 0.06 eq) and Cs_2CO_3 (130.3 mg, 0.40 mmol, 1.00 eq) were stirred in THF (2 mL) at 60 °C for 30 min. A solution of *N-tert*-butoxycarbonyl-5-chloro-3-piperidene (86.8 mg, 0.40 mmol, 1.00 eq) in THF (0.75 mL) and a solution of naphthalene-2-boronic acid (137.6 mg, 0.80 mmol, 2.00 eq) in THF (0.75 mL) were sequentially added *via* syringe and both flasks were rinsed with THF (0.25 mL each). The resulting mixture was then stirred for 16 h at 80 °C in a sealed flask. SiO_2 (20 mg) was added and the solvent was then carefully evaporated. The resulting solid was directly loaded onto a chromatographic column and eluted with pentane:Et_2O (9:1) to afford (+)-(*R*)-*N-tert*-butoxycarbonyl-5-(naphthalen-2-yl)-3-piperidene in 79% yield (98 mg, 0.32 mmol).

Enantiomeric excess of 95% was determined by HPLC [Chiralpak® IA; flow: 1.0 mL/min; hexane/*i*-PrOH: 85: 15; λ = 210 nm; minor enantiomer t_R = 6.6 min; major enantiomer t_R = 13.2 min].

^{1}H NMR (400 MHz, Chloroform-*d*) δ 1.21 – 1.57 (m, 9H), 3.14, 3.44 – 3.61, 3.83 and 4.13 (rotameric m, 2H), 3.68 (s, 1H), 3.83 and 4.13 (rotameric m, 2H), 5.99 (m, 2H), 7.37 (dd, *J* = 8.5, 1.8 Hz, 1H), 7.42 – 7.50 (m, 2H), 7.66 (s, 1H), 7.81 (m, 3H).

^{13}C NMR (101 MHz, Chloroform-*d*) δ 28.3 (d, *J* = 19.9 Hz, 3C), 41.7, 43.0 and 43.7 (rotameric, 1C), 47.2 and 48.4 (rotameric, 1C), 79.5, 125.5 (2C), 126.0, 126.3, 126.4, 127.6, 127.7 (2C), 128.1, 132.5, 133.5, 139.6, 154.7.

IR (ATR) ν_{max} /cm^{-1} = 2974 (s), 2929 (s), 1695 (l), 1420 (s), 1389 (m), 1365 (s), 1331 (m), 1301 (s), 1239 (m), 1164 (m), 1110 (s), 1016 (s), 858 (s), 817 (s), 748 (m).

HRMS (ESI): *m/z* calc. for $C_{20}H_{23}O_2NNa$ $[M+Na]^+$: 332.1621, found: 332.1623.

[α]^{25}$_{589}$ = +153.7° (*c* 1.00, $CHCl_3$).

(+)-(*R*)-*N-tert*-Butoxycarbonyl-5-(4-methylphenyl)-3-piperidene (52)

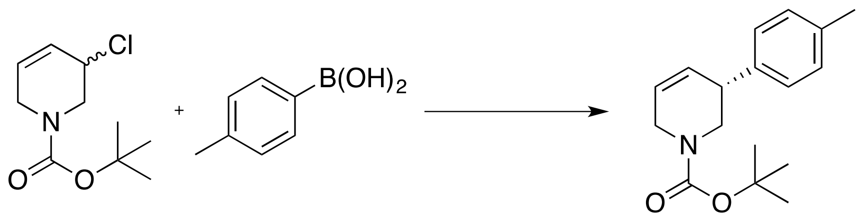

In a 10 mL round bottomed flask $[Rh(cod)(OH)]_2$ (4.6 mg, 0.01 mmol, 0.025 eq), (*R*)-(+)-5,5′-dichloro-2,2′-bis(diphenylphosphino)-6,6′-dimethoxy-1,1′-biphenyl (15.6 mg, 0.024 mmol, 0.06 eq) and Cs_2CO_3 (130.3 mg, 0.40 mmol, 1.00 eq) were stirred in THF (2 mL) at 60 °C for 30 min. A solution of *N-tert*-butoxycarbonyl-5-chloro-3-piperidene (86.8 mg, 0.40 mmol, 1.00 eq) in THF (0.75 mL) and a solution of 4-methylbenzeneboronic acid (108.8 mg, 0.80 mmol, 2.00 eq) in THF (0.75 mL) were sequentially added *via* syringe and both flasks were rinsed with THF (0.25 mL each). The resulting mixture was then stirred for 16 h at 80 °C in a sealed flask. SiO_2 (20 mg) was added and the solvent was then carefully evaporated. The resulting solid was directly loaded onto a chromatographic column and eluted with pentane:Et_2O (9:1) to afford (+)-(*R*)-*N-tert*-butoxycarbonyl-5-(4-methylphenyl)-3-piperidene in 81% yield (89 mg, 0.32 mmol).

Enantiomeric excess of 92% was determined by HPLC [Chiralpak® IB; flow: 1.0 mL/min; hexane/*i*-PrOH: 99: 1; λ = 210 nm; major enantiomer t_R = 4.9 min; minor enantiomer t_R = 5.4 min].

^{1}H NMR (400 MHz, Chloroform-*d*) δ 1.26 – 1.54 (m, 9H), 2.33 (s, 3H), 2.97, 3.31, 3.78 and 4.06 (rotameric m, 2H), 3.49 (m, 1H), 3.78 and 4.06 (rotameric m, 2H), 5.88 (br s, 2H), 7.08 – 7.15 (m, 4H).

^{1}H VT NMR (500 MHz, DMSO-*d*$_6$, 363 K) δ 1.33 (s, 9H), 2.28 (s, 3H), 3.30 (m, 1H), 3.46 (m, 1H), 3.70 (dd, *J* = 12.9, 4.8 Hz, 1H), 3.87 – 3.96 (m, 2H), 5.81 – 5.86 (m, 1H), 5.87 – 5.92 (m, 1H), 7.10 (m, 4H).

^{13}C NMR (101 MHz, Chloroform-*d*) δ 21.0, 28.3 (3C), 41.1, 42.9 and 43.6 (rotameric, 1C), 47.4 and 48.6 (rotameric, 1C), 79.4, 124.8 and 125.6 (rotameric, 1C), 127.7 (2C), 128.6 and 129.5 (rotameric, 1C), 129.2 (2C), 136.2, 139.2, 154.7.

^{13}C VT NMR (126 MHz, DMSO-*d*$_6$, 363 K) δ 20.9, 28.5 (3C), 40.9, 43.5, 47.9, 79.1, 125.8, 127.9 (2C), 128.9, 129.3 (2C), 136.0, 139.7, 154.4.

IR (ATR) ν_{max} /cm^{-1} = 2974 (s), 1696 (l), 1513 (s), 1419 (m), 1365 (m), 1332 (s), 1301 (s), 1237 (m), 1169 (m), 1110 (m), 1014 (s), 984 (s), 865 (s), 813 (m), 752 (s), 678 (s).

HRMS (ESI): *m*/*z* calc. for $C_{17}H_{23}O_2NNa$ $[M+Na]^+$: 296.1621, found: 296.1624.

[α]^{25}$_{589}$ = +122.5° (*c* 1.00, $CHCl_3$).

(+)-(*R*)-*N*-*tert*-Butoxycarbonyl-5-(4-methoxyphenyl)-3-piperidene (53)

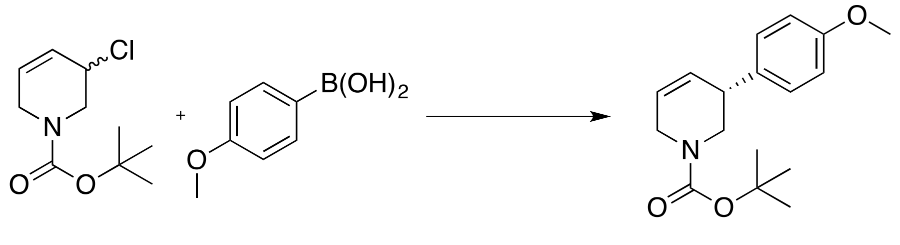

In a 10 mL round bottomed flask $[Rh(cod)(OH)]_2$ (4.6 mg, 0.01 mmol, 0.025 eq), (*R*)-(+)-5,5′-dichloro-2,2′-bis(diphenylphosphino)-6,6′-dimethoxy-1,1′-biphenyl (15.6 mg, 0.024 mmol, 0.06 eq) and Cs_2CO_3 (130.3 mg, 0.40 mmol, 1.00 eq) were stirred in THF (2 mL) at 60 °C for 30 min. A solution of *N*-*tert*-butoxycarbonyl-5-chloro-3-piperidene (86.8 mg, 0.40 mmol, 1.00 eq) in THF (0.75 mL) and a solution of 4-methoxybenzeneboronic acid (121.6 mg, 0.80 mmol, 2.00 eq) in THF (0.75 mL) were sequentially added *via* syringe and both flasks were rinsed with THF (0.25 mL each). The resulting mixture was then stirred for 16 h at 80 °C in a sealed flask. SiO_2 (20 mg) was added and the solvent was then carefully evaporated. The resulting solid was directly loaded onto a chromatographic column and eluted with pentane:Et_2O (9:1) to afford (+)-(*R*)-*N*-*tert*-butoxycarbonyl-5-(4-methoxyphenyl)-3-piperidene in 64% yield (75 mg, 0.26 mmol).

Enantiomeric excess of 94% was determined by HPLC [Chiralpak® IB; flow: 1.0 mL/min; hexane/*i*-PrOH: 99: 1; λ = 210 nm; major enantiomer t_R = 6.6 min; minor enantiomer t_R = 7.9 min].

^{1}**H NMR** (400 MHz, Chloroform-*d*) δ 1.38 (m, 9H), 2.96, 3.34, 3.70 - 3.85 and 3.98 (rotameric m, 2H), 3.40 - 3.55 (m, 1H), 3.78 (s, 3H), 3.70 - 3.85 and 3.98 (rotameric, 2H), 5.86 (br s, 2H), 6.82 – 6.88 (m, 2H), 7.08 – 7.16 (m, 2H).

^{13}**C NMR** (101 MHz, Chloroform-*d*) δ 28.4 (3C), 40.8, 43.0 and 43.7 (rotameric, 1C), 47.5 and 48.8 (rotameric, 1C), 55.4, 79.6, 114.0 (2C), 124.8 and 125.7 (rotameric, 1C), 128.7 and 129.6 (rotameric, 1C), 128.9 (2C), 134.4, 154.8, 158.5.

IR (ATR) ν_{max} /cm^{-1} = 2835 (s), 1694 (l), 1612 (s), 1512 (m), 1419 (m), 1365 (m), 1331 (s), 1300 (m), 1240 (l), 1171 (l), 1113 (m), 1036 (m), 984 (s), 869 (s), 829 (m), 768 (s), 678 (s).

HRMS (ESI): *m/z* calc. for $C_{17}H_{23}O_3NNa$ $[M+Na]^+$: 312.1570, found: 312.1571.

$[\alpha]^{25}_{589}$ = +118.9° (*c* 1.00, $CHCl_3$).

(+)-(*R*)-*N*-*tert*-Butoxycarbonyl-5-(3-methylphenyl)-3-piperidene (54)

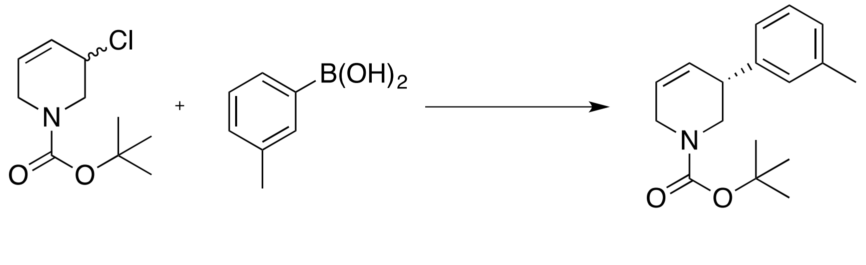

In a 10 mL round bottomed flask $[Rh(cod)(OH)]_2$ (4.6 mg, 0.01 mmol, 0.025 eq), (*R*)-(+)-5,5′-dichloro-2,2′-bis(diphenylphosphino)-6,6′-dimethoxy-1,1′-biphenyl (15.6 mg, 0.024 mmol, 0.06 eq) and Cs_2CO_3 (130.3 mg, 0.40 mmol, 1.00 eq) were stirred in THF (2 mL) at 60 °C for 30 min. A solution of *N*-*tert*-butoxycarbonyl-5-chloro-3-piperidene (86.8 mg, 0.40 mmol, 1.00 eq) in THF (0.75 mL) and a solution of 3-methylbenzeneboronic acid (108.8 mg, 0.80 mmol, 2.00 eq) in THF (0.75 mL) were sequentially added *via* syringe and both flasks were rinsed with THF (0.25 mL each). The resulting mixture was then stirred for 16 h at 80 °C in a sealed flask. SiO_2 (20 mg) was added and the solvent was then carefully evaporated. The resulting solid was directly loaded onto a chromatographic column and eluted with pentane:Et_2O (9:1) to afford (+)-(*R*)-*N*-*tert*-butoxycarbonyl-5-(3-methylphenyl)-3-piperidene in 66% yield (72 mg, 0.26 mmol).

Enantiomeric excess of 97% was determined by HPLC [Chiralpak® IB; flow: 1.0 mL/min; hexane/*i*-PrOH: 99: 1; λ = 210 nm; major enantiomer t_R = 4.9 min; minor enantiomer t_R = 5.6 min].

^{1}H NMR (400 MHz, Chloroform-*d*) δ 1.24 – 1.56 (m, 9H), 2.34 (s, 3H), 2.98, 3.39, 3.70 – 3.91 and 3.93 – 4.19 (rotameric m, 2H), 3.43 – 3.59 (m, 1H), 3.70 – 3.91 and 3.93 – 4.19 (rotameric m, 2H), 5.89 (br s, 2H), 6.99 – 7.08 (m, 3H), 7.20 (t, *J* = 7.7 Hz, 1H).

^{13}C NMR (101 MHz, Chloroform-*d*) δ 21.4, 28.3 (3C), 41.5, 43.0 and 43.6 (rotameric, 1C), 47.3 and 48.5 (rotameric, 1C), 79.4, 124.9, 125.7, 127.5, 128.4, 128.6, 129.4, 138.0, 142.2, 154.7.

IR (ATR) ν_{max} /cm^{-1} = 2974 (s), 2925 (s), 1696 (l), 1608 (s), 1420 (m), 1365 (m), 1295 (s), 1238 (m), 1173 (m), 1112 (m), 984 (s), 879 (s), 784 (s), 705 (s).

HRMS (ESI): *m/z* calc. for $C_{17}H_{23}O_2NNa$ $[M+Na]^+$: 296.1621, found: 296.1623.

$[\alpha]^{25}_{589}$ = +108.5° (*c* 1.00, $CHCl_3$).

(+)-(*R*)-*N*-*tert*-Butoxycarbonyl-5-(3-methoxyphenyl)-3-piperidene (55)

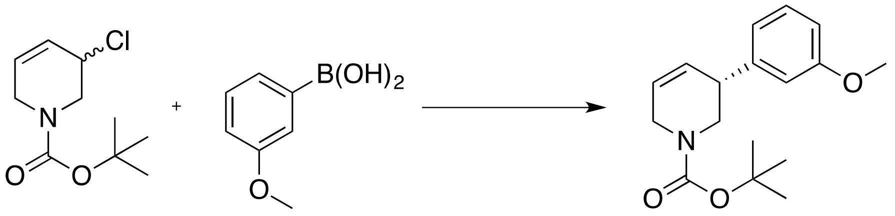

In a 10 mL round bottomed flask $[Rh(cod)(OH)]_2$ (4.6 mg, 0.01 mmol, 0.025 eq), (*R*)-(+)-5,5′-dichloro-2,2′-bis(diphenylphosphino)-6,6′-dimethoxy-1,1′-biphenyl (15.6 mg, 0.024 mmol, 0.06 eq) and Cs_2CO_3 (130.3 mg, 0.40 mmol, 1.00 eq) were stirred in THF (2 mL) at 60 °C for 30 min. A solution of *N*-*tert*-butoxycarbonyl-5-chloro-3-piperidene (86.8 mg, 0.40 mmol, 1.00 eq) in THF (0.75 mL) and a solution of 3-methoxybenzeneboronic acid (121.5 mg, 0.80 mmol, 2.00 eq) in THF (0.75 mL) were sequentially added *via* syringe and both flasks were rinsed with THF (0.25 mL each). The resulting mixture was then stirred for 16 h at 80 °C in a sealed flask. SiO_2 (20 mg) was added and the solvent was then carefully evaporated. The resulting solid was directly loaded onto a chromatographic column and eluted with pentane:Et_2O (8:2) to afford (+)-(*R*)-*N*-*tert*-butoxycarbonyl-5-(3-methoxyphenyl)-3-piperidene in 79% yield (92 mg, 0.32 mmol).

Enantiomeric excess of 96% was determined by HPLC [Chiralpak® IC; flow: 1.0 mL/min; hexane/*i*-PrOH: 95: 5; λ = 210 nm; major enantiomer t_R = 10.8 min; minor enantiomer t_R = 12.6 min].

^{1}H NMR (400 MHz, Chloroform-*d*) δ 1.23 – 1.55 (m, 9H), 3.01, 3.40, 3.67 – 3.89 and 4.05 (rotameric m, 2H), 3.42 – 3.58 (m, 1H), 3.78 (s, 3H), 3.67 – 3.89 and 4.05 (rotameric m, 2H), 5.88 (br s, 2H), 6.73 – 6.82 (m, 3H), 7.22 (t, *J* = 7.9 Hz, 1H).

^{13}C NMR (101 MHz, Chloroform-*d*) δ 28.3 (3C), 41.5, 42.9 and 43.6 (rotameric, 1C), 47.2 and 48.4 (rotameric, 1C), 55.2, 79.5, 112.1, 113.6, 120.3, 125.9, 128.1, 129.4, 143.9, 154.7, 159.7.

IR (ATR) ν_{max} /cm^{-1} = 2975 (s), 1694 (l), 1600 (s), 1487 (s), 1454 (m), 1420 (m), 1365 (m), 1263 (m), 1238 (m), 1159 (m), 1112 (m), 1050 (s), 1018 (s), 960 (s), 866 (s), 779 (s), 750 (s), 702 (s).

HRMS (ESI): *m/z* calc. for $C_{17}H_{23}O_3NNa$ $[M+Na]^+$: 312.1570, found: 312.1572.

$[\alpha]^{25}_{589}$ = +121.4° (*c* 1.00, $CHCl_3$).

(+)-(*R*)-*N*-*tert*-Butoxycarbonyl-5-(2-methylphenyl)-3-piperidene (56)

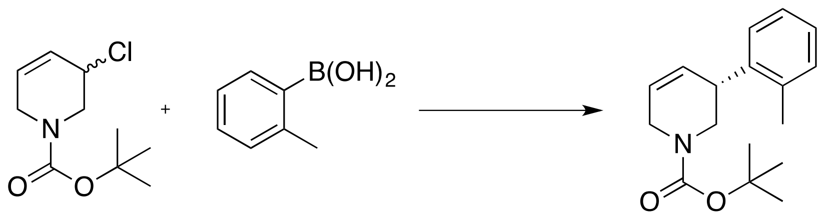

In a 10 mL round bottomed flask $[Rh(cod)(OH)]_2$ (4.6 mg, 0.01 mmol, 0.025 eq), (*R*)-(+)-5,5′-dichloro-2,2′-bis(diphenylphosphino)-6,6′-dimethoxy-1,1′-biphenyl (15.6 mg, 0.024 mmol, 0.06 eq) and Cs_2CO_3 (130.3 mg, 0.40 mmol, 1.00 eq) were stirred in THF (2 mL) at 60 °C for 30 min. A solution of *N*-*tert*-butoxycarbonyl-5-chloro-3-piperidene (86.8 mg, 0.40 mmol, 1.00 eq) in THF (0.75 mL) and a solution of 2-methylbenzeneboronic acid (108.8 mg, 0.80 mmol, 2.00 eq) in THF (0.75 mL) were sequentially added *via* syringe and both flasks were rinsed with THF (0.25 mL each). The resulting mixture was then stirred for 16 h at 80 °C in a sealed flask. SiO_2 (20 mg) was added and the solvent was then carefully evaporated. The resulting solid was directly loaded onto a chromatographic column and eluted with pentane:Et_2O (9:1) to afford (+)-(*R*)-*N*-*tert*-butoxycarbonyl-5-(2-methylphenyl)-3-piperidene in 29% yield (32 mg, 0.12 mmol).

Enantiomeric excess of 93% was determined by HPLC [Chiralpak® IC; flow: 1.0 mL/min; hexane/*i*-PrOH: 99: 1; λ = 210 nm; major enantiomer t_R = 11.1 min; minor enantiomer t_R = 12.2 min].

^{1}H NMR (400 MHz, Chloroform-*d*) δ 1.38 (m, 9H), 2.40 (s, 3H), 3.15 – 3.33 and 3.67 – 3.91 (rotameric m, 2H), 3.67 – 3.91 (m, 1H), 3.67 – 3.91 and 3.91 – 4.21 (rotameric m, 2H), 5.84 – 5.99 (m, 2H), 7.14 (m, 4H).

^{13}C NMR (101 MHz, Chloroform-*d*) δ 19.1, 28.3 (m, 3C), 37.7, 43.1, 47.2, 79.4, 126.1, 126.2, 126.6, 127.4, 128.8, 130.3, 135.7, 139.8, 154.6.

IR (ATR) ν_{max} /cm^{-1} = 2974 (s), 2929 (s), 1697 (l), 1478 (s), 1458 (s), 1420 (m), 1390 (s), 1365 (m), 1330 (s), 1294 (s), 1239 (m), 1168 (m), 1119 (s), 1013 (s), 984 (s), 865 (s), 755 (s), 728 (s), 689 (s), 661 (s).

HRMS (ESI): *m/z* calc. for $C_{17}H_{23}O_2NNa$ $[M+Na]^+$: 296.1621, found: 296.1625.

$[\alpha]^{25}_{589}$ = +46.1° (*c* 1.00, $CHCl_3$).

(+)-(*R*)-*N-tert*-Butoxycarbonyl-5-(3-hydroxyphenyl)-3-piperidene (57)

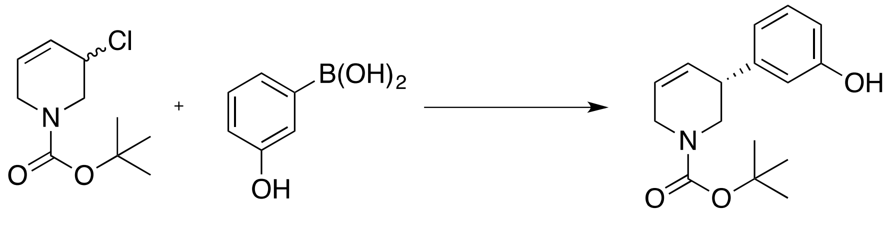

In a 10 mL round bottomed flask $[Rh(cod)(OH)]_2$ (4.6 mg, 0.01 mmol, 0.025 eq), (*R*)-(+)-5,5′-dichloro-2,2′-bis(diphenylphosphino)-6,6′-dimethoxy-1,1′-biphenyl (15.6 mg, 0.024 mmol, 0.06 eq) and Cs_2CO_3 (130.3 mg, 0.40 mmol, 1.00 eq) were stirred in THF (2 mL) at 60 °C for 30 min. A solution of *N-tert*-butoxycarbonyl-5-chloro-3-piperidene (86.8 mg, 0.40 mmol, 1.00 eq) in THF (0.75 mL) and a solution of 3-hydroxybenzeneboronic acid (110.3 mg, 0.80 mmol, 2.00 eq) in THF (0.75 mL) were sequentially added *via* syringe and both flasks were rinsed with THF (0.25 mL each). The resulting mixture was then stirred for 16 h at 80 °C in a sealed flask. SiO_2 (20 mg) was added and the solvent was then carefully evaporated. The resulting solid was directly loaded onto a chromatographic column and eluted with pentane:Et_2O (7:3) to afford (+)-(*R*)-*N-tert*-butoxycarbonyl-5-(3-hydroxyphenyl)-3-piperidene in 72% yield (79 mg, 0.29 mmol).

Enantiomeric excess of 94% was determined by HPLC [Chiralpak® IC; flow: 1.0 mL/min; hexane/*i*-PrOH: 95: 5; λ = 210 nm; major enantiomer t_R = 14.2 min; minor enantiomer t_R = 20.3 min].

^{1}H NMR (400 MHz, Chloroform-*d*) δ 1.38 (m, 9H), 3.01, 3.45, 3.67 and 3.88 – 4.20 (rotameric m, 2H), 3.45 (s, 1H), 3.80 and 3.88 – 4.20 (rotameric m, 2H), 5.87 (br s, 2H), 6.65 – 6.80 (m, 3H), 7.01 (s, 1H), 7.15 (m, 1H).

^{13}C NMR (101 MHz, Chloroform-*d*) δ 28.3 (3C), 41.4, 43.0 and 43.7 (rotameric, 1C), 47.3 and 48.4 (rotameric, 1C), 80.0, 113.9, 114.9, 119.8, 125.6, 128.2, 129.6, 143.7, 155.2, 156.5.

IR (ATR) ν_{max} /cm^{-1} = 3333 (s), 2976 (s), 2929 (s), 1668 (l), 1648 (l), 1599 (m), 1588 (m), 1478 (m), 1455 (l), 1432 (l), 1391 (s), 1366 (m), 1301 (m), 1243 (l), 1157 (l), 1119 (m), 1021 (s), 953 (s), 862 (s), 813 (s), 784 (s), 754 (s), 702 (s).

HRMS (ESI): *m/z* calc. for $C_{16}H_{21}O_3NNa$ $[M+Na]^+$: 298.1414, found: 298.1414.

$[\alpha]^{25}_{589}$ = +126.2° (*c* 1.00, $CHCl_3$).

(+)-(*R*)-*N-tert*-Butoxycarbonyl-5-(4-trifluoromethylphenyl)-3-piperidene (58)

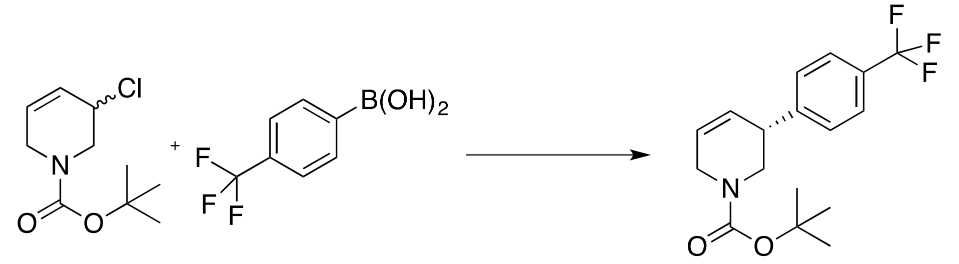

In a 10 mL round bottomed flask $[Rh(cod)(OH)]_2$ (4.6 mg, 0.01 mmol, 0.025 eq), (*R*)-(+)-5,5′-dichloro-2,2′-bis(diphenylphosphino)-6,6′-dimethoxy-1,1′-biphenyl (15.6 mg, 0.024 mmol, 0.06 eq) and Cs_2CO_3 (130.3 mg, 0.40 mmol, 1.00 eq) were stirred in THF (2 mL) at 60 °C for 30 min. A solution of *N-tert*-butoxycarbonyl-5-chloro-3-piperidene (86.8 mg, 0.40 mmol, 1.00 eq) in THF (0.75 mL) and a solution of 4-trifluoromethylbenzeneboronic acid (151.6 mg, 0.80 mmol, 2.00 eq) in THF (0.75 mL) were sequentially added *via* syringe and both flasks were rinsed with THF (0.25 mL each). The resulting mixture was then stirred for 16 h at 80 °C in a sealed flask. SiO_2 (20 mg) was added and the solvent was then carefully evaporated. The resulting solid was directly loaded onto a chromatographic column and eluted with pentane:Et_2O (9:1) to afford (+)-(*R*)-*N-tert*-butoxycarbonyl-5-(4-trifluoromethylphenyl)-3-piperidene in 65% yield (85 mg, 0.26 mmol).

Enantiomeric excess of 94% was determined by HPLC [Chiralpak® IC; flow: 1.0 mL/min; hexane/*i*-PrOH: 99: 1; λ = 210 nm; minor enantiomer t_R = 7.5 min; major enantiomer t_R = 8.5 min].

^{1}H NMR (400 MHz, Chloroform-*d*) δ 1.12 – 1.57 (m, 9H), 3.13, 3.61 and 3.79 – 4.25 (rotameric m, 2H), 3.61 (s, 1H), 3.79 – 4.25 (rotameric m, 2H), 5.82 – 6.02 (m, 2H), 7.32 (d, *J* = 8.0 Hz, 2H), 7.56 (d, *J* = 8.0 Hz, 2H).

^{19}F NMR (376 MHz, Chloroform-*d*) δ -62.44.

^{13}C NMR (101 MHz, Chloroform-*d*) δ 28.2 (3C), 41.4, 43.0 and 43.7 (rotameric, 1C), 46.9 and 48.2 (rotameric, 1C), 79.7, 124.2 (d, *J* = 271.9 Hz), 125.4 (2C), 126.9, 128.3 (3C), 129.1 (d, *J* = 32.4 Hz), 146.3, 154.6.

IR (ATR) ν_{max} /cm^{-1} = 2980 (s), 1673 (m), 1617 (m), 1420 (m), 1365 (s), 1327 (l), 1238 (m), 1163 (l), 1123 (l), 1068 (m), 982 (s), 840 (s), 755 (s), 646 (s), 620 (s).

HRMS (ESI): *m/z* calc. for $C_{17}H_{20}O_2NF_3Na$ $[M+Na]^+$: 350.1338, found: 350.1338.

$[\alpha]^{25}_{589}$ = +111.0° (*c* 1.00, $CHCl_3$).

(+)-(*R*)-*N-tert*-Butoxycarbonyl-5-(3-methoxycarbonylphenyl)-3-piperidene (59)

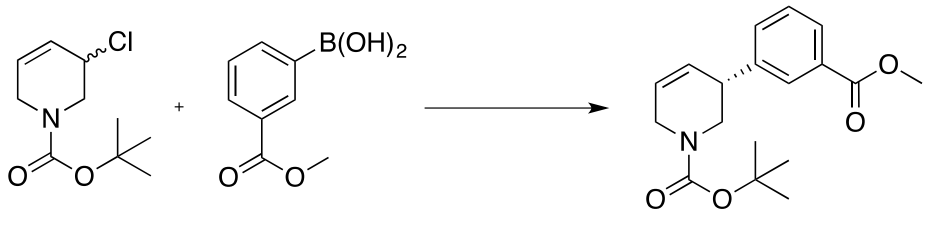

In a 10 mL round bottomed flask $[Rh(cod)(OH)]_2$ (4.6 mg, 0.01 mmol, 0.025 eq), (*R*)-(+)-5,5′-dichloro-2,2′-bis(diphenylphosphino)-6,6′-dimethoxy-1,1′-biphenyl (15.6 mg, 0.024 mmol, 0.06 eq) and Cs_2CO_3 (130.3 mg, 0.40 mmol, 1.00 eq) were stirred in THF (2 mL) at 60 °C for 30 min. A solution of *N-tert*-butoxycarbonyl-5-chloro-3-piperidene (86.8 mg, 0.40 mmol, 1.00 eq) in THF (0.75 mL) and a solution of 3-methoxycarbonylbenzeneboronic acid (144.0 mg, 0.80 mmol, 2.00 eq) in THF (0.75 mL) were sequentially added *via* syringe and both flasks were rinsed with THF (0.25 mL each). The resulting mixture was then stirred for 16 h at 80 °C in a sealed flask. SiO_2 (20 mg) was added and the solvent was then carefully evaporated. The resulting solid was directly loaded onto a chromatographic column and eluted with pentane:Et_2O (9:1) to afford (+)-(*R*)-*N-tert*-butoxycarbonyl-5-(3-methoxycarbonylphenyl)-3-piperidene in 68% yield (86 mg, 0.27 mmol).

Enantiomeric excess of 95% was determined by HPLC [Chiralpak® IC; flow: 1.0 mL/min; hexane/*i*-PrOH: 90: 10; λ = 210 nm; major enantiomer t_R = 13.0 min; minor enantiomer t_R = 23.6 min].

^{1}H NMR (400 MHz, Chloroform-*d*) δ 1.15 – 1.58 (m, 9H), 3.08, 3.36 – 3.49, 3.71 and 3.93 – 4.17 (rotameric m, 2H), 3.56 (s, 1H), 3.89 (s, 3H), 3.93 – 4.17 (rotameric m, 2H), 5.90 (m, 2H), 7.33 – 7.42 (m, 2H), 7.85 – 7.94 (m, 2H).

^{13}C NMR (101 MHz, Chloroform-*d*) δ 28.3 (3C), 41.3, 42.9 and 43.6 (rotameric, 1C), 47.1 and 48.3 (rotameric, 1C), 52.1, 79.6, 125.6 and 126.5 (rotameric, 1C), 127.5, 128.0, 128.5, 129.0, 130.4, 132.5, 142.6, 154.6, 167.0.

IR (ATR) ν_{max} /cm^{-1} = 2975 (s), 1723 (l), 1694 (l), 1420 (m), 1365 (m), 1286 (m), 1238 (m), 1165 (m), 1111 (m), 982 (s), 866 (s), 818 (s), 755 (m), 699 (s).

HRMS (ESI): *m/z* calc. for $C_{18}H_{23}O_4NNa$ $[M+Na]^+$: 340.1519, found: 340.1519.

$[\alpha]^{25}_{589}$ = +102.9° (*c* 1.00, $CHCl_3$).

(+)-(*R*)-*N-tert*-Butoxycarbonyl-5-(3-nitrophenyl)-3-piperidene (60)

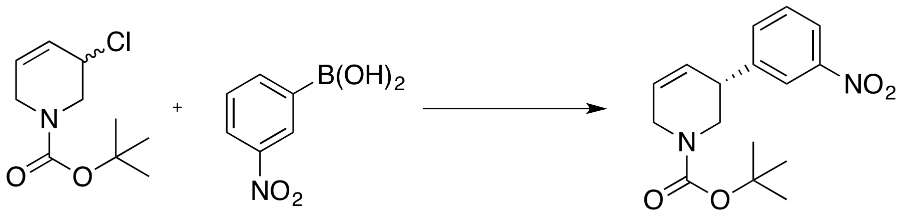

In a 10 mL round bottomed flask $[Rh(cod)(OH)]_2$ (4.6 mg, 0.01 mmol, 0.025 eq), (*R*)-(+)-5,5′-dichloro-2,2′-bis(diphenylphosphino)-6,6′-dimethoxy-1,1′-biphenyl (15.6 mg, 0.024 mmol, 0.06 eq) and Cs_2CO_3 (130.3 mg, 0.40 mmol, 1.00 eq) were stirred in THF (2 mL) at 60 °C for 30 min. A solution of *N-tert*-butoxycarbonyl-5-chloro-3-piperidene (86.8 mg, 0.40 mmol, 1.00 eq) in THF (0.75 mL) and a solution of 3-nitrobenzeneboronic acid (133.0 mg, 0.80 mmol, 2.00 eq) in THF (0.75 mL) were sequentially added *via* syringe and both flasks were rinsed with THF (0.25 mL each). The resulting mixture was then stirred for 16 h at 80 °C in a sealed flask. SiO_2 (20 mg) was added and the solvent was then carefully evaporated. The resulting solid was directly loaded onto a chromatographic column and eluted with

pentane:Et_2O (8:2) to afford (+)-(*R*)-*N-tert*-butoxycarbonyl-5-(3-nitrophenyl)-3-piperidene in 64% yield (78 mg, 0.26 mmol).

Enantiomeric excess of 94% was determined by HPLC [Chiralpak® IC; flow: 1.0 mL/min; hexane/*i*-PrOH: 95: 5; λ = 210 nm; major enantiomer t_R = 25.4 min; minor enantiomer t_R = 28.6 min].

^{1}H NMR (400 MHz, Chloroform-*d*) δ 1.33 (m, 9H), 3.31, 3.49 – 3.77 and 3.78 – 4.26 (rotameric m, 2H), 3.49 – 3.77 (m, 1H), 3.78 – 4.26 (rotameric m, 2H), 5.84 – 6.05 (m, 2H), 7.46 (t, *J* = 7.8 Hz, 1H), 7.55 (dt, *J* = 7.8, 1.5 Hz, 1H), 8.00 – 8.16 (m, 2H).

^{13}C NMR (101 MHz, Chloroform-*d*) δ 28.2 (3C), 41.1, 43.0 and 43.7 (rotameric, 1C), 46.7 and 48.0 (rotameric, 1C), 79.9, 121.9, 122.7, 126.5, 127.3, 129.3, 134.2, 144.4, 148.4, 154.6.

IR (ATR) ν_{max} /cm^{-1} = 2976 (s), 2930 (s), 1695 (l), 1530 (l), 1477 (s), 1421 (m), 1390 (s), 1365 (m), 1349 (m), 1298 (s), 1239 (m), 1164 (m), 1120 (s), 1019 (s), 863 (s), 808 (s), 769 (s), 738 (s), 690 (s), 669 (s).

HRMS (CI): *m/z* calc. for $C_{16}H_{21}O_4N_2$ $[M+H]^+$: 305.1496, found: 305.1500.

$[\alpha]^{25}_{589}$ = +117.5° (*c* 1.00, $CHCl_3$).

(+)-(*R*)-*N-tert*-Butoxycarbonyl-5-(4-fluorophenyl)-3-piperidene (61)

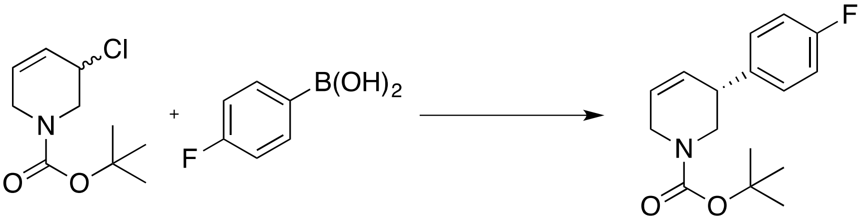

In a 10 mL round bottomed flask $[Rh(cod)(OH)]_2$ (4.6 mg, 0.01 mmol, 0.025 eq), (*R*)-(+)-5,5′-dichloro-2,2′-bis(diphenylphosphino)-6,6′-dimethoxy-1,1′-biphenyl (15.6 mg, 0.024 mmol, 0.06 eq) and Cs_2CO_3 (130.3 mg, 0.40 mmol, 1.00 eq) were stirred in THF (2 mL) at 60 °C for 30 min. A solution of *N-tert*-butoxycarbonyl-5-chloro-3-piperidene (86.8 mg, 0.40 mmol, 1.00 eq) in THF (0.75 mL) and a solution of 4-fluorobenzeneboronic acid (111.9 mg, 0.80 mmol, 2.00 eq) in THF (0.75 mL) were sequentially added *via* syringe and both flasks were rinsed with THF (0.25 mL each). The resulting mixture was then stirred for 16 h at 80 °C in a sealed flask. SiO_2 (20 mg) was added and the solvent was then carefully evaporated. The resulting solid was directly loaded onto a chromatographic column and eluted with pentane:Et_2O (9:1) to afford (+)-(*R*)-*N-tert*-butoxycarbonyl-5-(4-fluorophenyl)-3-piperidene in 51% yield (56 mg, 0.20 mmol).

Enantiomeric excess of 96% was determined by HPLC [Chiralpak® IC; flow: 1.0 mL/min; hexane/i-PrOH: 99: 1; λ = 210 nm; minor enantiomer t_R = 10.6 min; major enantiomer t_R = 11.4 min].

^{1}H NMR (400 MHz, Chloroform-*d*) δ 1.18 – 1.56 (m, 9H), 3.03, 3.36 – 3.58, 3.66, 3.76 – 4.17 (rotameric m, 2H), 3.36 – 3.58 (s, 1H), 3.76 – 4.17 (rotameric m, 2H), 5.82 – 5.96 (m, 2H), 6.95 – 7.02 (m, 2H), 7.12 – 7.19 (m, 2H).

^{19}F NMR (376 MHz, Chloroform-*d*) δ -116.45 (d, *J* = 74.9 Hz).

^{13}C NMR (101 MHz, Chloroform-*d*) δ 28.3 (3C), 40.8, 42.9 and 43.6 (rotameric, 1C), 47.3 and 48.5 (rotameric, 1C), 79.5, 115.2 (d, *J* = 21.3 Hz, 2C), 125.2 and 126.2 (rotameric, 1C), 127.9 and 129.0 (rotameric, 1C), 129.3 (2C), 137.9 (d, *J* = 3.2 Hz), 154.7, 161.8 (d, *J* = 244.7 Hz).

IR (ATR) ν_{max} /cm^{-1} = 2975 (s), 1695 (l), 1603 (s), 1509 (m), 1420 (m), 1366 (m), 1299 (s), 1236 (m), 1170 (m), 1114 (m), 1014 (s), 865 (s), 834 (m), 767 (s), 678 (s).

HRMS (ESI): *m/z* calc. for $C_{16}H_{20}O_2NFNa$ $[M+Na]^+$: 300.1370, found: 300.1371.

$[\alpha]^{25}_{589}$ = +92.2° (*c* 1.00, $CHCl_3$).

(+)-(*R*)-*N*-*tert*-Butoxycarbonyl-5-(4-chlorophenyl)-3-piperidene (62)

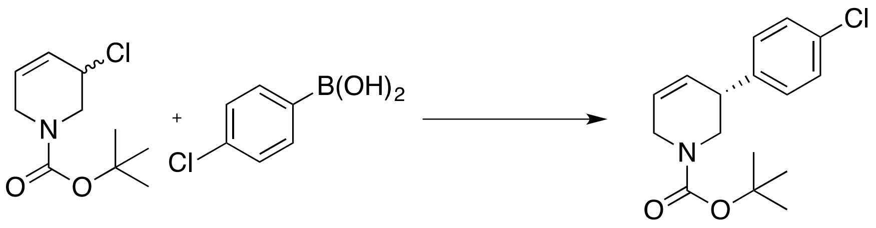

In a 10 mL round bottomed flask $[Rh(cod)(OH)]_2$ (4.6 mg, 0.01 mmol, 0.025 eq), (*R*)-(+)-5,5′-dichloro-2,2′-bis(diphenylphosphino)-6,6′-dimethoxy-1,1′-biphenyl (15.6 mg, 0.024 mmol, 0.06 eq) and Cs_2CO_3 (130.3 mg, 0.40 mmol, 1.00 eq) were stirred in THF (2 mL) at 60 °C for 30 min. A solution of *N*-*tert*-butoxycarbonyl-5-chloro-3-piperidene (86.8 mg, 0.40 mmol, 1.00 eq) in THF (0.75 mL) and a solution of 4-chlorobenzeneboronic acid (125.1 mg, 0.80 mmol, 2.00 eq) in THF (0.75 mL) were sequentially added *via* syringe and both flasks were rinsed with THF (0.25 mL each). The resulting mixture was then stirred for 16 h at 80 °C in a sealed flask. SiO_2 (20 mg) was added and the solvent was then carefully evaporated. The resulting solid was directly loaded onto a chromatographic column and eluted with pentane:Et_2O (9:1) to afford (+)-(*R*)-*N*-*tert*-butoxycarbonyl-5-(4-chlorophenyl)-3-piperidene in 63% yield (74 mg, 0.25 mmol).

Enantiomeric excess of 95% was determined by HPLC [Chiralpak® IC; flow: 1.0 mL/min; hexane/*i*-PrOH: 99: 1; λ = 210 nm; minor enantiomer t_R = 10.5 min; major enantiomer t_R = 11.4 min].

^{1}H NMR (400 MHz, Chloroform-*d*) δ 1.22 – 1.51 (m, 9H), 3.05, 3.35 – 3.56, 3.65 and 3.76 – 4.18 (rotameric m, 2H), 3.35 – 3.56 (s, 1H), 3.76 – 4.18 (rotameric m, 2H), 5.80 – 5.97 (m, 2H), 7.10 – 7.15 (m, 2H), 7.24 – 7.28 (m, 2H).

^{13}C NMR (101 MHz, Chloroform-*d*) δ 28.3 (3C), 40.9, 42.9 and 43.6 (rotameric, 1C), 47.0 and 48.3 (rotameric, 1C), 79.7, 125.4 and 126.4 (rotameric, 1C), 127.6, 128.5 (2C), 129.2 (2C), 132.5, 140.7, 154.6.

IR (ATR) ν_{max} /cm^{-1} = 2975 (s), 1695 (l), 1491 (s), 1420 (m), 1365 (m), 1333 (s), 1295 (s), 1237 (m), 1166 (m), 1116 (m), 1014 (s), 985 (s), 864 (s), 821 (s), 768 (s), 646 (s).

HRMS (ESI): *m/z* calc. for $C_{16}H_{20}O_2NClNa$ $[M+Na]^+$: 316.1075, found: 316.1074.

[α]^{25}$_{589}$ = +119.2° (*c* 1.00, $CHCl_3$).

(+)-(*R*)-*N*-*tert*-Butoxycarbonyl-5-(3-chlorophenyl)-3-piperidene (63)

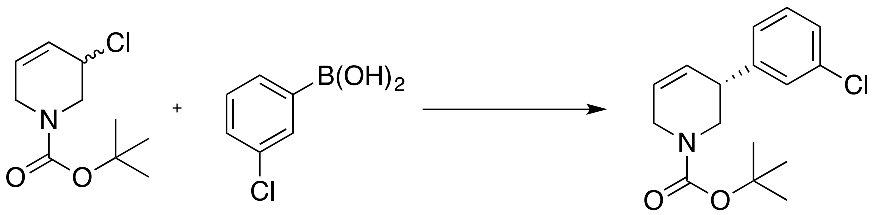

In a 10 mL round bottomed flask $[Rh(cod)(OH)]_2$ (4.6 mg, 0.01 mmol, 0.025 eq), (*R*)-(+)-5,5′-dichloro-2,2′-bis(diphenylphosphino)-6,6′-dimethoxy-1,1′-biphenyl (15.6 mg, 0.024 mmol, 0.06 eq) and Cs_2CO_3 (130.3 mg, 0.40 mmol, 1.00 eq) were stirred in THF (2 mL) at 60 °C for 30 min. A solution of *N*-*tert*-butoxycarbonyl-5-chloro-3-piperidene (86.8 mg, 0.40 mmol, 1.00 eq) in THF (0.75 mL) and a solution of 3-chlorobenzeneboronic acid (125.1 mg, 0.80 mmol, 2.00 eq) in THF (0.75 mL) were sequentially added *via* syringe and both flasks were rinsed with THF (0.25 mL each). The resulting mixture was then stirred for 16 h at 80 °C in a sealed flask. SiO_2 (20 mg) was added and the solvent was then carefully evaporated. The resulting solid was directly loaded onto a chromatographic column and eluted with pentane:Et_2O (9:1) to afford (+)-(*R*)-*N*-*tert*-butoxycarbonyl-5-(3-chlorophenyl)-3-piperidene in 66% yield (78 mg, 0.26 mmol).

Enantiomeric excess of 95% was determined by HPLC [Chiralpak® IB; flow: 1.0 mL/min; hexane/*i*-PrOH: 99: 1; λ = 210 nm; major enantiomer t_R = 5.5 min; minor enantiomer t_R = 5.9 min].

^{1}H NMR (400 MHz, Chloroform-*d*) δ 1.19 – 1.55 (m, 9H), 3.10, 3.42 – 3.58, 3.67 and 3.79 – 4.19 (rotameric m, 2H), 3.42 – 3.58 (s, 1H), 3.79 – 4.19 (rotameric m, 2H), 5.81 – 6.00 (m, 2H), 7.09 (m, 1H), 7.17 – 7.25 (m, 3H).

^{13}C NMR (101 MHz, Chloroform-*d*) δ 28.3 (3C), 41.2, 43.0 and 43.6 (rotameric, 1C), 47.0 and 48.2 (rotameric, 1C), 79.7, 126.2, 126.6, 126.9, 127.3, 128.0, 129.7, 134.3, 144.3, 154.6.

IR (ATR) ν_{max} /cm^{-1} = 2975 (s), 1694 (l), 1596 (s), 1572 (s), 1477 (s), 1420 (m), 1365 (m), 1233 (s), 1237 (m), 1166 (m), 1115 (m), 865 (s), 785 (s), 734 (s), 697 (s).

HRMS (ESI): *m*/*z* calc. for $C_{16}H_{20}O_2NClNa$ $[M+Na]^+$: 316.1075, found: 316.1074.

[α]^{25}$_{589}$ = +121.6° (*c* 1.00, $CHCl_3$).

(+)-(*R*)-*N*-*tert*-Butoxycarbonyl-5-(3-bromophenyl)-3-piperidene (64)

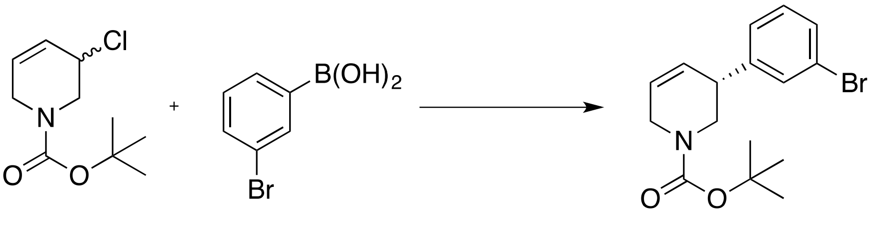

In a 10 mL round bottomed flask $[Rh(cod)(OH)]_2$ (4.6 mg, 0.01 mmol, 0.025 eq), (*R*)-(+)-5,5′-dichloro-2,2′-bis(diphenylphosphino)-6,6′-dimethoxy-1,1′-biphenyl (15.6 mg, 0.024 mmol,

0.06 eq) and Cs_2CO_3 (130.3 mg, 0.40 mmol, 1.00 eq) were stirred in THF (2 mL) at 60 °C for 30 min. A solution of *N-tert*-butoxycarbonyl-5-chloro-3-piperidene (86.8 mg, 0.40 mmol, 1.00 eq) in THF (0.75 mL) and a solution of 3-bromobenzeneboronic acid (160.6 mg, 0.80 mmol, 2.00 eq) in THF (0.75 mL) were sequentially added *via* syringe and both flasks were rinsed with THF (0.25 mL each). The resulting mixture was then stirred for 16 h at 80 °C in a sealed flask. SiO_2 (20 mg) was added and the solvent was then carefully evaporated. The resulting solid was directly loaded onto a chromatographic column and eluted with pentane:Et_2O (9:1) to afford (+)-(*R*)-*N-tert*-butoxycarbonyl-5-(3-bromophenyl)-3-piperidene in 54% yield (73 mg, 0.21 mmol).

Enantiomeric excess of 96% was determined by HPLC [Chiralpak® IB; flow: 1.0 mL/min; hexane/*i*-PrOH: 99: 1; λ = 210 nm; major enantiomer t_R = 5.8 min; minor enantiomer t_R = 6.4 min].

^{1}H NMR (400 MHz, Chloroform-*d*) δ 1.21 – 1.56 (m, 9H), 3.09, 3.40 – 3.57, 3.66 and 3.79 – 4.18 (rotameric m, 2H), 3.40 – 3.57 (s, 1H), 3.79 – 4.18 (rotameric m, 2H), 5.81 – 5.98 (m, 2H), 7.10 – 7.20 (m, 2H), 7.33 – 7.38 (m, 2H).

^{13}C NMR (101 MHz, Chloroform-*d*) δ 28.3 (3C), 41.2, 42.9 and 43.6 (rotameric, 1C), 47.0 and 48.2 (rotameric, 1C), 79.7, 122.6, 125.6, 126.6, 127.2, 129.8, 130.0, 130.9, 144.6, 154.6.

IR (ATR) ν_{max} /cm^{-1} = 2975 (s), 1695 (l), 1593 (s), 1567 (s), 1475 (m), 1421 (m), 1365 (m), 1332 (s), 1238 (m), 1165 (s), 1116 (m), 1074 (s), 1014 (s), 864 (s), 783 (s), 697 (s), 665 (s).

HRMS (ESI): *m/z* calc. for $C_{16}H_{20}O_2NBrNa$ $[M+Na]^+$: 360.0570, found: 360.0570.

$[\alpha]^{25}_{589}$ = +87.0° (*c* 1.00, $CHCl_3$).

(+)-(*R*)-*N-tert*-Butoxycarbonyl-5-(3-furanyl)-3-piperidene (65)

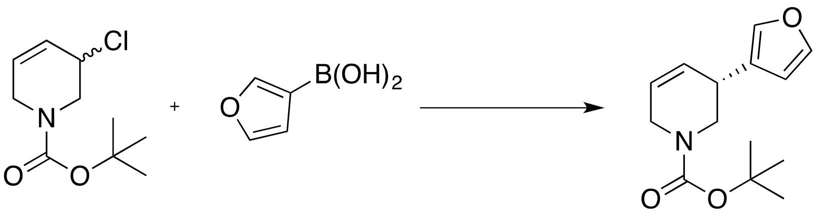

In a 10 mL round bottomed flask $[Rh(cod)(OH)]_2$ (4.6 mg, 0.01 mmol, 0.025 eq), (*R*)-(+)-5,5′-dichloro-2,2′-bis(diphenylphosphino)-6,6′-dimethoxy-1,1′-biphenyl (15.6 mg, 0.024 mmol, 0.06 eq) and Cs_2CO_3 (130.3 mg, 0.40 mmol, 1.00 eq) were stirred in THF (2 mL) at 60 °C for 30 min. A solution of *N-tert*-butoxycarbonyl-5-chloro-3-piperidene (86.8 mg, 0.40 mmol, 1.00 eq) in THF (0.75 mL) and a solution of 3-furanylboronic acid (89.5 mg, 0.80 mmol, 2.00 eq) in THF (0.75 mL) were sequentially added *via* syringe and both flasks were rinsed with THF (0.25 mL each). The resulting mixture was then stirred for 16 h at 80 °C in a sealed flask. SiO_2 (20 mg) was added and the solvent was then carefully evaporated. The resulting solid was directly loaded onto a chromatographic column and eluted with pentane:Et_2O (9:1) to afford (+)-(*R*)-*N-tert*-butoxycarbonyl-5-(3-furanyl)-3-piperidene in 53% yield (53 mg, 0.21 mmol) as a colorless oil.

Enantiomeric excess of 99% was determined by HPLC [Chiralpak® IC; flow: 1.0 mL/min; hexane/*i*-PrOH: 99: 1; λ = 210 nm; minor enantiomer t_R = 12.3 min; major enantiomer t_R = 14.1 min].

^{1}H NMR (400 MHz, Chloroform-*d*) δ 1.36 (s, 9H), 3.15, 3.29 – 3.55, 3.61 and 3.75 – 4.14 (rotameric m, 2H), 3.29 – 3.55 (m, 1H), 3.75 – 4.14 (rotameric m, 2H), 5.70 – 5.90 (m, 2H), 6.29 (m, 1H), 7.19 – 7.25 (m, 1H), 7.36 (m, 1H).

^{13}C NMR (101 MHz, Chloroform-*d*) δ 28.0 – 28.5 (3C), 32.4, 42.8 and 43.6 (rotameric, 1C), 45.8 and 47.0 (rotameric, 1C), 79.6, 110.0, 124.6 and 125.4 (rotameric, 1C), 125.9, 127.8 and 128.6 (rotameric, 1C), 139.4, 143.0, 154.8.

IR (ATR) ν_{max} /cm^{-1} = 2976 (s), 1694 (l), 1478 (s), 1420 (m), 1391 (s), 1366 (m), 1334 (s), 1302 (s), 1238 (m), 1171 (m), 1115 (s), 1029 (s), 967 (s), 873 (s), 786 (s), 743 (s), 641 (s).

HRMS (CI): *m/z* calc. for $C_{14}H_{20}O_3N$ $[M+H]^+$: 250.1438, found: 250.1442.

$[\alpha]^{25}_{589}$ = +105.9° (*c* 1.00, $CHCl_3$).

(+)-(*R*)-*N*-*tert*-Butoxycarbonyl-5-(2-benzofuranyl)-3-piperidene (66)

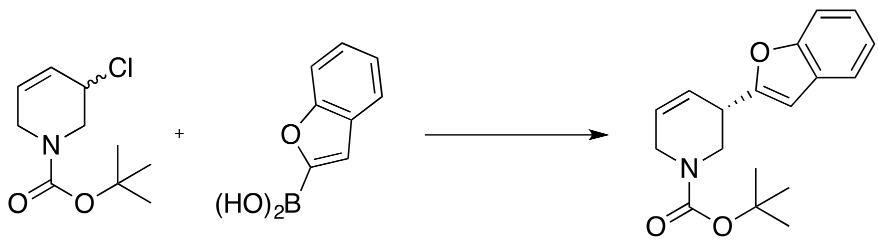

In a 10 mL round bottomed flask $[Rh(cod)(OH)]_2$ (4.6 mg, 0.01 mmol, 0.025 eq), (*R*)-(+)-5,5′-dichloro-2,2′-bis(diphenylphosphino)-6,6′-dimethoxy-1,1′-biphenyl (15.6 mg, 0.024 mmol, 0.06 eq) and Cs_2CO_3 (130.3 mg, 0.40 mmol, 1.00 eq) were stirred in THF (2 mL) at 60 °C for 30 min. A solution of *N*-*tert*-butoxycarbonyl-5-chloro-3-piperidene (86.8 mg, 0.40 mmol, 1.00 eq) in THF (0.75 mL) and a solution of 2-benzofuranylboronic acid (129.5 mg, 0.80 mmol, 2.00 eq) in THF (0.75 mL) were sequentially added *via* syringe and both flasks were rinsed with THF (0.25 mL each). The resulting mixture was then stirred for 16 h at 80 °C in a sealed flask. SiO_2 (20 mg) was added and the solvent was then carefully evaporated. The resulting solid was directly loaded onto a chromatographic column and eluted with pentane:Et_2O (9:1) to afford (+)-(*R*)-*N*-*tert*-butoxycarbonyl-5-(2-benzofuranyl)-3-piperidene in 79% yield (95 mg, 0.32 mmol).

Enantiomeric excess of 99% was determined by HPLC [Chiralpak® IC; flow: 1.0 mL/min; hexane/*i*-PrOH: 99:1; λ = 210 nm; minor enantiomer t_R = 12.9 min; major enantiomer t_R = 14.2 min].

^{1}H NMR (400 MHz, Chloroform-*d*) δ 1.31 (m, 9H), 3.52 – 3.71 and 3.89 – 4.13 (rotameric m, 2H), 3.52 – 3.71 (m, 1H), 3.72 – 3.89, 3.89 – 4.13 and 4.19 – 4.35 (rotameric m, 2H), 5.96 (m, 2H), 6.45 (br s, 1H), 7.18 (td, *J* = 7.3, 1.2 Hz, 1H), 7.23 (td, *J* = 7.7, 1.6 Hz, 1H), 7.43 (d, *J* = 8.0 Hz, 1H), 7.45 – 7.50 (m, 1H).

^{13}C NMR (101 MHz, Chloroform-*d*) δ 28.0 (3C), 35.6, 42.9 and 43.6 (rotameric, 1C), 44.8 and 45.0 (rotameric, 1C), 79.5, 103.7, 110.9, 120.6, 122.5, 123.6, 124.6, 127.5, 128.6, 154.6, 154.9, 158.1.

IR (ATR) v_{max} /cm^{-1} = 2976 (s), 1695 (l), 1585 (s), 1475 (s), 1455 (m), 1422 (m), 1391 (s), 1365 (m), 1298 (s), 1241 (m), 1169 (m), 1114 (s), 1017 (s), 929 (s), 862 (s), 803 (s), 750 (m), 647 (s).

HRMS (ESI): *m/z* calc. for $C_{18}H_{21}O_3NNa$ $[M+Na]^+$: 322.1414, found: 322.1413.

$[\alpha]^{25}{}_{589}$ = +170.6° (*c* 1.00, $CHCl_3$).

(+)-(*R*)-*N-tert*-Butoxycarbonyl-5-(3-thienyl)-3-piperidene (67)

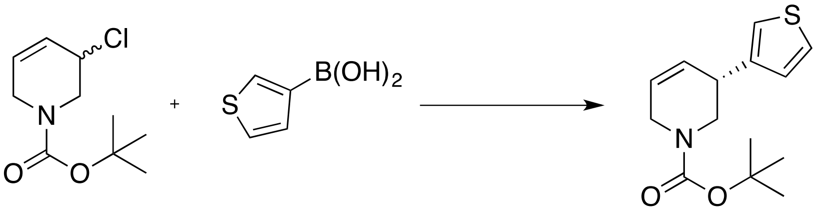

In a 10 mL round bottomed flask $[Rh(cod)(OH)]_2$ (4.6 mg, 0.01 mmol, 0.025 eq), (*R*)-(+)-5,5′-dichloro-2,2′-bis(diphenylphosphino)-6,6′-dimethoxy-1,1′-biphenyl (15.6 mg, 0.024 mmol, 0.06 eq) and Cs_2CO_3 (130.3 mg, 0.40 mmol, 1.00 eq) were stirred in THF (2 mL) at 60 °C for 30 min under an argon. A solution of *N-tert*-butoxycarbonyl-5-chloro-3-piperidene (86.8 mg, 0.40 mmol, 1.00 eq) in THF (0.75 mL) and a solution of 3-thienylboronic acid (102.4 mg, 0.80 mmol, 2.00 eq) in THF (0.75 mL) were sequentially added *via* syringe and both flasks were rinsed with THF (0.25 mL each). The resulting mixture was then stirred for 16 h at 80 °C in a sealed flask. SiO_2 (20 mg) was added and the solvent was then carefully evaporated. The resulting solid was directly loaded onto a chromatographic column and eluted with pentane:Et_2O (9:1) to afford (+)-(*R*)-*N-tert*-butoxycarbonyl-5-(3-thienyl)-3-piperidene in 74% yield (78 mg, 0.30 mmol).

Enantiomeric excess of 93% was determined by HPLC [Chiralpak® IA; flow: 1.0 mL/min; hexane/*i*-PrOH: 99: 1; λ = 210 nm; major enantiomer t_R = 7.2 min; minor enantiomer t_R = 7.8 min].

^{1}H NMR (400 MHz, Chloroform-*d*) δ 1.20 – 1.46 (m, 9H), 3.53 (rotameric s, 2H), 3.53 (s, 1H) 3.70 – 4.12 (rotameric, m, 2H), 5.66 – 5.92 (m, 2H), 6.86 – 6.99 (m, 2H), 7.17 – 7.23 (m, 1H).

^{13}C NMR (101 MHz, Chloroform-*d*) δ 28.4 (3C), 36.9, 42.9, 47.5, 79.5, 121.1, 125.5, 125.6, 127.3, 128.1, 142.9, 154.7.

IR (ATR) v_{max} /cm^{-1} = 2976 (s), 1693 (l), 1477 (s), 1420 (m), 1365 (m), 1333 (s), 1300 (s), 1240 (m), 1172 (m), 1113 (s), 1012 (s), 959 (s), 857 (s), 781 (s), 739 (s), 648 (s).

HRMS (ESI): *m/z* calc. for $C_{14}H_{19}O_2NNaS$ $[M+Na]^+$: 288.1029, found: 288.1028.

$[\alpha]^{25}{}_{589}$ = +125.9° (*c* 1.00, $CHCl_3$).

(+)-(*R*)-*N-tert*-Butoxycarbonyl-5-(6-indolyl)-3-piperidene (68)

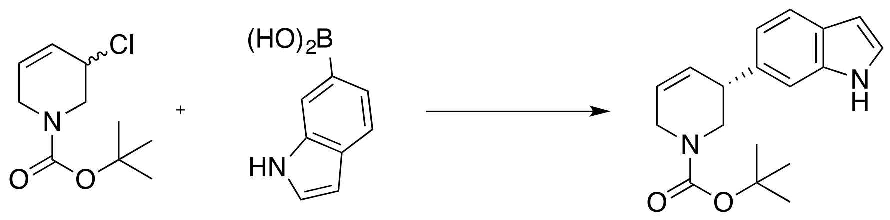

In a 10 mL round bottomed flask $[Rh(cod)(OH)]_2$ (4.6 mg, 0.01 mmol, 0.025 eq), (*R*)-(+)-5,5′-dichloro-2,2′-bis(diphenylphosphino)-6,6′-dimethoxy-1,1′-biphenyl (15.6 mg, 0.024 mmol, 0.06 eq) and Cs_2CO_3 (130.3 mg, 0.40 mmol, 1.00 eq) were stirred in THF (2 mL) at 60 °C for 30 min. A solution of *N-tert*-butoxycarbonyl-5-chloro-3-piperidene (86.8 mg, 0.40 mmol, 1.00 eq) in THF (0.75 mL) and a solution of 6-indolylboronic acid (128.8 mg, 0.80 mmol, 2.00 eq) in THF (0.75 mL) were sequentially added *via* syringe and both flasks were rinsed with THF (0.25 mL each). The resulting mixture was then stirred for 16 h at 80 °C in a sealed flask. SiO_2 (20 mg) was added and the solvent was then carefully evaporated. The resulting solid was directly loaded onto a chromatographic column and eluted with pentane:Et_2O (1:1) to afford (+)-(*R*)-*N-tert*-butoxycarbonyl-5-(6-indolyl)-3-piperidene in 80% yield (95.4 mg, 0.32 mmol).

Enantiomeric excess of 91% was determined by HPLC [Chiralpak® IC; flow: 1.0 mL/min; hexane/i-PrOH: 95: 5; λ = 210 nm; major enantiomer t_R = 9.6 min; minor enantiomer t_R = 14.6 min].

^{1}H NMR (400 MHz, Chloroform-*d*) δ 1.17 – 1.62 (m, 9H), 3.03, 3.40 – 3.58, 3.74 – 3.96 and 3.97 – 4.34 (rotameric m, 2H), 3.65 (s, 1H), 3.74 – 3.96 and 3.97 – 4.34 (rotameric m, 2H), 5.82 – 6.03 (m, 2H), 6.53 (s, 1H), 7.01 (d, *J* = 8.2 Hz, 1H), 7.14 (s, 1H), 7.19 (s, 1H), 7.61 (d, *J* = 8.1 Hz, 1H), 8.54 (s, 1H).

^{13}C NMR (101 MHz, Chloroform-*d*) δ 28.2 (3C), 41.8, 43.1 and 43.7 (rotameric, 1C), 47.9 and 49.1 (rotameric, 1C), 79.5, 102.1, 110.3, 120.2, 120.6, 124.4, 125.4, 126.8, 129.2, 135.9, 136.2, 155.0.

IR (ATR) ν_{max} /cm^{-1} = 3316 (s), 2975 (s), 2924 (s), 1677 (l), 1424 (m), 1358 (s), 1299 (s), 1246 (m), 1165 (m), 1112 (s), 963 (s), 860 (s), 812 (s), 762 (s), 729 (s).

HRMS (CI): *m*/*z* calc. for $C_{18}H_{23}O_2N_2$ $[M+H]^+$: 299.1754, found: 299.1756.

$[\alpha]^{25}_{589}$ = +119.5° (*c* 1.00, $CHCl_3$).

(–)-(*R*)-*N-tert*-Butoxycarbonyl-5-(2-chloro-6-pyridinyl)-3-piperidene (69)

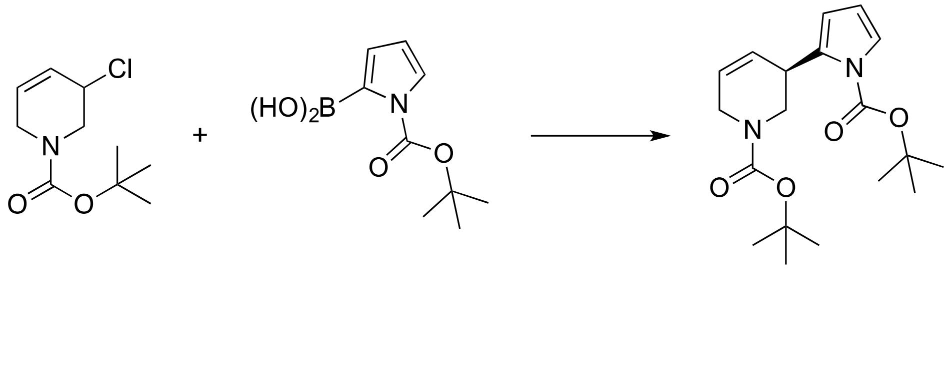

In a 10 mL round bottomed flask $[Rh(cod)(OH)]_2$ (4.6 mg, 0.01 mmol, 0.025 eq), *(S)*-BINAP (14.9 mg, 0.024 mmol, 0.06 eq) and Cs_2CO_3 (130.3 mg, 0.40 mmol, 1.00 eq) were stirred in THF (2 mL) at 80 °C for 30 min. A solution of *N-tert*-butoxycarbonyl-5-chloro-3-piperidene (86.8 mg, 0.40 mmol, 1.00 eq) in THF (0.75 mL) and a solution of *N*-(*tert*-butoxycarbonyl)pyrrole-2-boronic acid (253 mg, 1.20 mmol mmol, 3.00 eq) in THF (0.75 mL) were sequentially added *via* syringe and both flasks were rinsed with THF (0.25 mL each). The resulting mixture was then stirred for 16 h at 80 °C in a sealed flask. SiO_2 (20 mg) was added and the solvent was then carefully evaporated. The resulting solid was directly loaded onto a chromatographic column and eluted with petrol ether:ethyl acetate (9:1) to afford (–)-(*R*)-*N-tert*-butoxycarbonyl-5-(2-chloro-6-pyridinyl)-3-piperidene in 17% yield (23.9 mg, 0.07 mmol).

HPLC analysis indicated an enantiomeric excess of 95% [Chiralpak® IC; flow: 1.0 mL/min; hexane/*i*-PrOH 98:2; λ = 210 nm; major enantiomer t_R = 11.1 min; minor enantiomer t_R = 13.1 min].

^{1}H-NMR (400 MHz, $CDCl_3$) δ 1.13 – 1.50 (rotameric m, 9 H, CH_3), 1.60 (s, 9 H, CH_3), 3.33 – 3.37, 3.64 – 3.79 (rotameric m, 2 H), 3.80 – 4.13 (rotameric m, 2 H), 4.13 – 4.41 (rotameric m, 1 H), 5.67 – 5.99 (rotameric m, 3 H), 6.04 (dd, *J* = 3.3, 3.3 Hz, 1 H), 7.19 – 7.30 (m, 1 H).

^{13}C-NMR (100 MHz, $CDCl_3$) δ 28.2 (3C), 28.2 (3C), 34.8, 43.2, 46.4, 79.1, 83.6, 110.3, 113.8, 121.9, 126.4, 127.2, 135.2, 149.6, 154.9.

IR (ATR) ν_{max} /cm^{-1} = 2977s, 1739l, 1694l, 1480s, 1424m, 1368m, 1325l, 1239m, 1165l, 1118l, 1064s.

HRMS (ESI): *m/z* calc. for $C_{19}H_{28}N_2O_4Na$ $[M+Na]^+$: 371.1941, found: 371.1939.

$[\alpha]^{25}_{589}$ = –3.6° (*c* 1.00, $CHCl_3$).

(+)-(*S*)-*N-tert*-Butoxycarbonyl-5-(2-chloro-6-pyridinyl)-3-piperidene (70)

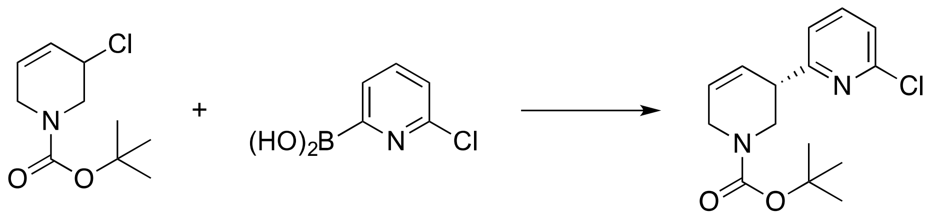

In a 10 mL round bottomed flask $[Rh(cod)(OH)]_2$ (4.6 mg, 0.01 mmol, 0.025 eq), (*R*)-(+)-5,5′-dichloro-2,2′-bis(diphenylphosphino)-6,6′-dimethoxy-1,1′-biphenyl (15.6 mg, 0.024 mmol, 0.06 eq) and Cs_2CO_3 (390.9 mg, 1.20 mmol, 3.00 eq) were stirred in THF (2 mL) at 80 °C for 30 min. A solution of *N-tert*-butoxycarbonyl-5-chloro-3-piperidene (86.8 mg, 0.40 mmol, 1.00 eq) in THF (0.75 mL) and a sonicated solution of 6-chloro-2-pyridinylboronic acid (189 mg, 1.20 mmol, 3.00 eq) and water (22 µL, 1.20 mmol, 3.00 eq) in THF (0.75 mL) were sequentially added *via* syringe and both flasks were rinsed with THF (0.25 mL each). The resulting mixture was then stirred for 16 h at 80 °C in a sealed flask. SiO_2 (20 mg) was added and the solvent was then carefully evaporated. The resulting solid was directly loaded onto a chromatographic column and eluted with a petrol:ethyl acetate (gradient from 15 to 20%) to afford (+)-(*S*)-*N-tert*-butoxycarbonyl-5-(2-chloro-6-pyridinyl)-3-piperidene in 40% yield (47.2 mg, 0.16 mmol).

HPLC for analysis indicated an enantiomeric excess of 95% [Chiralpak® IB; flow: 1.2 mL/min; hexane/*i*-PrOH 99.2:0.8; λ = 210 nm; major enantiomer t_R = 8.3 min; minor enantiomer t_R = 9.4 min].

^{1}H-NMR (400 MHz, $CDCl_3$) δ 1.23 – 1.43 (rotameric m, 9H), 3.45 – 4.33 (rotameric m, 5H), 5.96 (m, 2H), 7.10 (d, *J* = 7.6 Hz, 1H), 7.20 (d, *J* = 7.9 Hz, 1H), 7.56 (t, *J* = 7.8, 7.8 Hz, 1H).

^{13}C-NMR (100 MHz, $CDCl_3$) δ 28.4 (3C), 43.2, 43.6, 46.8, 79.7, 120.7, 122.3, 125.9, 127.7, 139.1, 151.1, 154.7, 162.8.

IR (ATR) ν_{max} /cm^{-1} = 1697l, 1583s, 1561s, 1415l, 1367m, 1131s, 1302m, 1239s, 1162l, 797s.

HRMS (ESI): *m/z* calc. for $C_{15}H_{19}N_2O_2ClNa$ $[M+Na]^+$: 317.1038, found: 317.1028.

[α]^{25}$_{589}$ = +45.9° (*c* 1.00, $CHCl_3$).

(+)-(*S*,*E*)-*N*-*tert*-Butoxycarbonyl-5-styryl-3-piperidene (71)

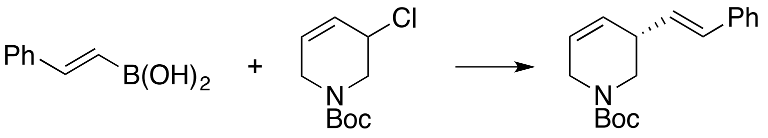

In a 10 mL round bottomed flask $[Rh(cod)(OH)]_2$ (4.6 mg, 0.01 mmol, 0.025 eq), (*R*)-BINAP (14.9 mg, 0.024 mmol, 0.06 eq) and Cs_2CO_3 (130.3 mg, 0.40 mmol, 1.00 eq) were stirred in THF (2 mL) at 60 °C for 30 min. A solution of *N-tert*-butoxycarbonyl-5-chloro-3-piperidene (86.8 mg, 0.40 mmol, 1.00 eq) in THF (0.75 mL) and a solution of *trans*-phenylvinylboronic acid (118.4 mg, 0.80 mmol, 2.00 eq) in THF (0.75 mL) were sequentially added *via* syringe and both flasks were rinsed with THF (0.25 mL each). The resulting mixture was then stirred for 16 h at 80 °C in a sealed flask. SiO_2 (20 mg) was added and the solvent was then carefully evaporated. The resulting solid was directly loaded onto a chromatographic column and eluted with hexane:EtOAc (9:1) to obtain (+)-(*S*,*E*)-*N*-*tert*-butoxycarbonyl-5-styryl-3-piperidene in 91% yield (103.5 mg, 0.36 mmol).

Enantiomeric excess of 96% was determined by HPLC [Chiralpak® IA; flow: 0.8 mL/min; hexane/i-PrOH: 99.4: 0.6; λ = 210 nm; major enantiomer t_R = 12.2 min; minor enantiomer t_R = 13.2 min].

^{1}H NMR (400 MHz, $CDCl_3$) δ 7.33 – 7.17 (m, 4H), 7.20 – 7.08 (m, 1H), 6.37 (d, *J* = 15.9 Hz, 1H), 6.05 (dd, *J* = 16.0, 7.4 Hz, 1H), 5.72 (s, 2H), 4.02 – 3.63 (m, 4H), 3.14 – 2.90 (m, 1H), 1.41 (s, 2H), 1.37 (s, 7H).

^{13}C NMR (101 MHz, $CDCl_3$) δ 155.0, 137.4, 130.9, 130.2, 128.6 (2C), 127.4, 126.3, 80.5, 79.8, 46.2, 44.9, 43.7, 42.9, 38.9, 31.1, 28.8, 28.6.

IR (ν_{max}/cm^{-1}): 3028, 1692, 1418, 1365.

HRMS (EI) *m/z* calc. for $C_{18}H_{23}NaNO_2^+$ $[M]^+$: 308.1621, found: 308.1621.

[α]^{25}$_{589}$ = +162.7° (c 3.03, $CHCl_3$) for 92% ee.

(–)-(*S*)-*N-tert*-Butoxycarbonyl-5-(1-phenylvinyl)-3-piperidene (72)

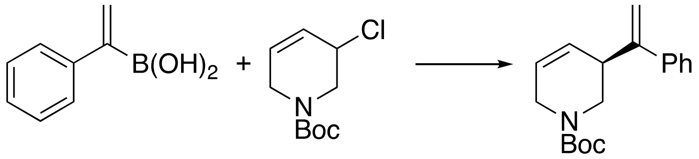

In a 10 mL round bottomed flask $[Rh(cod)(OH)]_2$ (4.6 mg, 0.01 mmol, 0.025 eq), (*S*)-(+)-5,5'-dichloro-2,2'-bis(diphenylphosphino)-6,6'-dimethoxy-1,1'-biphenyl (15.6 mg, 0.024 mmol, 0.06 eq) and Cs_2CO_3 (130.3 mg, 0.40 mmol, 1.00 eq) were stirred in THF (2 mL) at 60 °C for 30 min. The mixture was then allowed to cool to room temperature before the addition of a solution of *N-tert*-butoxycarbonyl-5-chloro-3-piperidene (86.8 mg, 0.40 mmol, 1.00 eq) in THF (0.75 mL) and a solution of (1-phenylvinyl)boronic acid (118.4 mg, 0.80 mmol, 2.00 eq) in THF (0.75 mL) *via* syringe and both flasks were rinsed with THF (0.25 mL each). The resulting mixture was then stirred for 72 h at RT under Ar. SiO_2 (20 mg) was added and the solvent was then carefully evaporated. The resulting solid was directly loaded onto a chromatographic column and eluted with pentane:Et_2O (9:1) to afford (–)-(*S*)-*N-tert*-butoxycarbonyl-5-(1-phenylvinyl)-3-piperidene in 78% yield (89 mg, 0.31 mmol).

Enantiomeric excess of 99% was determined by HPLC [Chiralpak® ID; flow: 1.0 mL/min; hexane/i-PrOH: 99: 1; λ = 210 nm; minor enantiomer t_R = 8.5 min; major enantiomer t_R = 9.9 min].

^{1}H NMR (400 MHz, $CDCl_3$) δ 7.34 (m, 2H), 7.31 – 7.16 (m, 3H), 5.86 – 5.63 (m, 2H), 5.28 (m, 1H), 5.01 – 4.96 (m, 1H), 3.88 – 3.71 (m, 2.2H), 3.45 – 3.36 (m, 2.3H), 3.10 (s, 0.5H), 1.46 – 1.23 (m, 9H).

^{13}C NMR (101 MHz, $CDCl_3$) δ 154.9, 130.0, 128.5, 128.4 (2 C), 127.9, 127.5, 126.4, 126.2, 125.8, 114.5, 79.5, 77.2, 45.6, 43.1, 40.0, 28.5, 28.3.

IR ($ν_{max}$ /cm^{-1}): 1695, 2975.

HRMS (EI) *m/z* calc. for $C_{18}H_{24}NO_2$ $[M+1]^+$: 286.1802, found: 286.1801.

$[α]^{25}_{589}$ = –65.82° (c 3.15, $CHCl_3$) for 99% ee.

~~**(+)-(*R*)-*N-tert*-Butoxycarbonyl-5-(1-phenylvinyl)-3-piperidene (72)**~~

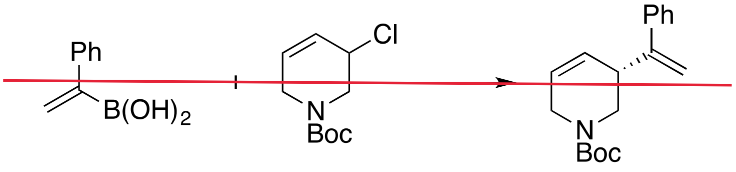

~~In a 10 mL round bottomed flask $[Rh(cod)(OH)]_2$ (4.6 mg, 0.01 mmol, 0.025 eq), (*R*)-(+)-5,5'-dichloro-2,2'-bis(diphenylphosphino)-6,6'-dimethoxy-1,1'-biphenyl (15.6 mg, 0.024 mmol, 0.06 eq) and Cs_2CO_3 (130.3 mg, 0.40 mmol, 1.00 eq) were stirred in THF (2 mL) at 60 °C for 30 min. A solution of *N-tert*-butoxycarbonyl-5-chloro-3-piperidene (86.8 mg, 0.40 mmol, 1.00 eq) in THF (0.75 mL) and a solution of (1-phenylvinyl)boronic acid (118.4 mg, 0.80 mmol, 2.00 eq) in THF (0.75 mL) were sequentially added *via* syringe and both flasks were rinsed with THF (0.25 mL each). The resulting mixture was then stirred for 16 h at 80 °C in a sealed flask. SiO_2 (20 mg) was added and the solvent was then carefully evaporated. The~~

resulting solid was directly loaded onto a chromatographic column and eluted with hexane:EtOAc (9:1) to afford (+)-(*R*)-*N-tert*-butoxycarbonyl-5-(1-phenylvinyl)-3-piperidene in 99% yield (113.9 mg, 0.39 mmol).

Enantiomeric excess of 99% was determined by HPLC [Chiralpak® IA; flow: 0.8 mL/min; hexane/i-PrOH: 99.4: 0.6; λ = 210 nm; major enantiomer t_R = 12.2 min; minor enantiomer t_R = 13.2 min].

^{1}H NMR (400 MHz, $CDCl_3$) δ 7.35 (d, *J* = 8.1 Hz, 1H), 7.31 – 7.08 (m, 3H), 6.37 (d, *J* = 15.9 Hz, 1H), 6.05 (dd, *J* = 16.1, 7.4 Hz, 1H), 5.76 (d, *J* = 30.7 Hz, 2H), 4.06 – 3.67 (m, 3H), 3.39 (s, 2H), 3.22 – 2.86 (m, 1H), 1.47 – 0.99 (m, 9H).

^{13}C NMR (101 MHz, $CDCl_3$) δ 155.3, 137.7, 131.3, 130.5, 128.9, 128.8, 127.9, 127.7, 126.6, 126.2, 115.0, 100.4, 80.1 and 79.9 (rotameric, 1C), 45.9, 43.5, 40.4, 28.9, 28.8.

IR (ν_{max}/cm^{-1}): 3028, 2928, 2839, 1695, 1419, 1365.

HRMS (EI) *m/z* calc. for $C_{18}H_{23}NaNO_2$ $[M]^+$: 308.1621, found: 308.1627.

$[\alpha]^{25}_{589}$ = +123.3° (c 0.99, $CHCl_3$) for 99% ee.

(+)-(*R*,*E*)-*N-tert*-Butoxycarbonyl-5-(5-pheny-1-pentenyl)-3-piperidene (73)

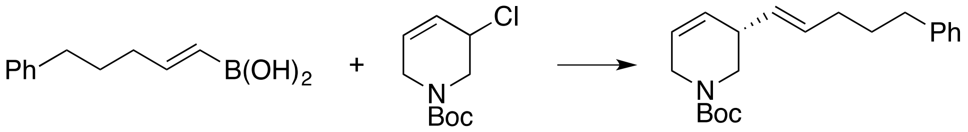

In a 10 mL round bottomed flask $[Rh(cod)(OH)]_2$ (4.6 mg, 0.01 mmol, 0.025 eq), (*R*)-BINAP (14.9 mg, 0.024 mmol, 0.06 eq) and Cs_2CO_3 (130.3 mg, 0.40 mmol, 1.00 eq) were stirred in THF (2 mL) at 60 °C for 30 min. A solution of *N-tert*-butoxycarbonyl-5-chloro-3-piperidene (86.8 mg, 0.40 mmol, 1.00 eq) in THF (0.75 mL) and a solution of (5-phenyl-1-pentenyl)boronic acid (152.0 mg, 0.80 mmol, 2.00 eq) in THF (0.75 mL) were sequentially added *via* syringe and both flasks were rinsed with THF (0.25 mL each). The resulting mixture was then stirred for 16 h at 80 °C in a sealed flask. SiO_2 (20 mg) was added and the solvent was then carefully evaporated. The resulting solid was directly loaded onto a chromatographic column and eluted with hexane:EtOAc (9:1) to afford (+)-(*R*,*E*)-*N-tert*-Butoxycarbonyl-5-(5-pheny-1-pentenyl)-3-piperidene in 88% yield (114.5 mg, 0.35 mmol).

Enantiomeric excess of 98% was determined by HPLC [Chiralpak® IB (2 x IB columns connected; flow: 1.20 mL/min; hexane/i-PrOH: 99.4: 0.6; λ = 210 nm; major enantiomer t_R = 12.0 min; minor enantiomer t_R = 13.3 min].

^{1}H NMR (400 MHz, $CDCl_3$) δ 7.20 (m, 2H), 7.15 – 7.05 (m, 3H), 5.62 (s, 2H), 5.44 (dtd, *J* = 15.4, 6.6, 1.1 Hz, 1H), 5.27 (ddt, *J* = 15.4, 7.3, 1.4 Hz, 1H), 3.86 (m, *J* = 19.3 Hz, 1H), 3.71 (m, 1H), 3.50 (m, 1H), 2.98 (m, 1H), 2.79 (m, 1H), 2.53 (t, *J* = 7.7 Hz, 2H), 1.97 (q, *J* = 7.1 Hz, 2H), 1.61 (t, *J* = 7.7 Hz, 2H), 1.38 (s, 9H).

^{13}C NMR (101 MHz, $CDCl_3$) δ 142.5, 131.4, 130.4, 128.5 (2C), 128.3 (2C), 125.7, 100.0, 79.5, 46.9, 44.9, 35.3, 32.1, 31.1, 28.5 (3C).

IR (ν_{max}/cm^{-1}): 3028, 2928, 2839, 1695, 1419, 1365.

HRMS (CI) *m/z* calc. for $C_{18}H_{30}NO_2$ $[M+H]^+$: 328.2277, found: 328.2274.

$[\alpha]^{25}_{589}$ = +121.3° (c 0.89, $CHCl_3$) for 98% ee.

8. Analytical data for figure 5

(–)-(*S*)-2-(Cyclohex-2-en-1-yl)-6-phenylpyridine (74)

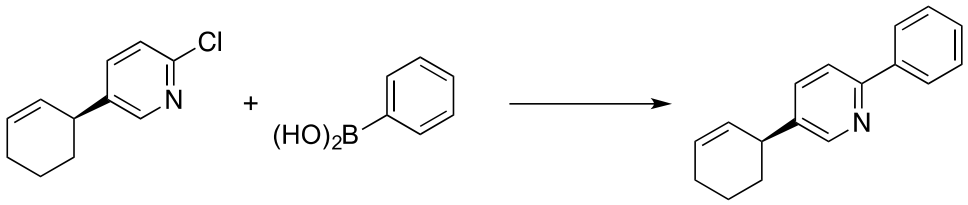

In a 5 mL round bottomed flask were added **45a** (38.7 mg, 0.20 mmol, 1.00 eq), benzeneboronic acid (36.3 mg, 0.30 mmol, 1.50 eq), palladium(II) acetate (2.3 mg, 0.01 mmol, 0.05 eq), SPhos (8.2 mg, 0.02 mmol, 0.10 eq) and K_2CO_3 (83 mg, 0.60 mmol, 3.00 eq). MeCN (0.75 mL) and H_2O (0.5 mL, degassed by sonication *in vacuo* for 2 min prior to the addition) were added and the flask was sealed under Ar. The resulting mixture was stirred for 1 h at 100 °C. The reaction mixture was then extracted with ethyl acetate (2 × 3 mL), dried over $MgSO_4$, filtered over a plug of Celite® and evaporated *in vacuo*. The resulting residue was purified by column chromatography over silica and eluted with petrol ether:EtOAc (97:3) to afford (–)-(*S*)-2-(cyclohex-2-en-1-yl)-6-phenylpyridine in 58% yield (27.3 mg, 0.12 mmol).

HPLC analysis indicated an enantiomeric excess of 98% [Chiralpak® IE; flow: 1.0 mL/min; hexane/*i*-PrOH 98:2; λ = 210 nm; major enantiomer t_R = 13.5 min; minor enantiomer t_R = 14.5 min].

^{1}H-NMR (400 MHz, $CDCl_3$) δ 1.52 – 1.72 (m, 2 H), 1.72 – 1.82 (m, 1 H), 1.99 – 2.17 (m, 3 H), 3.48 (m, 1 H), 5.67 – 5.77 (m, 1 H), 5.97 (ddt, *J* = 9.9, 3.7, 2.3, 2.3 Hz, 1 H), 7.36 – 7.43 (m, 1 H), 7.43 – 7.52 (m, 2 H), 7.60 (dd, *J* = 8.1, 2.3 Hz, 1 H), 7.67 (dd, *J* = 8.2, 0.9 Hz, 1 H), 7.93 – 8.05 (m, 2 H), 8.56 (d, *J* = 2.3 Hz, 1 H).

^{13}C-NMR (100 MHz, $CDCl_3$) δ 20.9, 24.9, 32.3, 39.0, 120.2, 126.8 (2C), 128.6, 128.7, 128.8 (2C), 129.4, 136.0, 139.4, 140.2, 149.4, 155.3.

IR (ATR) ν_{max} /cm^{-1} = 3021s, 2929m, 2857s, 1473l, 1446s, 1021s, 843s, 784s, 742l, 724s, 694m.

HRMS (EI/FI): *m/z* calc. for $C_{17}H_{17}N$ $[M]^+$: 235.1361, found: 235.1349.

$[\alpha]^{25}_{589}$ = –126.8° (*c* 1.00, $CHCl_3$).

(–)-(*S*)-3-(5-(Cyclohex-2-en-1-yl)pyridin-2-yl)oxazolidin-2-one (75)

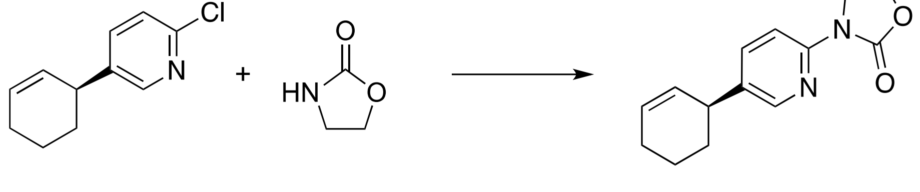

In a 5 mL round bottomed flask purged with Ar were added (–)-(*S*)-2-chloro-5-(cyclohex-2-en-1-yl)-pyridine **45a** (35 mg, 0.18 mmol, 1.00 eq), 2-oxazolidinone (24 mg, 0.27 mmol, 1.50

eq), copper(I) iodide (34 mg, 0.18 mmol, 1.00 eq), *N,N'*-dimethylethylenediamine (0.2 mL, 0.27 mmol, 1.50 eq) and K_2CO_3 (50 mg, 0.36 mmol, 2.00 eq) and suspended in toluene (1.0 mL). The flask was sealed and the mixture was then stirred for 60 h at 140 °C. The reaction was quenched at room temperature by the addition of NH_4Cl (sat. aq., 2 mL). The mixture was extracted with ethyl acetate (2 × 3 mL) and the combined organic layers were washed with brine (2 mL), dried over $MgSO_4$, filtered and evaporated *in vacuo*. The resulting residue was purified by SiO_2 flash column chromatography and eluted with petrol ether:EtOAc (1:1) to produce (–)-(*S*)-3-(5-(cyclohex-2-en-1-yl)pyridin-2-yl)oxazolidin-2-one in 53% yield (67% brsm, 23.1 mg, 0.09 mmol).

^{1}H-NMR (400 MHz, $CDCl_3$) δ 1.52 (dddd, *J* = 13.0, 10.1, 8.0, 2.9 Hz, 1 H), 1.57 – 1.67 (m, 1 H), 1.67 – 1.77 (m, 1 H), 1.95 – 2.04 (m, 1 H), 2.05 – 2.13 (m, 2 H), 3.32 – 3.47 (m, 1 H), 4.21 – 4.33 (m, 2 H), 4.44 – 4.53 (m, 2 H), 5.60 – 5.70 (m, 1 H), 5.93 (dddd, *J* = 9.9, 3.7, 3.7, 2.3 Hz, 1 H), 7.55 (dd, *J* = 8.7, 2.4 Hz, 1 H), 8.12 (d, *J* = 8.6 Hz, 1 H), 8.17 (d, *J* = 2.4 Hz, 1 H).

^{13}C-NMR (100 MHz, $CDCl_3$) δ 20.8, 24.9, 32.4, 38.7, 44.1, 62.0, 112.6, 128.8, 129.3, 137.1, 137.3, 146.7, 149.2, 155.1.

IR (ATR) ν_{max} /cm^{-1} = 2929s, 1745l, 1602s, 1482m, 1407l, 1225m, 1227m, 1045s, 1010s, 835s, 753s.

HRMS (ESI): *m/z* calc. for $C_{14}H_{17}N_2O_2$ $[M+H]^+$: 245.1285, found: 245.1286.

$[\alpha]^{25}_{589}$ = –119.3° (*c* 1.00, $CHCl_3$).

(–)-(*S*)-4-(6-(Cyclohex-2-en-1-yl)pyridin-2-yl)morpholine (76)

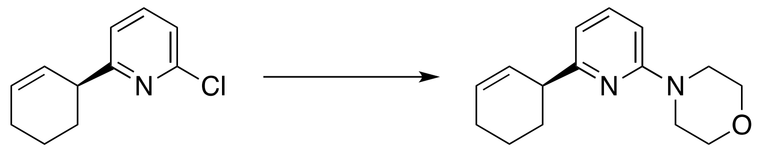

(–)-(*S*)-2-Chloro-6-(cyclohex-2-en-1-yl)-pyridine **45b** (36 mg, 0.16 mmol) in morpholine (0.5 mL) was stirred for 16 h at 130 °C in a sealed vial under argon. Water (1 mL) and ethyl acetate (2 mL) were then added and the aqueous layer extracted with ethyl acetate (2 mL). The combined organic layers were washed with brine (1 mL), dried over $MgSO_4$, filtered and evaporated *in vacuo*. The crude was purified by column chromatography over silica and eluted with petrol ether:EtOAc (9:1) to give (–)-(*S*)-4-(6-(cyclohex-2-en-1-yl)pyridin-2-yl)morpholine in 38% yield (50% brsm, 15.1 mg, 0.06 mmol).

^{1}H-NMR (400 MHz, $CDCl_3$) δ 1.59 – 1.69 (m, 1 H), 1.75 (dddd, *J* = 12.4, 10.6, 8.3, 2.8 Hz, 2 H), 1.97 – 2.10 (m, 3 H), 3.42 (m, 1 H), 3.46 – 3.55 (m, 4 H), 3.76 – 3.86 (m, 4 H), 5.84 (dt, *J* = 7.4, 2.7, 2.7 Hz, 2 H), 6.45 (d, *J* = 8.4 Hz, 1 H), 6.58 (d, *J* = 7.4 Hz, 1 H), 7.43 (dd, *J* = 8.3, 7.3 Hz, 1 H).

^{13}C-NMR (100 MHz, $CDCl_3$) δ 21.3, 25.3, 30.0, 44.0, 45.9 (2C), 67.0 (2C), 104.1, 111.6, 128.2, 129.6, 137.9, 159.3, 163.8.

IR (ATR) ν_{max} /cm^{-1} = 2925s, 2848s, 2581l, 1452l, 1371s, 1248m, 1120m, 976s, 791s, 736s.

HRMS (ESI): *m/z* calc. for $C_{15}H_{20}N_2O$ $[M+H]^+$: 245.1648, found: 245.1648.

[α]^{25}$_{589}$ = –10.6° (*c* 1.00, $CHCl_3$).

(–)-((*S*)-5-(Cyclohex-2-en-1-yl)thiophen-2-yl)phenylmethanol (77)

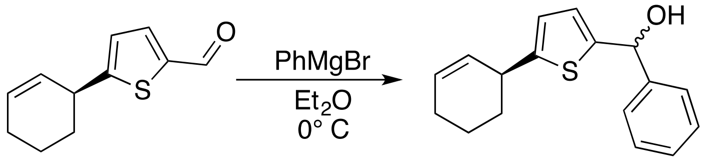

Phenylmagnesium bromide (3.0 M in Et_2O, 75 µL, 0.25 mmol, 2.30 eq) was added dropwise to a solution of (–)-(*S*)-5-(cyclohex-2-en-1-yl)thiophene-2-carbaldehyde **37** (21.0 mg, 0.11 mmol, 1.00 eq) in Et_2O (0.5 mL) at 0 °C. After 5 min the reaction mixture was allowed to reach room temperature and was stirred 45 min. NH_4Cl (sat. aq., 2 mL) was then added and the resulting mixture was extracted with Et_2O (2 × 3 mL). The combined extracts were dried over $MgSO_4$, filtered and evaporated *in vacuo*. The resulting residue was purified by column chromatography over silica and eluted with petrol ether and EtOAc (95:5) to obtain (–)-((*S*)-5-(cyclohex-2-en-1-yl)thiophen-2-yl)phenylmethanol in 81% yield (24.1 mg, 0.09 mmol, 1:1 d.r., verified by ^{13}C-NMR).

^{1}H-NMR (500 MHz, DMSO-*d$_6$*) δ 1.50 – 1.60 (m, 2 H), 1.61 – 1.72 (m, 1 H), 1.92 – 2.03 (m, 3 H), 3.56 – 3.63 (m, 1 H), 5.65 – 5.71 (m, 1 H), 5.76 – 5.82 (m, 1 H), 5.84 (d, *J* = 4.3 Hz, 1 H), 6.11 (m, 1 H), 6.64 (ddd, *J* = 3.5, 3.5, 0.9 Hz, 1 H), 6.68 (ddd, *J* = 3.5, 1.9, 0.9 Hz, 1 H), 7.22 – 7.27 (m, 1 H), 7.30 – 7.36 (m, 2 H), 7.38 – 7.43 (m, 2 H).

^{13}C-NMR (126 MHz, DMSO-*d$_6$*) δ 20.6 and 20.7 (1C, rotameric), 24.8, 32.4 and 32.4 (1C, rotameric), 36.6 and 36.6 (1C, rotameric), 71.8, 123.1 and 123.2 (1C, rotameric), 123.8 and 123.8 (1C, rotameric), 126.5 (2C), 127.5, 128.3 and 128.4 (1C, rotameric), 128.5 (2C, rotameric), 130.1 and 130.1 (1C, rotameric), 145.4, 148.2 and 148.2 (1C, rotameric), 149.1 and 149.2 (1C, rotameric).

IR (ATR) ν_{max} /cm^{-1} = 2932m, 1116l, 1089m, 1067l, 1047sh, 1028sh, 991l, 950m, 700s.

[α]^{25}$_{589}$ = –79.7° (*c* 1.00, $CHCl_3$).

(+)-(*S*)-2-(Tetrahydro-2*H*-pyran-3-yl)pyridine (78)

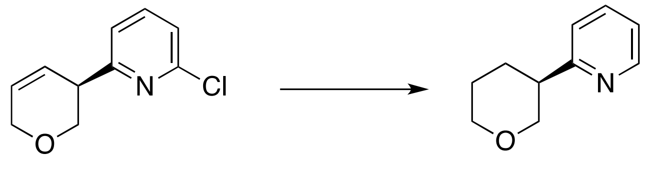

A 5 mL round bottomed flask was charged with (–)-(*S*)-2-chloro-6-(3,6-dihydro-2*H*-pyran-3-yl)pyridine **47** (51 mg, 0.26 mmol, 1.00 eq), Pd/C (10% on carbon, 3.4 mg, 3.2 µmol, 0.015 eq) and $NaHCO_3$ (21.8 mg, 0.26 mmol, 1.00 eq). MeOH (5 mL) was added and the reaction mixture was stirred overnight at room temperature under H_2 atmosphere (using a balloon). The resulting mixture was then filtered over Celite® and evaporated *in vacuo* to afford the chloride salt. The crude was then diluted in EtOAc (2 mL), washed with K_2CO_3 (sat. aq. 2 mL), dried over $MgSO_4$, filtered and evaporated *in vacuo* to obtain (+)-(*S*)-2-(tetrahydro-2*H*-pyran-3-yl)pyridine in 79% yield (33.5 mg, 0.21 mmol).

HPLC analysis indicated an enantiomeric excess of 97% [Chiralpak® IB; flow: 1.0 mL/min; hexane/*i*-PrOH 99:1; λ = 210 nm; minor enantiomer t_R = 13.7 min; major enantiomer t_R = 14.7 min].

^{1}H-NMR (400 MHz, $CDCl_3$) δ 1.65 – 1.98 (m, 3 H), 2.02 – 2.12 (m, 1 H), 3.02 (ddt, *J* = 10.8, 10.8, 4.0, 4.0 Hz, 1 H), 3.49 (ddd, *J* = 11.3, 11.2, 2.9 Hz, 1 H), 3.60 (dd, *J* = 10.8, 10.8 Hz, 1 H), 3.98 (m, 1 H), 4.08 (ddd, *J* = 11.2, 4.2, 2.0 Hz, 1 H), 7.12 (ddd, *J* = 7.5, 4.9, 1.2 Hz, 1 H), 7.16 – 7.21 (m, 1 H), 7.61 (ddd, *J* = 7.7, 7.7, 1.9 Hz, 1 H), 8.54 (ddd, *J* = 4.9, 1.9, 1.0 Hz, 1 H).

^{13}C-NMR (100 MHz, $CDCl_3$) δ 26.0, 29.5, 44.8, 68.3, 72.5, 121.7, 122.3, 136.6, 149.5, 162.2.

IR (ATR) ν_{max} /cm^{-1} = 2936m, 2847m, 1590m, 1472m, 1435m, 1088l, 1031s, 856s, 777s, 750s.

HRMS (CI): *m/z* calc. for $C_{10}H_{14}NO$ [M+H]$^+$: 164.1070, found: 164.1072.

$[\alpha]^{25}_{589}$ = +5.2° (*c* 1.00, $CHCl_3$).

(+)-(*R*)-*N*-*tert*-Butoxycarbonyl-3-(3-aminophenyl)-piperidine (79)

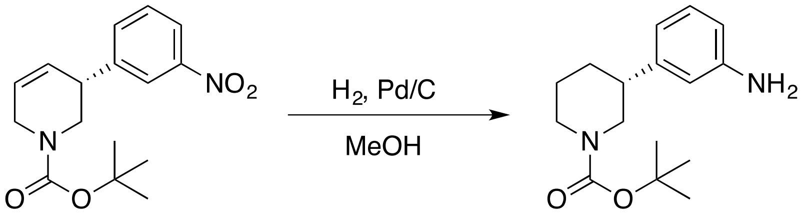

(*R*)-*N*-*tert*-butoxycarbonyl-5-(3-nitrophenyl)-3-piperidene **69** (62 mg, 0.20 mmol, 1.00 eq) and Pd/C (10% weight on carbon, 21 mg, 0.02 mmol, 0.10 eq) were suspended in MeOH (4 mL) under Ar. The mixture was stirred overnight at room temperature under H_2 atmosphere (using a balloon). The solids were then filtered over Celite®, washed with EtOAc and evaporated *in vacuo*. The resulting residue was purified by column chromatography over silica and eluted with pentane:Et_2O (1:1) to afford (+)-(*R*)-*N*-*tert*-butoxycarbonyl-3-(3-aminophenyl)-piperidine in 40% yield (22 mg, 0.08 mmol).

Enantiomeric excess of 93% was determined by HPLC [Chiralpak® IB; flow: 1.0 mL/min; hexane/*i*-PrOH: 80: 20; λ = 210 nm; major enantiomer t_R = 10.3 min; minor enantiomer t_R = 14.7 min].

^{1}H NMR (400 MHz, Chloroform-*d*) δ 1.47 (s, 9H), 1.51 – 1.65 (m, 2H), 1.74 (m, 1H), 1.96 – 2.04 (m, 1H), 2.57 (m, 1H), 2.70 (t, *J* = 11.9 Hz, 2H), 3.54 (br s, 2H), 4.15 (br s, 2H), 6.53 – 6.58 (m, 2H), 6.63 (dt, *J* = 7.6, 1.3 Hz, 1H), 7.06 – 7.13 (m, 1H).

^{13}C NMR (101 MHz, Chloroform-*d*) δ 25.5, 28.5 (3C), 31.7, 42.6, 44.0, 50.7, 79.4, 113.4, 114.0, 117.3, 129.4, 144.9, 146.5, 154.9.

IR (ATR) ν_{max} /cm^{-1} = 3363 (s), 2975 (s), 2933 (s), 2865 (s), 1680 (l), 1607 (m), 1422 (m), 1365 (m), 1302 (s), 1267 (m), 1166 (m), 1148 (m), 995 (s), 952 (s), 862 (s), 783 (s), 701 (s).

HRMS (ESI): *m/z* calc. for $C_{16}H_{24}O_2N_2Na$ [M+Na]$^+$: 299.1730, found: 299.1731.

$[\alpha]^{25}{}_{589}$ = +95.3° (*c* 1.00, $CHCl_3$).

(+)-(*S*,*Z*)-5-Bromo-2-(dibenzofuran-4-yl)pent-3-en-1-ol (80)

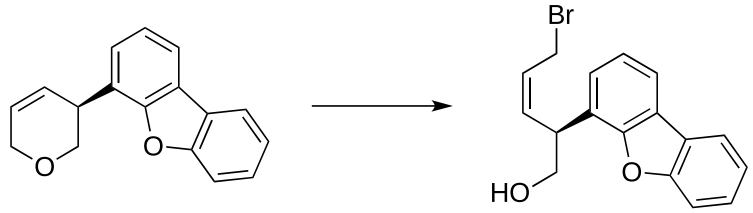

Boron tribromide (1 M in CH_2Cl_2, 0.17 mL, 0.17 mmol) was added over 5 min to a solution of (–)-(*S*)-4-(3,6-dihydro-2*H*-pyran-3-yl)dibenzofuran **43** (42.7 mg, 0.17 mmol) in CH_2Cl_2 (0.6 mL) at 0 °C. The reaction mixture was stirred for 1 h at 0 °C before the addition of water (2 mL). The aqueous layer was extracted with CH_2Cl_2 (3 x 1 mL). The combined organic layers were washed with brine, dried over $MgSO_4$ and concentrated *in vacuo*. The resulting residue was purified by SiO_2 column chromatography over silica and eluted with petrol ether:EtOAc (4:1) to obtain (+)-(*S*,*Z*)-5-bromo-2-(dibenzofuran-4-yl)pent-3-en-1-ol in 75% yield (42.3 mg, 0.13 mmol).

^{1}H-NMR (400 MHz, $CDCl_3$) δ 4.02 (dd, *J* = 10.7, 7.5 Hz, 1 H), 4.05 – 4.14 (m, 2 H), 4.18 – 4.26 (m, 1 H), 4.53 (ddd, *J* = 9.7, 6.9, 6.9 Hz, 1 H), 5.95 – 6.03 (m, 1 H), 6.07 (dd, *J* = 10.7, 9.8 Hz, 1 H), 7.29 – 7.39 (m, 3 H), 7.48 (ddd, *J* = 8.4, 7.3, 1.4 Hz, 1 H), 7.61 (d, *J* = 8.3 Hz, 1 H), 7.86 (dd, *J* = 6.0, 2.9 Hz, 1 H), 7.95 (d, *J* = 7.7 Hz, 1 H).

^{13}C-NMR (100 MHz, $CDCl_3$) δ 26.9, 41.8, 65.4, 111.9, 119.6, 120.9, 123.0, 123.4, 124.4 (2C), 124.7, 126.0, 127.4, 128.6, 133.5, 154.2, 156.1.

IR (ATR) ν_{max} /cm^{-1} = 3381s, 2937s, 1474s, 1450m, 1423m, 1328s, 1205m, 1185l, 1052ms, 843s, 753l.

HRMS (EI): *m*/*z* calc. for $C_{17}H_{15}BrO_2$ $[M]^+$: 330.0255, found: 330.0252.

$[\alpha]^{25}{}_{589}$ = +128.7° (*c* 1.00, $CHCl_3$).

(–)-(*S*)-*N*-*tert*-Butoxycarbonyl-3-(3-methoxyphenyl)-piperidine (2H-*ent*-55)

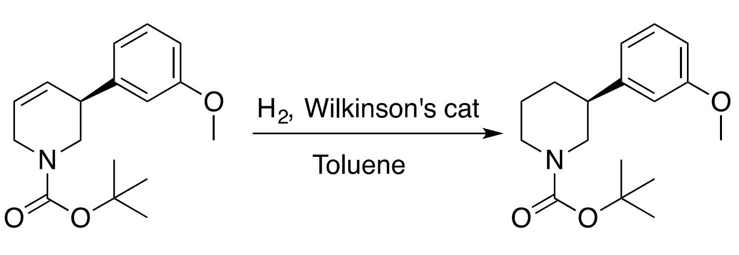

(*S*)-*N*-*tert*-Butoxycarbonyl-5-(3-methoxyphenyl)-3-piperidene *ent*-**55** (393 mg, 1.36 mmol, 1.00 eq) was dissolved in toluene (7 mL) and $(PPh_3)_3RhCl$ (227 mg, 0.25 mmol, 0.18 eq) was added. The reaction mixture was stirred overnight at room temperature under H_2 atmosphere (using a balloon). The catalyst was then filtered off using a Celite® pad and washed with EtOAc (5 mL) and the solvent was evaporated *in vacuo*. The resulting residue was purified by column chromatography over silica and eluted with hexane:EtOAc (4:1) to afford (–)-(*S*)-*N*-*tert*-butoxycarbonyl-3-(3-methoxyphenyl)-piperidine in 97% yield (383 mg, 1.32 mmol).

Enantiomeric excess of 96% was determined by HPLC [Chiralpak® IC; flow: 1.0 mL/min; hexane/*i*-PrOH: 80: 20; λ = 210 nm; minor enantiomer t_R = 14.5 min; major enantiomer t_R = 17.1 min].

^{1}H NMR (400 MHz, Chloroform-*d*) δ 1.47 (s, 9H), 1.49 – 1.69 (m, 2H), 1.75 (m, 1H), 1.97 – 2.07 (m, 1H), 2.44 – 2.98 (m, 3H), 3.80 (s, 3H), 4.16 (br s, 2H), 6.75 – 6.79 (m, 2H), 6.83 (dt, *J* = 7.6, 1.2 Hz, 1H), 7.19 – 7.30 (m, 1H).

^{13}C NMR (101 MHz, Chloroform-*d*) δ 25.5, 28.5 (3C), 31.8, 42.6, 44.3, 50.5, 55.2, 79.4, 111.6, 113.1, 119.5, 129.4, 145.3, 154.8, 159.7.

IR (ATR) ν_{max} /cm^{-1} = 2974 (s), 2934 (s), 2856 (s), 1691 (l), 1602 (s), 1584 (s), 1466 (s), 1419 (m), 1365 (s), 1285 (s), 1262 (m), 1155 (m), 1049 (s), 987 (s), 948 (s), 864 (s), 780 (s), 700 (s).

HRMS (ESI): *m/z* calc. for $C_{17}H_{25}O_3NNa$ [M+Na]$^+$: 314.1727, found: 314.1728.

[α]^{25}$_{589}$ = –46.4° (*c* 1.00, $CHCl_3$).

(+)-(*S*)-3-(3-Methoxyphenyl)-piperidine (81)

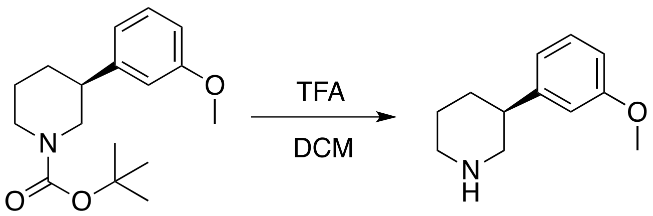

Trifluoroacetic acid (0.96 mL, 12.60 mmol, 10.0 eq) was added dropwise to a solution of (*S*)-*N-tert*-Butoxycarbonyl-3-(3-methoxyphenyl)-piperidine (0.37 g, 1.26 mmol, 1.00 eq) in CH_2Cl_2 (9.60 mL) at 0 °C. . The resulting mixture was allowed to warm to room temperature and stirred for another 30 min. The solution was then diluted with CH_2Cl_2 (5 mL), prior to the addition of $NaHCO_3$ (sat., aq.). The aqueous phase was extracted with EtOAc. The combined organic layers were washed with K_2CO_3 (sat., aq.) and brine, dried over $MgSO_4$, filtered and concentrated *in vacuo* to obtain (+)-(*S*)-3-(3-methoxyphenyl)-piperidine in 88% yield (0.21 g, 1.11 mmol).

^{1}H NMR (400 MHz, Chloroform-*d*) δ 1.51 – 1.66 (m, 2H), 1.78 (m, 2H), 2.00 (m, 1H), 2.58 – 2.70 (m, 3H), 3.06 – 3.14 (m, 1H), 3.16 (m, 1H), 3.80 (s, 3H), 6.72 – 6.78 (m, 2H), 6.81 (dt, *J* = 7.6, 1.2 Hz, 1H), 7.22 (td, *J* = 7.8, 0.6 Hz, 1H).

^{13}C NMR (101 MHz, Chloroform-*d*) δ 27.1, 32.1, 44.4, 46.7, 54.1, 55.1, 111.2, 113.2, 119.5, 129.3, 146.7, 159.6.

IR (ATR) ν_{max} /cm^{-1} = 3313 (br), 3000 (s), 2931 (l), 2851 (m), 1603 (l), 1585 (m), 1490 (m), 1464 (m), 1435 (m), 1267 (l), 1162 (m), 1046 (m), 931 (s), 859 (s), 783 (m), 754 (s), 699 (m).

HRMS (ESI): *m/z* calc. for $C_{12}H_{18}ON$ [M+H]$^+$: 192.1383, found: 192.1382.

[α]^{25}$_{589}$ = +6.1 (*c* 1.00, $CHCl_3$).

(–)-(*S*)-*N-tert*-Butoxycarbonyl-3-(3-hydroxyphenyl)-piperidine (2H-*ent*-57)

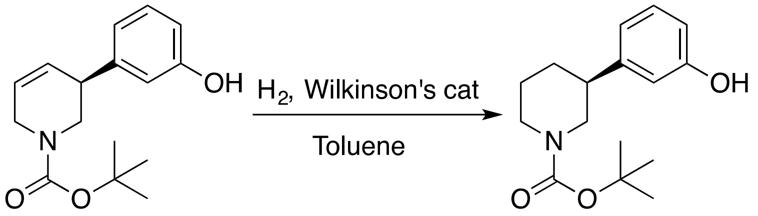

(*S*)-*N*-*tert*-butoxycarbonyl-5-(3-hydroxyphenyl)-3-piperidene *ent*-**57** (758 mg, 2.75 mmol, 1.00 eq) was dissolved in toluene (14 mL) and $(PPh_3)_3RhCl$ (458 mg, 0.49 mmol, 0.18 eq) was added. The reaction mixture was stirred overnight at room temperature under H_2 atmosphere (using a balloon). The catalyst was then filtered off using a Celite® pad, washed with EtOAc (10 mL) and evaporated *in vacuo*. The resulting residue was purified by column chromatography over silica and eluted with hexane:EtOAc (4:1) to afford (–)-(*S*)-*N*-*tert*-butoxycarbonyl-3-(3-hydroxyphenyl)-piperidine 58% yield (440 mg, 1.59 mmol).

Enantiomeric excess of 94% was determined by HPLC [Chiralpak® IC; flow: 1.0 mL/min; hexane/*i*-PrOH: 95: 5; λ = 210 nm; minor enantiomer t_R = 7.3 min; major enantiomer t_R = 8.9 min].

^{1}H NMR (400 MHz, Chloroform-*d*) δ 1.48 (s, 9H), 1.58 (m, 2H), 1.74 (m, 1H), 2.00 (m, 1H), 2.61 (m, 1H), 2.72 (br s, 2H), 3.99 – 4.30 (m, 2H), 6.02 (s, 1H), 6.71 (m, 2H), 6.76 (d, *J* = 7.7 Hz, 1H), 7.14 (t, *J* = 8.1 Hz, 1H).

^{13}C NMR (101 MHz, Chloroform-*d*) δ 25.5, 28.5 (3C), 31.6, 42.4, 44.7, 50.5, 79.8, 113.6, 114.3, 119.1, 129.6, 145.1, 155.1, 156.1.

IR (ATR) ν_{max} /cm^{-1} = 3325 (s), 2976 (s), 2934 (s), 2858 (s), 1660 (l), 1601 (m), 1588 (m), 1477 (m), 1432 (m), 1393 (s), 1367 (m), 1268 (m), 1159 (l), 1028 (s), 997 (s), 953 (s), 862 (s), 816 (s), 784 (s), 764 (s), 700 (s).

HRMS (ESI): *m/z* calc. for $C_{16}H_{23}O_3NNa$ $[M+Na]^+$: 300.1570, found: 300.1572.

$[\alpha]^{25}_{589}$ = –55.4° (*c* 1.00, $CHCl_3$).

(+)-(*S*)-3-(3-Hydroxyphenyl)-piperidine hydrochloride (82)

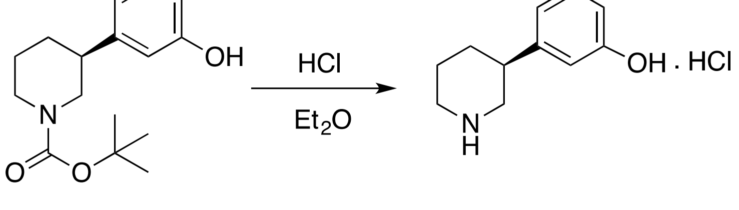

A 1M solution of HCl in Et_2O (6.3 mL, 6.3 mmol, 10.0 eq) was added dropwise to a solution of (*S*)-*N*-*tert*-Butoxycarbonyl-3-(3-hydroxyphenyl)-piperidine (175 mg, 0.63 mmol, 1.00 eq) in Et_2O (5 mL) under argon and the mixture was stirred 16 h at room temperature. The resulting precipitate was then filtered, washed with Et_2O and concentrated *in vacuo* to afford (+)-(*S*)-3-(3-hfydroxyphenyl)-piperidine hydrochloride in 68% yield (91 mg, 0.43 mmol).

^{1}**H NMR** (400 MHz, DMSO-d_6) δ 1.65 (m, 1H), 1.83 (m, 3H), 2.78 – 3.02 (m, 3H), 3.24 (m, 2H), 6.68 (m, 3H), 7.05 – 7.21 (m, 1H), 9.13 (s, 2H), 9.48 (s, 1H).

^{13}**C NMR** (101 MHz, DMSO-d_6) δ 22.7, 29.9, 39.6, 43.3, 48.5, 114.4 (2C), 117.9, 130.0, 143.7, 158.1.

IR (ATR) v_{max} /cm^{-1} = 3202 (s), 2951 (s), 2806 (s), 2388 (s), 2348 (s), 2180 (s), 2116 (s), 1615 (s), 1586 (m), 1492 (s), 1471 (s), 1446 (s), 1328 (s), 1283 (s), 1261 (s), 1215 (s), 1167 (s), 1066 (s), 1038 (s), 978 (s), 942 (s), 914 (s), 882 (s), 859 (s), 789 (l), 759 (s), 699 (l), 673 (s), 640 (s).

HRMS (ESI): *m/z* calc. for $C_{11}H_{17}ON$ $[M+H]^+$: 178.1226, found: 178.1225.

[α]^{25}$_{589}$ = +6.6° (*c* 1.00, DMSO).

(–)-(*S*)-N-Propyl-3-(3-hydroxyphenyl)-piperidine, ((–)-preclamol)

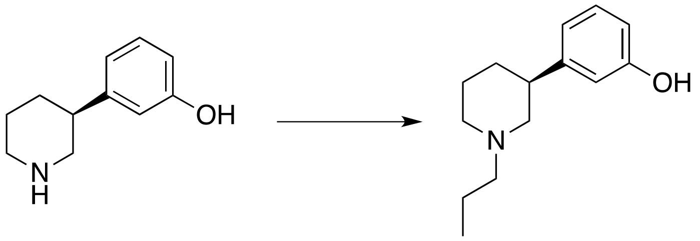

Freshly distilled propanal (33.2 µL, 0.46 mmol, 2.00 eq), $NaBH_3CN$ (0.25 mL, 0.25 mmol, 1.09 eq) and acetic acid (6.9 µL, 0.12 mmol, 0.50 eq) were sequentially added to a solution of (*S*)-3-(3-hydroxyphenyl)-piperidine **82** (50 mg, 0.23 mmol, 1.00 eq) in MeOH (2 mL). The reaction mixture was stirred for 16 h at room temperature. Water (5 mL) was then added and the resulting mixture was extracted with EtOAc (2x5 mL). The combined organic phases were dried over $MgSO_4$, filtered and concentrated *in vacuo*. The resulting residue was purified by flash column chromatography over silica and eluted with CH_2Cl_2:MeOH:Et_3N (90:9:1) to afford (–)-preclamol in 98% yield (51.6 mg, 0.23 mmol).

^{1}**H NMR** (400 MHz, Chloroform-*d*) δ 0.87 (t, *J* = 7.3 Hz, 3H), 1.43 – 1.65 (m, 3H), 1.71 – 1.87 (m, 2H), 2.00 (m, 3H), 2.38 (m, 2H), 2.94 (tt, *J* = 11.8, 3.5 Hz, 1H), 3.08 (d, *J* = 12.0, 1H), 3.23 (dt, *J* = 11.4, 3.7, 1H), 6.68 – 6.75 (m, 2H), 6.78 (m, 1H), 7.18 (t, *J* = 7.9 Hz, 1H).

^{13}**C NMR** (101 MHz, Chloroform-*d*) δ 12.1, 19.2, 25.2, 29.9, 41.8, 54.0, 61.2, 61.4, 114.3, 114.7, 117.4, 129.8, 145.3, 157.0.

IR (ATR) v_{max} /cm^{-1} = 3045 (s), 2934 (l), 2876 (l), 2813 (m), 2363 (s), 1737 (s), 1589 (m), 1456 (m), 1378 (s), 1283 (m), 1180 (s), 1135 (s), 1081 (s), 1031 (s), 993 (s), 959 (s), 860 (s), 784 (m), 699 (m).

HRMS (ESI): *m/z* calc. for $C_{14}H_{22}ON$ $[M+H]^+$: 220.1696; found: 220.1695.

[α]^{25}$_{589}$ = –11.1° (*c* 1.00, $CHCl_3$).[lit. [α]^{22}$_{589}$ = –8.9° (*c* 1.20, $CHCl_3$)].[6]

(+)-(*S*)-*N-tert*-butoxycarbonyl-3-(pyridin-2-yl)-piperidine

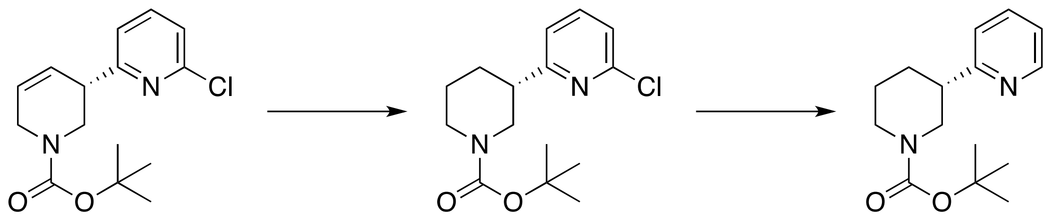

(+)-(*S*)-*N*-*tert*-Butoxycarbonyl-5-(2-chloro-6-pyridinyl)-3-piperidene **70** (44.3 mg, 0.15 mmol, 1.00 eq) was dissolved in toluene (2 mL) and $(PPh_3)_3RhCl$ (25 mg, 0.03 mmol, 0.18 eq) was added. The reaction mixture was stirred overnight at room temperature under H_2 atmosphere (using a balloon). The catalyst was then filtered off using a silica plug and washed with EtOAc (5 mL). The solvent was evaporated *in vacuo* to afford (+)-(*S*)-*N*-*tert*-butoxycarbonyl-3-(2-chloro-6-pyridinyl)-piperidine (quant.) which was used in the next step without further purification. For an analytical purposes an aliquot of the crude was purified by column chromatography over silica and eluted with petrol ether:EtOAc (8:2).

The crude was diluted in MeOH (4 mL) and $NaHCO_3$ (12.6 mg, 0.15 mmol, 1.00 eq) and Pd/C (10% on carbon, 3.2 mg, 3.0 µmol, 0.02 eq) were added. The resulting mixture was stirred overnight at room temperature under H_2 (using a balloon). The solids were removed by filtration through Celite®. The solvent was then evaporated to afford crude (+)-(*S*)-*N*-*tert*-butoxycarbonyl-3-(pyridin-2-yl)-piperidine. For an analytical purposes an aliquot of the crude was purified by column chromatography over silica and eluted with petrol ether:EtOAc (8:2).

Analytical data for (+)-(*S*)-*N-tert*-Butoxycarbonyl-3-(2-chloro-6-pyridinyl)-piperidine

^{1}H-NMR (400 MHz, $CDCl_3$) δ 1.45 (s, 9 H), 1.50 – 1.62 (m, 1 H), 1.64 – 1.88 (m, 2 H), 1.94 – 2.06 (m, 1 H), 2.72 – 2.87 (m, 2 H), 2.88 – 3.16 (m, 1 H), 3.91 – 4.33 (m, 2 H), 7.08 (d, *J* = 7.6 Hz, 1 H), 7.15 (d, *J* = 7.9 Hz, 1 H), 7.56 (t, *J* = 7.8, 7.8 Hz, 1 H).

^{13}C-NMR (100 MHz, $CDCl_3$) δ 25.1, 28.6 (3C), 30.7, 44.0 (2C), 48.8, 79.6, 120.5, 122.3, 139.1, 151.1, 154.9, 163.9.

IR (ATR) ν_{max} /cm^{-1} = 2934s, 1689l, 1583s, 1558s, 1415l, 1365sm, 1260m, 1160l, 1145l, 795s.

HRMS (ESI): *m*/*z* calc. for $C_{15}H_{22}ClN_2O_2$ $[M+H]^+$: 297.1364, found: 297.1362.

$[\alpha]^{25}_{589}$ = +73.6° (*c* 1.00, $CHCl_3$) measured for 89% ee.

Analytical data for (+)-(*S*)-*N-tert*-butoxycarbonyl-3-(2-pyridinyl)-piperidine

HPLC analysis indicated an enantiomeric excess of 95% [Chiralpak® IC; flow: 1.0 mL/min; hexane/*i*-PrOH 80:20; λ = 210 nm; major enantiomer t_R = 10.0 min; minor enantiomer t_R = 11.5 min].

^{1}H-NMR (400 MHz, $CDCl_3$) δ 1.45 (s, 9 H), 1.51 – 1.67 (m, 1 H), 1.68 – 1.86 (m, 2 H), 1.98 – 2.10 (m, 1 H), 2.66 – 2.88 (m, 2 H, H-3), 2.89 – 3.16 (m, 1 H), 3.86 – 4.48 (m, 2 H), 7.12 (ddd, *J* = 7.5, 4.9, 1.2 Hz, 1 H), 7.16 (ddd, *J* = 7.8, 1.1, 1.1 Hz, 1 H), 7.60 (ddd, *J* = 7.7, 7.7, 1.9 Hz, 1 H), 8.53 (ddd, *J* = 4.9, 1.9, 0.9 Hz, 1 H).

^{13}C-NMR (100 MHz, $CDCl_3$) δ 25.3, 28.6 (3C), 30.9 (C-6), 44.2, 44.4, 49.2, 79.5, 121.7, 122.2, 136.6, 149.4, 155.0, 162.8.

IR (ATR) ν_{max} /cm^{-1} = 2933s, 1688l, 1590s, 1472s, 1418m, 1365s, 1259m, 1172m, 1144m, 768s.

HRMS (ESI): *m/z* calc. for $C_{15}H_{23}N_2O_2$ $[M+H]^+$: 263.1754, found: 263.1754.

$[\alpha]^{25}_{589}$ = +58.4° (*c* 1.00, $CHCl_3$) measured for 89% ee.

(+)-(*S*)-3-(Pyridin-2-yl)-piperidine, (+)-(*S*)-isoanabasine

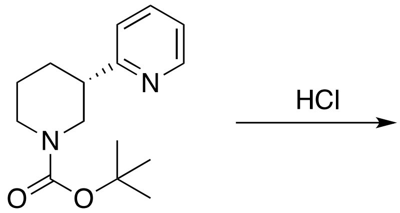

HCl (4 N in dioxane, 0.4 mL, 1.6 mmol, 10.0 eq) was added dropwise to a solution of crude (+)-(*S*)-*N*-*tert*-butoxycarbonyl-3-(pyridin-2-yl)-piperidene (0.15 mmol, 1.00 eq) in dioxane (2 mL). The reaction mixture was stirred for 20 h at room temperature. The solvent was then evaporated and the crude was dissolved in NaOH (1 M, aq., 5 mL). The mixture was extracted with CH_2Cl_2 (3 × 3 mL), washed with brine (2 mL), dried over $MgSO_4$, filtered and evaporated *in vacuo*. The resulting residue was purified by column chromatography over silica and eluted with CH_2Cl_2:MeOH:Et_3N (90:9:1) to afford (+)-(*S*)-isoanabasine in 38% yield (over 3 steps, 9.2 mg, 0.06 mmol). The spectroscopic data matches the reported values in the literature.[7]

^{1}H-NMR (400 MHz, $CDCl_3$) δ 1.50 – 1.66 (m, 1 H), 1.66 – 1.83 (m, 2 H), 1.97 – 2.06 (m, 1 H), 2.66 (ddd, *J* = 12.3, 12.0, 2.4 Hz, 1 H), 2.75 – 2.91 (m, 2 H), 3.07 (ddd, *J* = 12.6, 3.1, 3.1 Hz, 1 H), 3.15 – 3.30 (m, 1 H), 7.08 (dd, *J* = 7.4, 5.0 Hz, 1 H), 7.12 (d, *J* = 7.9 Hz, 1 H), 7.57 (dd, *J* = 7.7, 7.7 Hz, 1 H), 8.50 (d, *J* = 4.9 Hz, 1 H).

^{13}C-NMR (100 MHz, $CDCl_3$) δ 26.6, 30.9, 45.8, 46.5, 52.2, 121.5, 121.8, 136.5, 149.2, 163.8.

LRMS (ESI): *m/z* calc. for $C_{10}H_{15}N_2$ $[M+H]^+$: 163.1, found: 163.1.

$[\alpha]^{25}_{589}$ = +11.1° (*c* 1.00, $CHCl_3$, 95% ee); (Lit: $[\alpha]^{20}_{589}$ +15.2° (*c* 1.0, ethanol)).

(–)-(*S*)-*tert*-Butyl-3-(4-aminophenyl)piperidine-1-carboxylate (83)

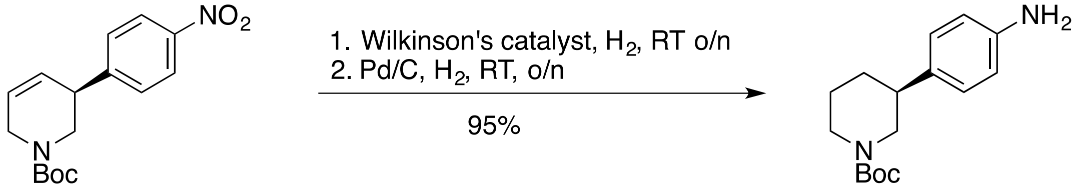

84 (121.7 mg, 0.40 mmol, 1.00 eq) was dissolved in toluene (2 mL) and $(PPh_3)_3RhCl$ (66.6 mg, 0.07 mmol, 0.18 eq) was added. The reaction mixture was stirred overnight at room temperature under H_2 atmosphere (using a balloon). The catalyst was then filtered off using

a silica plug and washed with dichloromethane (5 mL). The solvent was then evaporated and the resulting solid (116.4 mg, 0.38 mmol) was used in the next step without further purification.
The above obtained crude (50.1 mg, 0.16 mmol) was then diluted in MeOH (1 mL) and Pd/C was added (5 mg, 10% wt). The resulting mixture was stirred overnight at room temperature under H_2 (using a balloon). The solids were removed by filtration through Celite®. The solvent was then evaporated and the title compound was obtained as an off-white solid in quantitative yield (44.0 mg, 0.16 mmol) and 94% ee. The spectroscopic data matches the reported values in the literature.[8]

^{1}H NMR (400 MHz, $CDCl_3$) δ 7.02 (d, *J* = 8.4 Hz, 2H), 6.65 (d, *J* = 8.3 Hz, 2H), 4.37 – 3.90 (m, 2H), 3.62 (s, 2H), 2.80 – 2.48 (m, 3H), 2.03 – 1.91 (m, 1H), 1.81 – 1.67 (m, 1H), 1.62 – 1.51 (m, 3H), 1.46 (s, 9H).

^{13}C NMR (101 MHz, $CDCl_3$) δ 153.9, 143.8, 132.7, 126.9 (2C), 114.2 (2C), 78.3, 50.1, 42.8, 40.7, 30.9, 27.5(3C), 24.6.

IR (ν_{max}/cm^{-1}): 3461, 3357, 2973, 2932, 1683, 1540.

HRMS (CI) *m/z* calc. for $C_{16}H_{26}N_2O_2$ $[M+H]^+$: 277.1916, found: 277.1910.

$[\alpha]^{25}_{589}$ = –48.8° (c 0.82, $CHCl_3$) for 95% ee.

(–)-(*R*,*E*)-*N*-*tert*-Butoxycarbonyl-5-(4-nitrophenyl)-3-piperidene: *large scale* (84)

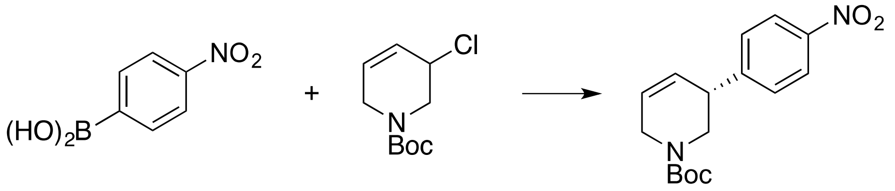

In a 250 mL round bottomed flask $[Rh(cod)(OH)]_2$ (102.6 mg, 0.23 mmol, 0.018 eq), (*S*)-(+)-5,5′-Dichloro-2,2′-bis(diphenylphosphino)-6,6′-dimethoxy-1,1′-biphenyl (351.8 mg, 0.54 mmol, 0.04 eq) and Cs_2CO_3 (5.86 g, 18.0 mmol, 1.44 eq) were stirred in THF (90 mL) at 60 °C for 30 min. A solution of *N*-*tert*-butoxycarbonyl-5-chloro-3-piperidene (2.74 g, 12.5 mmol, 1.00 eq) in THF (35 mL) was then added *via* syringe and the flask rinsed with THF (10 mL) followed by a second solution of 4-nitrophenylboronic acid (6.00 g, 36.0 mmol, 2.00 eq) in 35 mL of THF, which was also added *via* syringe and the flask rinsed with THF (10 mL). The flask containing the mixture was then sealed with para film and the reaction was stirred for 16 h at 80 °C before filtering off the solids and adding SiO_2 (400 mg). The solvent was then carefully evaporated and the solid directly loaded into a chromatographic column. The column was eluted with hexane:EtOAc (9:1) to obtain the pure product in 90% yield (3.40 g, 11.3 mmol) as a colorless oil.

Enantiomeric excess of 94% was determined by HPLC [Chiralpak® IA; flow: 1.30 mL/min; hexane/i-PrOH: 95: 5; λ = 210 nm; minor enantiomer t_R = 7.9 min; major enantiomer t_R = 9.2 min].

^{1}H NMR (400 MHz, $CDCl_3$) δ 8.15 (d, *J* = 8.2 Hz, 2H), 7.37 (d, *J* = 8.0 Hz, 2H), 5.99 and 5.95 – 5.82 (m, rotameric, 2H), 4.11 – 3.94 (m, rotameric, 3H), 3.67 – 3.52 (m) and 3.26 (s, rotameric, 2H), 1.43 (s, 9H).

^{13}**C NMR** (101 MHz, $CDCl_3$) δ 154.5, 149.9, 146.9, 128.8, 127.3 (2C), 126.4, 123.7, 79.9, 47.9, 46.6, 43.6 and 43.0 (1C, rotameric), 41.4, 28.3 (3C).

IR (v_{max}/cm^{-1}): 2976, 2929, 1696, 1420.

HRMS (CI) *m/z* calc. for $C_{16}H_{20}N_2O_4$ [M+Na]$^+$: 327.1315, found: 327.1317.

[α]^{25} $_{589}$ = –147.3° (c 0.42, $CHCl_3$) for 94% ee.

(–)-(*S*)-*tert*-Butyl-3-(4-bromophenyl)-3,6-dihydropyridine-1(2*H*)-carboxylate (85)

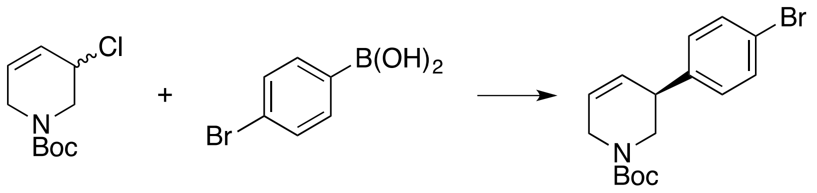

In a 5 mL round bottomed flask $[Rh(cod)(OH)]_2$ (4.6 mg, 0.01 mmol, 0.025 eq), (*S*)-(+)-5,5′-dichloro-2,2′-bis(diphenylphosphino)-6,6′-dimethoxy-1,1′-biphenyl (15.6 mg, 0.024 mmol, 0.06 eq) and Cs_2CO_3 (130.3 mg, 0.40 mmol, 1.00 eq) were stirred in THF (2 mL) at 60 °C for 30 min. A solution of *N*-*tert*-butoxycarbonyl-5-chloro-3-piperidene (87 mg, 0.40 mmol, 1.00 eq) in THF (1.5 mL) was then added *via* syringe and the flask rinsed with THF (0.5 mL) followed by a second solution of 4-bromophenylboronic acid (160.7 mg, 0.80 mmol, 2.00 eq) in 1.5 mL of THF, which was also added *via* syringe and the flask rinsed with THF (0.5 mL). The resulting mixture was then stirred for 16 h at 80 °C before the addition of SiO_2 (20 mg). The solvent was then carefully evaporated and the solid directly loaded into a chromatographic column. Elution with hexane:EtOAc (9:1) afforded the pure product in 97% yield (131.0 mg, 0.39 mmol) as a colorless oil.

Enantiomeric excess of 95% was determined by HPLC [Chiralpak® IC; flow: 1.0 mL/min; hexane/i-PrOH: 99:1; λ = 210 nm; minor enantiomer t_R = 11.0 min; major enantiomer t_R = 12.2 min].

^{1}**H NMR** (500 MHz, $CDCl_3$) δ 7.41 (d, *J* = 8.3 Hz, 2H), 7.11 – 7.05 (m, 2H), 5.97 – 5.78 (m, 2H), 4.20 –3.60 (m, 3H), 3.50–3.44 (m, 1H), 1.48 – 1.19 (m, 10H).

^{13}**C NMR** (126 MHz, $CDCl_3$) δ 154.8, 141.3, 131.6 and 131.2 (rotameric, 2C), 129.7 (2C), 128.7 and 128.6 (rotameric, 1C), 127.6 and 127.5 (rotameric, 1C), 126.5 and 125.6 (rotameric, 1C), 120.7, 79.7, 53.6, 48.4 and 47.1 (rotameric, 1C), 43.7 and 43.0 (rotameric, 1C), 41.1, 28.5–28.0 (rotameric, 3C).

IR (v_{max}/cm^{-1}): 2976, 2929, 1696, 1420.

HRMS (CI) *m/z* calc. for $C_{16}H_{20}BrO_2$ [M+H]$^+$: 338.0705, found: 338.0706.

[α]^{25} $_{589}$ = –107.6° (c 0.93, $CHCl_3$) for 95% ee.

tert-Butyl (_S_)-3-(4-bromophenyl)piperidine-1-carboxylate (86)

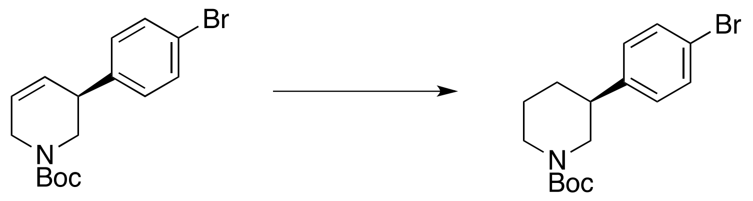

85 (47.5 mg, 0.14 mmol) was dissolved in toluene (0.7 mL). Wilkinson's catalyst (22.0 mg, 0.02 mmol, 0.18 eq) was then added and the system was purged with H_2. The mixture was stirred overnight at room temperature under H_2 atmosphere. The solvent was evaporated and the crude was purified by flash column chromatography eluting with hexane:EtOAc (9:1) affording the title compound in 99% yield (45.9 mg, 0.13 mmol) and 95% ee. The spectroscopic data is in accordance with the values reported in the literature.[9]

Enantiomeric excess of 95% was determined by HPLC [Chiralpak® IC; flow: 1.0 mL/min; hexane/i-PrOH: 99:1; λ = 210 nm; major enantiomer t_R = 12.2 min; minor enantiomer t_R = 14.0 min].

^{1}H NMR (400 MHz, $CDCl_3$) δ 7.42 (d, *J* = 8.8 Hz, 2H), 7.16 (d, *J* = 8.4 Hz, 2H), 4.13 (s, 2H), 2.75 – 2.58 (m, 3H), 2.04 – 1.91 (m, 1H), 1.80 – 1.69 (m, 1H), 1.63 – 1.46 (m, 2H), 1.46 (s, 9H).

^{13}C NMR (101 MHz, $CDCl_3$) δ 154.9, 142.6, 131.7 (2C), 129.0 (2C), 120.4, 79.7, 50.9, 42.2, 31.8, 31.7, 28.6 (3C), 25.5.

IR ($ν_{max}$/cm^{-1}): 2975, 2932, 1691, 1420.

MS (ES) *m/z* calc. for $C_{16}H_{22}BrNNaO_2$ $[M]^+$: 364.0, found: 364.0.

$[α]^{25}_{589}$ = –46.1° (c 1.64, $CHCl_3$) for 95% ee. [Lit. $[α]^{25}_{589}$ = –62.4° (c 0.37, $CHCl_3$) for 99.3% ee].

2-(4-Bromophenyl)-_N_-(_tert_-butyl)-2_H_-indazole-7-carboxamide (87)

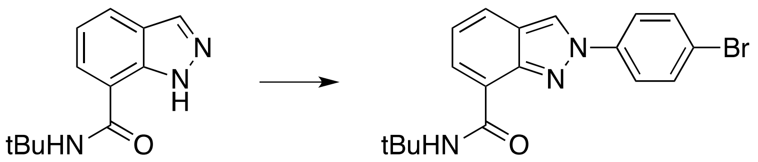

p-Dibromobenzene (1.09 g, 4.60 mmol, 2.00 eq) was dissolved in dimethylacetamide (3.8 mL) and *N*-(*tert*-butyl)-1*H*-indazole-7-carboxamide (500 mg, 2.30 mmol) and K_2CO_3 (0.95 g, 6.90 mmol, 3.00 eq) were then added under Ar. The mixture was degassed by a N_2 stream over 1 h. Then CuBr (33.0 mg, 0.23 mmol, 0.10 eq) was added followed by 8-hydroxyquinoline (67 mg, 0.46 mmol, 0.20 eq). N_2 was streamed for 30 more minutes. The resulting mixture was stirred at 110 °C under Ar for 20 h. After cooling to 40 °C, Celite® (14.5 g) was added, and the mixture aged for 1 h before being filtered, washing the cake with DMAc (1 × 100 mL). Filtration, washing with 2:1 v/ v DMAc/water (1 × 10 mL) followed by

water (1 × 15 mL), and drying *in vacuo* at 20−25 °C under a N_2 sweep afforded **87** in 61% yield (522 mg, 0.14 mmol) as an off yellow solid.

^{1}H NMR (400 MHz, $CDCl_3$) δ 9.24 (s, 1H), 8.53 (s, 1H), 8.30 (dd, *J* = 7.0, 1.1 Hz, 1H), 7.86 (dd, *J* = 8.4, 1.1 Hz, 1H), 7.83 – 7.78 (m, 2H), 7.76 – 7.70 (m, 2H), 7.28 (dd, *J* = 8.4, 7.0 Hz, 1H), 1.61 (s, 9H).

^{13}C NMR (101 MHz, $CDCl_3$) δ 164.0, 147.0, 139.0, 132.9 (2C), 130.3, 124.1, 123.8, 123.1, 123.0, 122.0, 122.0 (2C), 121.3, 51.4, 29.1 (3C).

IR (ν_{max}/cm^{-1}): 3383, 1650.

HRMS (CI) *m/z* calc. for $C_{18}H_{19}BrN_3O$ $[M+H]^+$: 372.0711, found: 372.0707.

_N_-(_tert_-Butyl)-2-(4-(4,4,5,5-tetramethyl-1,3,2-dioxaborolan-2-yl)phenyl)-2_H_-indazole-7-carboxamide (88)

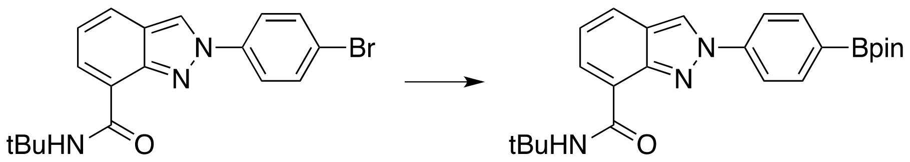

Pd(dppf)Cl_2 (70.2 mg, 0.10 mmol, 0.10 eq) was dissolved in DMF (8 mL). To that solution were sequentially added $B_2(pin)_2$ (1.00 g, 3.96 mmol, 4.13 eq), KOAc (288.3 mg, 2.94 mmol, 3.06 eq) and 2-(4-Bromophenyl)-*N*-(*tert*-butyl)-2*H*-indazole-7-carboxamide **87** (357.1 mg, 0.96 mmol, 1.00 eq). The resulting mixture was stirred at 80 °C for 2 h and then it was diluted with EtOAc. The organic phase was extracted with water and brine. Then it was dried over $MgSO_4$, filtered and concentrated under reduced pressure. The crude was purified by flash column chromatography eluting with hexane:EtOAc (7:3) affording the title compound in 90% yield (362.3 mg, 0.86 mmol).

^{1}H NMR (500 MHz, $CDCl_3$) δ 9.35 (s, 1H), 8.58 (s, 1H), 8.27 (dd, *J* = 7.0, 1.1 Hz, 1H), 8.04 – 7.97 (m, 2H), 7.95 – 7.88 (m, 2H), 7.84 (dd, *J* = 8.4, 1.1 Hz, 1H), 7.25 (dd, *J* = 8.4, 7.0 Hz, 1H), 1.58 (s, 9H), 1.38 (s, 12H).

^{13}C NMR (126 MHz, $CDCl_3$) δ 164.1, 146.9, 141.9, 136.3 (2C), 130.1, 124.0, 123.6, 122.9, 122.7, 121.2, 120.5, 119.5 (2C), 84.2 (2C), 51.3, 29.0 (3C), 24.9 (4C).

HRMS (CI) *m/z* calc. for $C_{24}H_{31}BN_3O_3$ $[M+H]^+$: 420.2458, found: 420.2450.

(_S_)-_tert_-Butyl-3-(4-(7-(_tert_-butylcarbamoyl)-2_H_-indazol-2-yl)phenyl)-3,6-dihydropyridine-1(2_H_)-carboxylate (89)

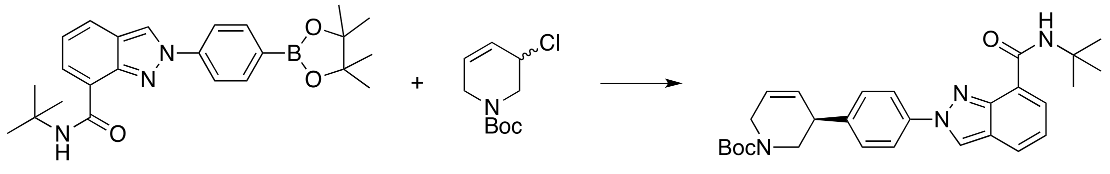

In a 5 mL round bottomed flask $[Rh(cod)(OH)]_2$ (2.3 mg, 0.005 mmol, 0.025 eq), (*S*)-(+)-5,5′-Dichloro-2,2′-bis(diphenylphosphino)-6,6′-dimethoxy-1,1′-biphenyl **88** (7.8 mg, 0.012 mmol,

0.06 eq) and Cs_2CO_3 (65.2 mg, 0.20 mmol, 1.00 eq) were stirred in THF (1 mL) at 60 °C for 30 min. A solution of *N*-*tert*-butoxycarbonyl-5-chloro-3-piperidene (43.5 mg, 0.20 mmol, 1.00 eq) in THF (0.5 mL) was then added *via* syringe and the flask rinsed with THF (0.5 mL) followed by a second solution of *N*-(*tert*-Butyl)-2-(4-(4,4,5,5-tetramethyl-1,3,2-dioxaborolan-2-yl)phenyl)-2*H*-indazole-7-carboxamide (167.7 mg, 0.40 mmol, 2.00 eq) in 0.5 mL of THF, which was also added *via* syringe and the flask rinsed with THF (0.5 mL). The resulting mixture was then stirred for 16 h at 80 °C before the addition of SiO_2 (20 mg). The solvent was then carefully evaporated and the solid directly loaded into a chromatographic column. The column was eluted with hexane:EtOAc (7:3) to obtain the pure product in 94% yield (89.6 mg, 0.19 mmol) as a yellow oil.

Enantiomeric excess of 98% was determined by HPLC [Chiralpak® ID; flow: 1.20 mL/min; hexane/i-PrOH: 80:20; λ = 210 nm; minor enantiomer t_R = 18.6 min; major enantiomer t_R = 21.0 min].

^{1}H NMR (500 MHz, $CDCl_3$) δ 9.28 and 9.26 (br. s, rotameric, 1H), 8.51 and 8.43 (s, rotameric, 1H), 8.19 (dd, *J* = 7.0, 1.0 Hz, 1H), 7.93 and 7.83 (m, rotameric, 1H), 7.76 (d, *J* = 8.6 Hz, 2H), 7.34 (d, *J* = 8.7 Hz, 1H), 7.17 (dd, *J* = 8.4, 7.0 Hz, 1H), 6.01 – 5.80 (m, rotameric, 2H), 4.28 – 3.80 (m, rotameric, 2H), 3.54 (m, rotameric, 2H), 1.90 (br. s, 2H), 1.51 (s, 9H), 1.17 (s, 9H).

^{13}C NMR (126 MHz, $CDCl_3$) δ 164.2, 146.8, 142.7, 138.7, 136.3, 130.1 and 130.1 (rotameric), 129.9 and 129.3 (rotameric, 2C), 123.9, 123.6, 122.9 and 122.7 (rotameric), 121.1 (2C), 120.6 (2C), 119.5 and 119.5 (rotameric), 84.2, 75.0 (rotameric), 51.3, 29.0 and 29.0 (rotameric), 28.4 and 28.3 (rotameric, 3C), 24.9 and 24.9 (rotameric, 3C).

IR ($ν_{max}$/cm^{-1}): 3321, 2965, 2927, 1734, 1697, 1662, 773.

HRMS (CI) *m/z* calc. for $C_{28}H_{35}N_4O_3$ $[M+H]^+$: 475.2709, found: 475.2709.

$[α]^{25}_{589}$ = –50.6° (c 0.34, $CHCl_3$) for 98% ee.

(*S*)-*tert*-Butyl-3-(4-(7-(*tert*-butylcarbamoyl)-2*H*-indazol-2-yl)phenyl)piperidine-1-carboxylate (90)

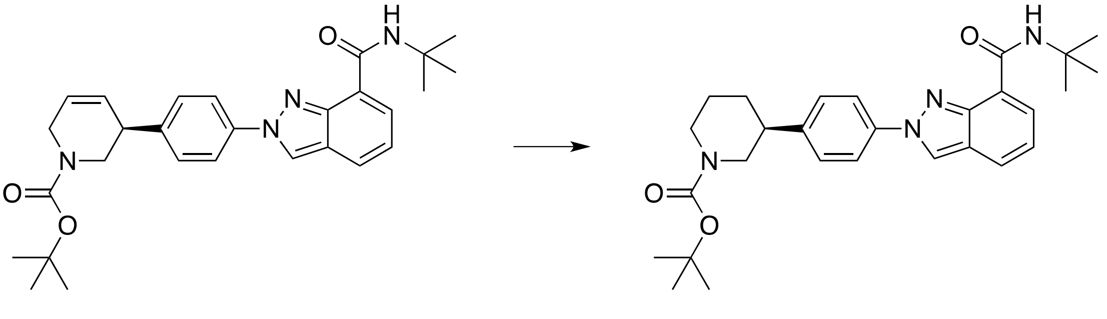

(*S*)-*tert*-Butyl-3-(4-(7-(*tert*-butylcarbamoyl)-2*H*-indazol-2-yl)phenyl)-3,6-dihydropyridine-1(2*H*)-carboxylate **89** (13.5 mg, 0.03 mmol, 1.00 eq) was dissolved in toluene (0.4 mL) and $(PPh_3)_3RhCl$ (4.7 mg, 0.005 mmol, 0.18 eq) was added and the mixture was stirred overnight at room temperature under H_2 atmosphere (using a balloon). The solvent was evaporated and the residue purified by flash column chromatography eluting with hexane:EtOAc (7:3) obtaining the title product in quantitative yield (14 mg, 0.03 mmol). The spectroscopic analysis matches the reported values in the literature.[9]

^{1}H NMR (400 MHz, MeOH-d_4) δ 9.62 (s, 1H), 9.01 (s, 1H), 8.13 (dd, *J* = 7.1, 1.1 Hz, 1H), 8.12 – 7.81 (m, 3H), 7.55 (d, *J* = 8.7 Hz, 2H), 7.28 (dd, *J* = 8.4, 7.1 Hz, 1H), 4.34 – 3.98 (m, 2H), 3.04 – 2.75 (m, 2H), 2.17 (d, *J* = 4.3 Hz, 1H), 1.82– 1.72 (m, 2H), 1.64–1.60 (m, 1H), 1.62 (s, 9H), 1.51 (s, 9H).

^{13}C NMR (101 MHz, MeOD) δ 166.6, 156.7, 148.0, 145.7, 140.0, 130.8, 129.9 (2C), 126.6, 125.4, 124.1, 123.4, 123.4, 121.9 (2C), 81.4, 52.8, 43.8, 32.9, 29.5 (3C), 28.9 (3C), 27.2.

MS (ES) *m/z* calc. for $C_{28}H_{37}N_4O_3$ $[M+H]^+$: 477.3, found: 477.3.

$[\alpha]^{25}{}_{589}$ = –57.5° (c 0.44, $CHCl_3$) for 98% ee. [lit. $[\alpha]^{25}{}_{589}$ = –62.8 (c 0.25, DMSO)].

Supplementary figures

Supplementary figure 1: Asymmetric coupling with racemic allyl halides and different ligands. In blue, results obtained using the allyl chloride; in red, results in the case of allyl bromide coupling.

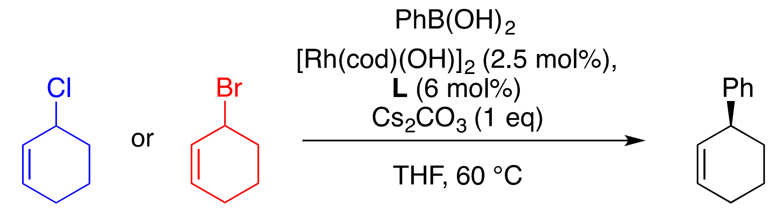

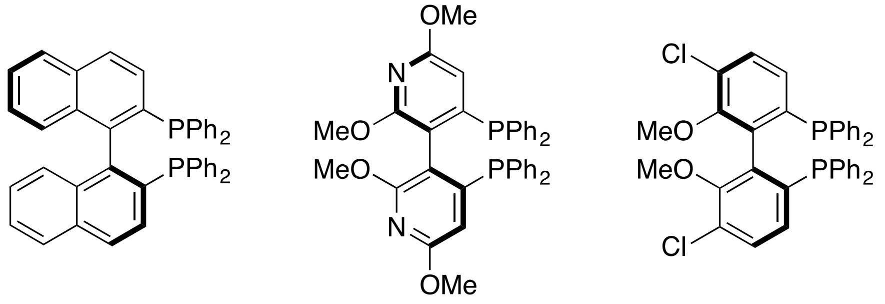

**1**	**5**	**6**
**(*S*)-BINAP**	**(*S*)-P-PHOS**	**(*S*)-Cl-MeO-BIPHEP**
**35% yield, 99% ee**	***76% yield, 81% ee***	***99% yield, 99% ee***
50% yield, 98% ee		

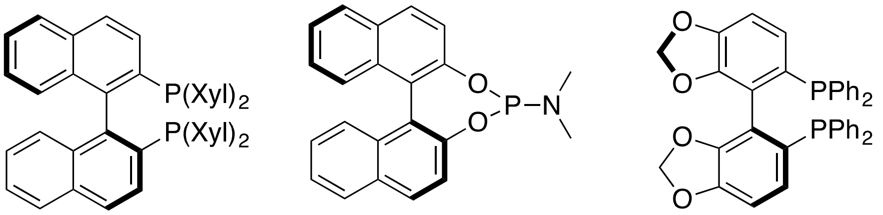

**7**	**8**	**9**
**(*S*)-Xyl-BINAP**	**(*S*)-MonoPHOS**	**(*S*)-SegPHOS**
52% yield, 99.3% ee	***7% yield, 86% ee***	
11% yield, 97% ee	***58% yield, 89% ee***	***99% yield, 98% ee***

Supplementary figure 2: ^{1}H, ^{13}C-NMR spectra, SFC traces of compound **1**

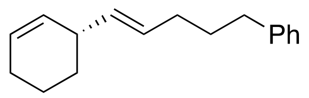

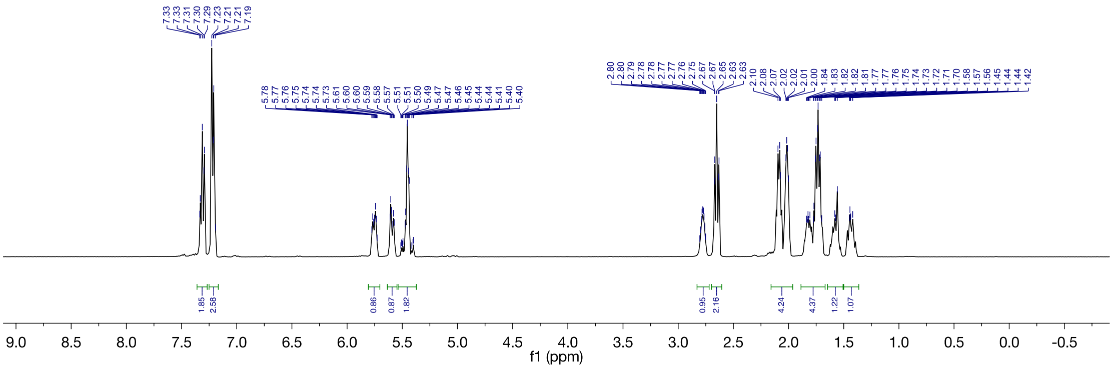

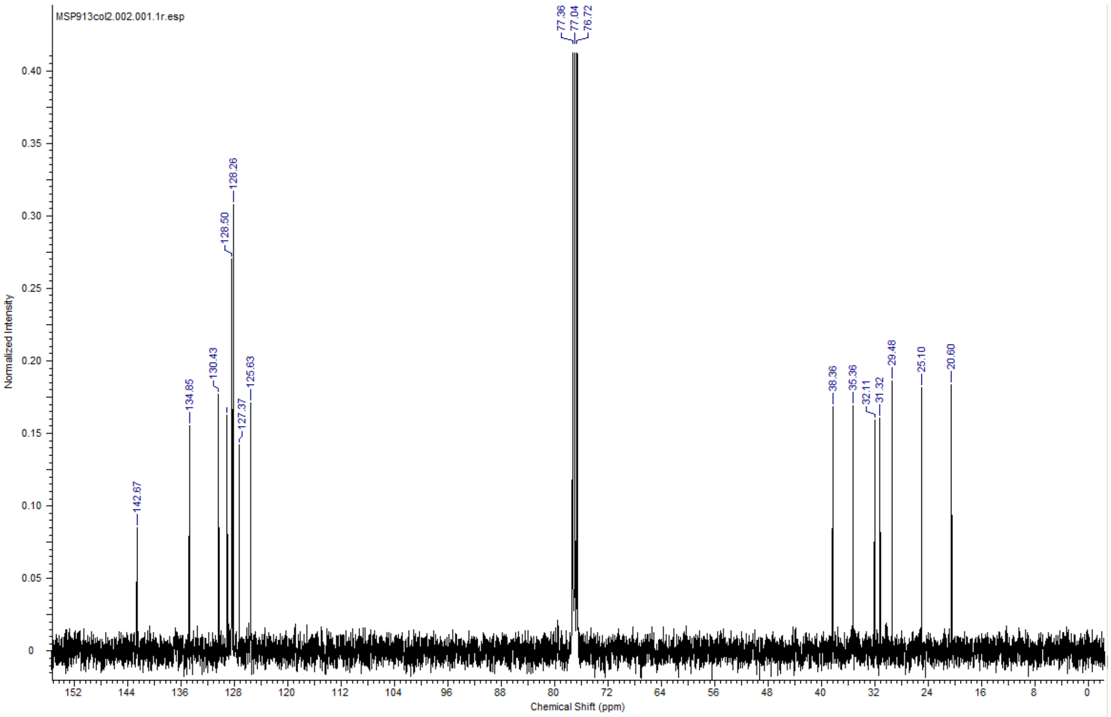

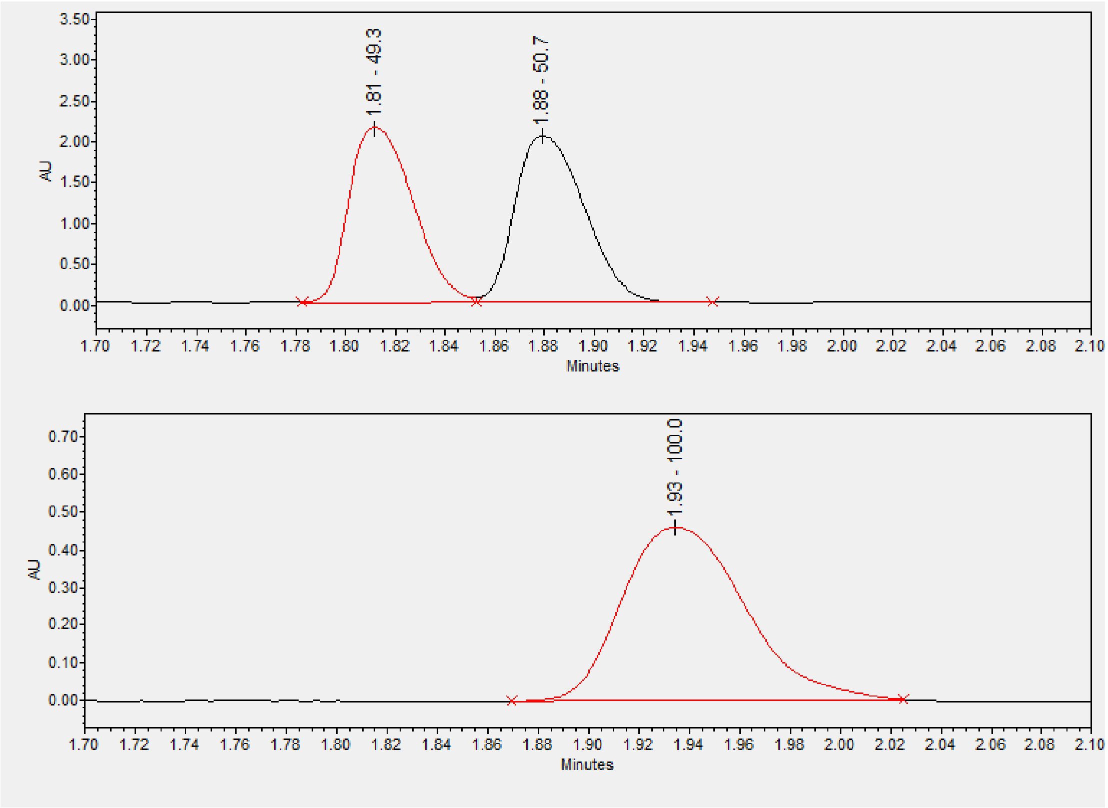

Supplementary figure 3: ^{1}H, ^{13}C-NMR spectra, GC traces of compound **2**

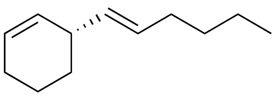

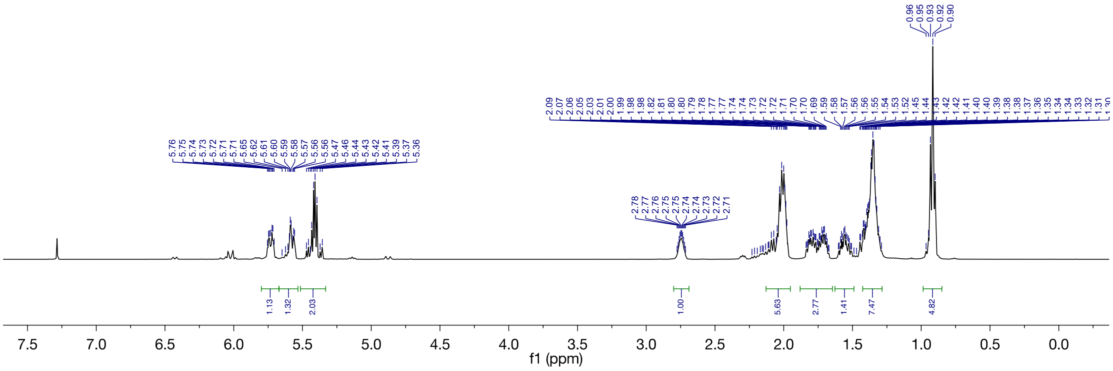

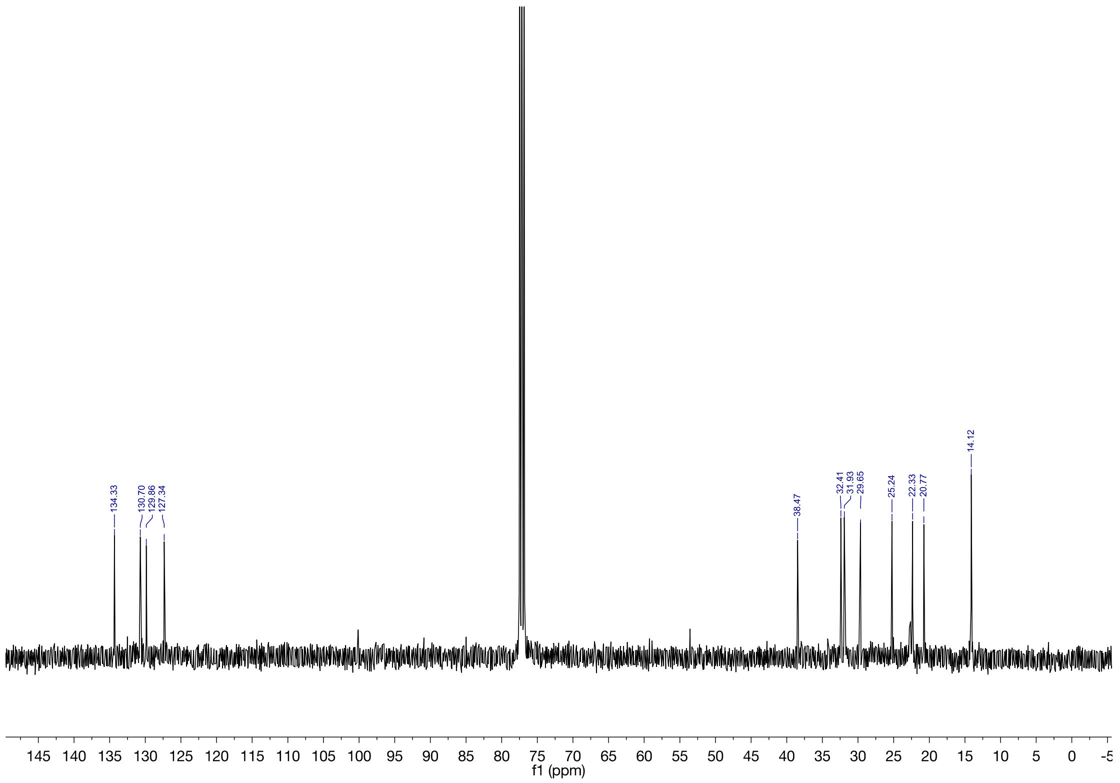
134.33
130.70
129.86
127.34
38.47
32.41
31.93
29.65
25.24
22.33
20.77
14.12
145 140 135 130 125 120 115 110 105 100 95 90 85 80 75 70 65 60 55 50 45 40 35 30 25 20 15 10 5 0 -5
f1 (ppm)

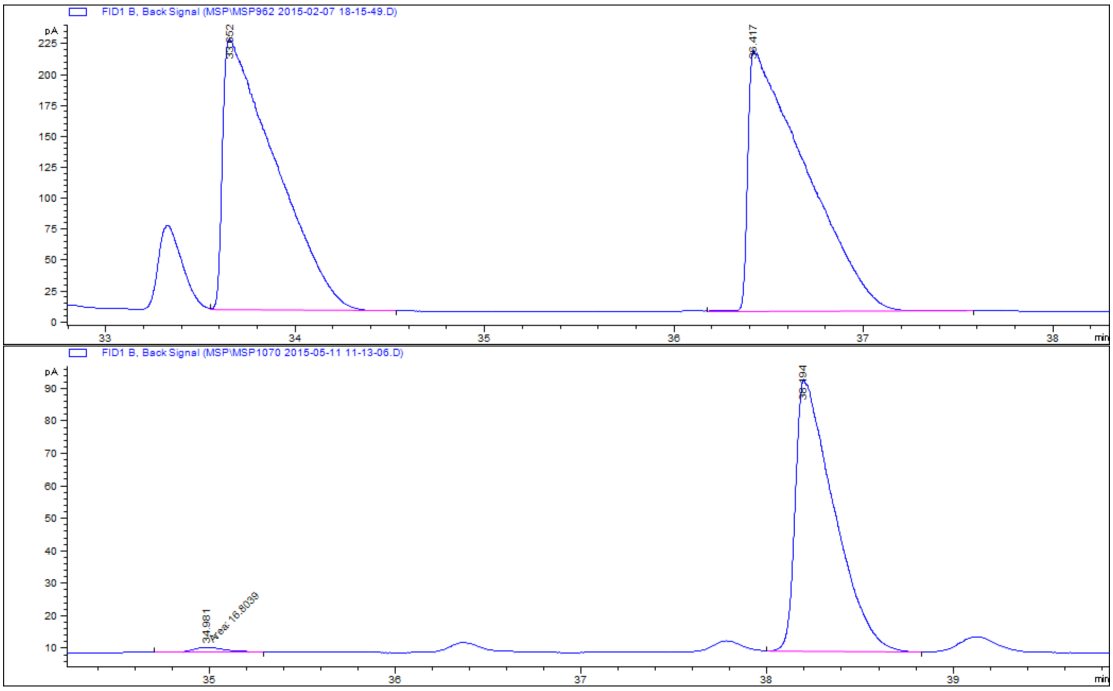

Supplementary figure 4: ^{1}H, ^{13}C-NMR spectra, SFC traces of compound **3**

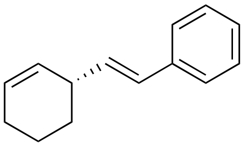

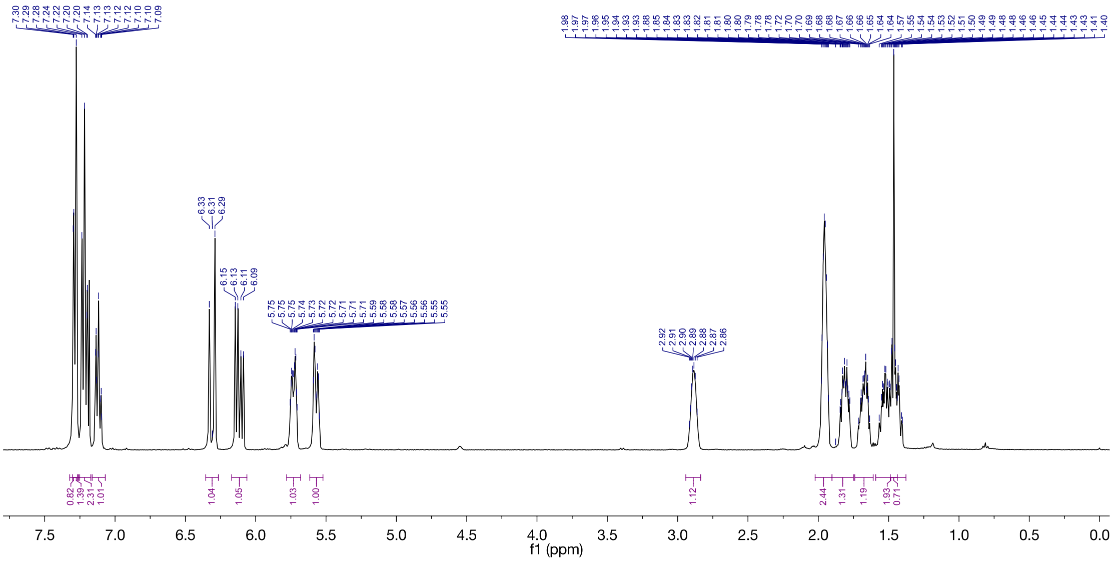

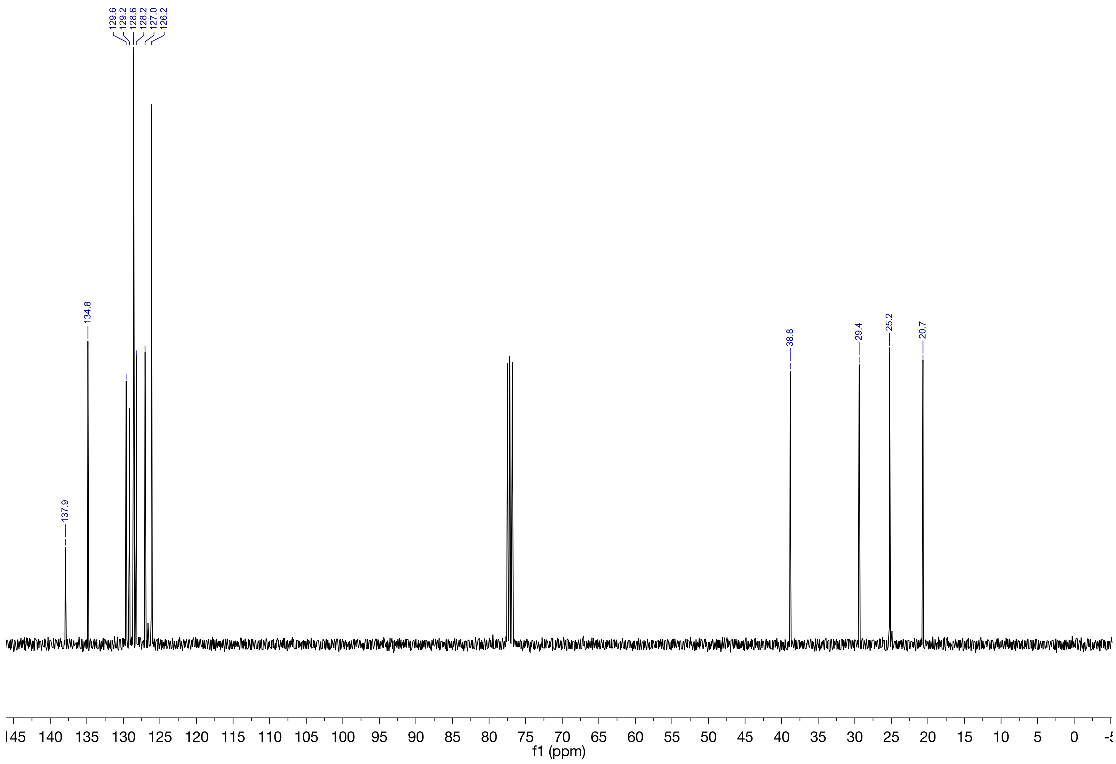
137.9
134.8
129.6
129.2
128.6
128.2
127.0
126.2
38.8
29.4
25.2
20.7
f1 (ppm)

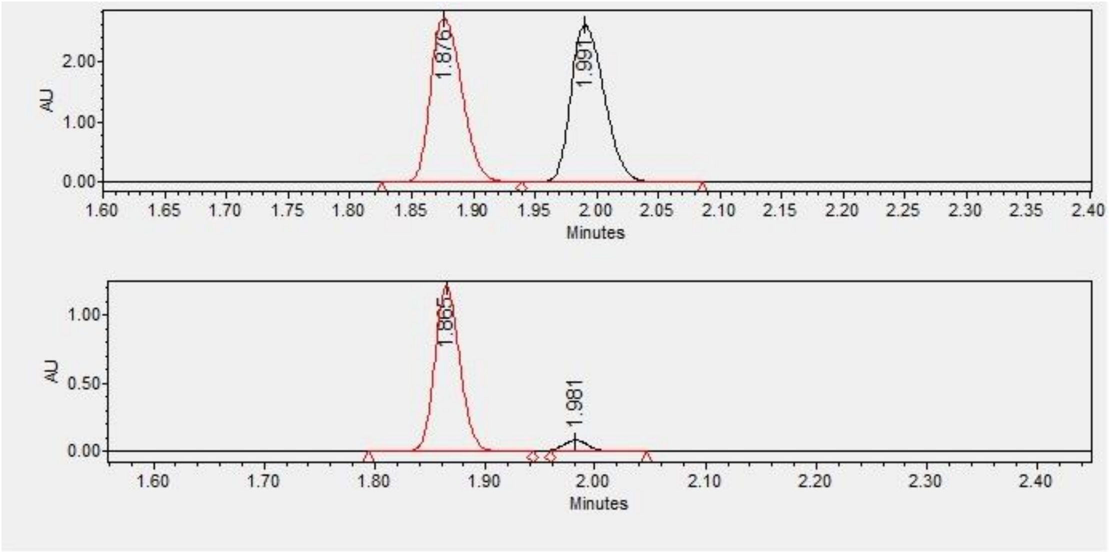
1.876
1.991
AU
Minutes
1.865
1.981
AU
Minutes

Supplementary figure 5: ^{1}H, ^{13}C-NMR spectra, SFC traces of compound **4**

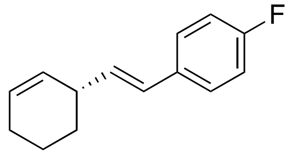

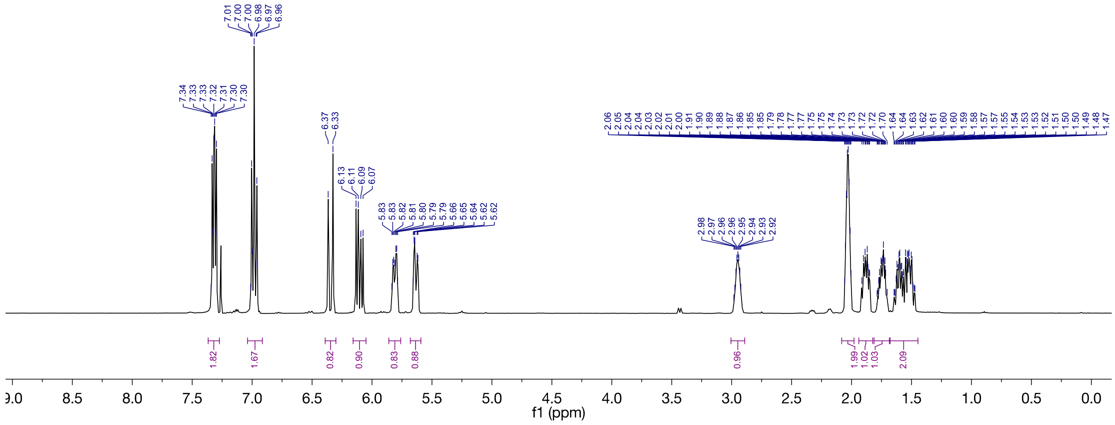

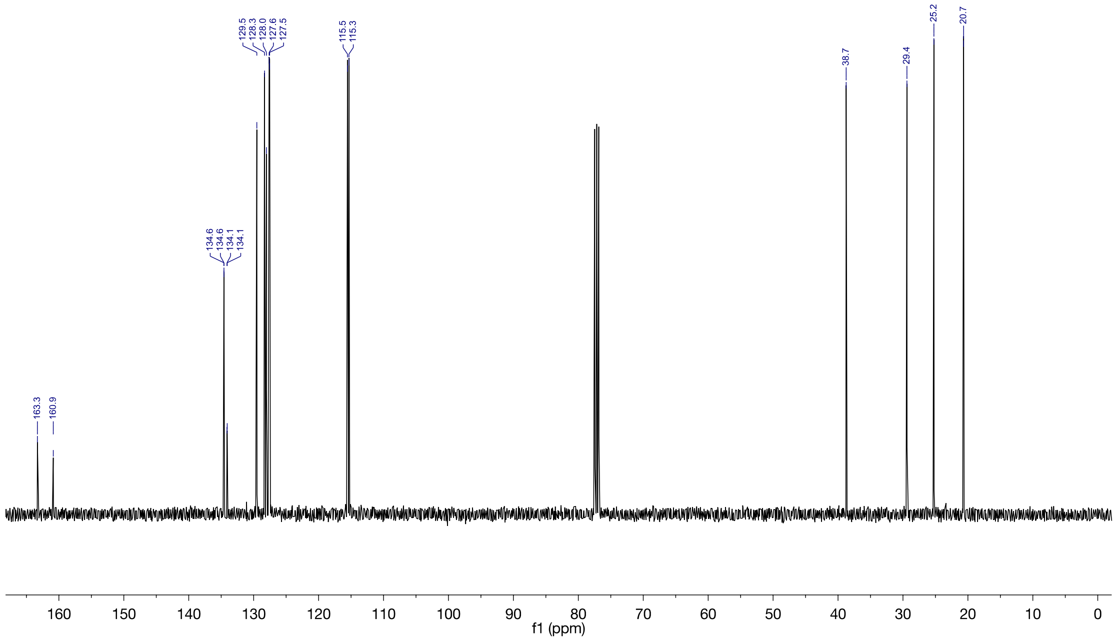

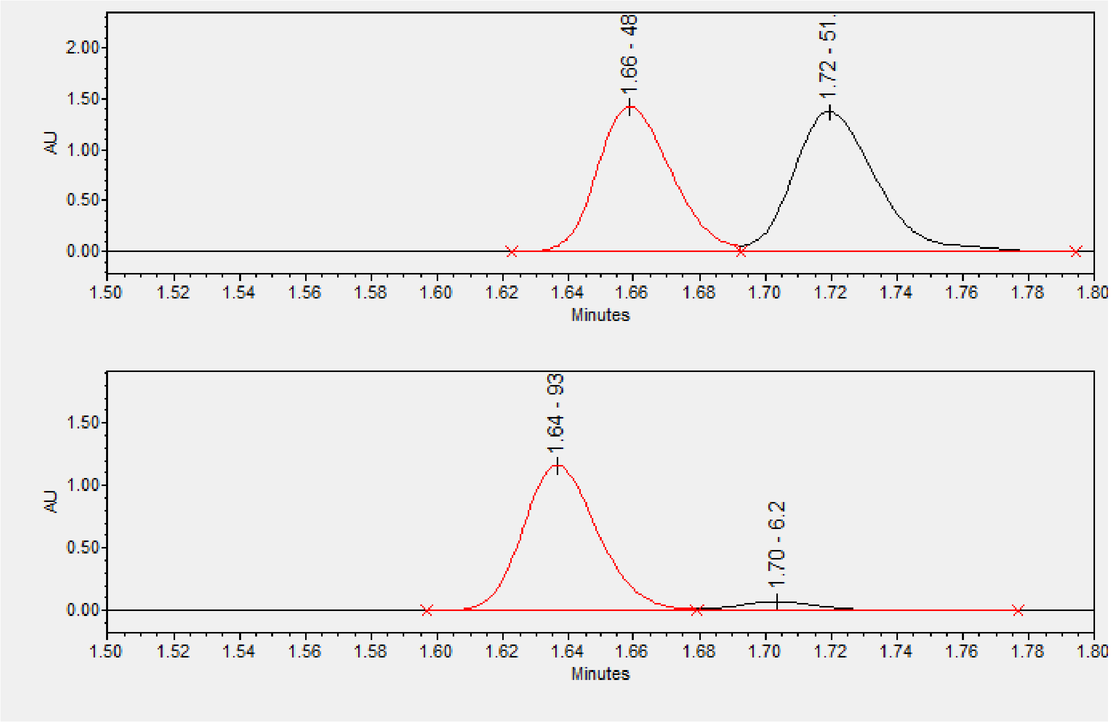

Supplementary figure 6: ^{1}H, ^{13}C-NMR spectra, SFC traces of compound **5**

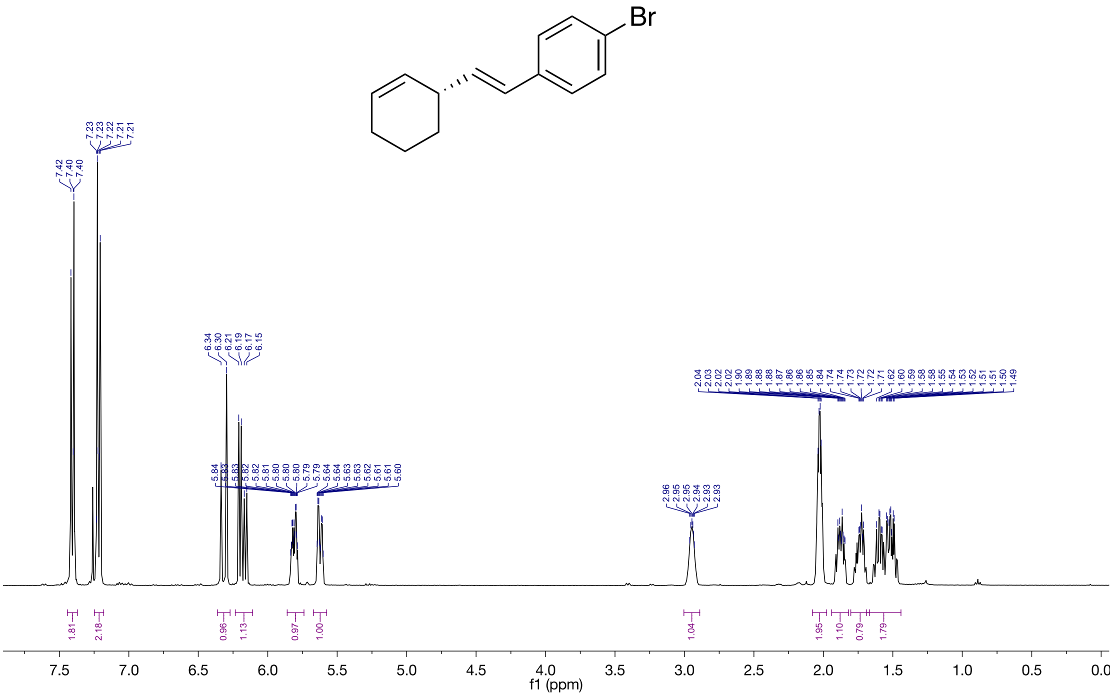

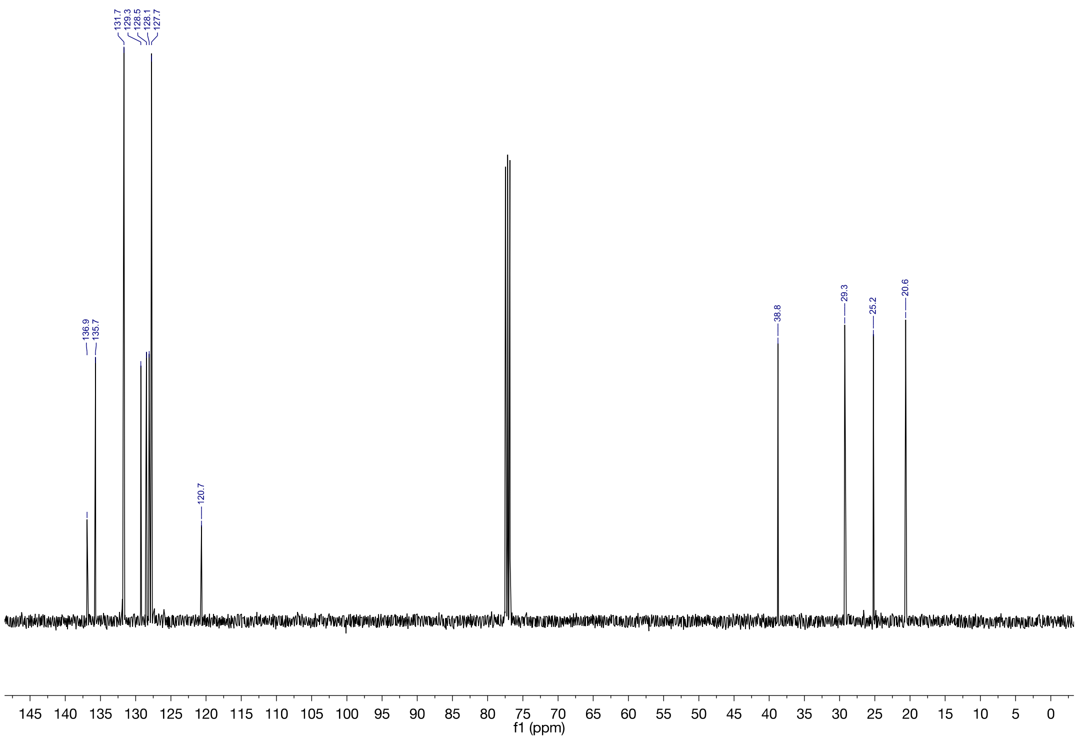

136.9
135.7
131.7
129.3
128.5
128.1
127.7
120.7
38.8
29.3
25.2
20.6
145 140 135 130 125 120 115 110 105 100 95 90 85 80 75 70 65 60 55 50 45 40 35 30 25 20 15 10 5 0
f1 (ppm)

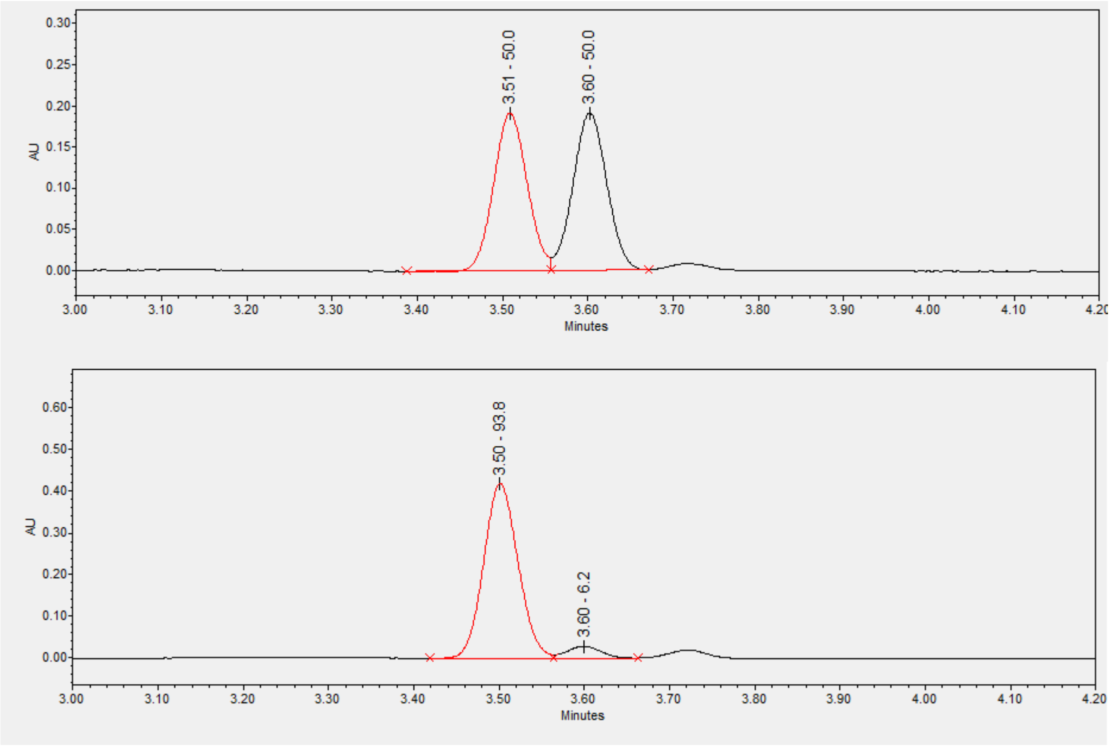

3.51 - 50.0
3.60 - 50.0
AU
Minutes
3.50 - 93.8
3.60 - 6.2
AU
Minutes

Supplementary figure 7: ^{1}H, ^{13}C-NMR spectra, SFC traces of compound **6**

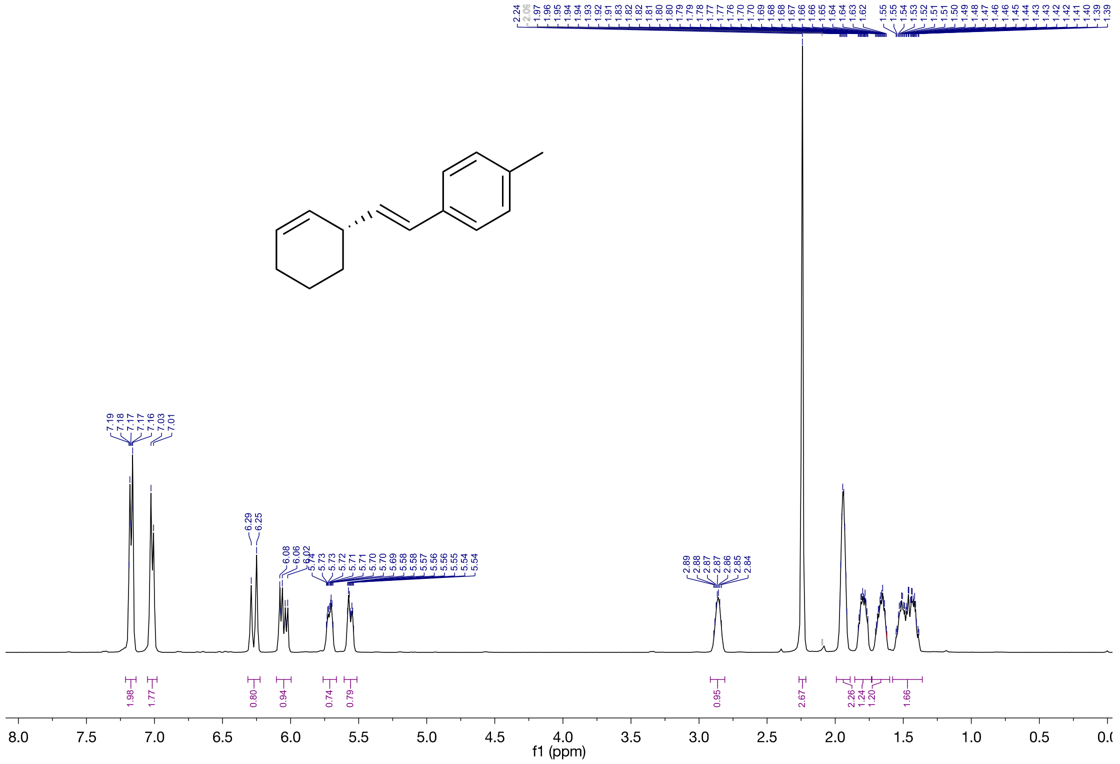

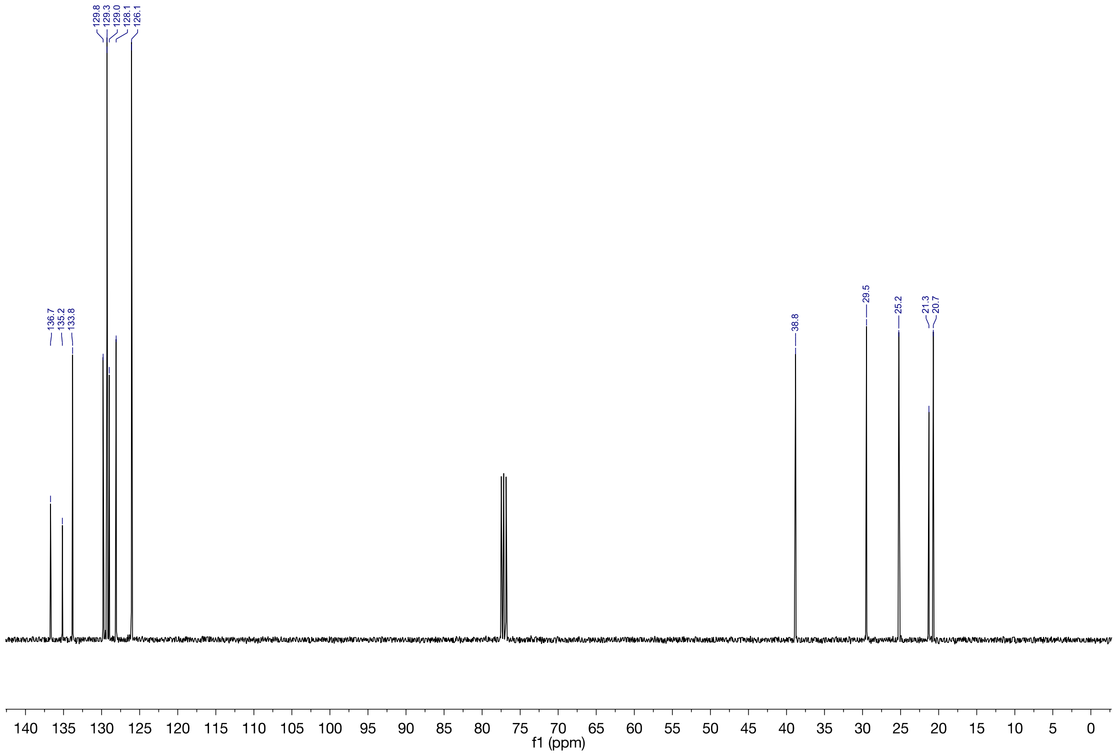

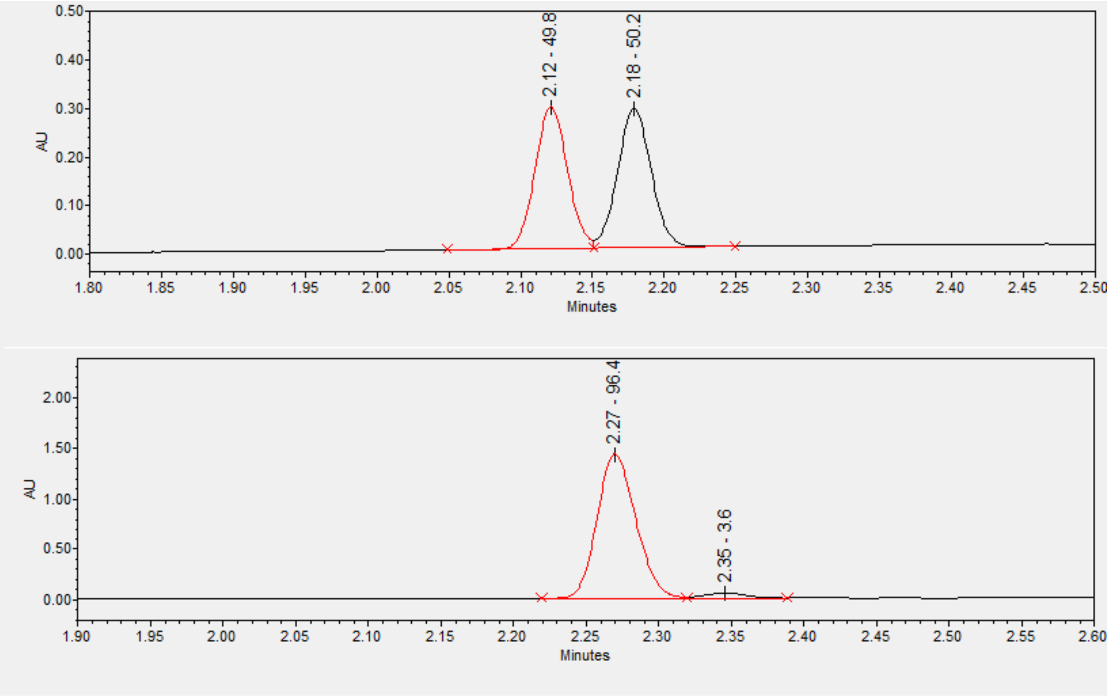

Supplementary figure 8: ^{1}H, ^{13}C-NMR spectra, SFC traces of compound **7**

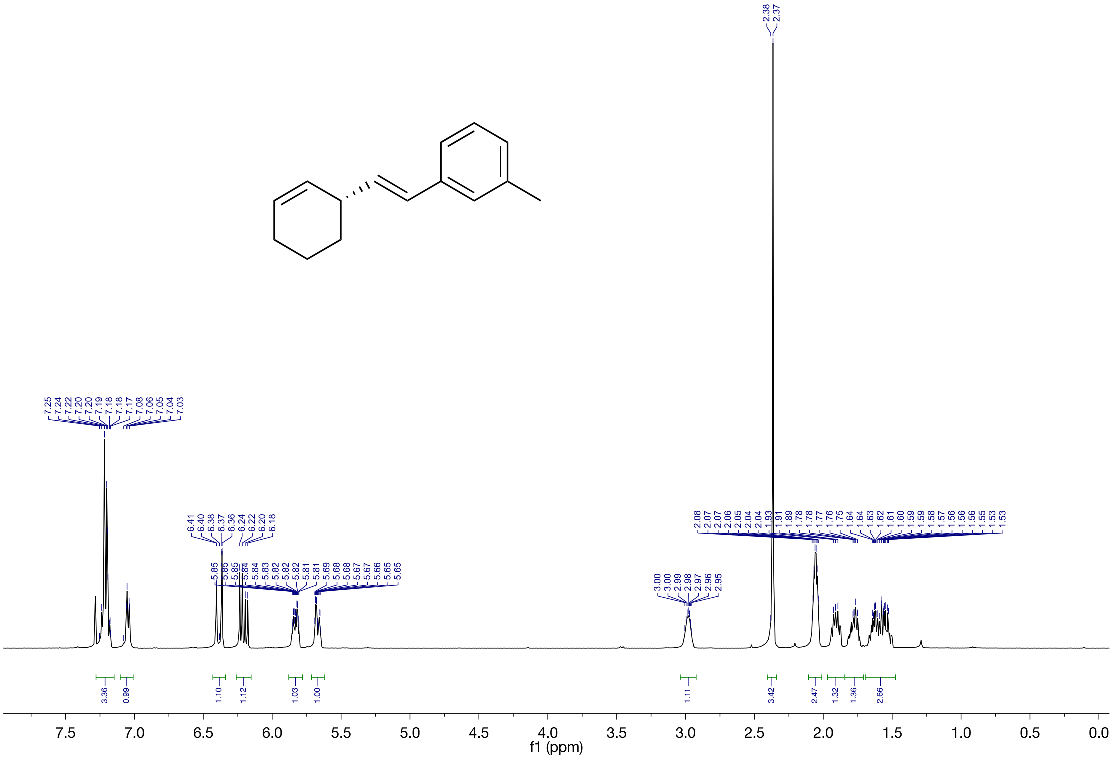

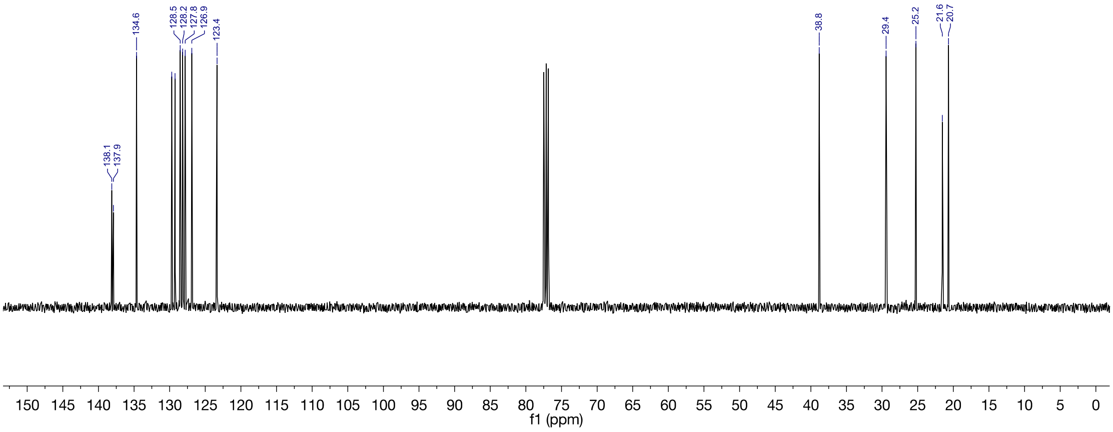
138.1
137.9
134.6
128.5
128.2
127.8
126.9
123.4
38.8
29.4
25.2
21.6
20.7
150 145 140 135 130 125 120 115 110 105 100 95 90 85 80 75 70 65 60 55 50 45 40 35 30 25 20 15 10 5 0
f1 (ppm)

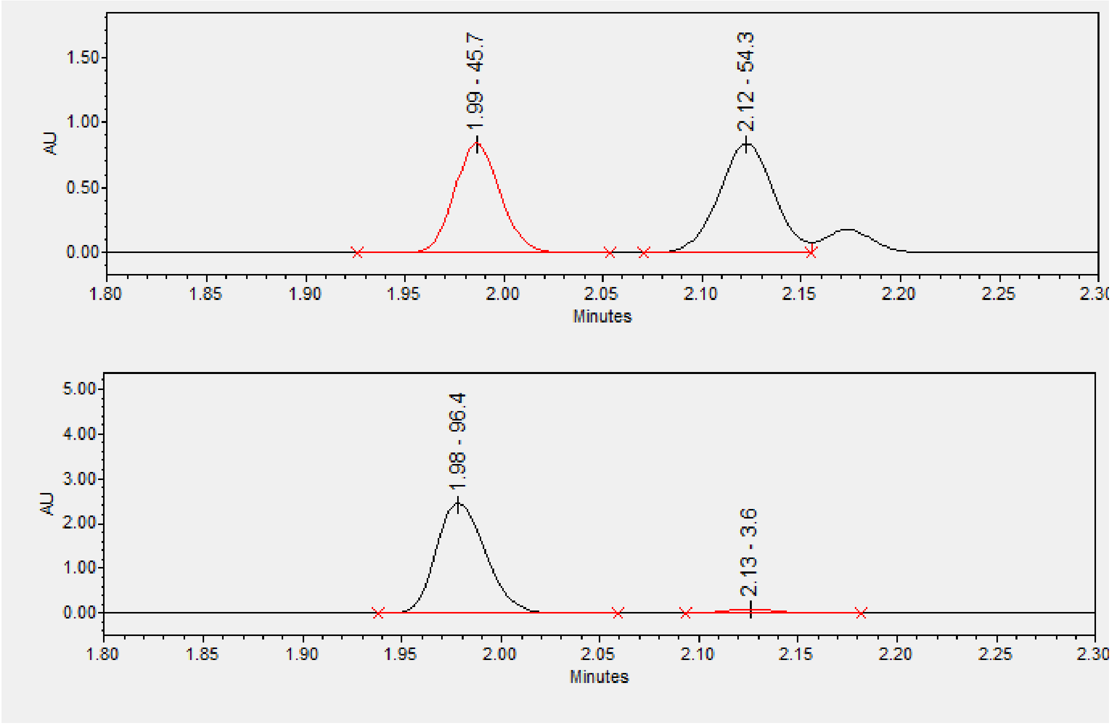
1.99 - 45.7
2.12 - 54.3
AU
Minutes
1.98 - 96.4
2.13 - 3.6
AU
Minutes

Supplementary figure 9: ^{1}H, ^{13}C-NMR spectra, HPLC traces of compound **8**

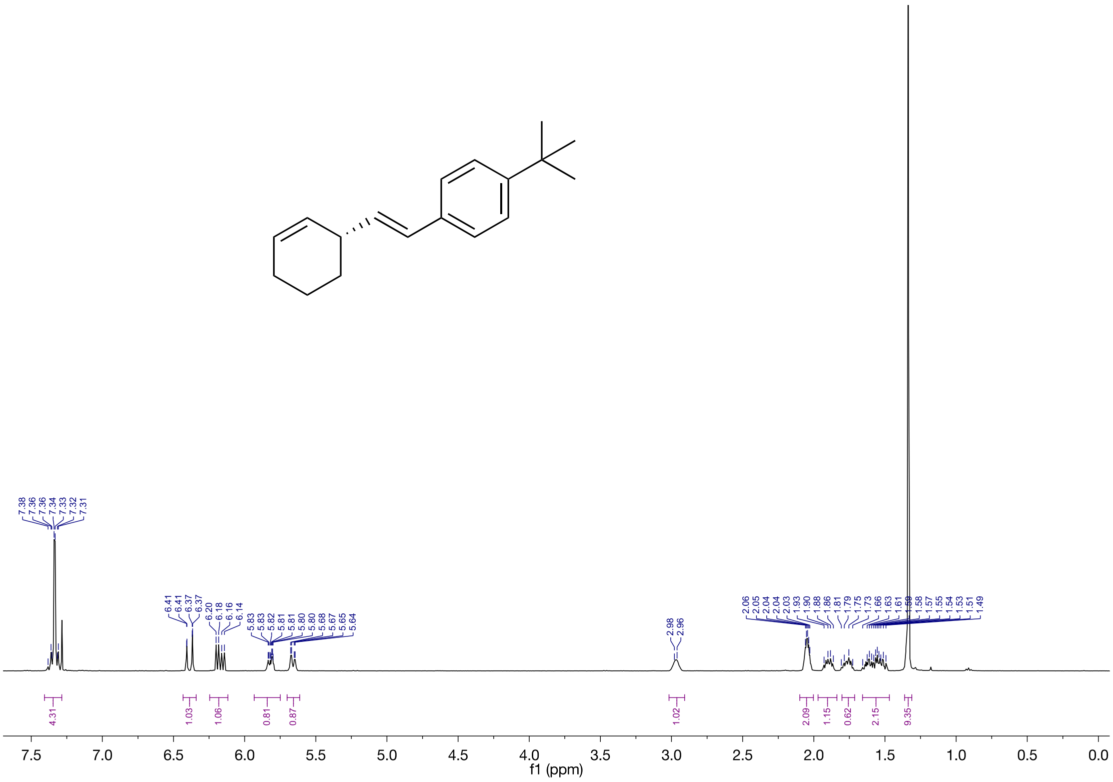

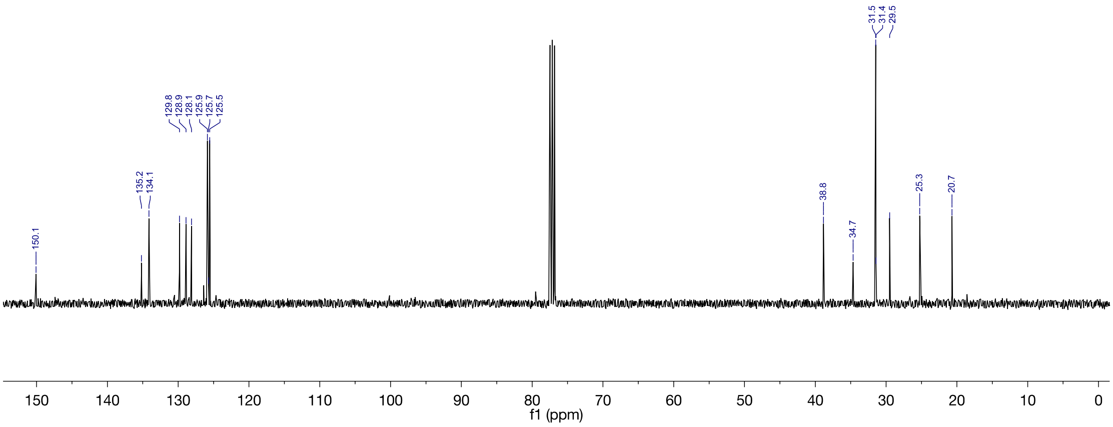

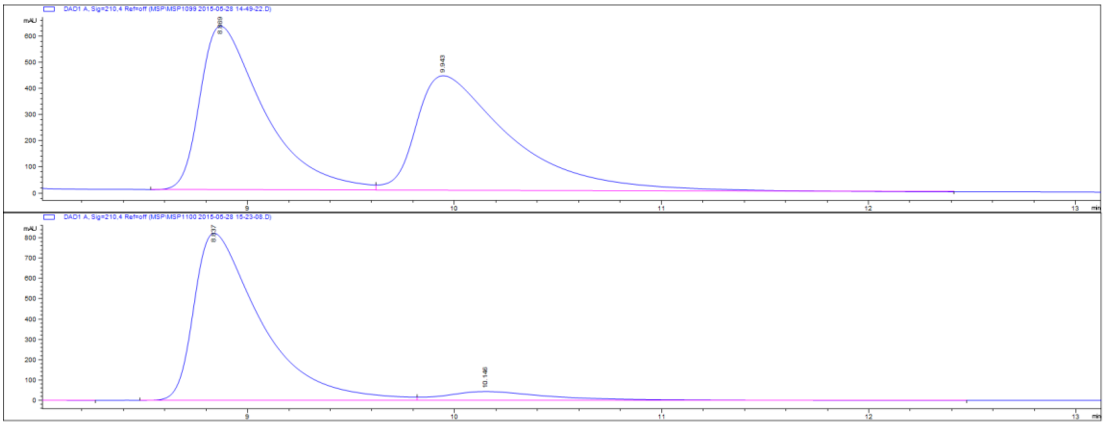

Supplementary figure 10: ^{1}H, ^{13}C-NMR spectra, SFC traces of compound **9**

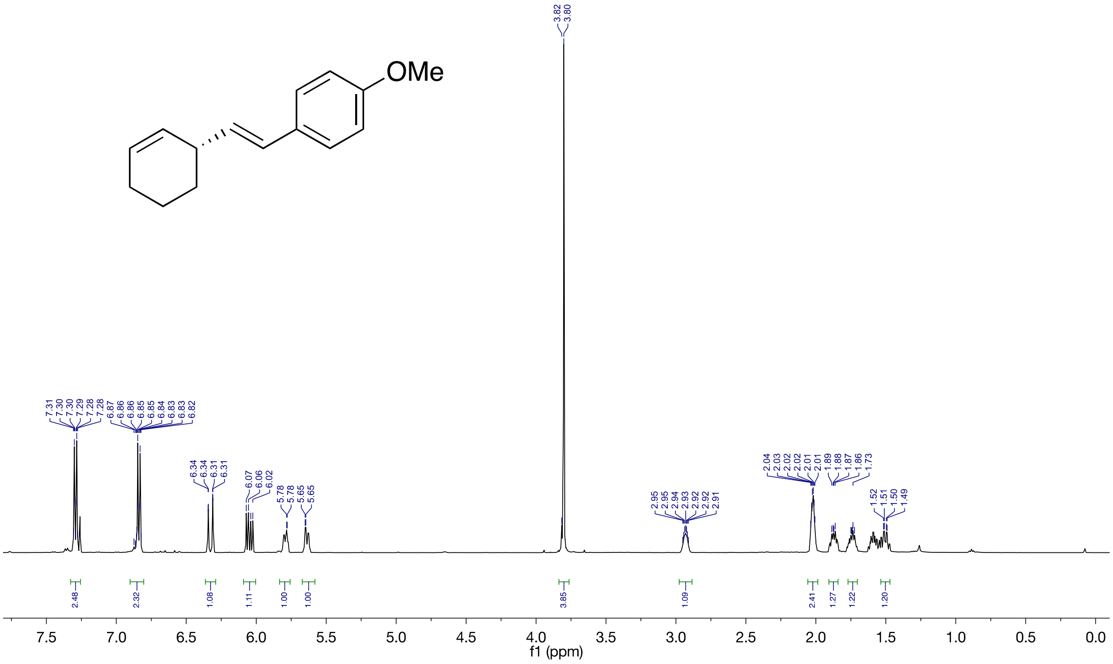

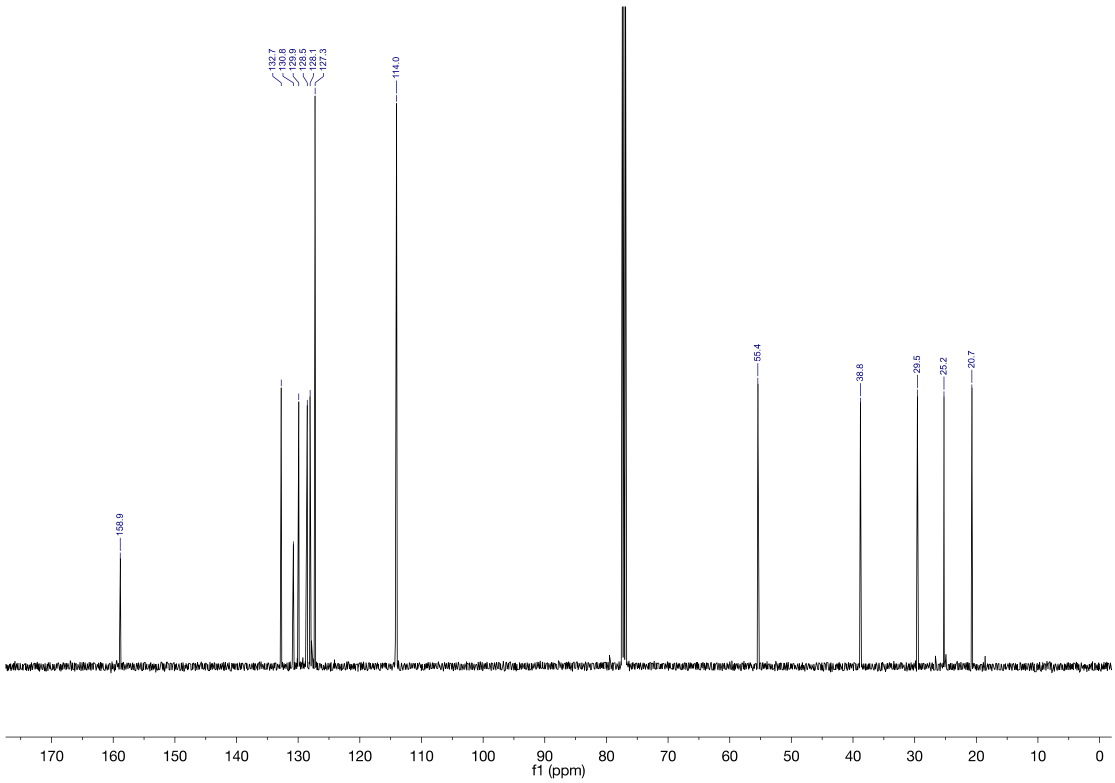

158.9
132.7
130.8
129.9
128.5
128.1
127.3
114.0
55.4
38.8
29.5
25.2
20.7
170 160 150 140 130 120 110 100 90 80 70 60 50 40 30 20 10 0
f1 (ppm)

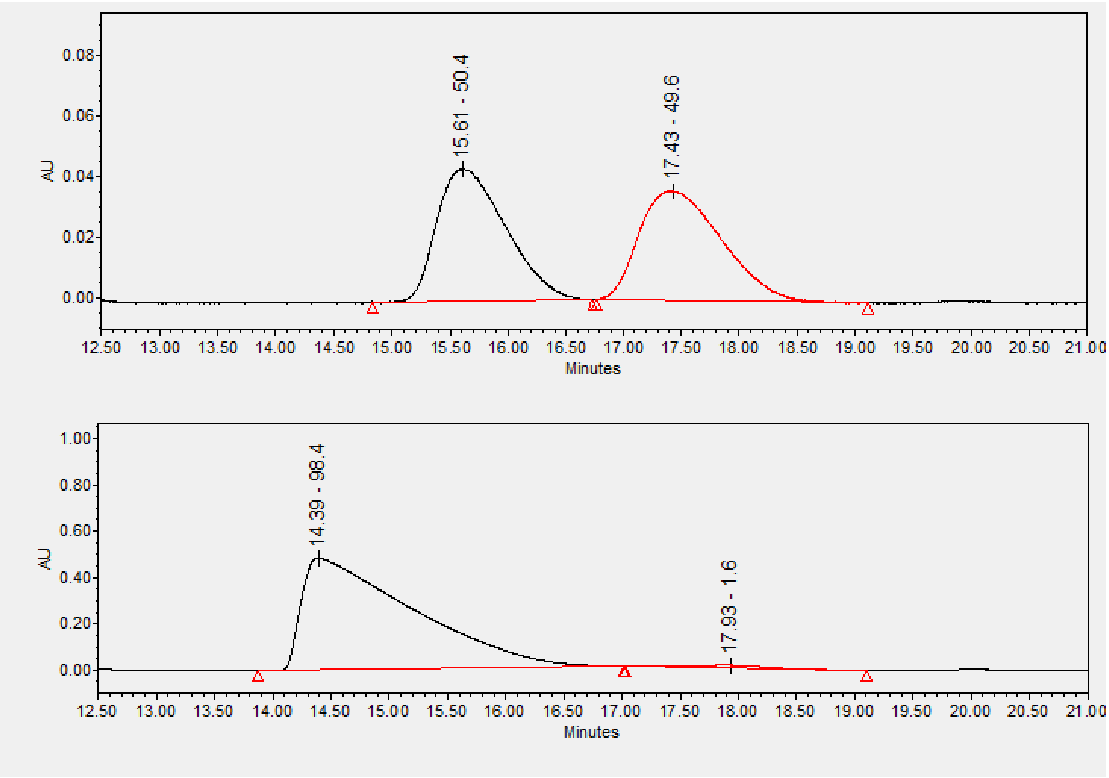

Supplementary figure 11: ^{1}H, ^{13}C-NMR spectra, HPLC traces of compound **10**

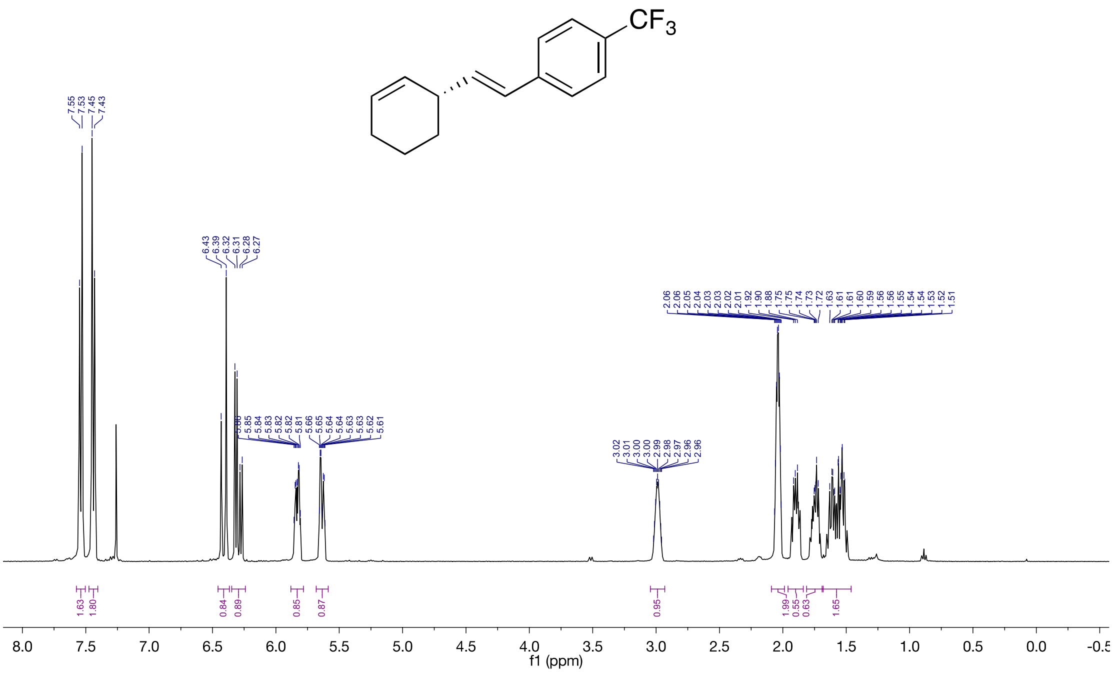

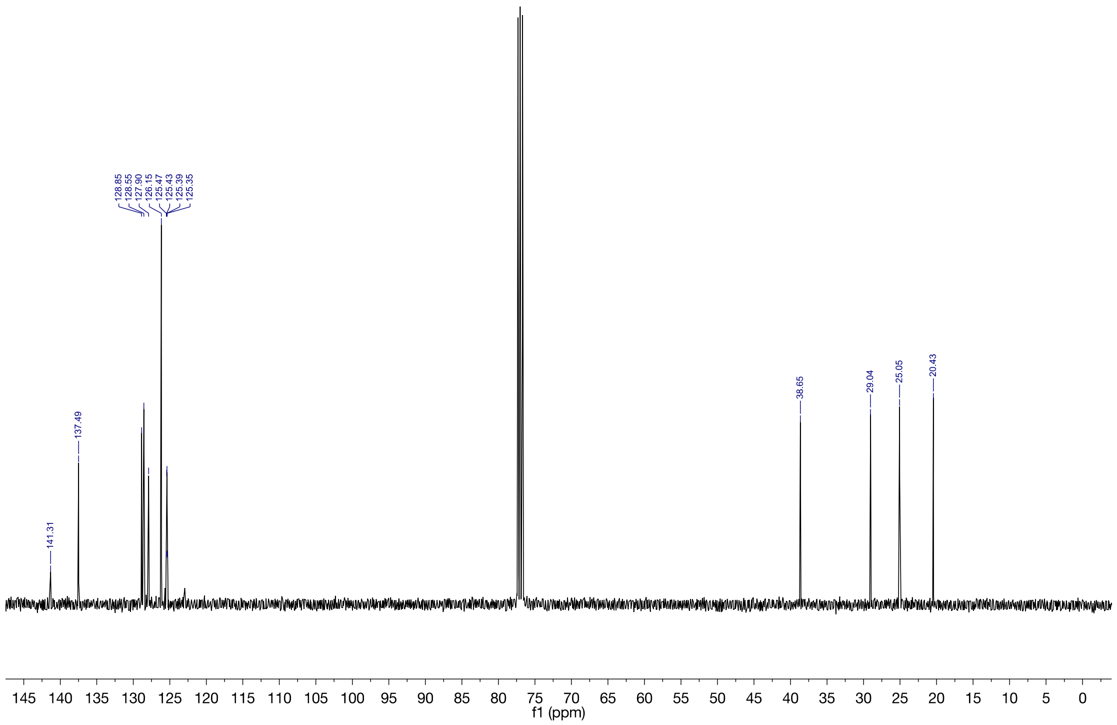

128.85
128.55
127.90
126.15
125.47
125.43
125.39
123.35
137.49
141.31
38.65
29.04
25.05
20.43
145 140 135 130 125 120 115 110 105 100 95 90 85 80 75 70 65 60 55 50 45 40 35 30 25 20 15 10 5 0
f1 (ppm)

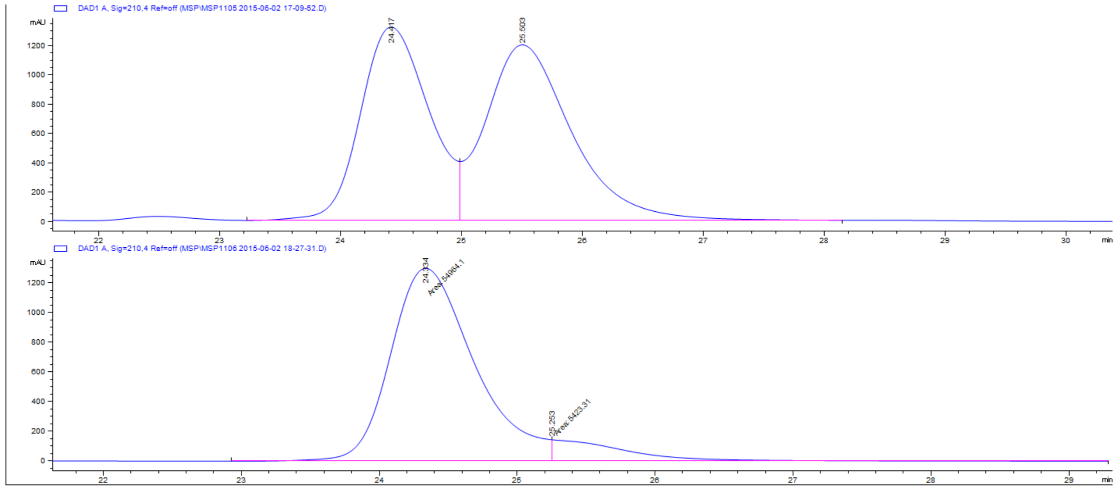

DAD1 A, Sig=210,4 Ref=off (MSP\MSP1105 2015-06-02 17-09-52.D)
mAU
24.417
25.503
min
DAD1 A, Sig=210,4 Ref=off (MSP\MSP1106 2015-06-02 18-27-31.D)
24.334
Area: 54964.1
25.253
Area: 5423.31

Supplementary figure 12: ^{1}H, ^{13}C-NMR spectra, SFC traces of compound **11**

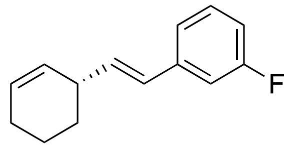

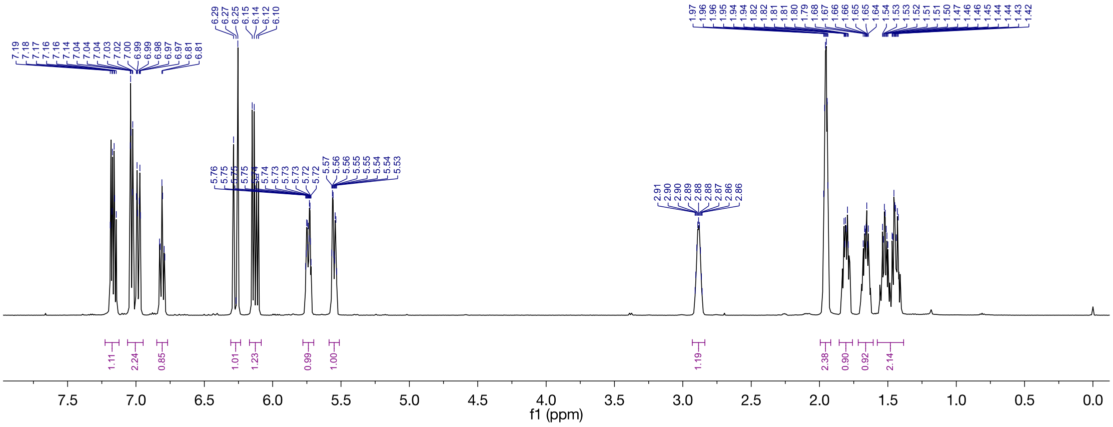

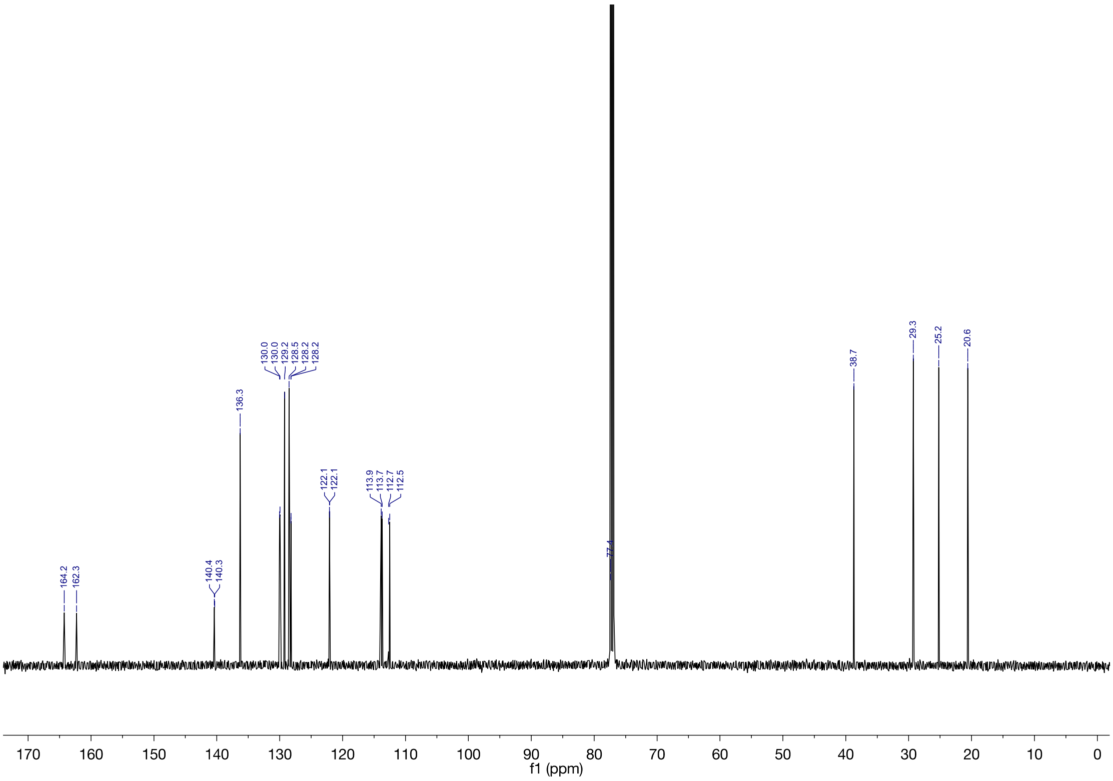

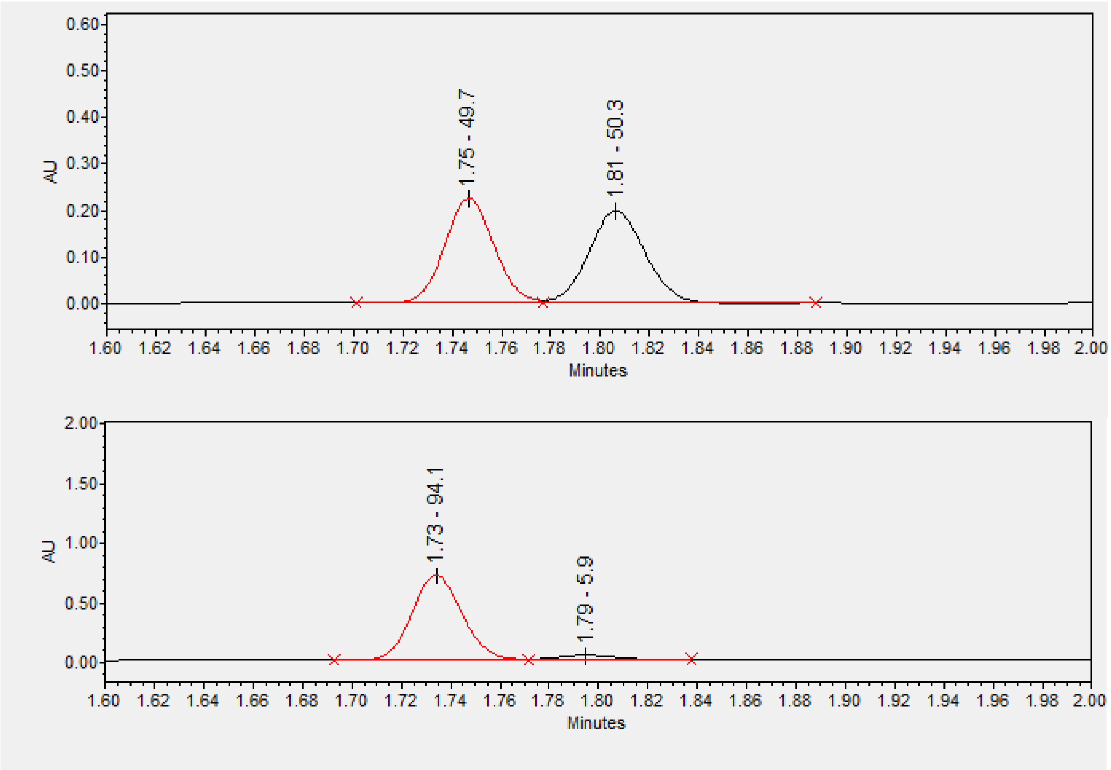

Supplementary figure 13: ^{1}H, ^{13}C-NMR spectra, SFC traces of compound **12**

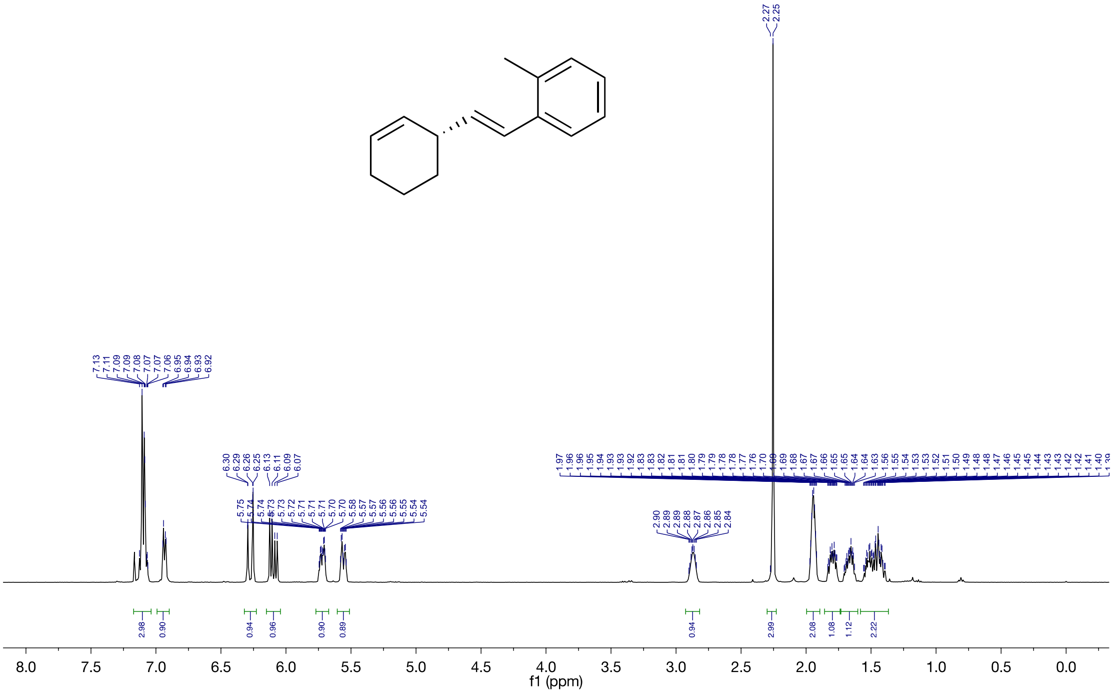

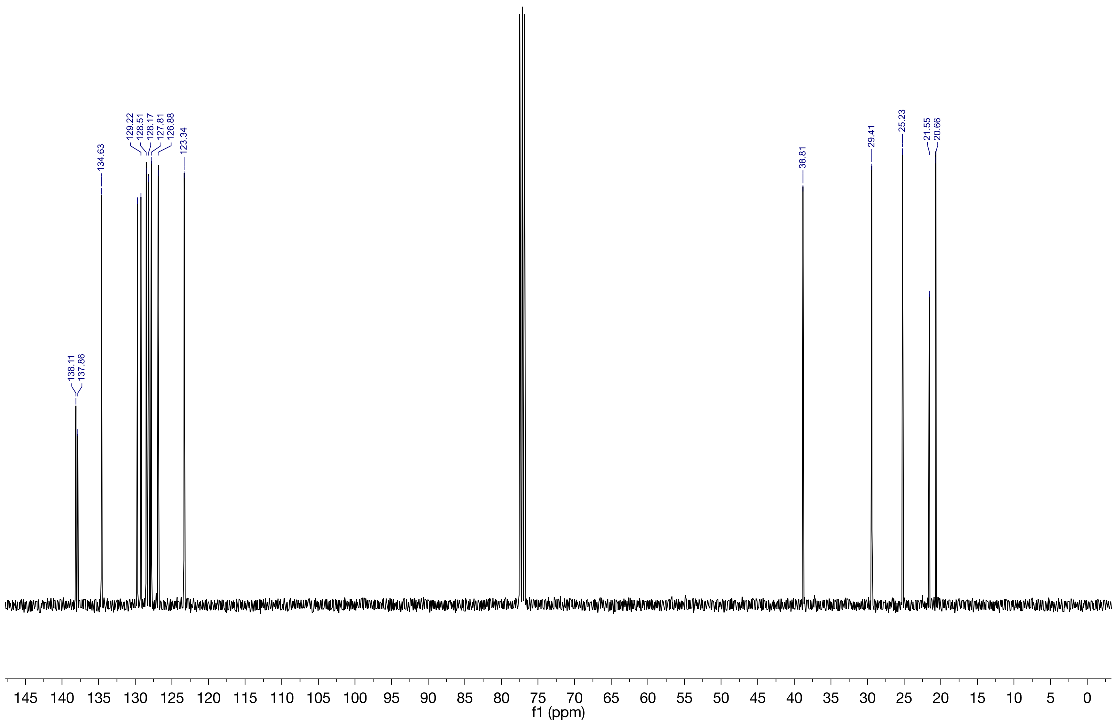

138.11
137.86
134.63
129.22
128.51
128.17
127.81
126.88
123.34
38.81
29.41
25.23
21.55
20.66
145 140 135 130 125 120 115 110 105 100 95 90 85 80 75 70 65 60 55 50 45 40 35 30 25 20 15 10 5 0
f1 (ppm)

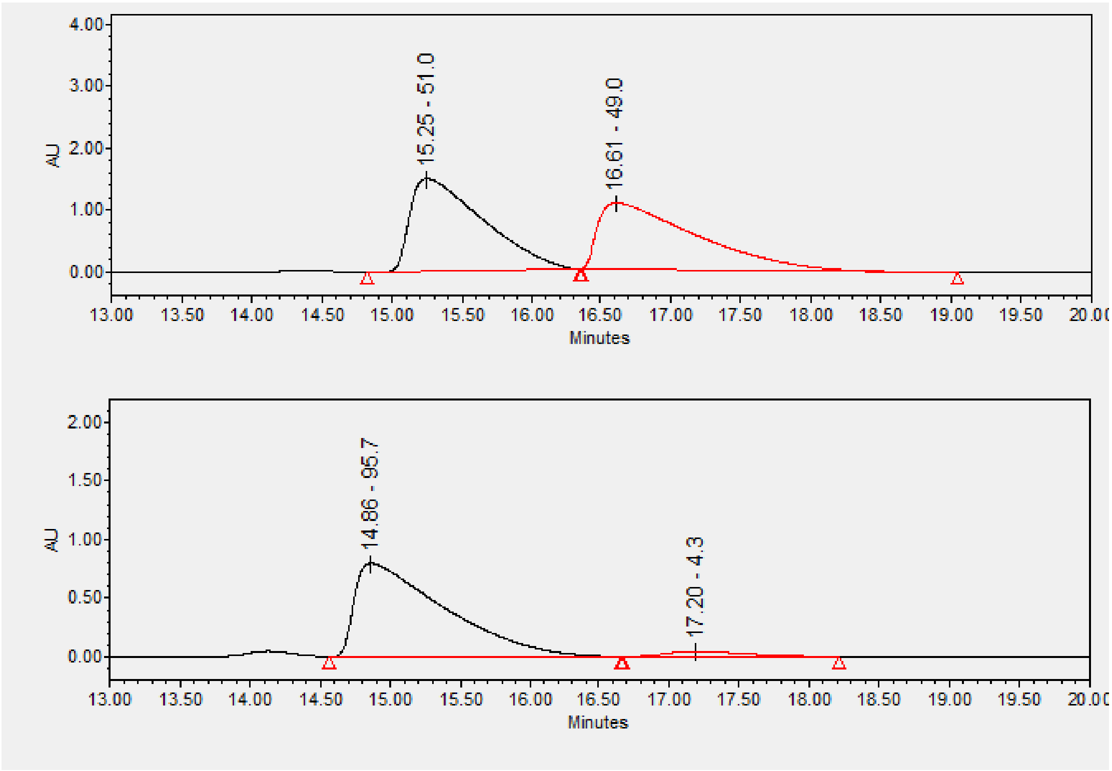

AU
4.00
3.00
2.00
1.00
0.00
15.25 - 51.0
16.61 - 49.0
13.00 13.50 14.00 14.50 15.00 15.50 16.00 16.50 17.00 17.50 18.00 18.50 19.00 19.50
Minutes
AU
2.00
1.50
1.00
0.50
0.00
14.86 - 95.7
17.20 - 4.3
13.00 13.50 14.00 14.50 15.00 15.50 16.00 16.50 17.00 17.50 18.00 18.50 19.00 19.50
Minutes

Supplementary figure 14: ^{1}H, ^{13}C-NMR spectra, SFC traces of compound **13**

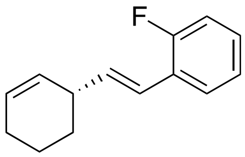

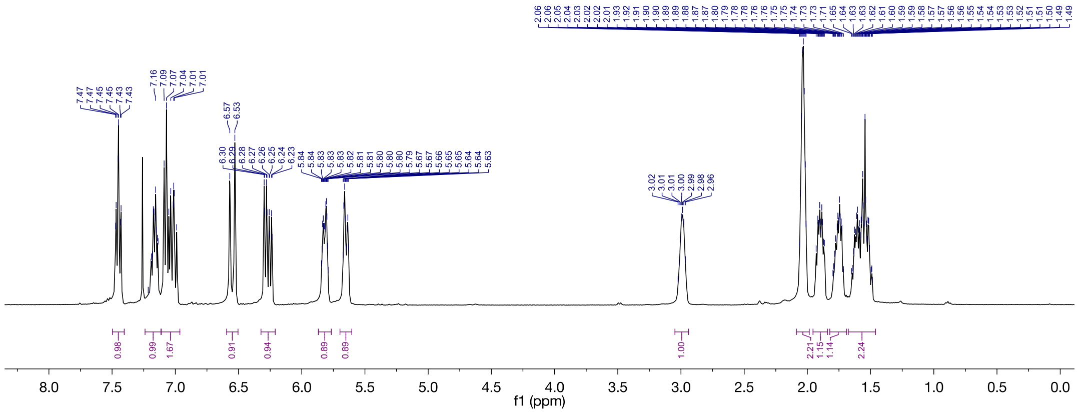

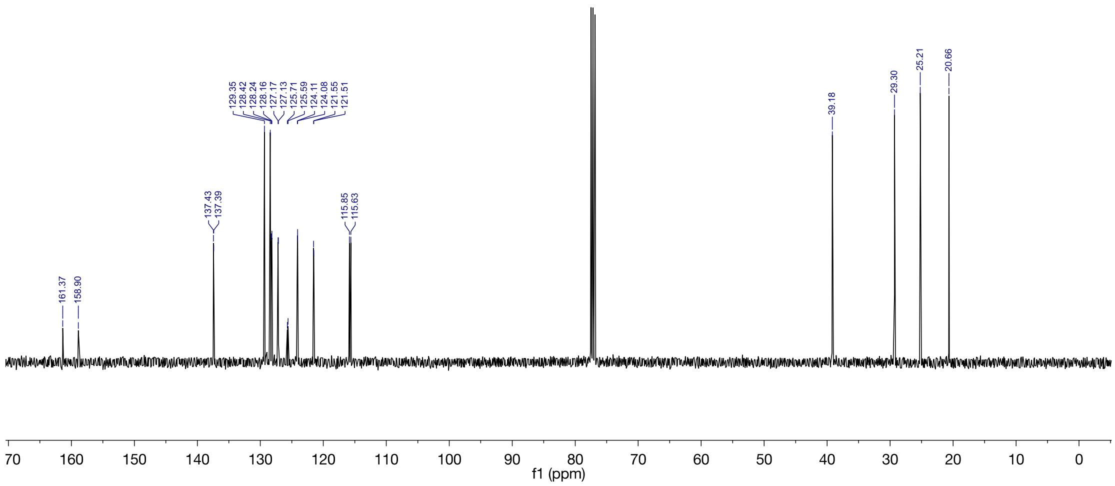

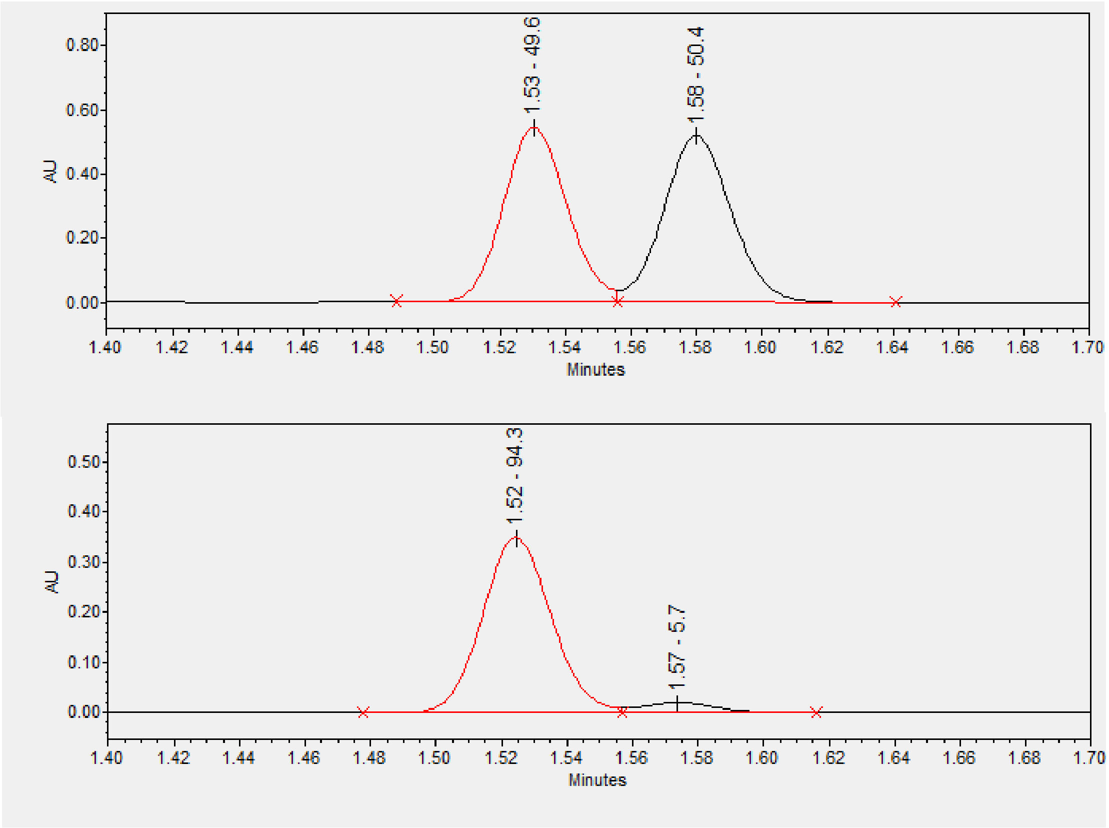

Supplementary figure 15: ^{1}H, ^{13}C-NMR spectra, SFC traces of compound **14**

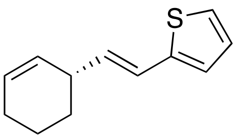

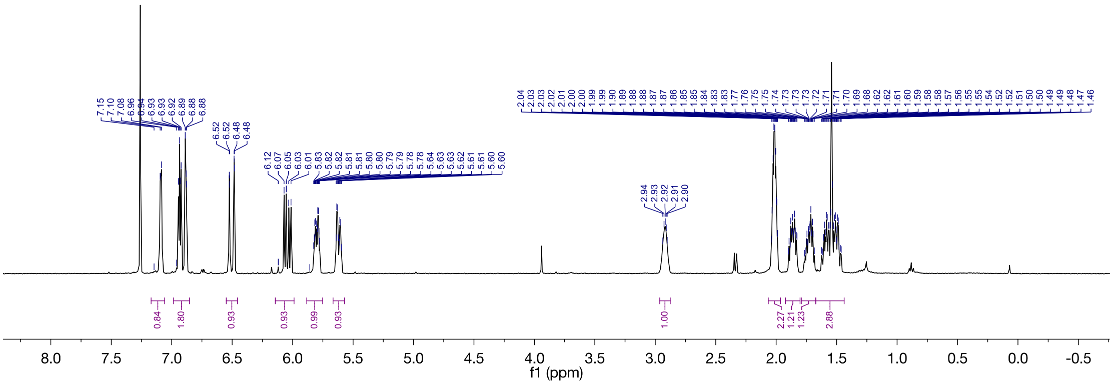

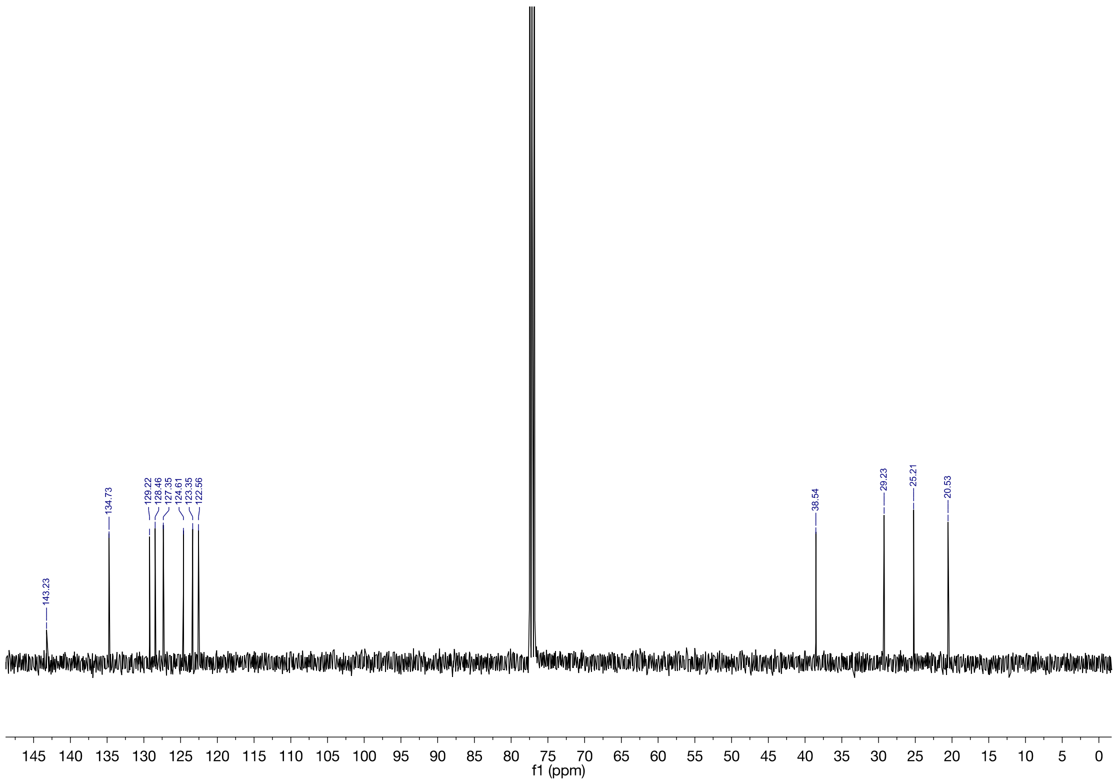
143.23
134.73
129.22
128.46
127.35
124.61
123.35
122.56
38.54
29.23
25.21
20.53
145 140 135 130 125 120 115 110 105 100 95 90 85 80 75 70 65 60 55 50 45 40 35 30 25 20 15 10 5 0
f1 (ppm)

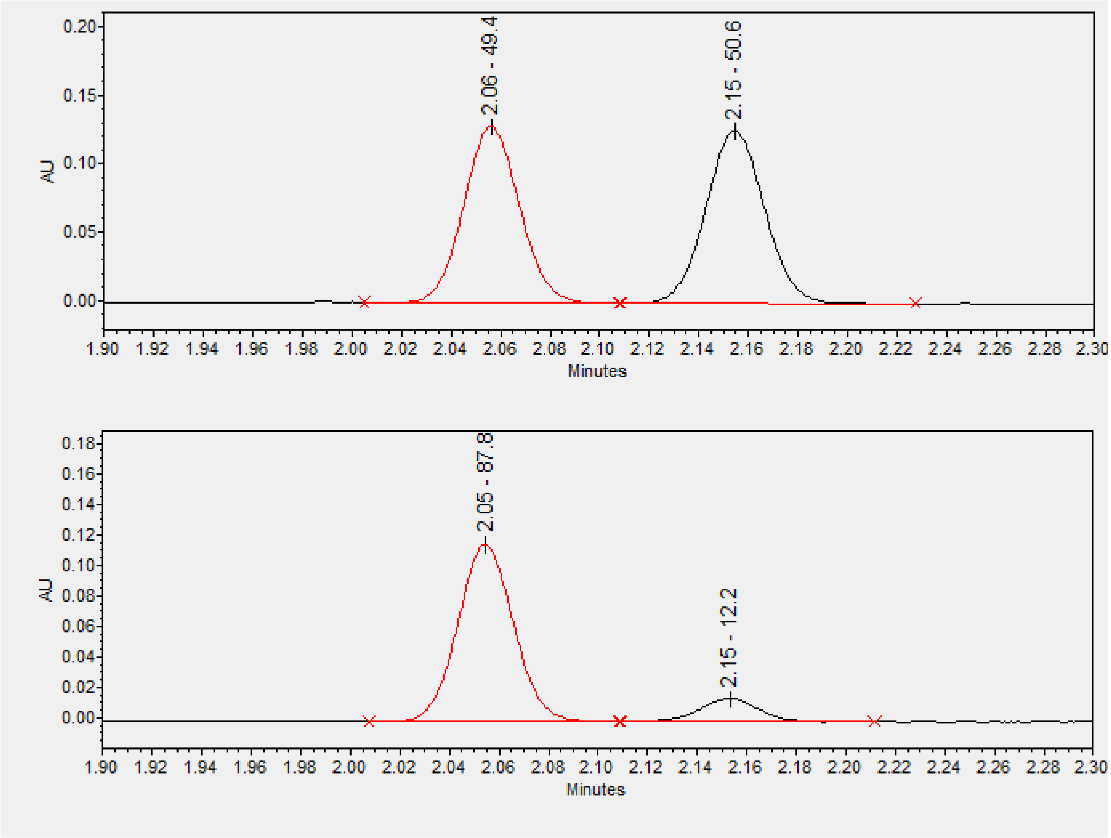

Supplementary figure 16: ^{1}H, ^{13}C-NMR spectra, SFC traces of compound **15**

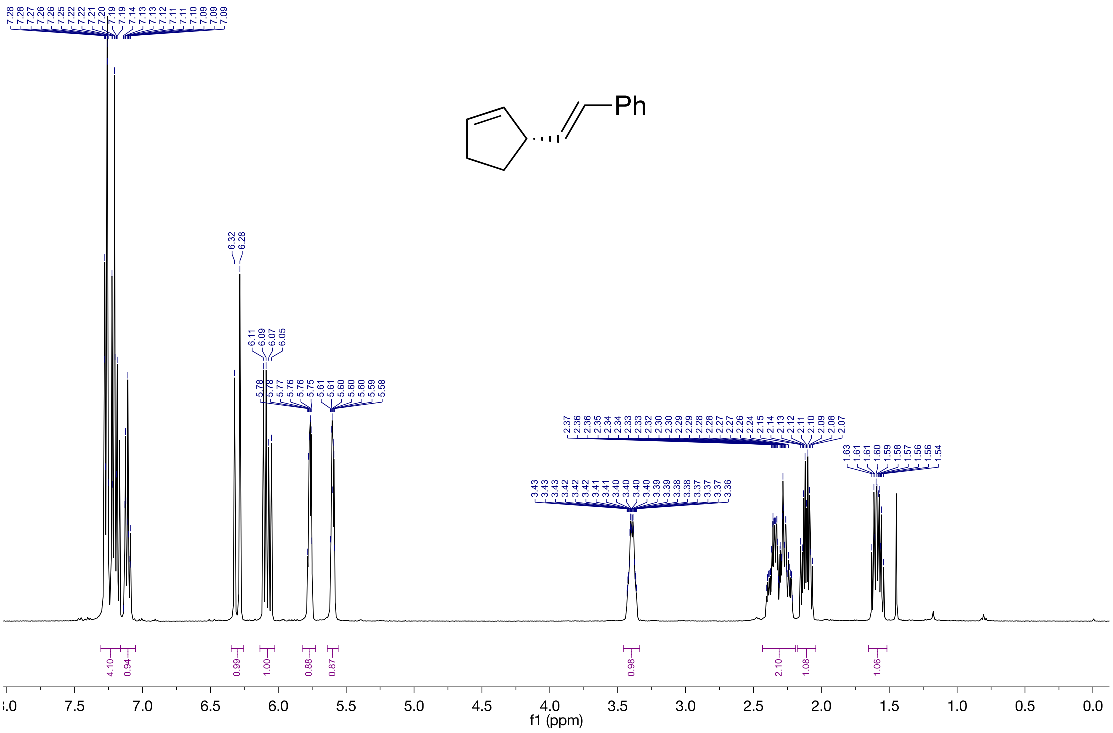

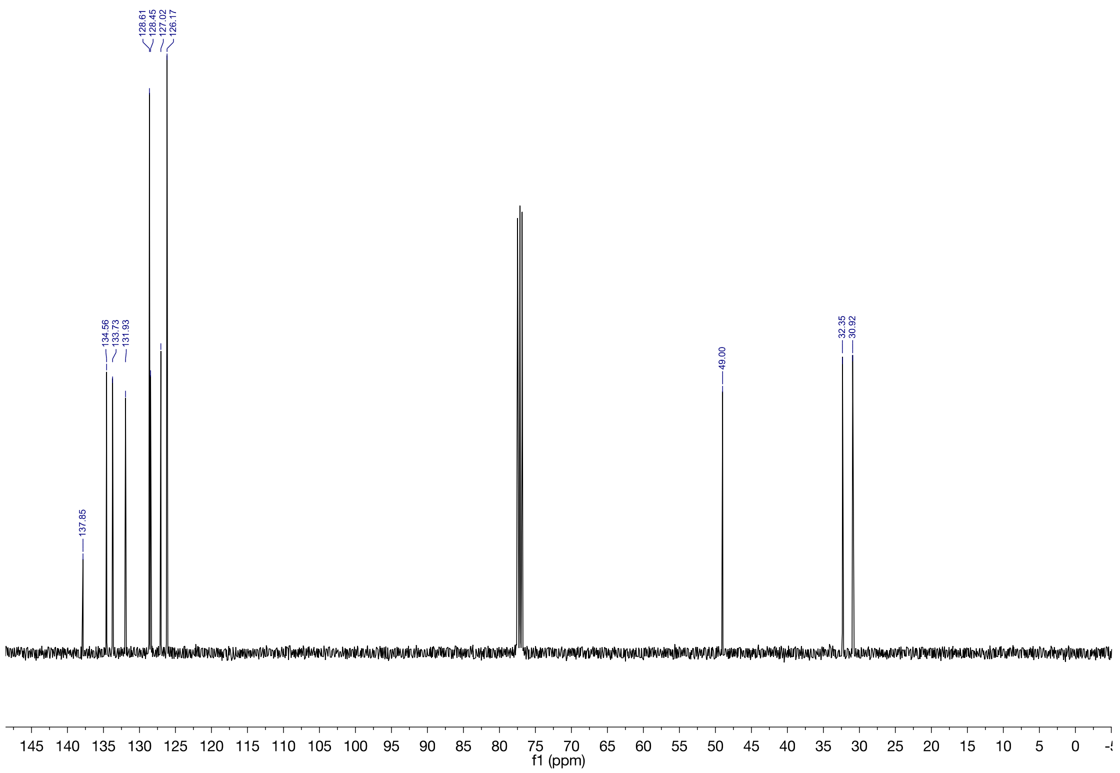
137.85
134.56
133.73
131.93
128.61
128.45
127.02
126.17
49.00
32.35
30.92
f1 (ppm)

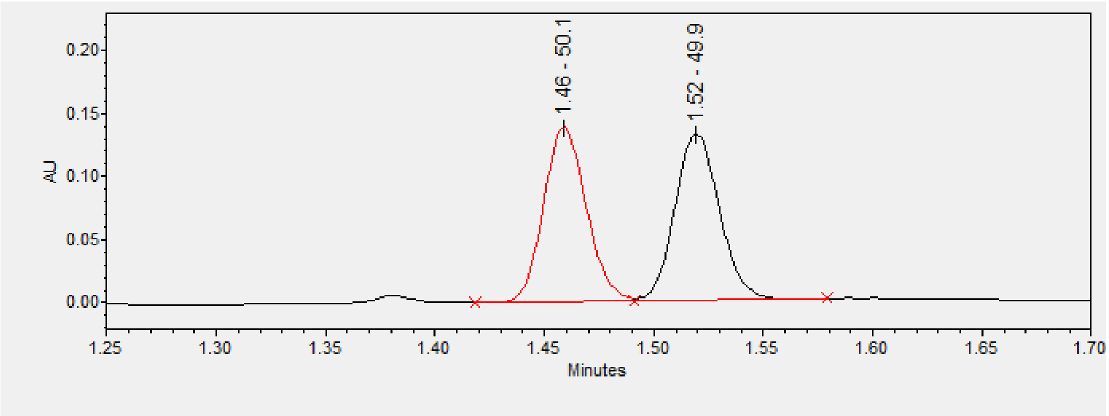
1.46 - 50.1
1.52 - 49.9
AU
Minutes

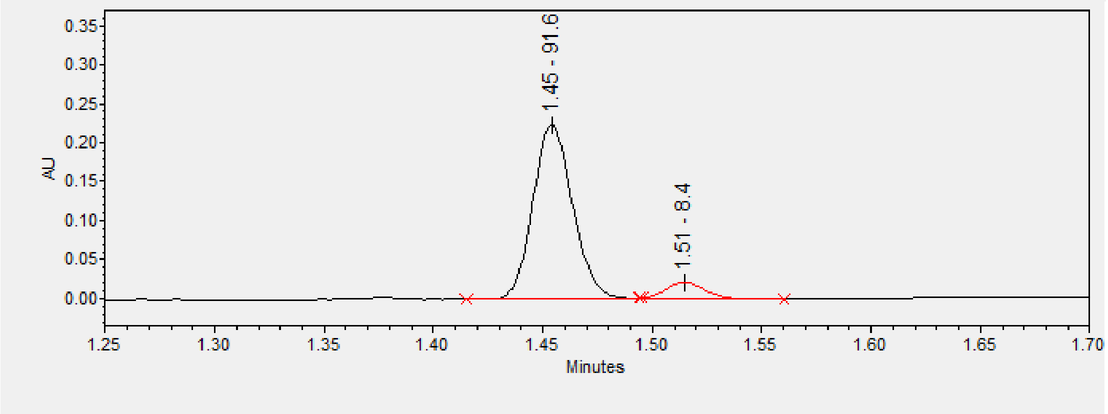
1.45 - 91.6
1.51 - 8.4
AU
Minutes

Supplementary figure 17: ^{1}H, ^{13}C-NMR spectra, HPLC traces of compound **16**

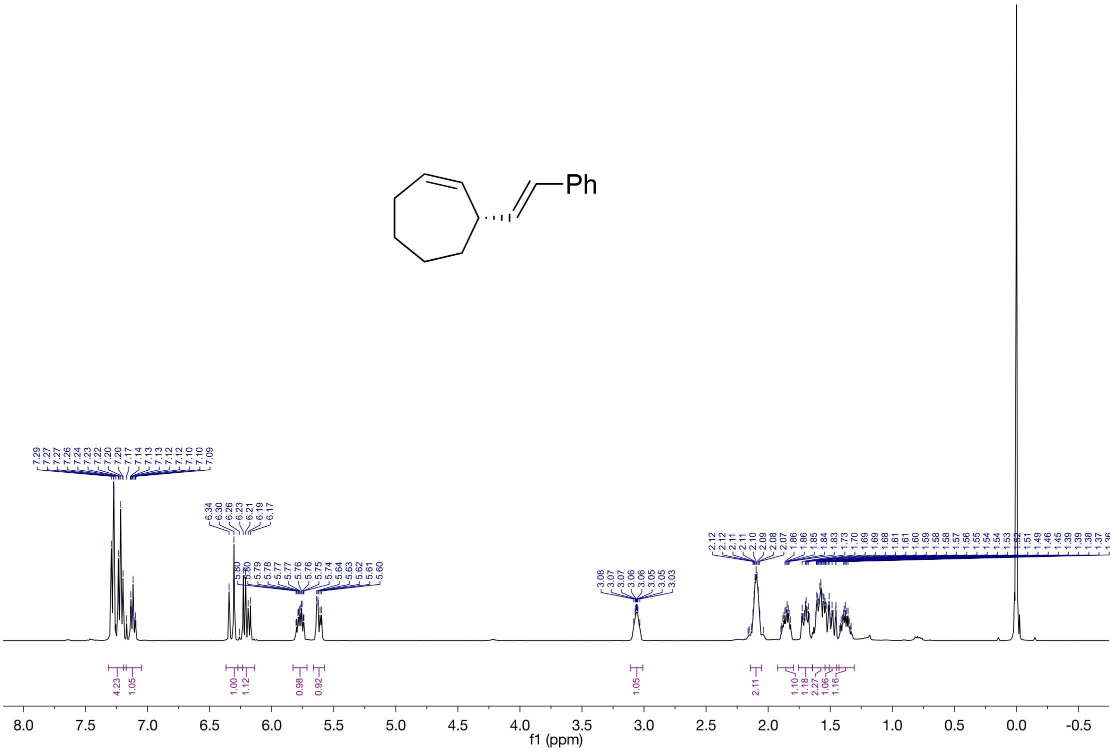

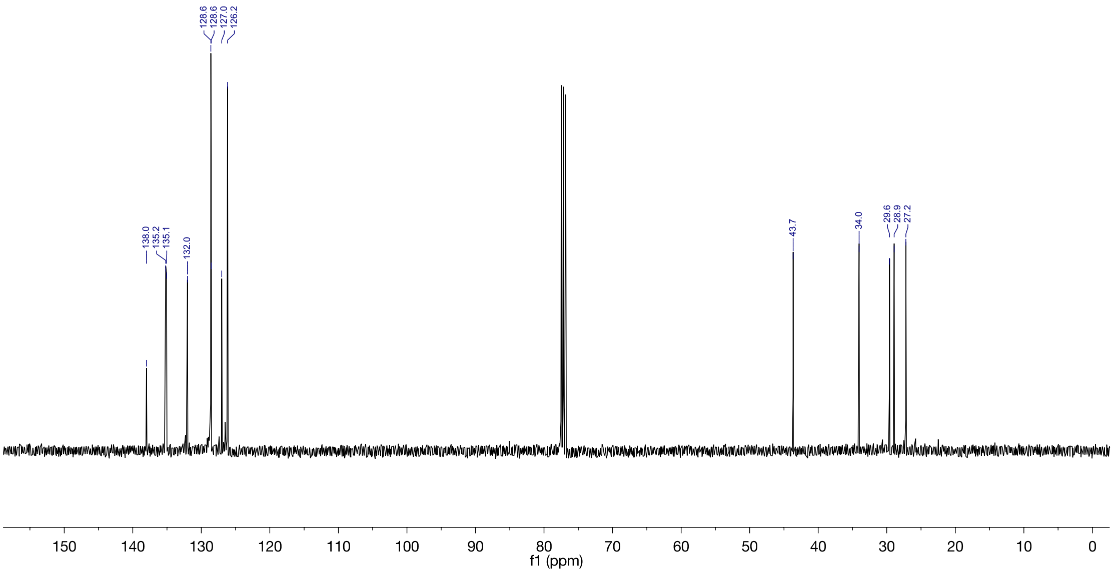

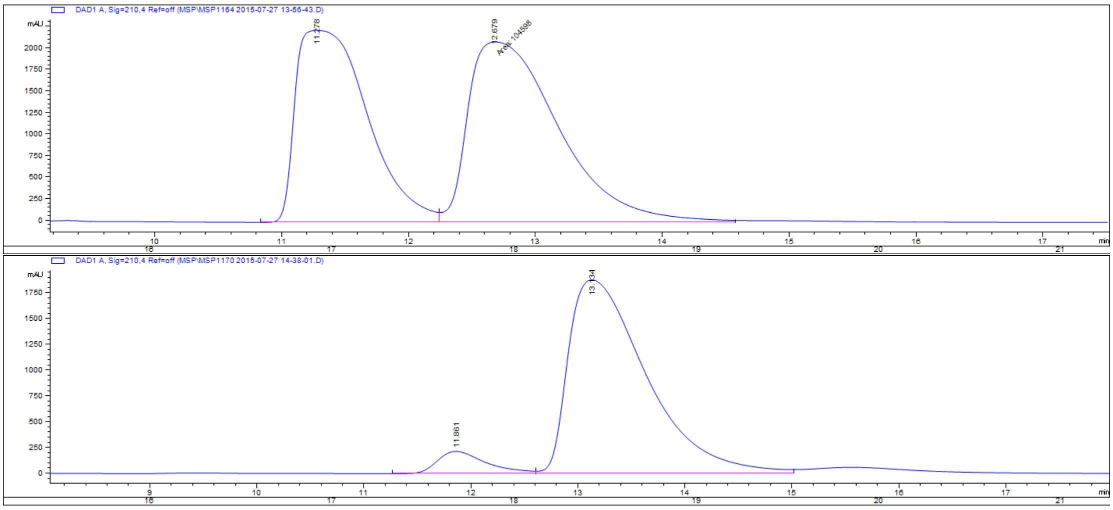

Supplementary figure 18: ^{1}H, ^{13}C-NMR spectra, SFC traces of compound **17**

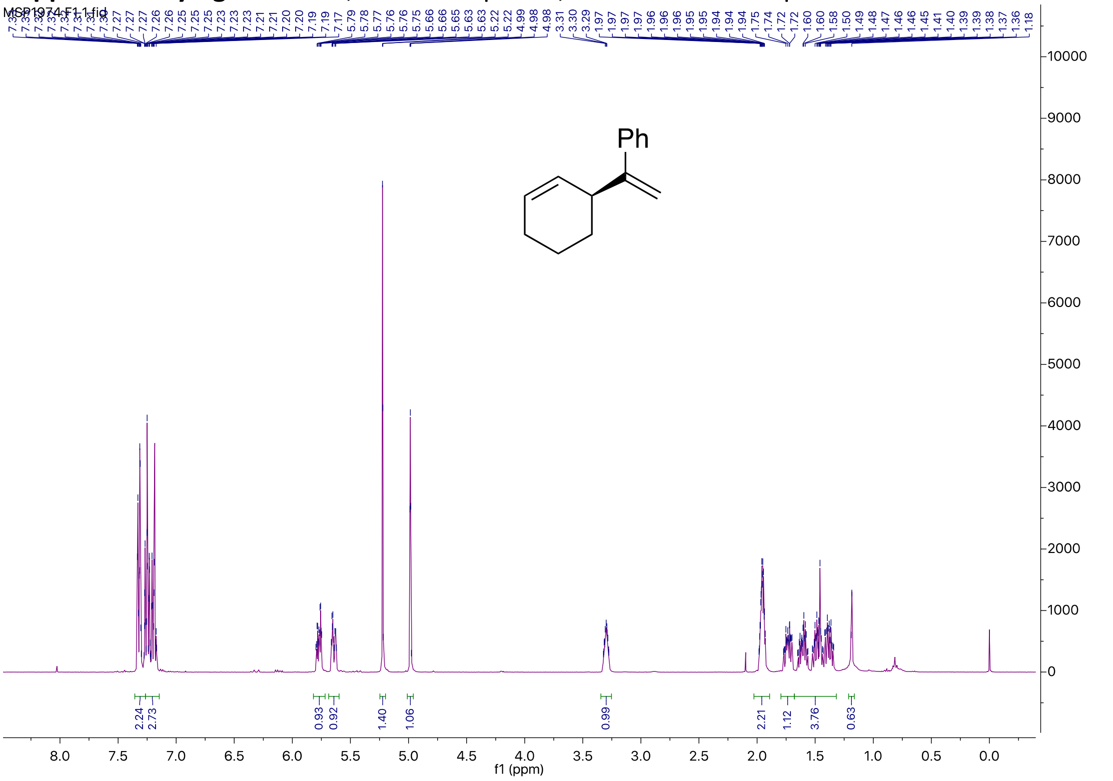

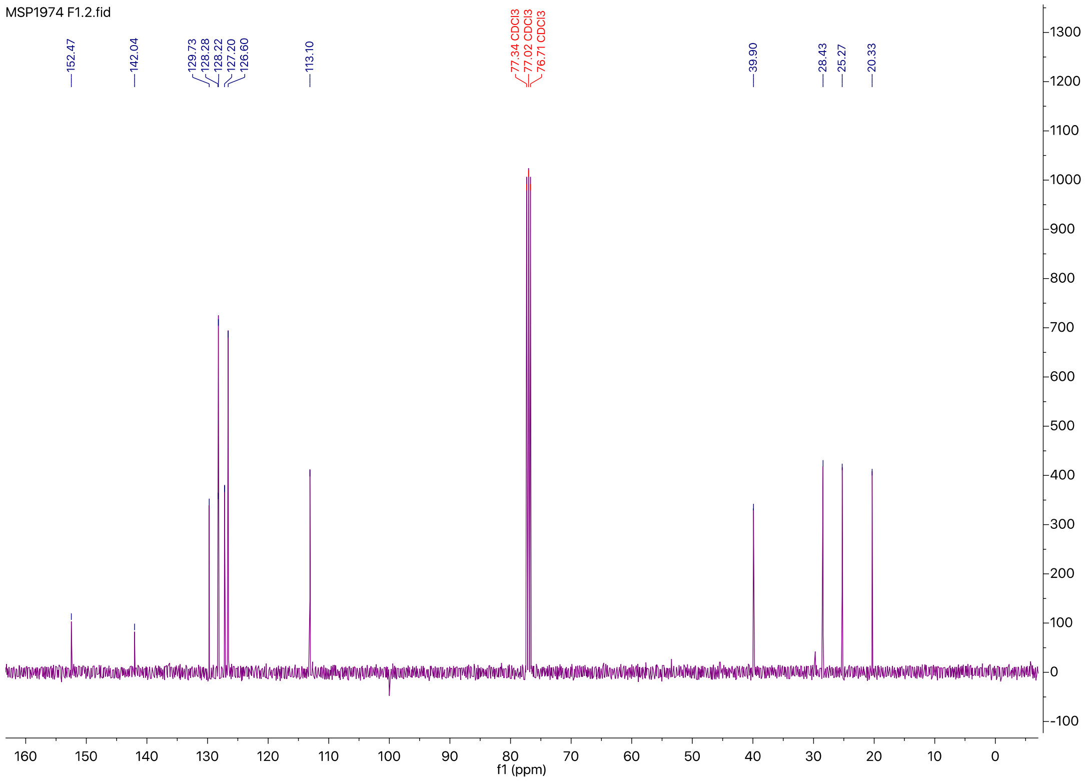
MSP1974 F1.2.fid
152.47
142.04
129.73
128.28
128.22
127.20
126.60
113.10
77.34 CDCl3
77.02 CDCl3
76.71 CDCl3
39.90
28.43
25.27
20.33
f1 (ppm)

SFC traces:

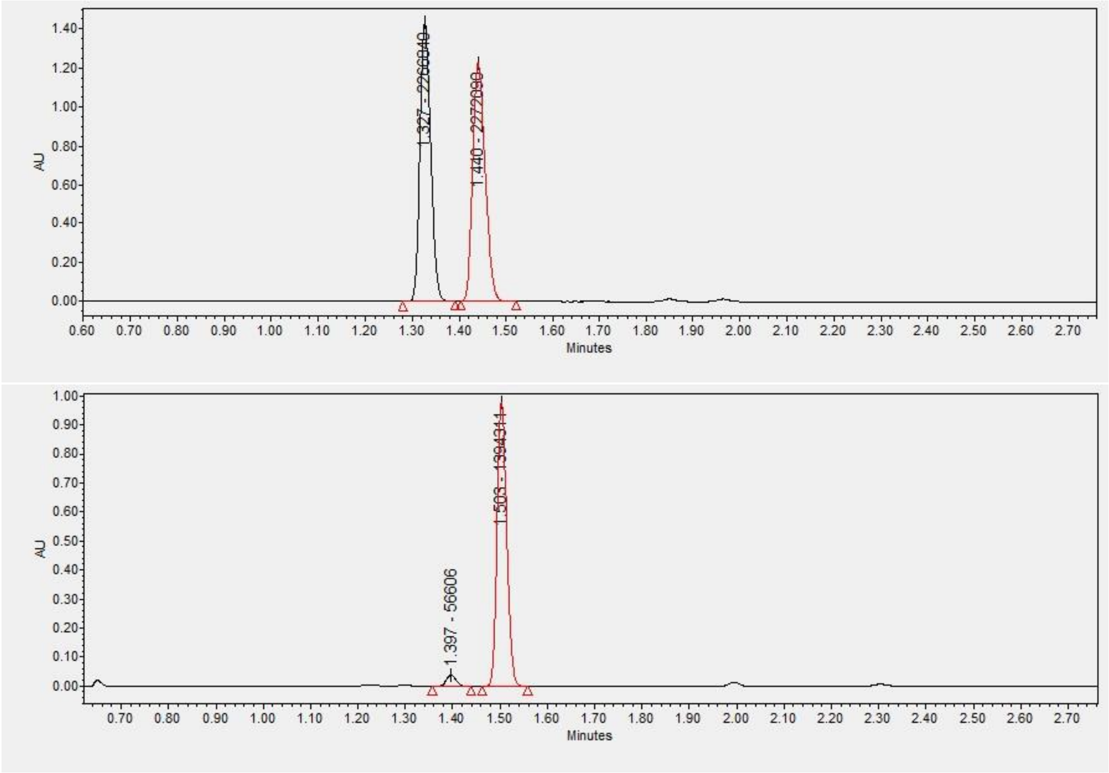
1.327 - 2260040
1.440 - 2272090
AU
Minutes
1.397 - 56606
1.503 - 1394311
AU
Minutes

Supplementary figure 18: ^{1}H, ^{13}C-NMR spectra, SFC traces of compound **17**

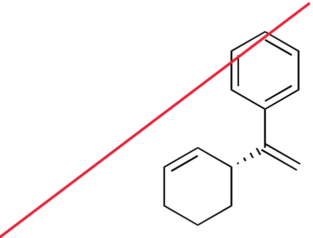

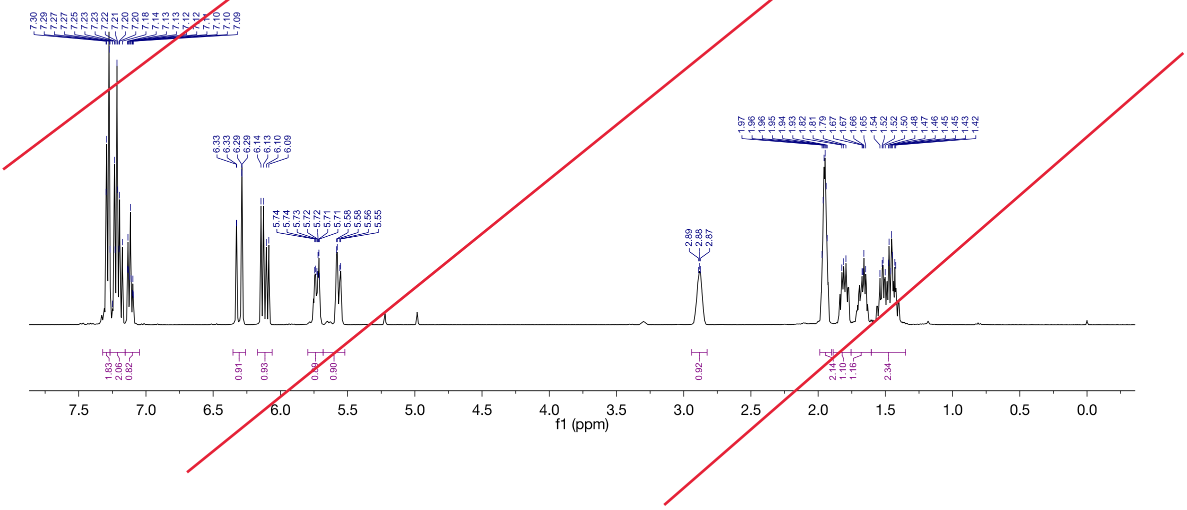

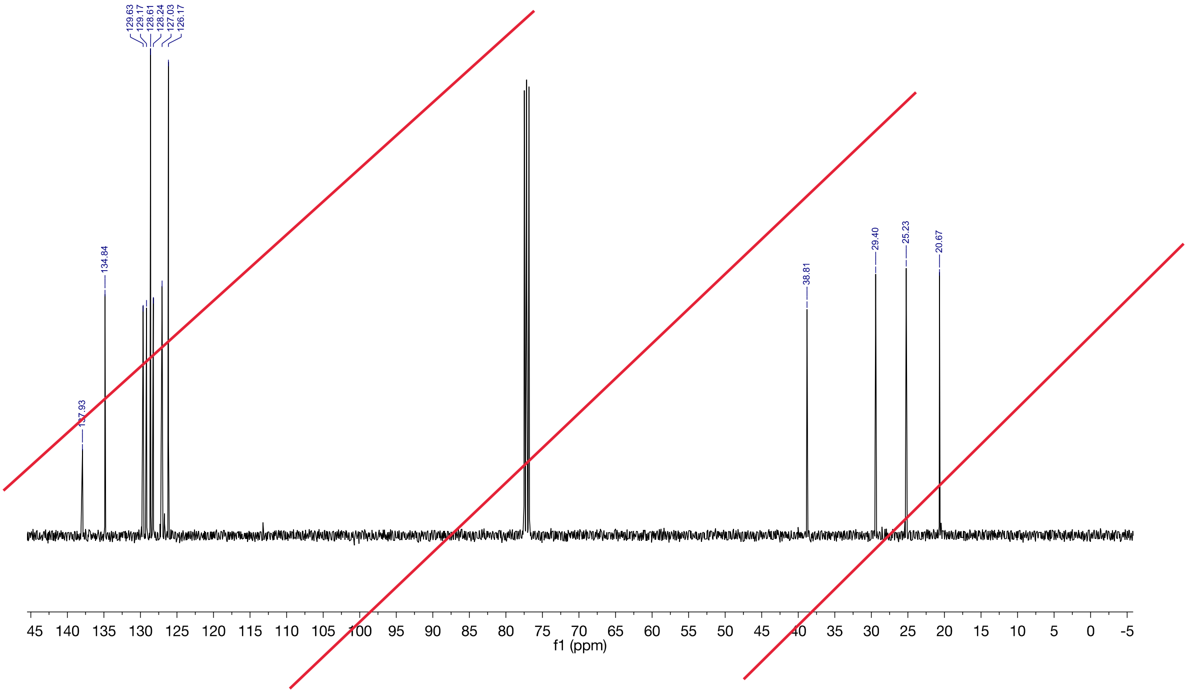

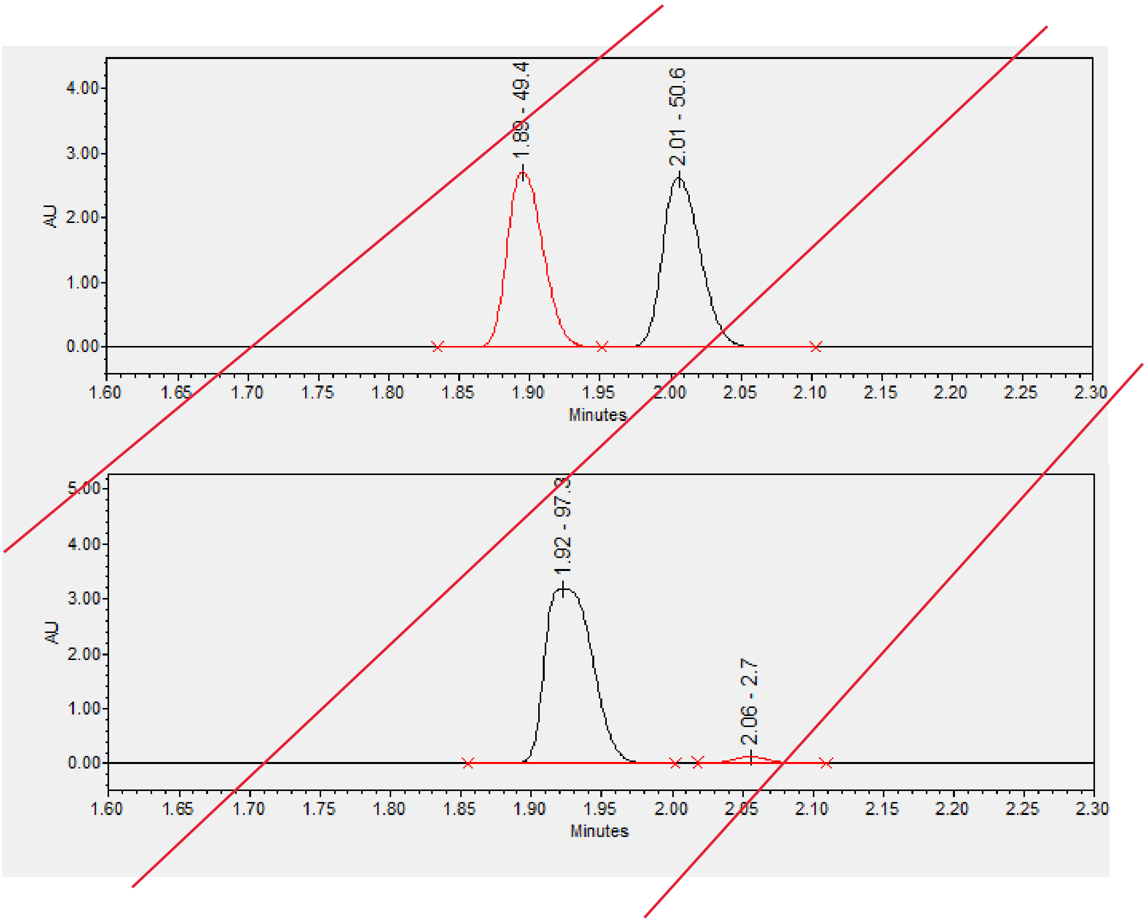

AU
4.00
3.00
2.00
1.00
0.00
1.89 - 49.4
2.01 - 50.6
1.60 1.65 1.70 1.75 1.80 1.85 1.90 1.95 2.00 2.05 2.10 2.15 2.20 2.25 2.30
Minutes
AU
5.00
4.00
3.00
2.00
1.00
0.00
1.92 - 97.3
2.06 - 2.7
1.60 1.65 1.70 1.75 1.80 1.85 1.90 1.95 2.00 2.05 2.10 2.15 2.20 2.25 2.30
Minutes

Supplementary figure 19: ^{1}H, ^{13}C-NMR spectra, HPLC traces of compound **18**

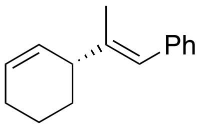

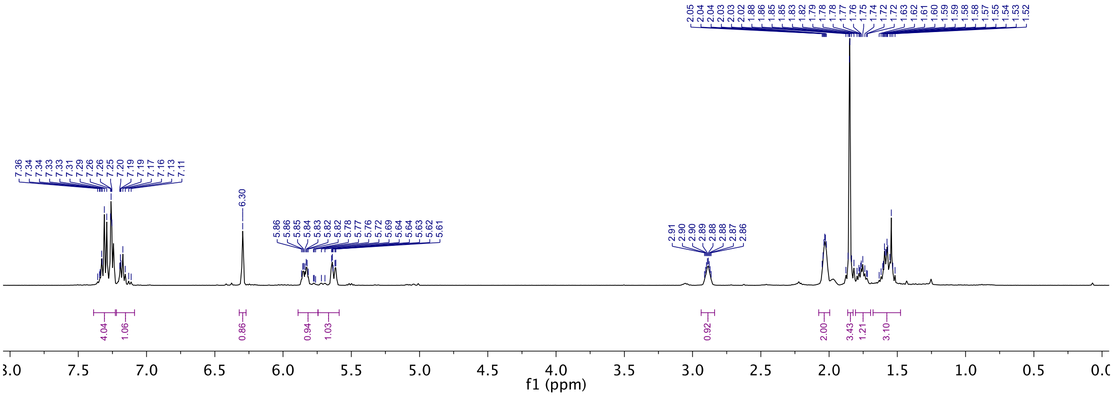

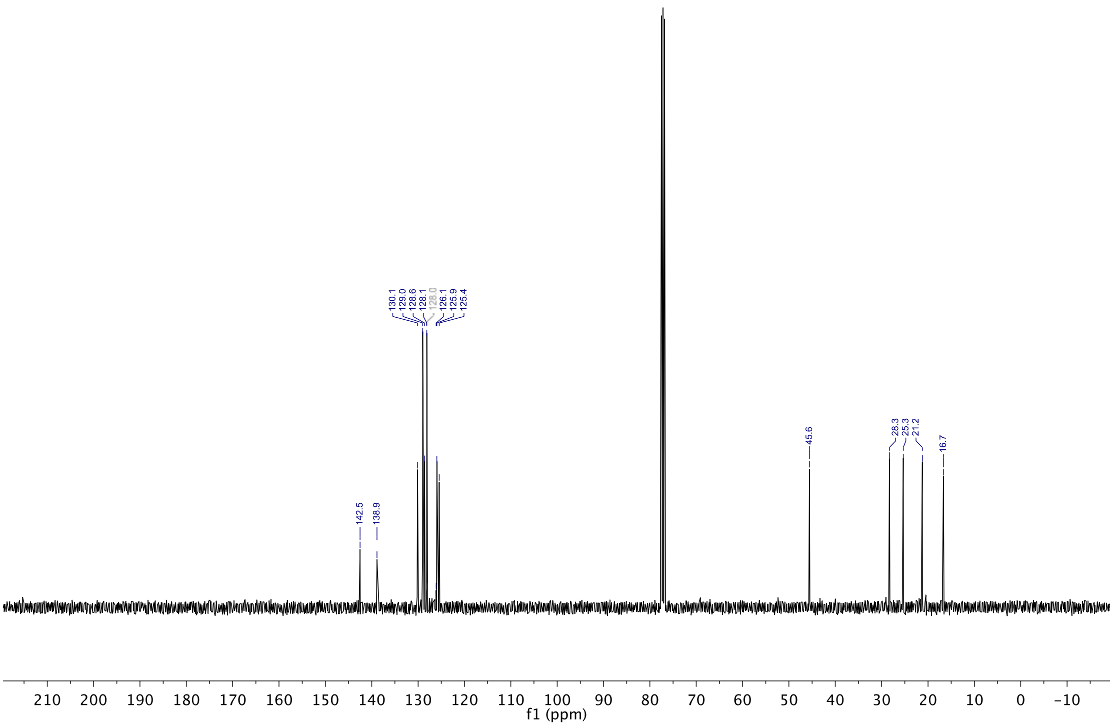

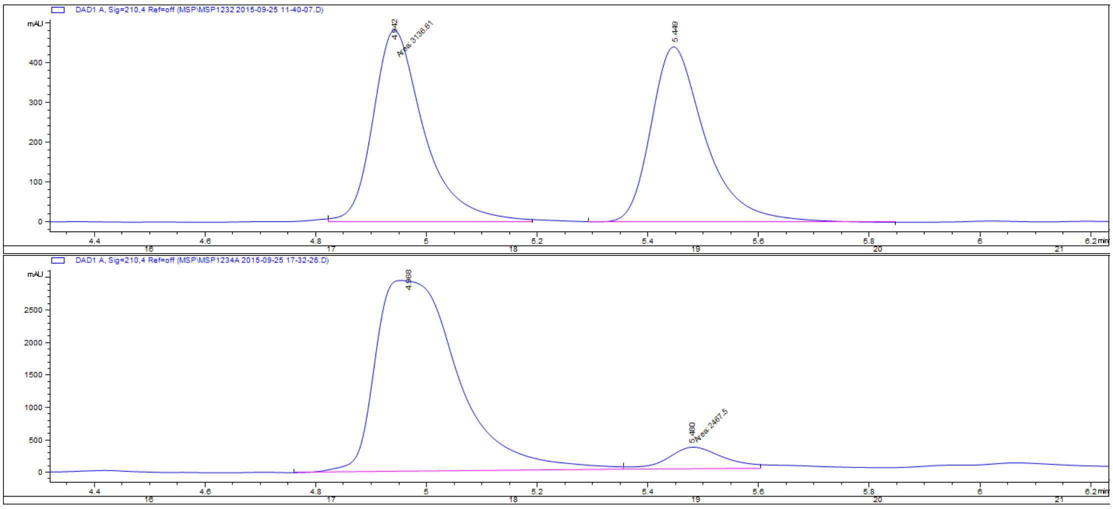

Supplementary figure 20: ^{1}H, ^{13}C-NMR spectra, HPLC traces of compound **19**

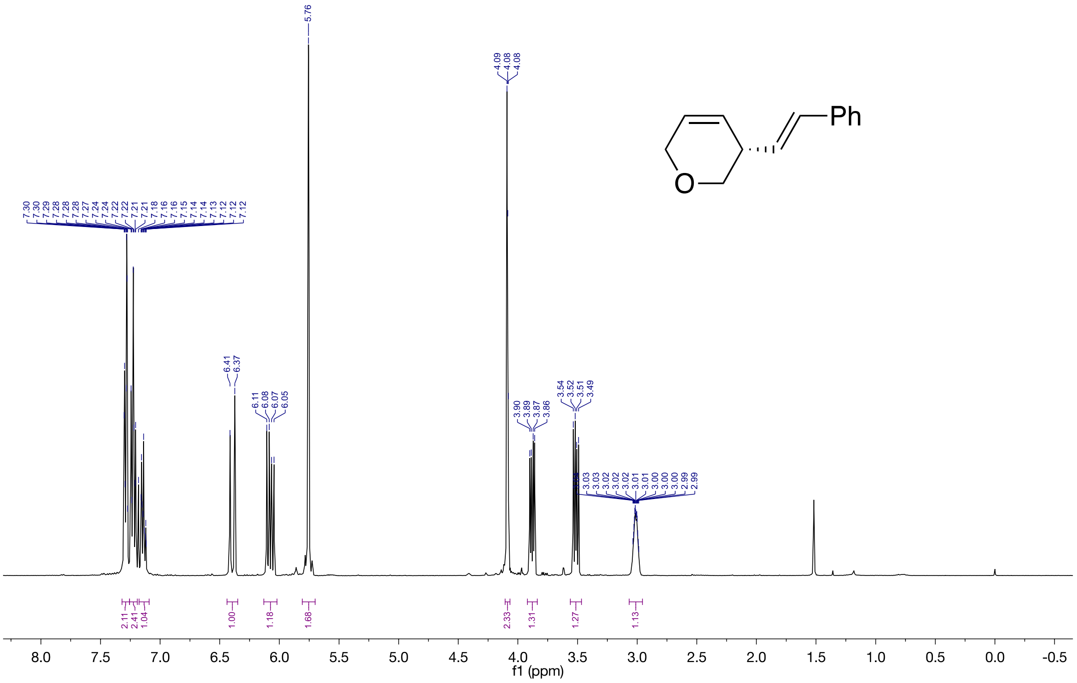

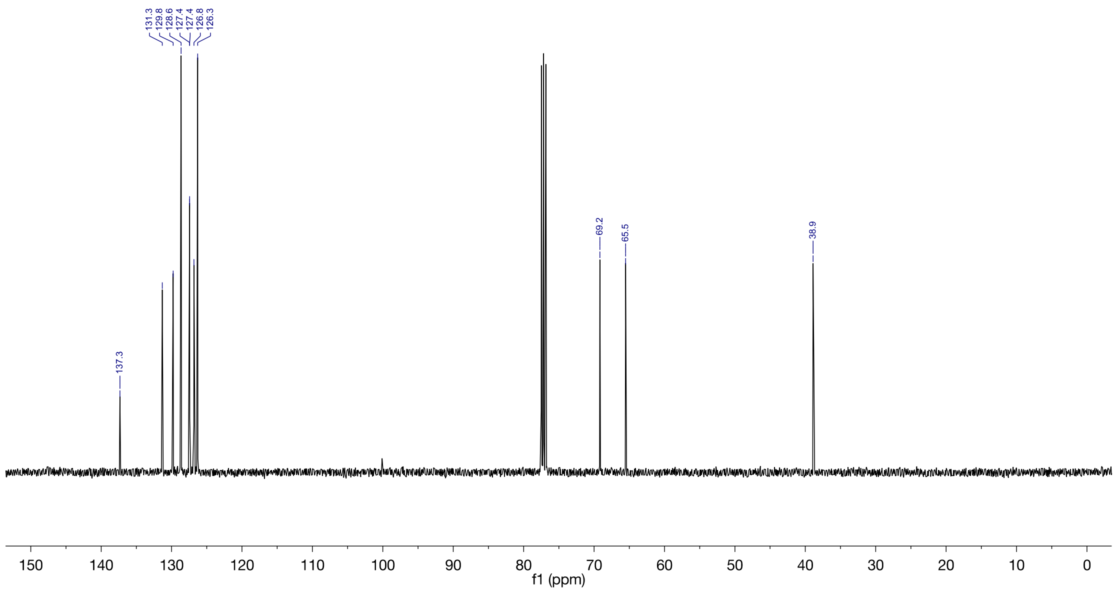
131.3
129.8
128.6
127.4
127.4
126.8
126.3
137.3
69.2
65.5
38.9
150 140 130 120 110 100 90 80 70 60 50 40 30 20 10 0
f1 (ppm)

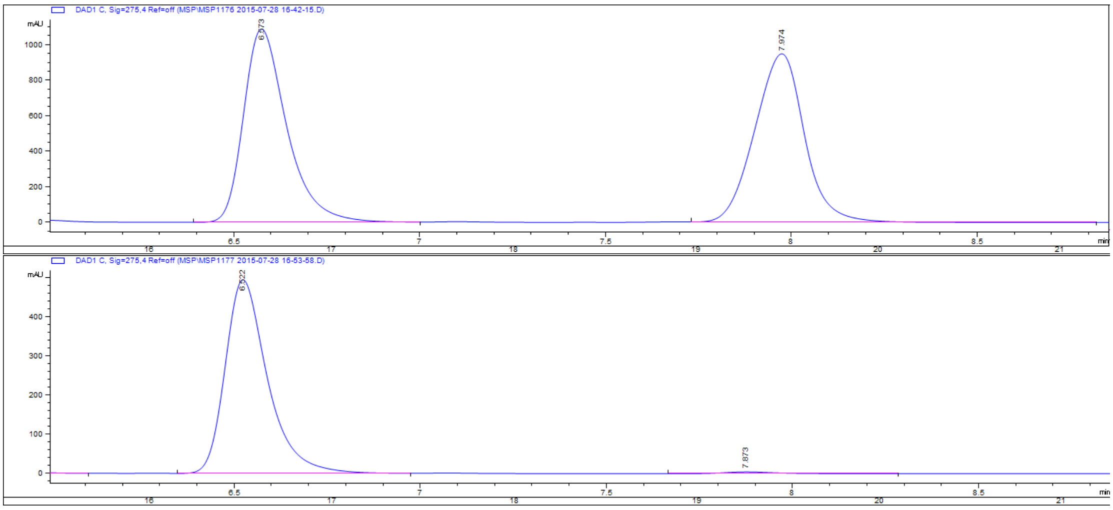

Supplementary figure 21: ^{1}H, ^{13}C-NMR spectra, HPLC traces of compound **20**

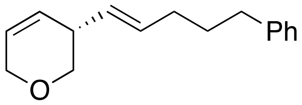

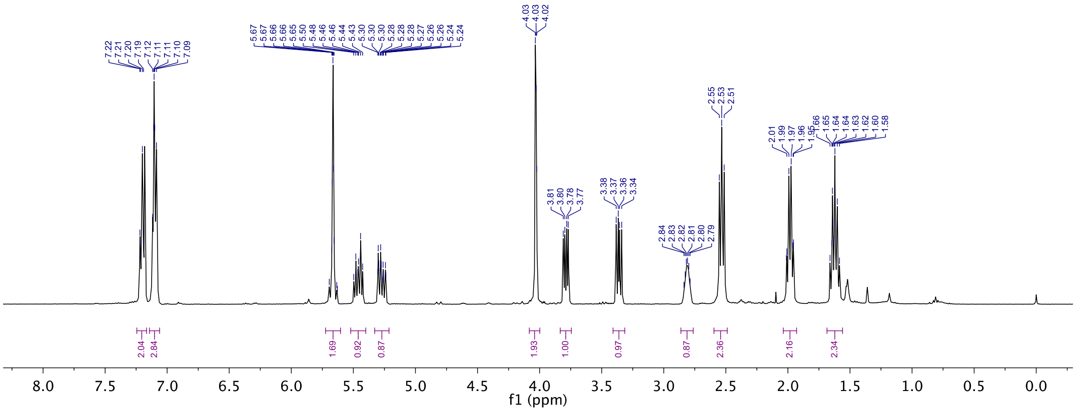

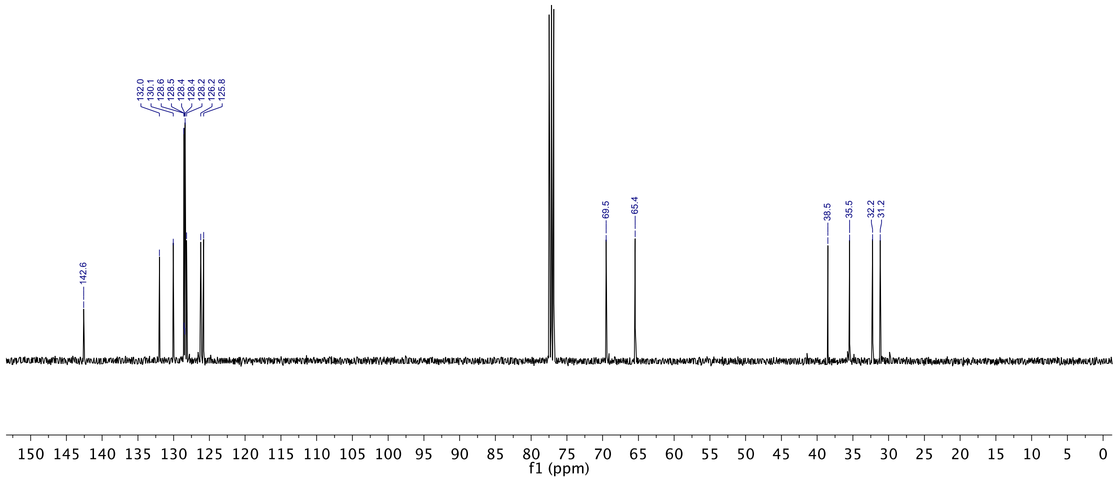
142.6
132.0
130.1
128.6
128.5
128.4
128.4
128.2
126.2
125.8
69.5
65.4
38.5
35.5
32.2
31.2
150 145 140 135 130 125 120 115 110 105 100 95 90 85 80 75 70 65 60 55 50 45 40 35 30 25 20 15 10 5 0
f1 (ppm)

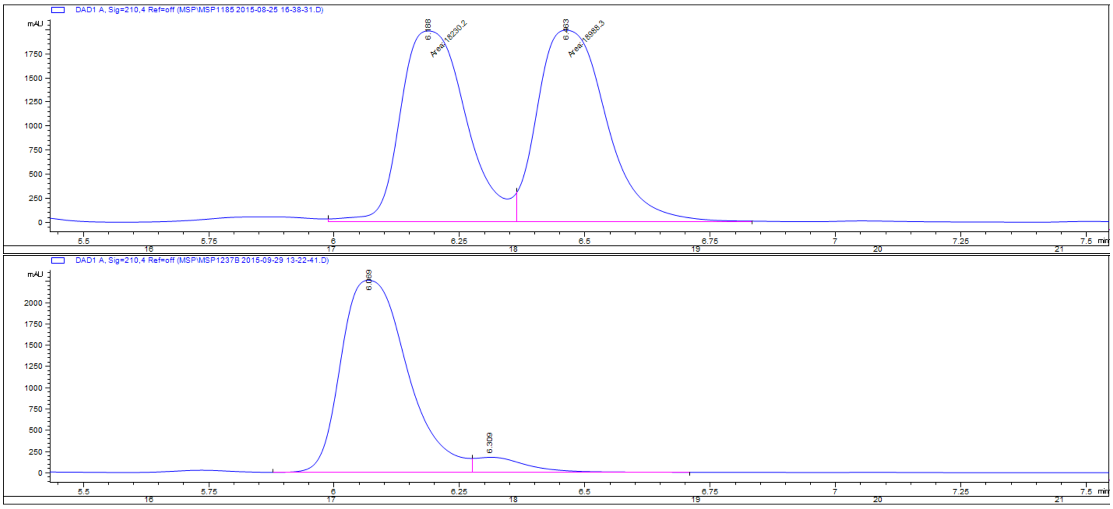

Supplementary figure 22: ^{1}H, ^{13}C-NMR spectra, GC traces of compound **21**

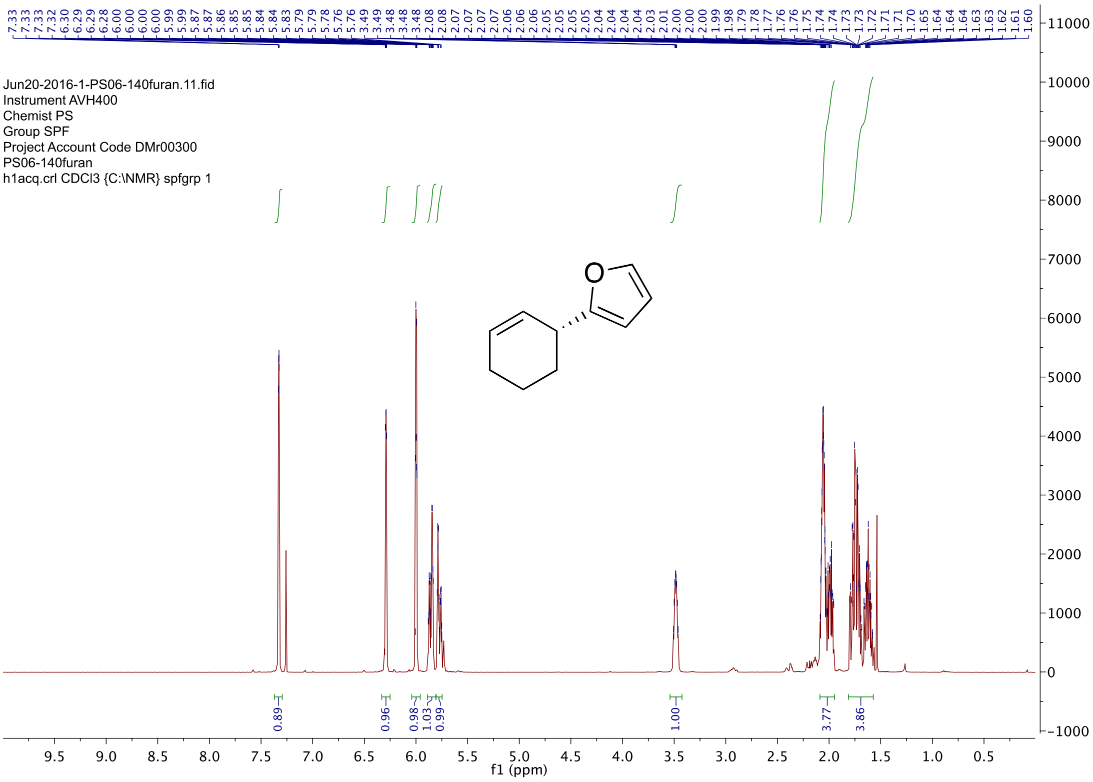

Jun20-2016-1-PS06-140furan.10.fid
Instrument AVH400
Chemist PS
Group SPF
Project Account Code DMr00300
PS06-140furan
c13acq.crl CDCl3 {C:\NMR} spfgrp 1

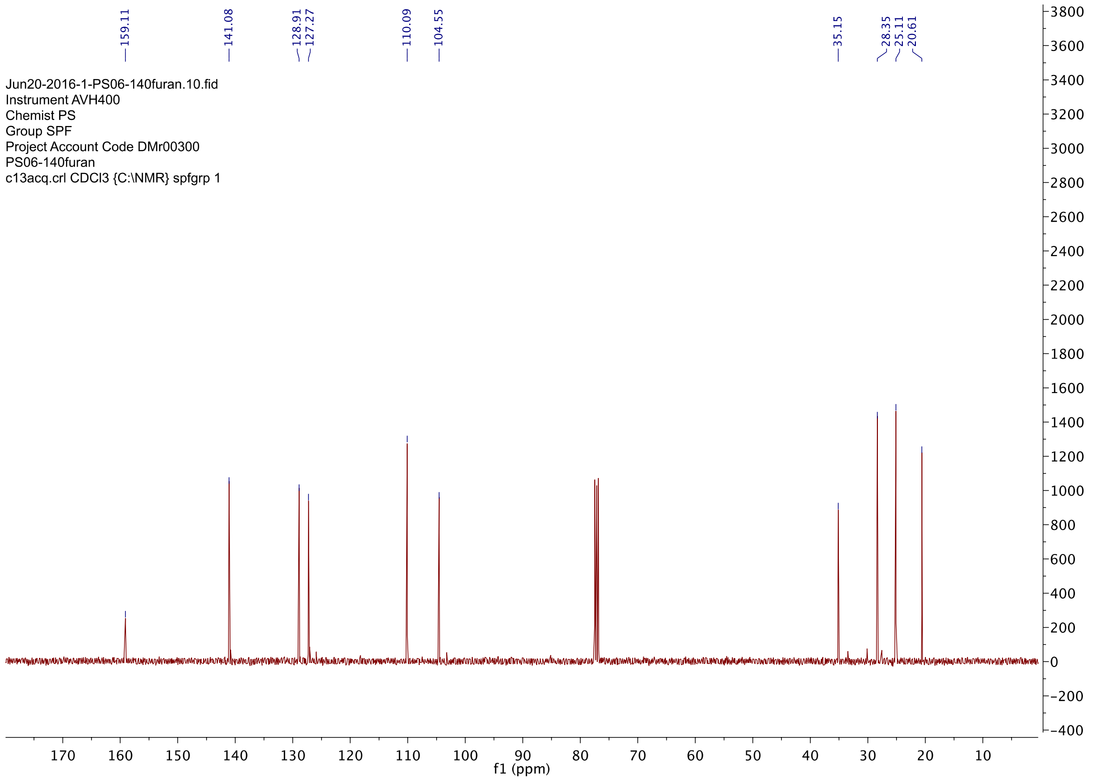

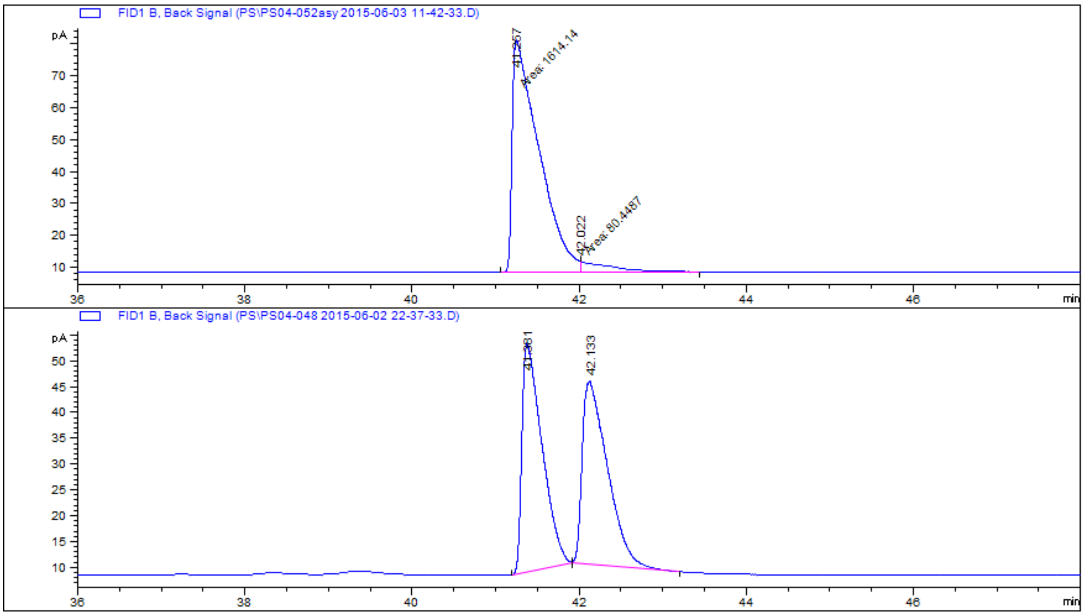

Supplementary figure 23: ^{1}H, ^{13}C-NMR spectra, HPLC traces of compound **22**

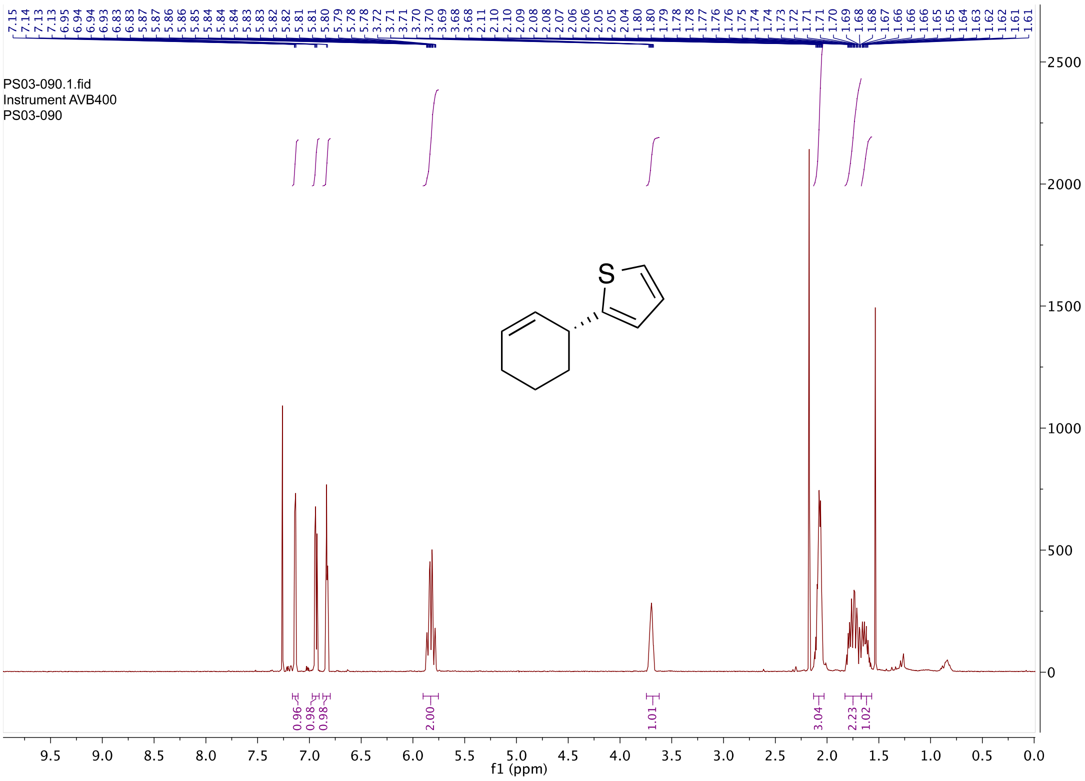

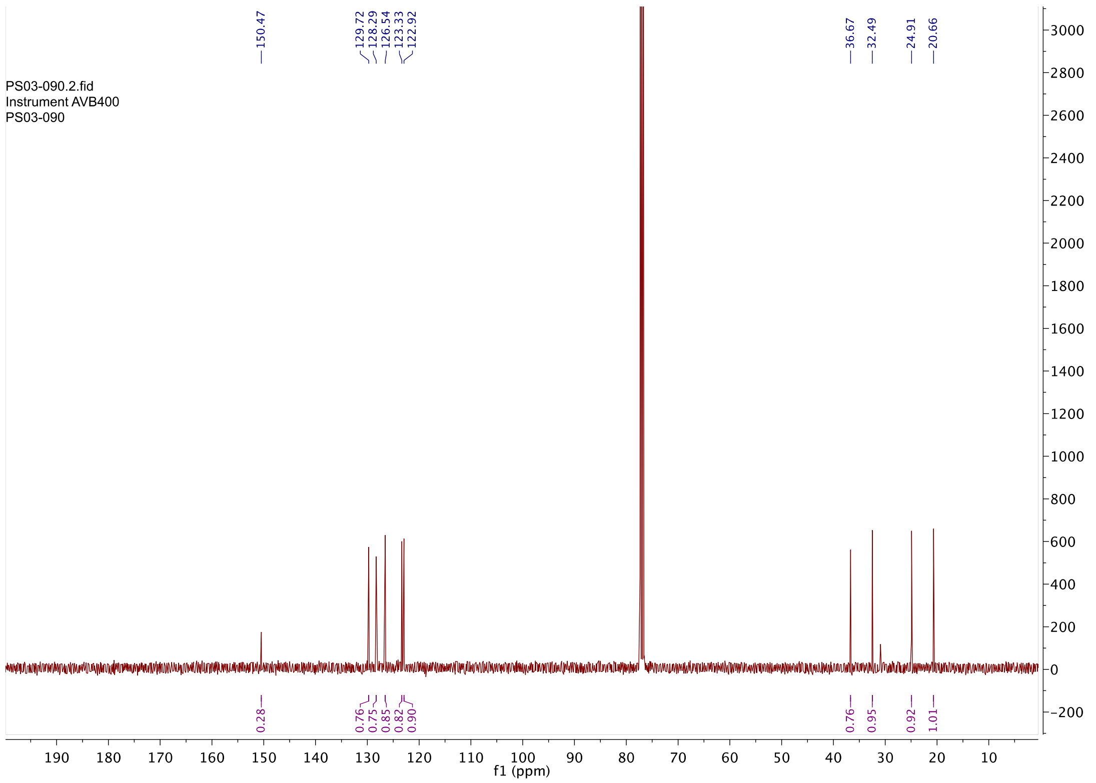

PS03-090.2.fid
Instrument AVB400
PS03-090
150.47
129.72
128.29
126.54
123.33
122.92
36.67
32.49
24.91
20.66
0.28
0.76
0.75
0.85
0.82
0.90
0.76
0.95
0.92
1.01
f1 (ppm)

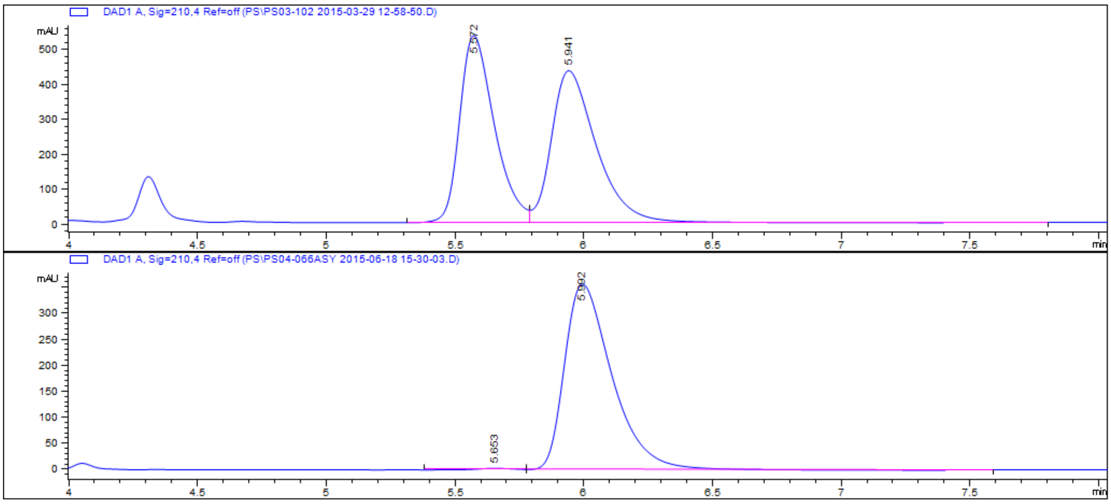

Supplementary figure 24: ^{1}H, ^{13}C-NMR spectra of compound **23**

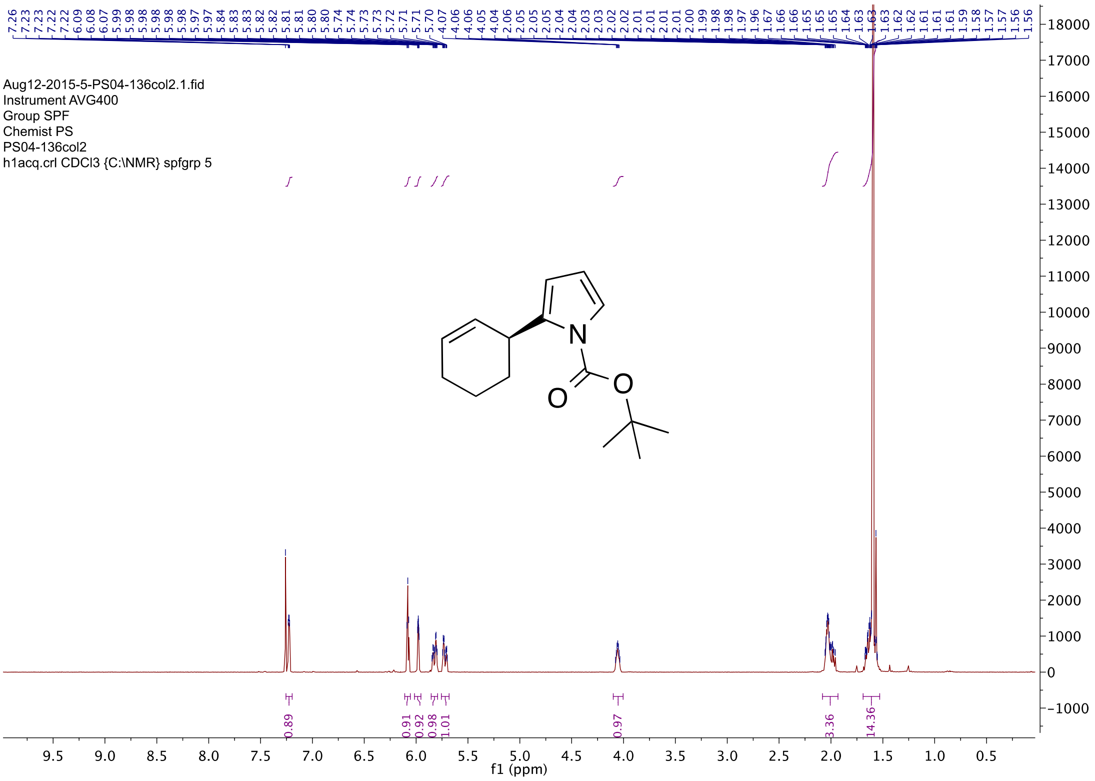

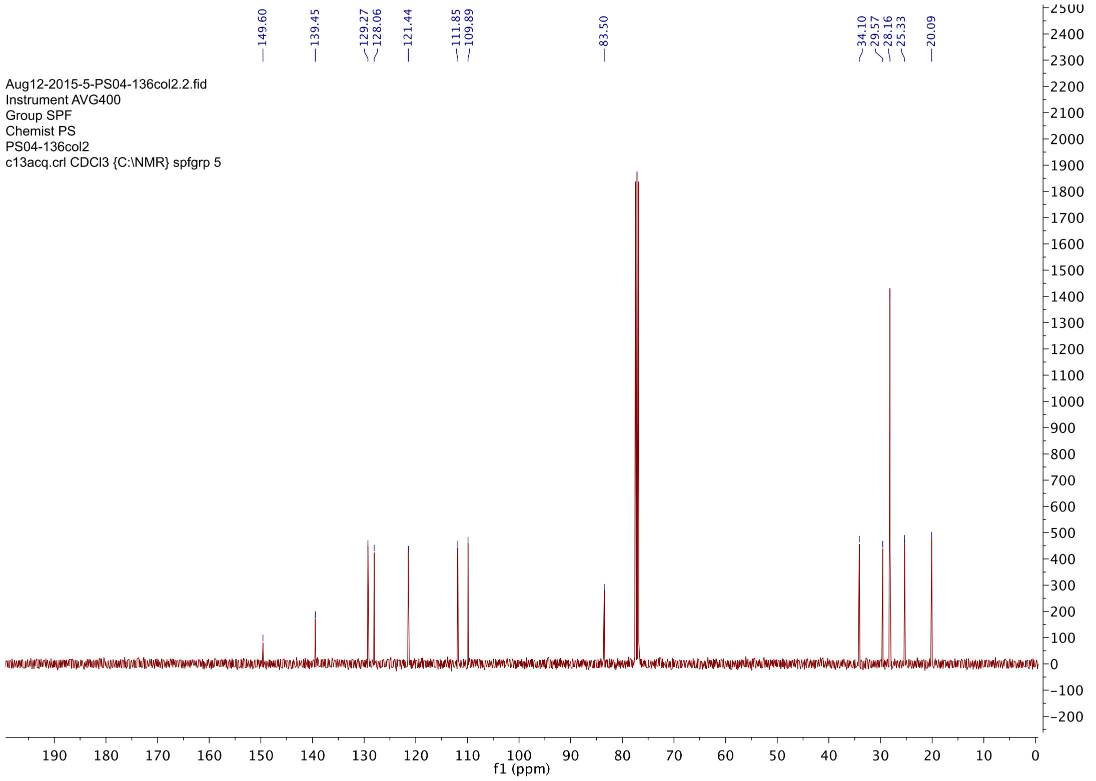

Aug12-2015-5-PS04-136col2.2.fid
Instrument AVG400
Group SPF
Chemist PS
PS04-136col2
c13acq.crl CDCl3 {C:\NMR} spfgrp 5
149.60
139.45
129.27
128.06
121.44
111.85
109.89
83.50
34.10
29.57
28.16
25.33
20.09
f1 (ppm)

Supplementary figure 25: ^{1}H, ^{13}C-NMR spectra, HPLC traces of compound **23a**

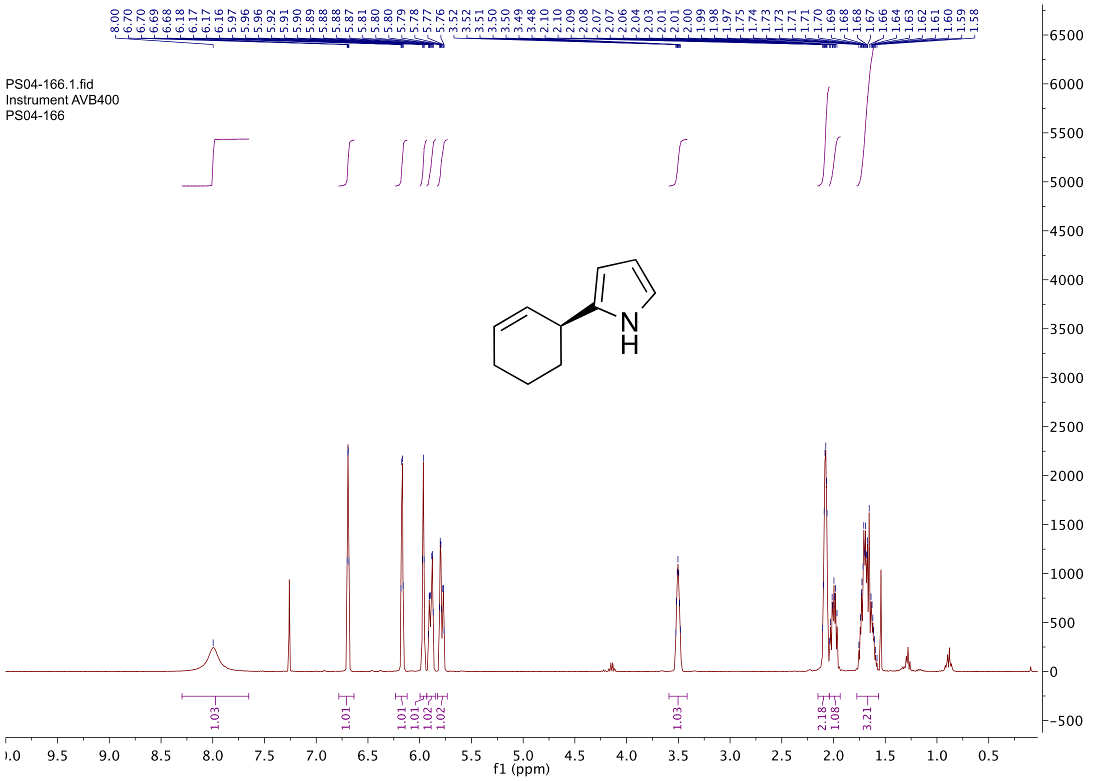

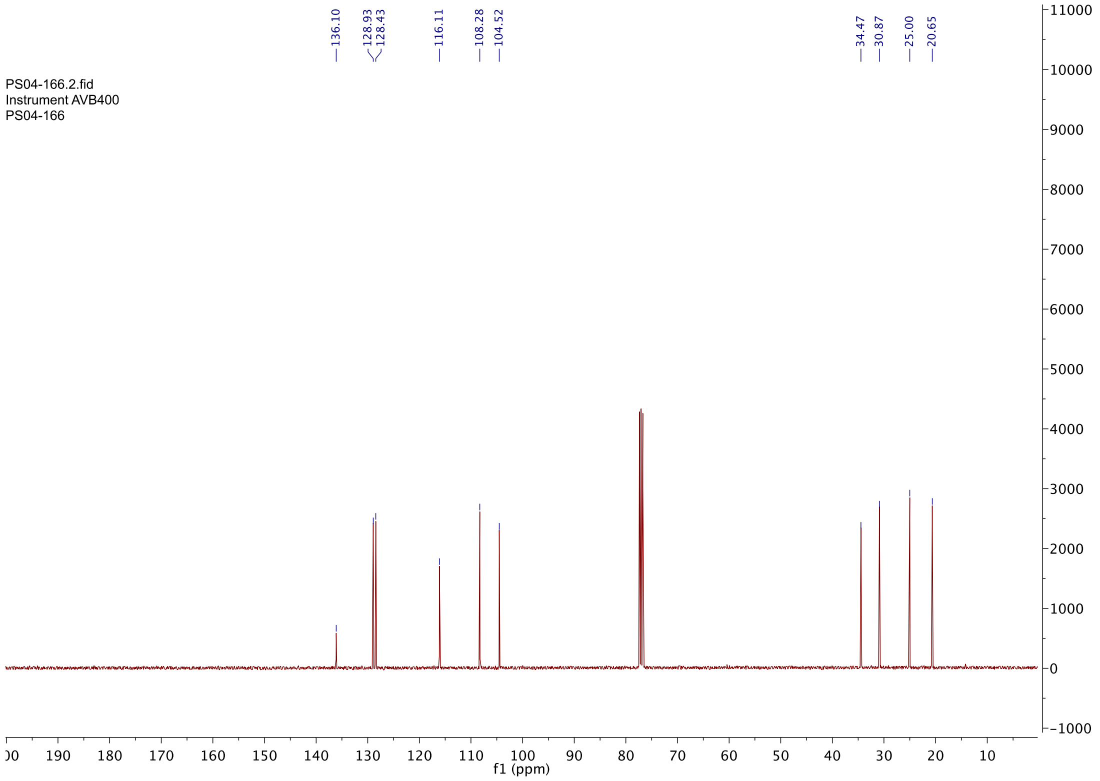

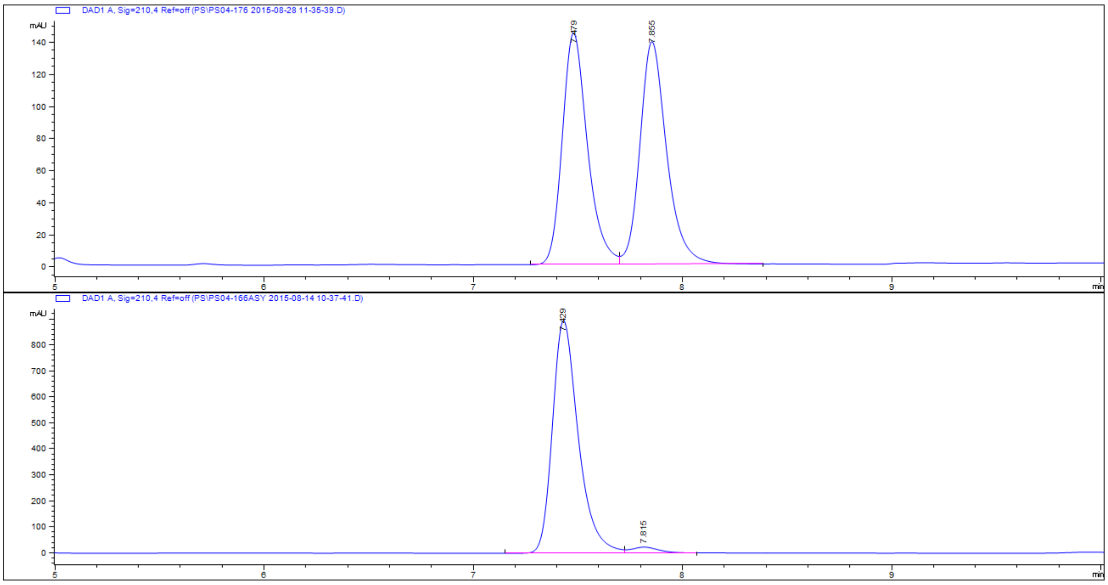

Supplementary figure 26: ^{1}H, ^{13}C-NMR spectra, GC traces of compound **24**

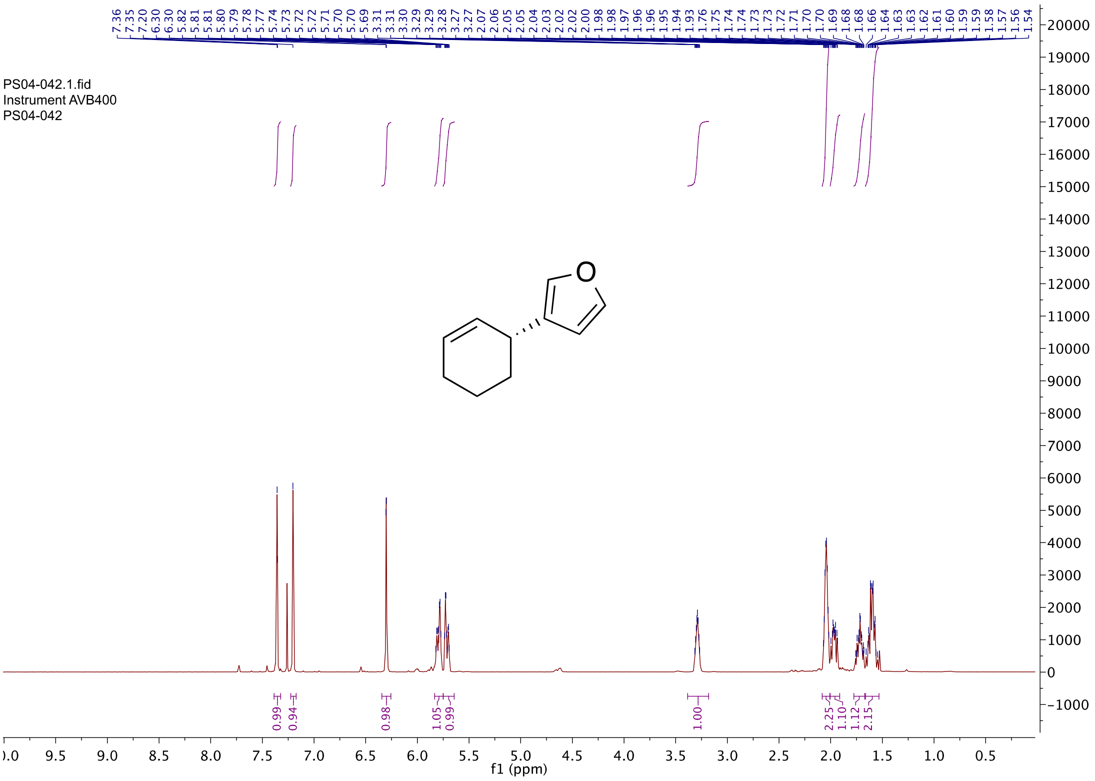

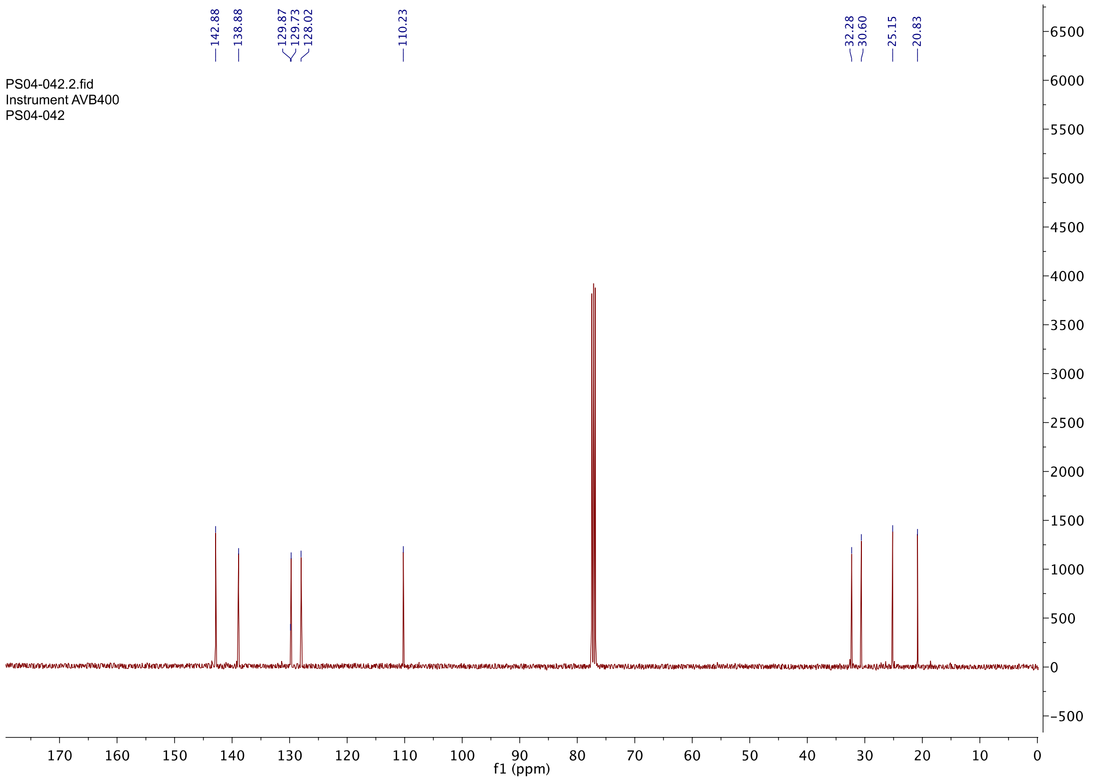
142.88
138.88
129.87
129.73
128.02
110.23
32.28
30.60
25.15
20.83
PS04-042.2.fid
Instrument AVB400
PS04-042
f1 (ppm)

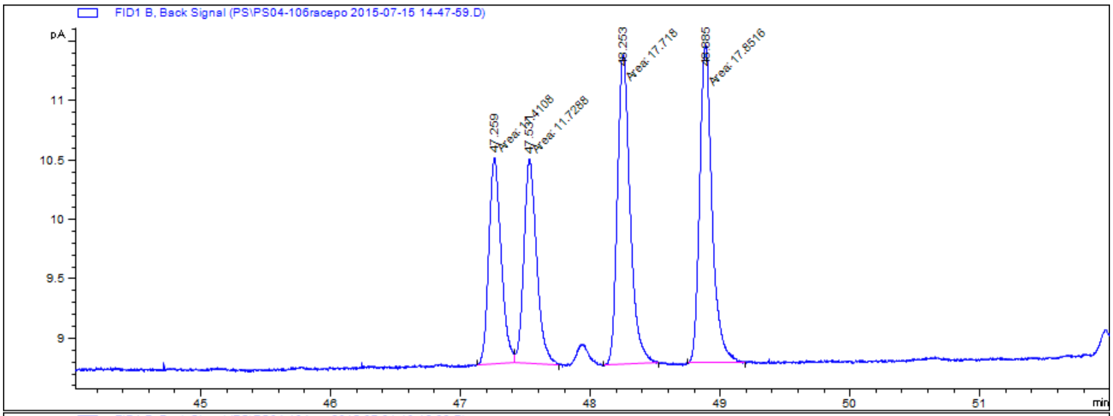
FID1 B, Back Signal (PS\PS04-106racepo 2015-07-15 14-47-59.D)
pA
47.259
47.534
Area: 11.7288
48.253
Area: 17.718
48.885
Area: 17.8516
min

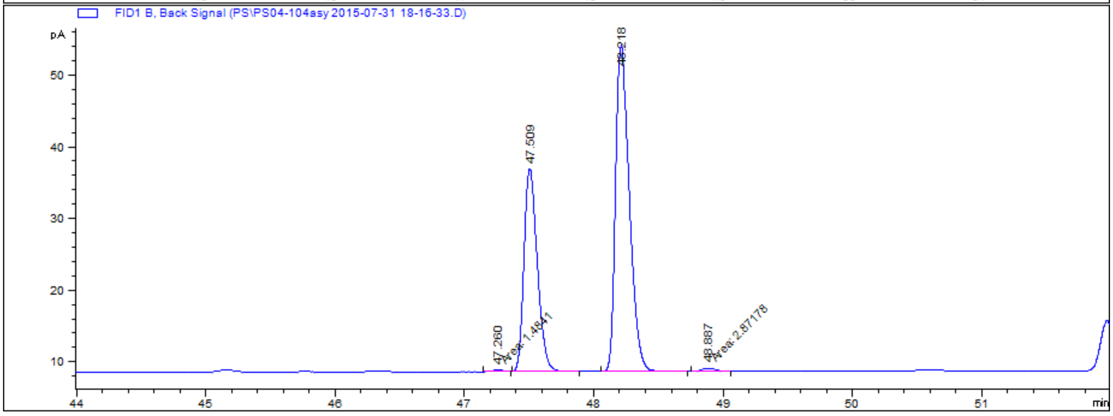
FID1 B, Back Signal (PS\PS04-104asy 2015-07-31 18-16-33.D)
pA
47.260
47.509
Area: 1.4941
48.218
48.887
Area: 2.87178
min

Supplementary figure 27: ^{1}H, ^{13}C-NMR spectra, HPLC traces of compound **25**

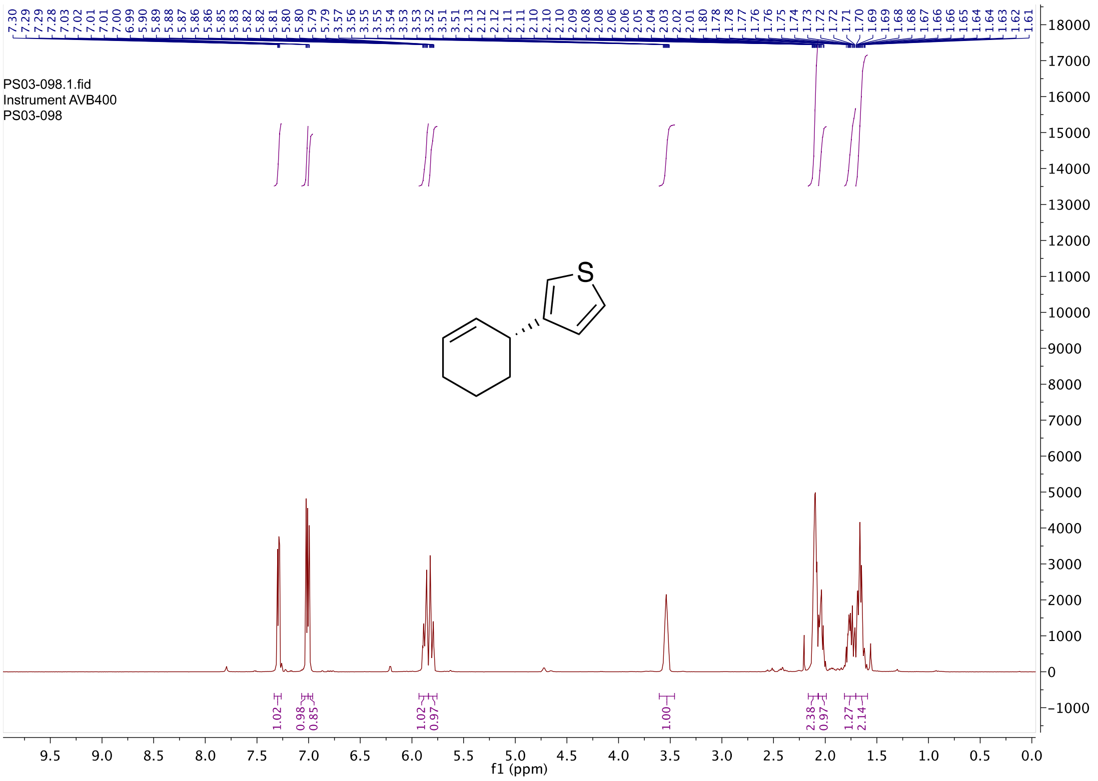

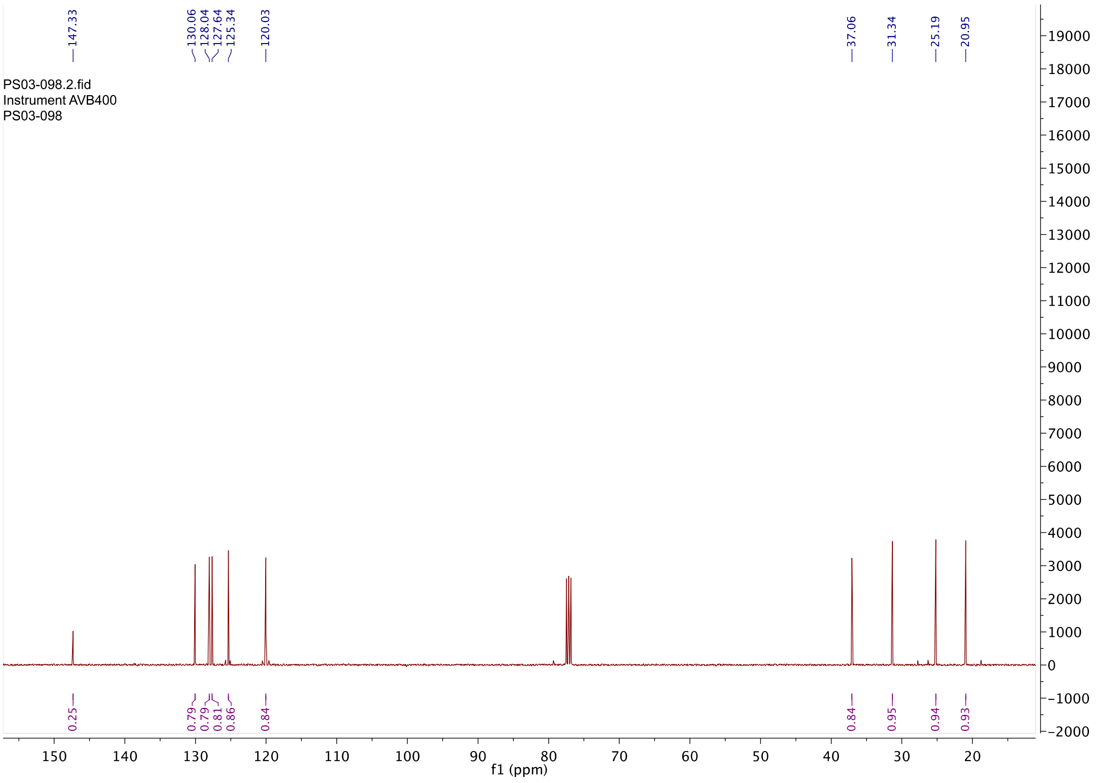

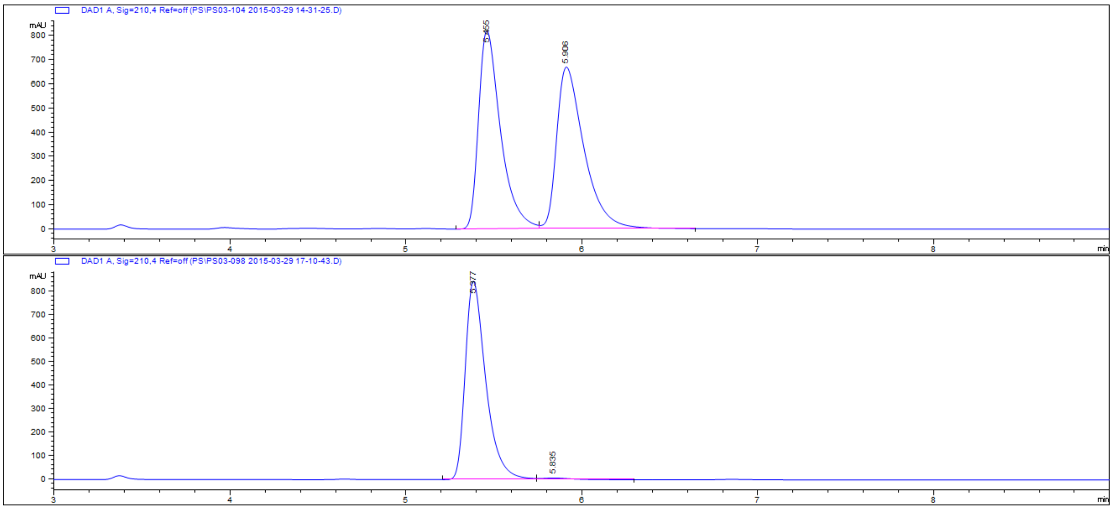

Supplementary figure 28: ^{1}H, ^{13}C-NMR spectra, HPLC traces of compound **27**

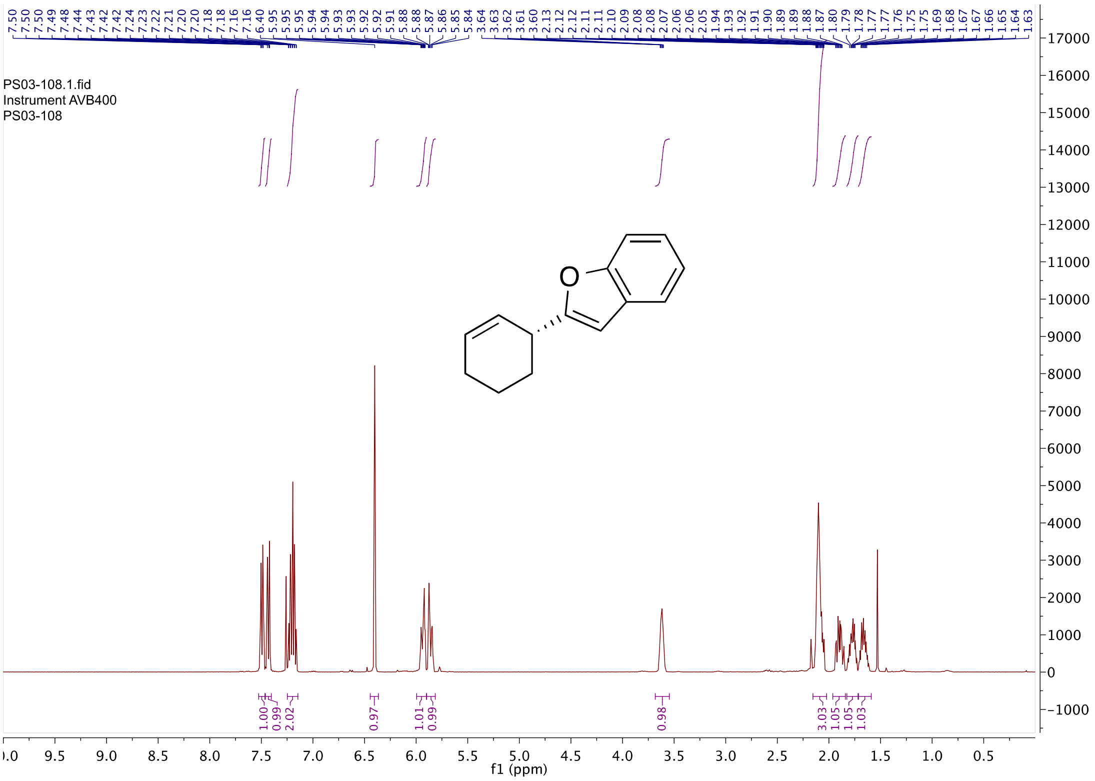

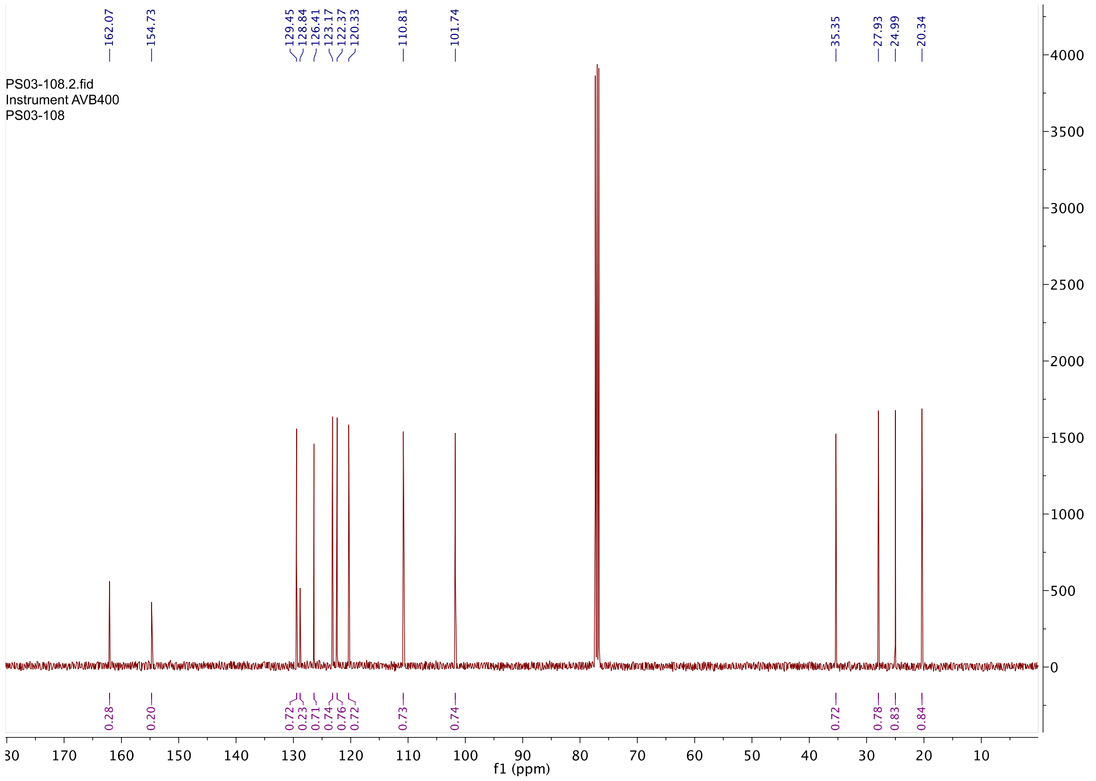

PS03-108.2.fid
Instrument AVB400
PS03-108
162.07
154.73
129.45
128.84
126.41
123.17
122.37
120.33
110.81
101.74
35.35
27.93
24.99
20.34
0.28
0.20
0.72
0.23
0.71
0.74
0.76
0.72
0.73
0.74
0.72
0.78
0.83
0.84
4000
3500
3000
2500
2000
1500
1000
500
0
170
160
150
140
130
120
110
100
90
80
70
60
50
40
30
20
10
f1 (ppm)

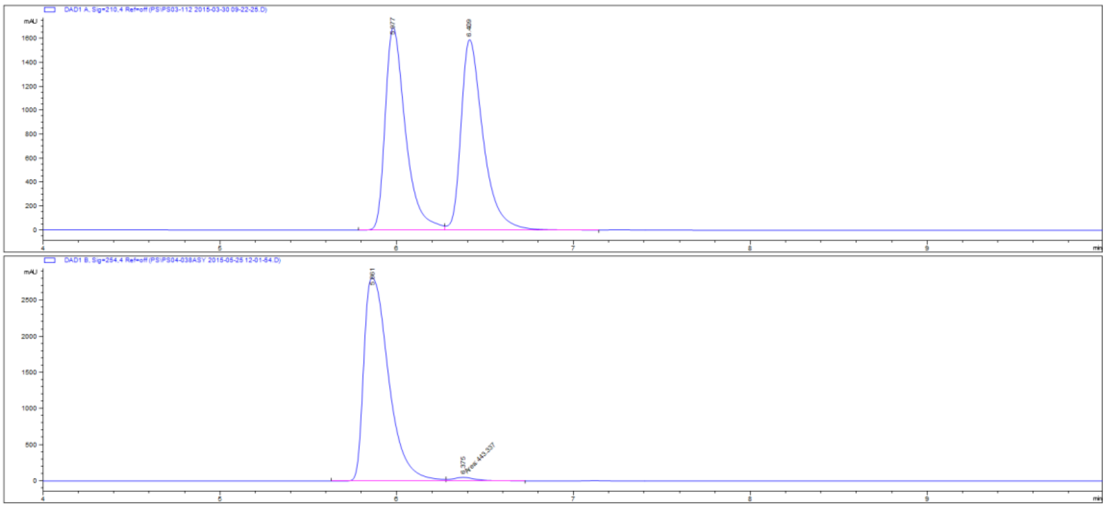

Supplementary figure 29: ^{1}H, ^{13}C-NMR spectra, HPLC traces of compound **28**

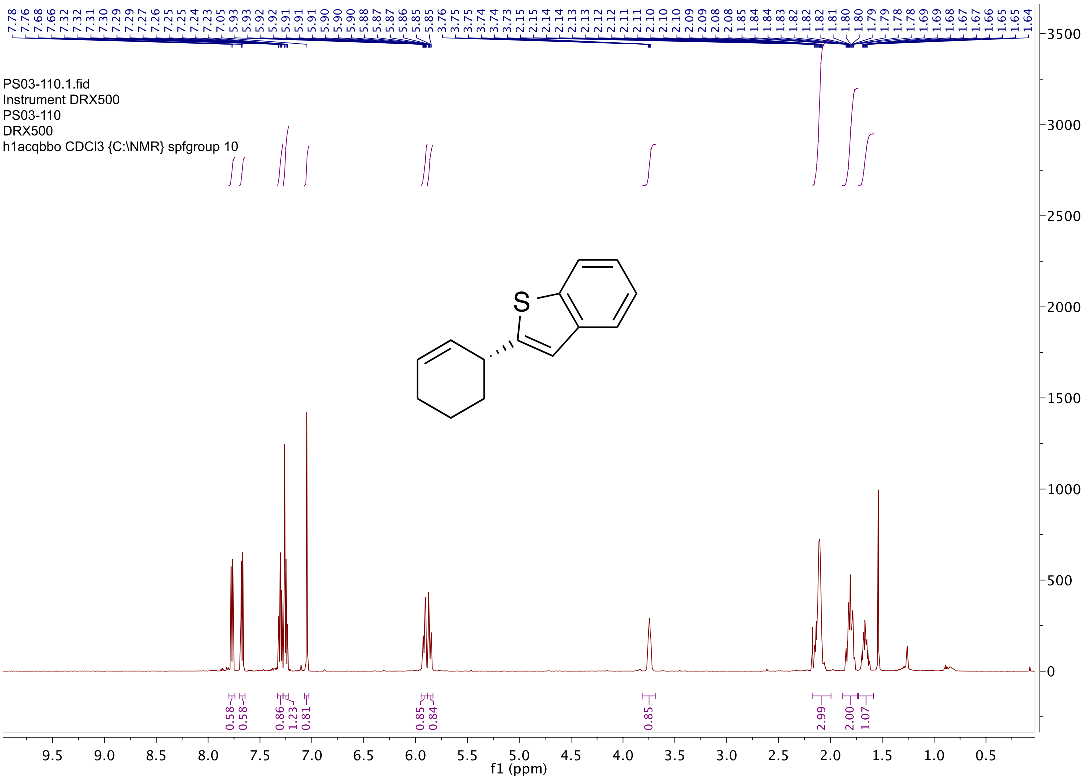

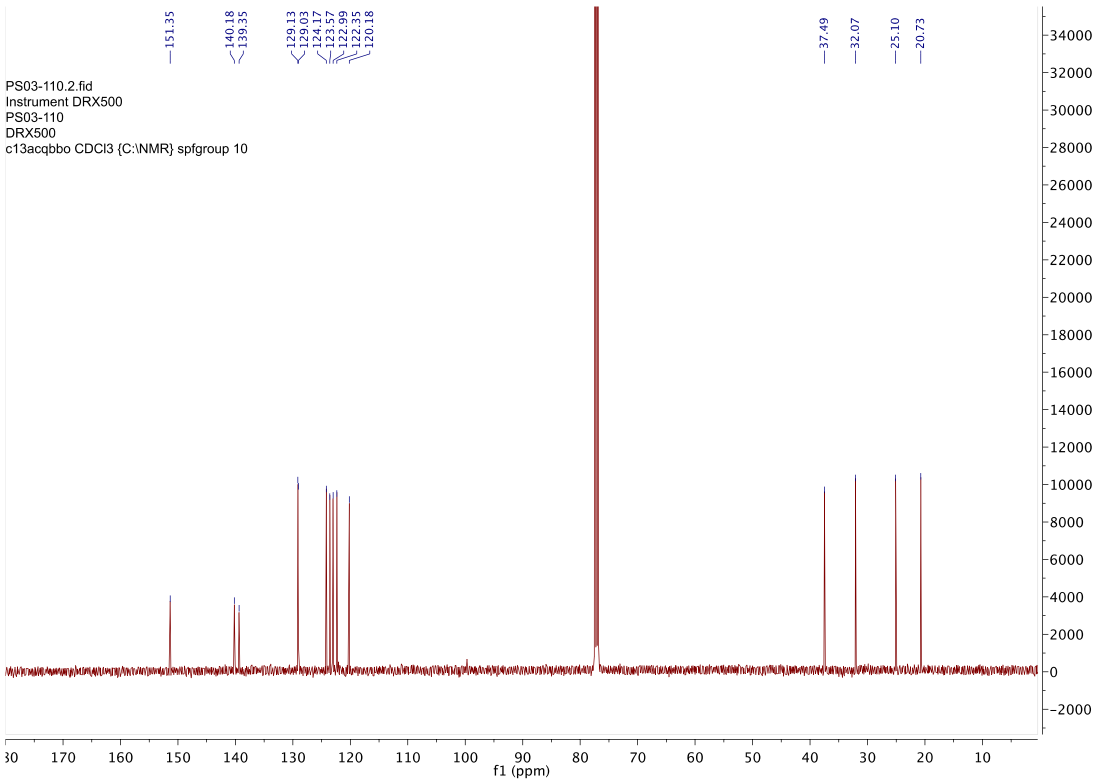

151.35
140.18
139.35
129.13
129.03
124.17
123.57
122.99
122.35
120.18
37.49
32.07
25.10
20.73
PS03-110.2.fid
Instrument DRX500
PS03-110
DRX500
c13acqbbo CDCl3 {C:\NMR} spfgroup 10
f1 (ppm)

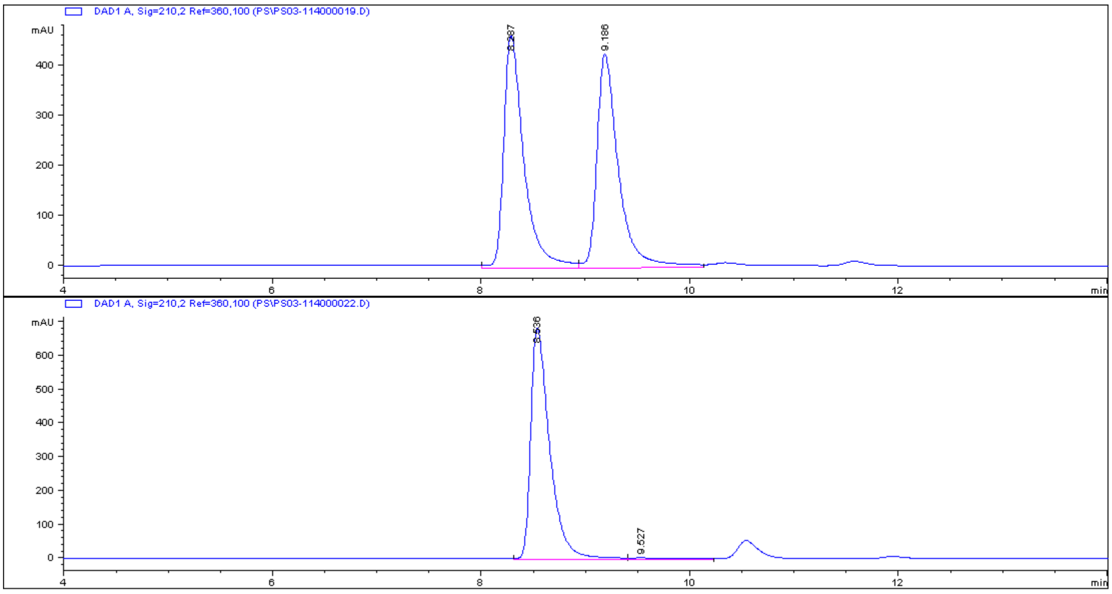

Supplementary figure 30: ^{1}H, ^{13}C-NMR spectra, HPLC traces of compound **29**

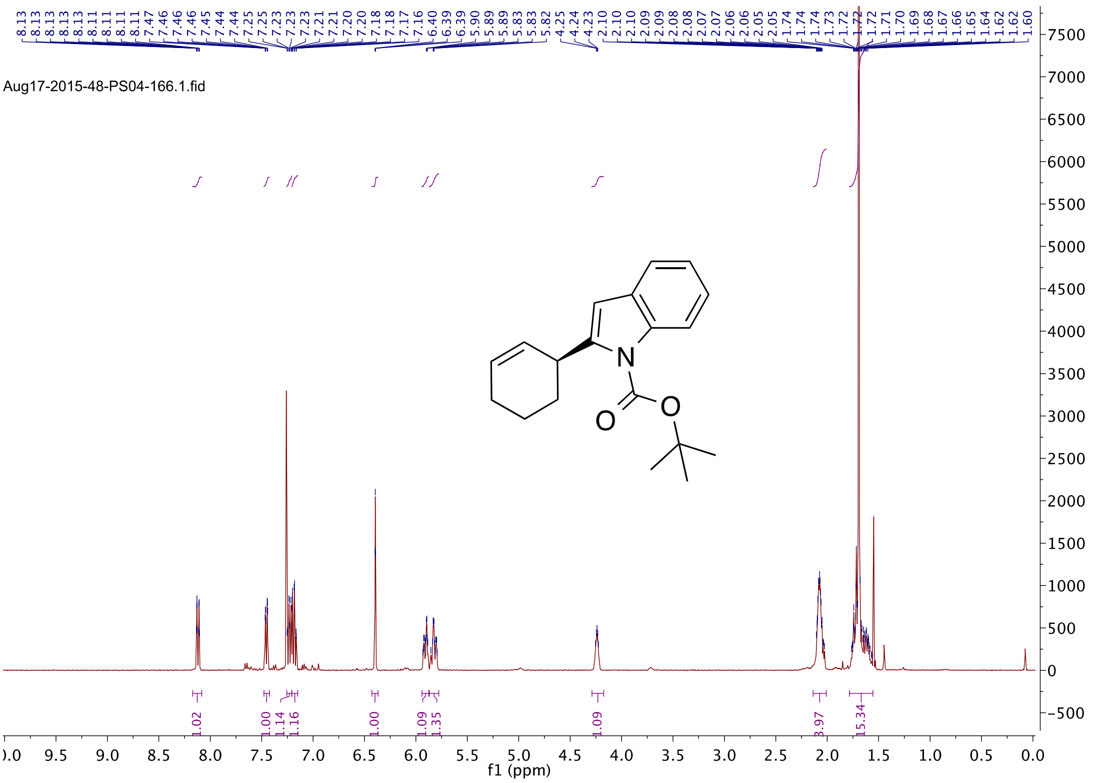

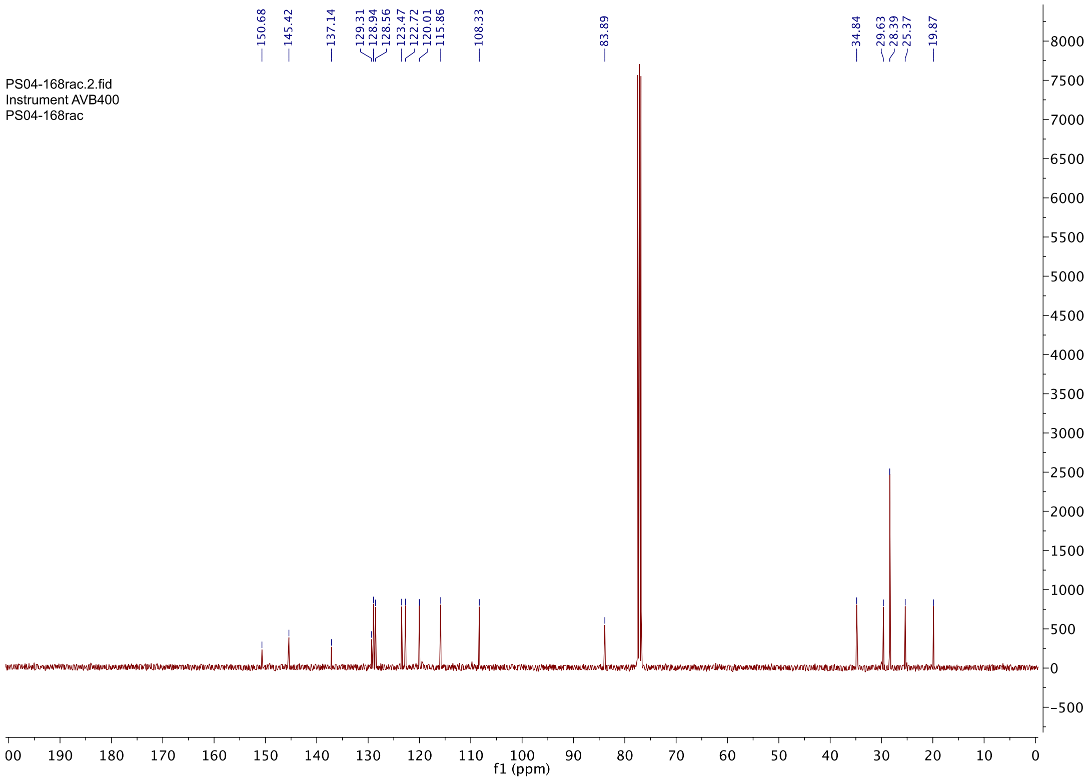
PS04-168rac.2.fid
Instrument AVB400
PS04-168rac
150.68
145.42
137.14
129.31
128.94
128.56
123.47
122.72
120.01
115.86
108.33
83.89
34.84
29.63
28.39
25.37
19.87
f1 (ppm)

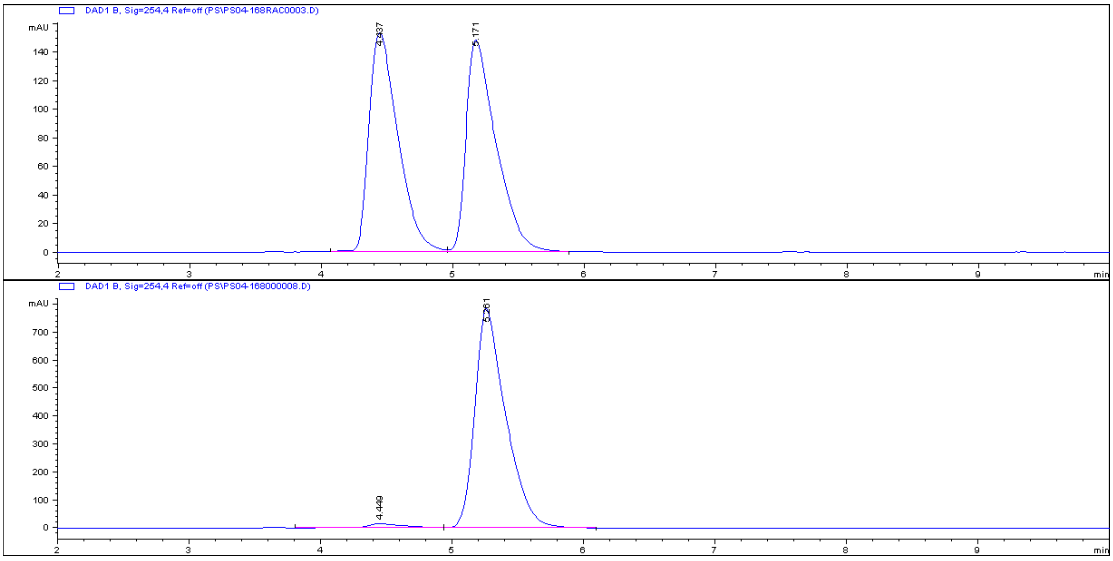
DAD1 B, Sig=254,4 Ref=off (PS\PS04-168RAC0003.D)
mAU
4.437
5.171
min
DAD1 B, Sig=254,4 Ref=off (PS\PS04-168000008.D)
mAU
4.449
5.261
min

Supplementary figure 31: ^{1}H, ^{13}C-NMR spectra, HPLC traces of compound **30**

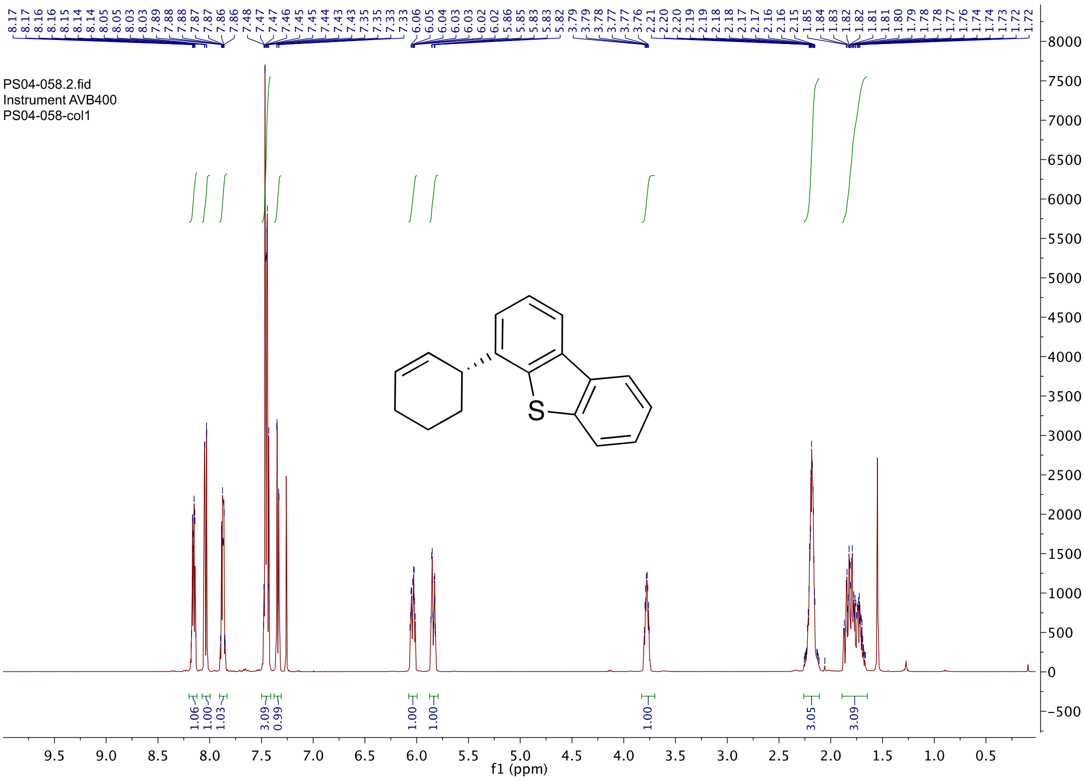

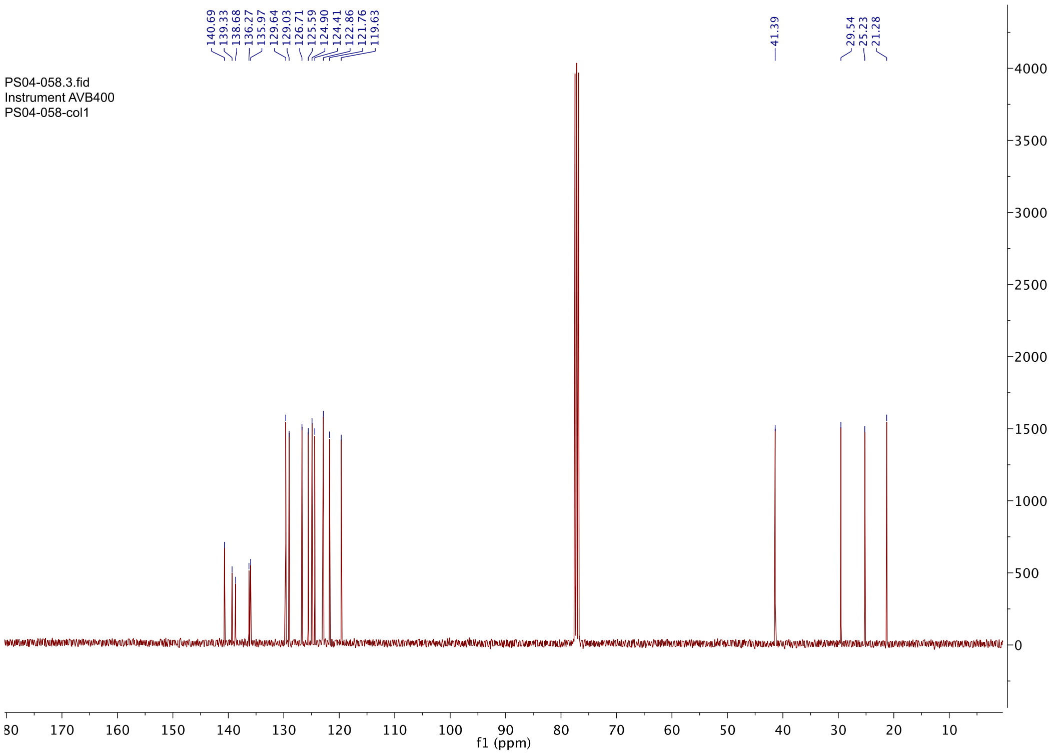

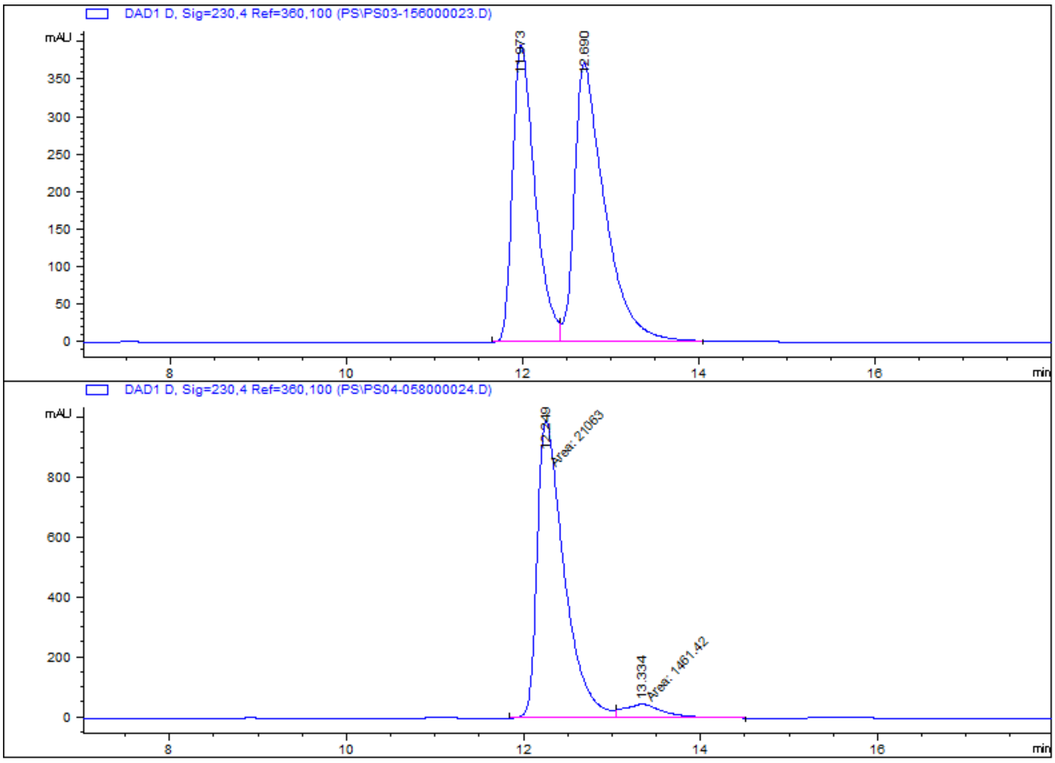

Supplementary figure 32: ^{1}H, ^{13}C-NMR spectra, HPLC traces of compound **33**

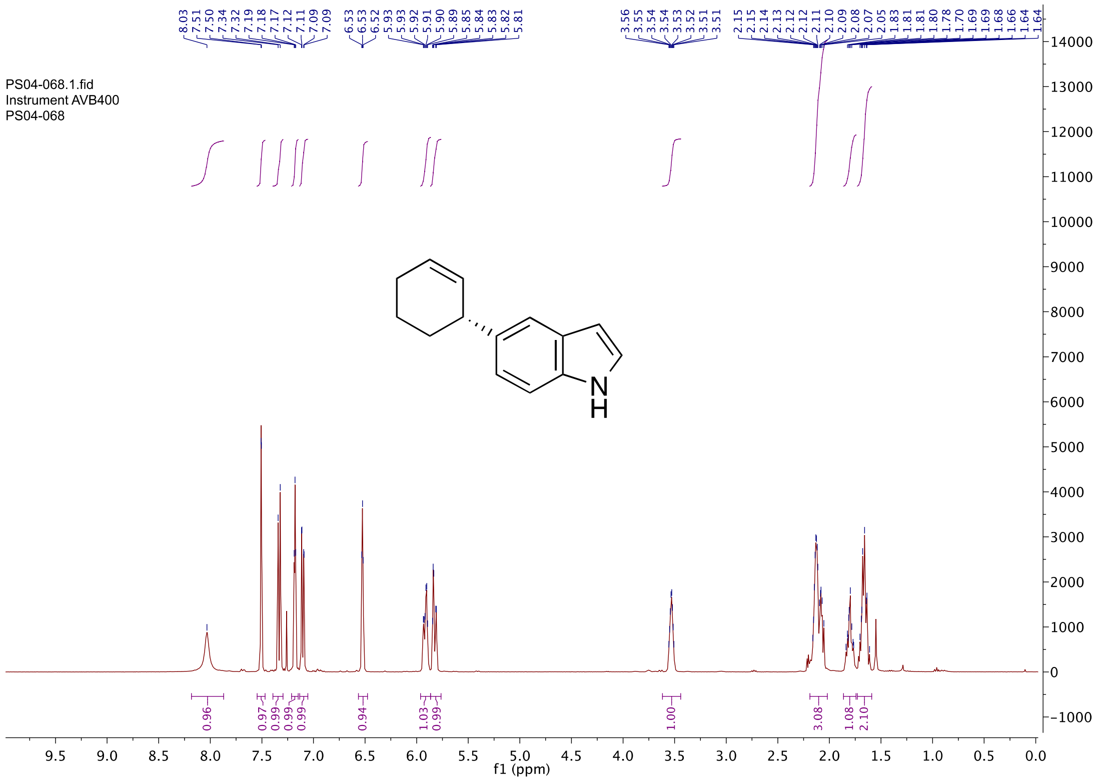

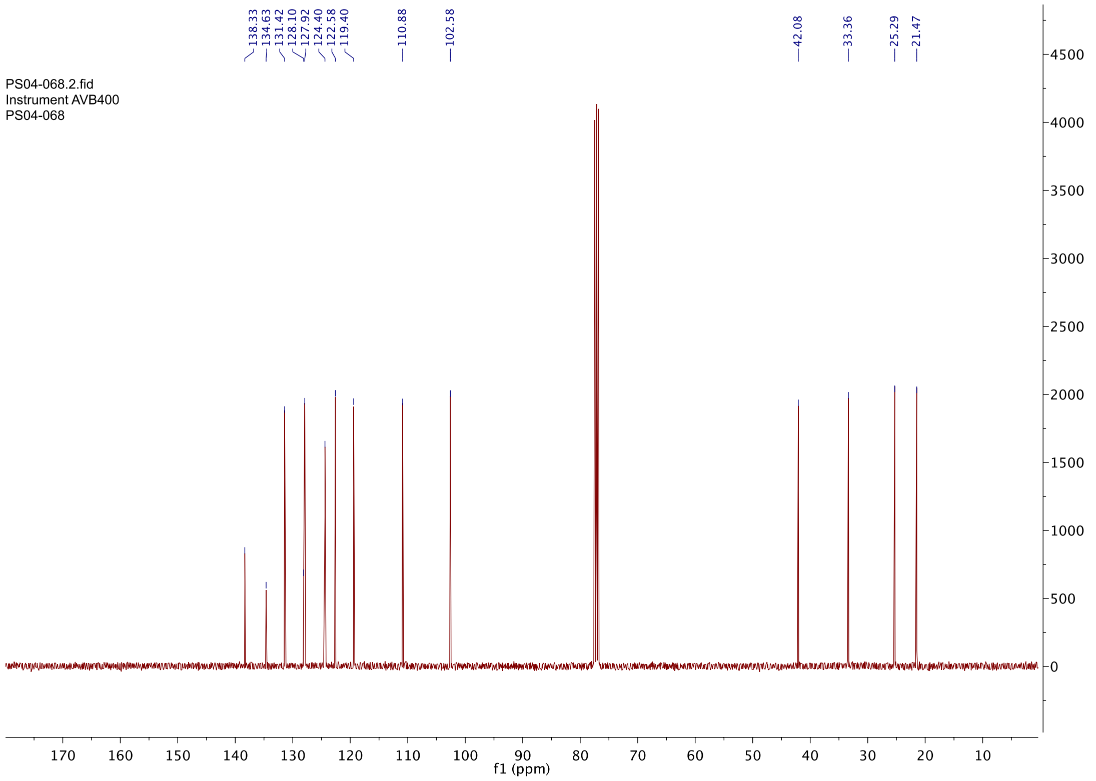

PS04-068.2.fid
Instrument AVB400
PS04-068
138.33
134.63
131.42
128.10
127.92
124.40
122.58
119.40
110.88
102.58
42.08
33.36
25.29
21.47
f1 (ppm)

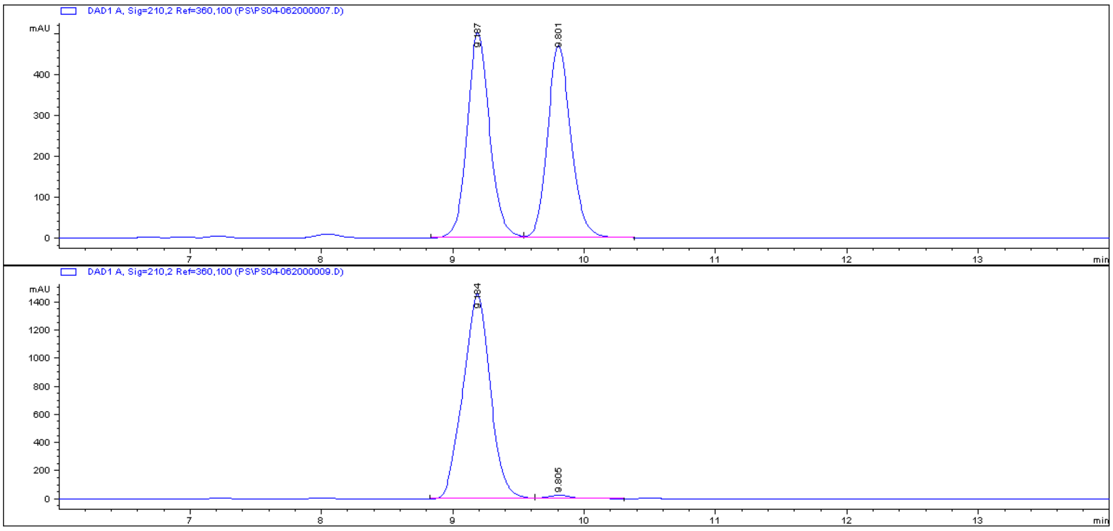

DAD1 A, Sig=210,2 Ref=360,100 (PS\PS04-062000007.D)
mAU
9.187
9.801
min
DAD1 A, Sig=210,2 Ref=360,100 (PS\PS04-062000009.D)
mAU
9.184
9.805
min

Supplementary figure 33: ^{1}H, ^{13}C-NMR spectra, HPLC traces of compound **34**

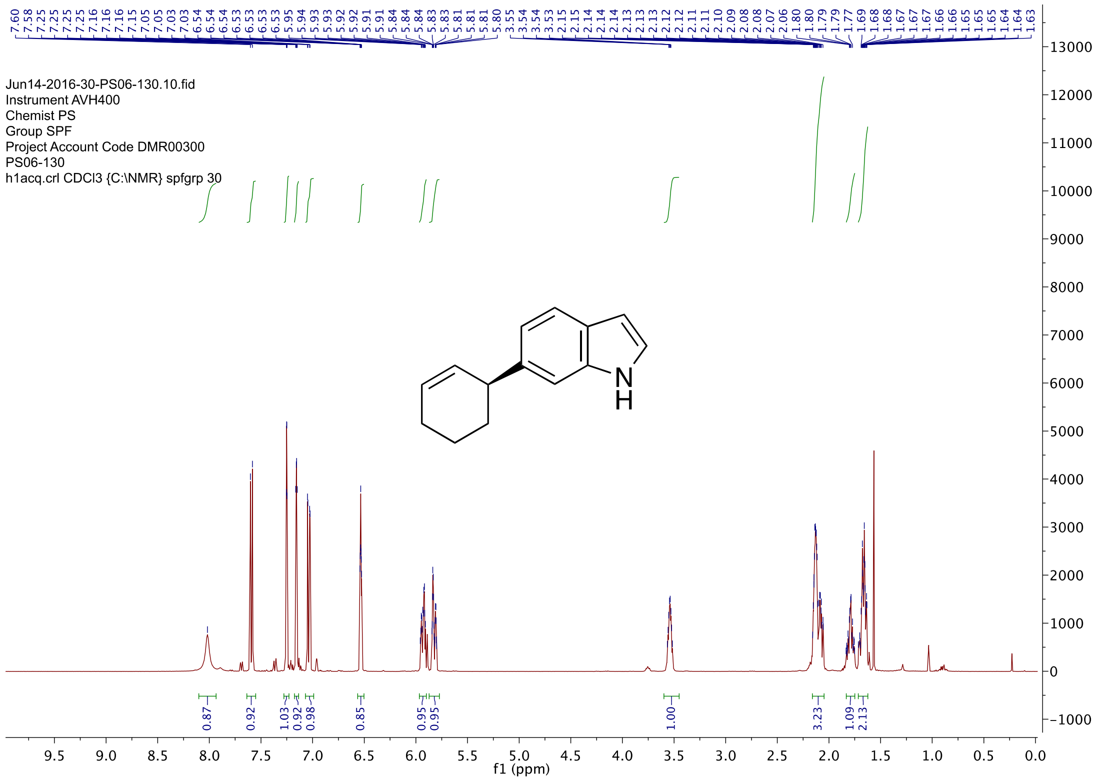

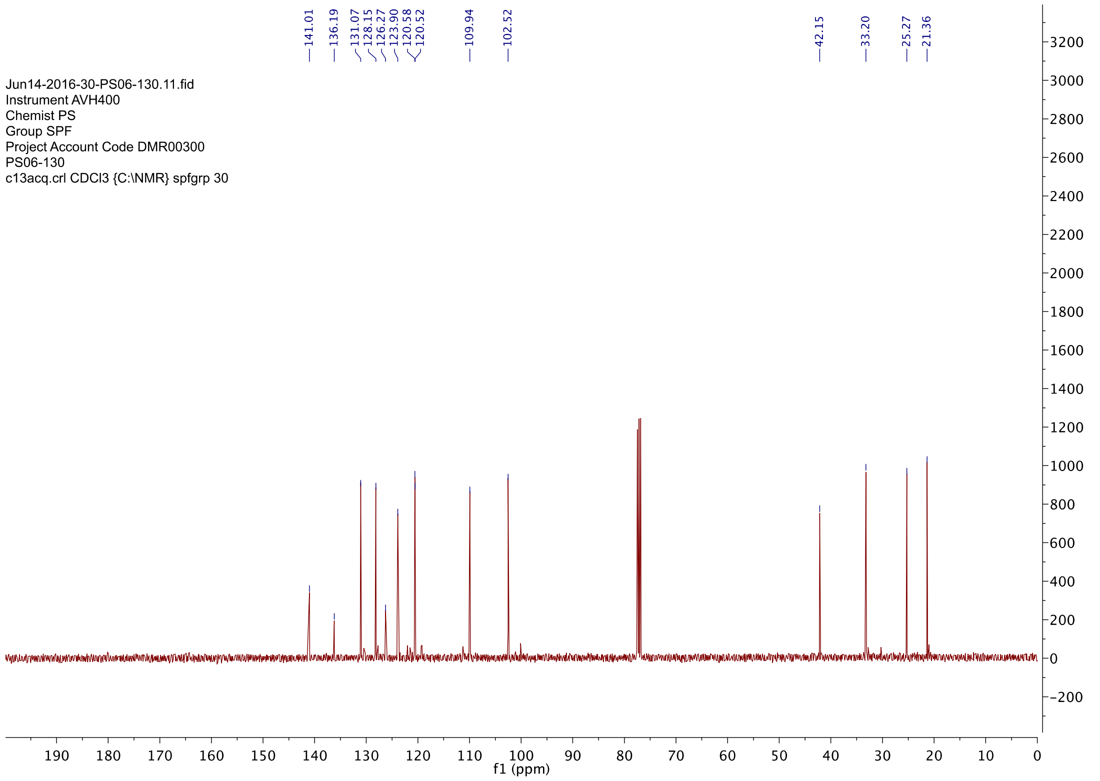

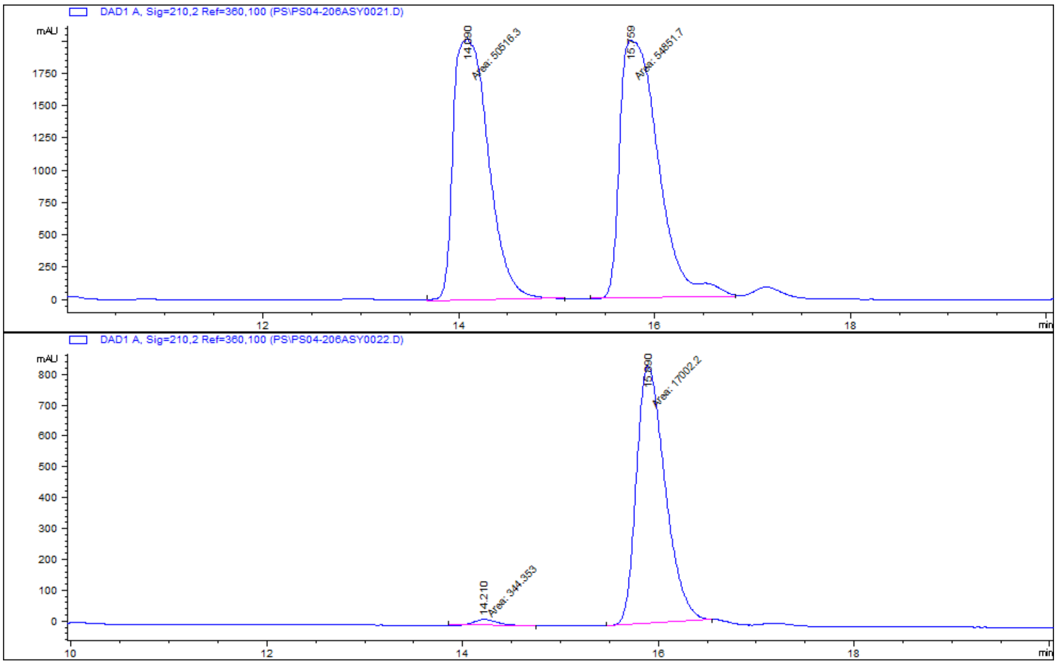

Supplementary figure 34: ^{1}H, ^{13}C-NMR spectra, HPLC traces of compound **36**

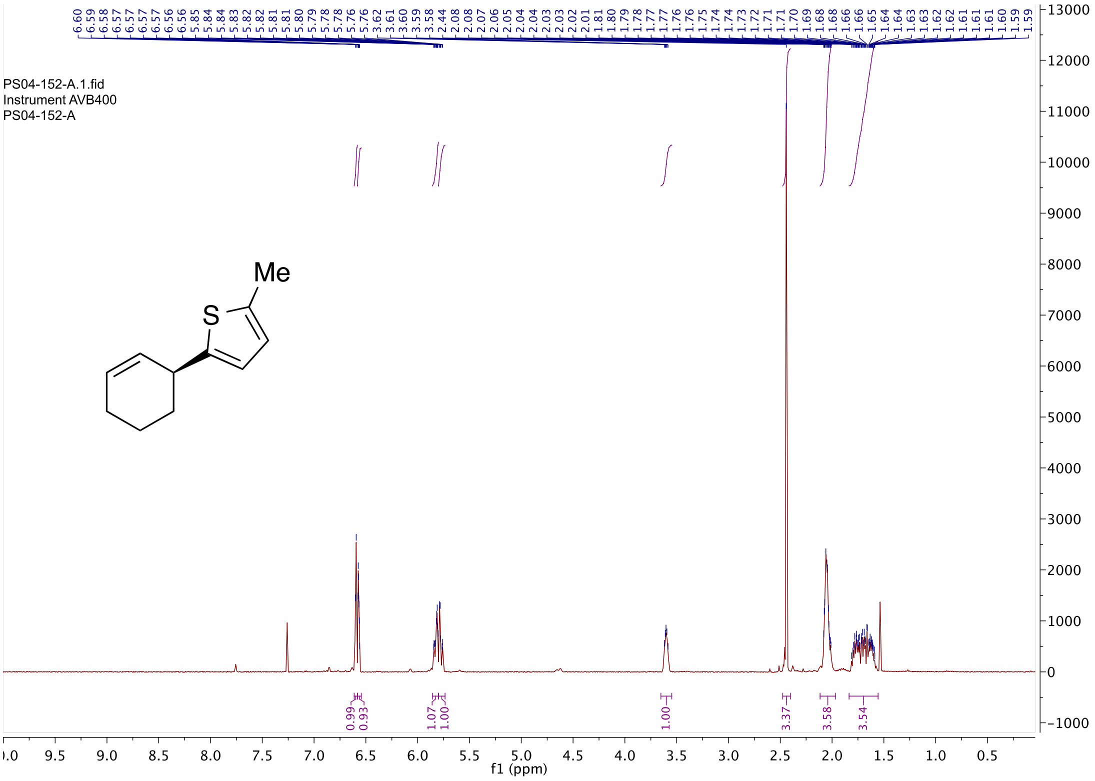

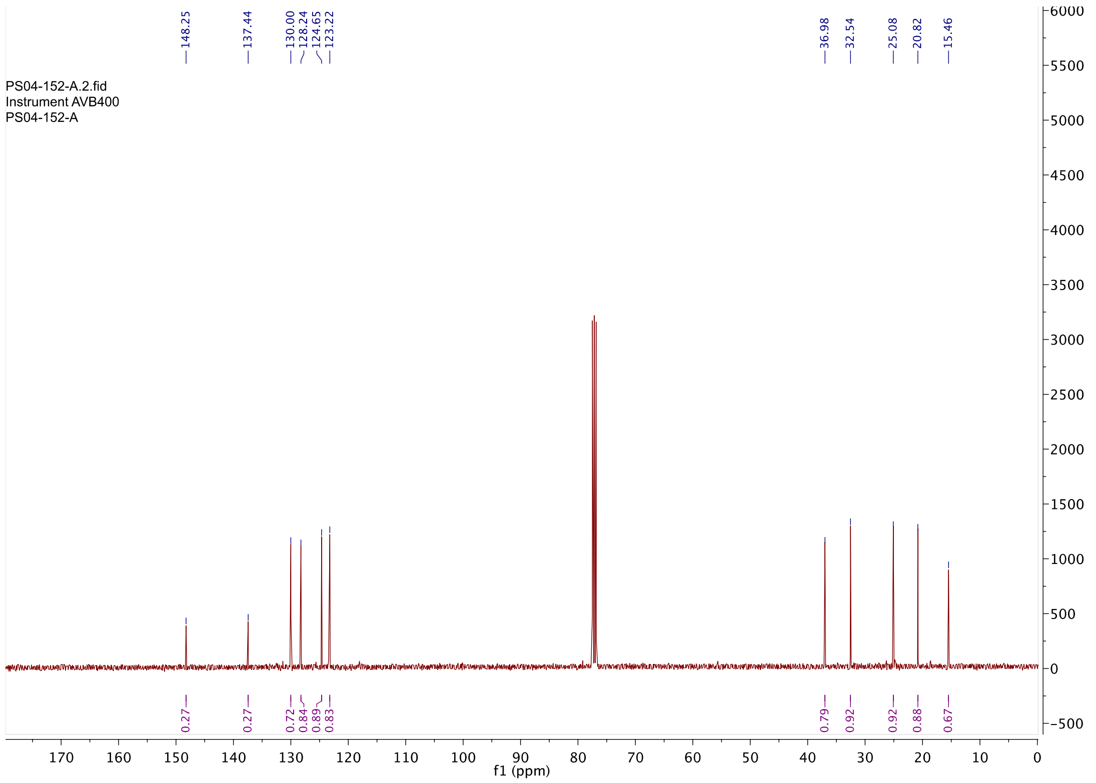

PS04-152-A.2.fid
Instrument AVB400
PS04-152-A
148.25
137.44
130.00
128.24
124.65
123.22
36.98
32.54
25.08
20.82
15.46
0.27
0.27
0.72
0.84
0.89
0.83
0.79
0.92
0.92
0.88
0.67
f1 (ppm)

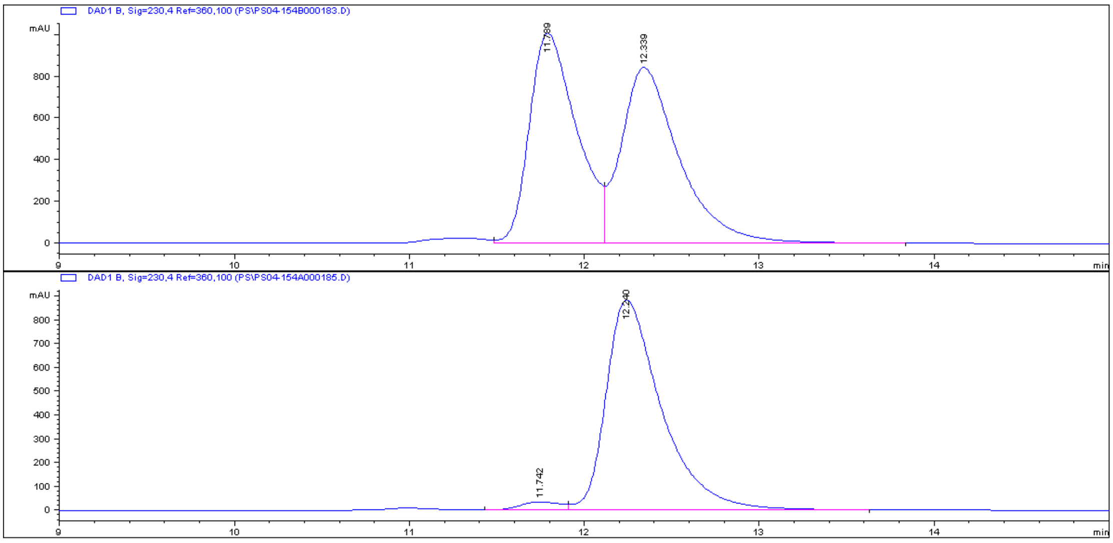

Supplementary figure 35: ^{1}H, ^{13}C-NMR spectra, HPLC traces of compound **37**

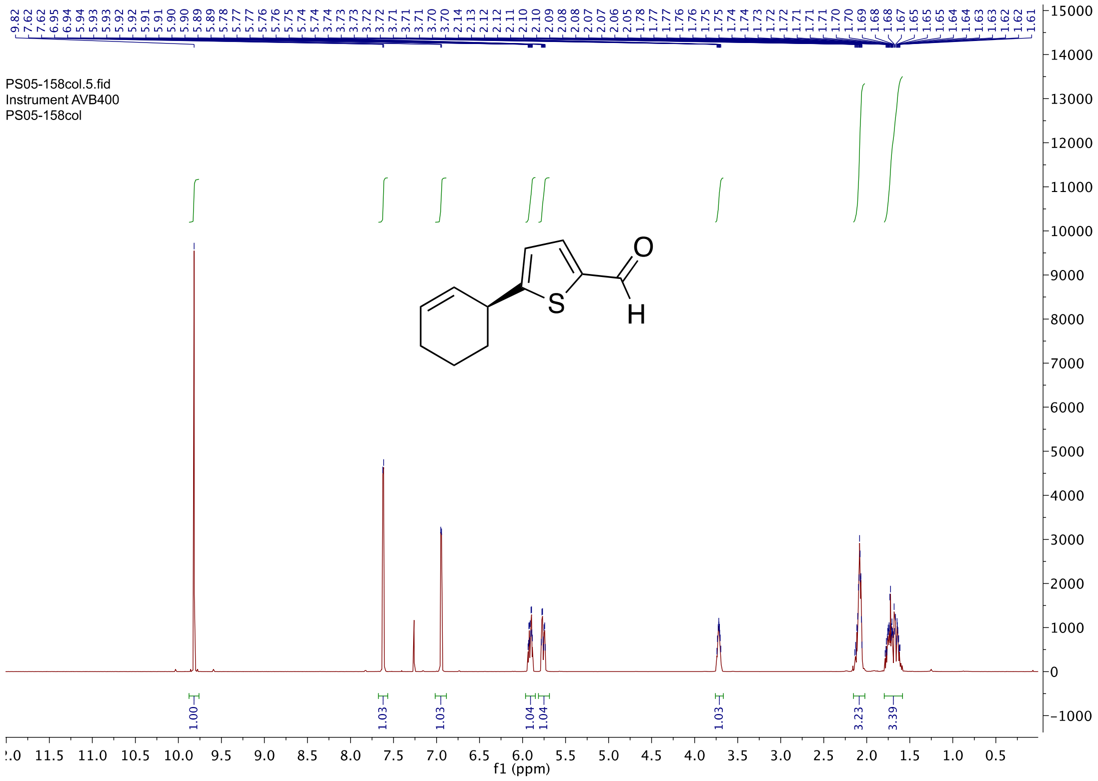

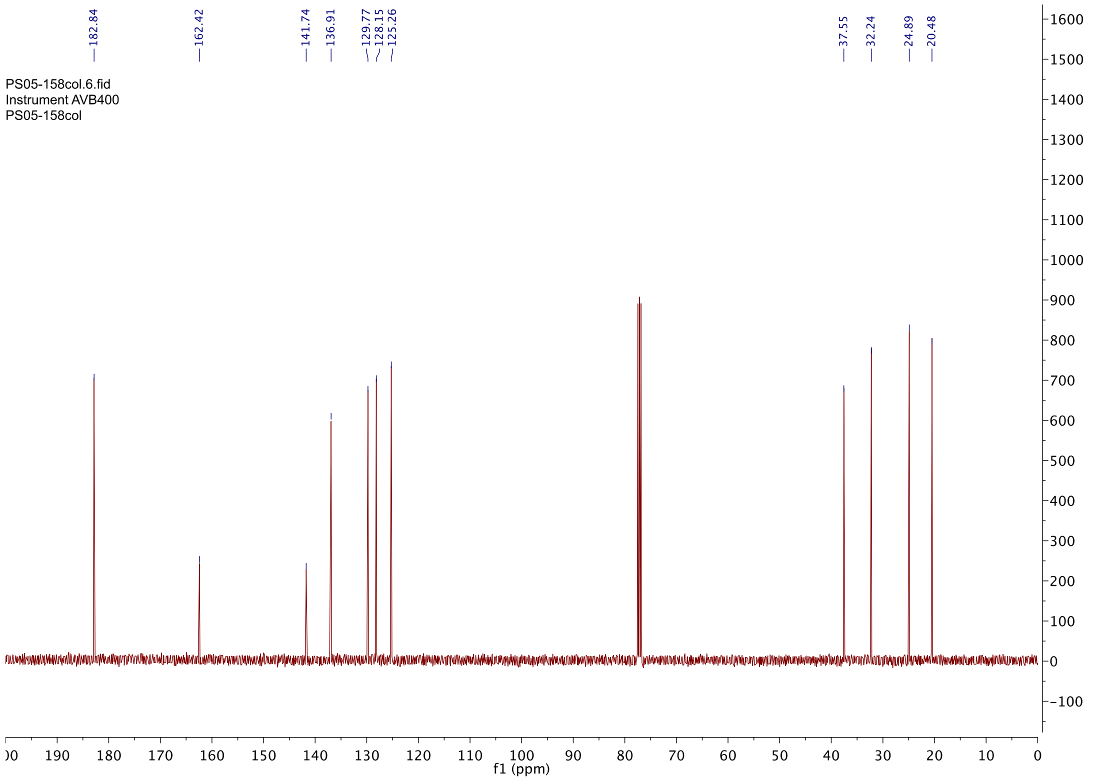

PS05-158col.6.fid
Instrument AVB400
PS05-158col
182.84
162.42
141.74
136.91
129.77
128.15
125.26
37.55
32.24
24.89
20.48
f1 (ppm)

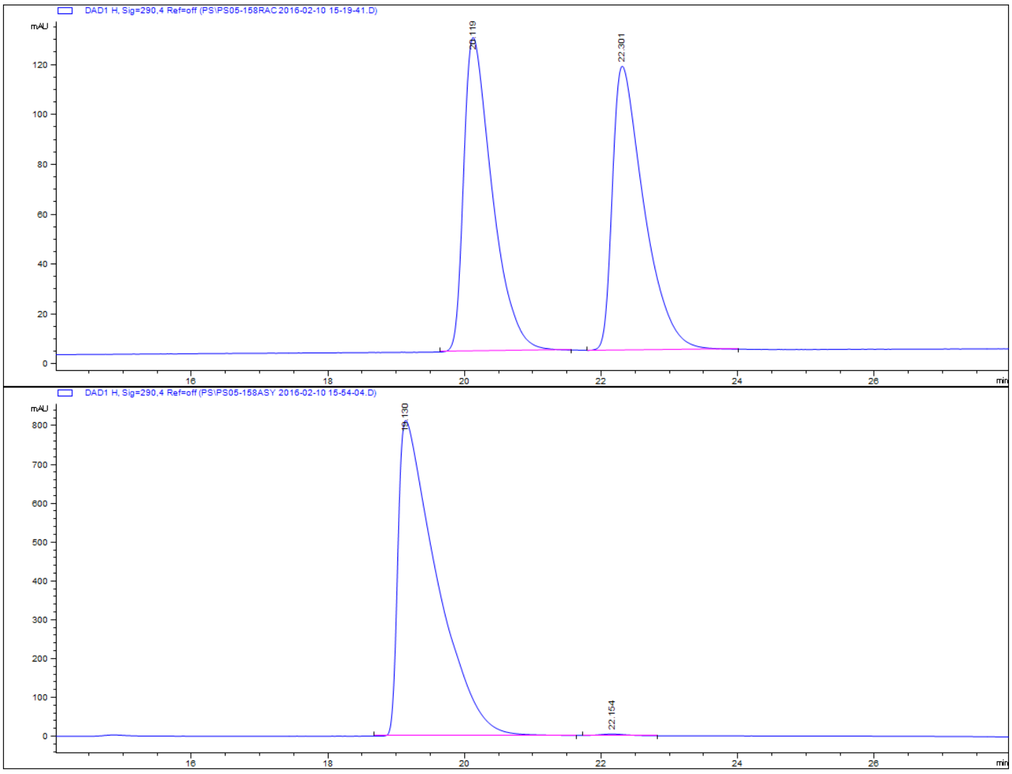

Supplementary figure 36: ^{1}H, ^{13}C-NMR spectra, HPLC traces of compound **38**

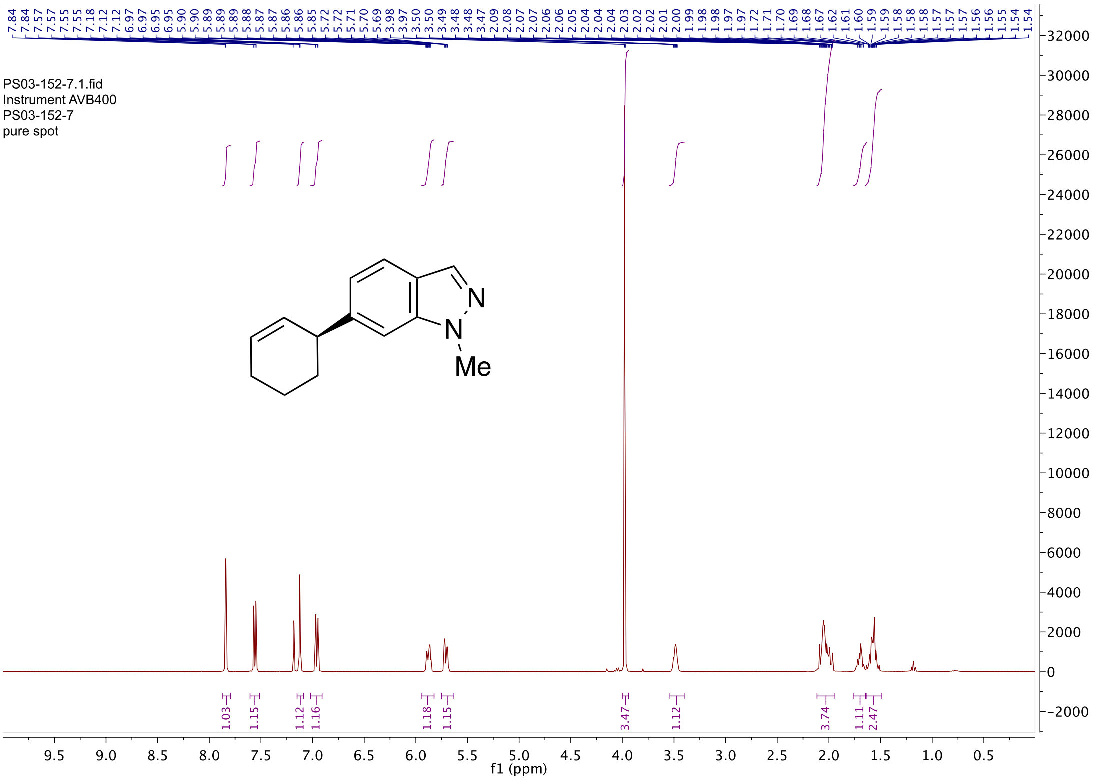

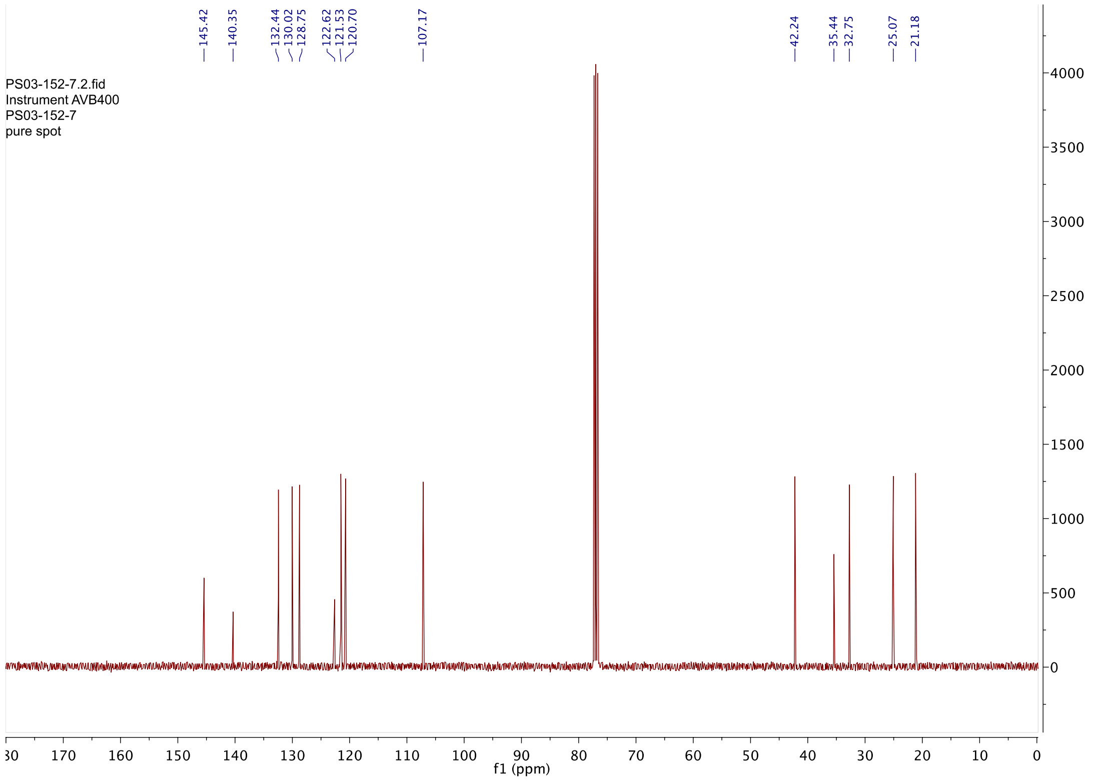
145.42
140.35
132.44
130.02
128.75
122.62
121.53
120.70
107.17
42.24
35.44
32.75
25.07
21.18
PS03-152-7.2.fid
Instrument AVB400
PS03-152-7
pure spot
f1 (ppm)

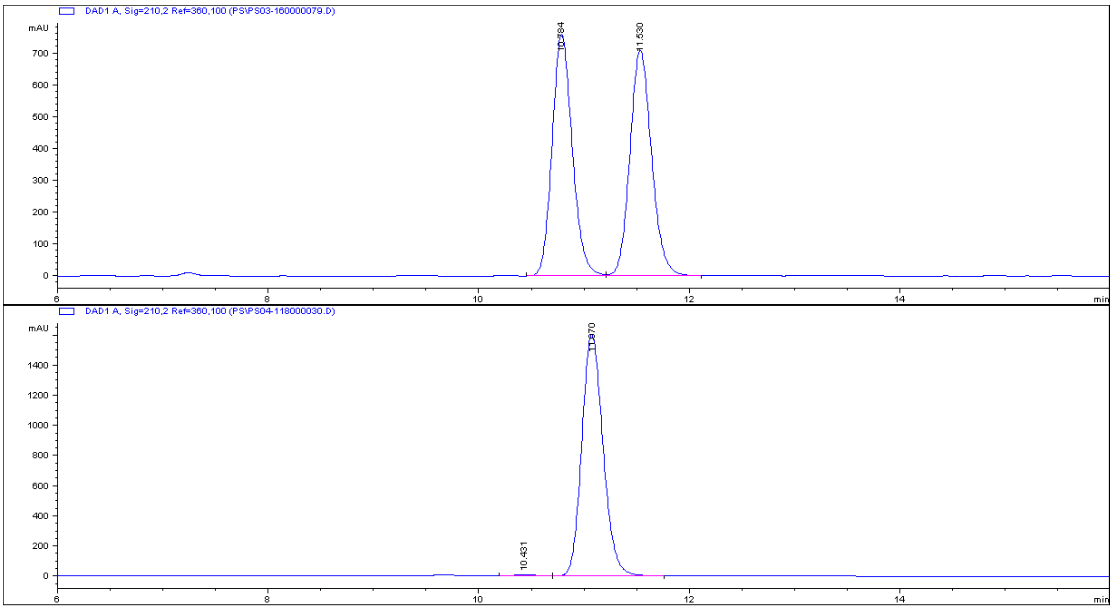
DAD1 A, Sig=210,2 Ref=360,100 (PS\PS03-160000079.D)
mAU
10.784
11.530
min
DAD1 A, Sig=210,2 Ref=360,100 (PS\PS04-118000030.D)
mAU
10.431
11.070
min

Supplementary figure 37: ^{1}H, ^{13}C-NMR spectra, HPLC traces of compound **39**

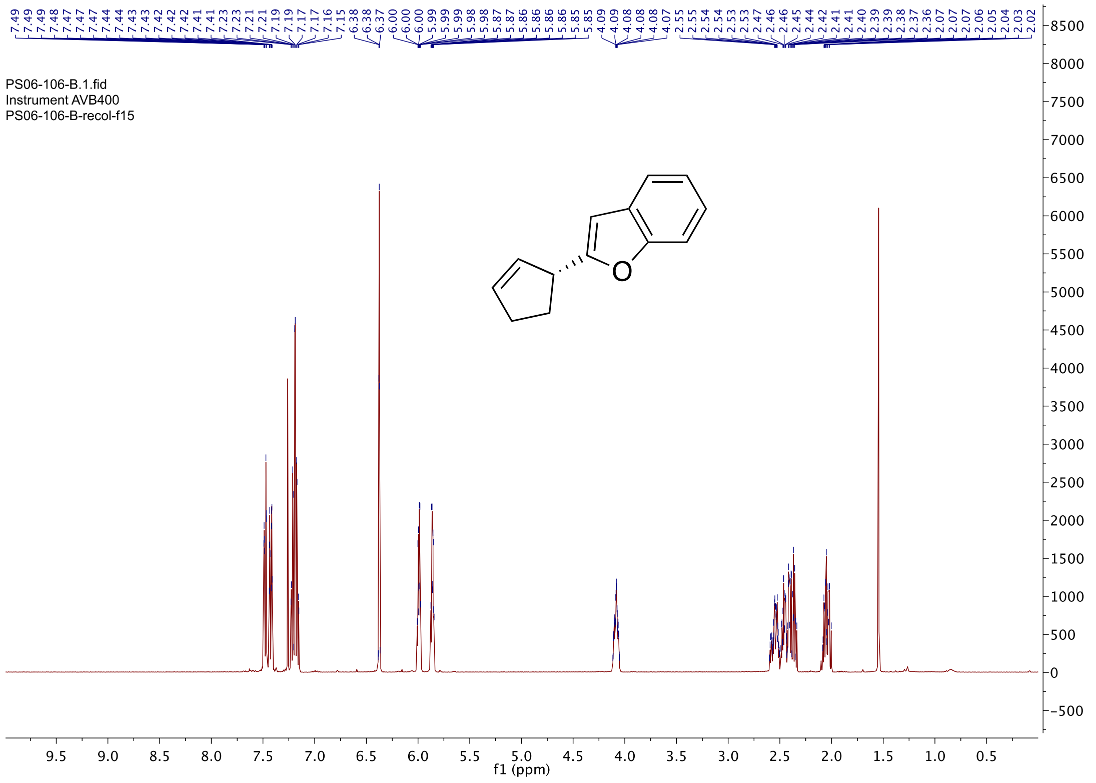

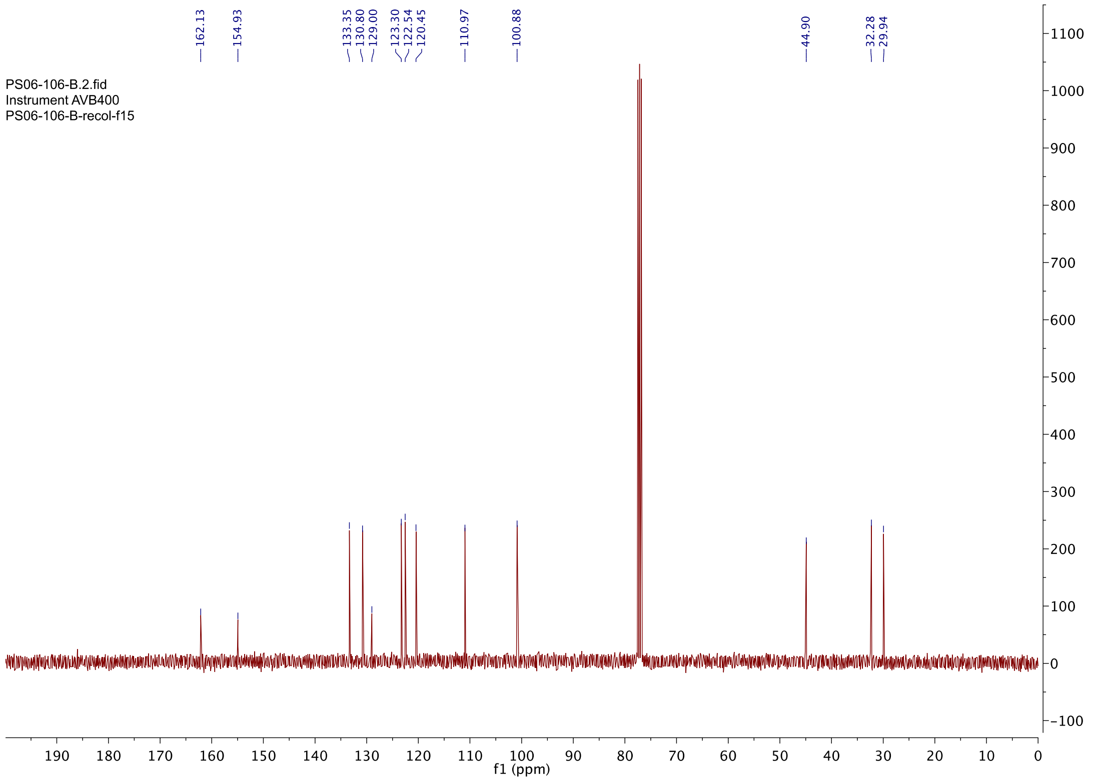

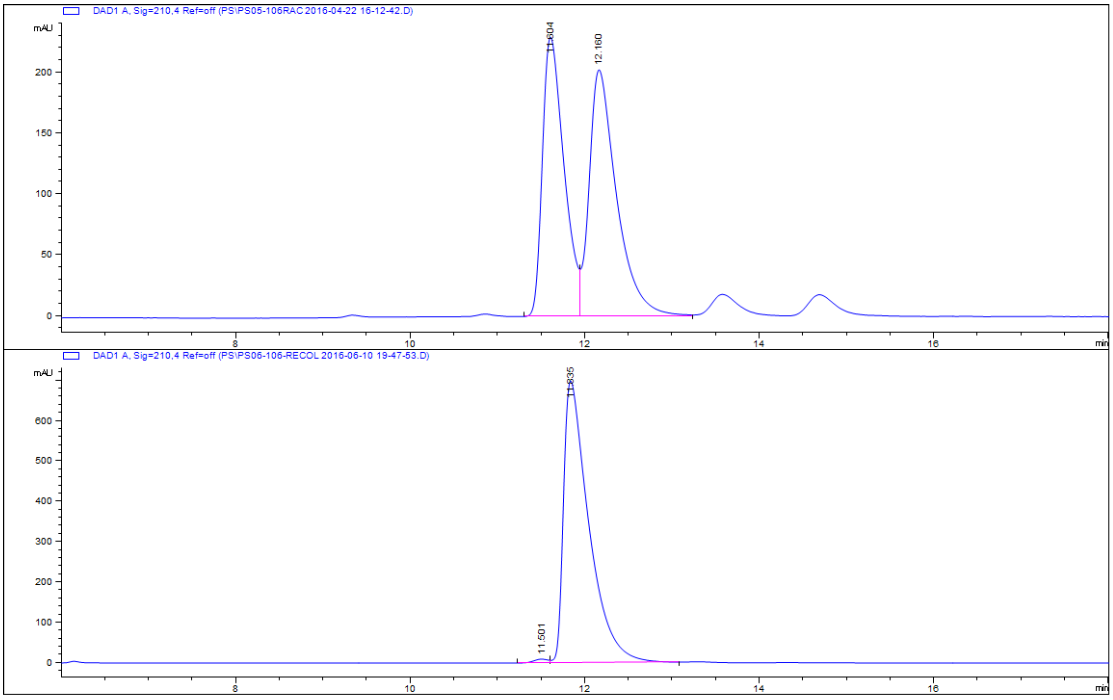

Supplementary figure 38: ^{1}H, ^{13}C-NMR spectra, HPLC traces of compound **40**

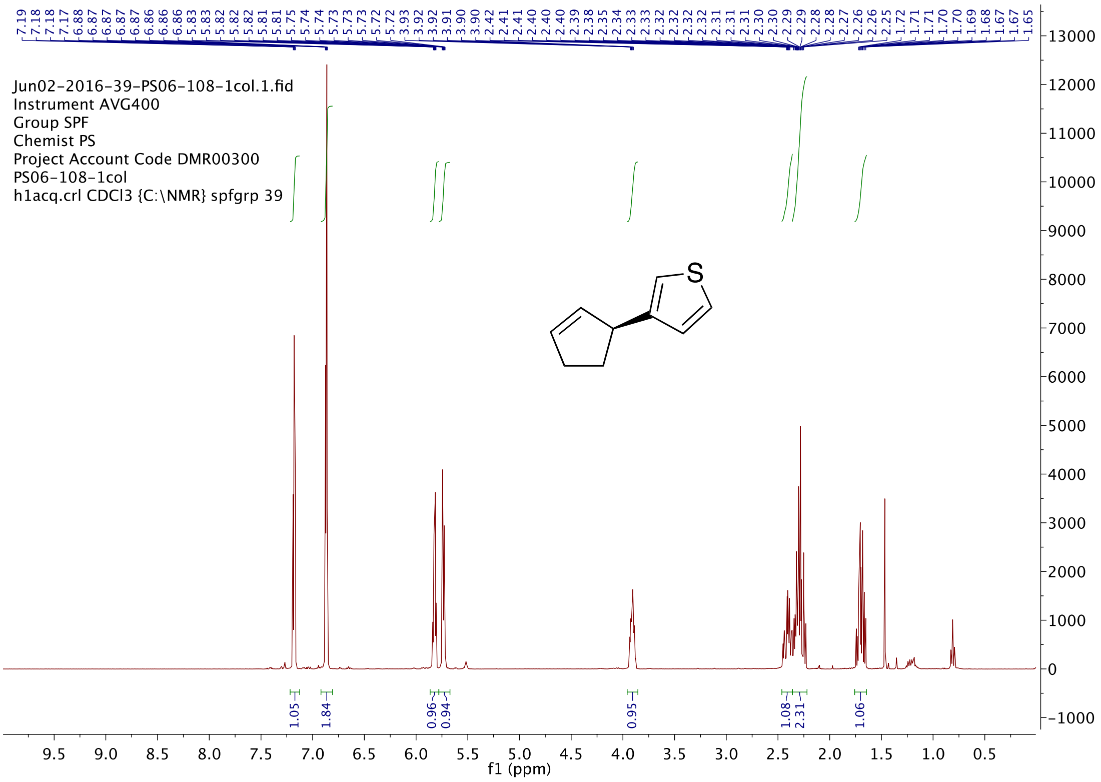

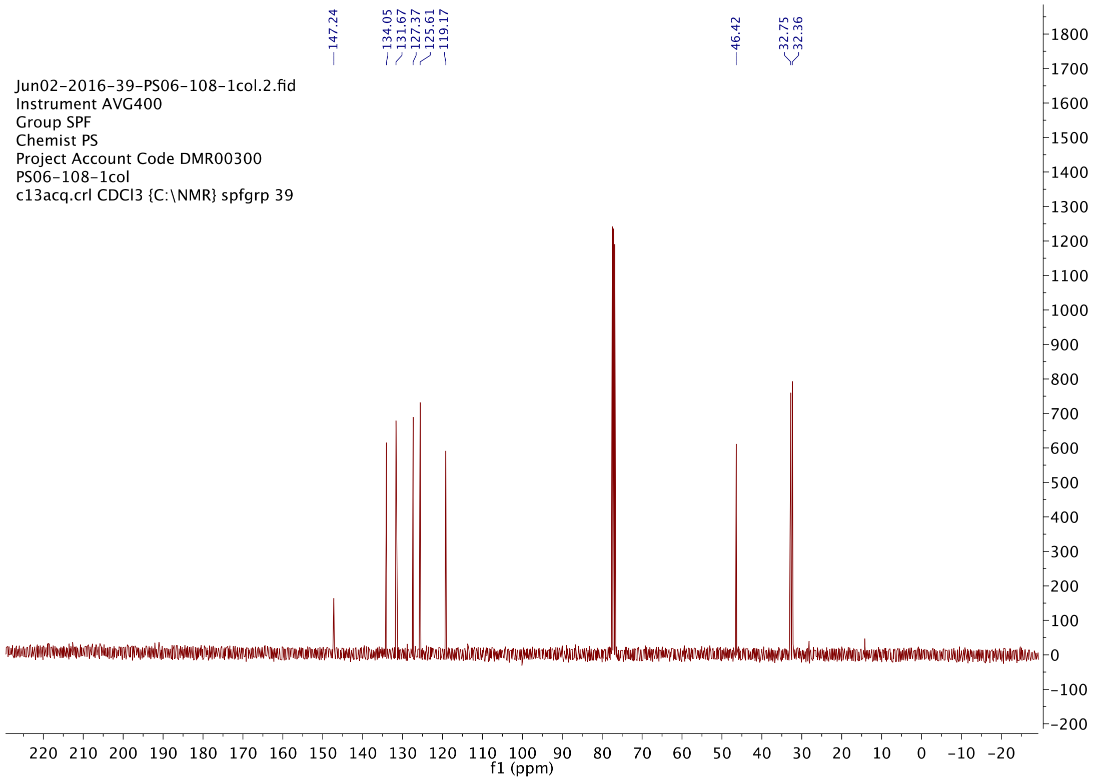
147.24
134.05
131.67
127.37
125.61
119.17
46.42
32.75
32.36
Jun02-2016-39-PS06-108-1col.2.fid
Instrument AVG400
Group SPF
Chemist PS
Project Account Code DMR00300
PS06-108-1col
c13acq.crl CDCl3 {C:\NMR} spfgrp 39
f1 (ppm)

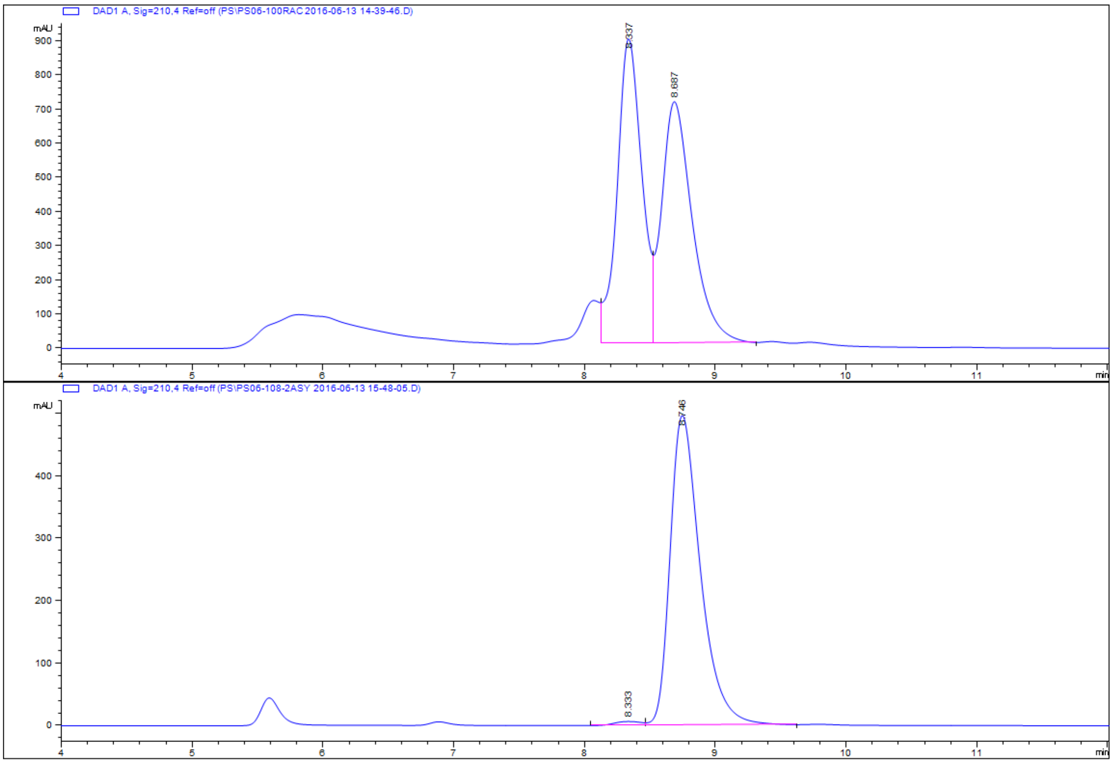
DAD1 A, Sig=210,4 Ref=off (PS\PS06-100RAC 2016-06-13 14-39-46.D)
mAU
8.337
8.687
min
DAD1 A, Sig=210,4 Ref=off (PS\PS06-108-2ASY 2016-06-13 15-48-05.D)
mAU
8.333
8.746
min

Supplementary figure 39: ^{1}H, ^{13}C-NMR spectra, HPLC traces of compound **41**

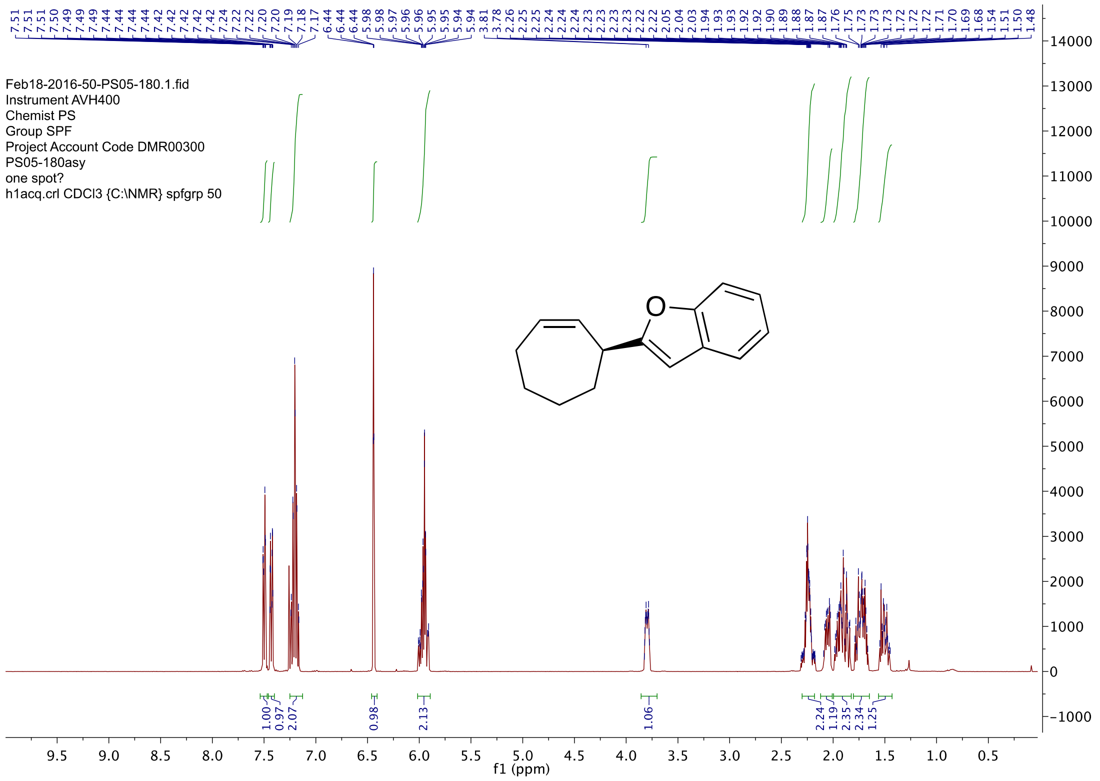

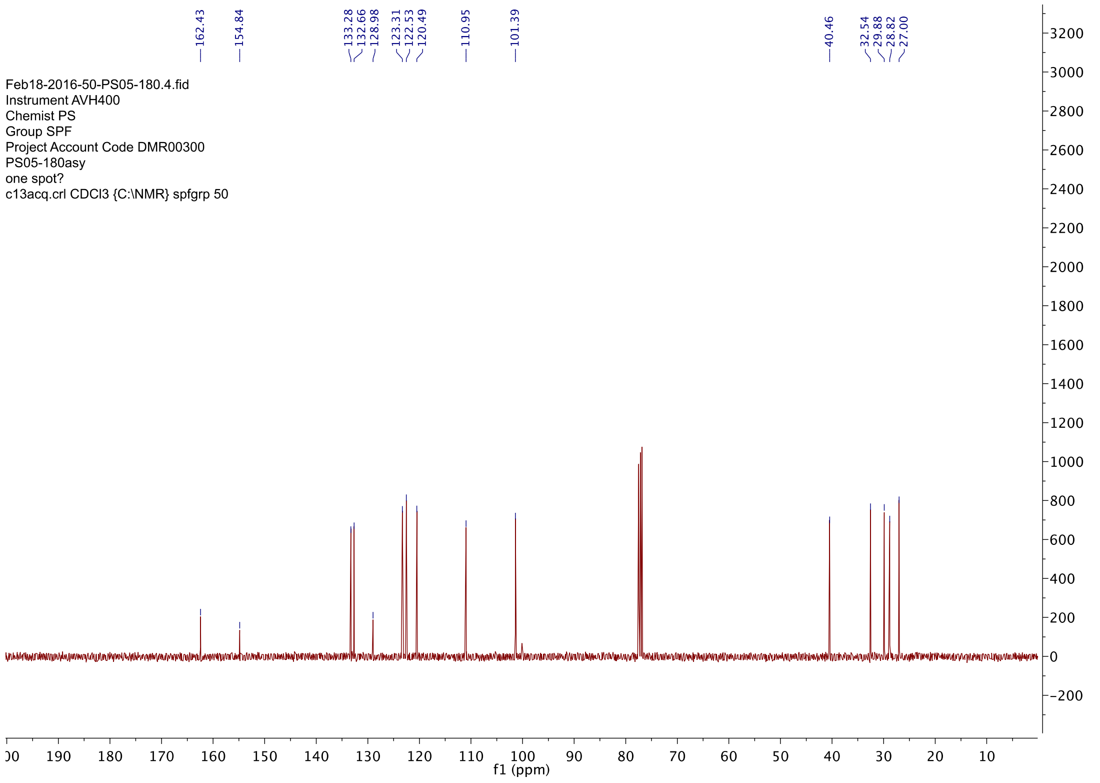

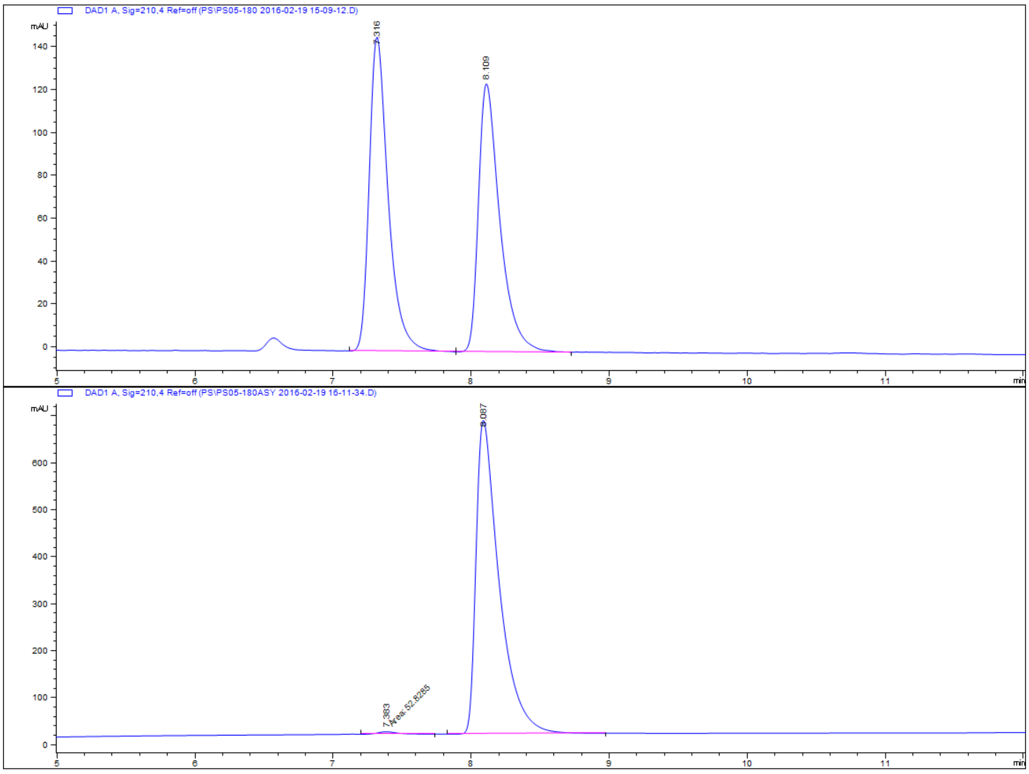

Supplementary figure 40: ^{1}H, ^{13}C-NMR spectra, HPLC traces of compound **42**

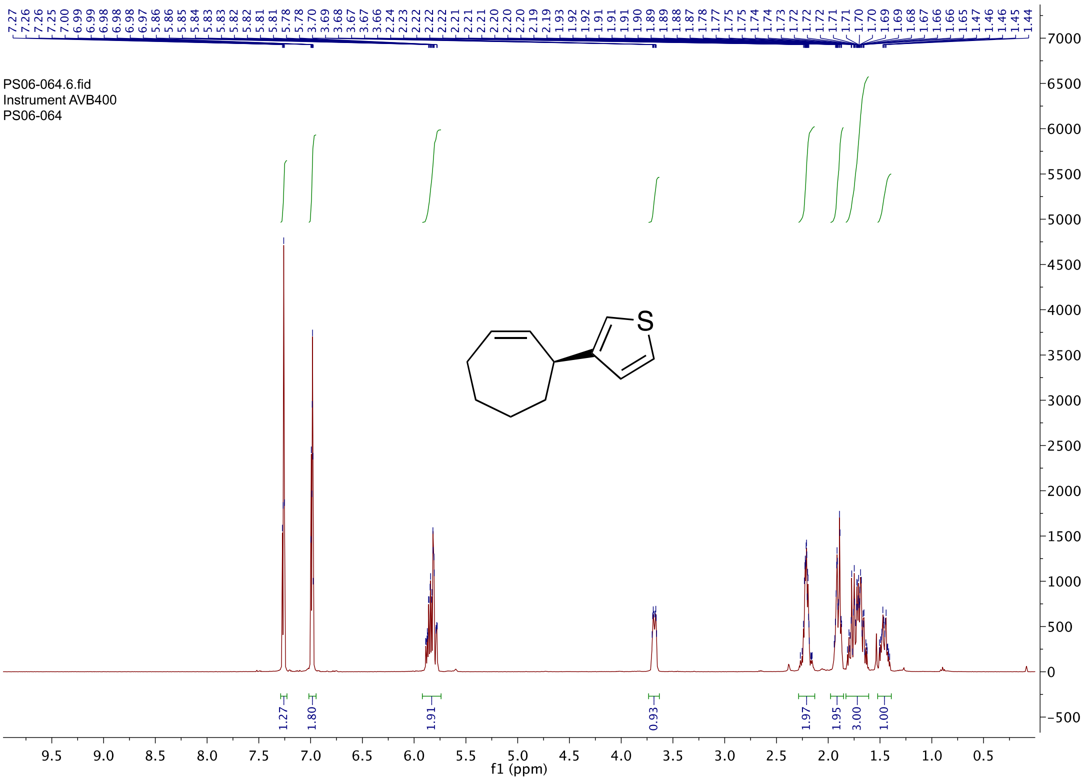

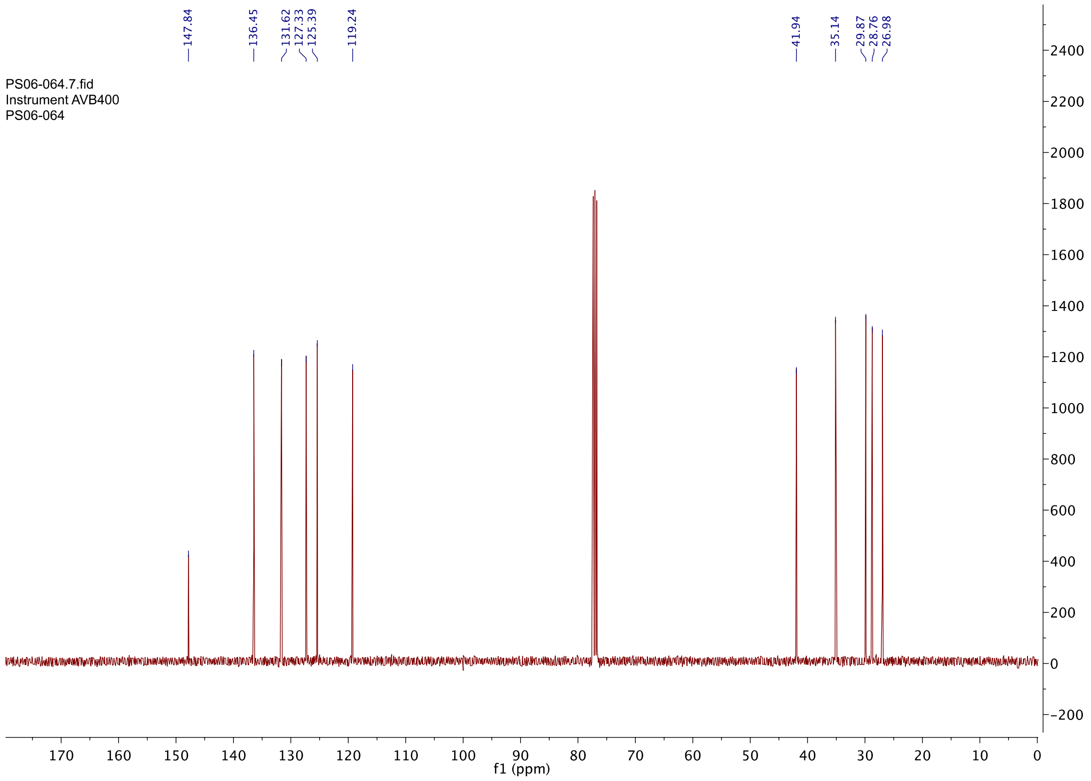
147.84
136.45
131.62
127.33
125.39
119.24
41.94
35.14
29.87
28.76
26.98
PS06-064.7.fid
Instrument AVB400
PS06-064
f1 (ppm)

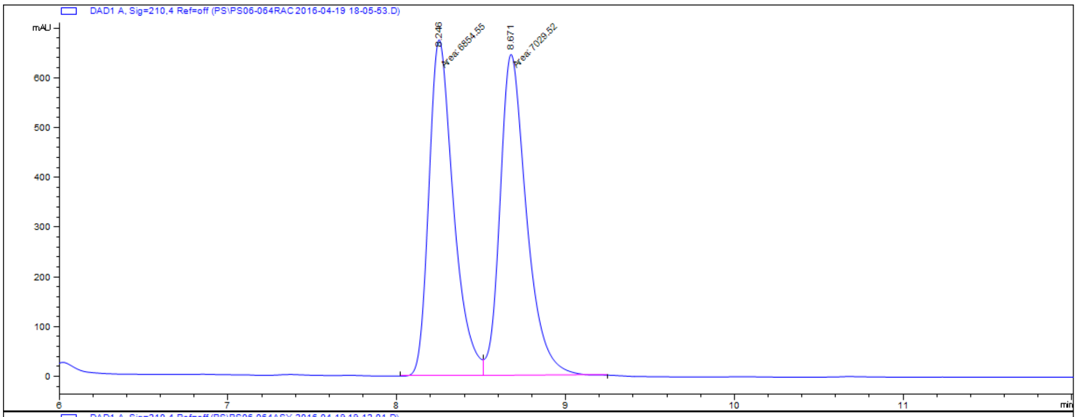
DAD1 A, Sig=210,4 Ref=off (PS\PS06-064RAC 2016-04-19 18-05-53.D)
mAU
8.248
Area: 6854.55
8.671
Area: 7029.52
min

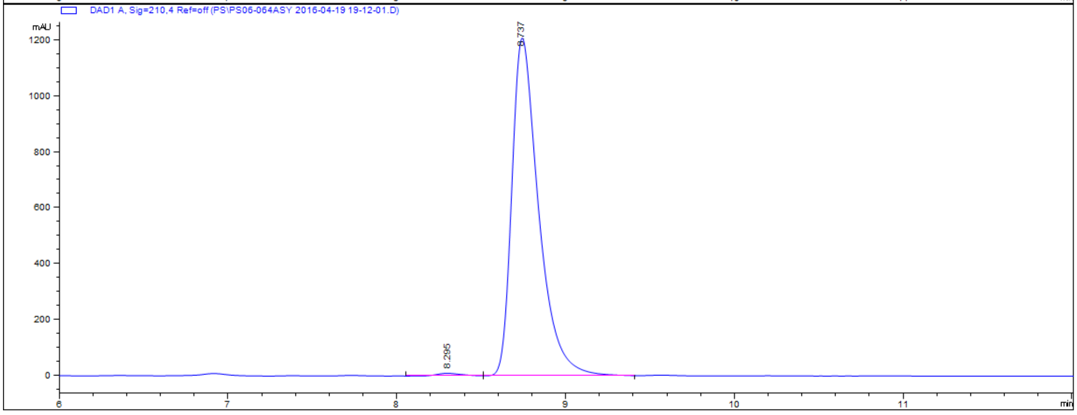
DAD1 A, Sig=210,4 Ref=off (PS\PS06-064ASY 2016-04-19 19-12-01.D)
mAU
8.737
8.295
min

Supplementary figure 41: ^{1}H, ^{13}C-NMR spectra, HPLC traces of compound **43**

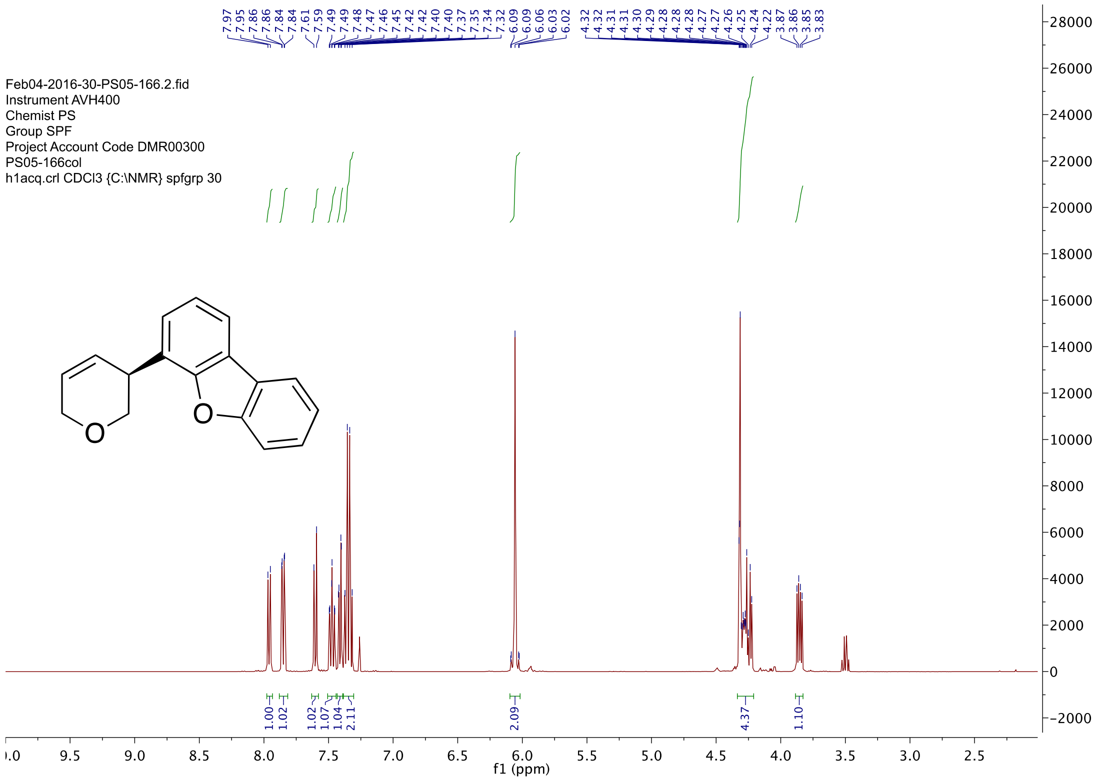

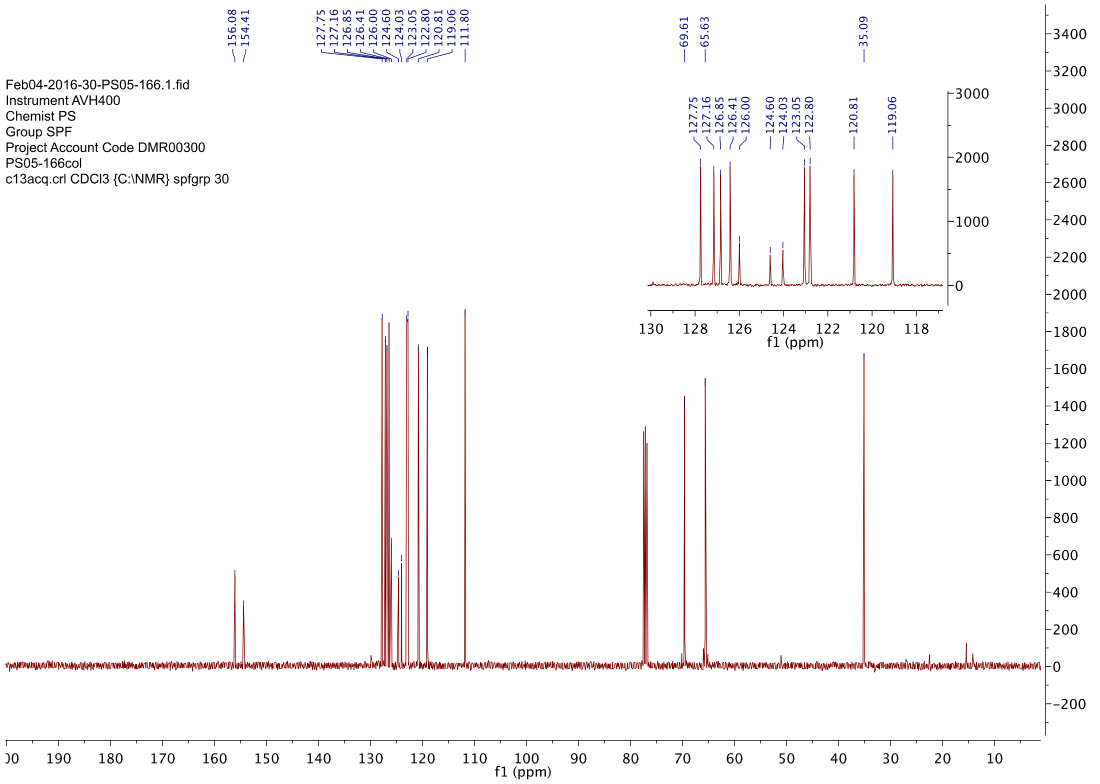

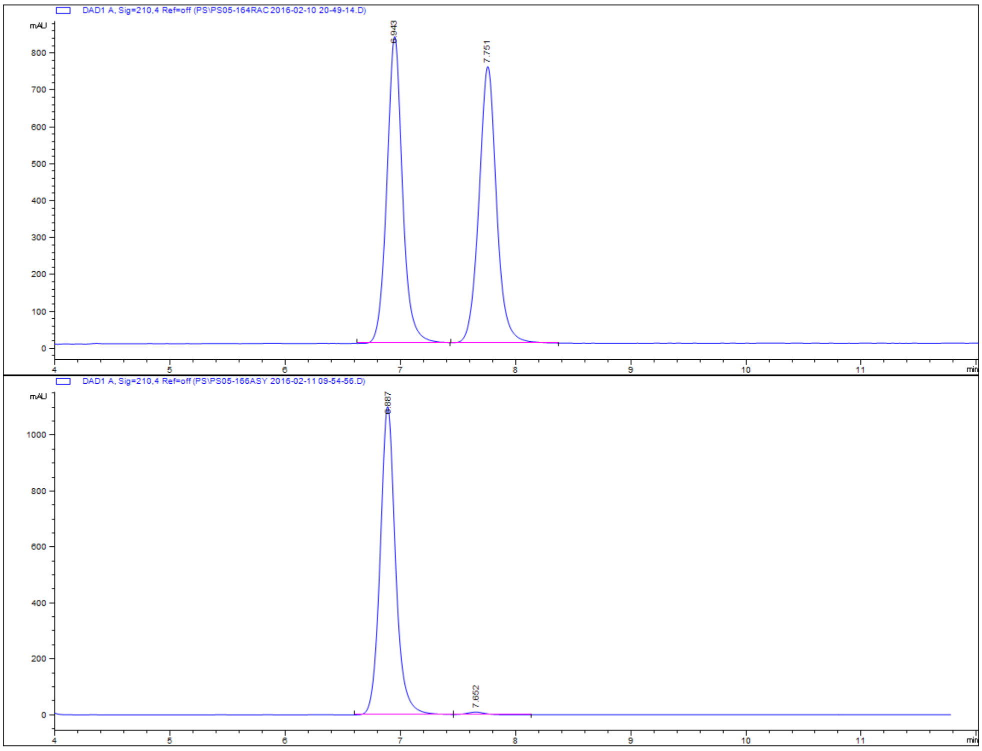

Supplementary figure 42: ^{1}H, ^{13}C-NMR spectra, HPLC traces of compound **44**

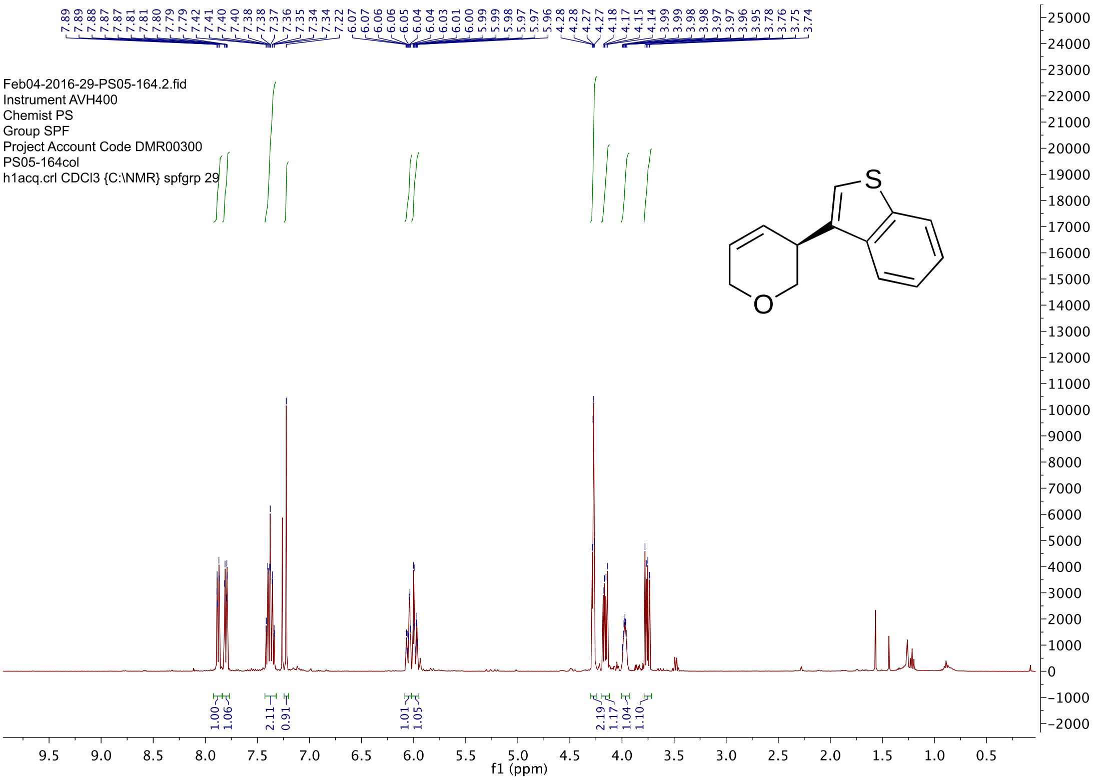

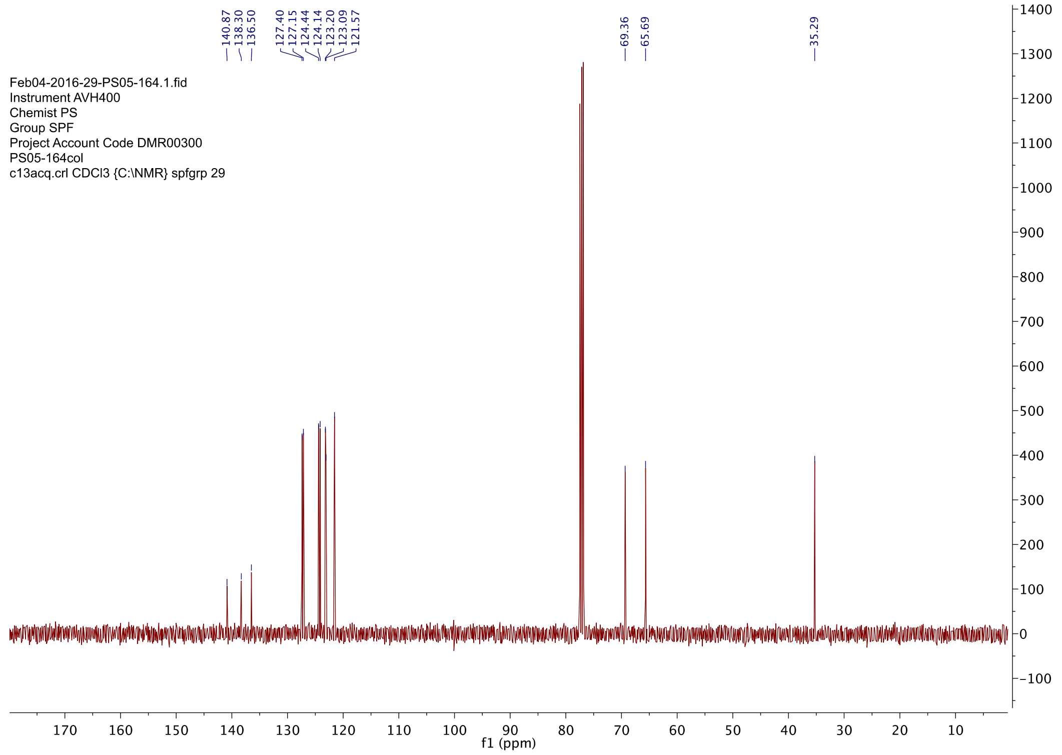

Feb04-2016-29-PS05-164.1.fid
Instrument AVH400
Chemist PS
Group SPF
Project Account Code DMR00300
PS05-164col
c13acq.crl CDCl3 {C:\NMR} spfgrp 29
140.87
138.30
136.50
127.40
127.15
124.44
124.14
123.20
123.09
121.57
69.36
65.69
35.29
f1 (ppm)

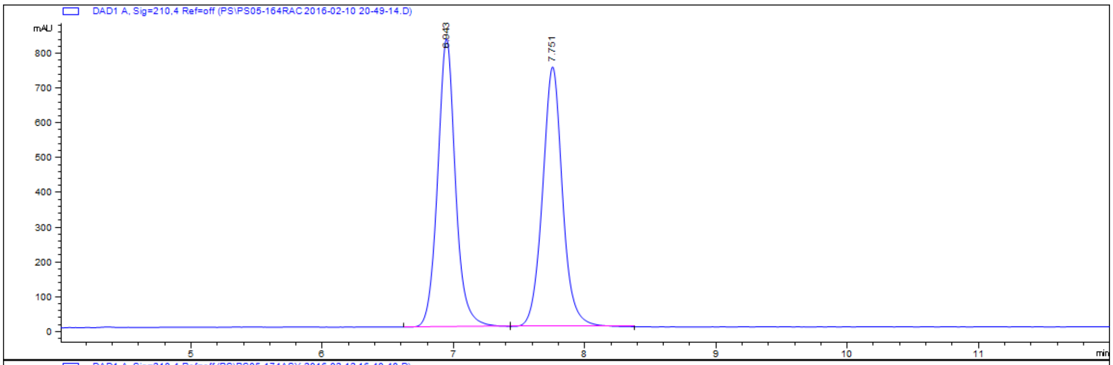

DAD1 A, Sig=210,4 Ref=off (PS\PS05-164RAC 2016-02-10 20-49-14.D)
mAU
6.943
7.751
min

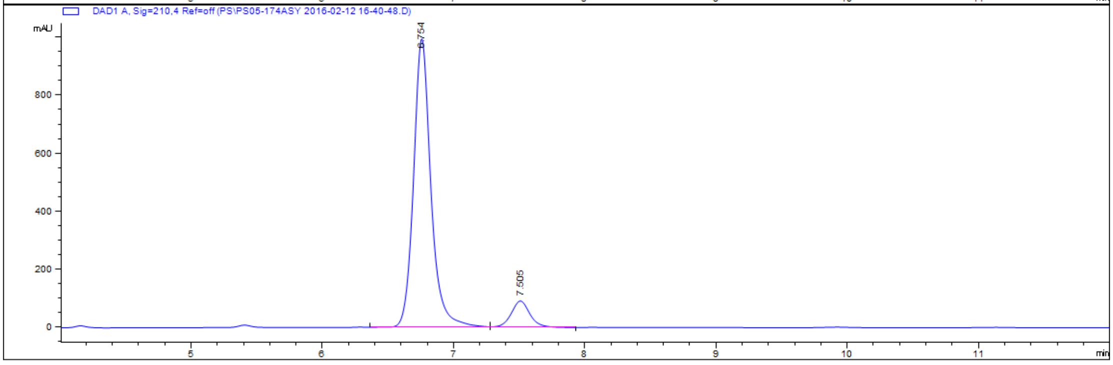

DAD1 A, Sig=210,4 Ref=off (PS\PS05-174ASY 2016-02-12 16-40-48.D)
mAU
6.754
7.505
min

Supplementary figure 43: ^{1}H, ^{13}C-NMR spectra, HPLC traces of compound **45a**

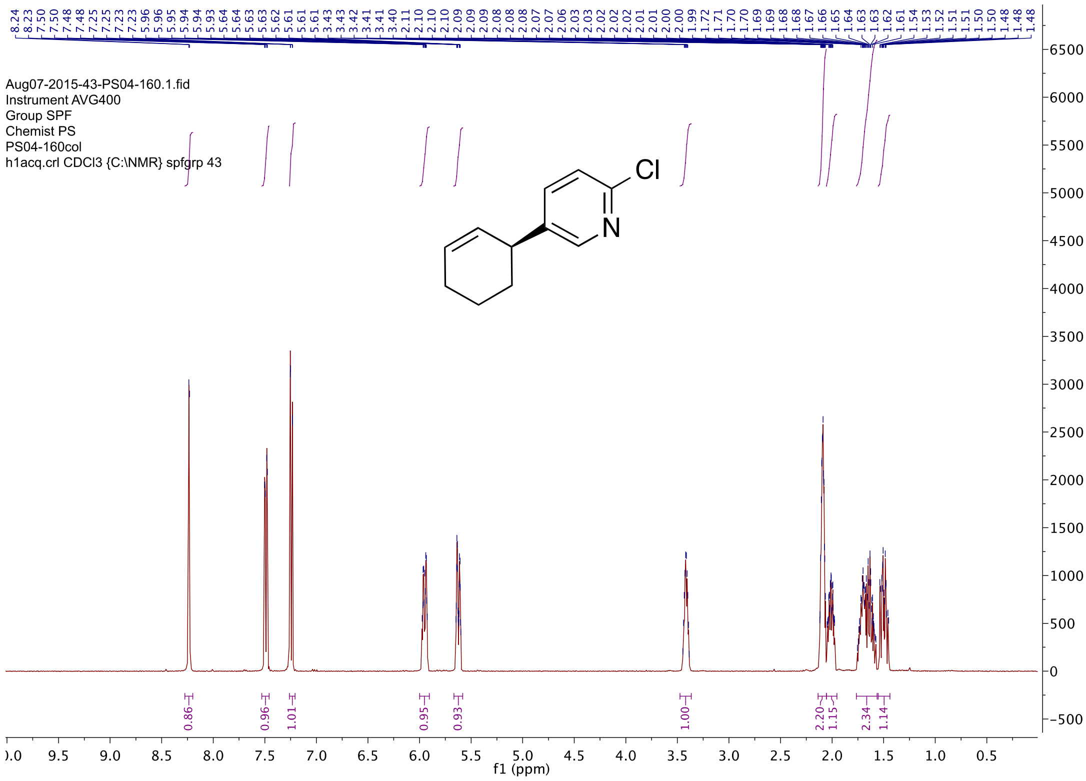

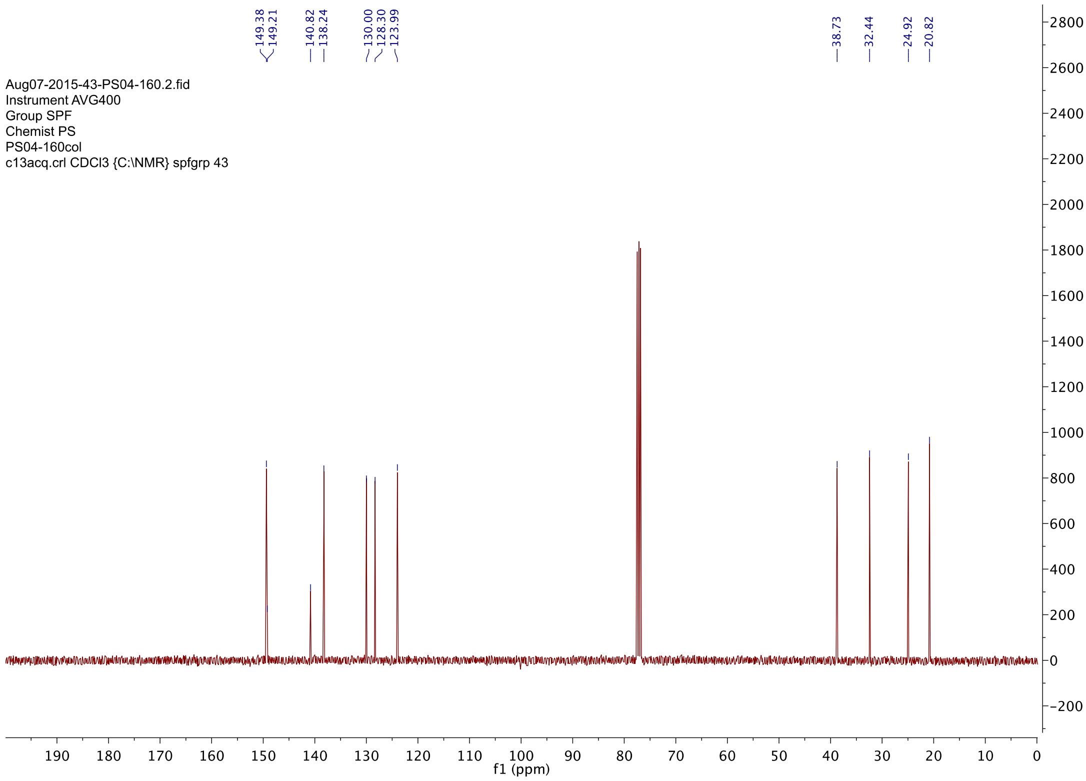

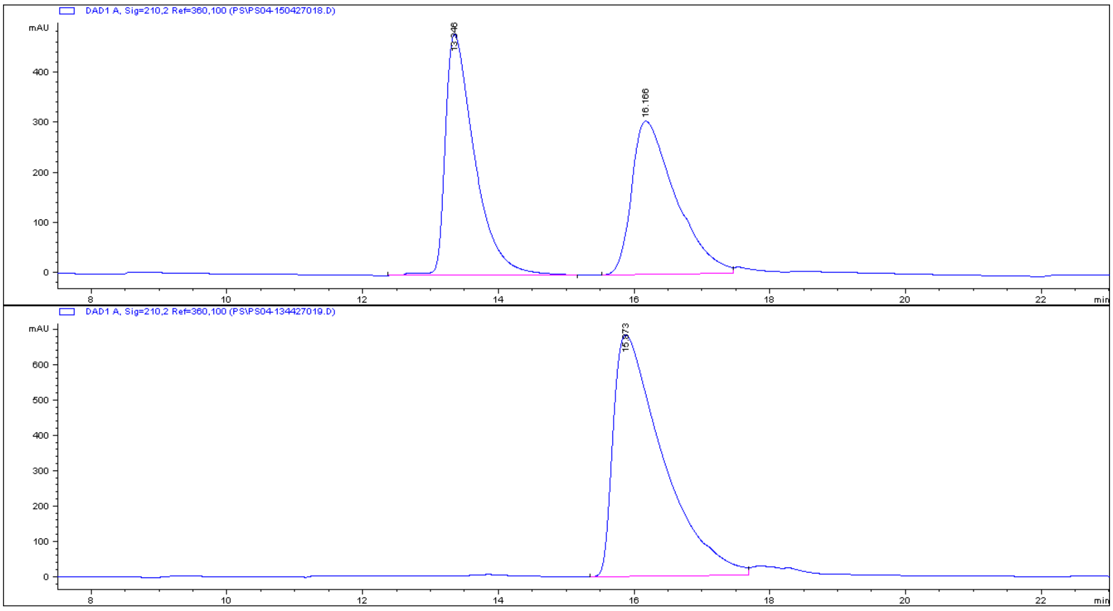

Supplementary figure 44: ^{1}H, ^{13}C-NMR spectra, HPLC traces of compound **45b**

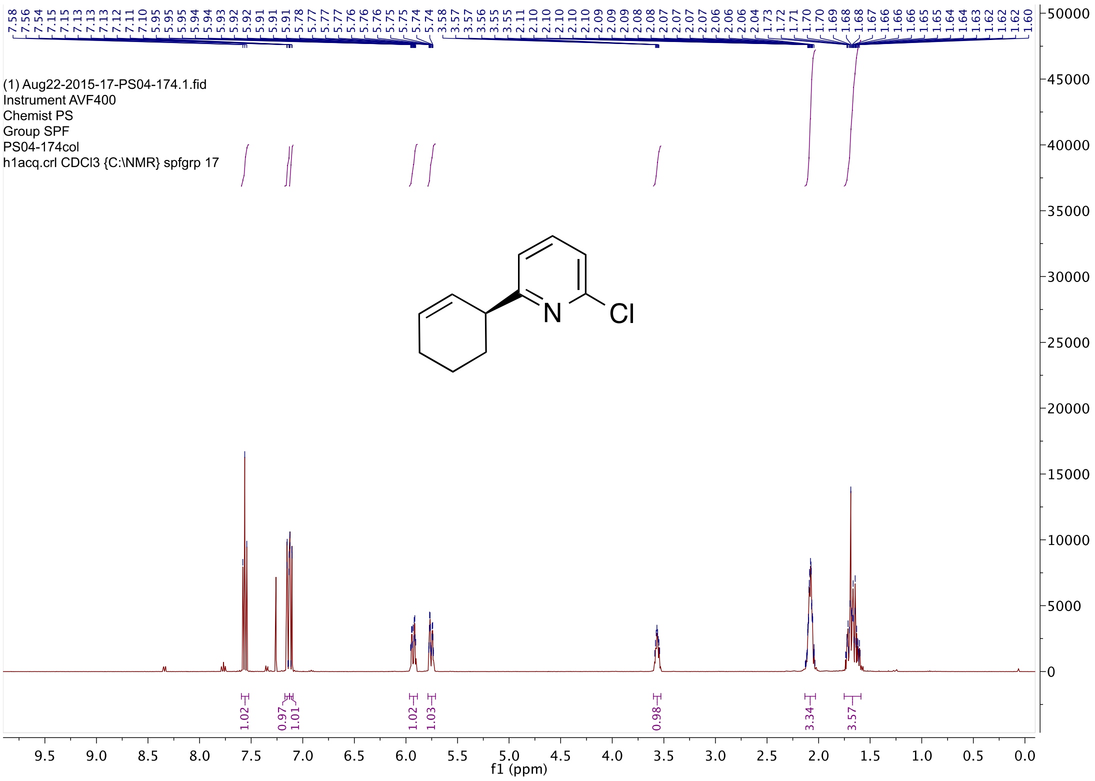

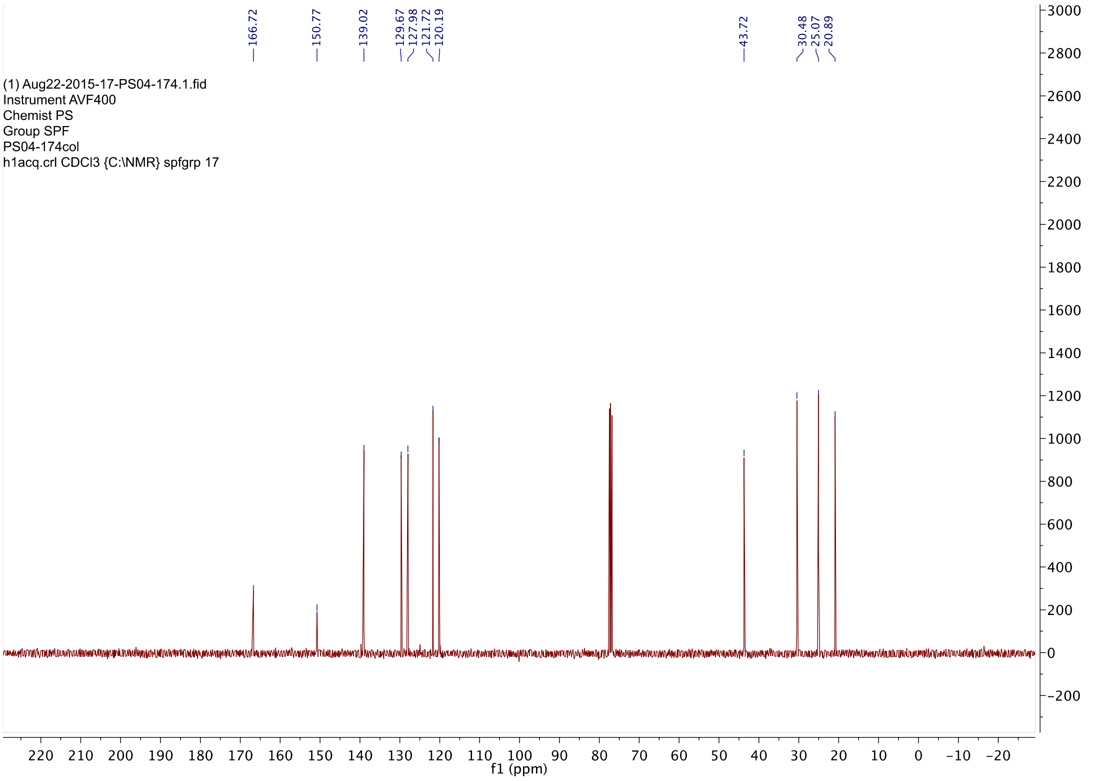
166.72
150.77
139.02
129.67
127.98
121.72
120.19
43.72
30.48
25.07
20.89
(1) Aug22-2015-17-PS04-174.1.fid
Instrument AVF400
Chemist PS
Group SPF
PS04-174col
h1acq.crl CDCl3 {C:\NMR} spfgrp 17
f1 (ppm)

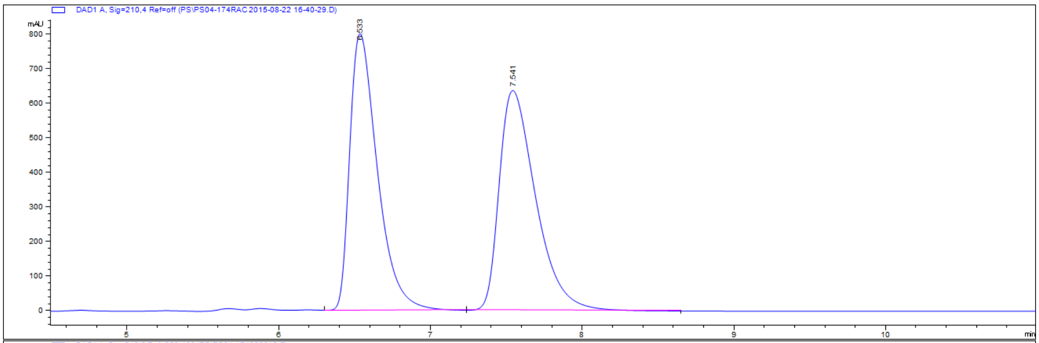
DAD1 A, Sig=210,4 Ref=off (PS\PS04-174RAC 2015-08-22 16-40-29.D)
mAU
6.533
7.541
min

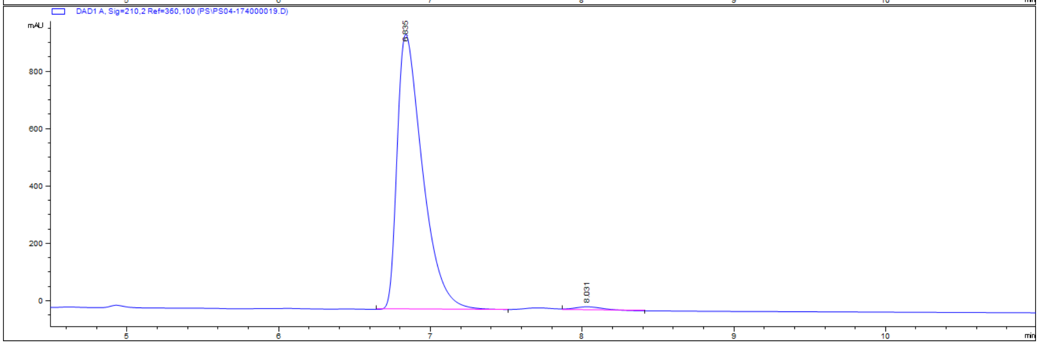
DAD1 A, Sig=210,2 Ref=360,100 (PS\PS04-174000019.D)
mAU
6.835
8.031
min

Supplementary figure 45: ^{1}H, ^{13}C-NMR spectra, HPLC traces of compound **45c**

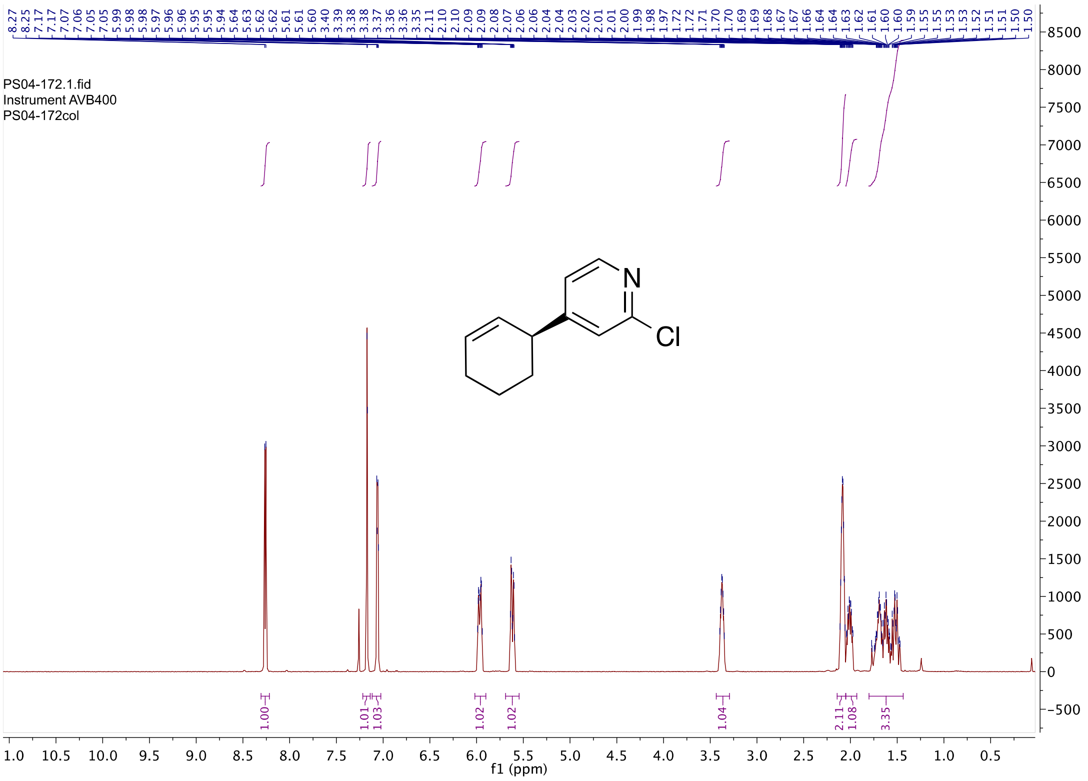

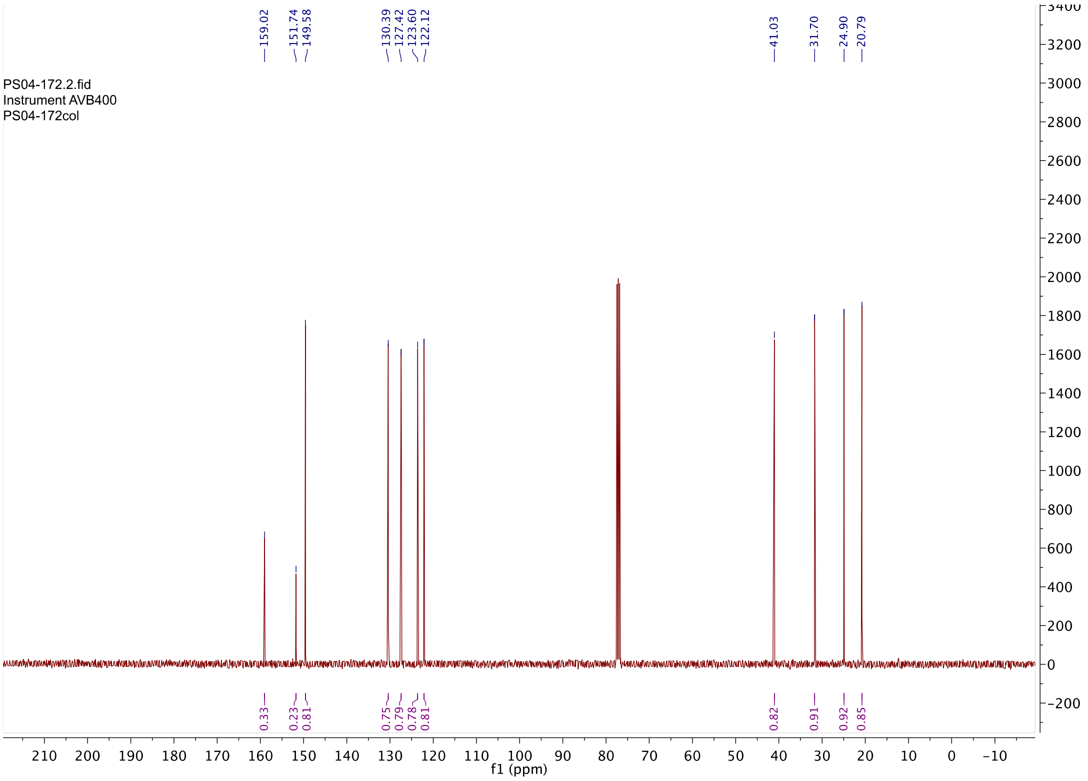

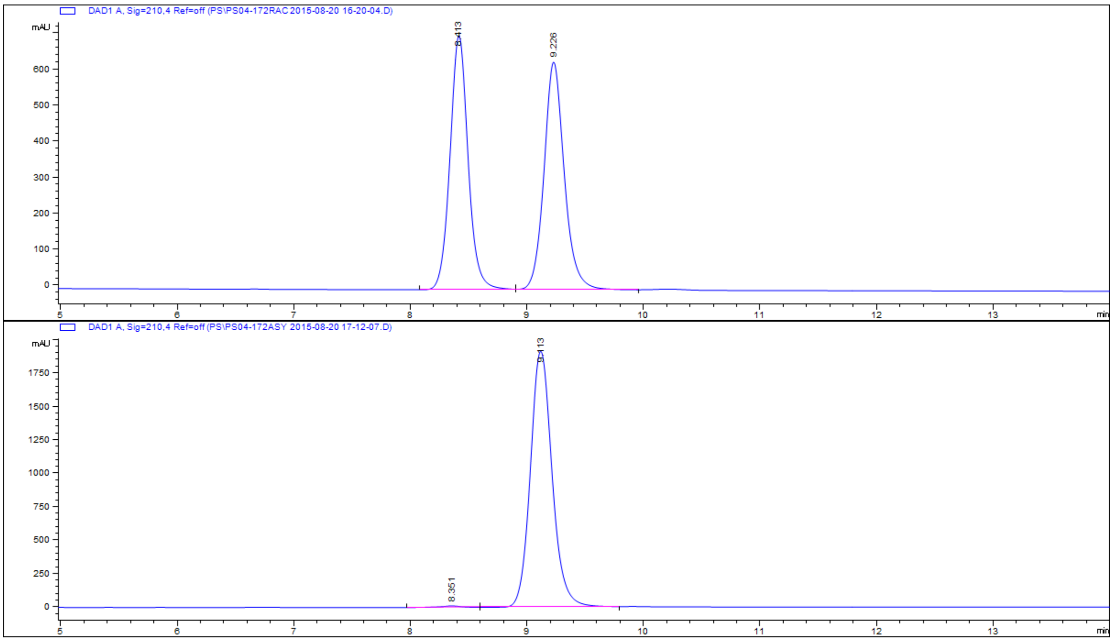

Supplementary figure 46: ^{1}H, ^{13}C-NMR spectra, HPLC traces of compound **45d**

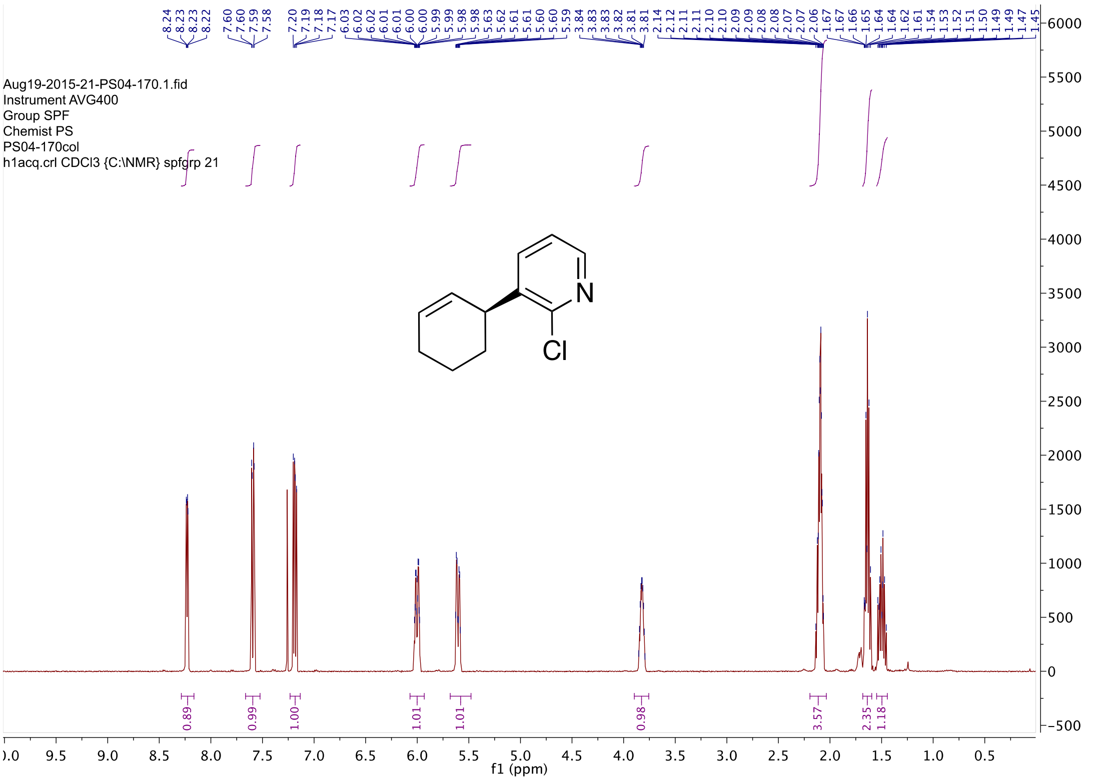

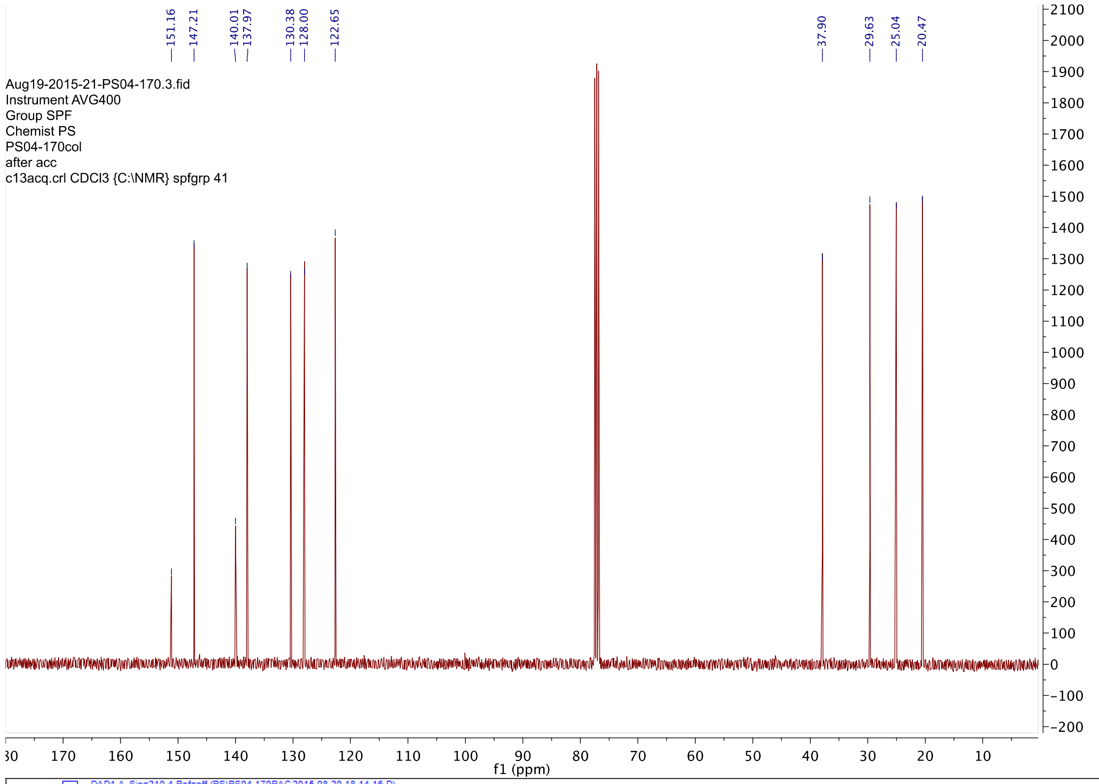

151.16
147.21
140.01
137.97
130.38
128.00
122.65
37.90
29.63
25.04
20.47
Aug19-2015-21-PS04-170.3.fid
Instrument AVG400
Group SPF
Chemist PS
PS04-170col
after acc
c13acq.crl CDCl3 {C:\NMR} spfgrp 41
f1 (ppm)

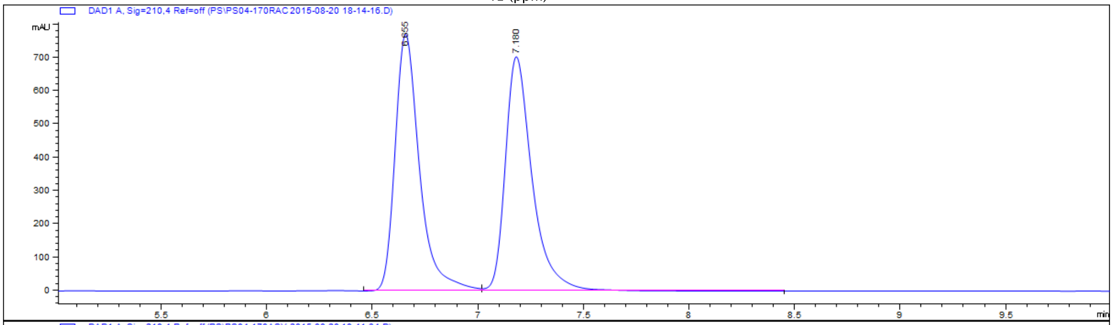

DAD1 A, Sig=210,4 Ref=off (PS\PS04-170RAC 2015-08-20 18-14-16.D)
mAU
6.655
7.180
min

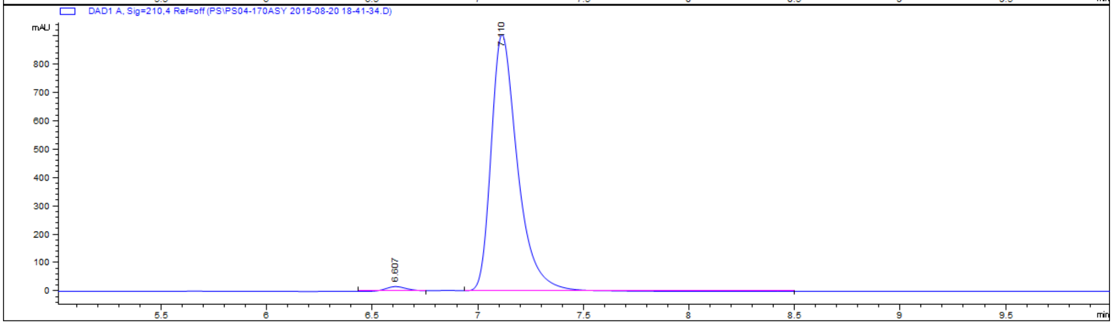

DAD1 A, Sig=210,4 Ref=off (PS\PS04-170ASY 2015-08-20 18-41-34.D)
mAU
6.607
7.110
min

Supplementary figure 47: ^{1}H, ^{13}C-NMR spectra, HPLC traces of compound **46**

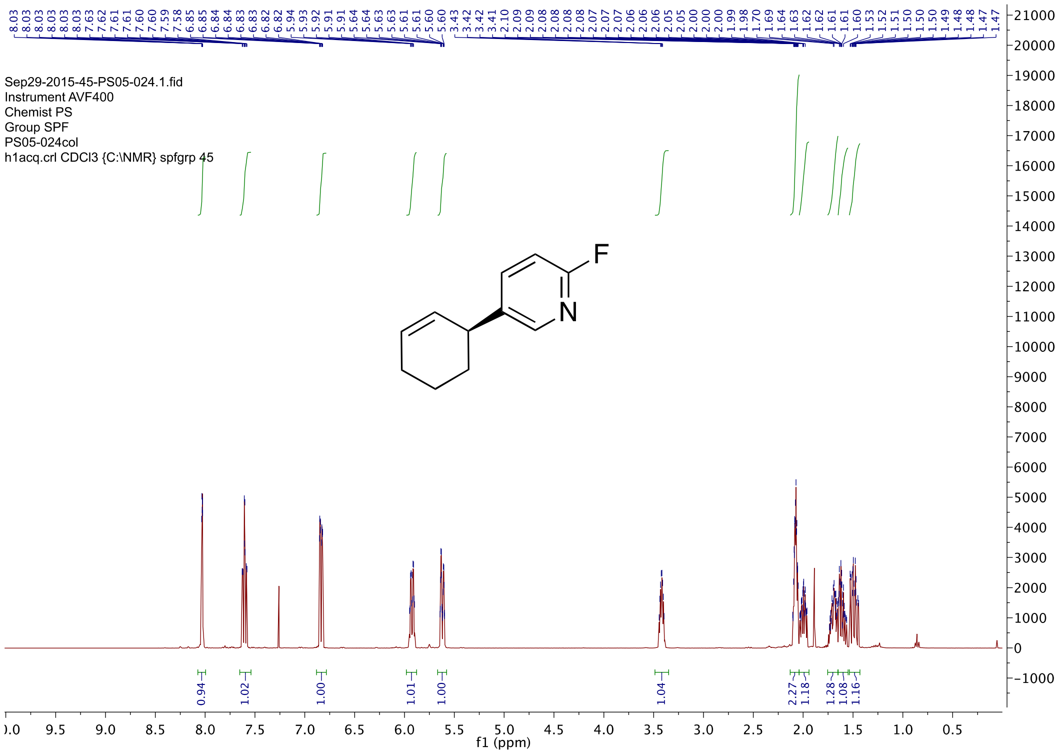

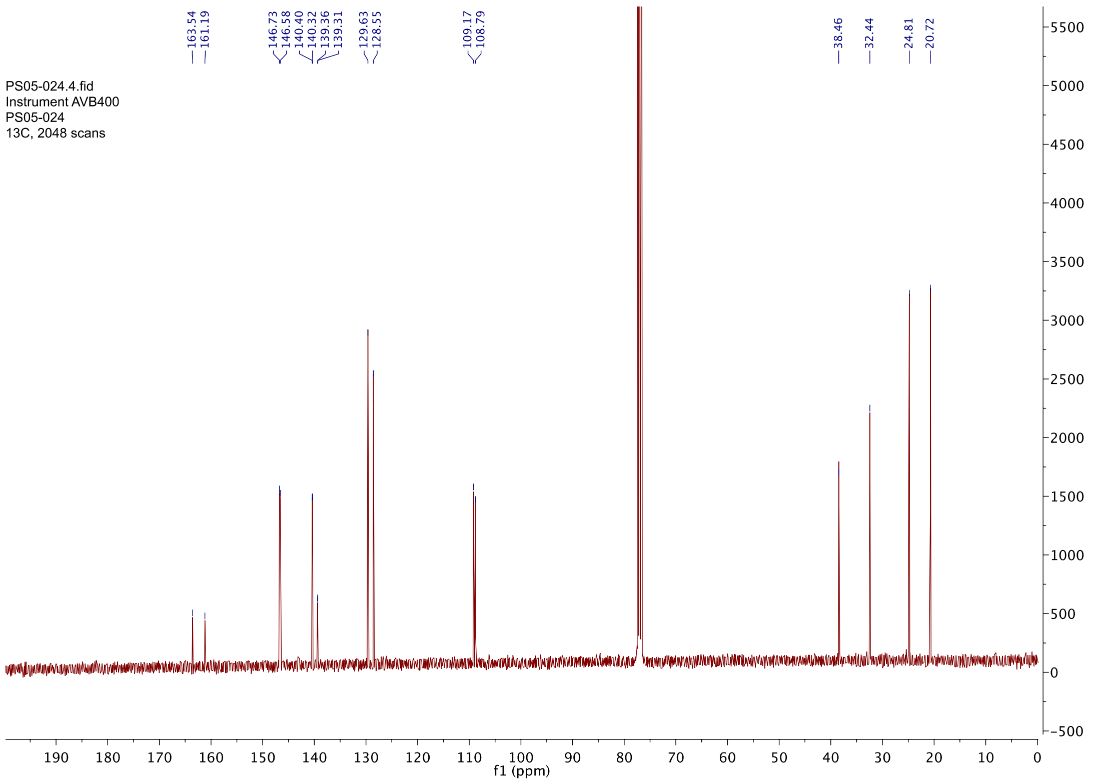
PS05-024.4.fid
Instrument AVB400
PS05-024
13C, 2048 scans
163.54
161.19
146.73
146.58
140.40
140.32
139.36
139.31
129.63
128.55
109.17
108.79
38.46
32.44
24.81
20.72
f1 (ppm)

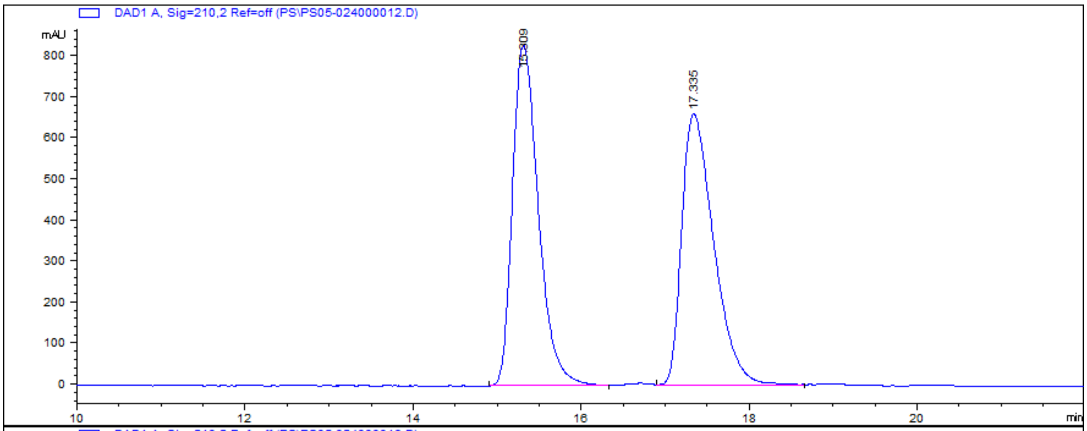
DAD1 A, Sig=210,2 Ref=off (PS\PS05-024000012.D)
mAU
15.309
17.335
min

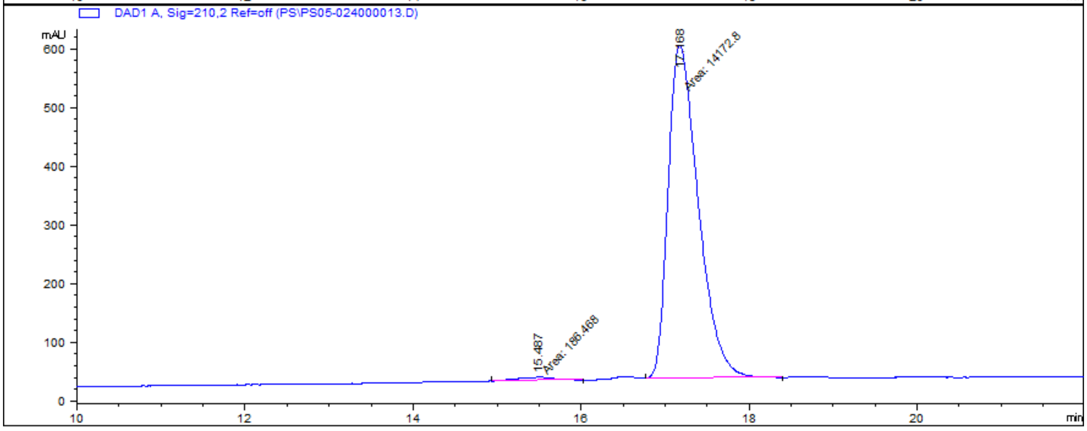
DAD1 A, Sig=210,2 Ref=off (PS\PS05-024000013.D)
mAU
15.487
Area: 186.468
17.168
Area: 14172.8
min

Supplementary figure 48: ^{1}H, ^{13}C-NMR spectra, HPLC traces of compound **47**

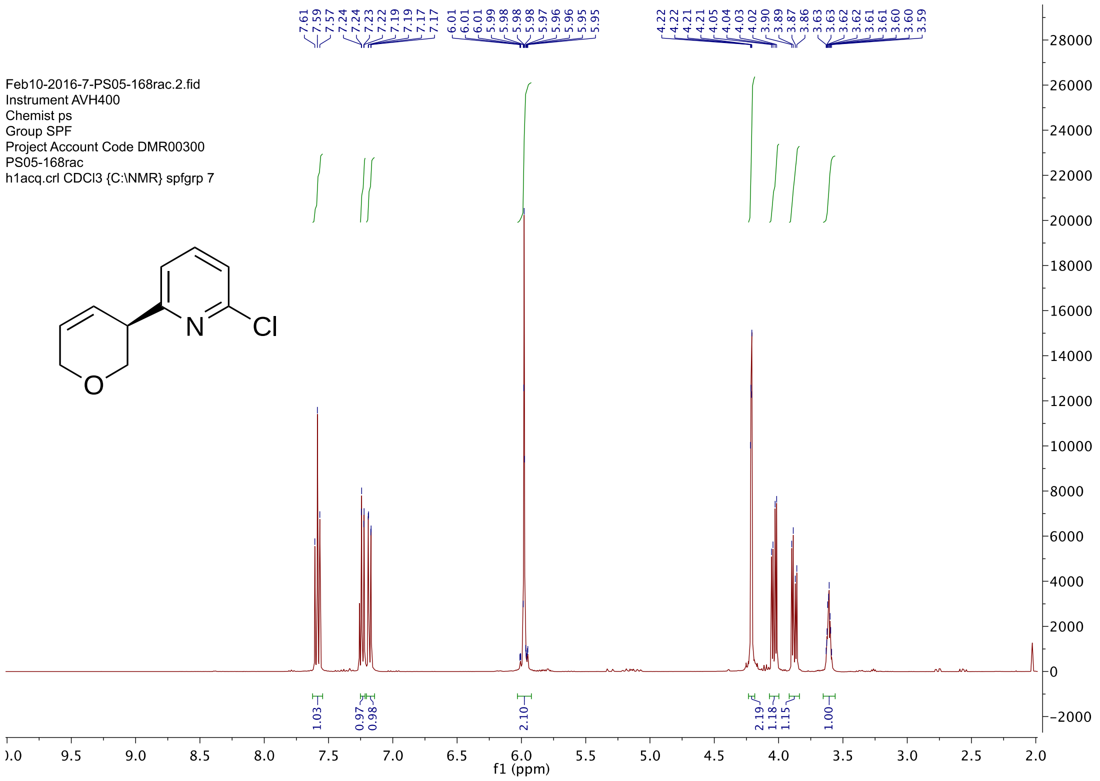

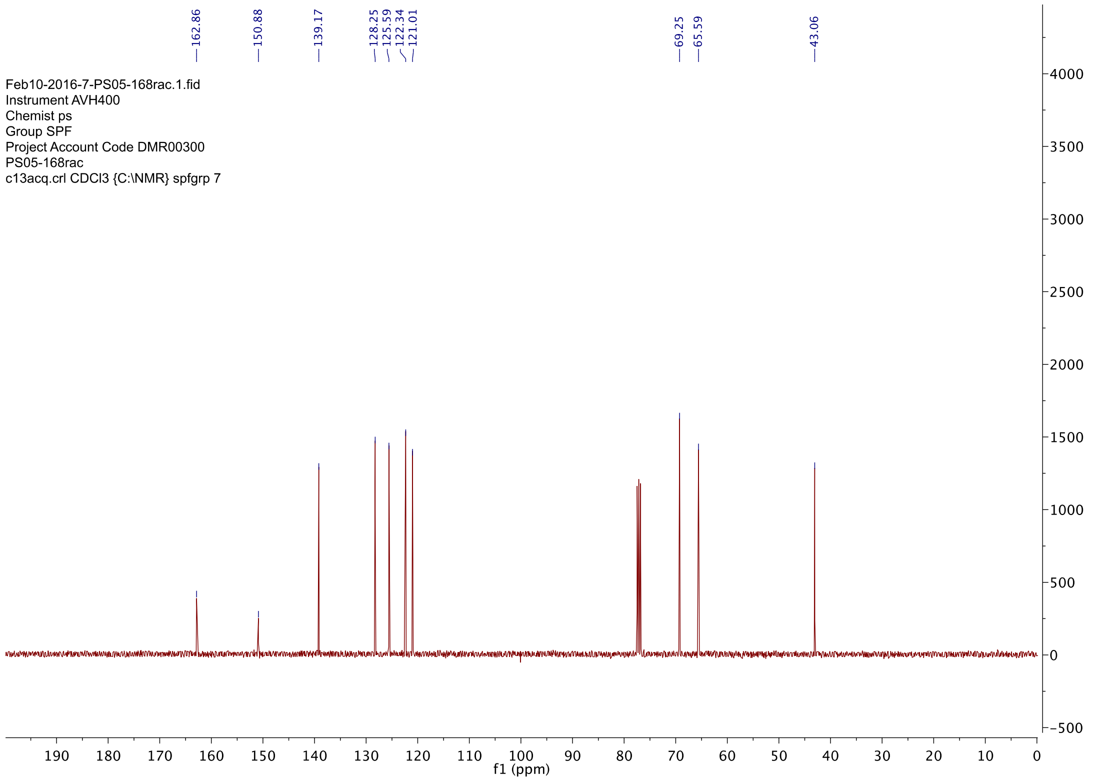

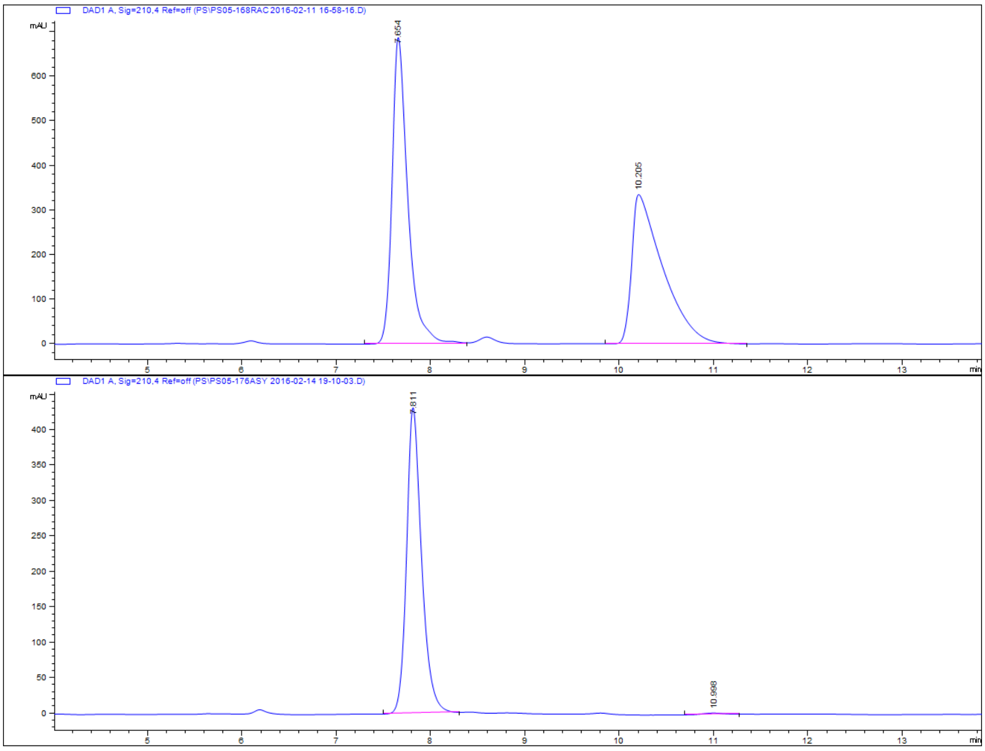

Supplementary figure 49: ^{1}H, ^{13}C-NMR spectra, HPLC traces of compound **48**

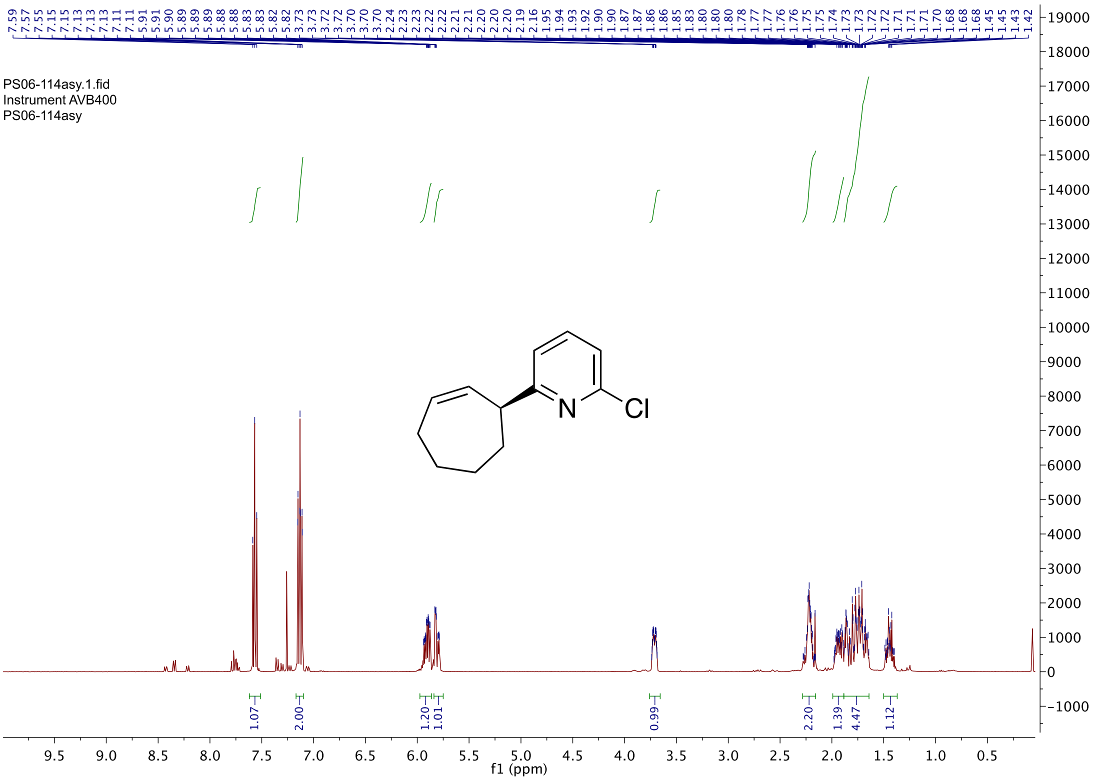

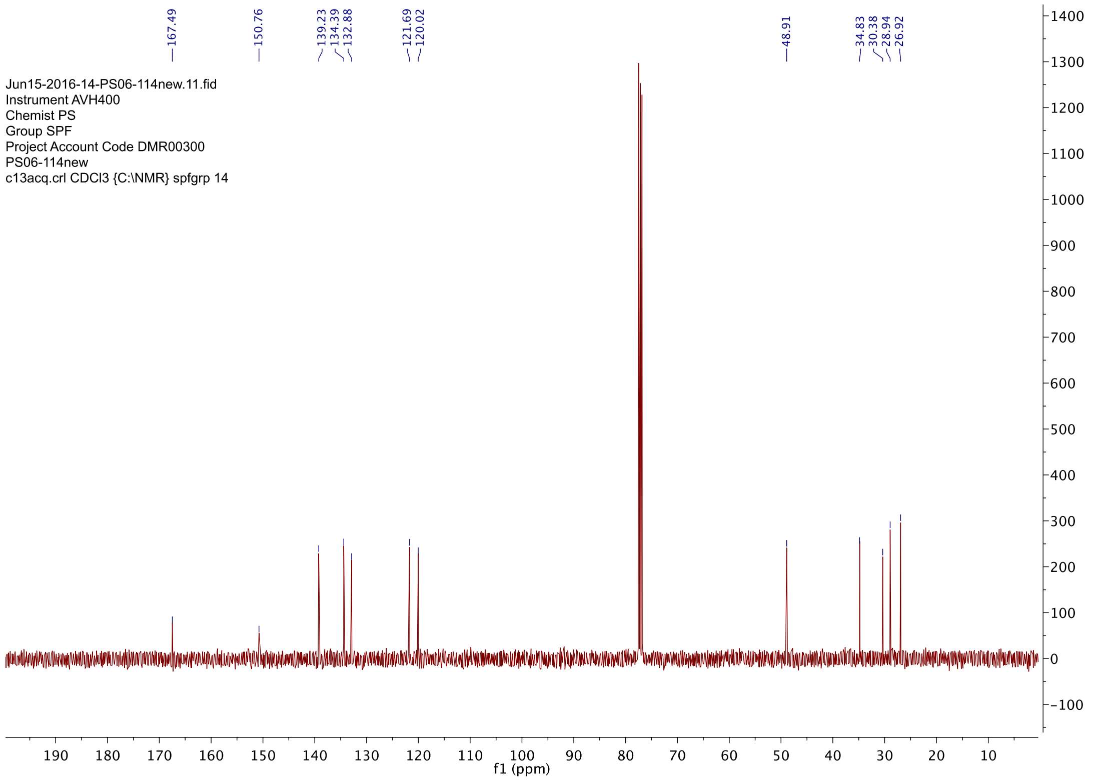
167.49
150.76
139.23
134.39
132.88
121.69
120.02
48.91
34.83
30.38
28.94
26.92
Jun15-2016-14-PS06-114new.11.fid
Instrument AVH400
Chemist PS
Group SPF
Project Account Code DMR00300
PS06-114new
c13acq.crl CDCl3 {C:\NMR} spfgrp 14
f1 (ppm)

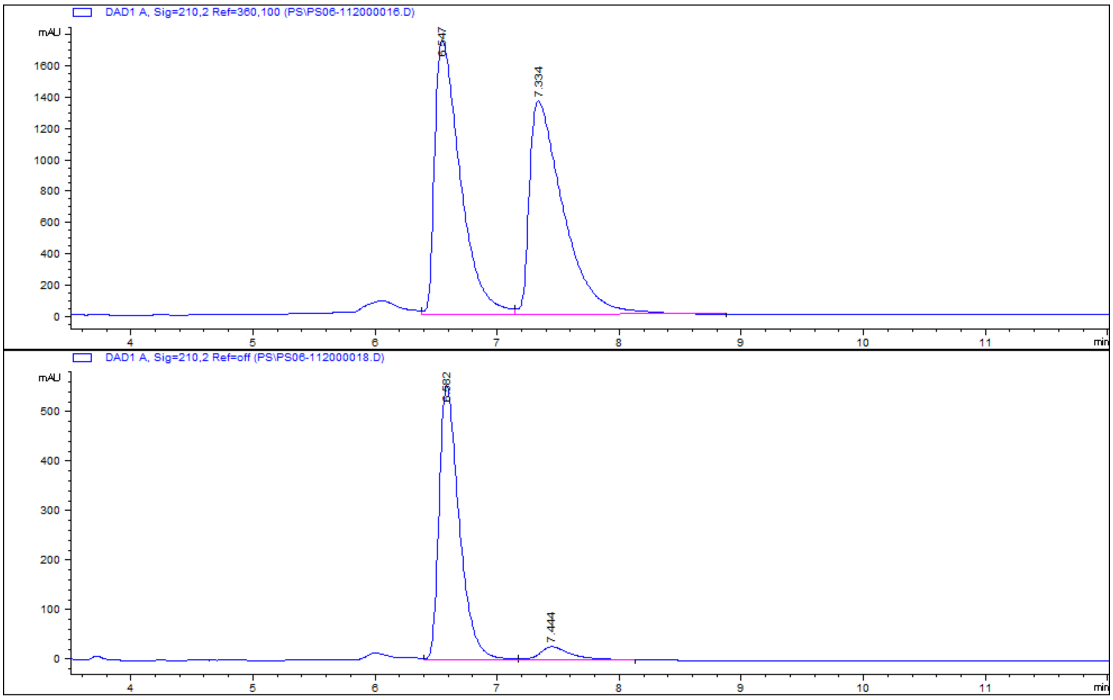
DAD1 A, Sig=210,2 Ref=360,100 (PS\PS06-112000016.D)
mAU
6.547
7.334
min
DAD1 A, Sig=210,2 Ref=off (PS\PS06-112000018.D)
mAU
6.582
7.444
min

Supplementary figure 50: ^{1}H, ^{13}C-NMR spectra, HPLC traces of compound **49**

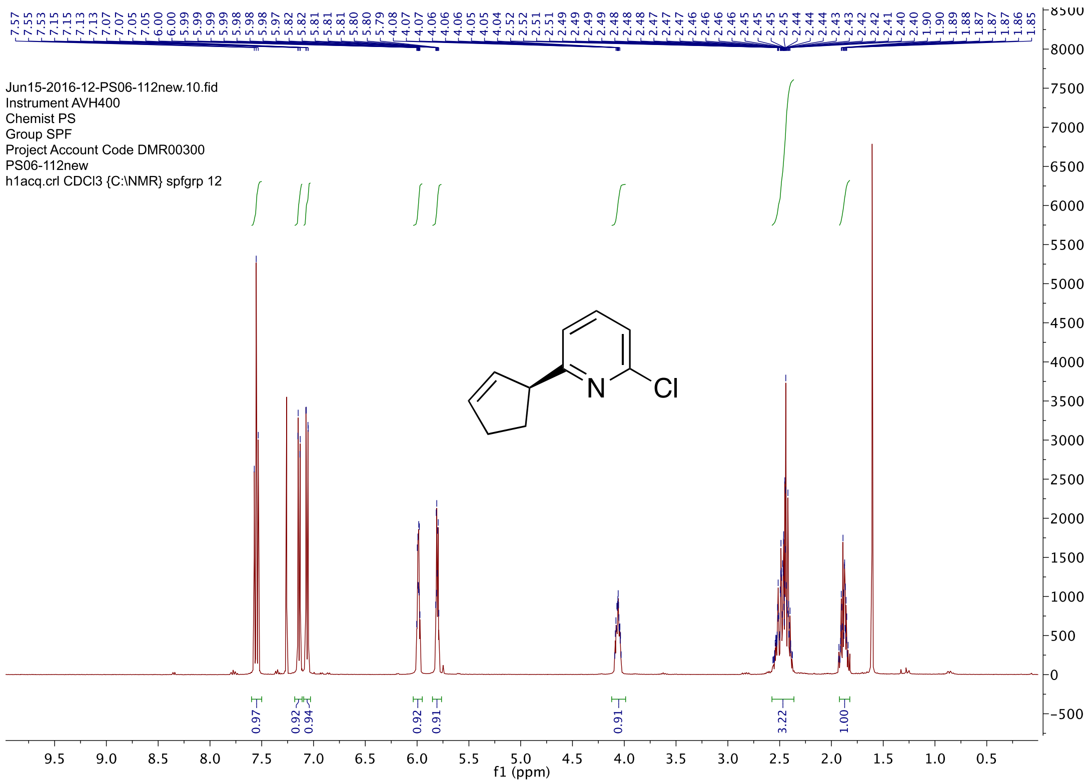

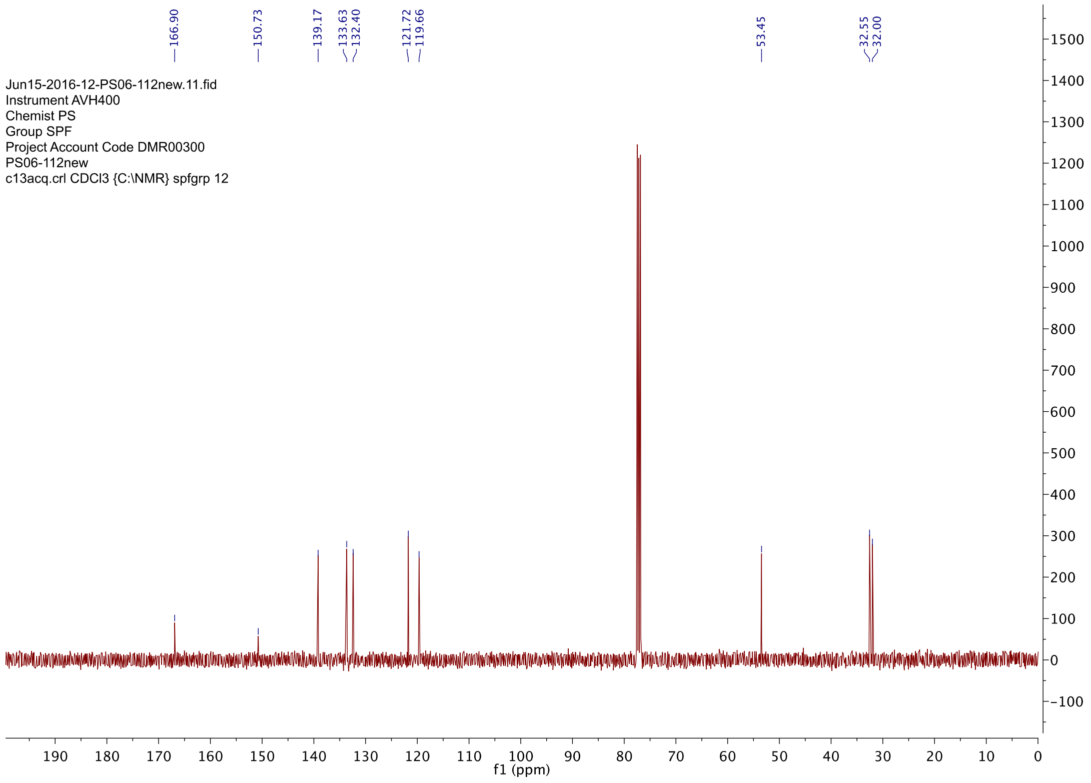

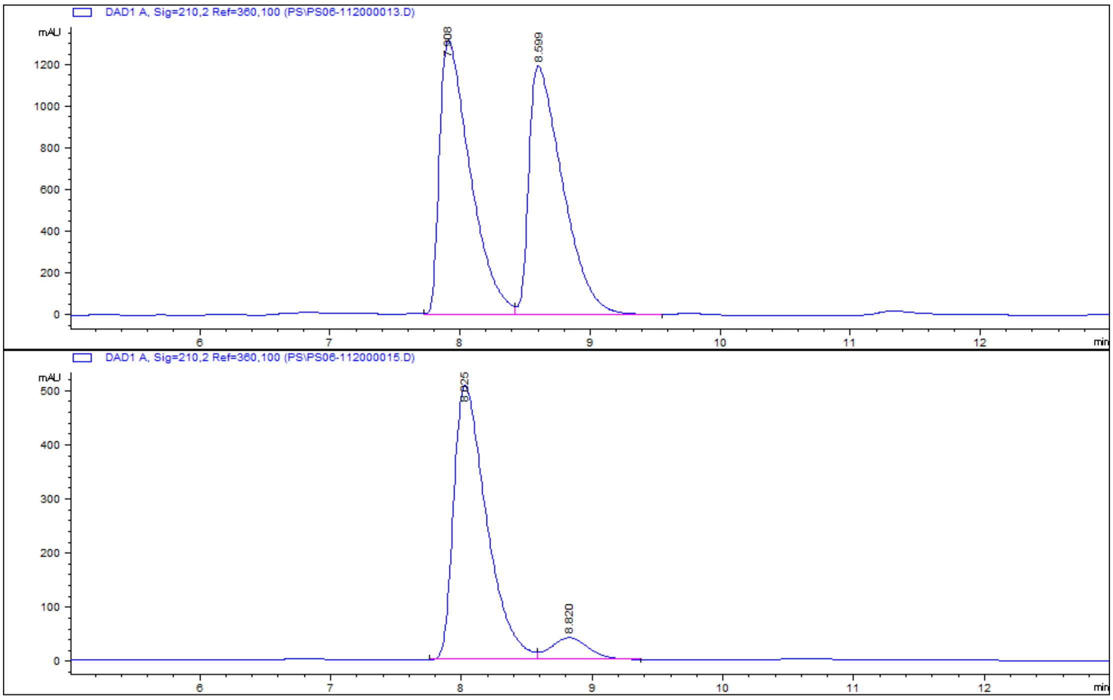

Supplementary figure 51: ^{1}H, ^{13}C-NMR spectra, HPLC traces of compound ***N-tert*-Butoxycarbonyl-5-chloro-3-piperidene**

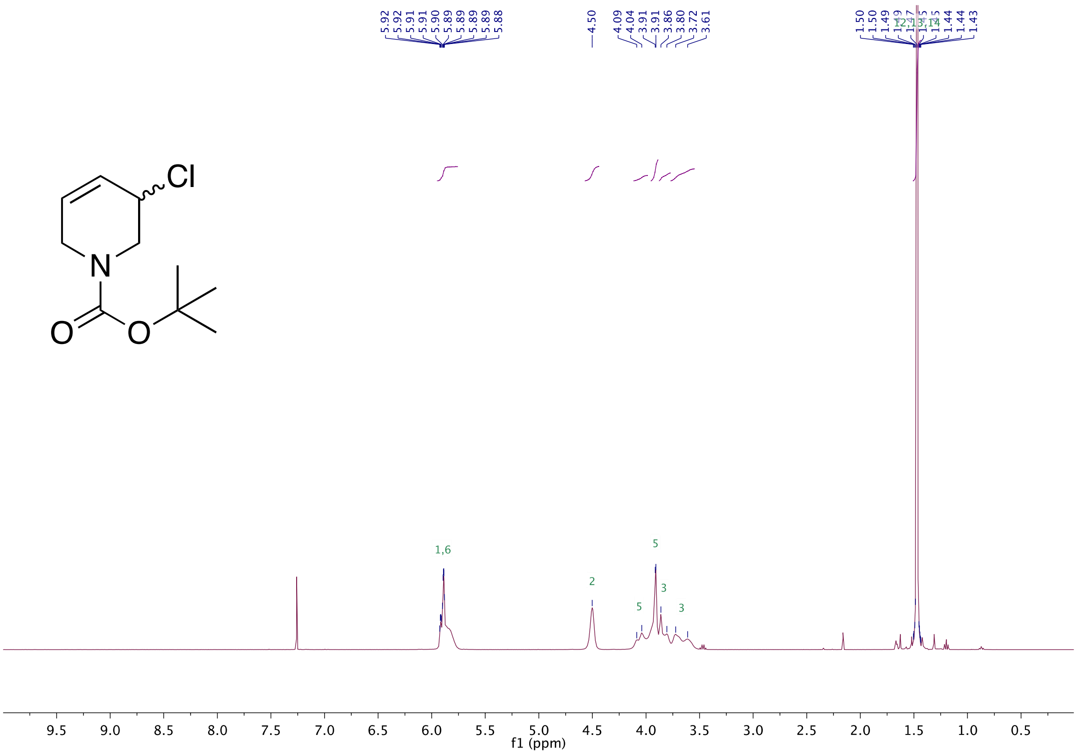

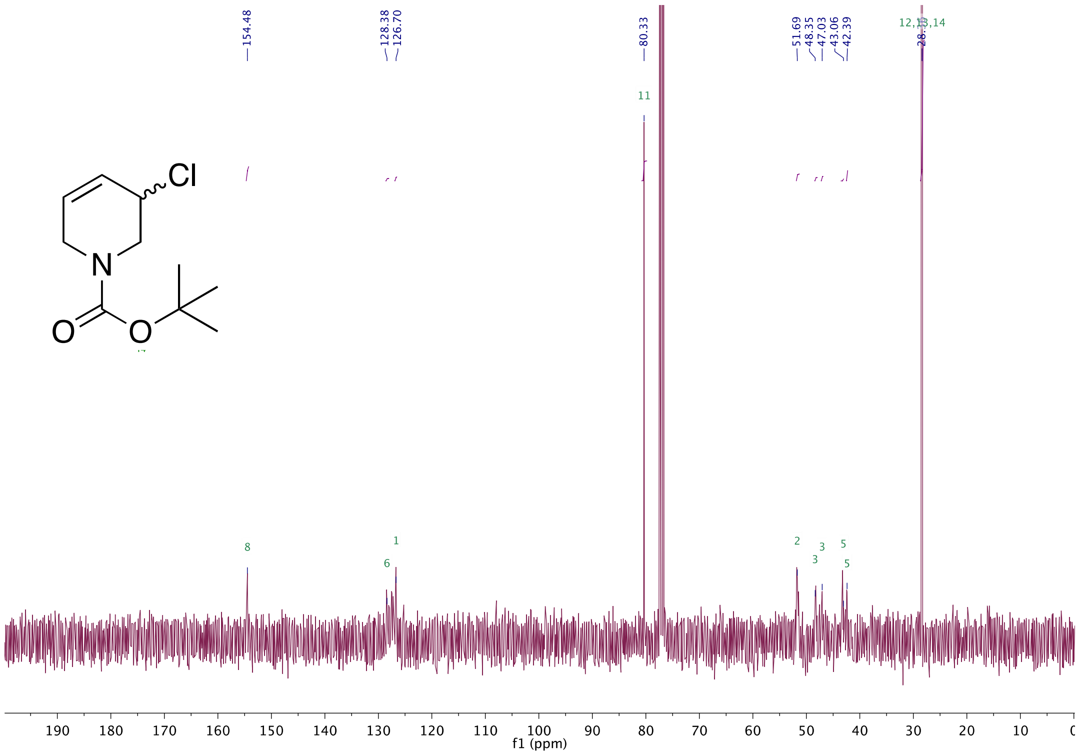
Cl
N
O
O
154.48
128.38
126.70
80.33
51.69
48.35
47.03
43.06
42.39
12,13,14
11
8
6
1
2
3
3
5
5
190 180 170 160 150 140 130 120 110 100 90 80 70 60 50 40 30 20 10
f1 (ppm)

Supplementary figure 52: Ligand screening for the asymmetric coupling using *N-tert*-butoxycarbonyl-5-chloro-3-piperidene:

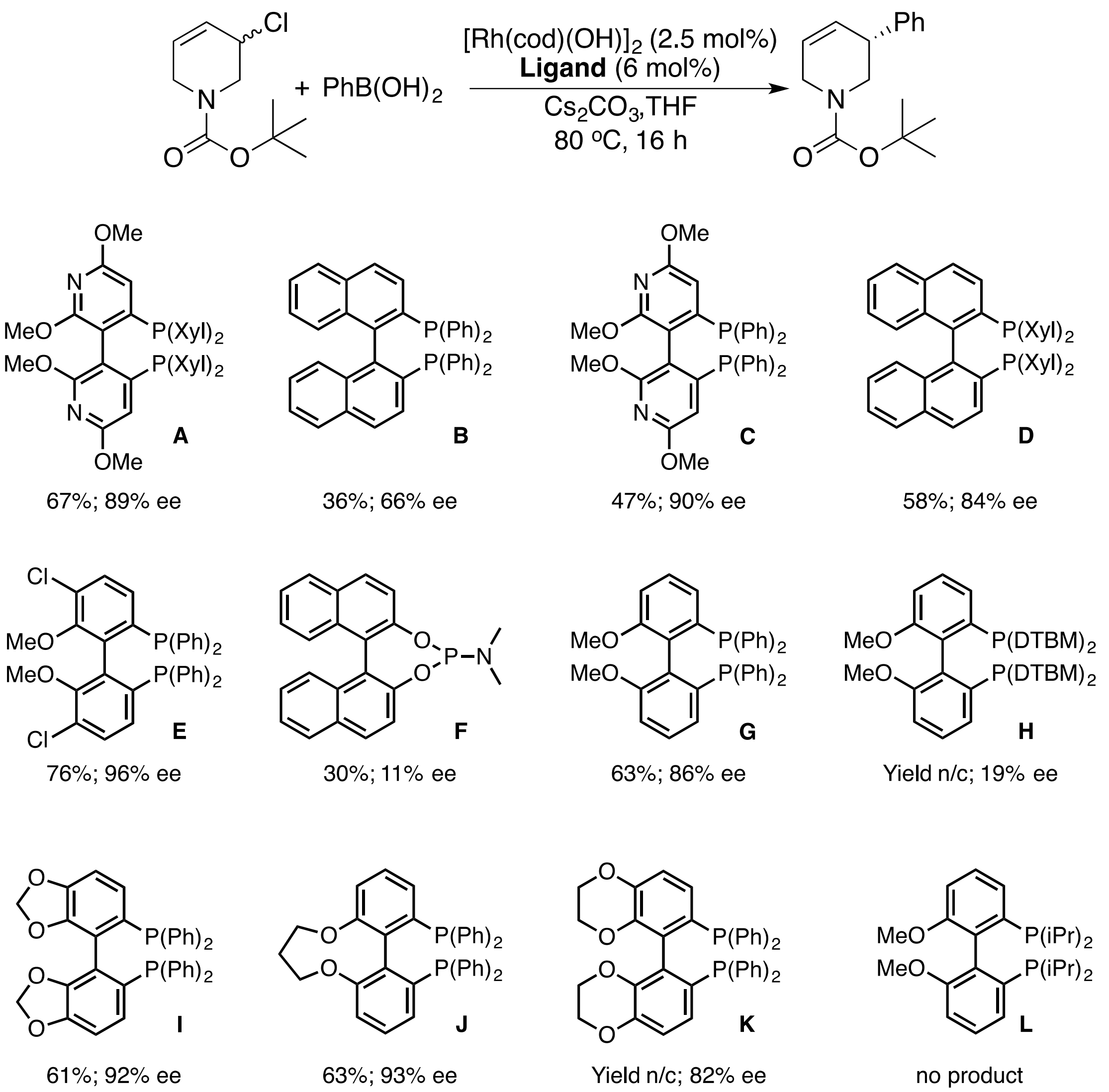

Supplementary figure 53: ^{1}H, ^{13}C-NMR spectra, HPLC traces of compound **50**

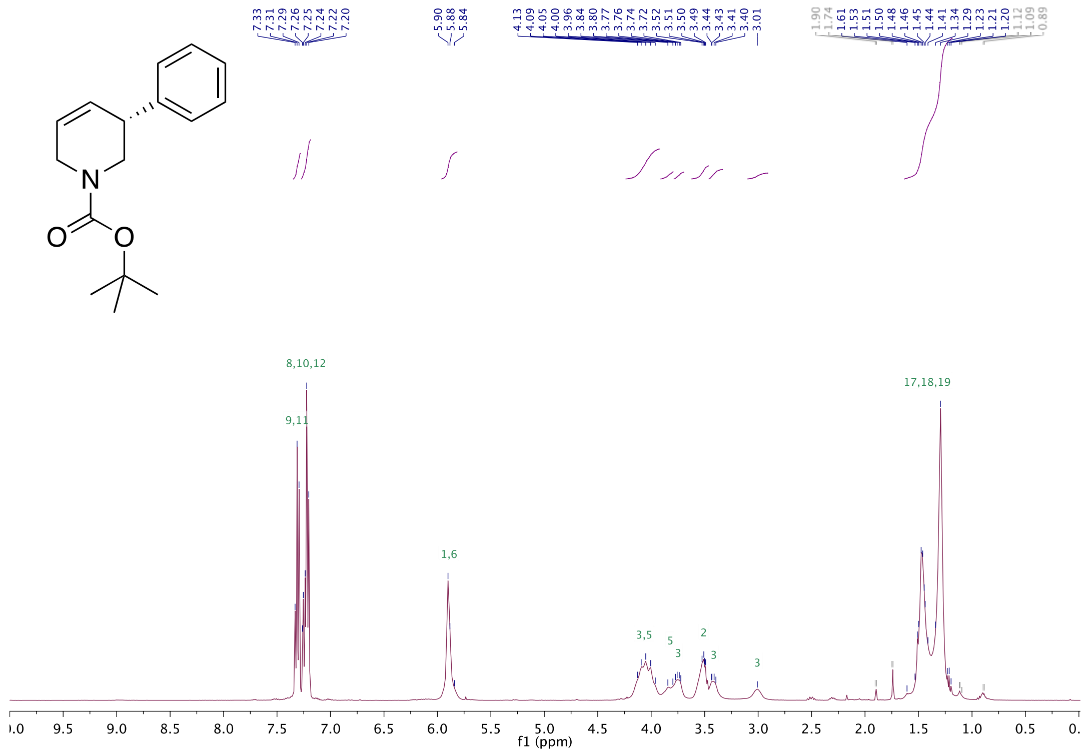

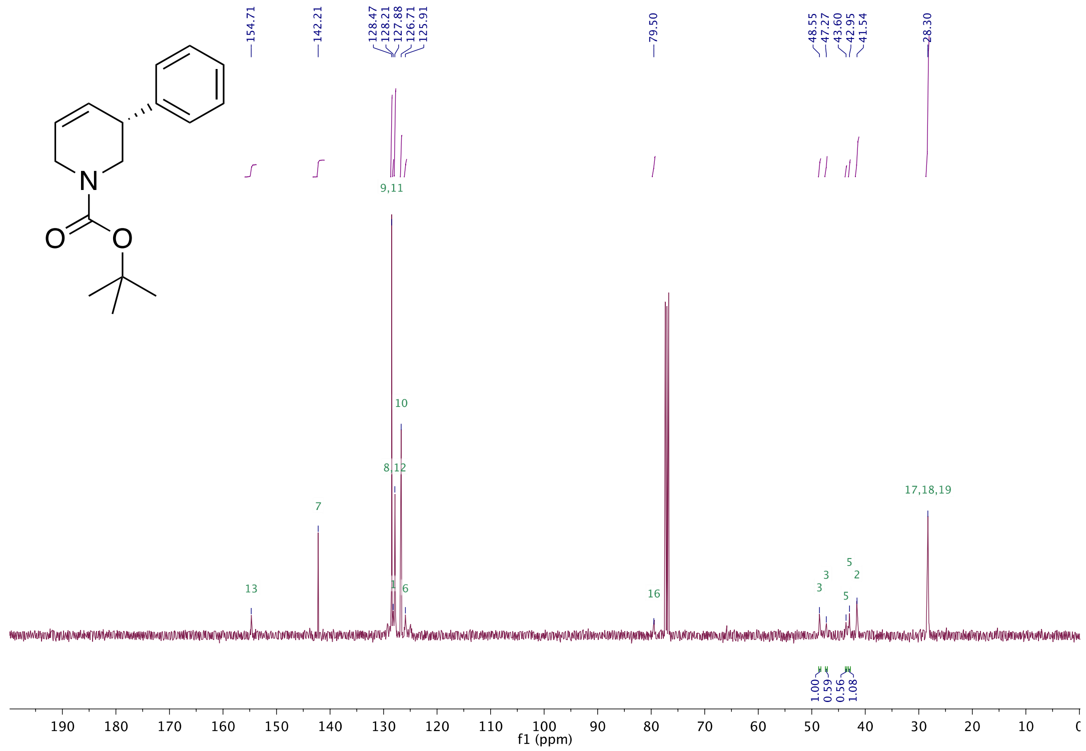

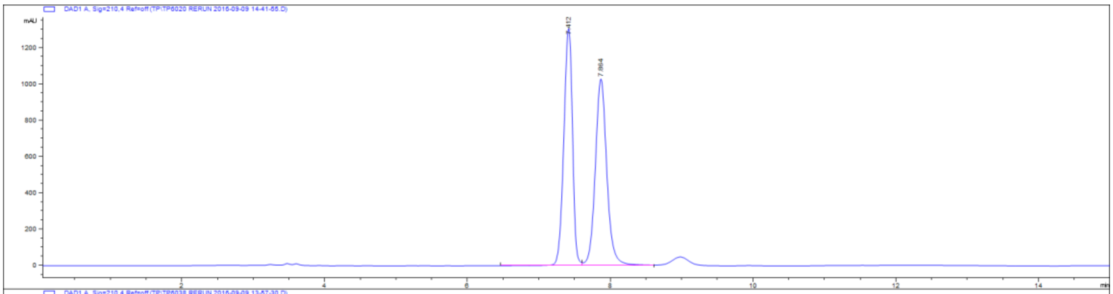
DAD1 A, Sig=210,4 Ref=off (TP\TP6020 RERUN 2016-09-09 14-41-55.D)
mAU
7.412
7.864
min

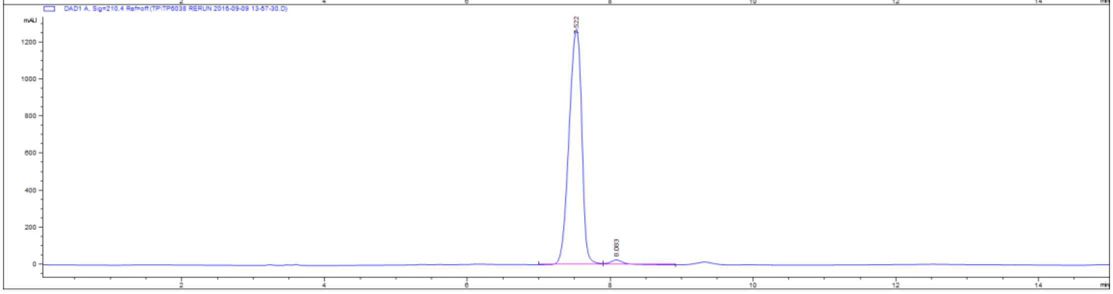
DAD1 A, Sig=210,4 Ref=off (TP\TP6038 RERUN 2016-09-09 13-57-30.D)
mAU
7.522
8.083

Supplementary figure 54: ^{1}H, ^{13}C-NMR spectra, HPLC traces of compound **51**

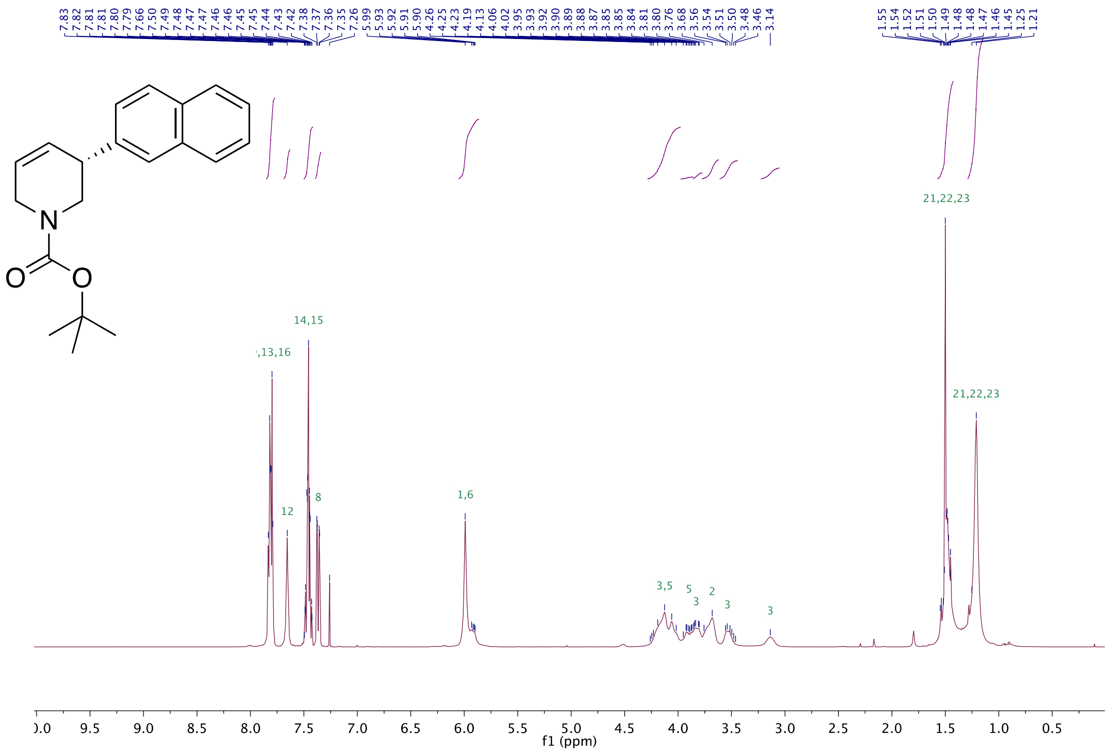

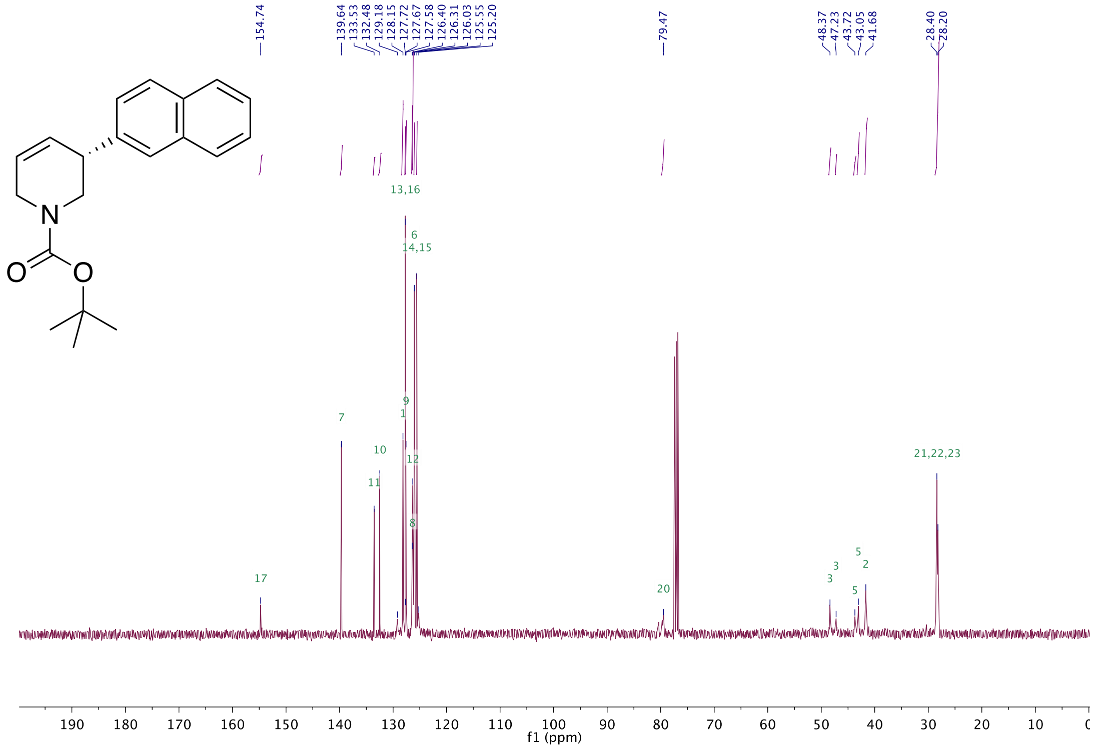

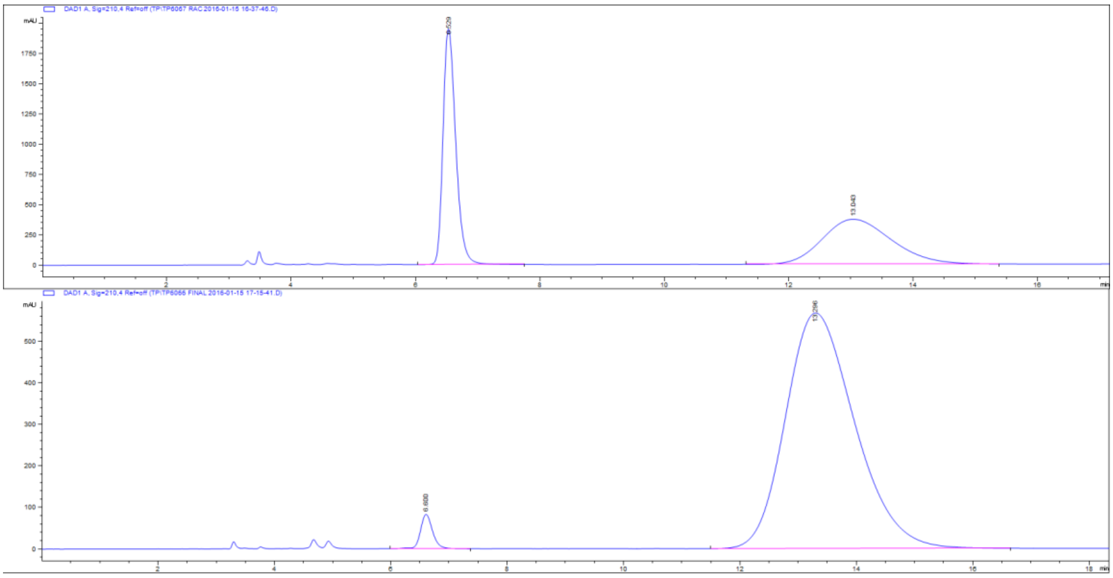

Supplementary figure 55: ^{1}H, ^{1}H-VT, ^{13}C, ^{13}C-VT-NMR spectra, HPLC traces of compound **52**

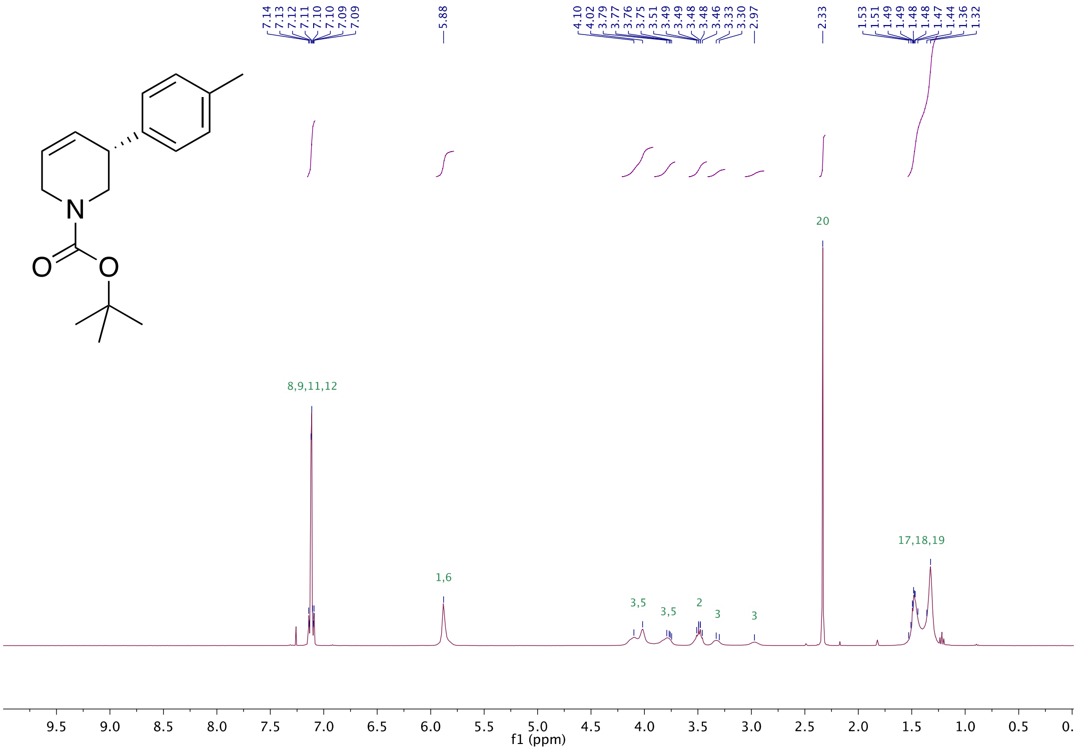

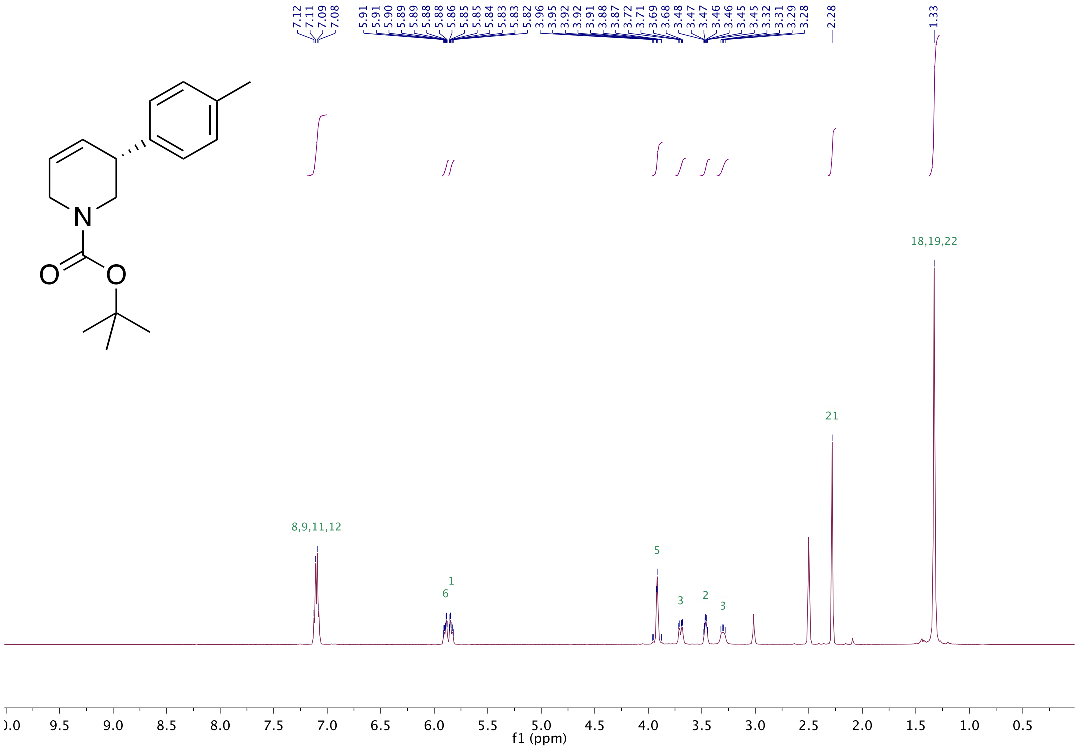

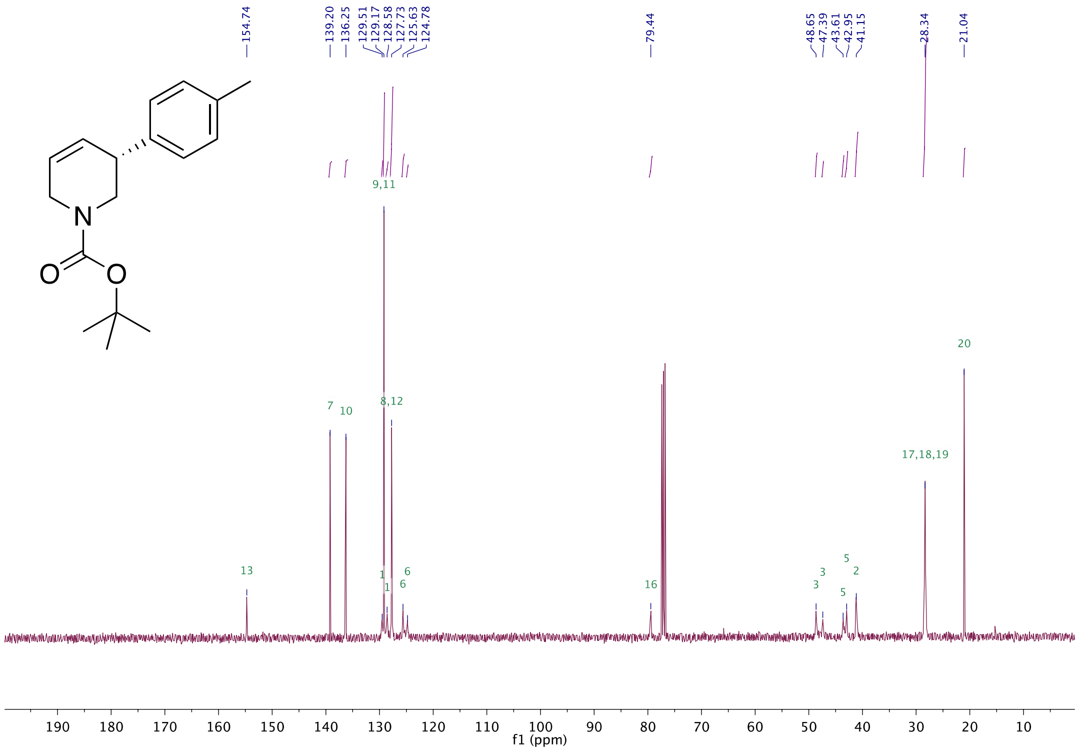

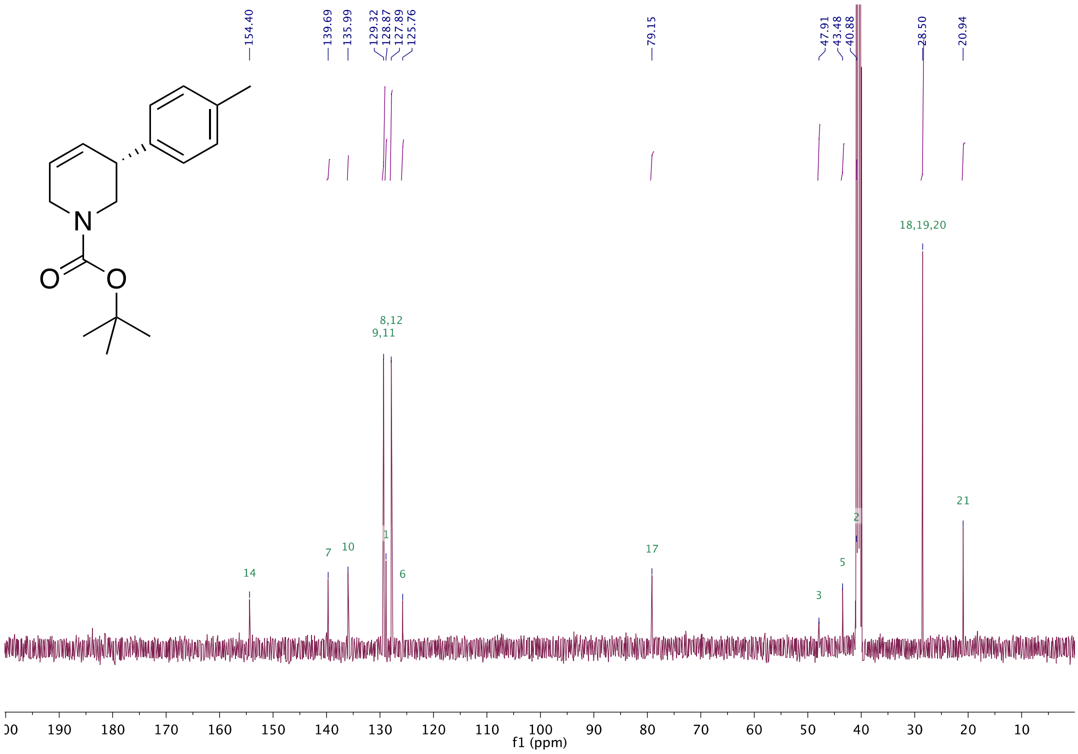
154.40
139.69
135.99
129.32
128.87
127.89
125.76
79.15
47.91
43.48
40.88
28.50
20.94
N
O
O
18,19,20
8,12
9,11
21
14
7
10
1
6
17
3
5
2
f1 (ppm)

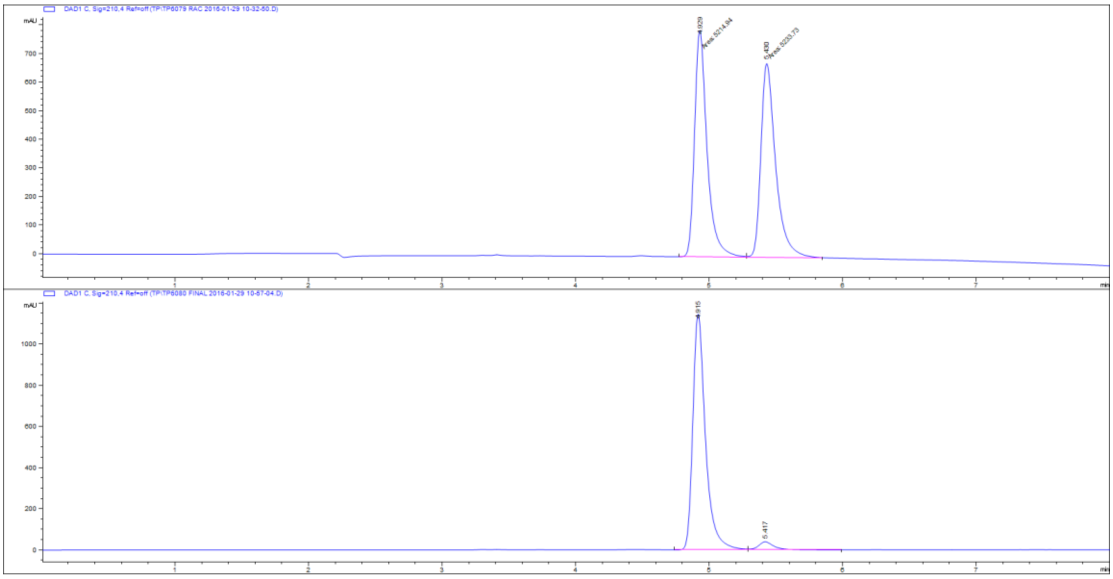
mAU
min

Supplementary figure 56: ^{1}H, ^{13}C-NMR spectra, HPLC traces of compound **53**

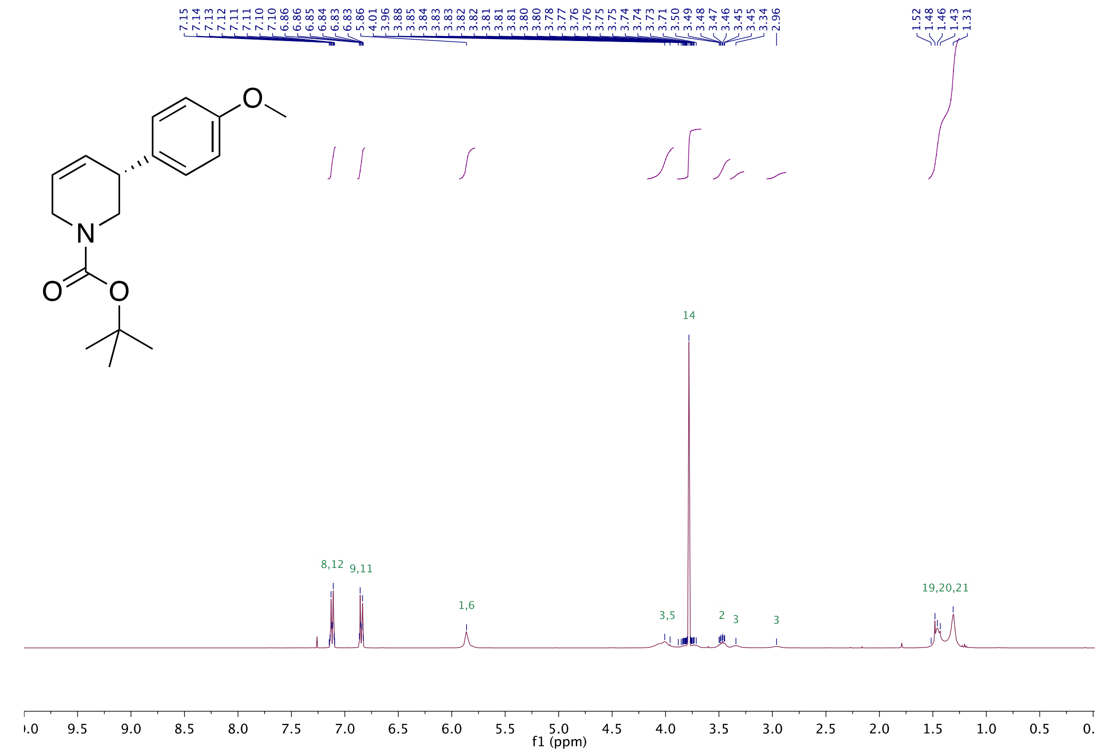

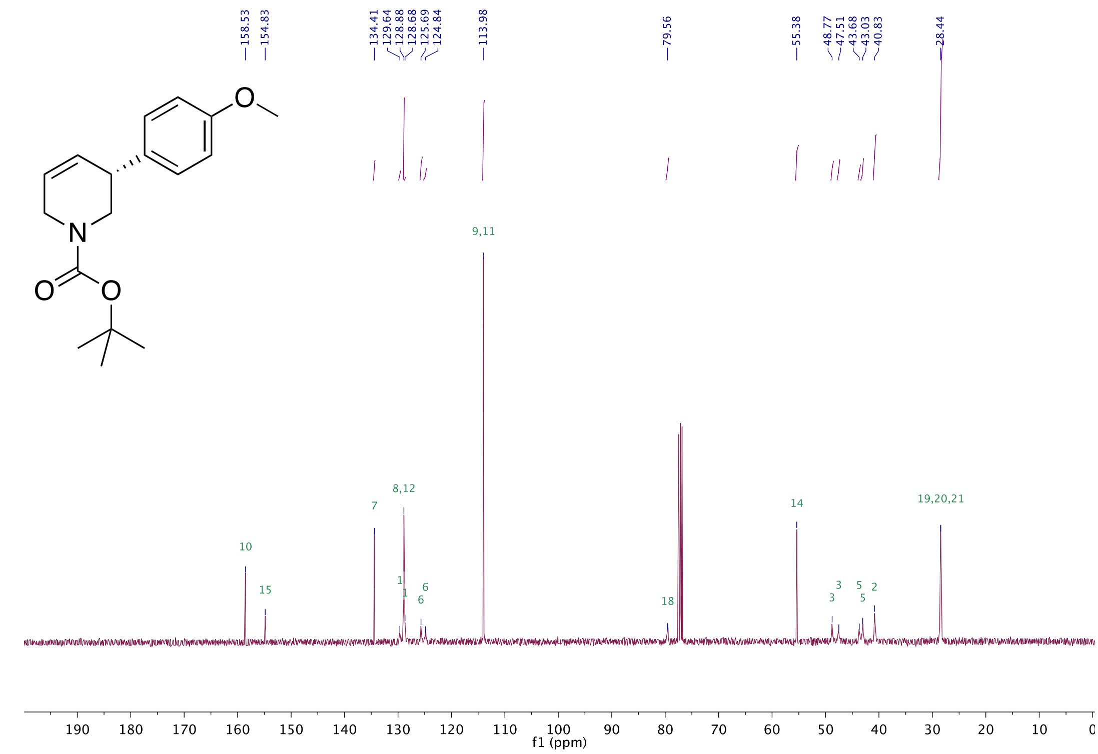

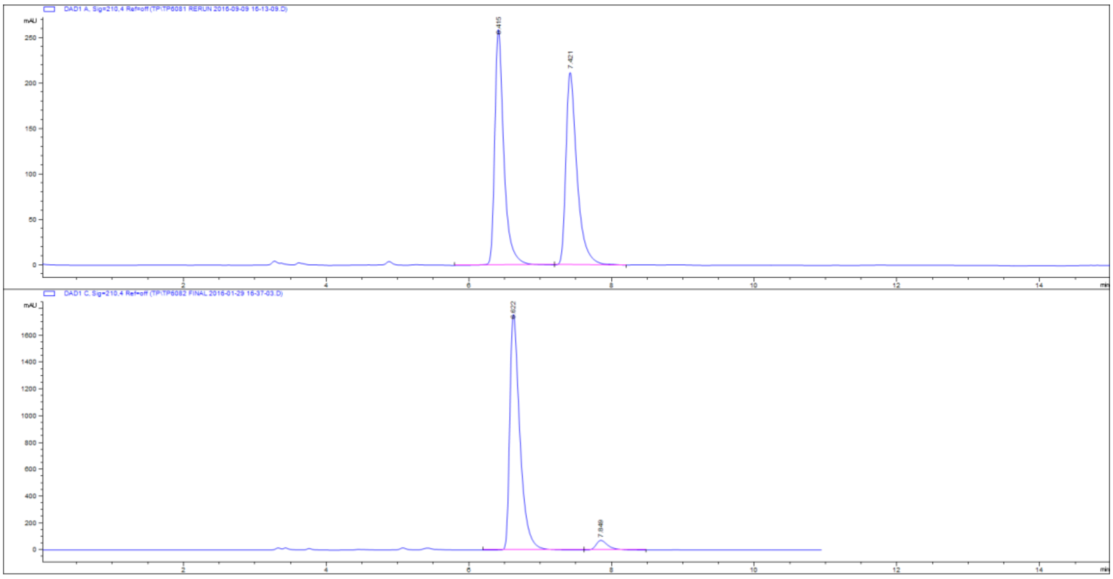

Supplementary figure 57: ^{1}H, ^{13}C-NMR spectra, HPLC traces of compound **54**

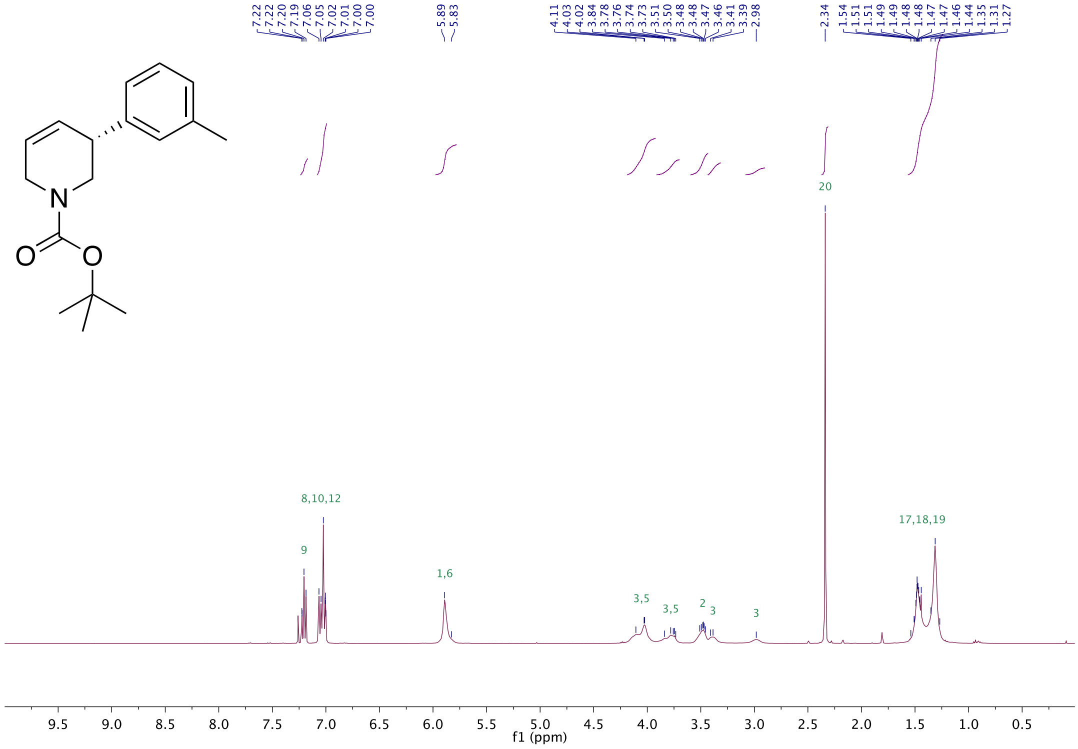

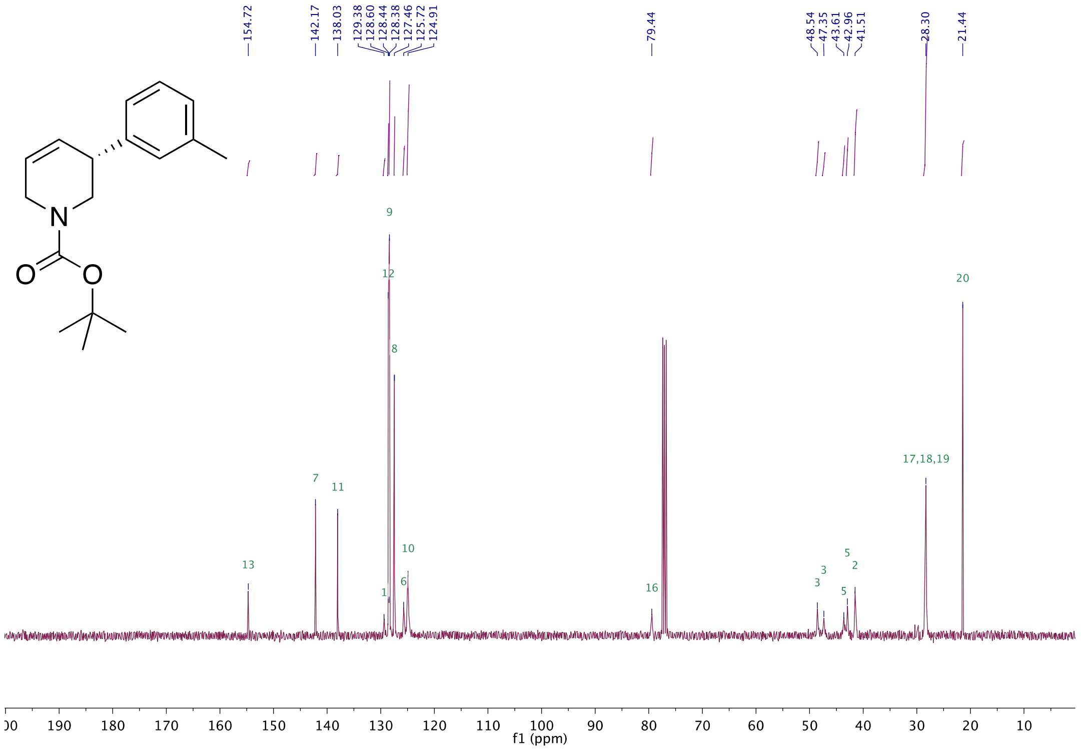

154.72
142.17
138.03
129.38
128.60
128.44
128.38
127.46
125.72
124.91
79.44
48.54
47.35
43.61
42.96
41.51
28.30
21.44
N
O
O
9
12
8
20
17,18,19
7
11
10
13
6
1
16
5
3
2
f1 (ppm)

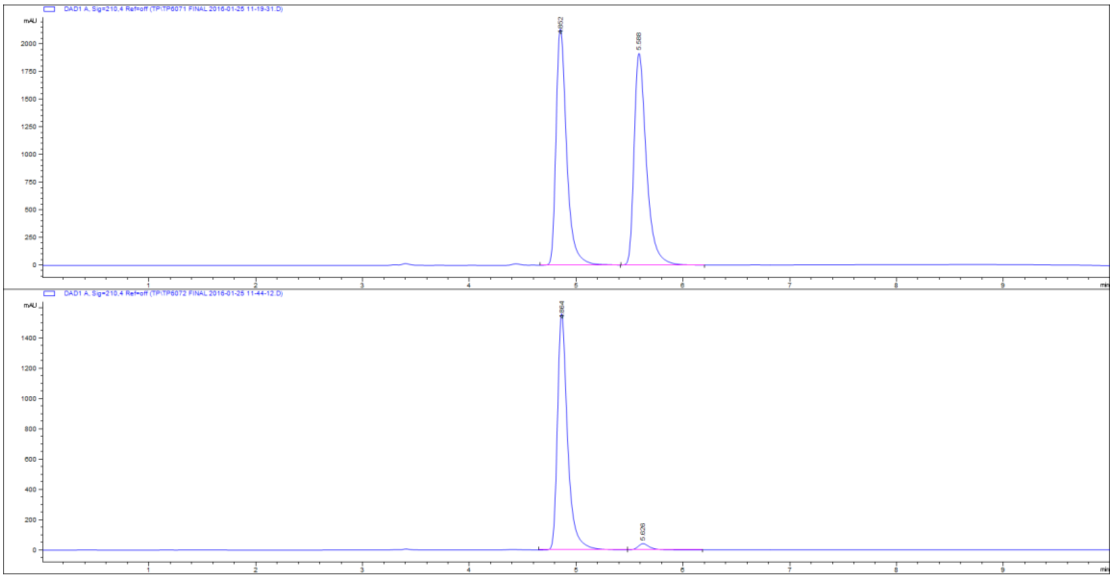

Supplementary figure 58: ^{1}H, ^{13}C-NMR spectra, HPLC traces of compound **55**

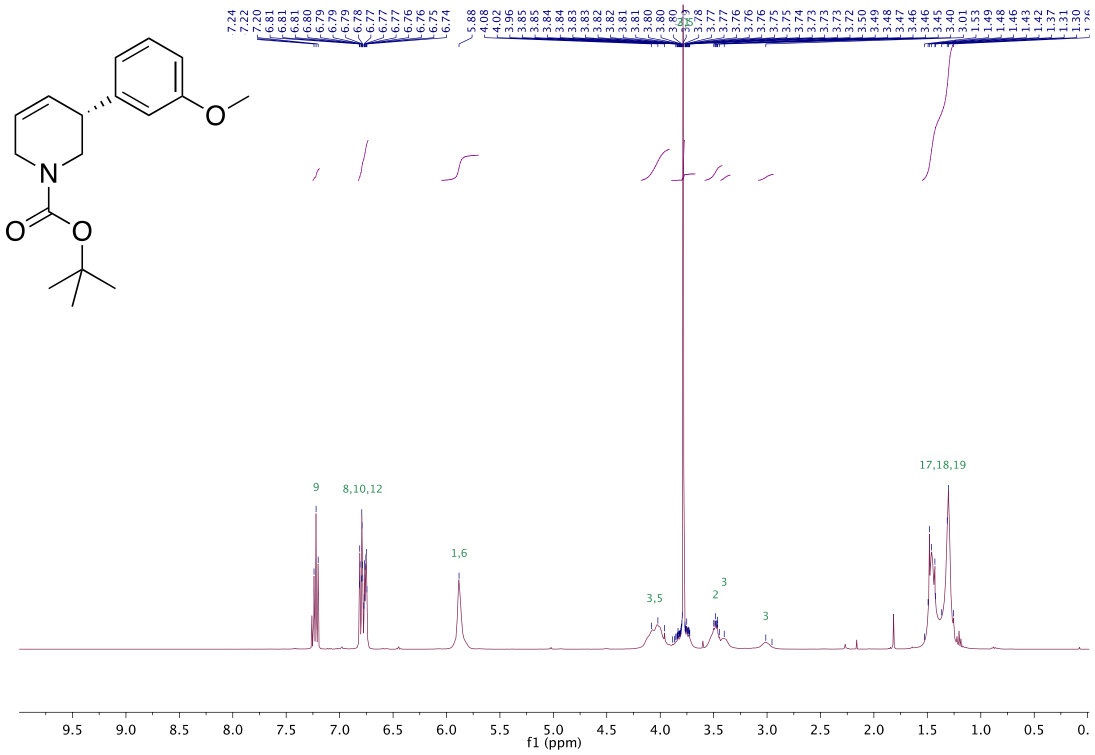

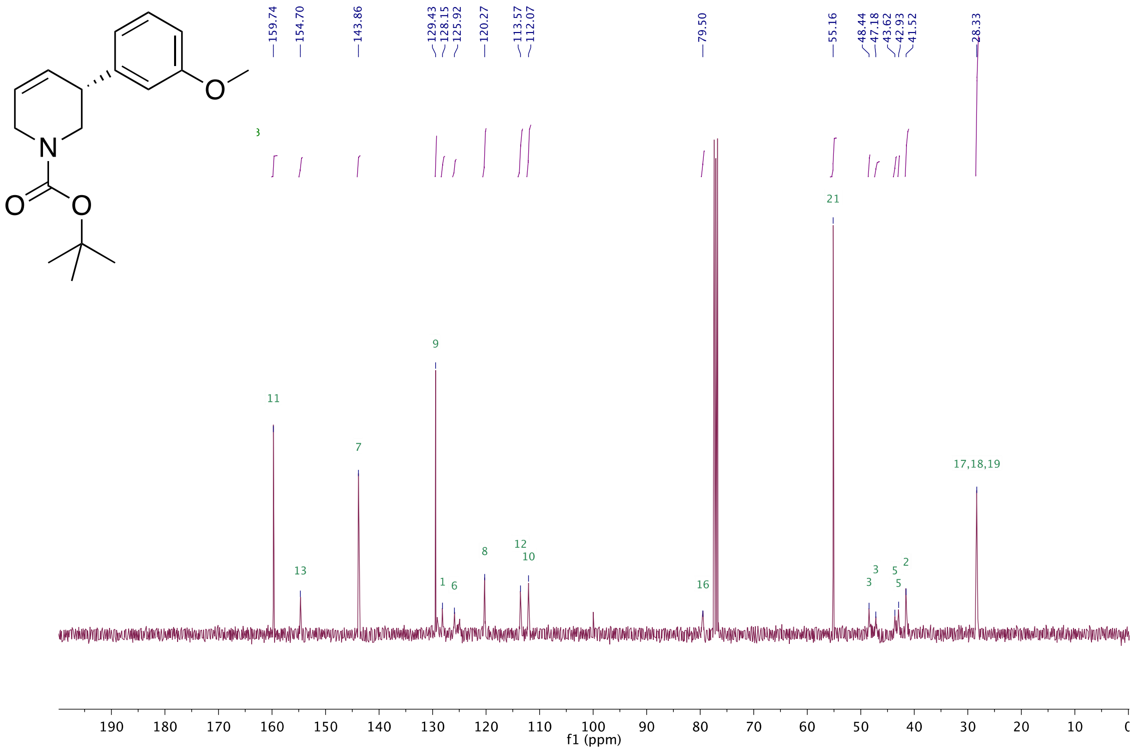

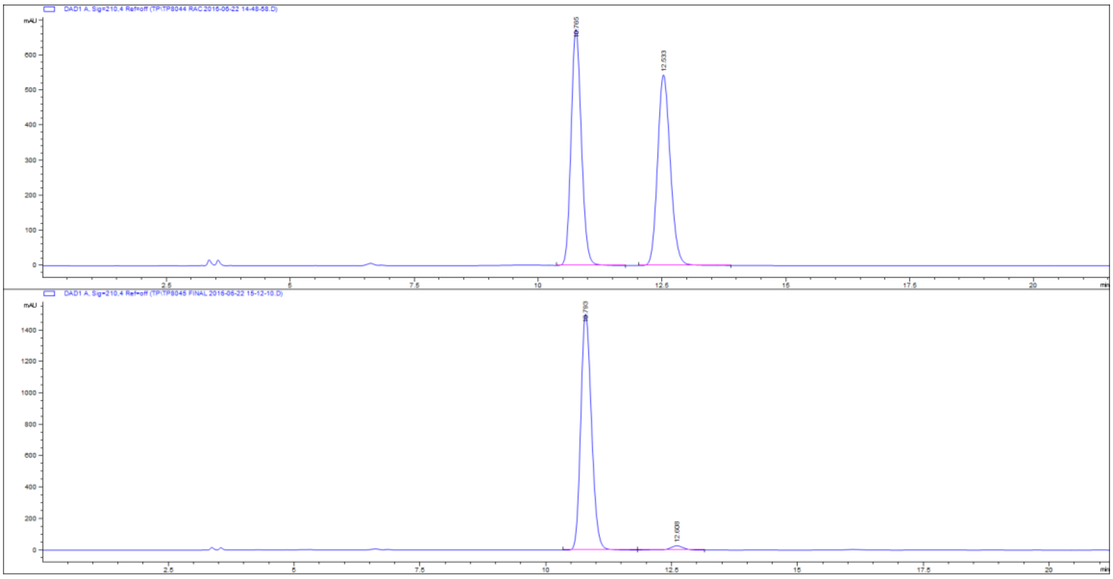

Supplementary figure 59: ^{1}H, ^{13}C-NMR spectra, HPLC traces of compound **56**

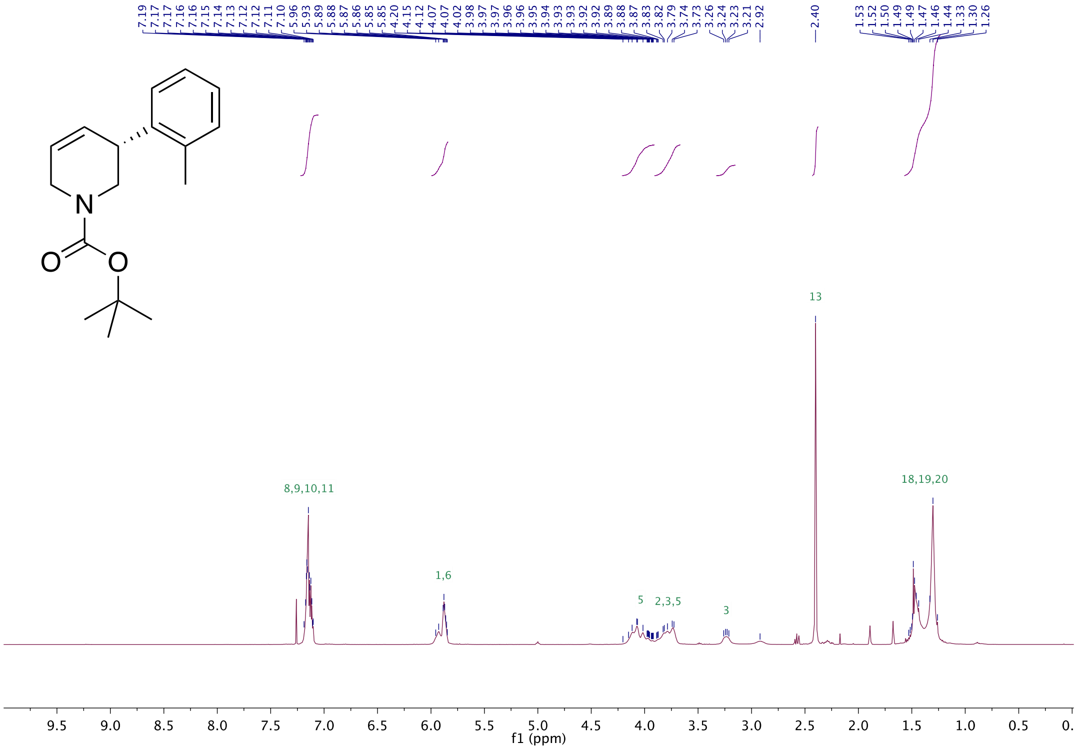

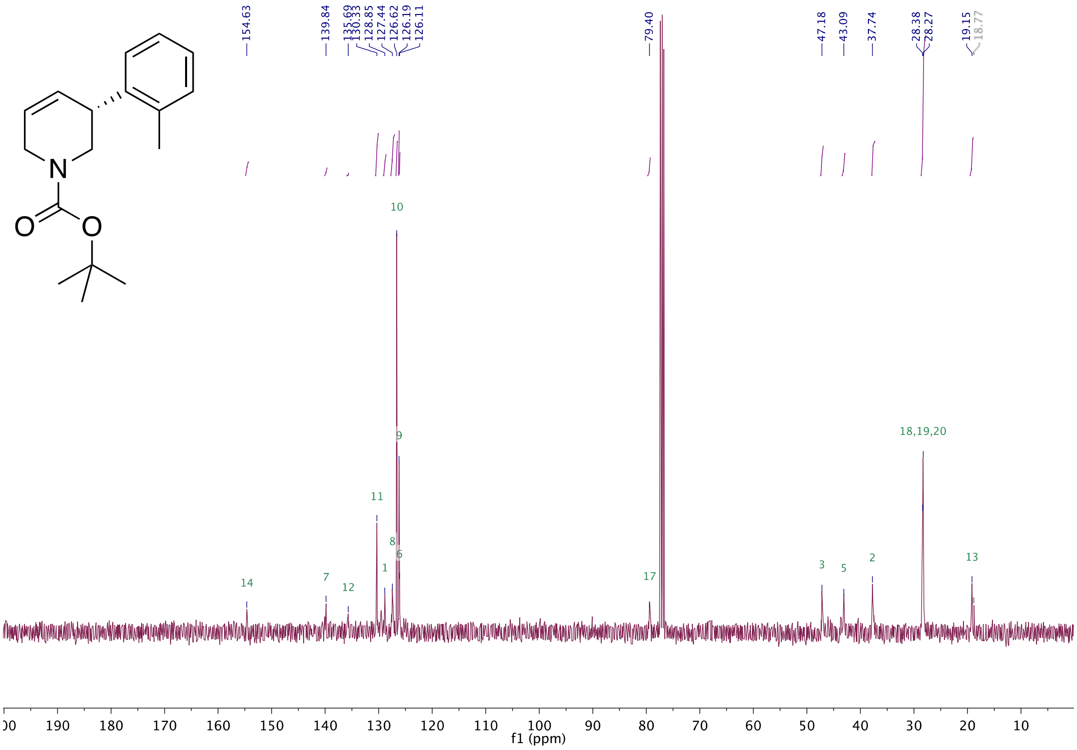

154.63
139.84
135.69
130.33
128.85
127.44
126.62
126.19
126.11
79.40
47.18
43.09
37.74
28.38
28.27
19.15
18.77
14
7
12
11
1
8
10
9
6
17
3
5
2
18,19,20
13
f1 (ppm)

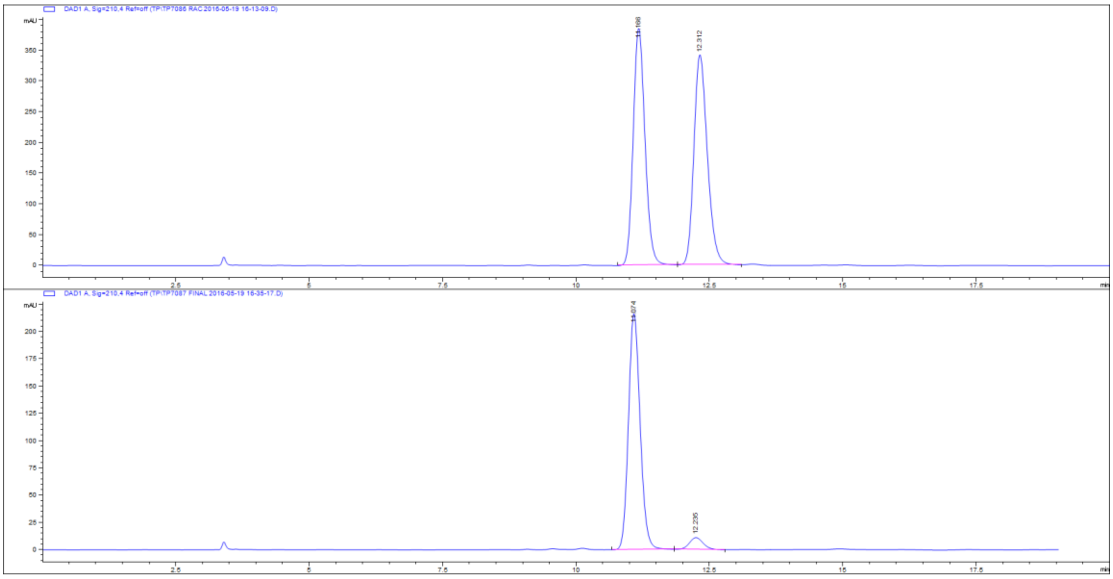

11.198
12.312
11.074
12.235

Supplementary figure 60: ^{1}H, ^{13}C-NMR spectra, HPLC traces of compound **57**

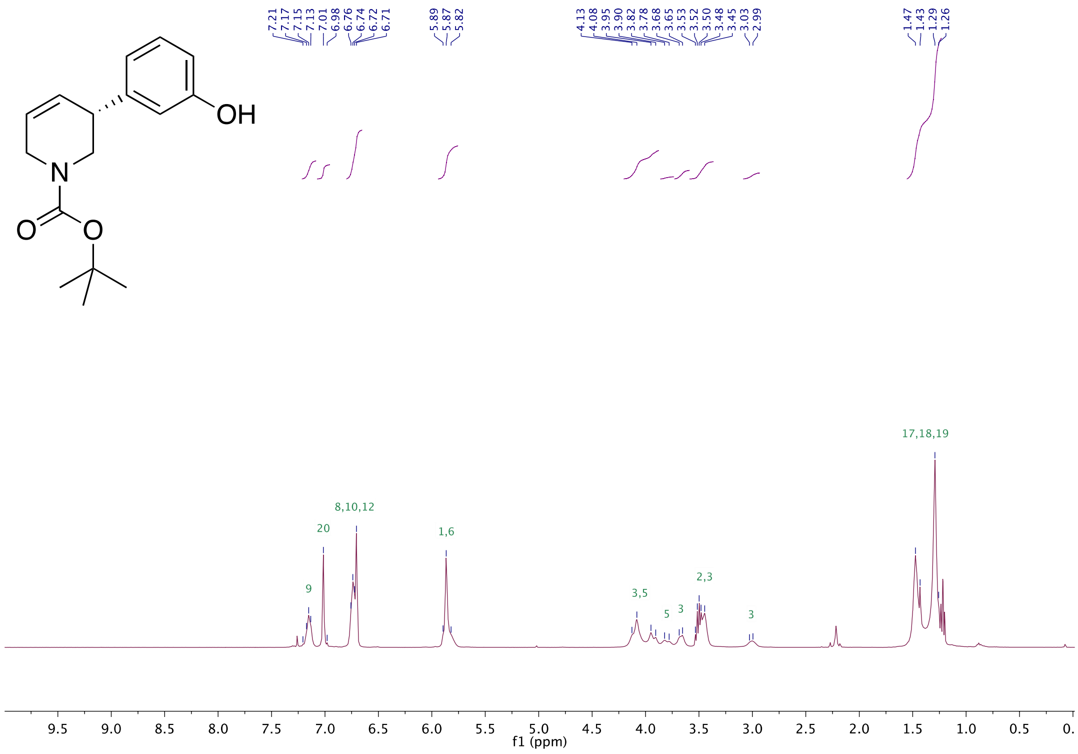

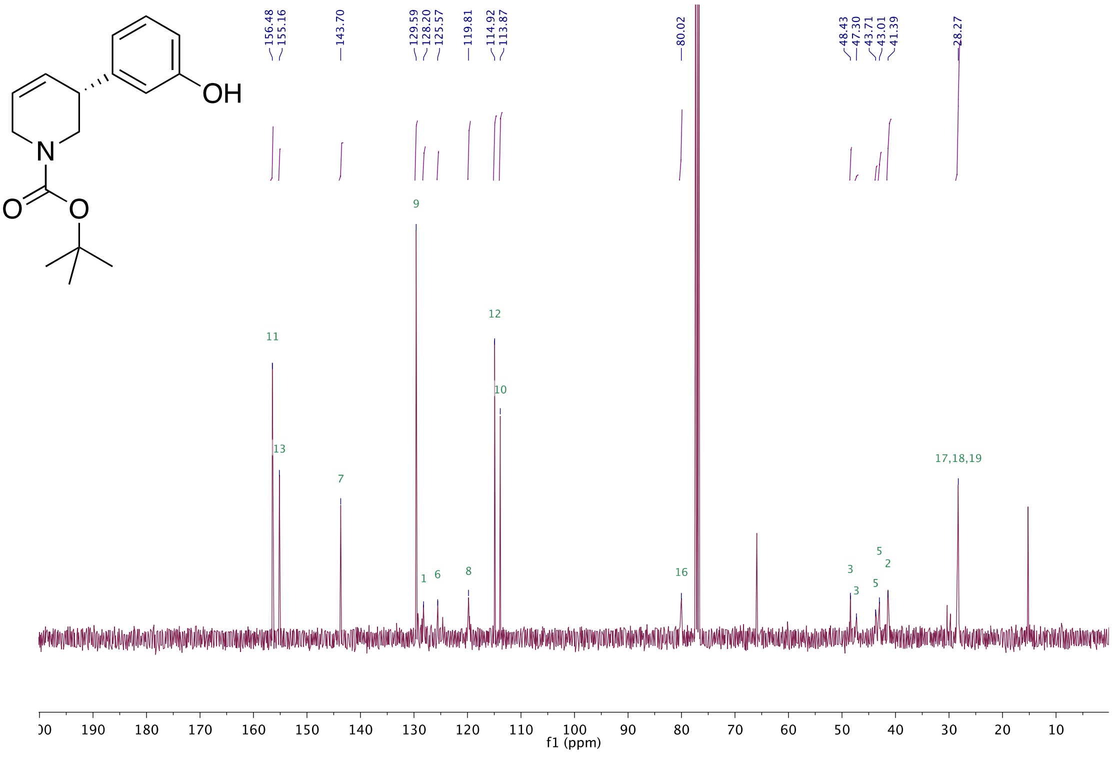

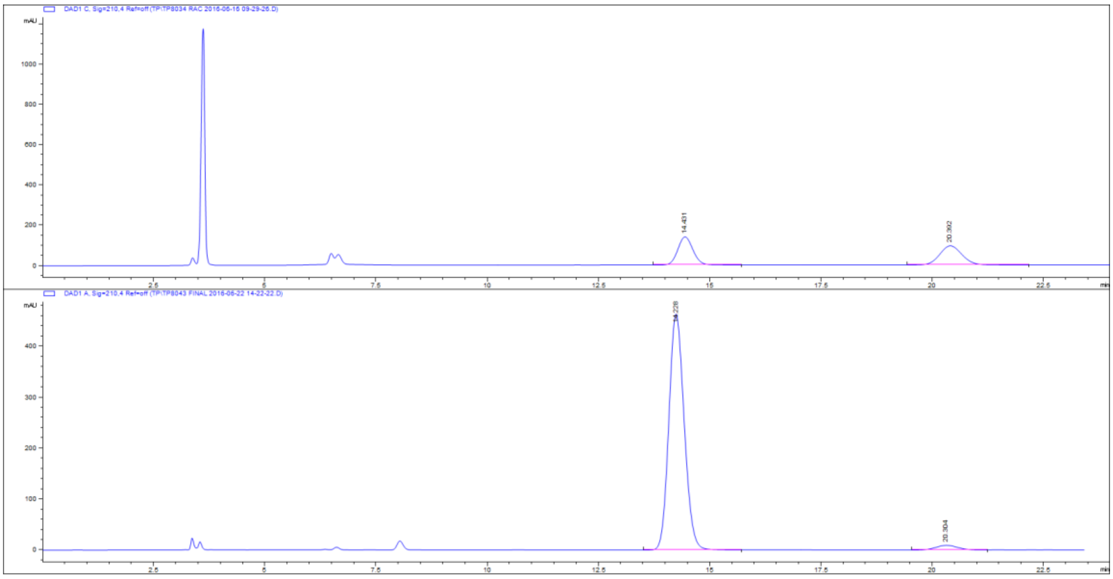

Supplementary figure 61: ^{1}H, ^{13}C, ^{19}F-NMR spectra, HPLC traces of compound **58**

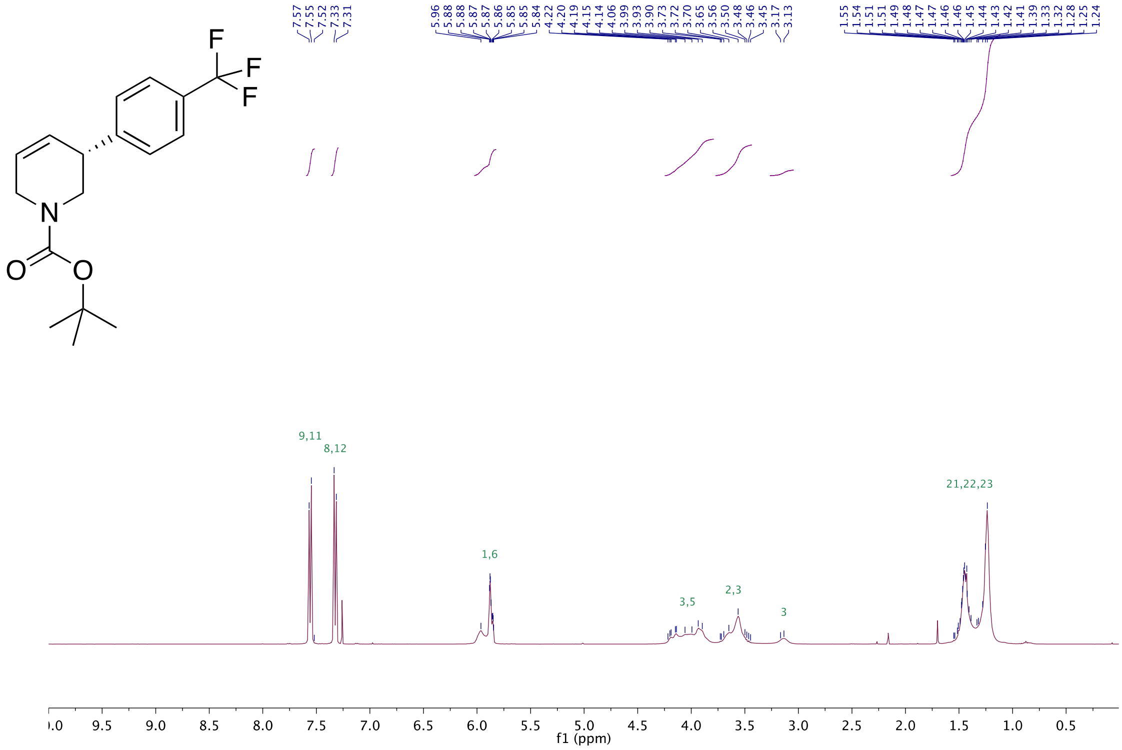

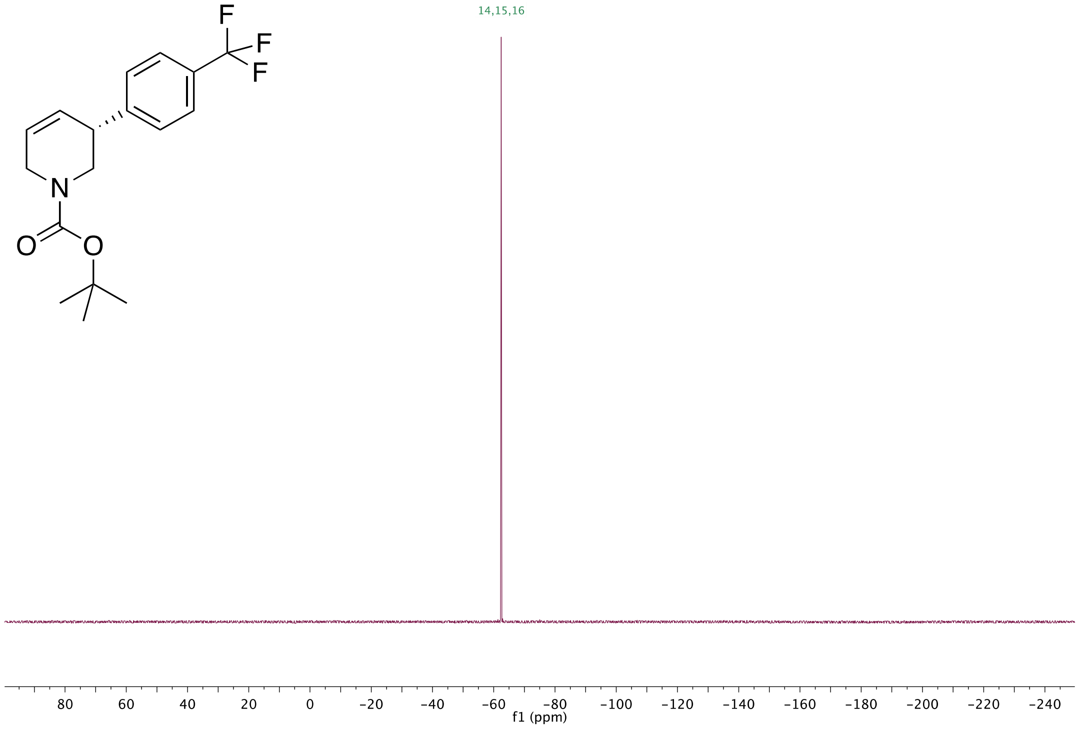

14,15,16
80
60
40
20
0
−20
−40
−60
−80
−100
−120
−140
−160
−180
−200
−220
−240
f1 (ppm)

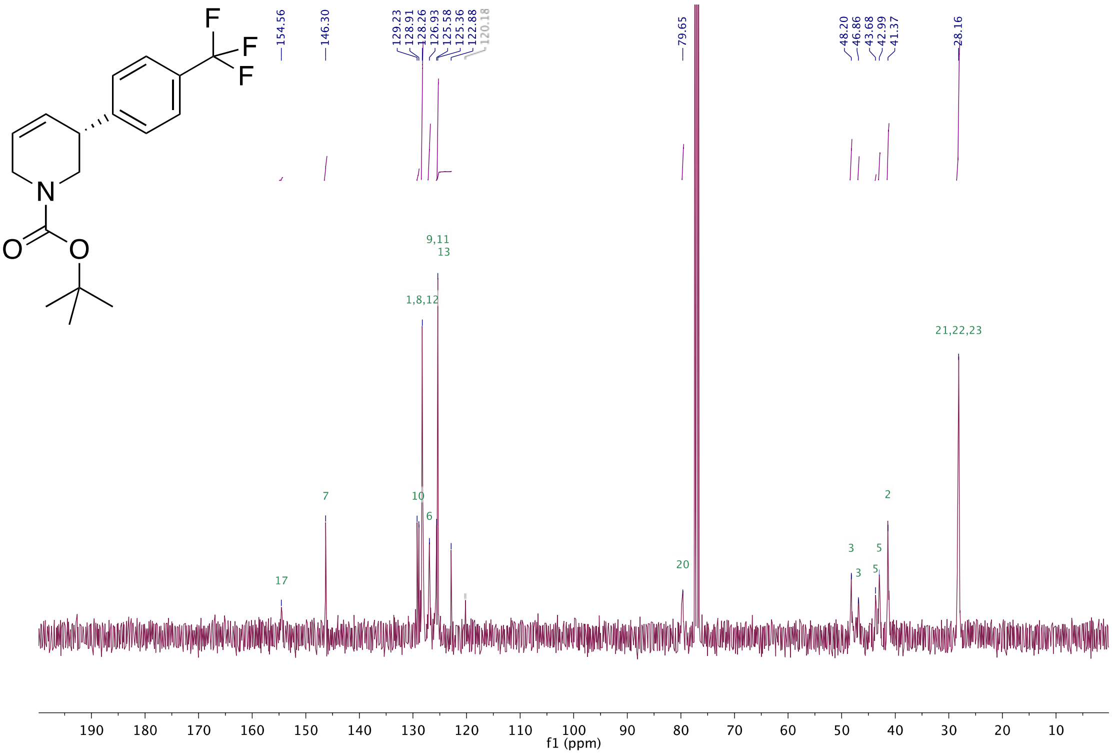

154.56
146.30
129.23
128.91
128.26
126.93
125.58
125.36
122.88
120.18
79.65
48.20
46.86
43.68
42.99
41.37
28.16
9,11
13
1,8,12
21,22,23
7
10
6
17
20
3
3
5
5
2
190
180
170
160
150
140
130
120
110
100
90
80
70
60
50
40
30
20
10
f1 (ppm)

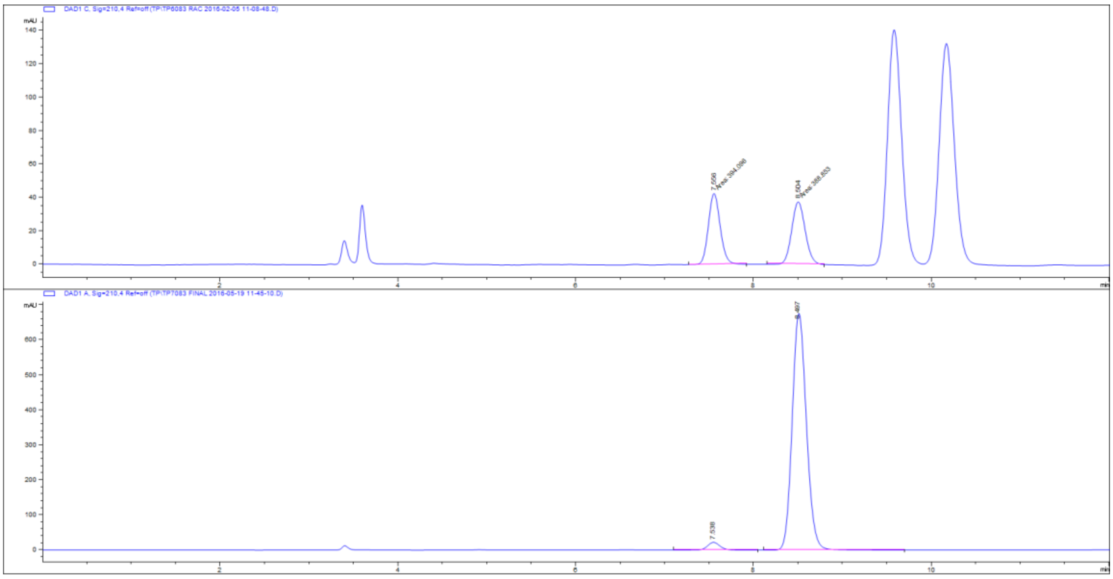

Supplementary figure 62: ^{1}H, ^{13}C-NMR spectra, HPLC traces of compound **59**

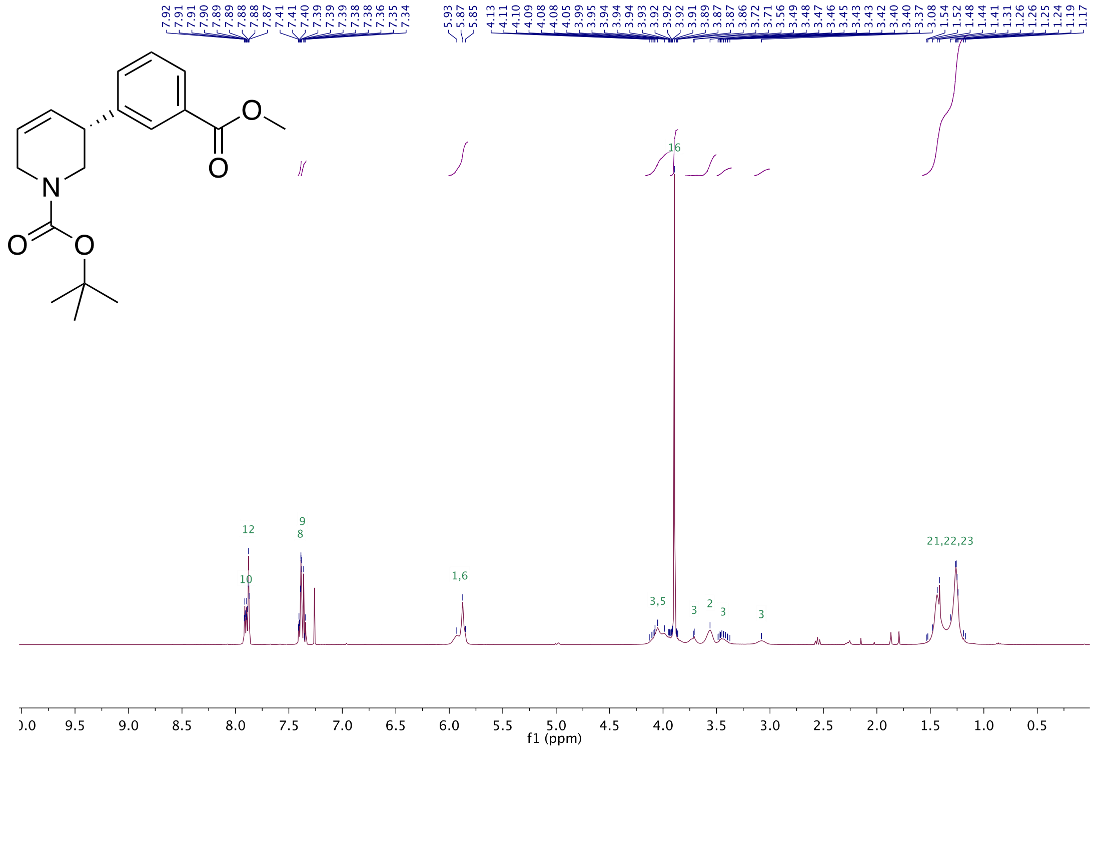

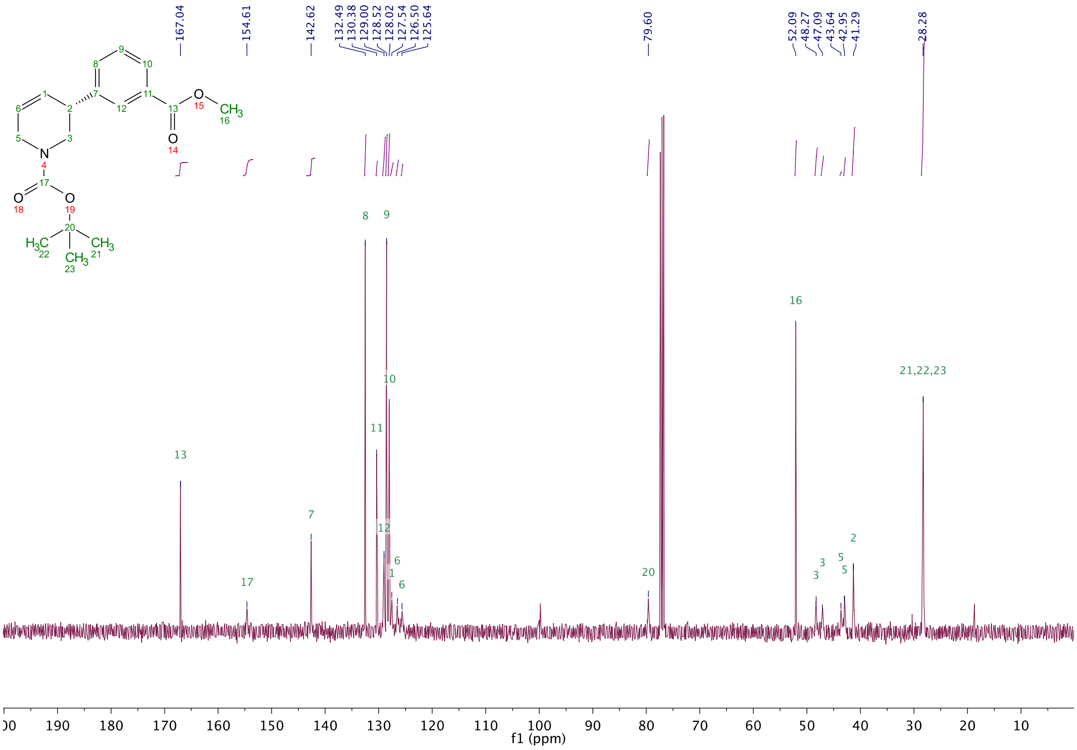
167.04
154.61
142.62
132.49
130.38
129.00
128.52
128.02
127.54
126.50
125.64
79.60
52.09
48.27
47.09
43.64
42.95
41.29
28.28
f1 (ppm)

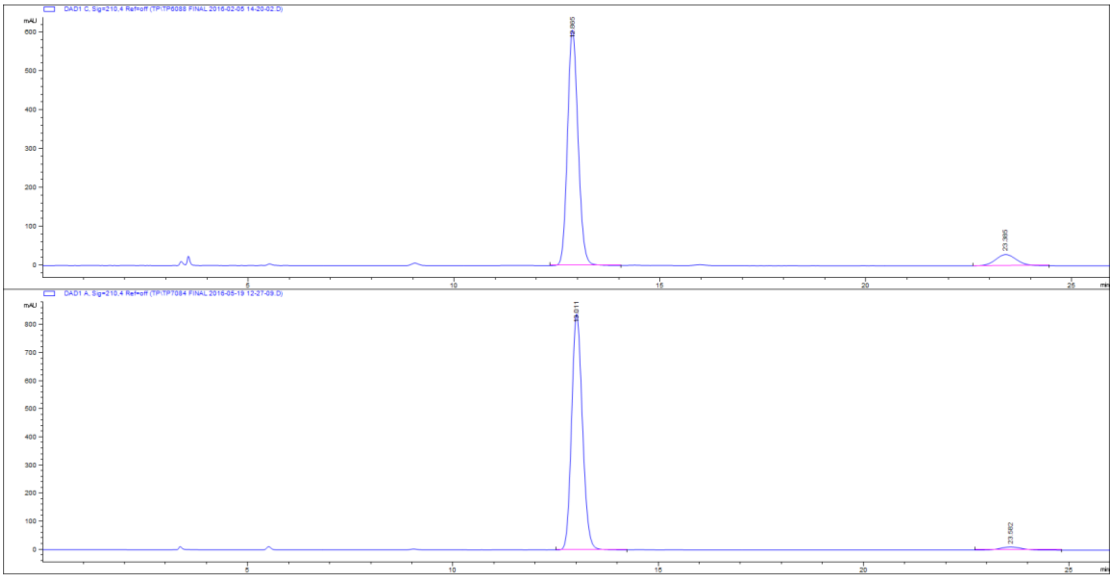
mAU
min

Supplementary figure 63: ^{1}H, ^{13}C-NMR spectra, HPLC traces of compound **60**

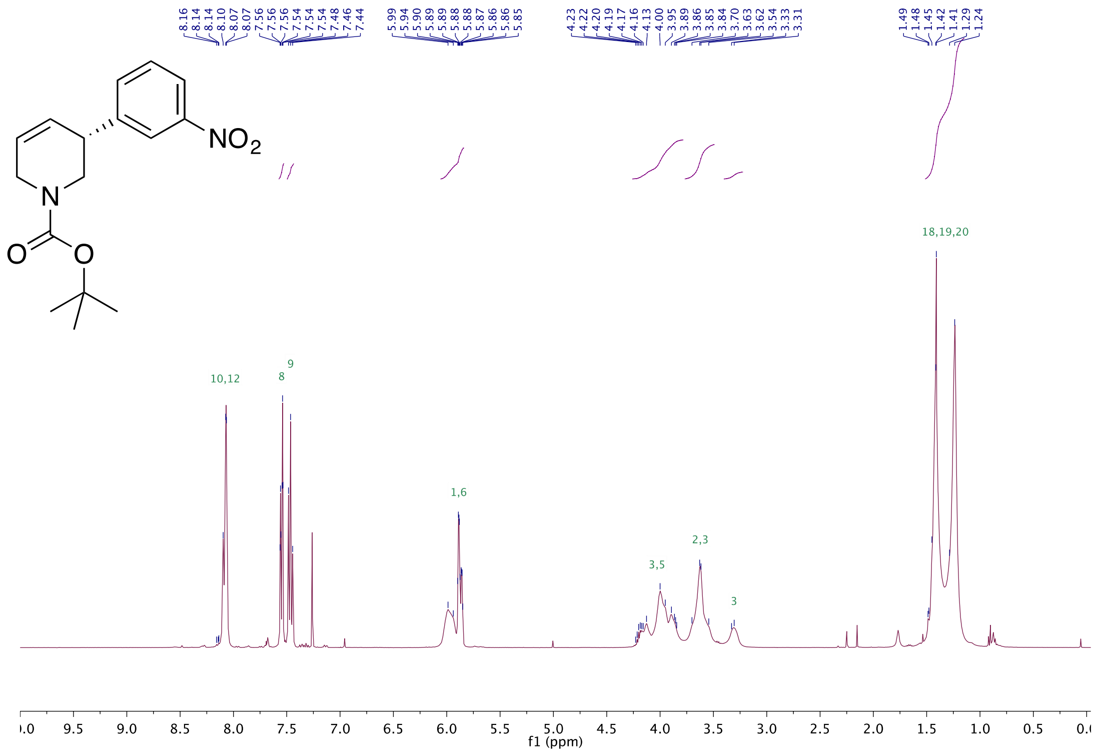

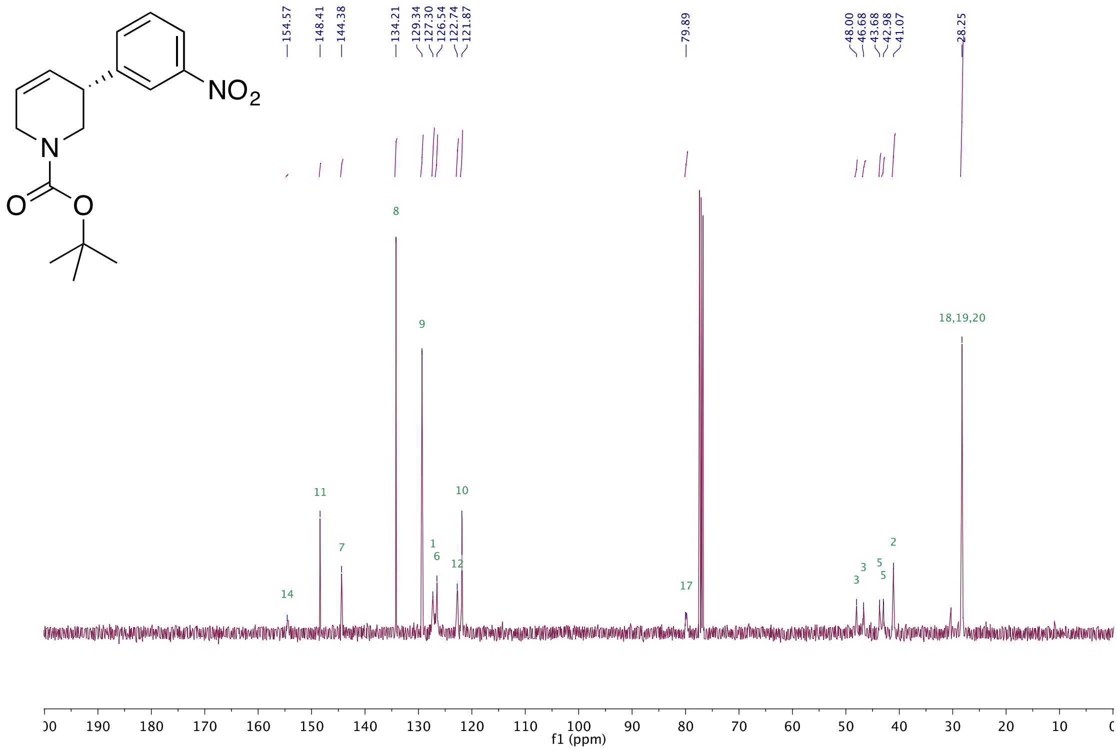

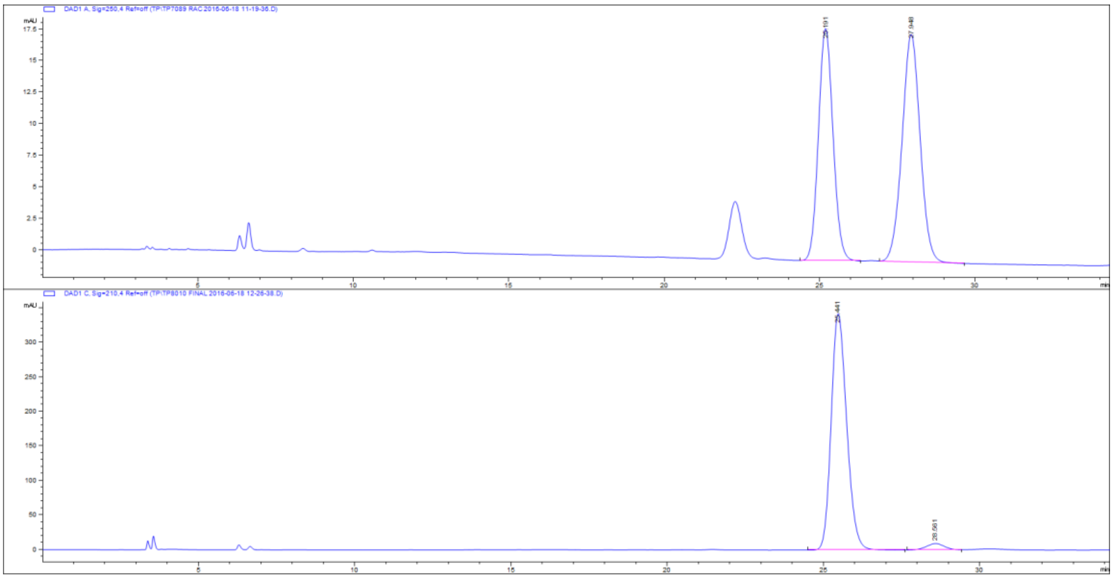

Supplementary figure 64: ^{1}H, ^{13}C, ^{19}F-NMR spectra, HPLC traces of compound **61**

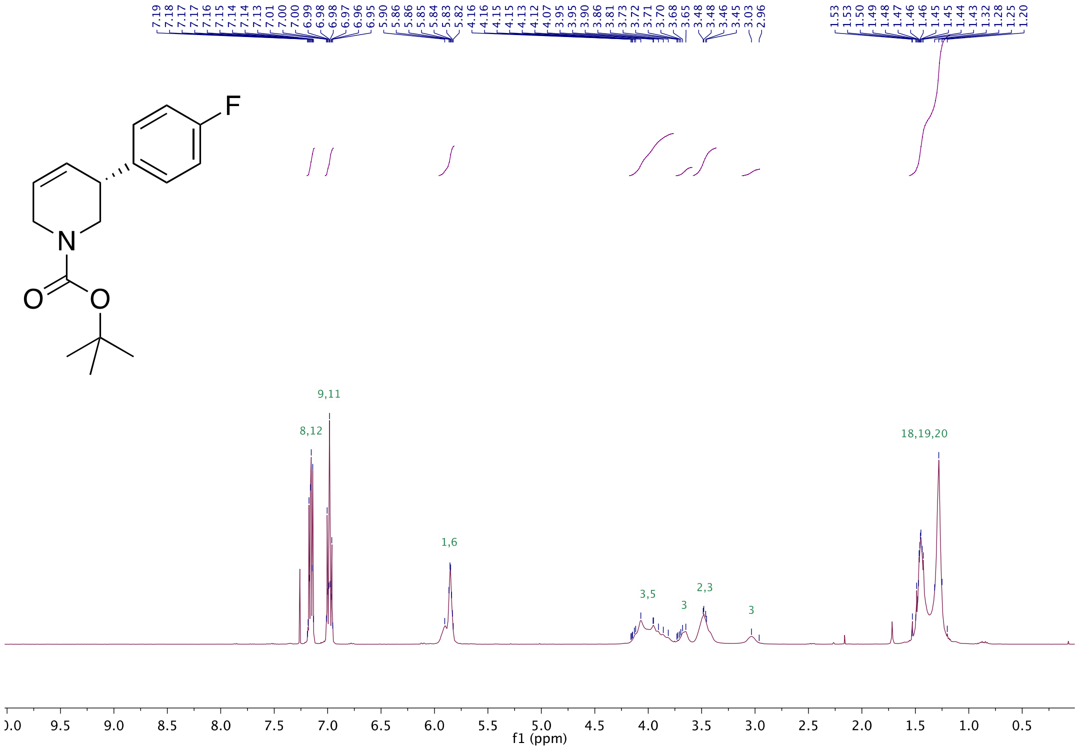

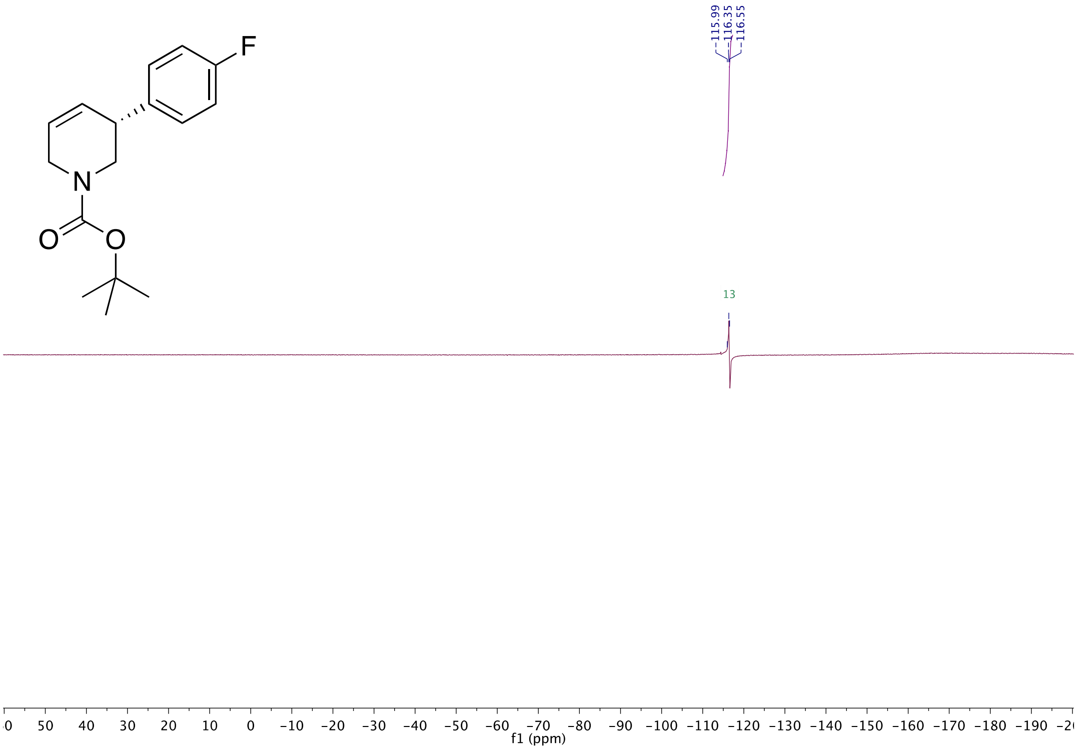

F
N
O
O
-115.99
-116.35
-116.55
13
f1 (ppm)

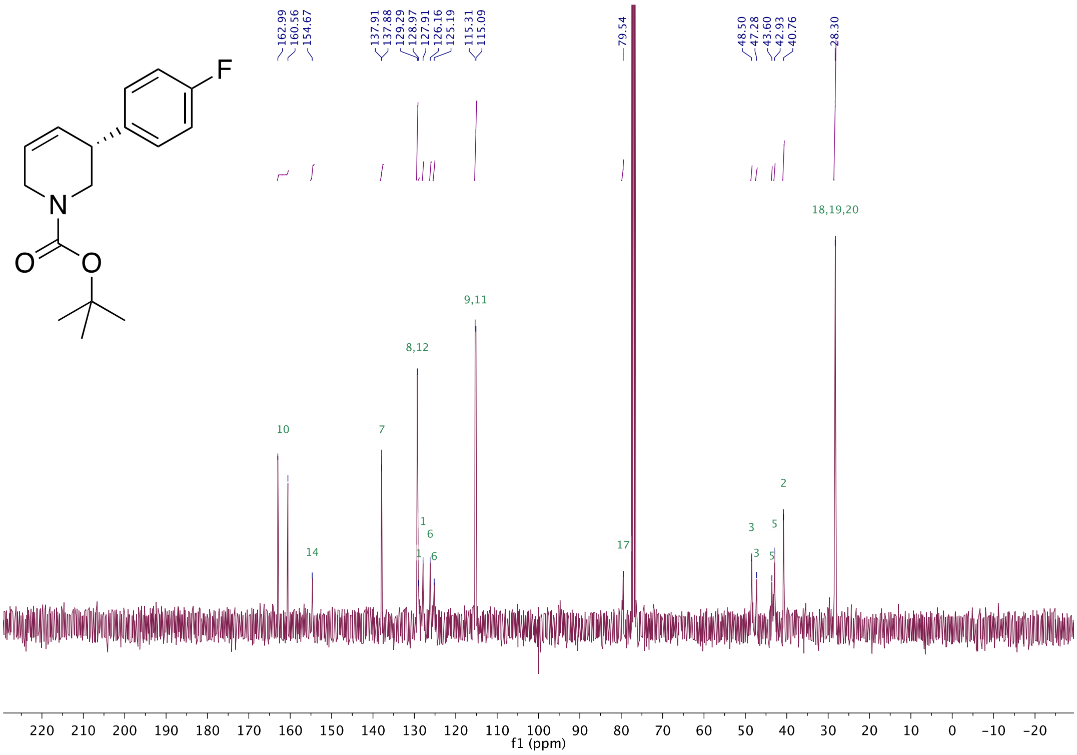

F
N
O
O
162.99
160.56
154.67
137.91
137.88
129.29
128.97
127.91
126.16
125.19
115.31
115.09
79.54
48.50
47.28
43.60
42.93
40.76
28.30
18,19,20
9,11
8,12
10
7
14
1
6
17
3
5
2
f1 (ppm)

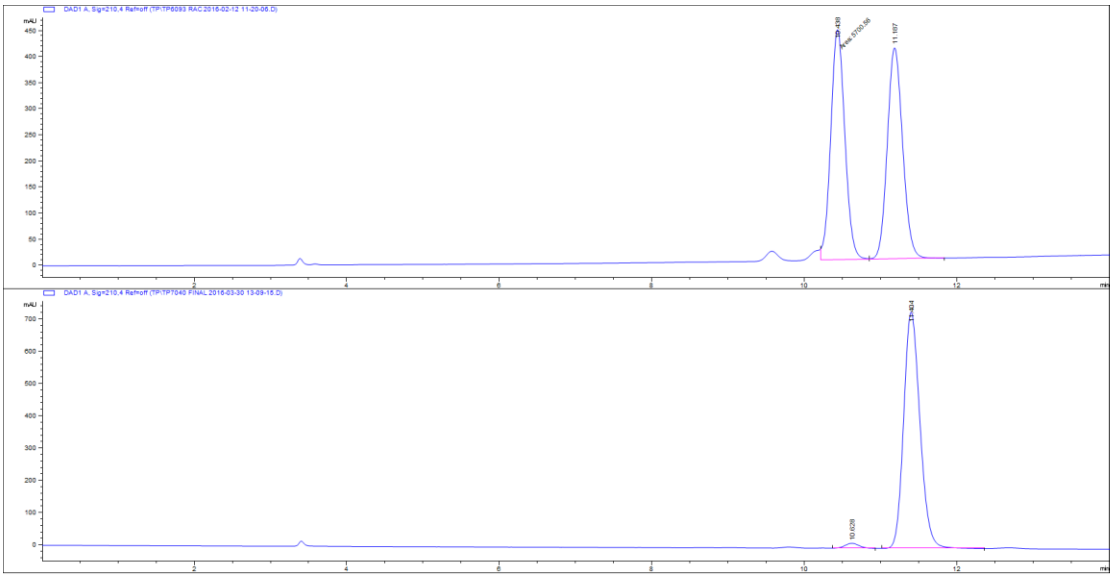

Supplementary figure 65: ^{1}H, ^{13}C-NMR spectra, HPLC traces of compound **62**

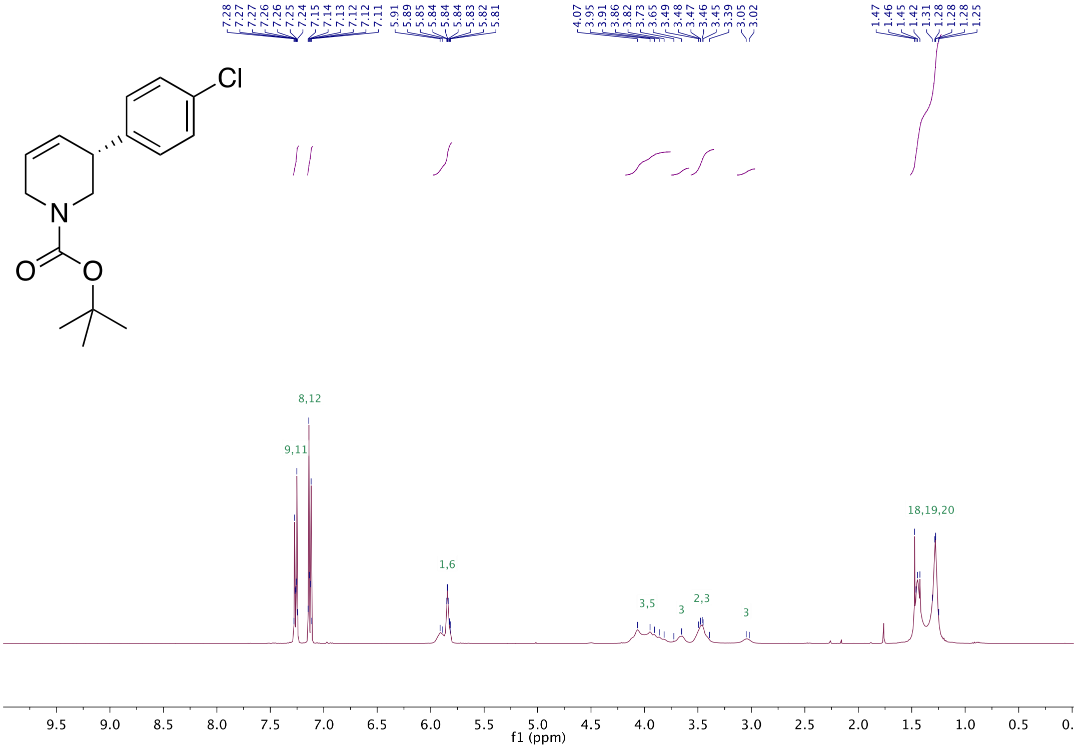

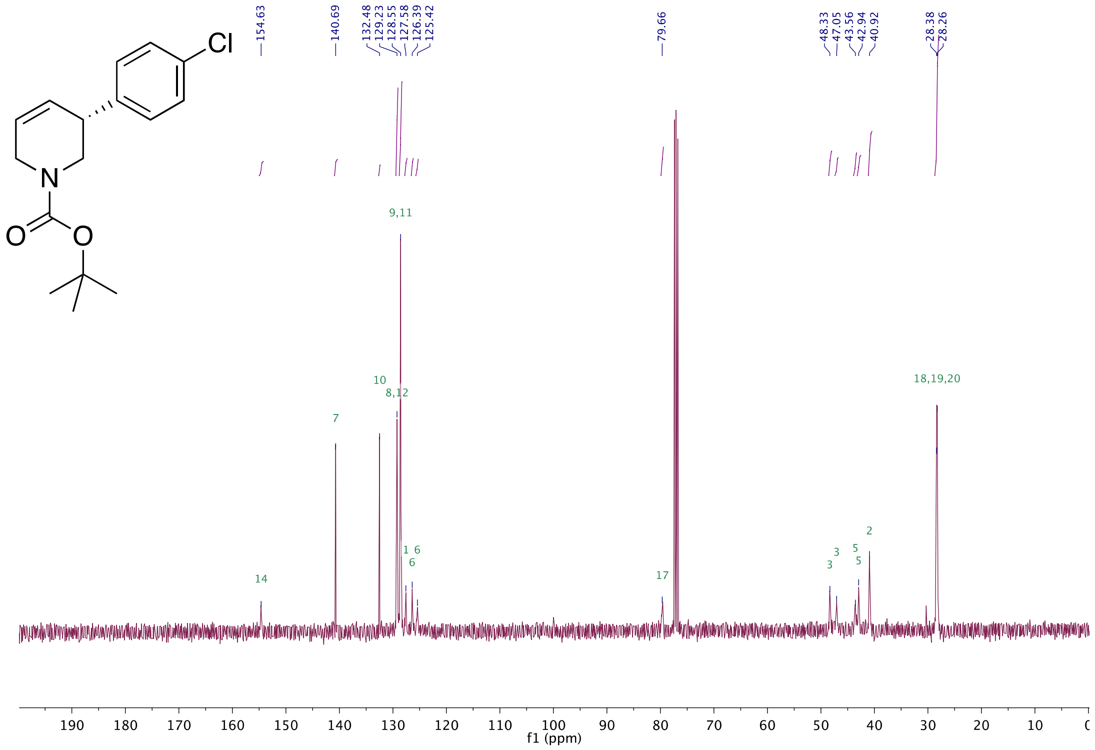

Cl
N
O
O
154.63
140.69
132.48
129.23
128.55
127.58
126.39
125.42
79.66
48.33
47.05
43.56
42.94
40.92
28.38
28.26
9,11
10
8,12
7
18,19,20
14
1 6
6
17
3
3
5
5
2
190 180 170 160 150 140 130 120 110 100 90 80 70 60 50 40 30 20 10 0
f1 (ppm)

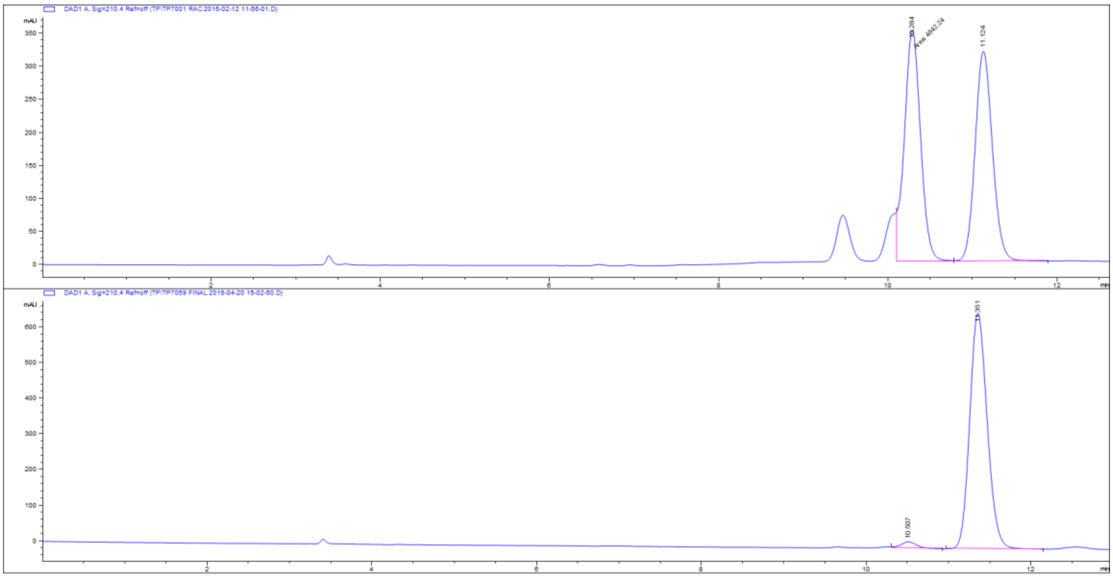

10.284
11.124
10.507
11.351

Supplementary figure 66: ^{1}H, ^{13}C-NMR spectra, HPLC traces of compound **63**

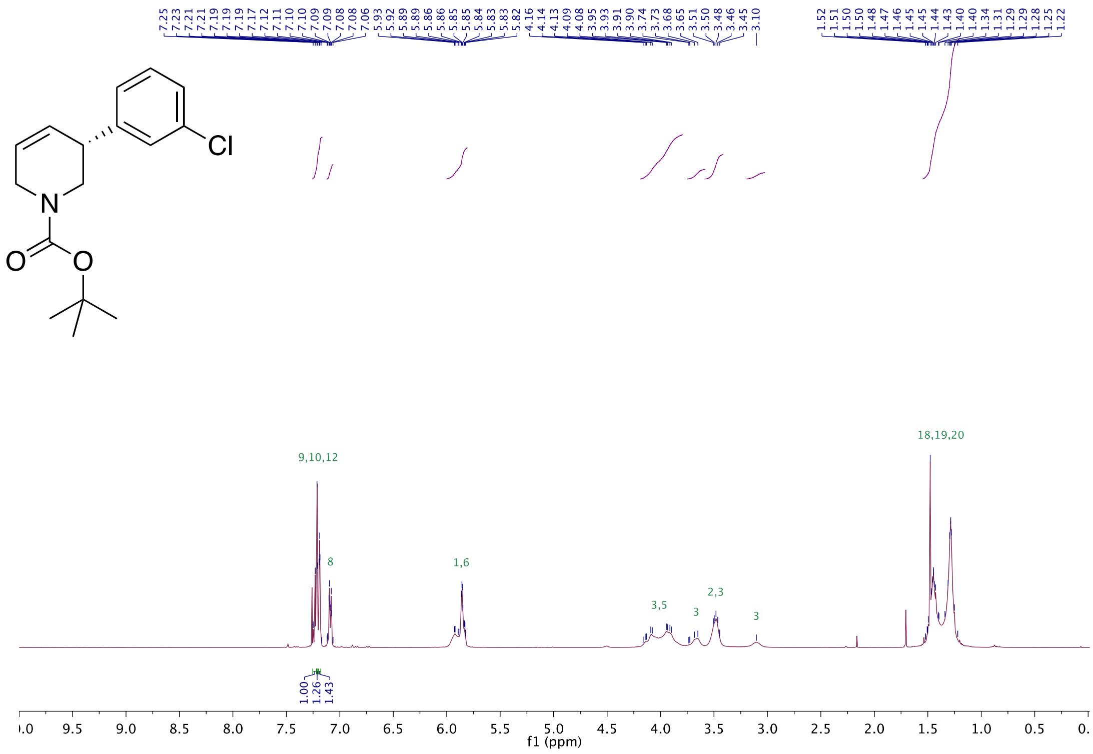

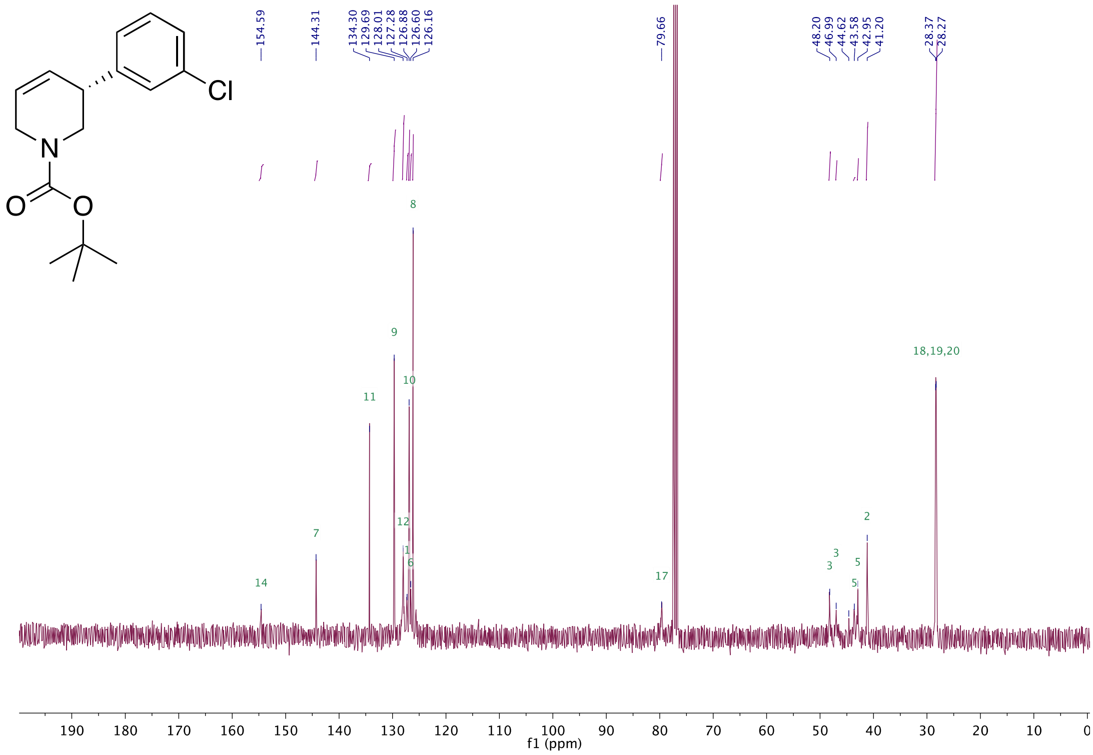

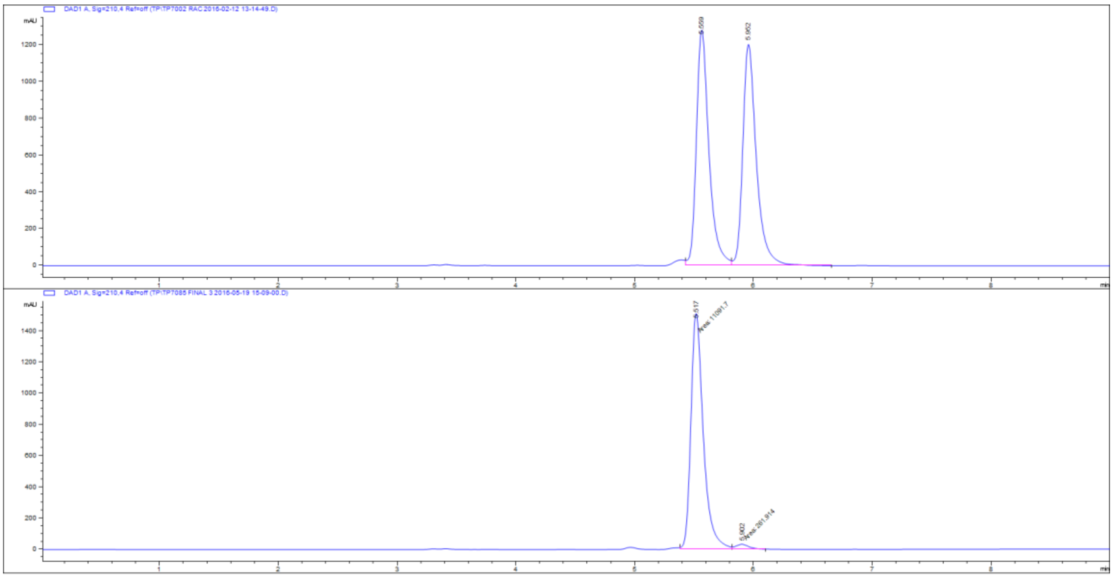

Supplementary figure 67: ^{1}H, ^{13}C-NMR spectra, HPLC traces of compound **64**

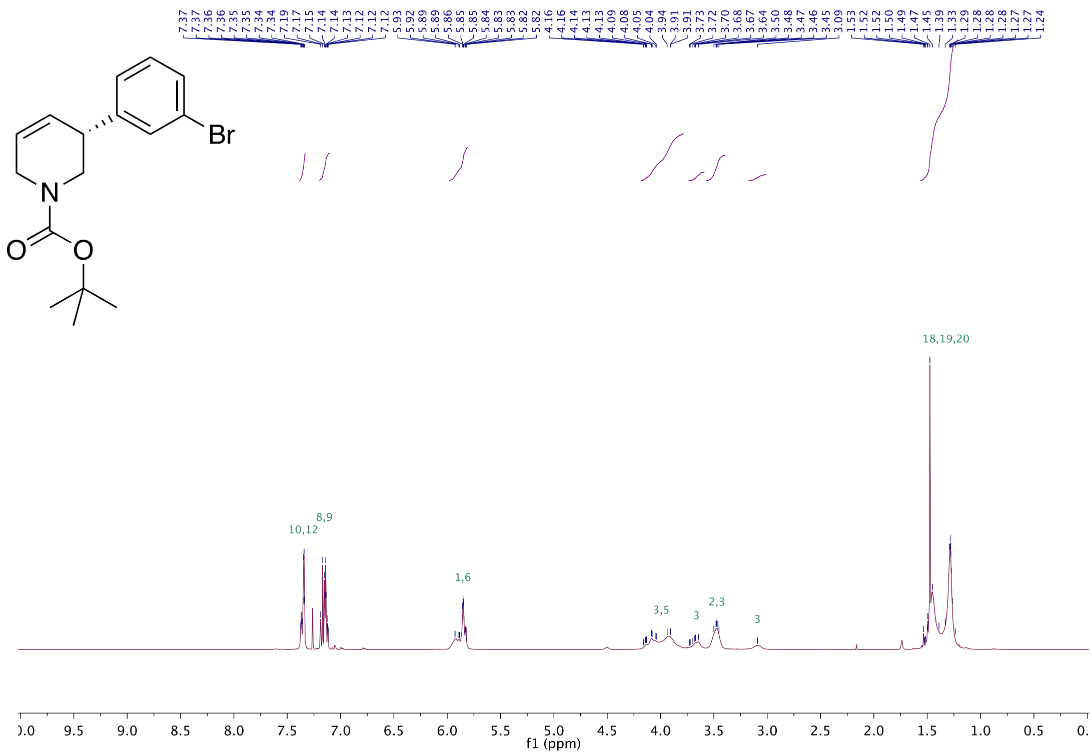

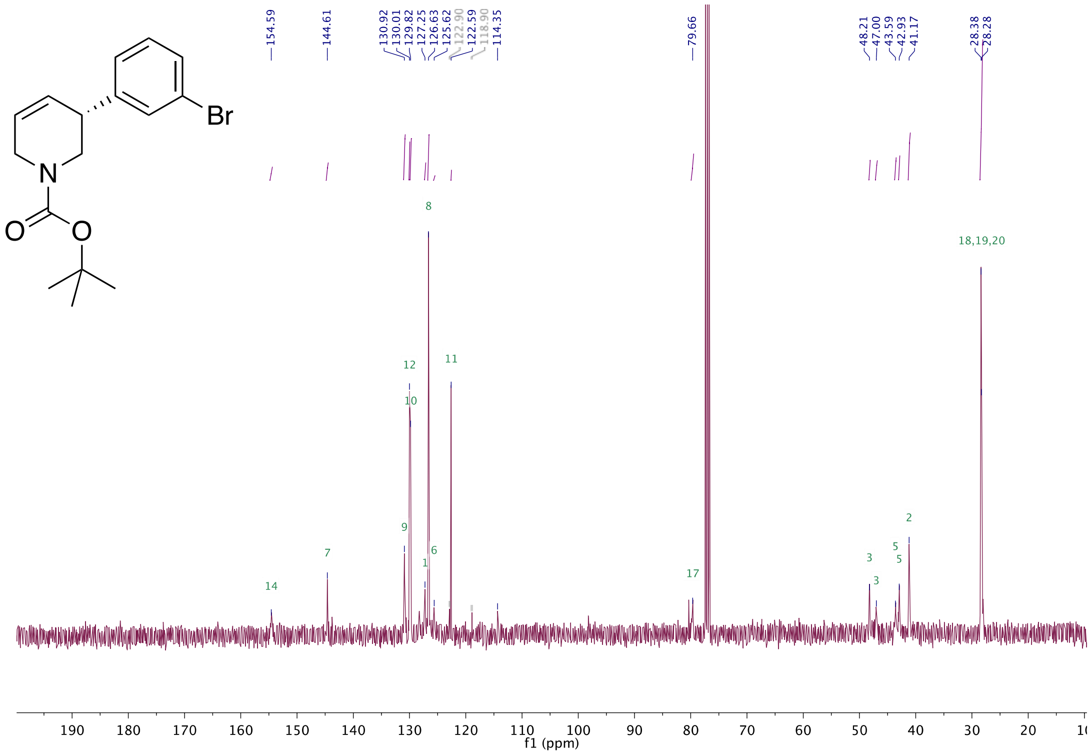
Br
N
O
O
154.59
144.61
130.92
130.01
129.82
127.25
126.63
125.62
122.90
122.59
118.90
114.35
79.66
48.21
47.00
43.59
42.93
41.17
28.38
28.28
14
7
9
12
10
8
11
6
17
3
3
5
5
2
18,19,20
190 180 170 160 150 140 130 120 110 100 90 80 70 60 50 40 30 20
f1 (ppm)

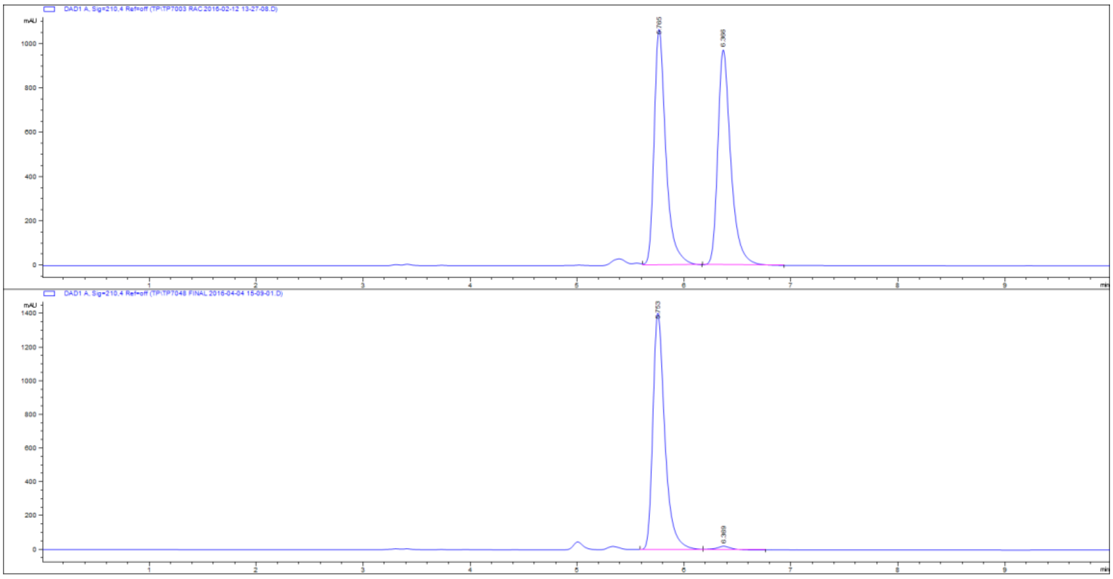
mAU
5.765
6.366
mAU
5.753
6.369

Supplementary figure 68: ^{1}H, ^{13}C-NMR spectra, HPLC traces of compound **65**

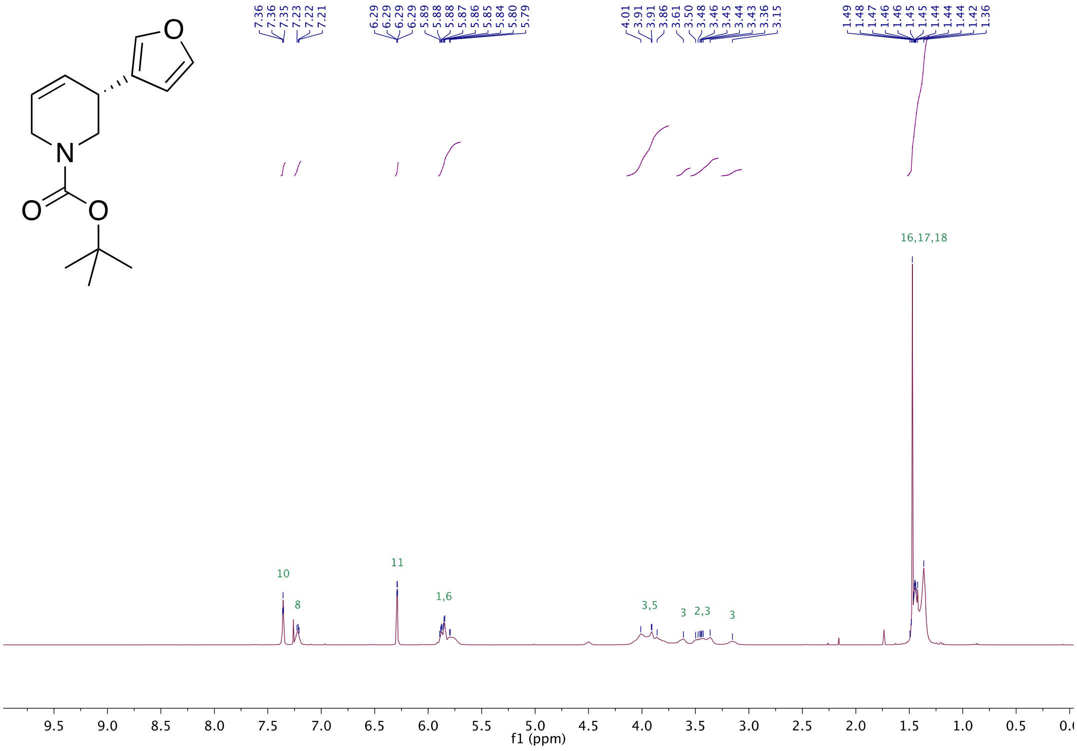

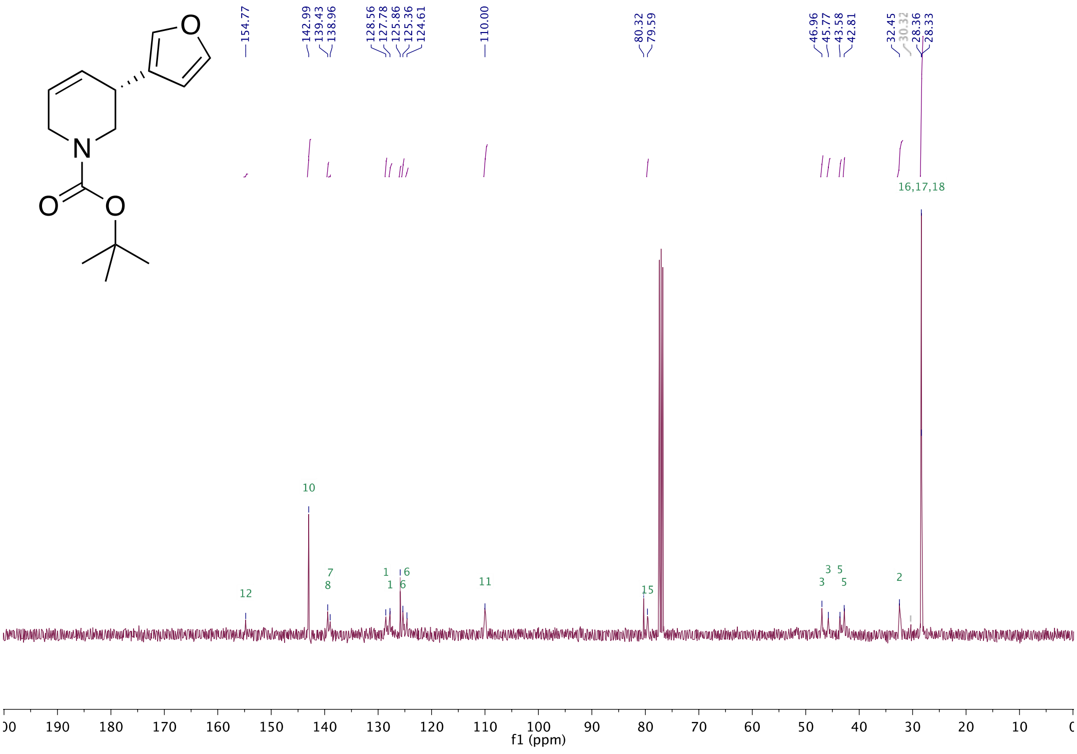

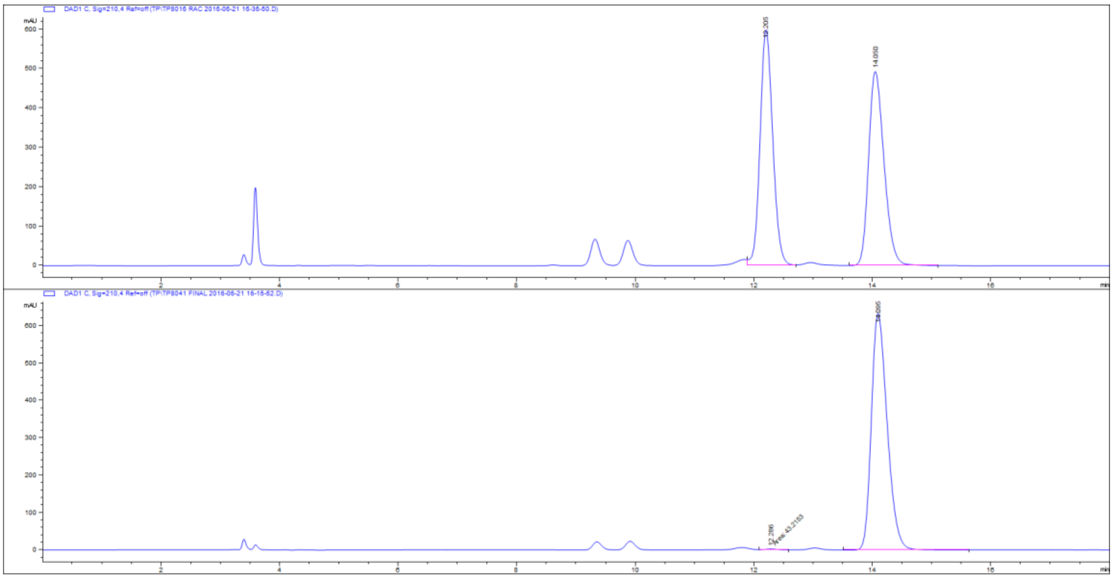

Supplementary figure 69: ^{1}H, ^{13}C-NMR spectra, HPLC traces of compound **66**

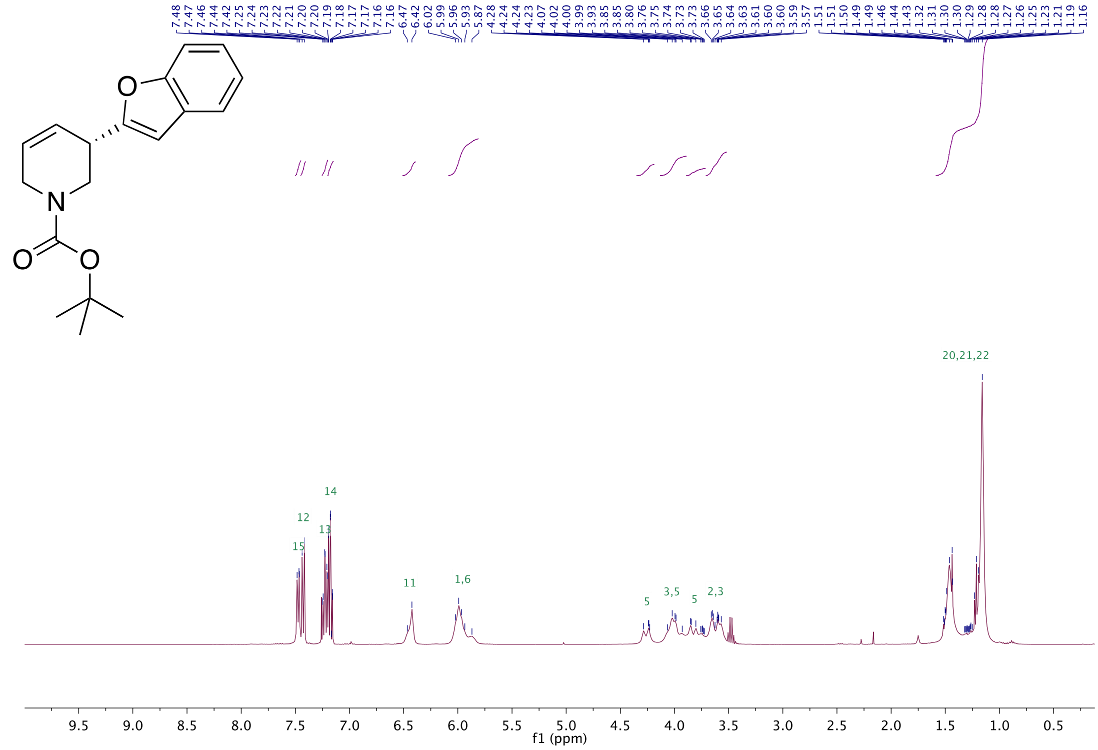

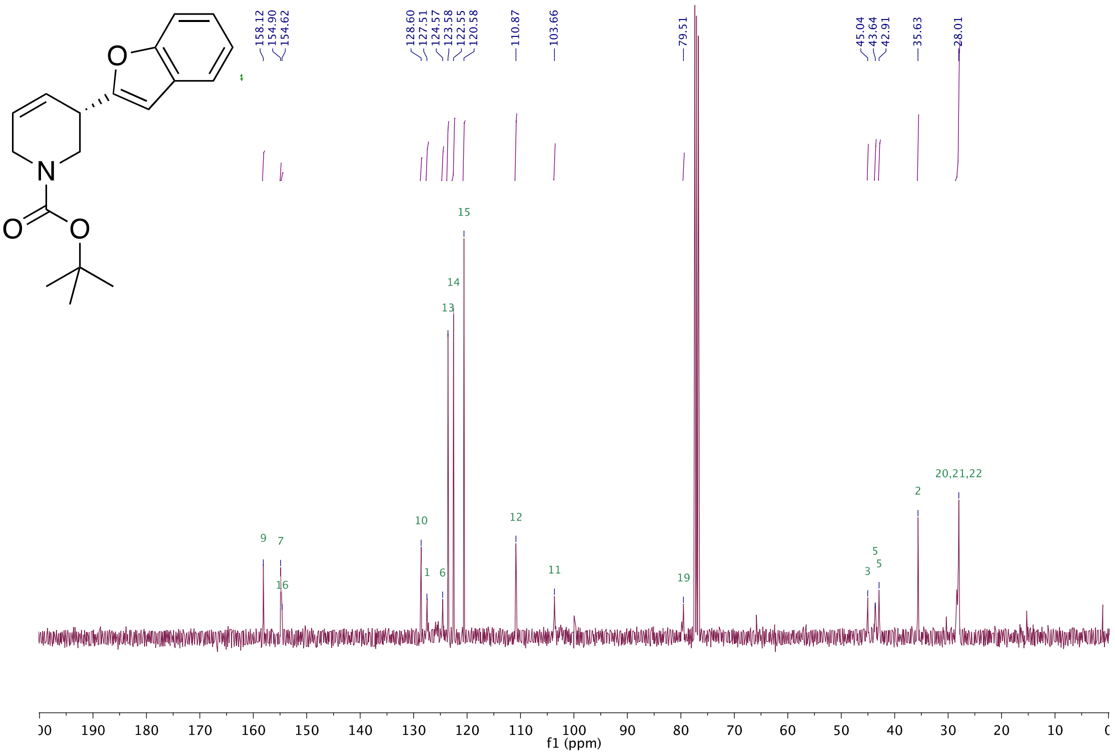
O
N
O
O
158.12
154.90
154.62
128.60
127.51
124.57
123.58
122.55
120.58
110.87
103.66
79.51
45.04
43.64
42.91
35.63
28.01
9
7
16
10
1
6
13
14
15
12
11
19
3
5
5
2
20,21,22
f1 (ppm)

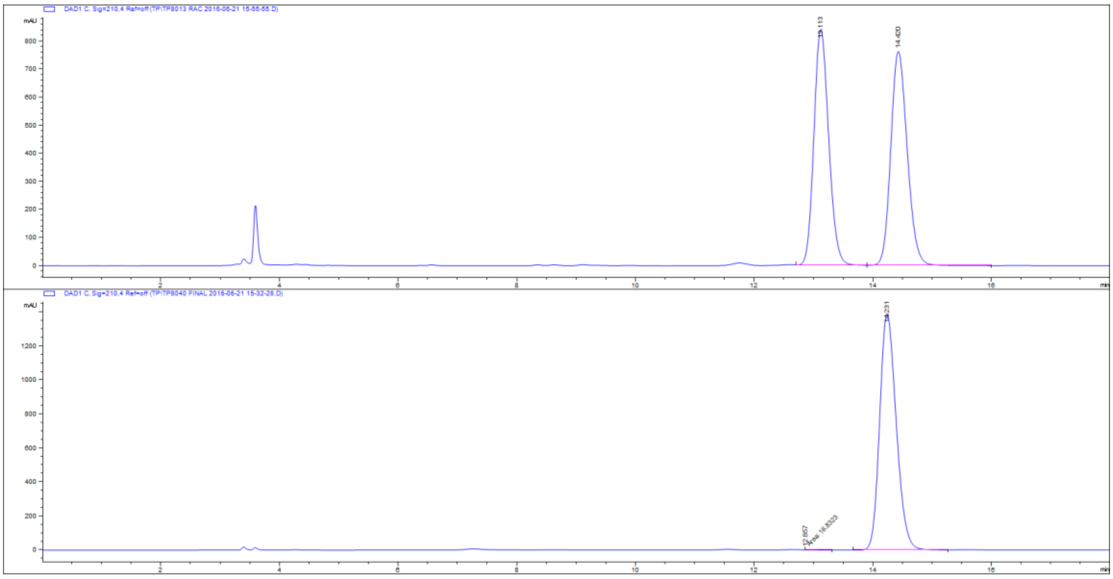

Supplementary figure 70: ^{1}H, ^{13}C-NMR spectra, HPLC traces of compound **67**

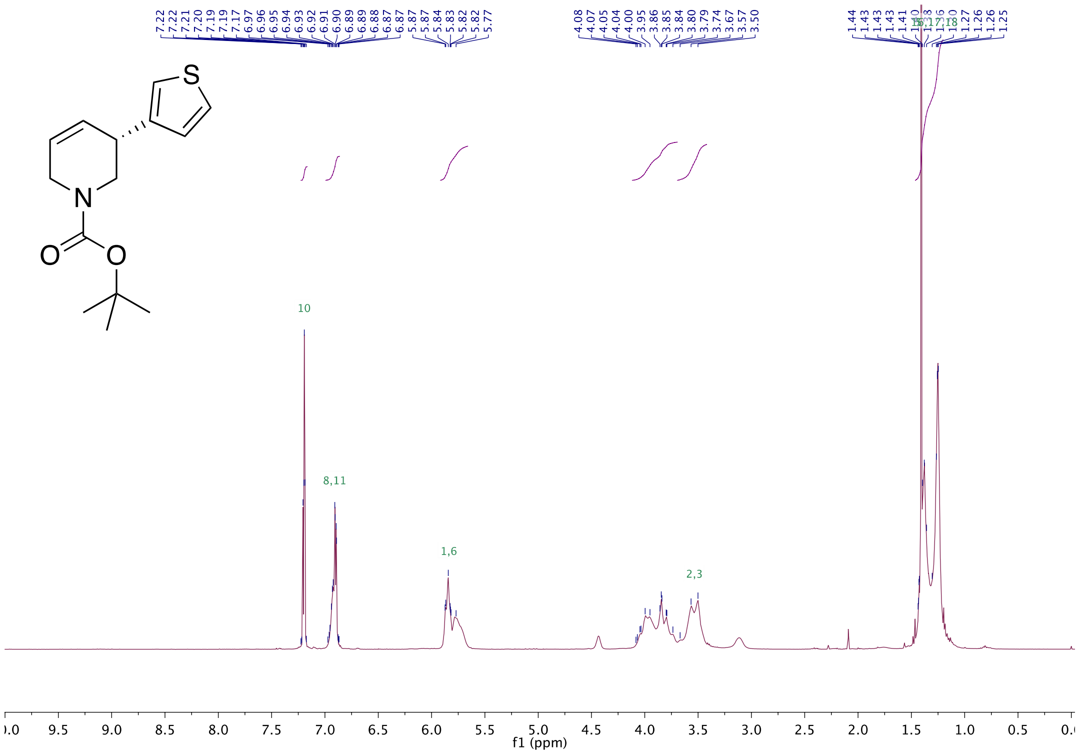

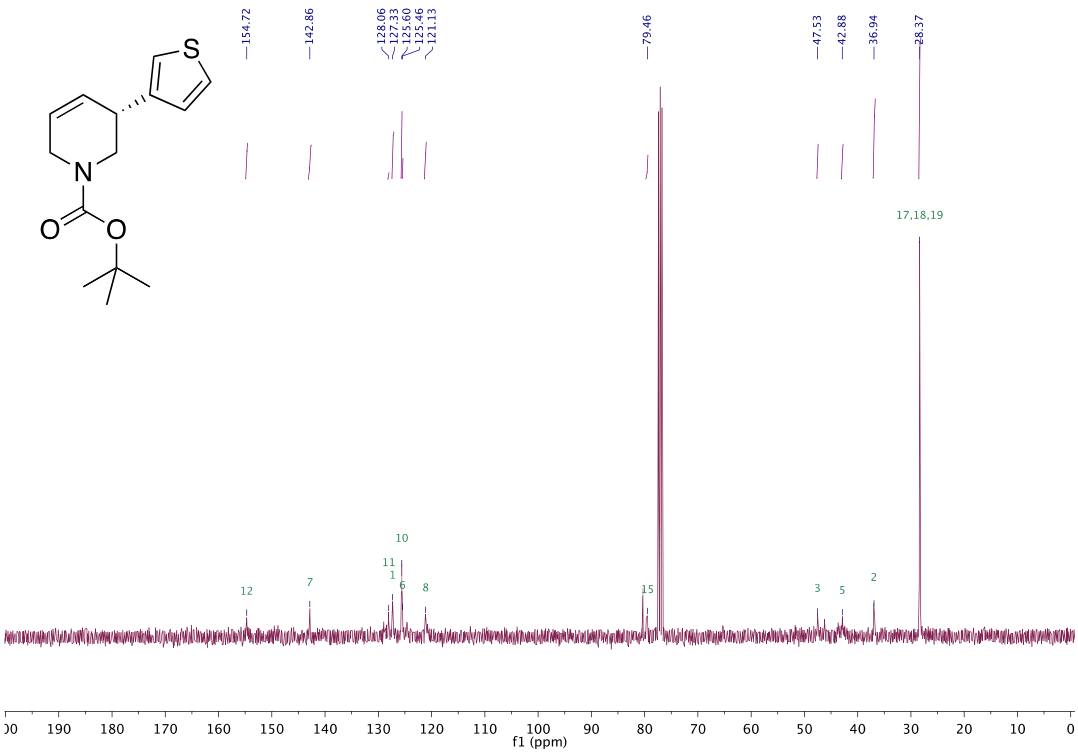

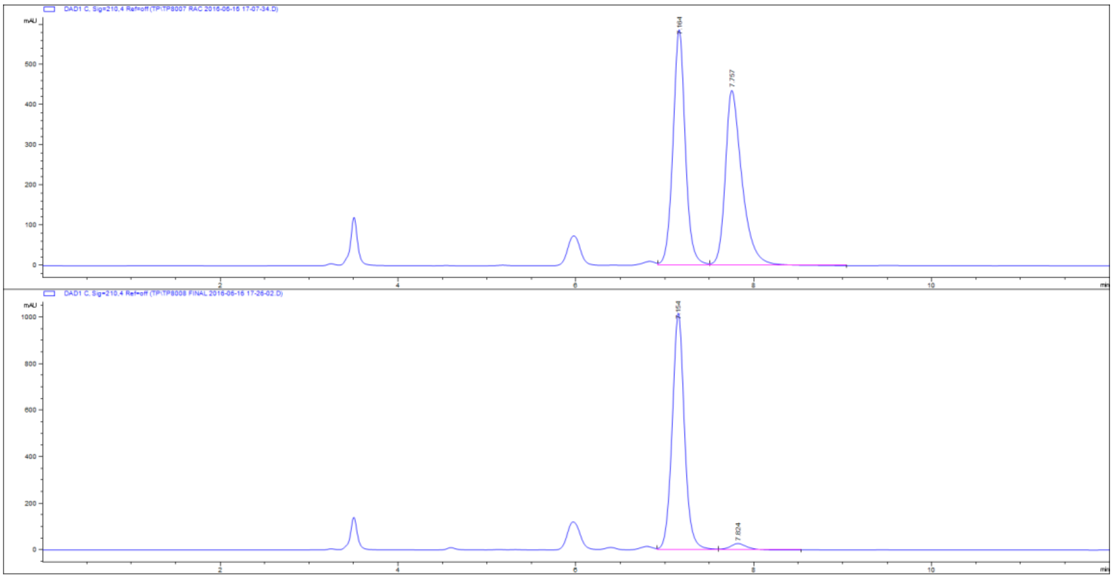

Supplementary figure 71: ^{1}H, ^{13}C-NMR spectra, HPLC traces of compound **68**

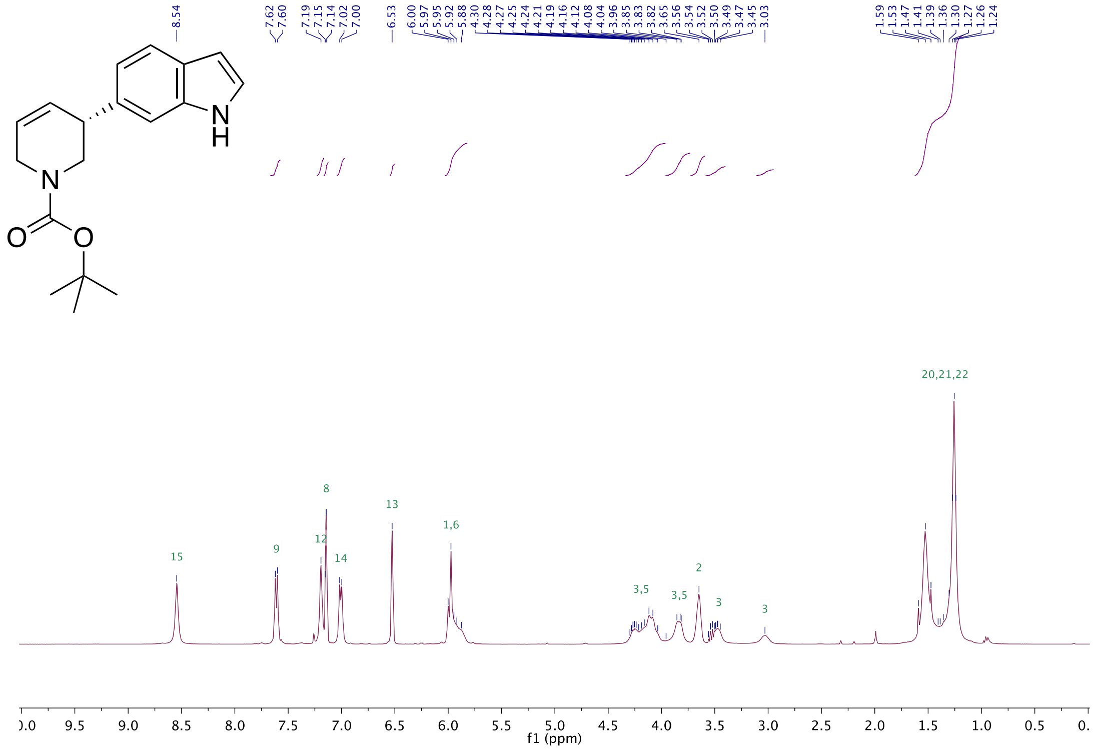

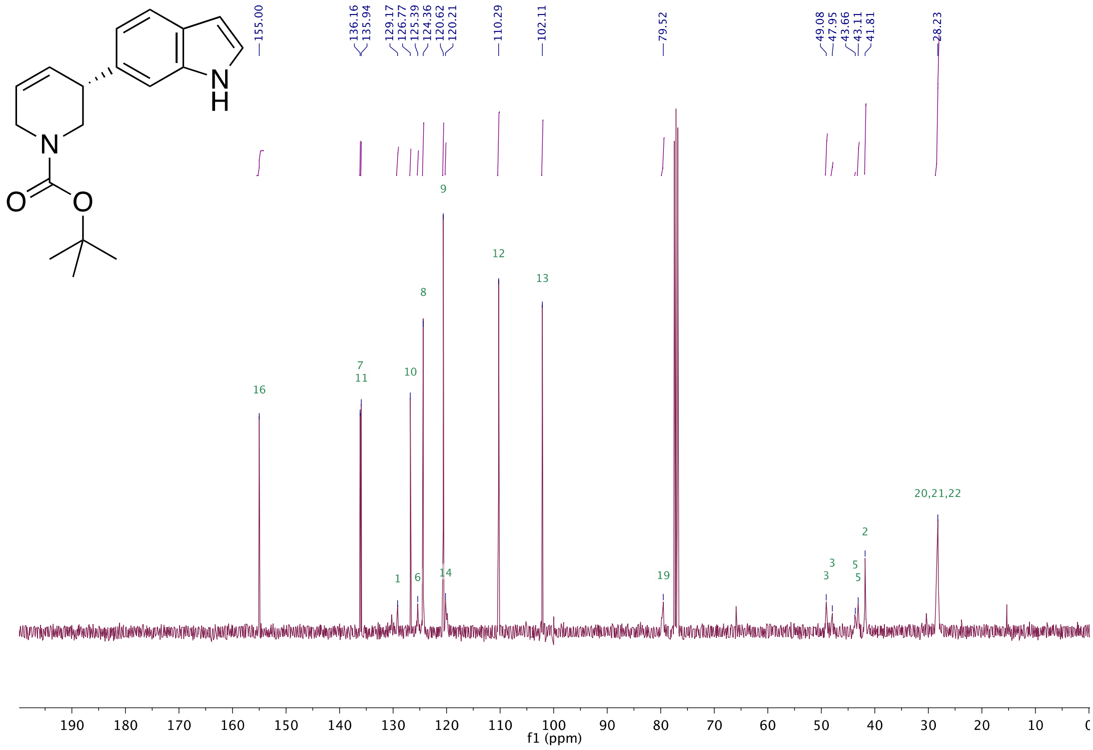

N
O
O
NH
155.00
136.16
135.94
129.17
126.77
125.39
124.36
120.62
120.21
110.29
102.11
79.52
49.08
47.95
43.66
43.11
41.81
28.23
16
7
11
10
1
6
8
9
14
12
13
19
3
3
5
5
2
20,21,22
190
180
170
160
150
140
130
120
110
100
90
80
70
60
50
40
30
20
10
f1 (ppm)

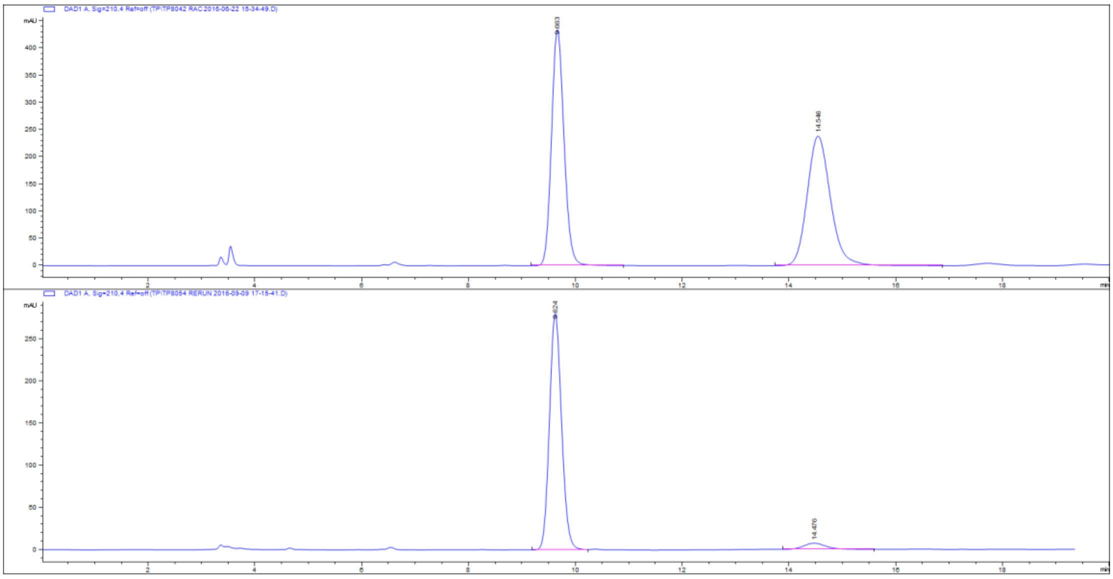

Supplementary figure 72: ^{1}H, ^{13}C-NMR spectra, HPLC traces of compound **69**

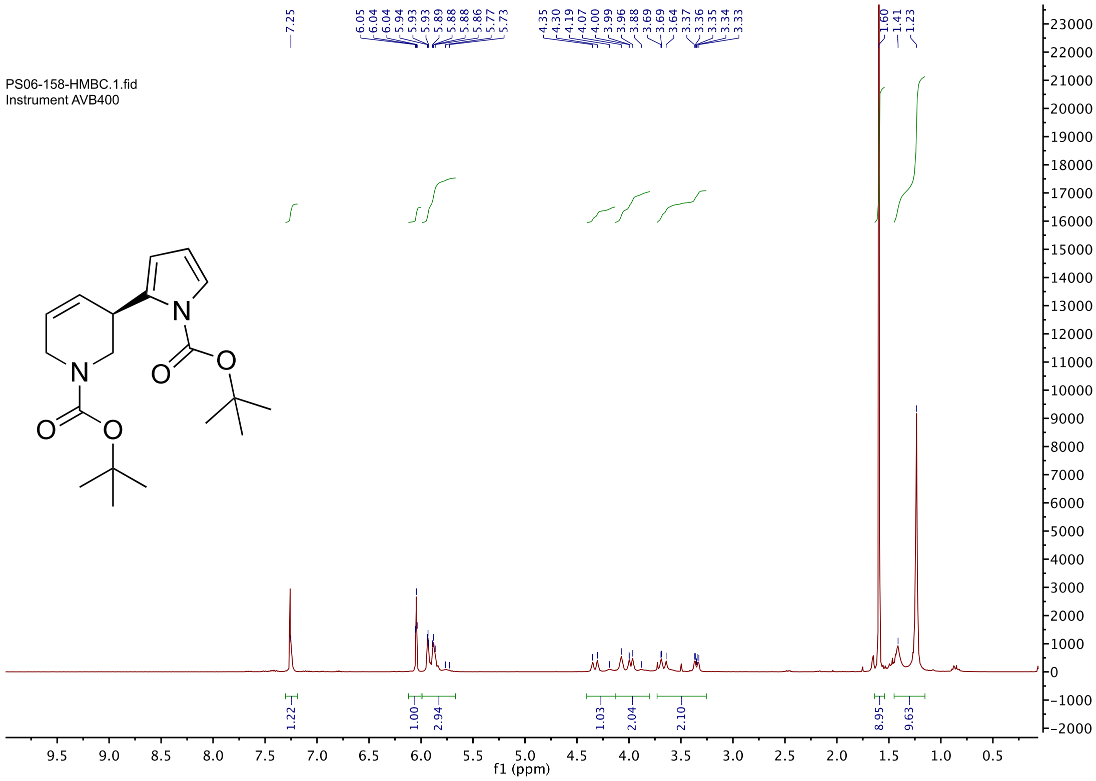

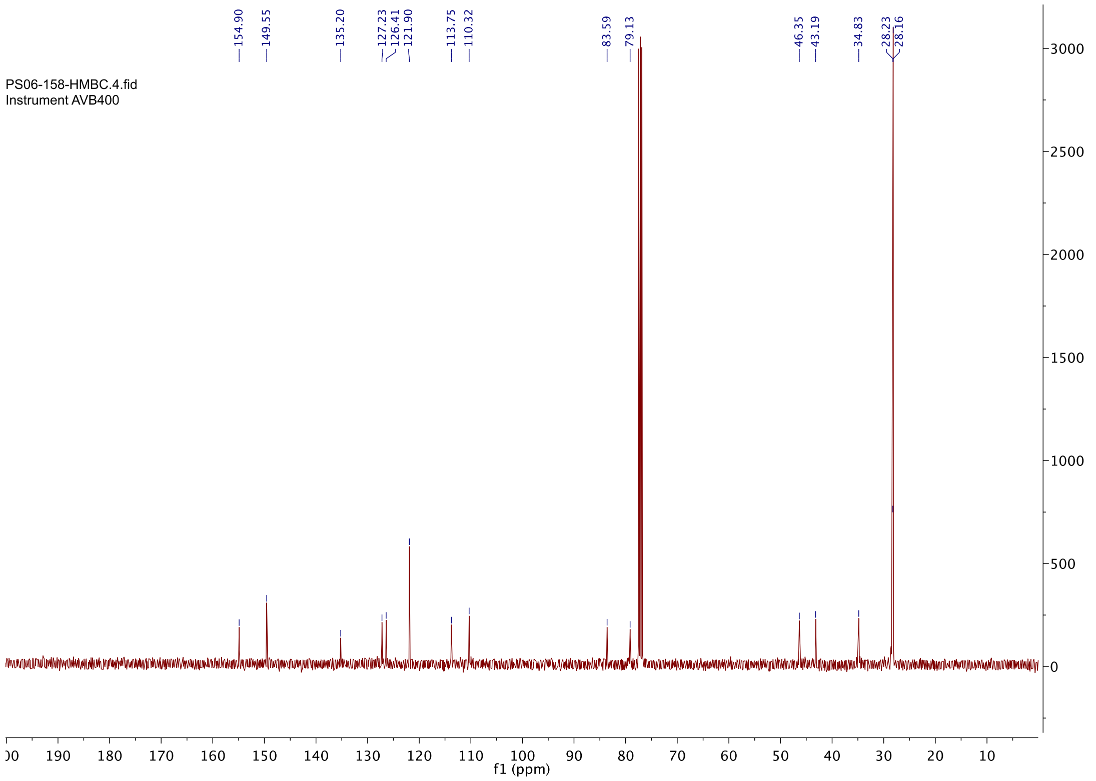

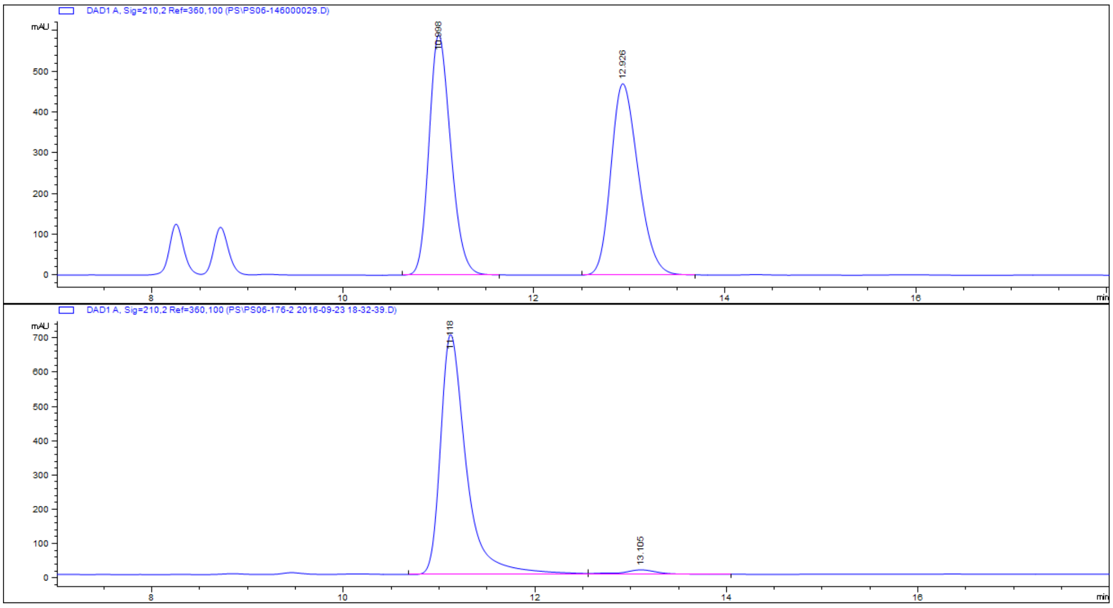

Supplementary figure 73: ^{1}H, ^{13}C-NMR spectra, HPLC traces of compound **70**

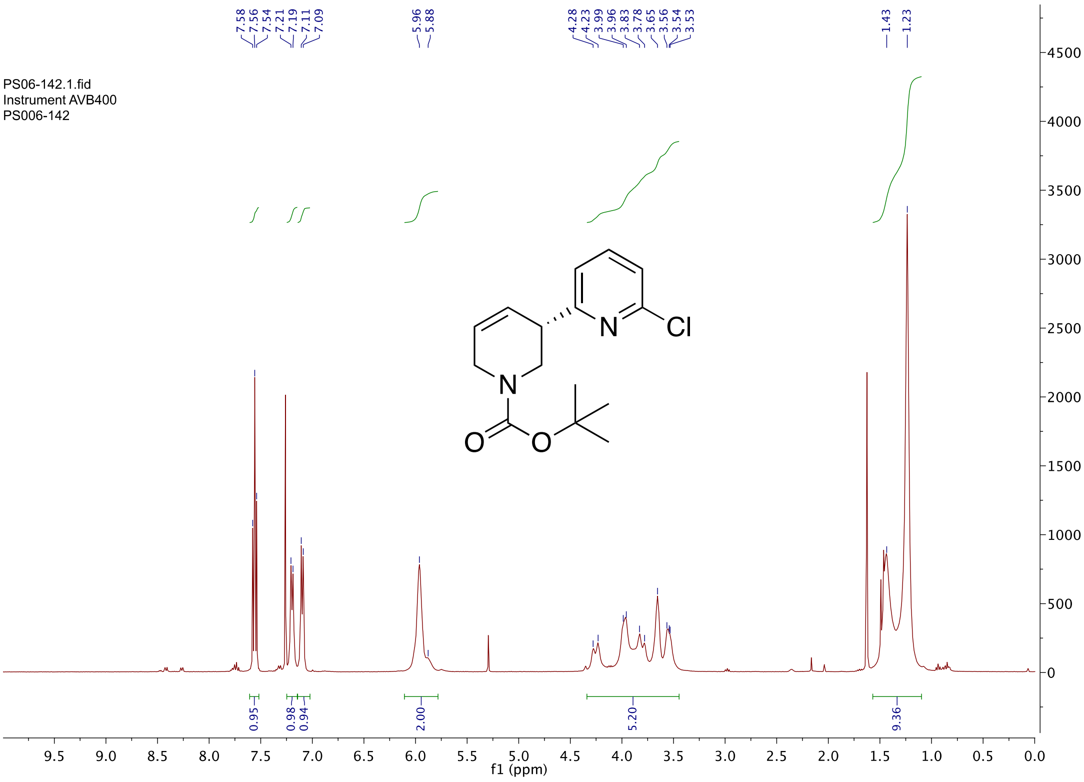

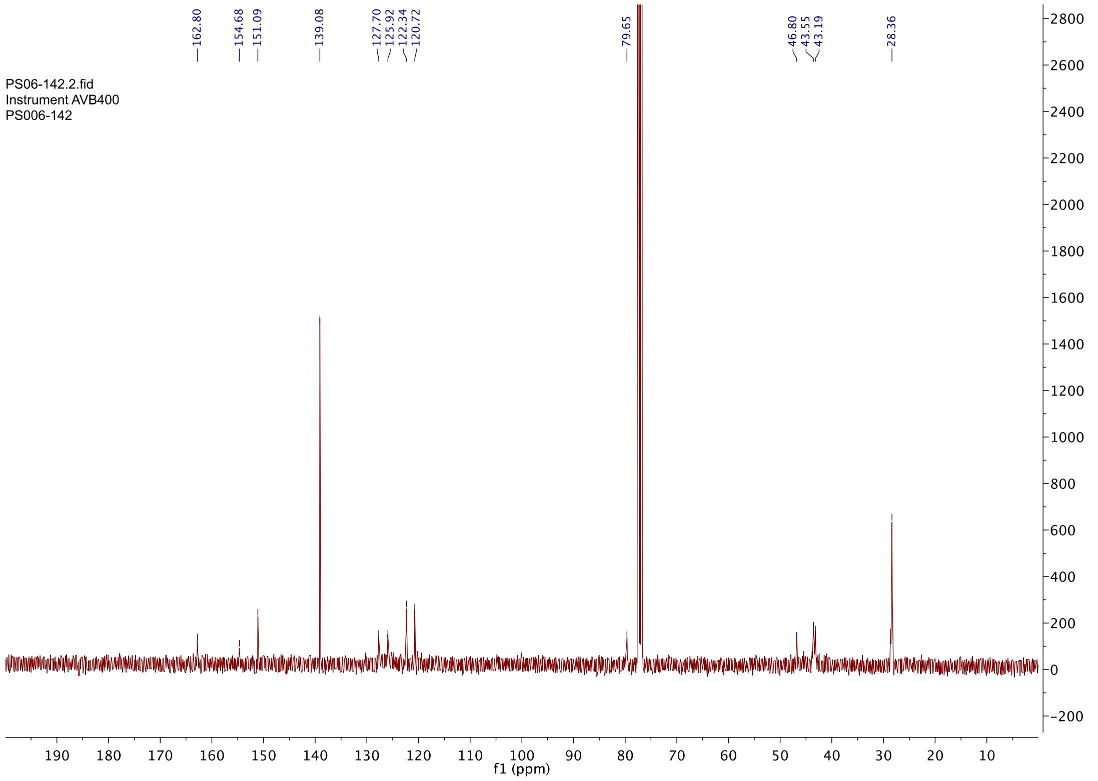
PS06-142.2.fid
Instrument AVB400
PS006-142
162.80
154.68
151.09
139.08
127.70
125.92
122.34
120.72
79.65
46.80
43.55
43.19
28.36
f1 (ppm)

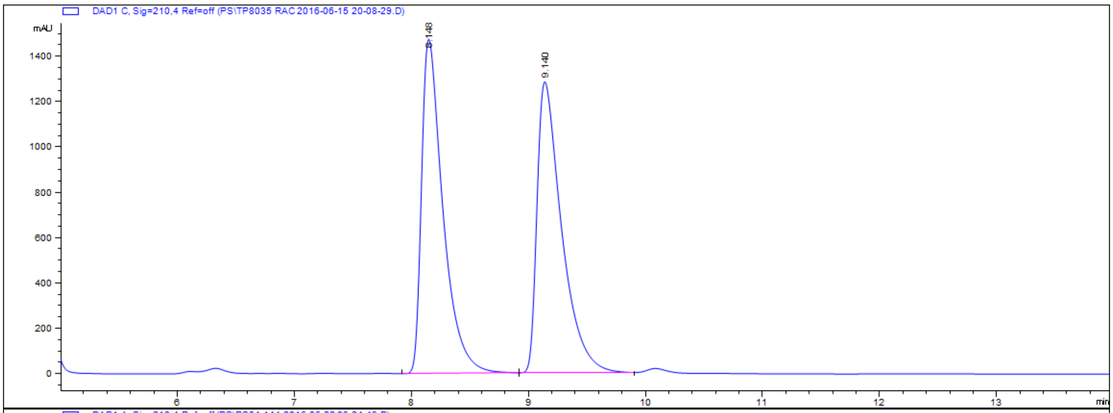
DAD1 C, Sig=210,4 Ref=off (PS\TP8035 RAC 2016-06-15 20-08-29.D)
mAU
8.148
9.140
min

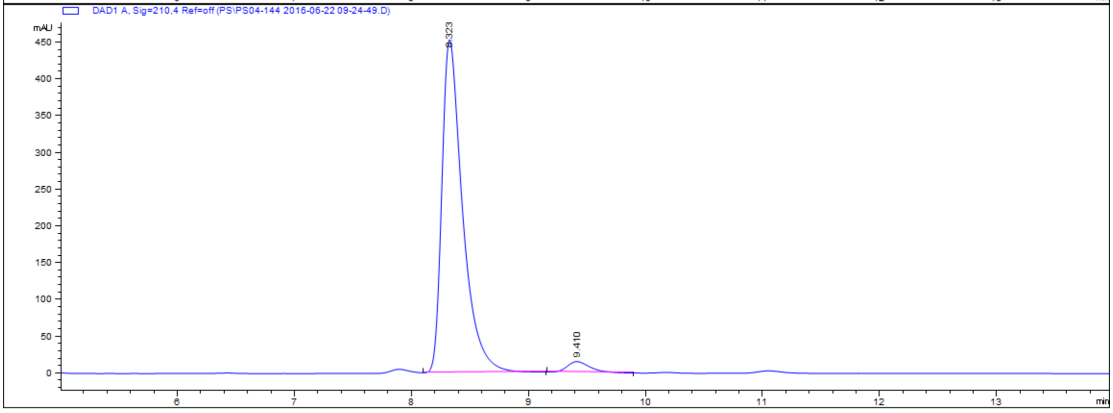
DAD1 A, Sig=210,4 Ref=off (PS\PS04-144 2016-06-22 09-24-49.D)
mAU
8.323
9.410
min

Supplementary figure 74: ^{1}H, ^{13}C-NMR spectra, HPLC traces of compound **71**

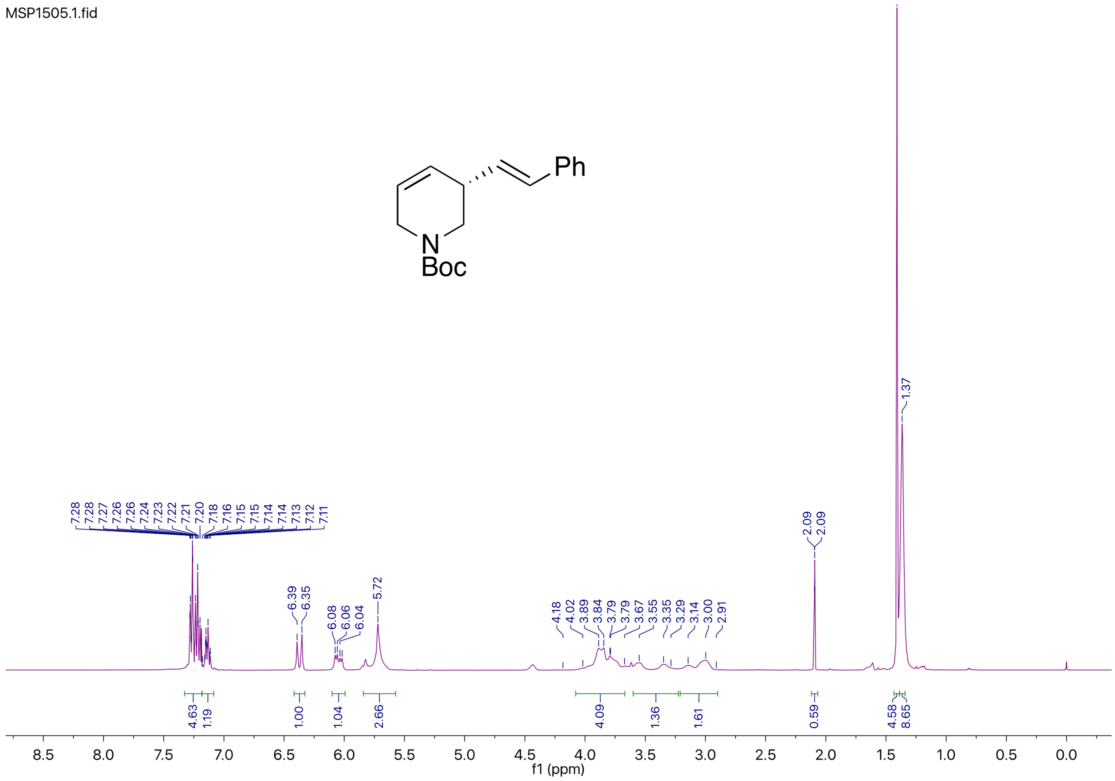

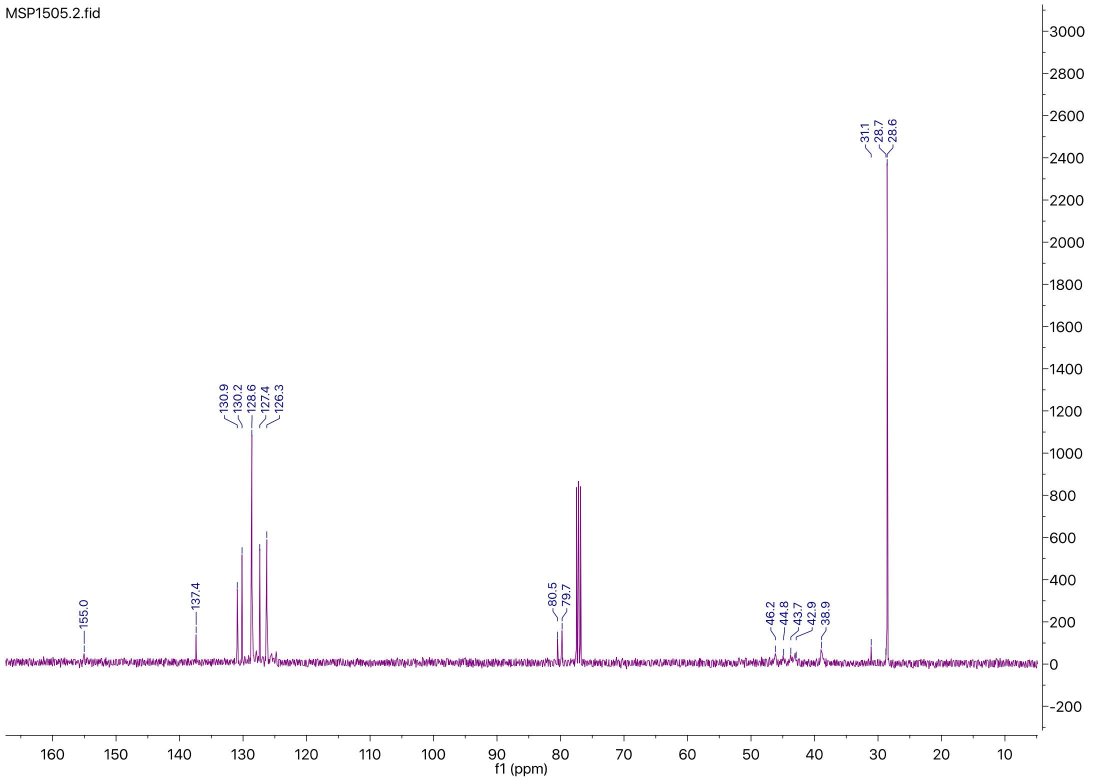

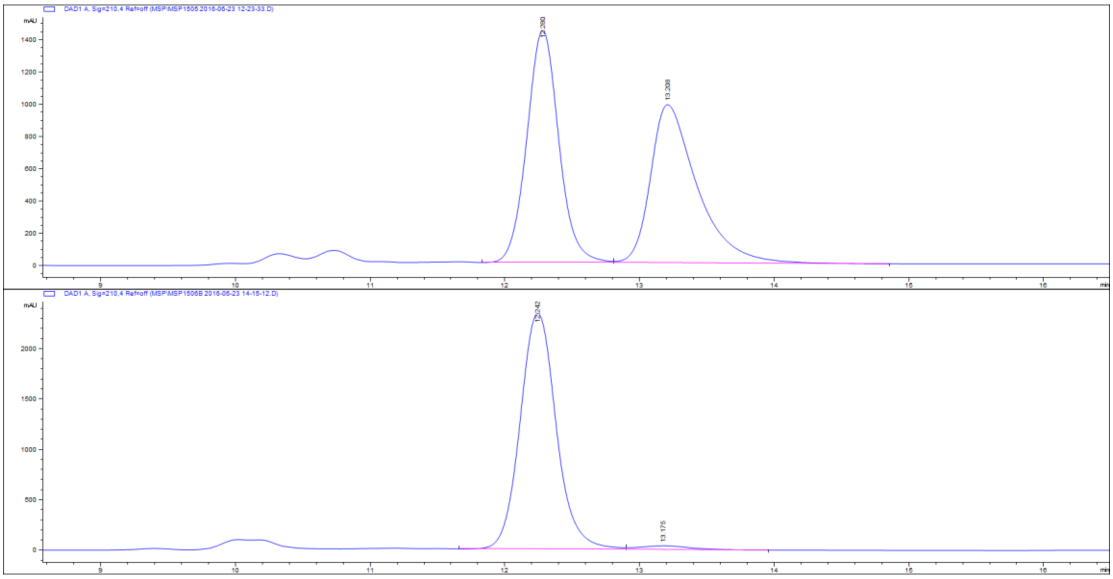

Supplementary figure 75: ^{1}H, ^{13}C-NMR spectra, HPLC traces of compound **72**

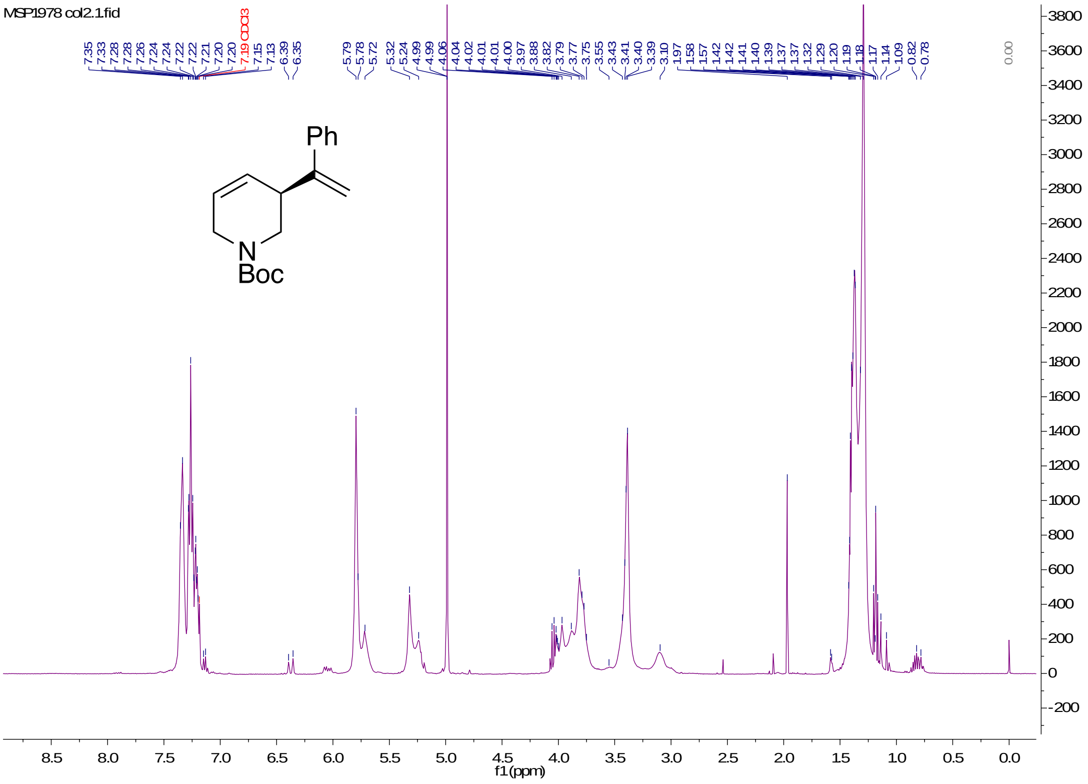

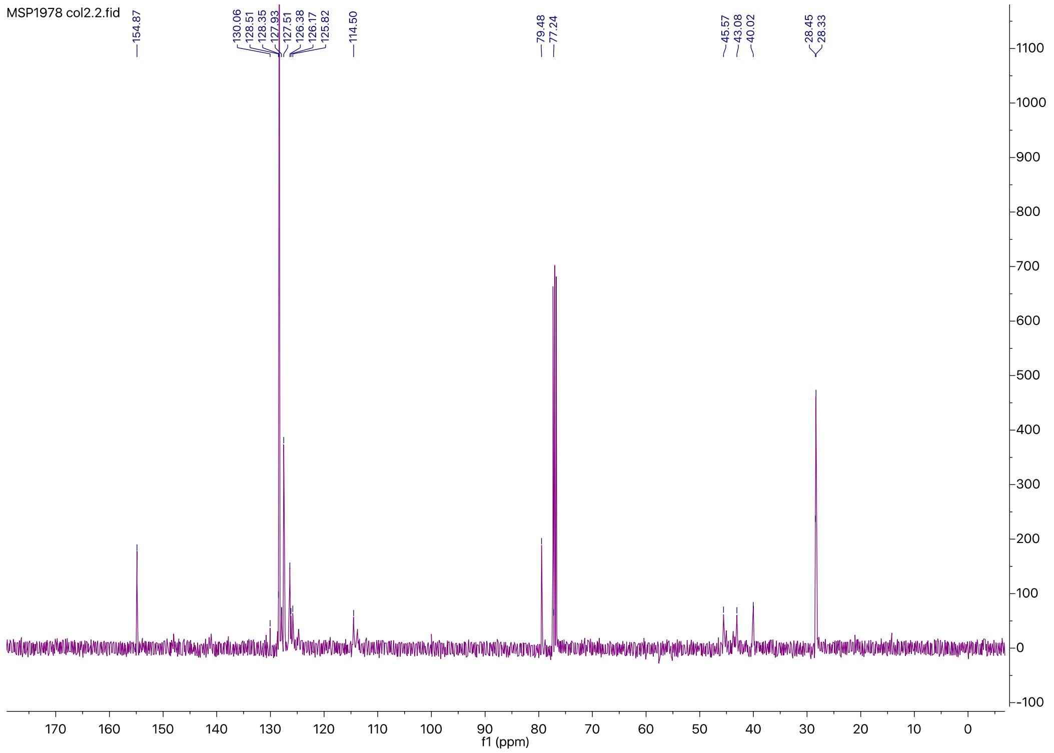
MSP1978 col2.2.fid
154.87
130.06
128.51
128.35
127.93
127.51
126.38
126.17
125.82
114.50
79.48
77.24
45.57
43.08
40.02
28.45
28.33
f1 (ppm)

HPLC traces:

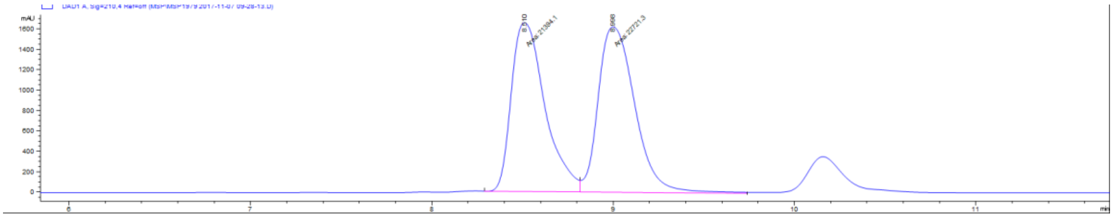

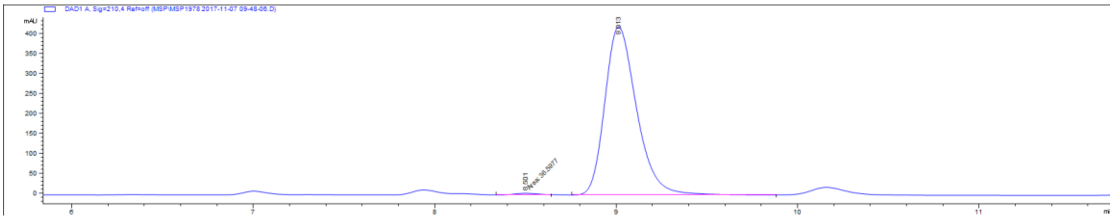

Supplementary figure 75: ^{1}H, ^{13}C-NMR spectra, HPLC traces of compound **72**

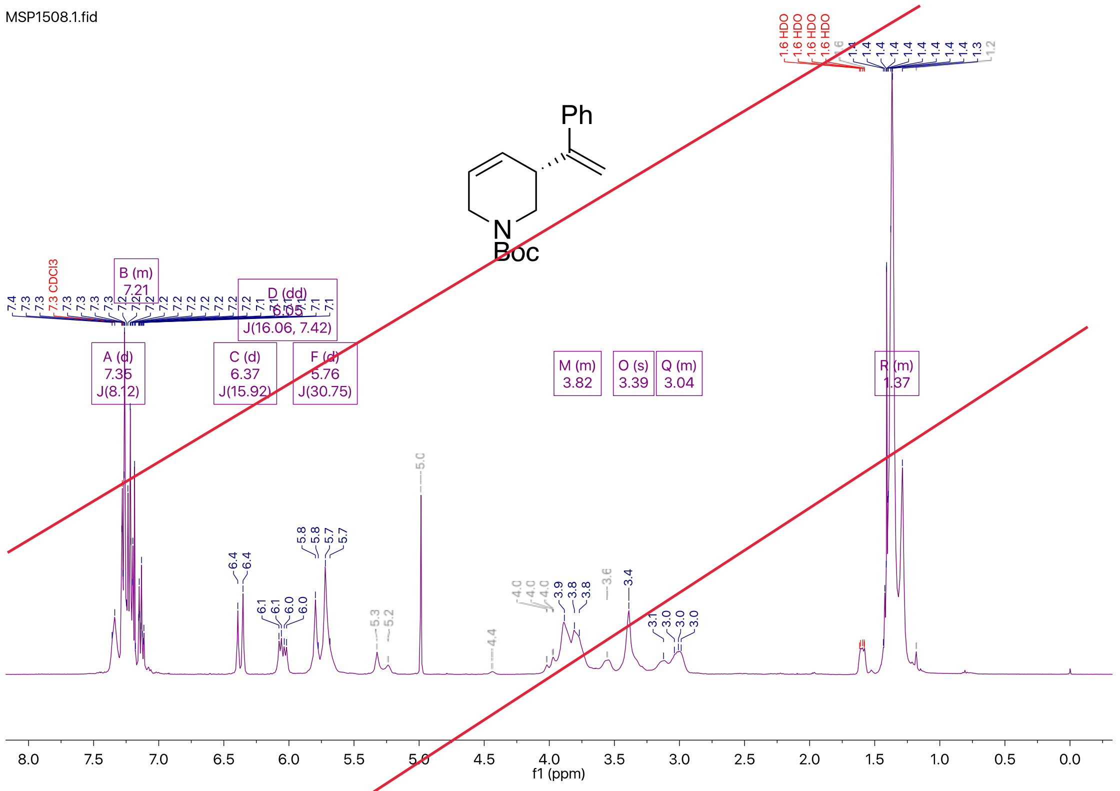

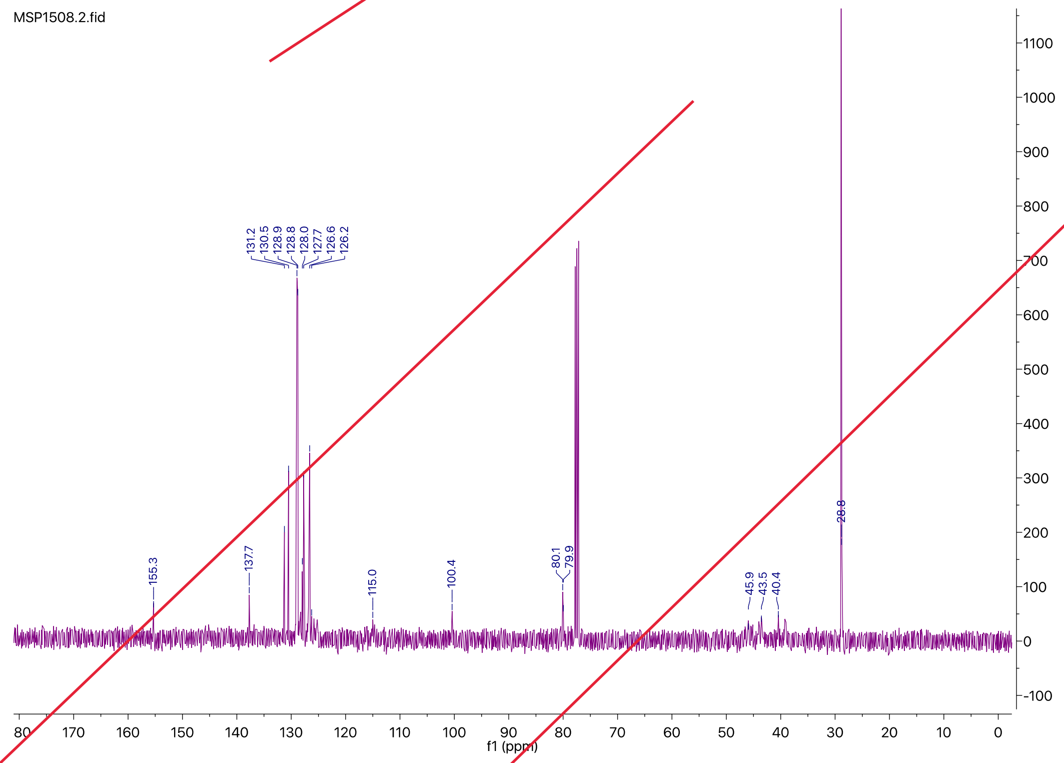

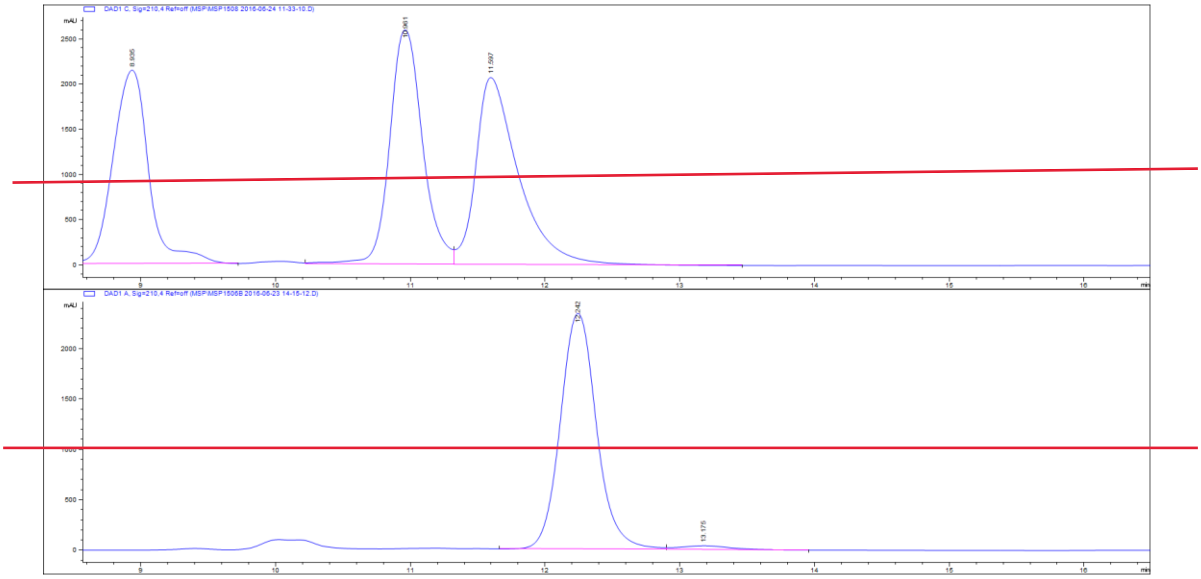

DAD1 C, Sig=210,4 Ref=off (MSP\MSP1508 2016-06-24 11-33-10.D)
mAU
8.935
10.961
11.597
min
DAD1 A, Sig=210,4 Ref=off (MSP\MSP1506B 2016-06-23 14-15-12.D)
mAU
12.242
13.175
min

Supplementary figure 76: ^{1}H, ^{13}C-NMR spectra, HPLC traces of compound **73**

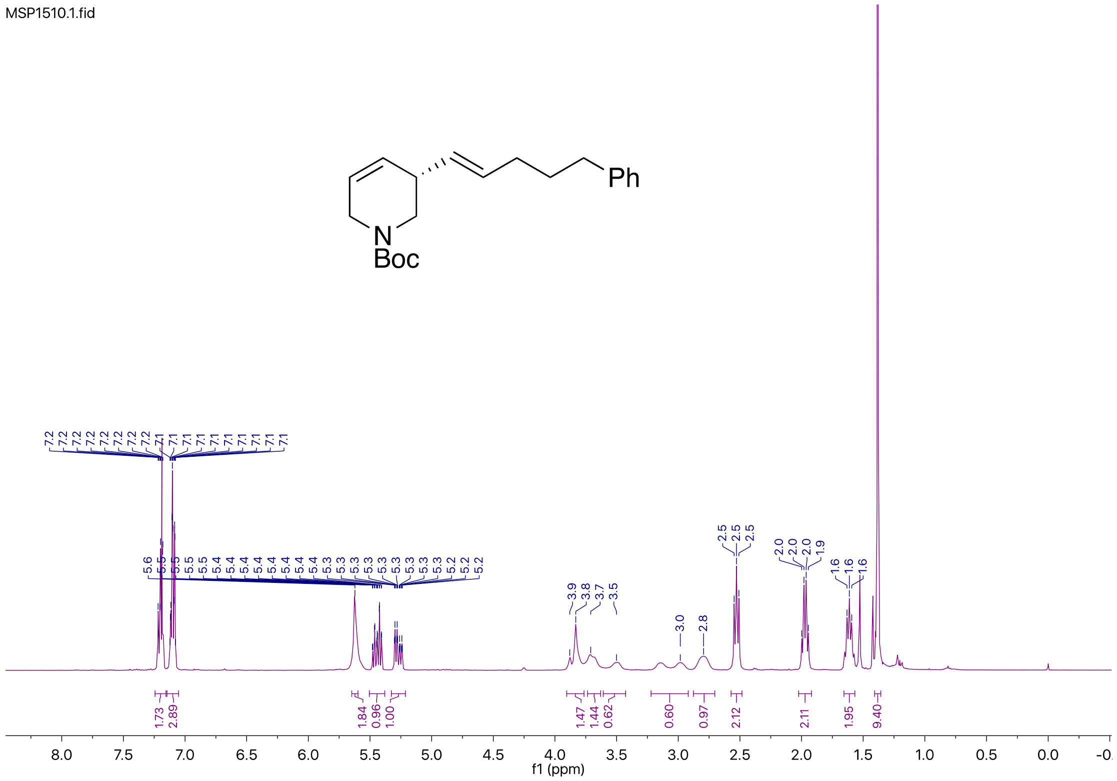

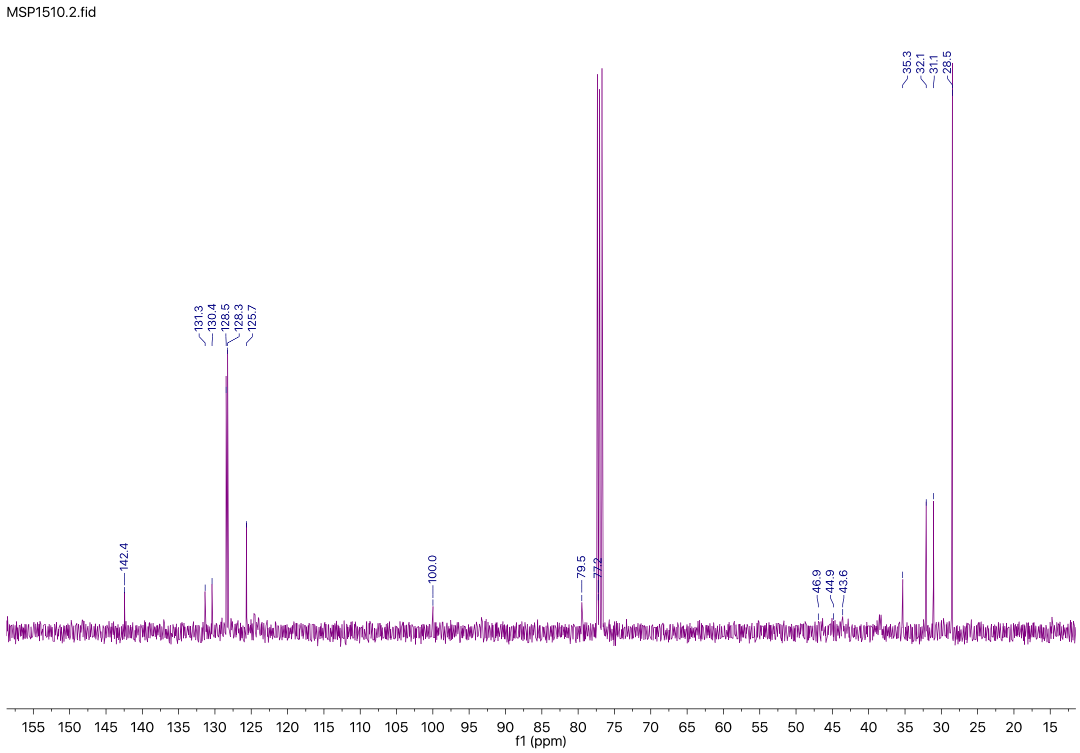

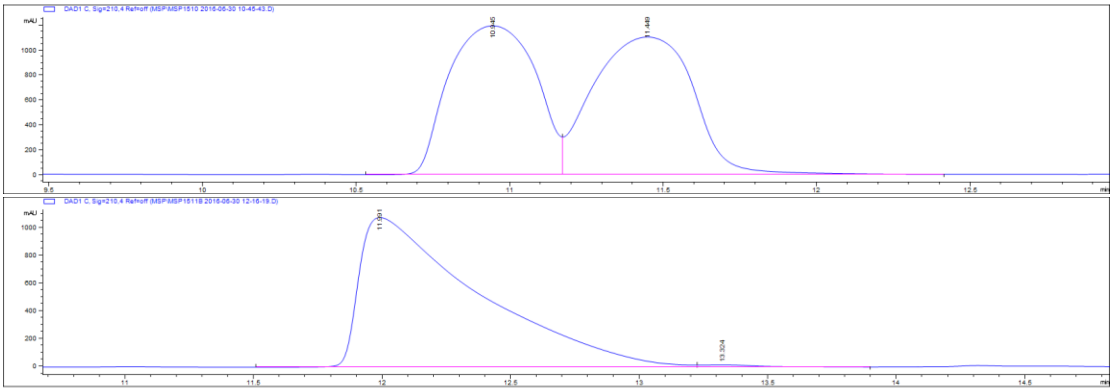

Supplementary figure 77: ^{1}H, ^{13}C-NMR spectra, HPLC traces of compound **74**

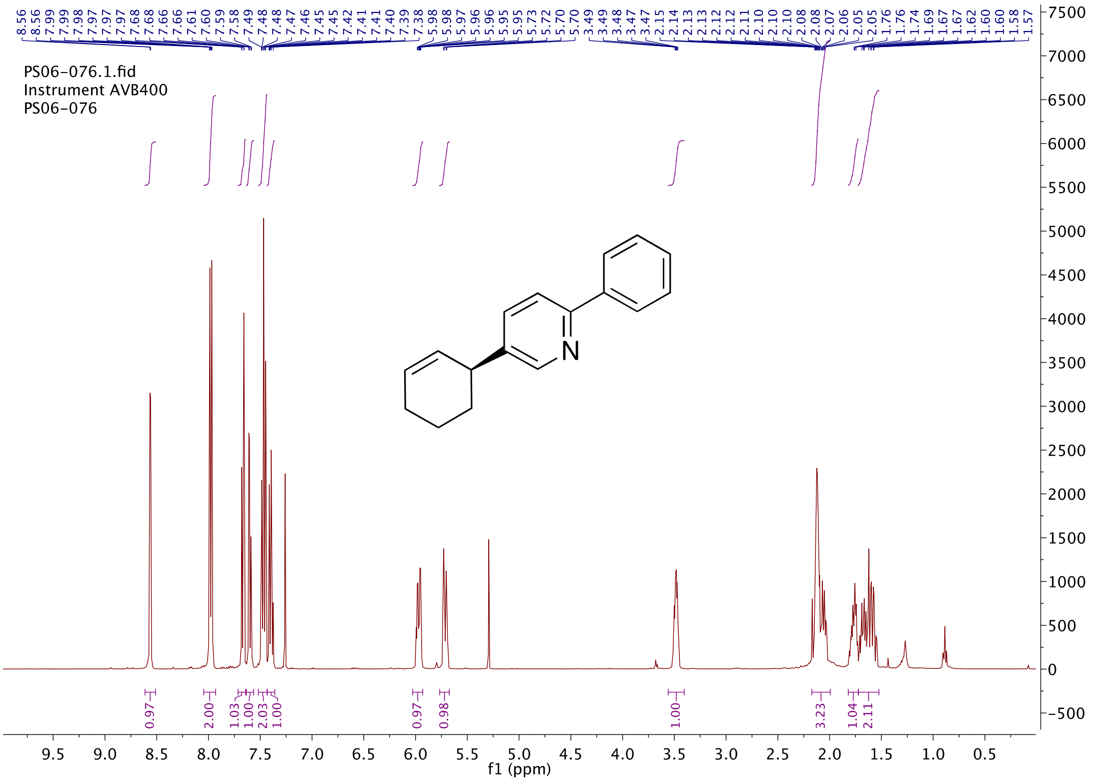

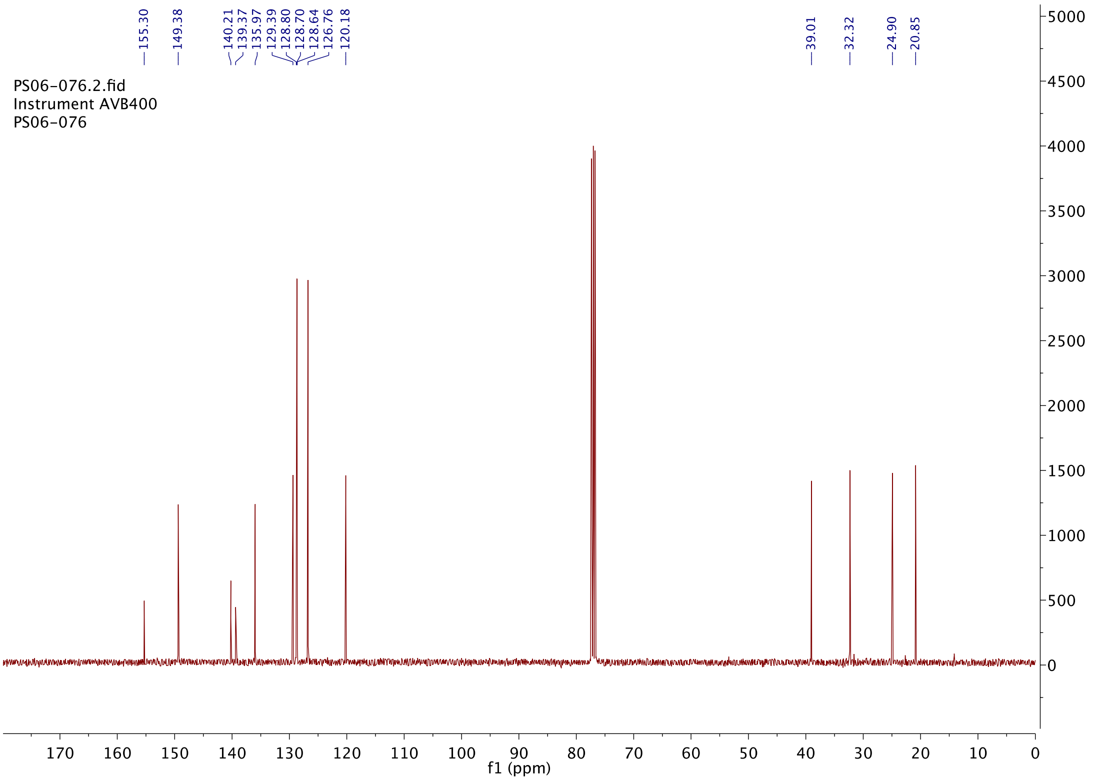

PS06-076.2.fid
Instrument AVB400
PS06-076
155.30
149.38
140.21
139.37
135.97
129.39
128.80
128.70
128.64
126.76
120.18
39.01
32.32
24.90
20.85
f1 (ppm)

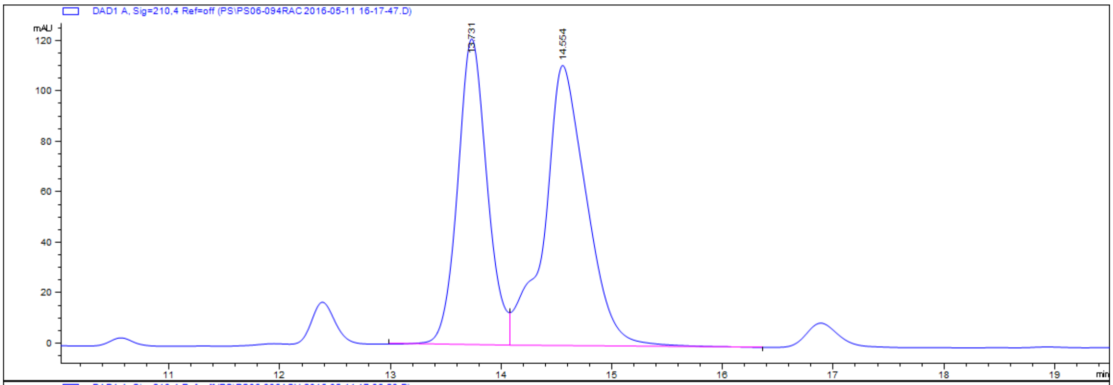

DAD1 A, Sig=210,4 Ref=off (PS\PS06-094RAC 2016-05-11 16-17-47.D)
mAU
13.731
14.554
min

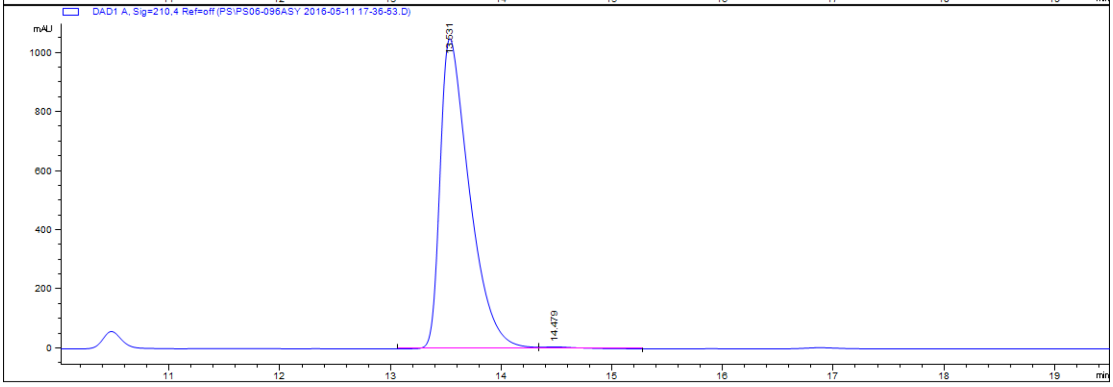

DAD1 A, Sig=210,4 Ref=off (PS\PS06-096ASY 2016-05-11 17-36-53.D)
mAU
13.531
14.479
min

Supplementary figure 78: ^{1}H, ^{13}C-NMR spectra of compound **75**

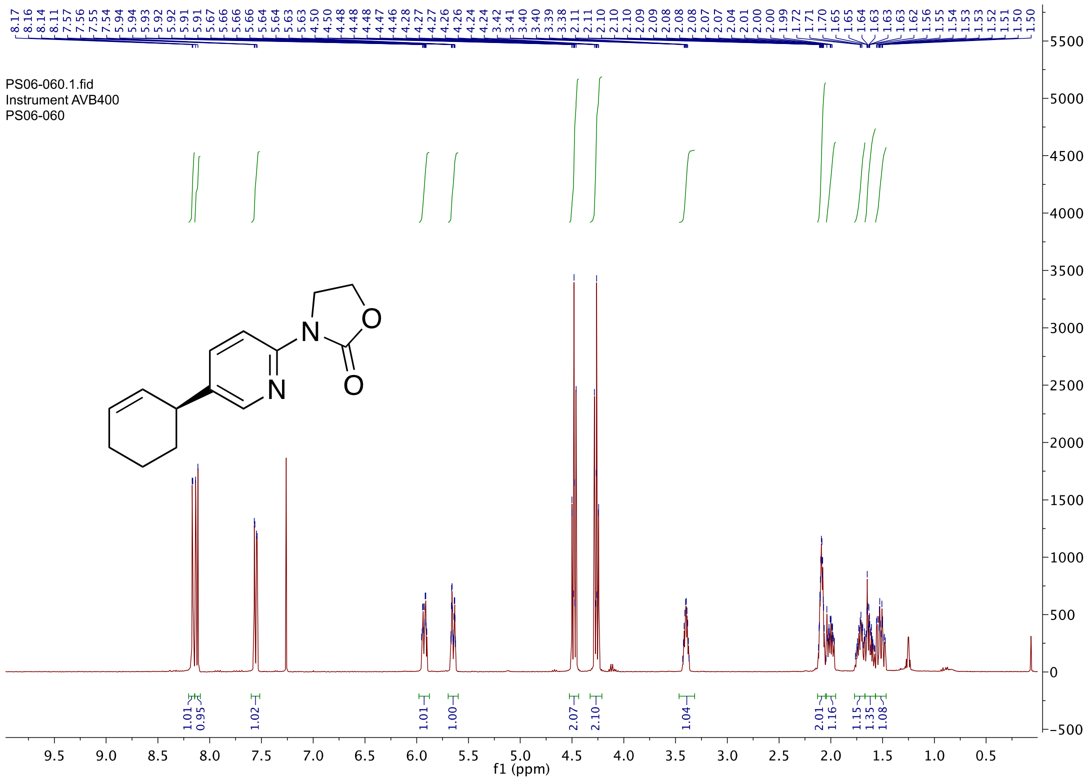

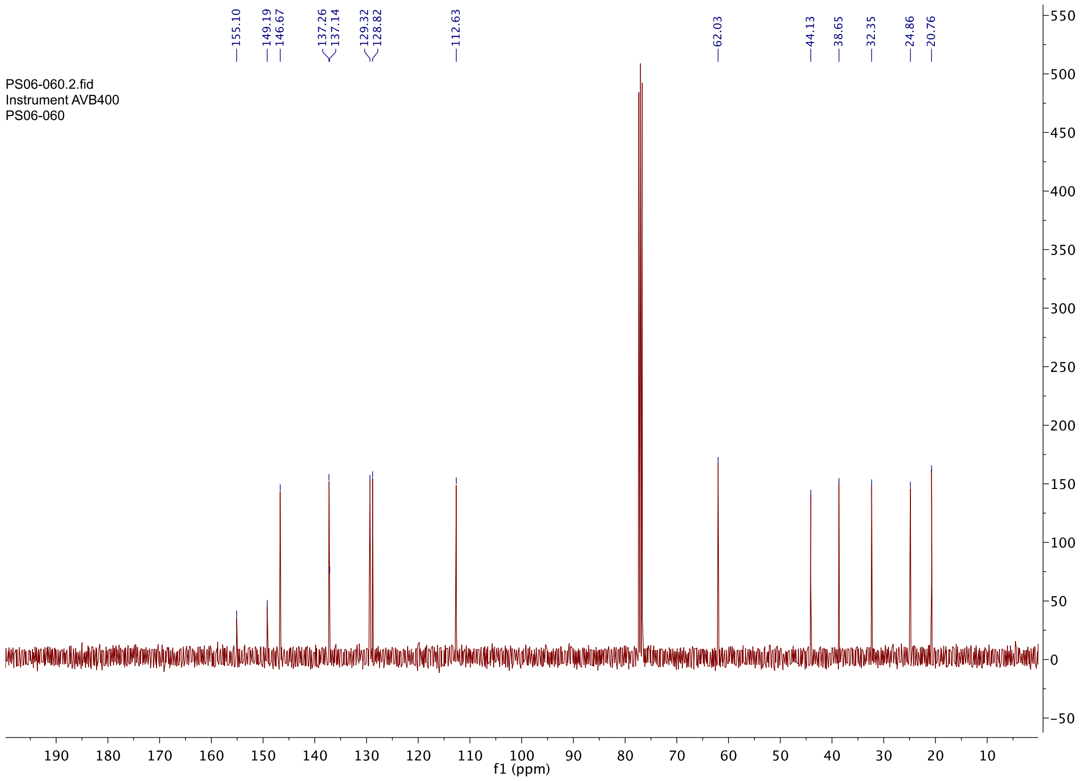

Supplementary figure 79: ^{1}H, ^{13}C-NMR spectra of compound **76**

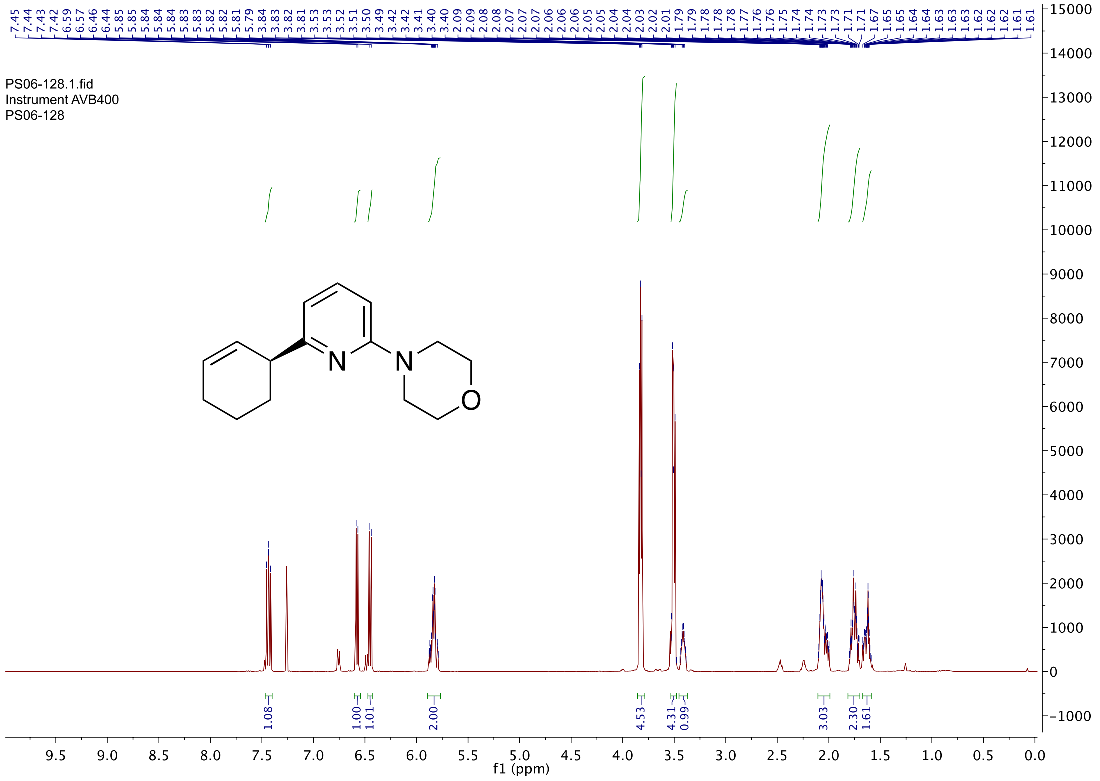

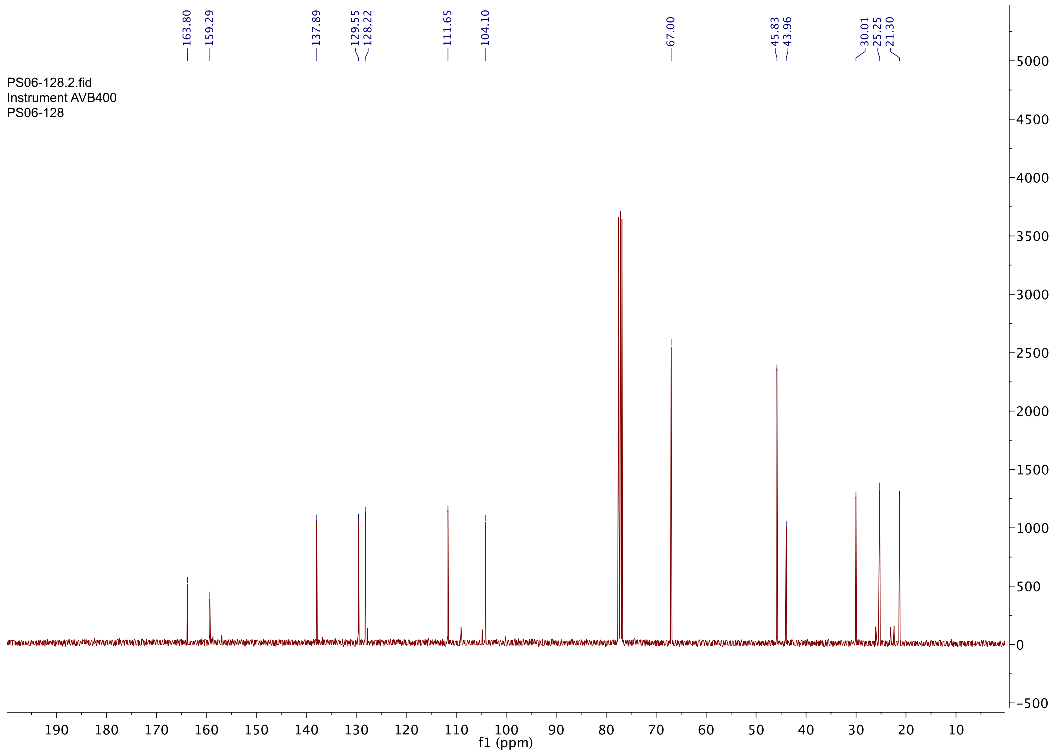

Supplementary figure 80: ^{1}H, ^{13}C-NMR spectra of compound **77**

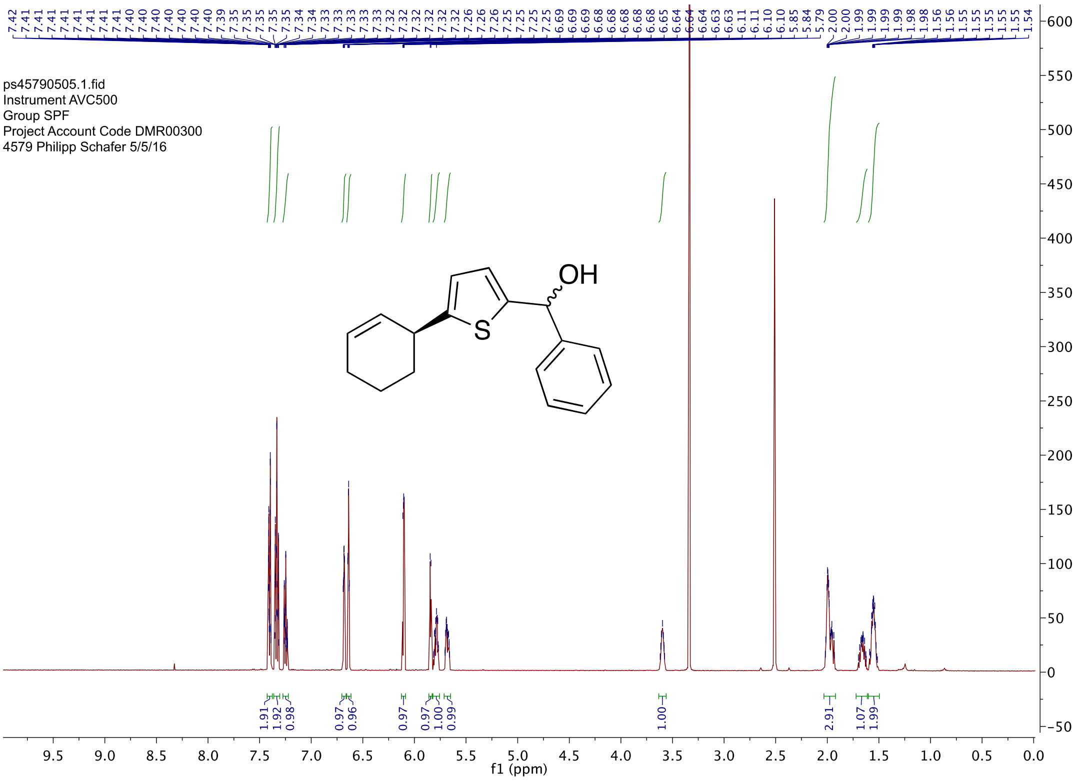

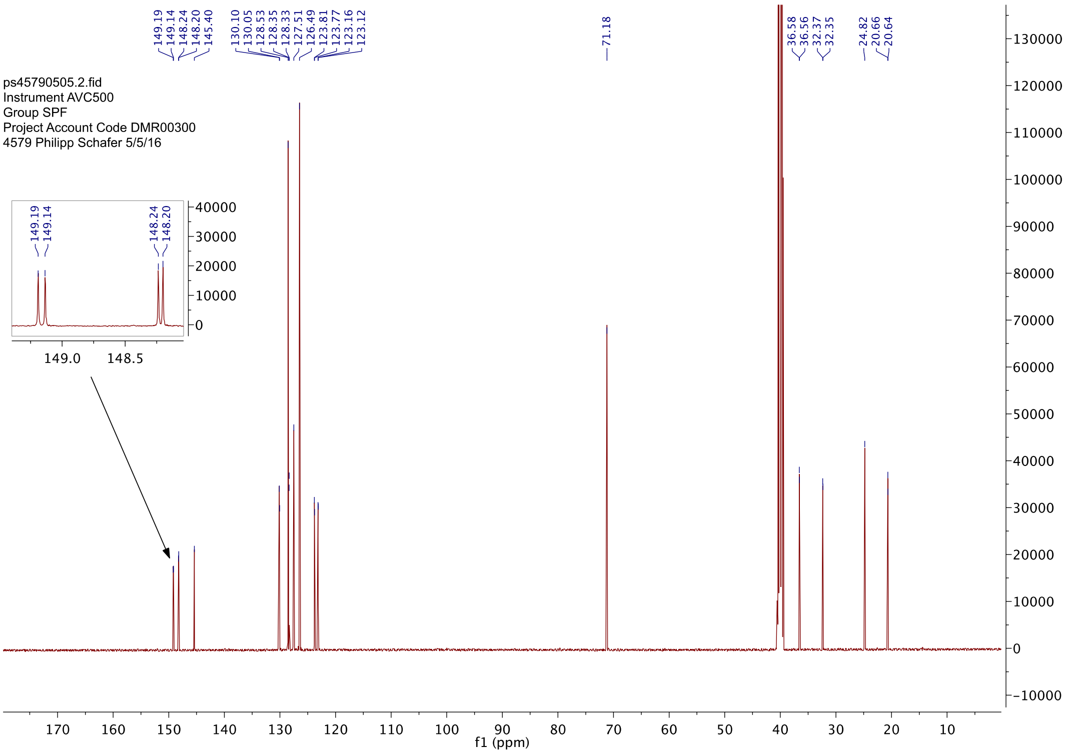

Supplementary figure 81: ^{1}H, ^{13}C-NMR spectra and HPLC traces of compound **78**

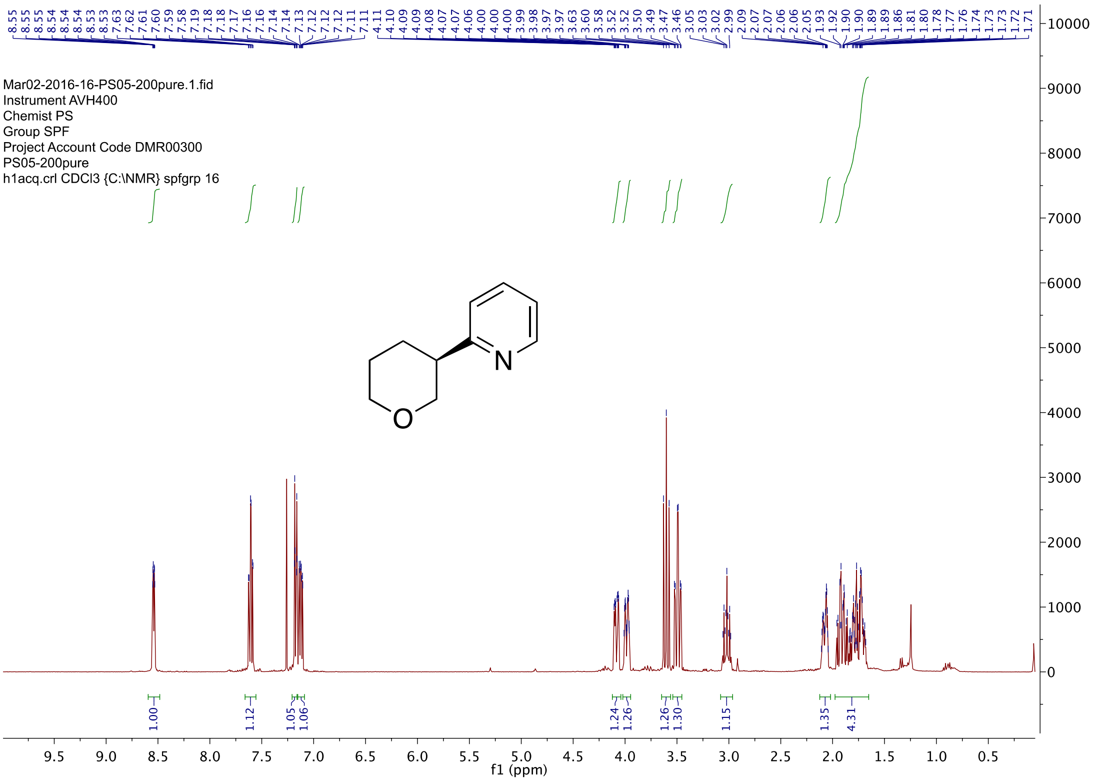

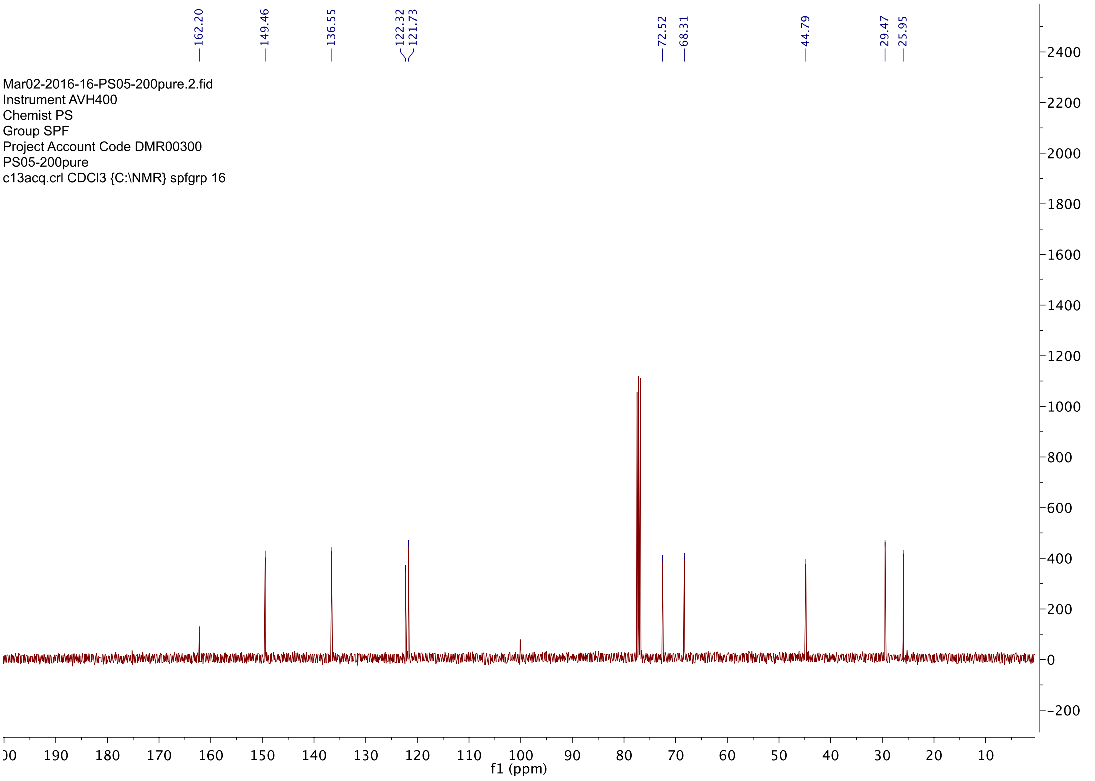

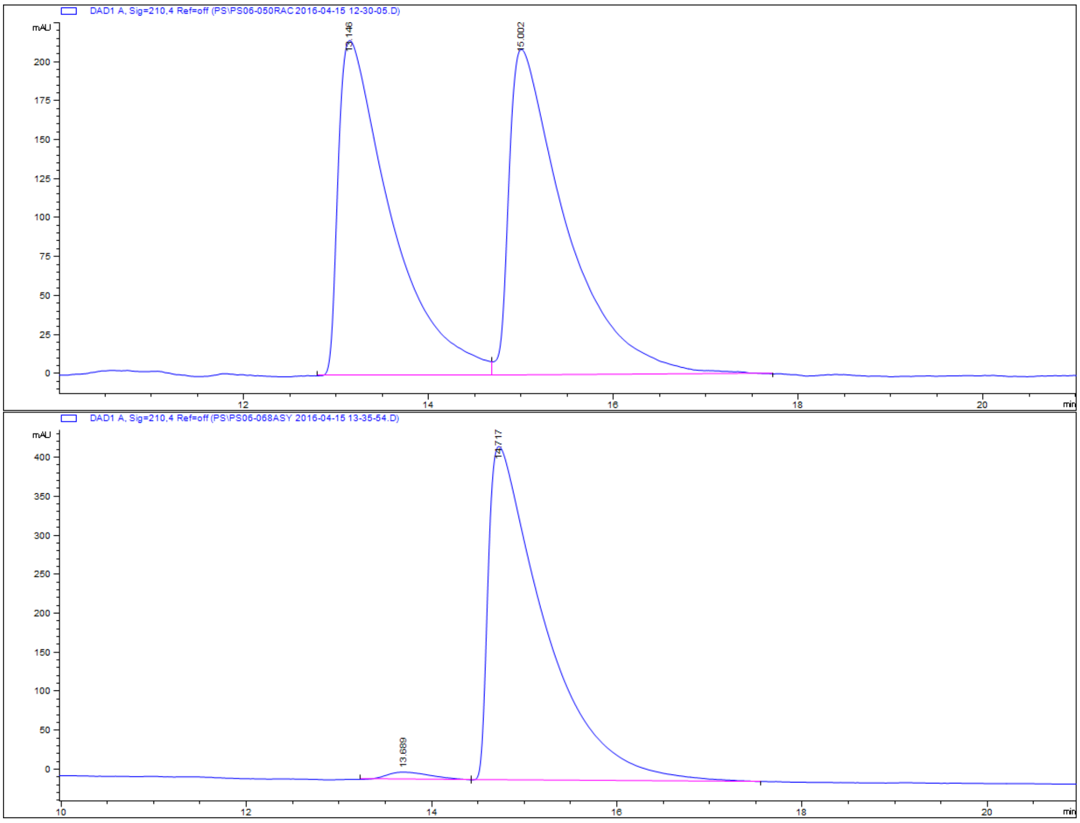

Supplementary figure 82: ^{1}H, ^{13}C-NMR spectra of compound **79**

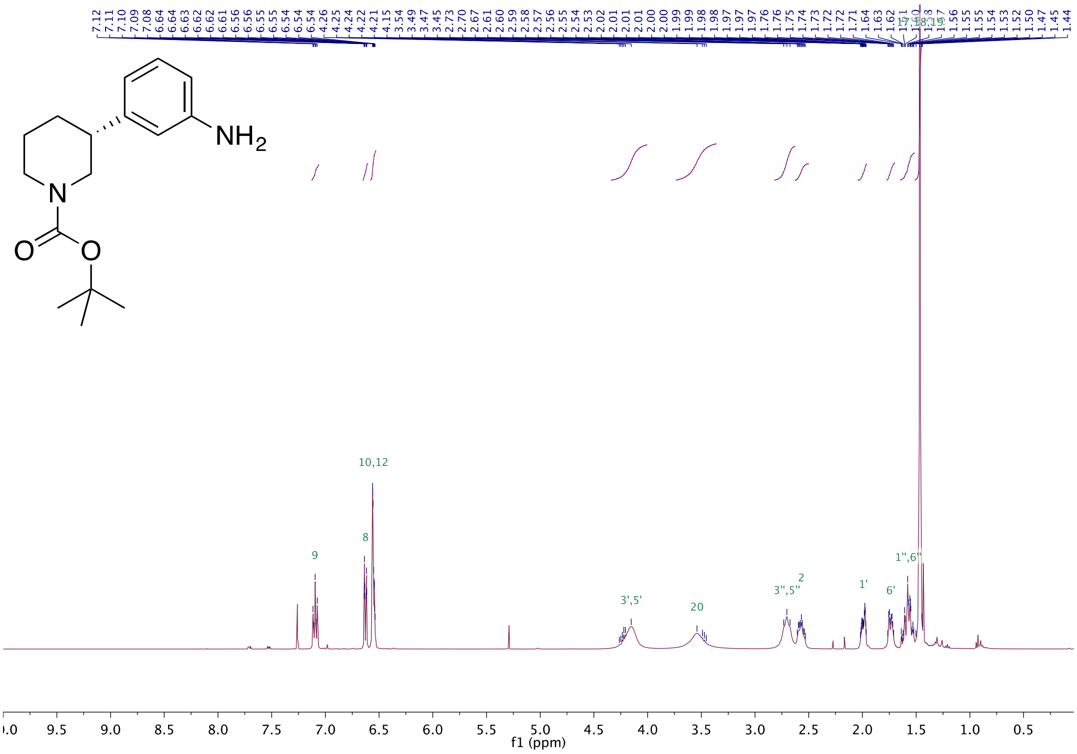

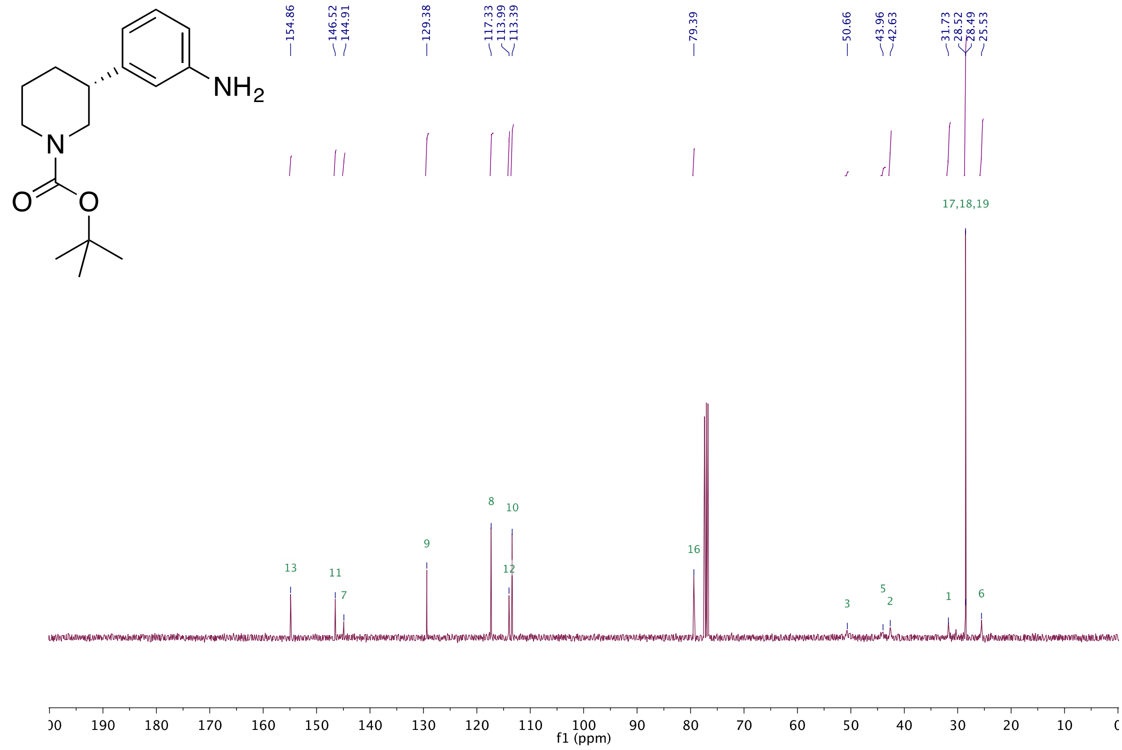

Supplementary figure 83: ^{1}H, ^{13}C-NMR spectra and HPLC traces of compound **80**

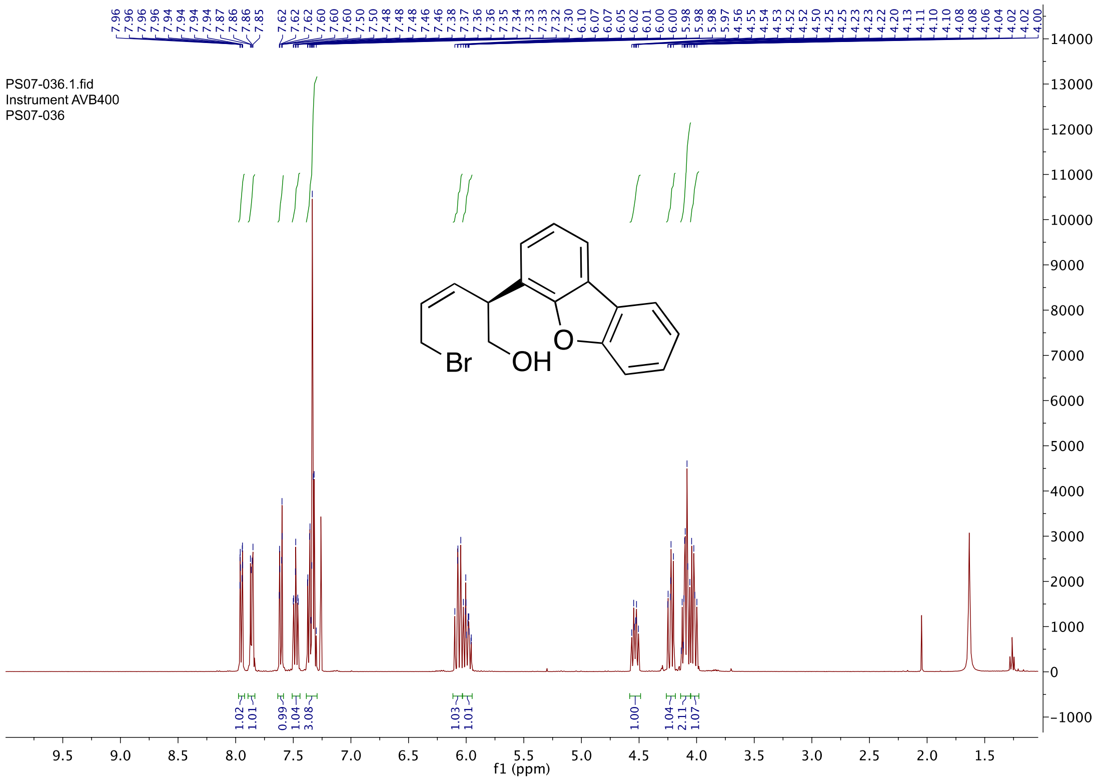

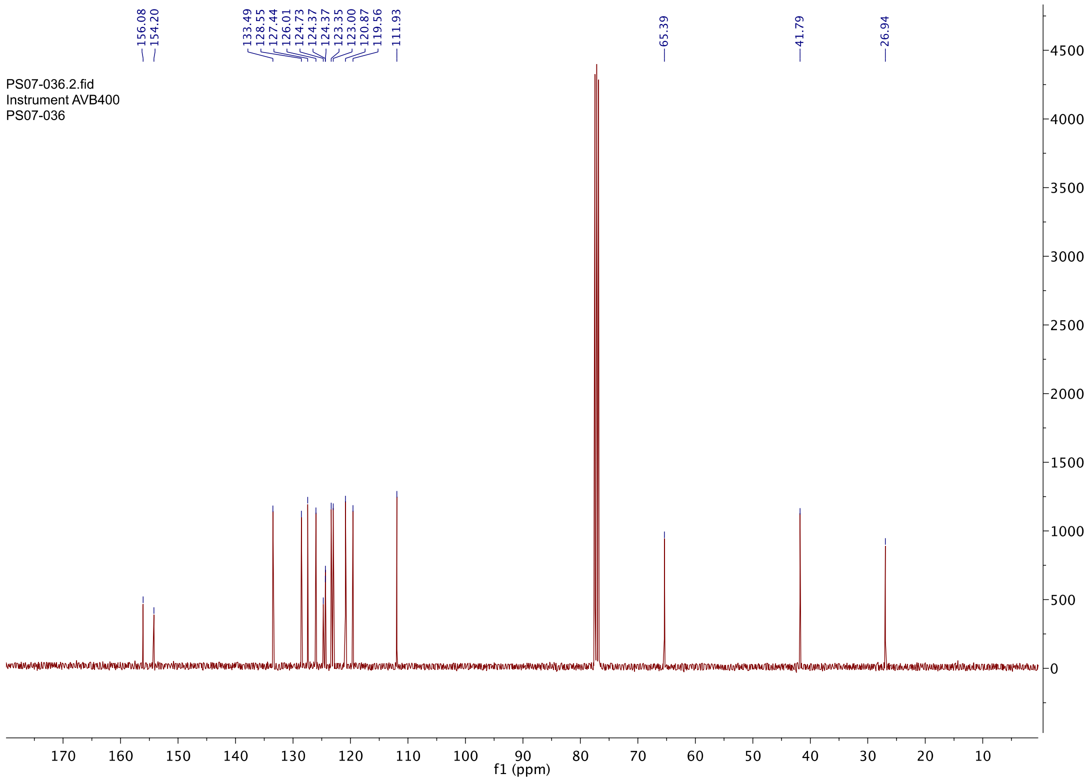
PS07-036.2.fid
Instrument AVB400
PS07-036
156.08
154.20
133.49
128.55
127.44
126.01
124.73
124.37
124.37
123.35
123.00
120.87
119.56
111.93
65.39
41.79
26.94
f1 (ppm)

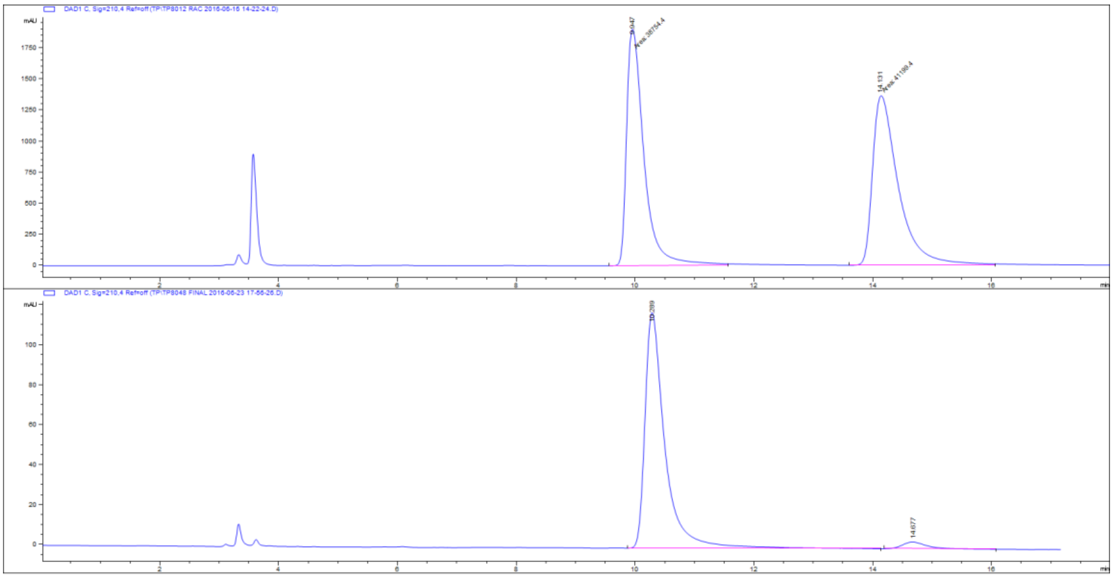
DAD1 C, Sig=210,4 Ref=off (TP\TP8012 RAC 2016-06-16 14-22-24.D)
mAU
14.131
DAD1 C, Sig=210,4 Ref=off (TP\TP8048 FINAL 2016-06-23 17-56-26.D)
mAU
14.677

Supplementary figure 84: ^{1}H, ^{13}C-NMR spectra and HPLC traces of compound **2H-ent-55**

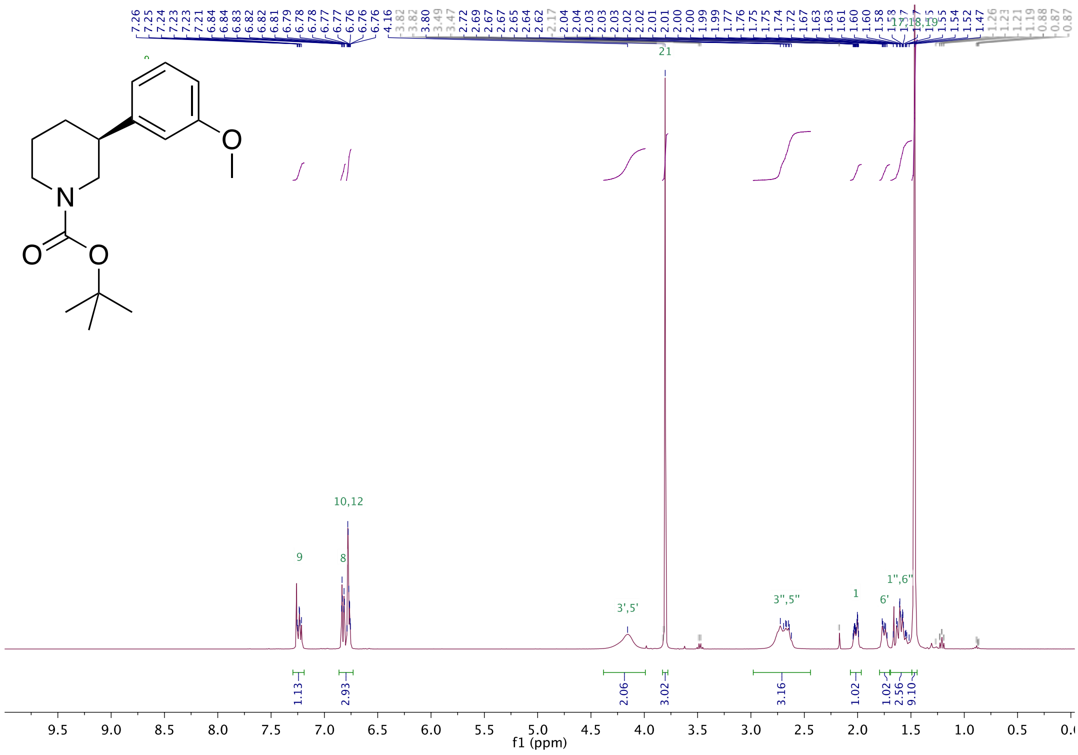

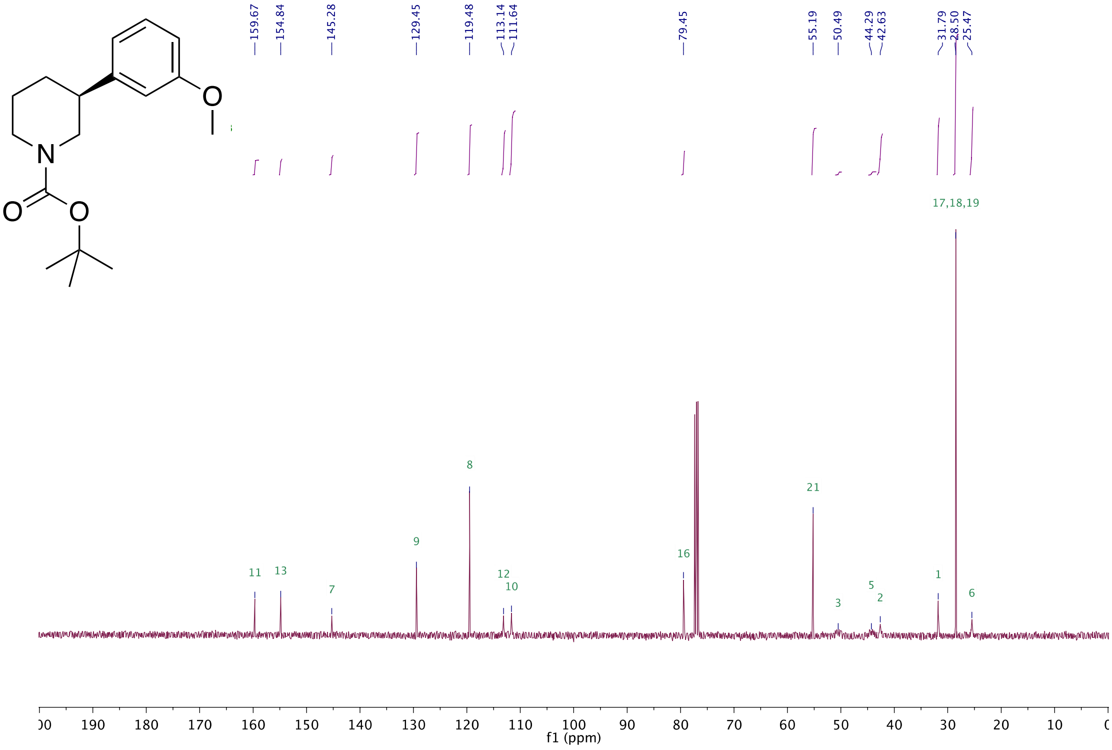

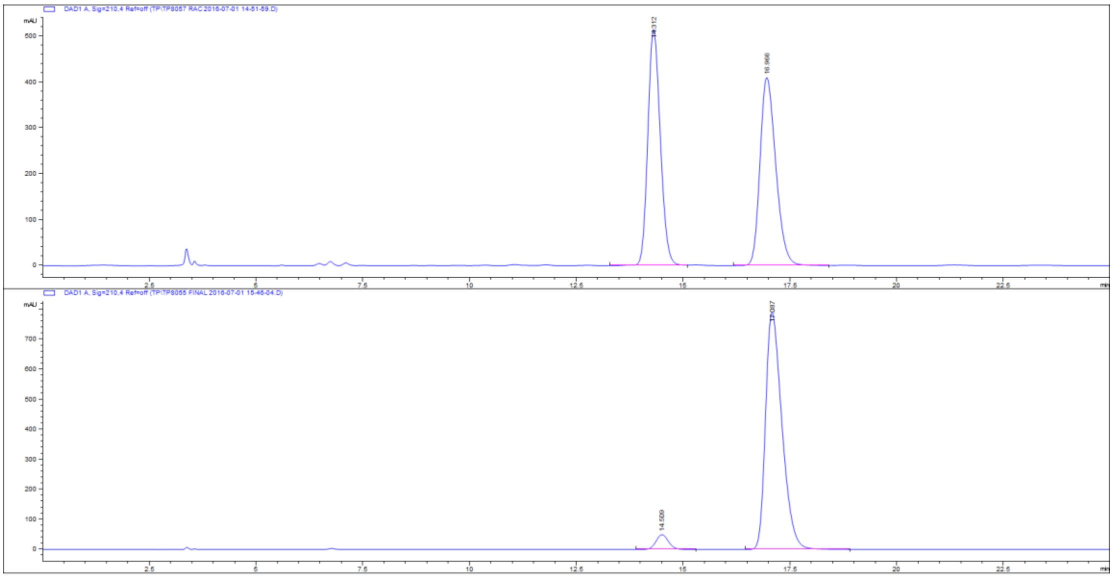

Supplementary figure 85: ^{1}H, ^{13}C-NMR spectra of compound **81**

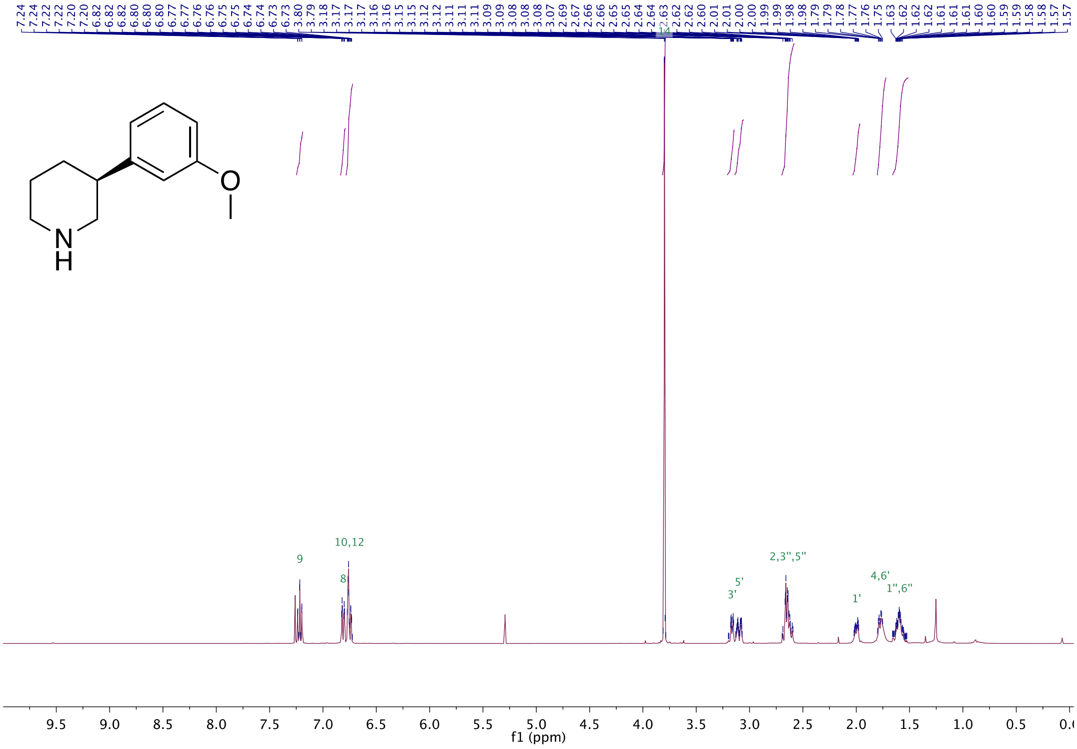

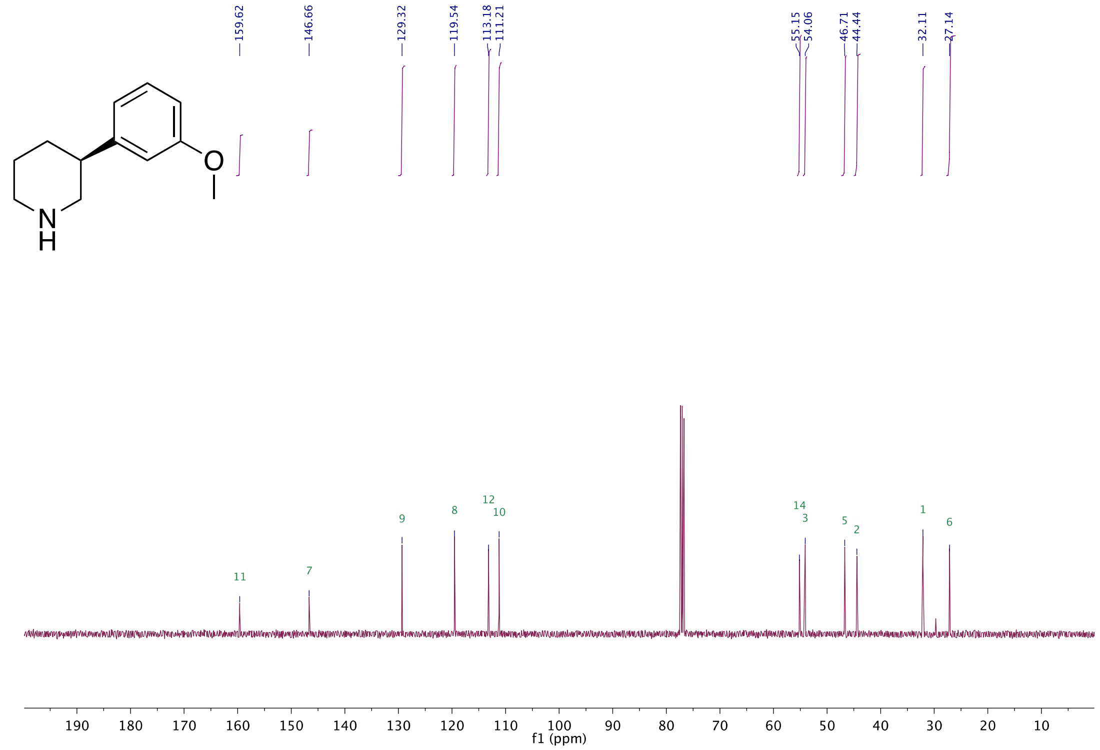
159.62
146.66
129.32
119.54
113.18
111.21
55.15
54.06
46.71
44.44
32.11
27.14
11
7
9
8
12
10
14
3
5
2
1
6
190
180
170
160
150
140
130
120
110
100
90
80
70
60
50
40
30
20
10
f1 (ppm)

Supplementary figure 86: ^{1}H, ^{13}C-NMR spectra and HPLC traces of compound **2H-ent-57**

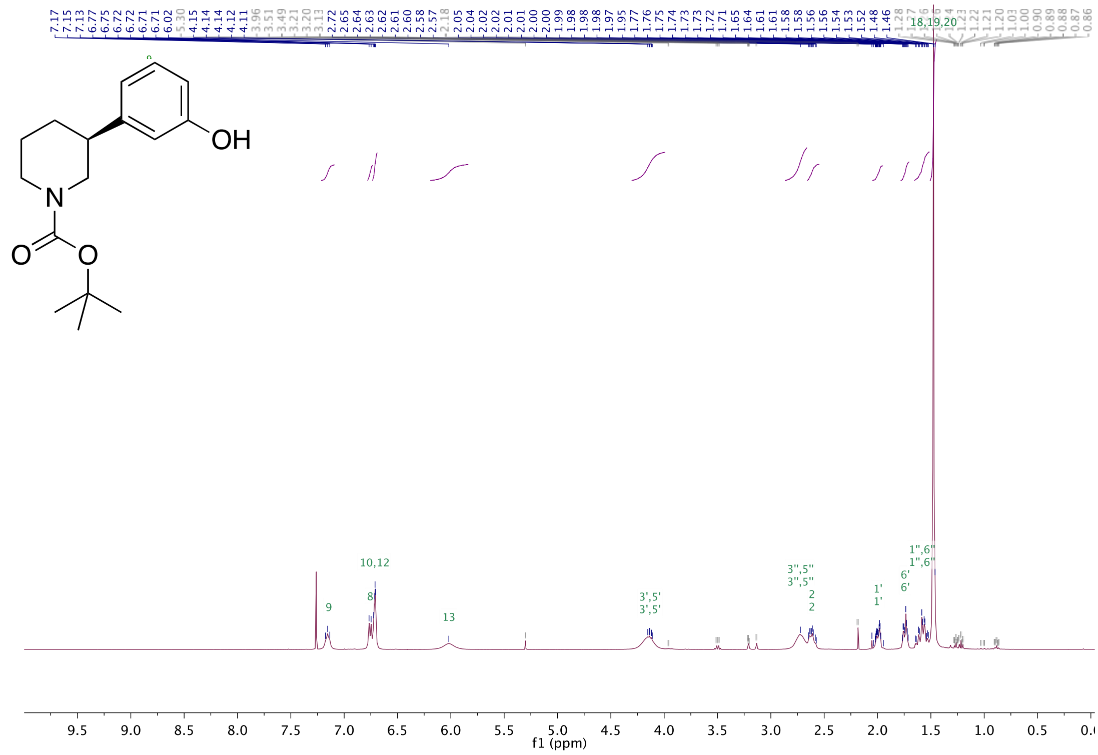

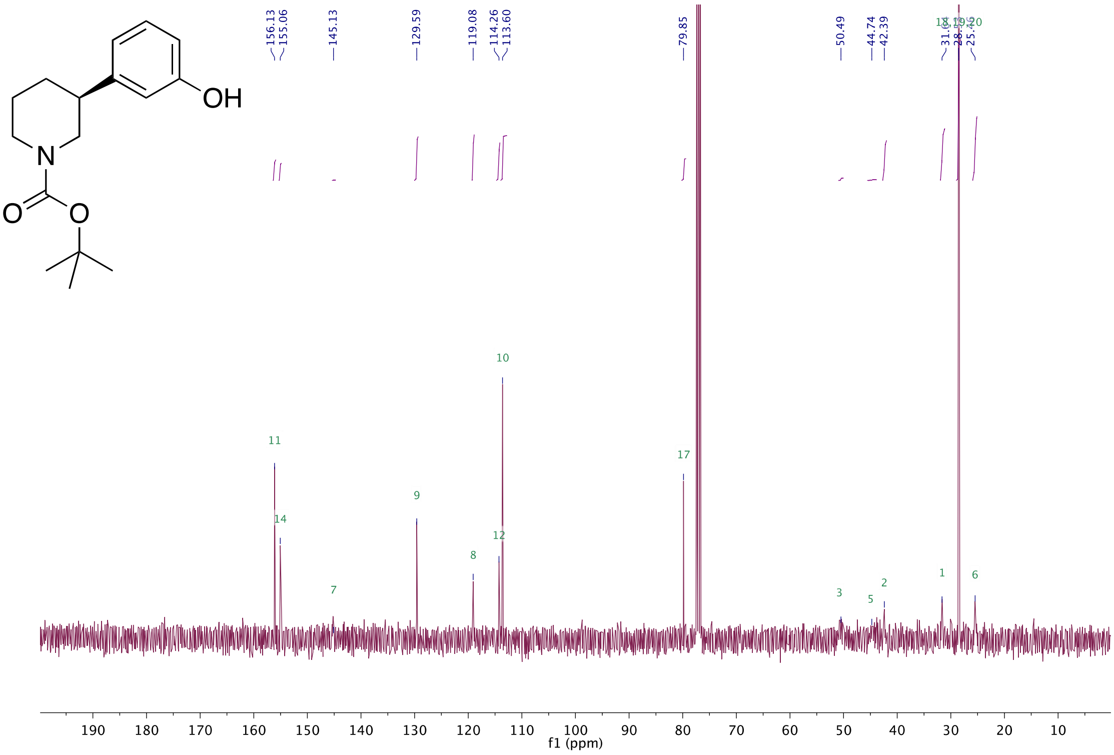

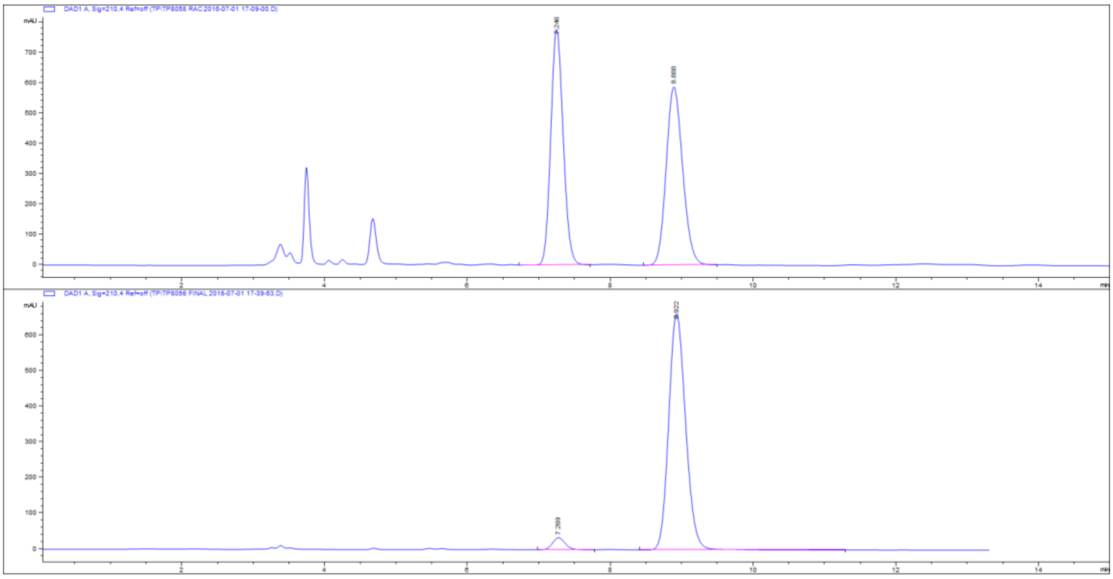

Supplementary figure 87: ^{1}H, ^{13}C-NMR spectra of compound **82**

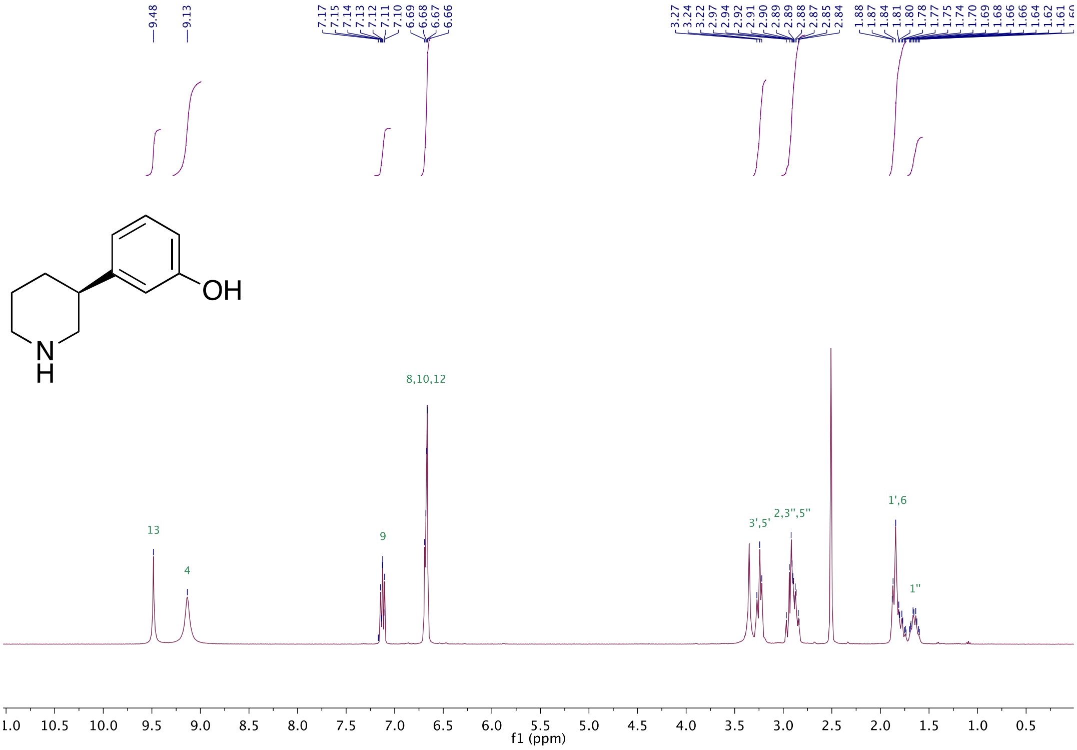

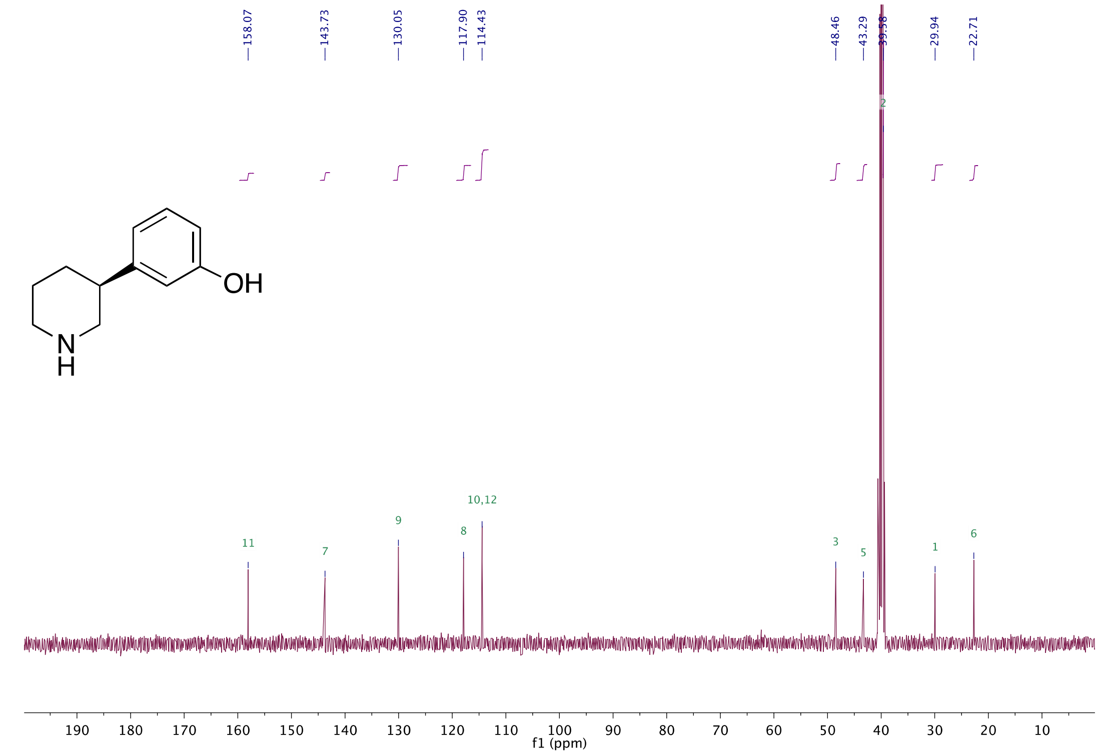
158.07
143.73
130.05
117.90
114.43
48.46
43.29
39.58
29.94
22.71
11
7
9
8
10,12
3
5
2
1
6
190 180 170 160 150 140 130 120 110 100 90 80 70 60 50 40 30 20 10
f1 (ppm)

Supplementary figure 88: ^{1}H, ^{13}C-NMR spectra of compound **(–)-preclamol**

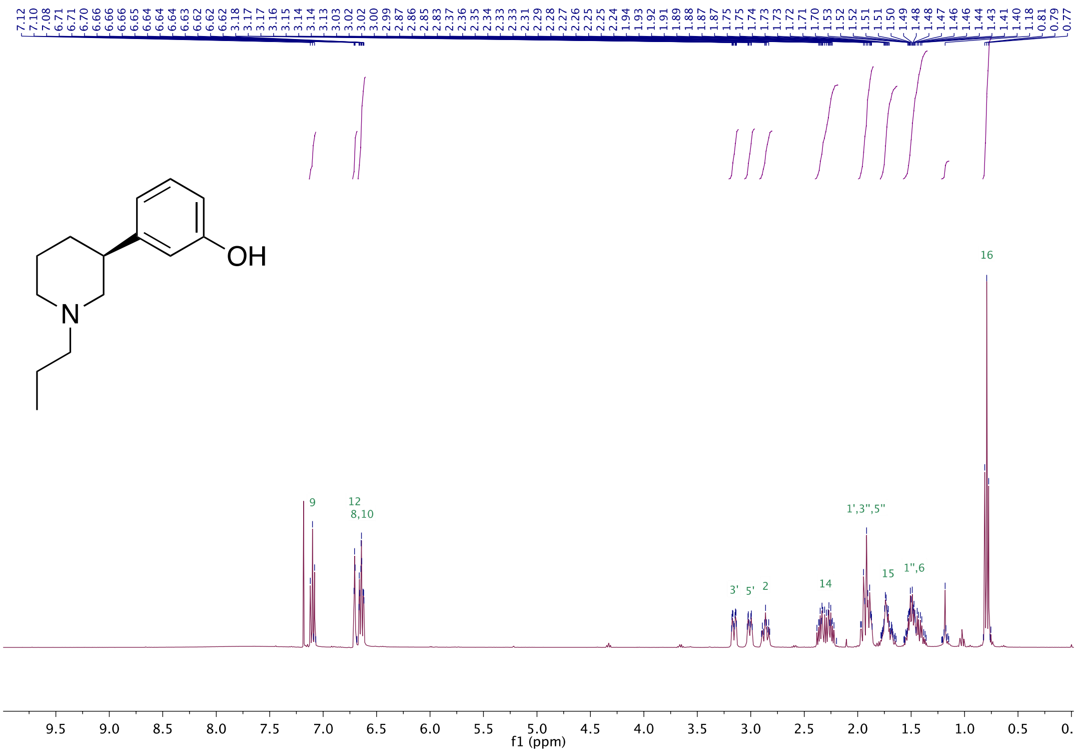

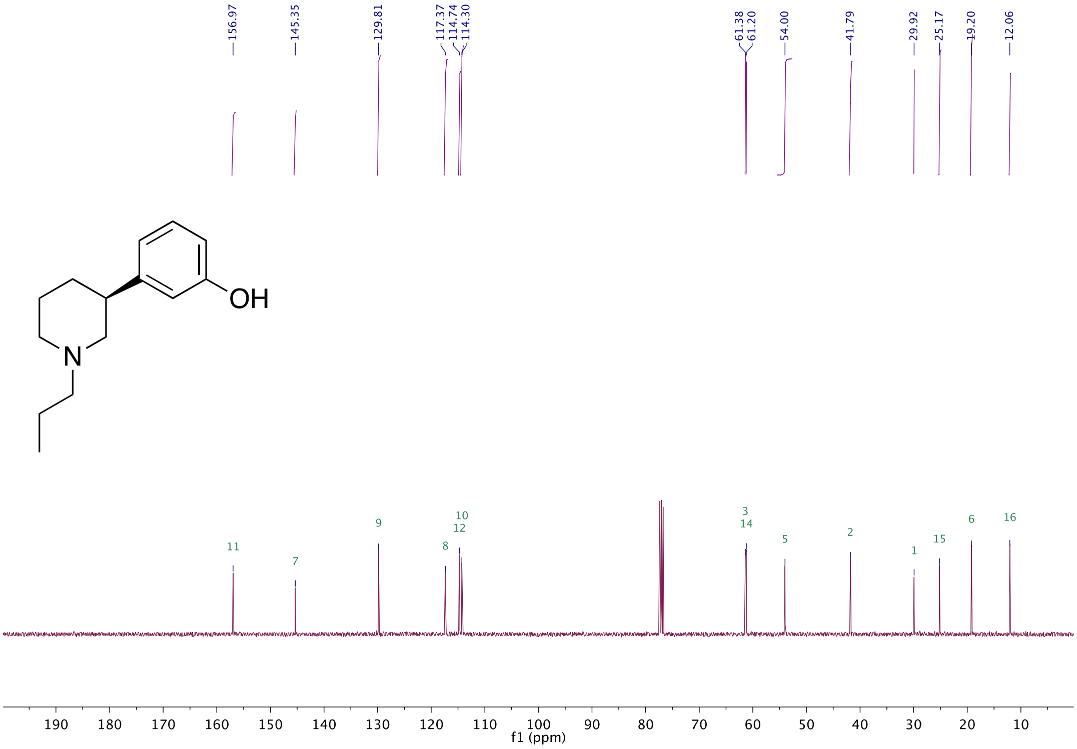

Supplementary figure 89: ^{1}H, ^{13}C-NMR spectra of compound **(+)-(*S*)-*N*-*tert*-Butoxycarbonyl-3-(2-chloro-6-pyridinyl)-piperidine**

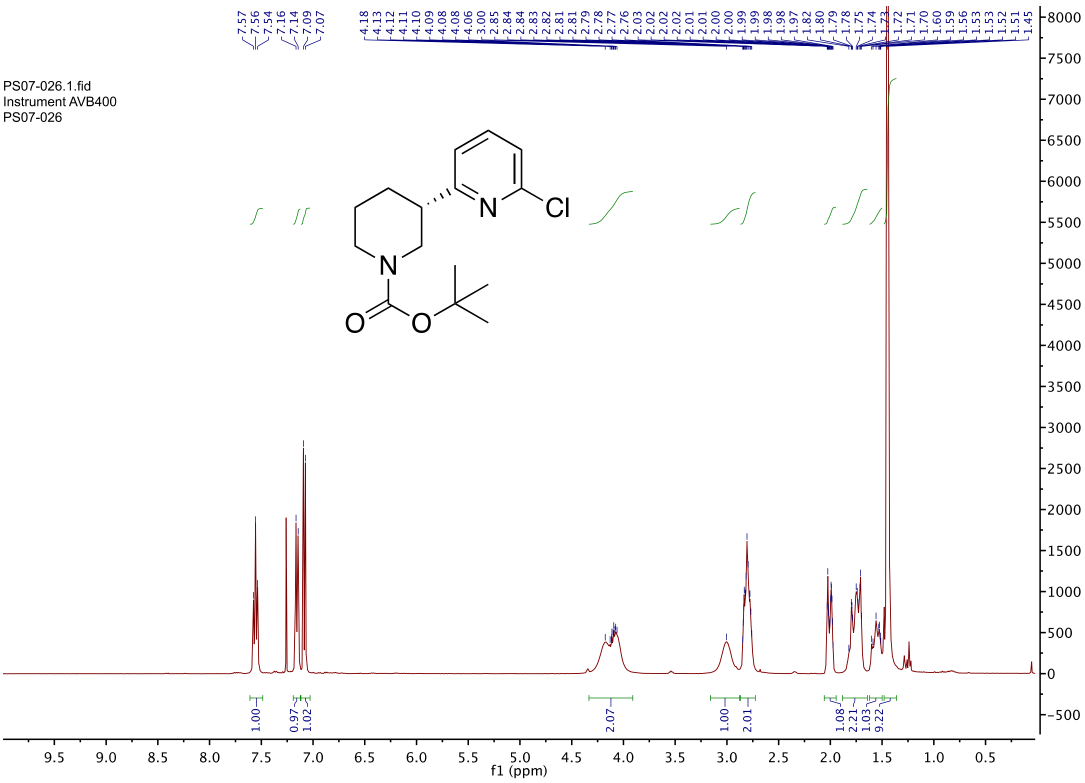

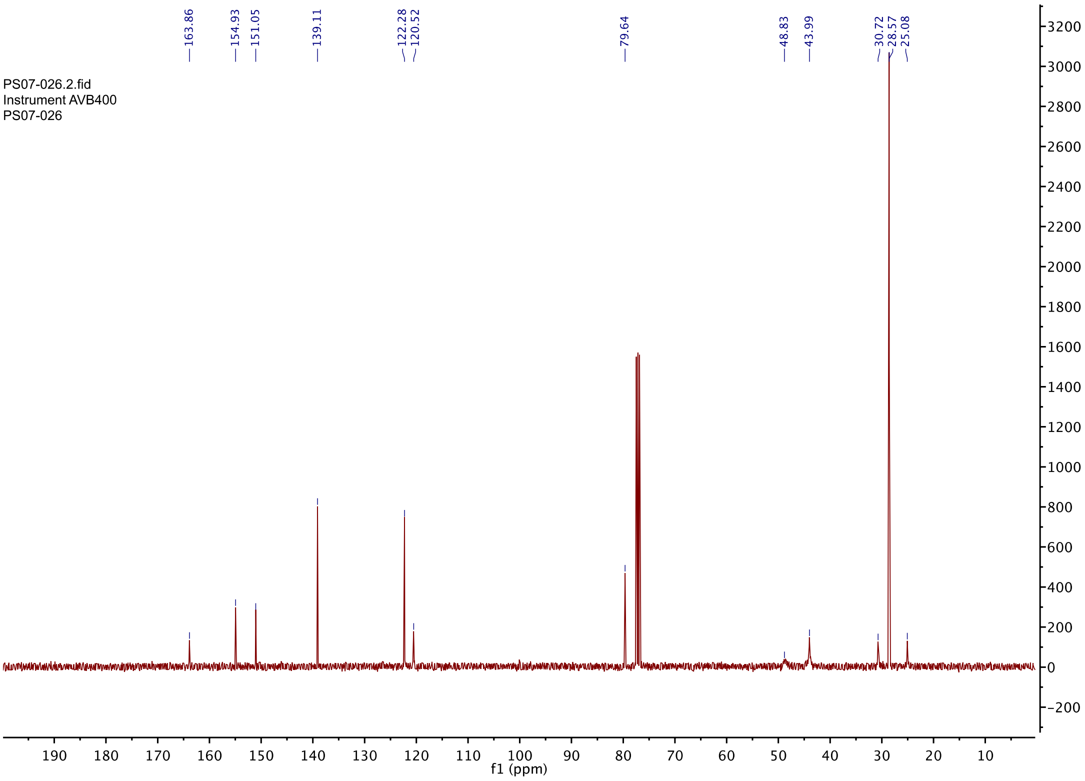

Supplementary figure 90: ^{1}H, ^{13}C-NMR spectra and HPLC traces of compound **(+)-(*S*)-*N*-*tert*-butoxycarbonyl-3-(2-pyridinyl)-piperidine**

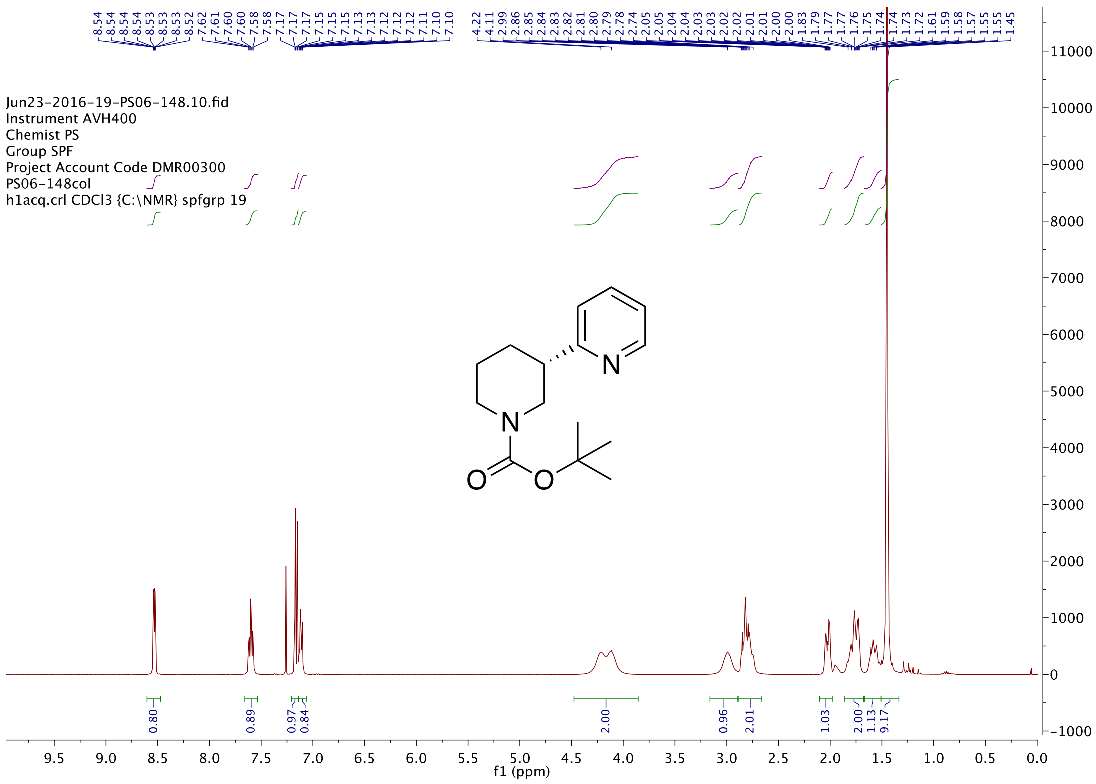

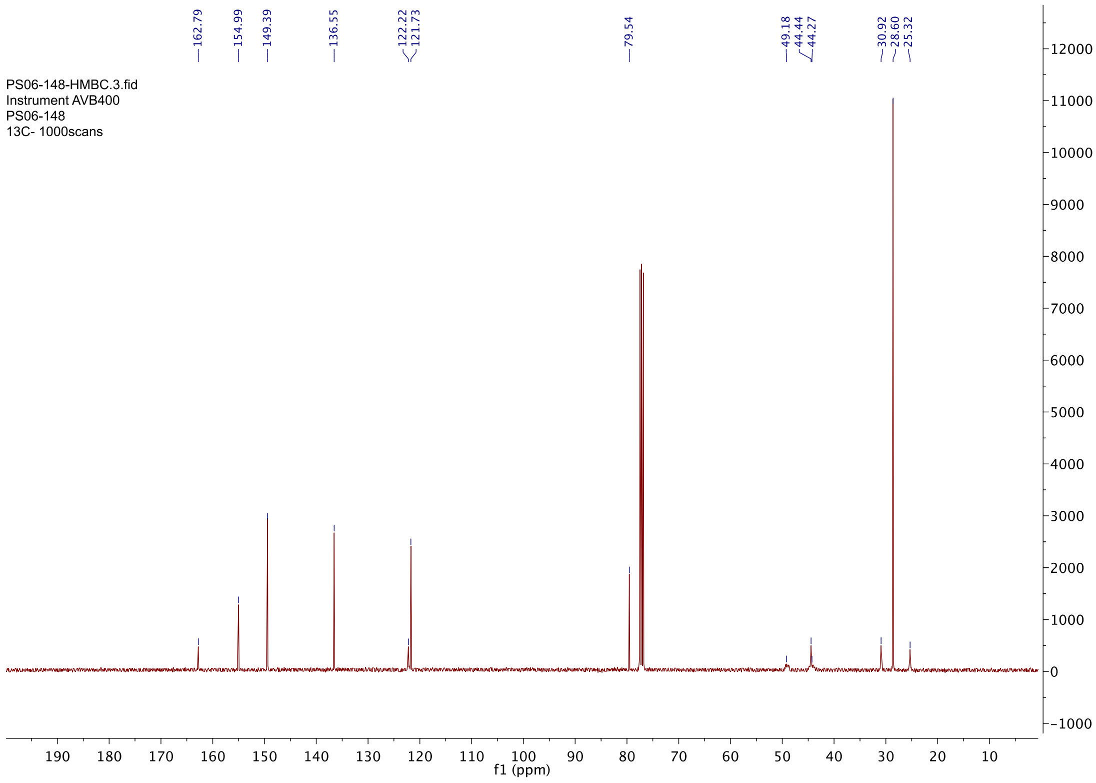

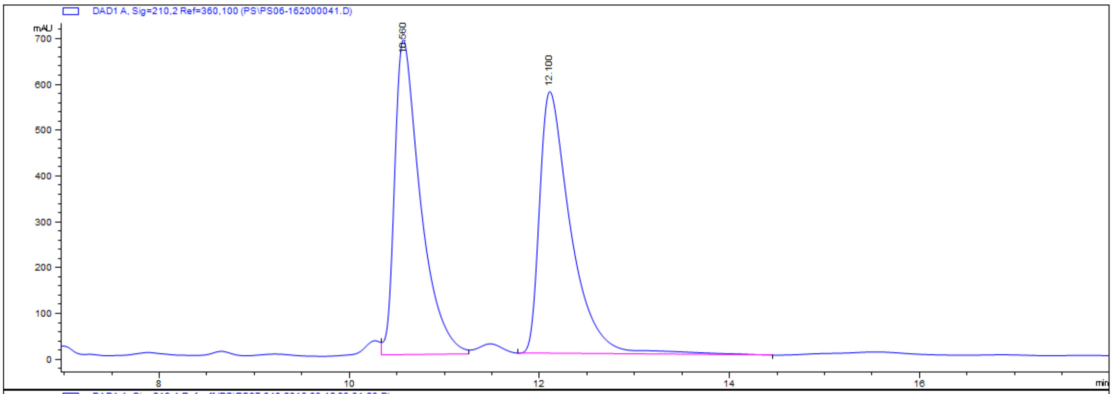
DAD1 A, Sig=210,2 Ref=360,100 (PS\PS06-162000041.D)
mAU
700
600
500
400
300
200
100
0
10.560
12.100
8
10
12
14
16
min

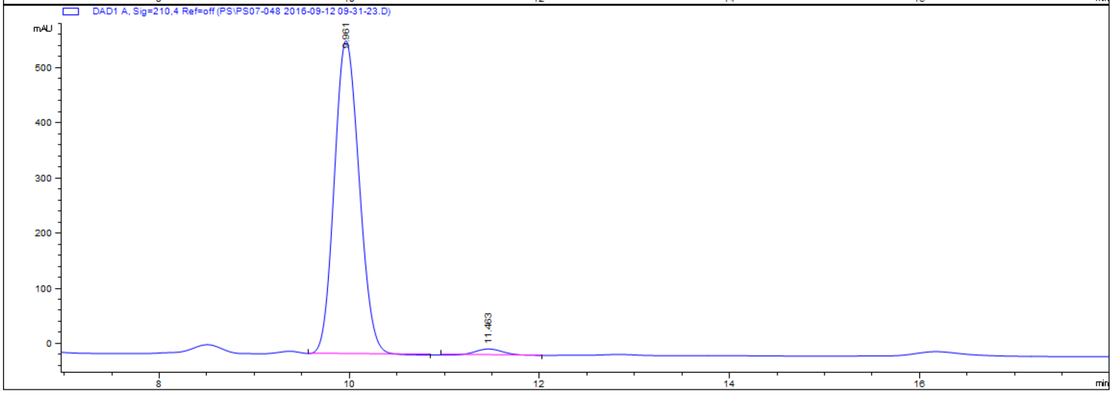
DAD1 A, Sig=210,4 Ref=off (PS\PS07-048 2016-09-12 09-31-23.D)
mAU
500
400
300
200
100
0
9.961
11.463
8
10
12
14
16
min

Supplementary figure 91: ^{1}H, ^{13}C-NMR spectra of compound **(+)-(*S*)-isoanabasine**

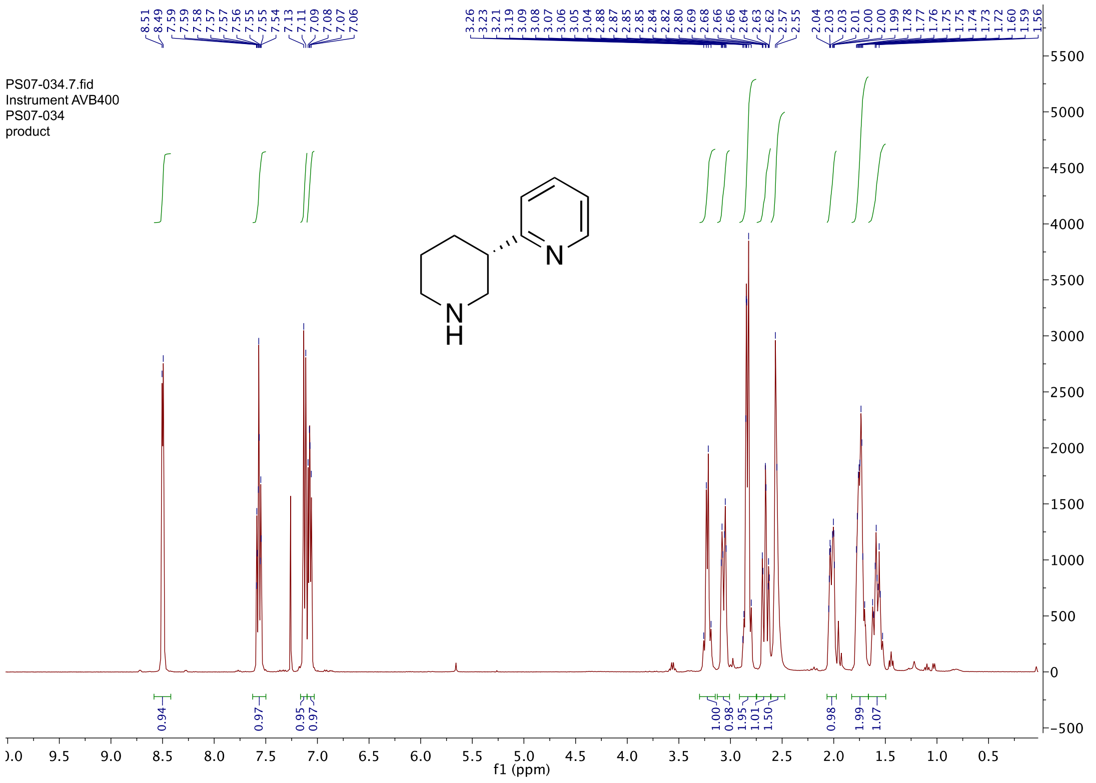

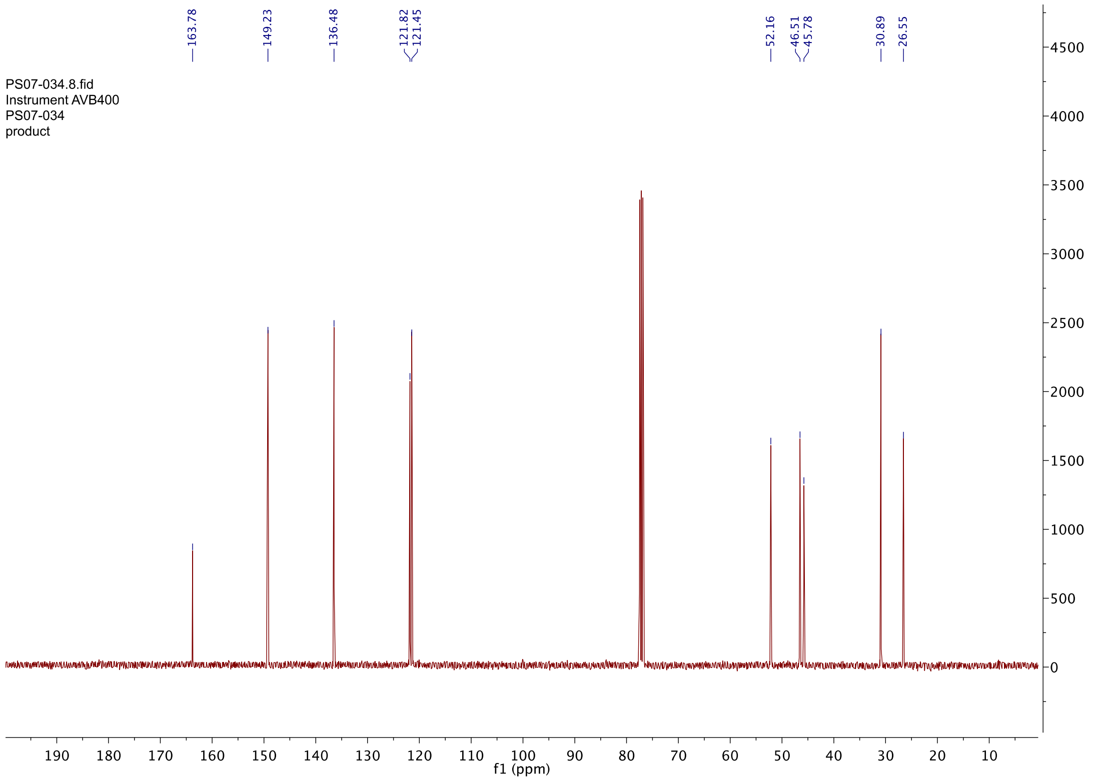

Supplementary figure 92: ^{1}H, ^{13}C-NMR spectra and HPLC traces of compound **83**

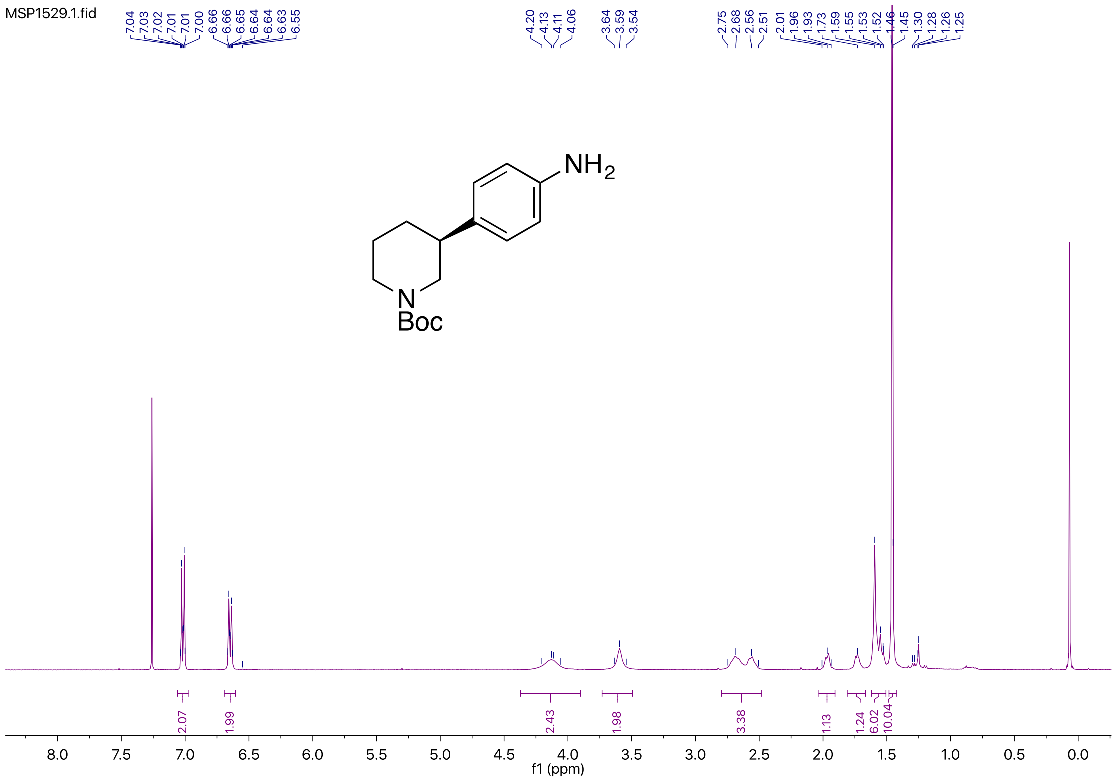

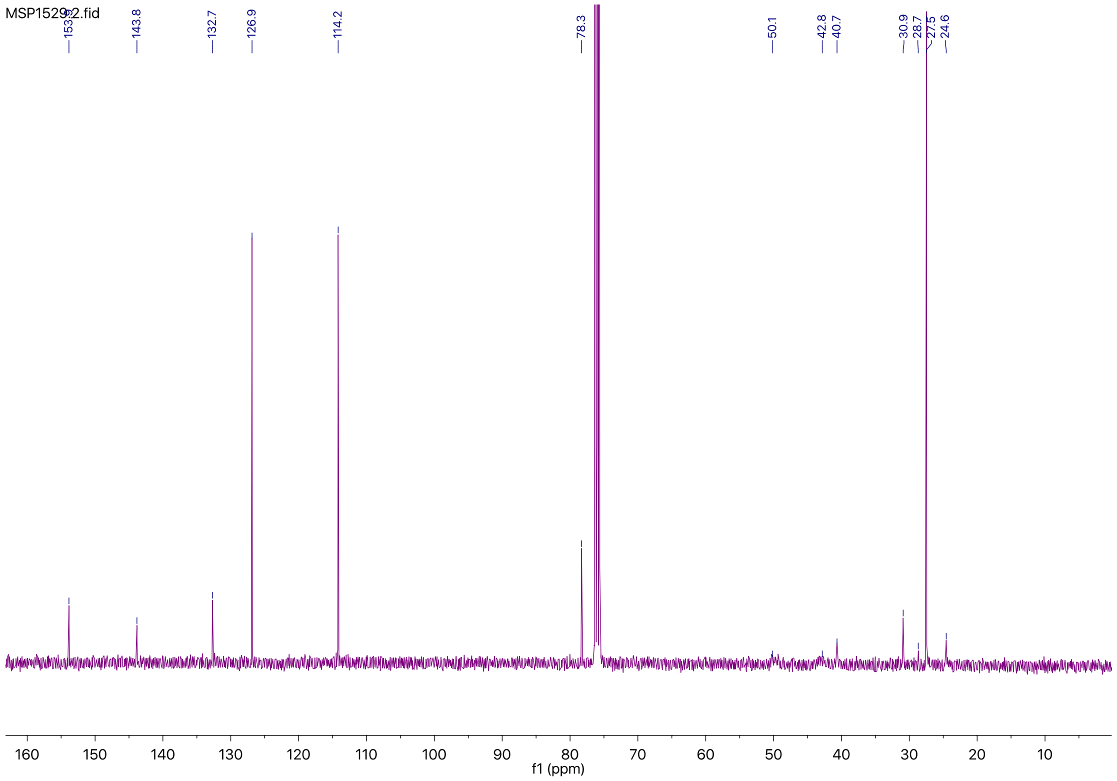

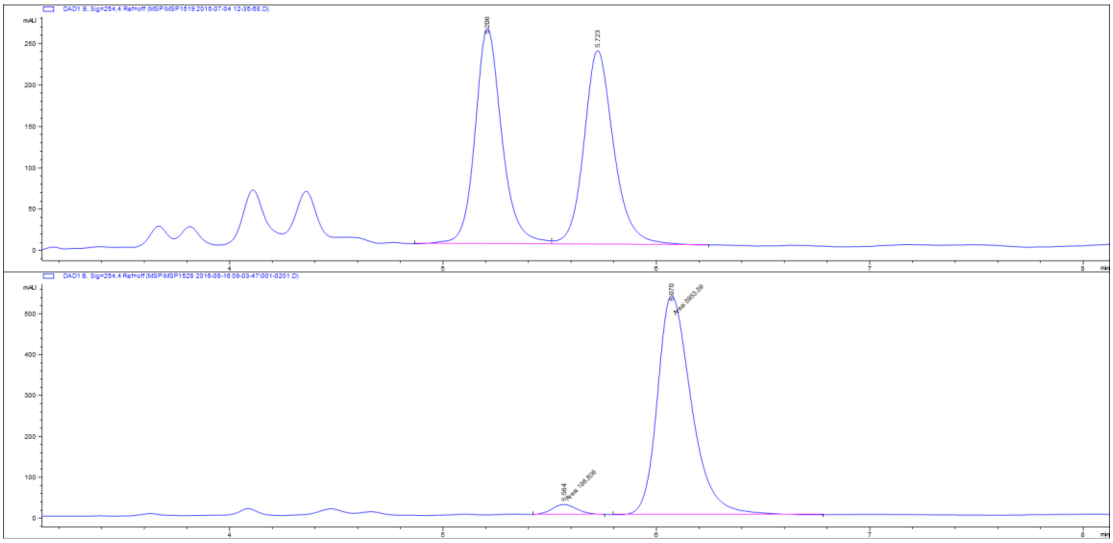

Supplementary figure 93: ^{1}H, ^{13}C-NMR spectra and HPLC traces of compound **84**

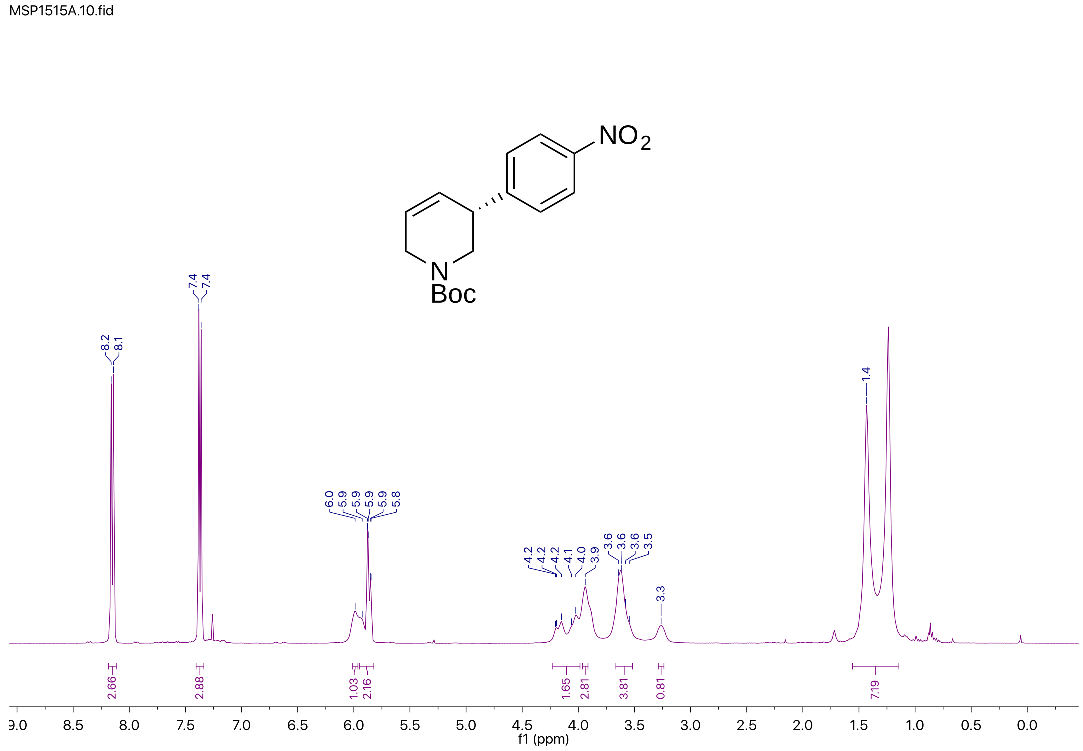

MSP1515A.11.fid

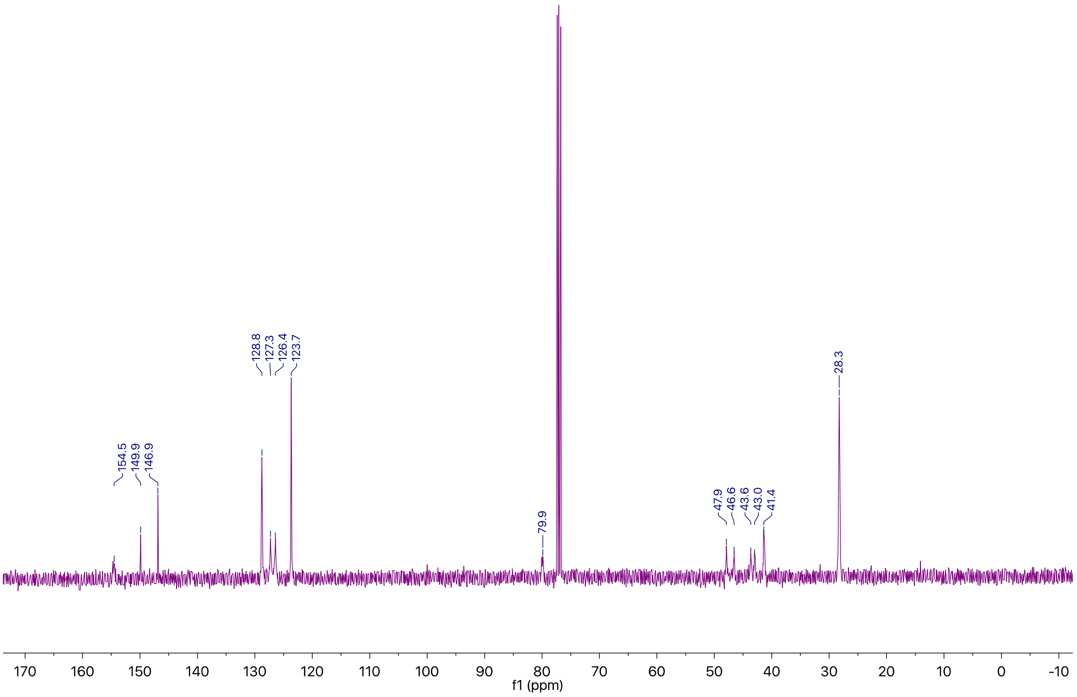

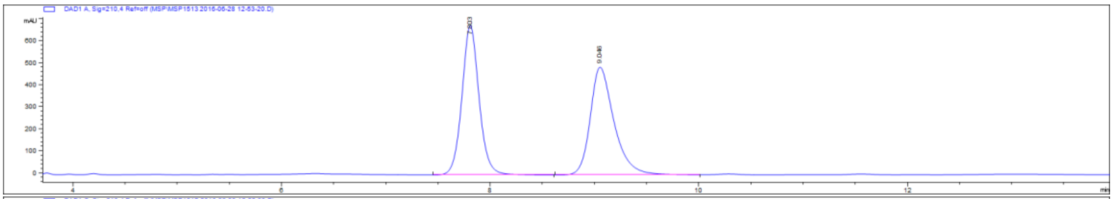

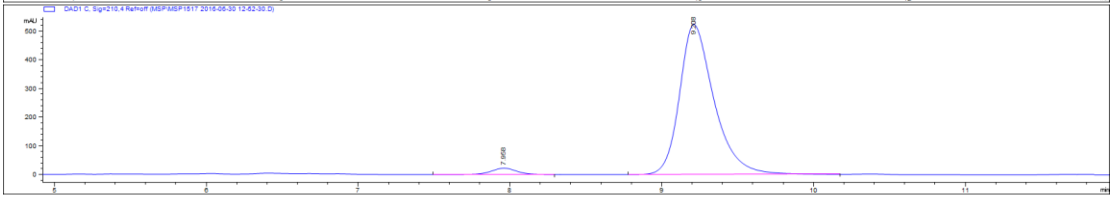

Supplementary figure 94: ^{1}H, ^{13}C-NMR spectra and HPLC traces of compound **85**

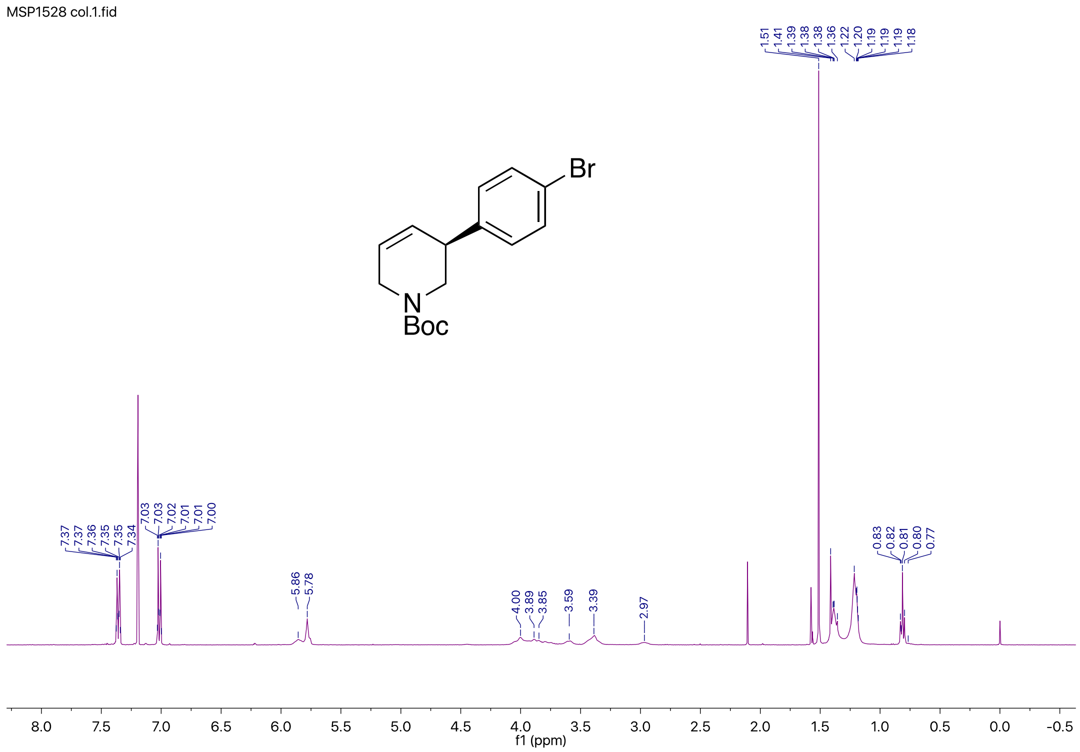

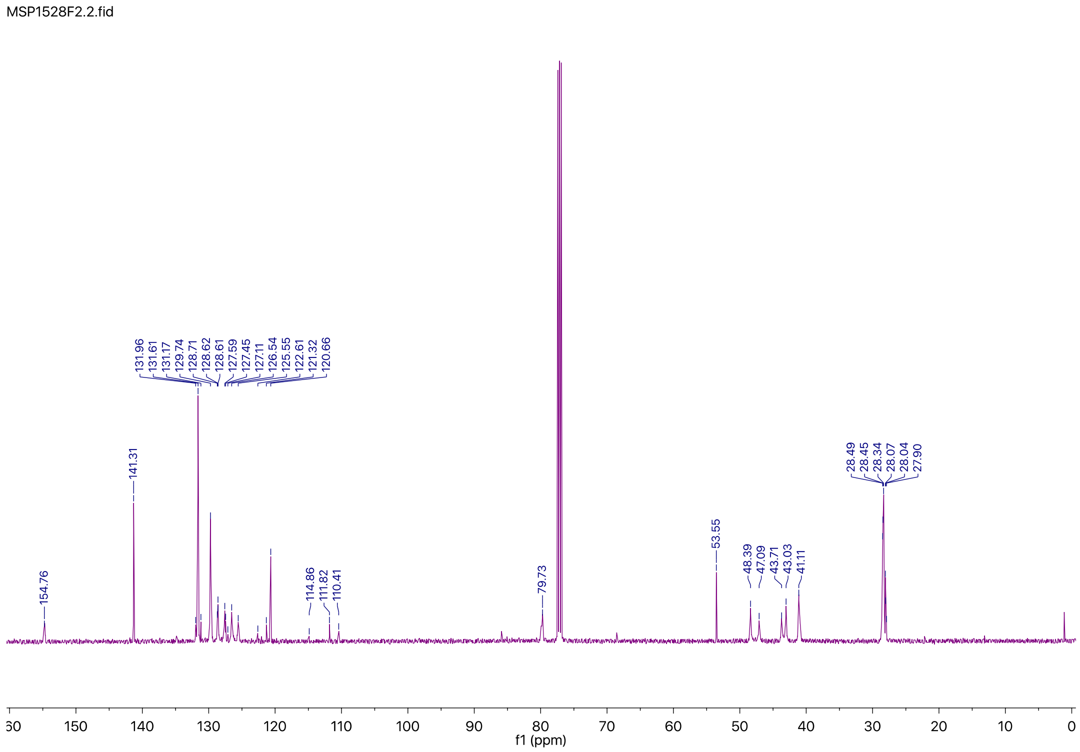

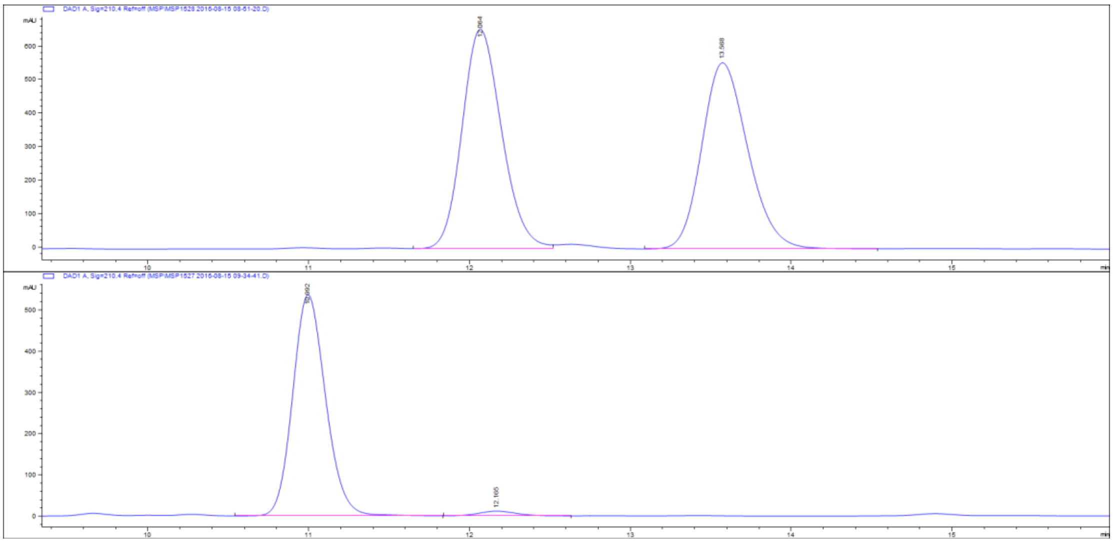

Supplementary figure 95: ^{1}H, ^{13}C-NMR spectra and HPLC traces of compound **86**

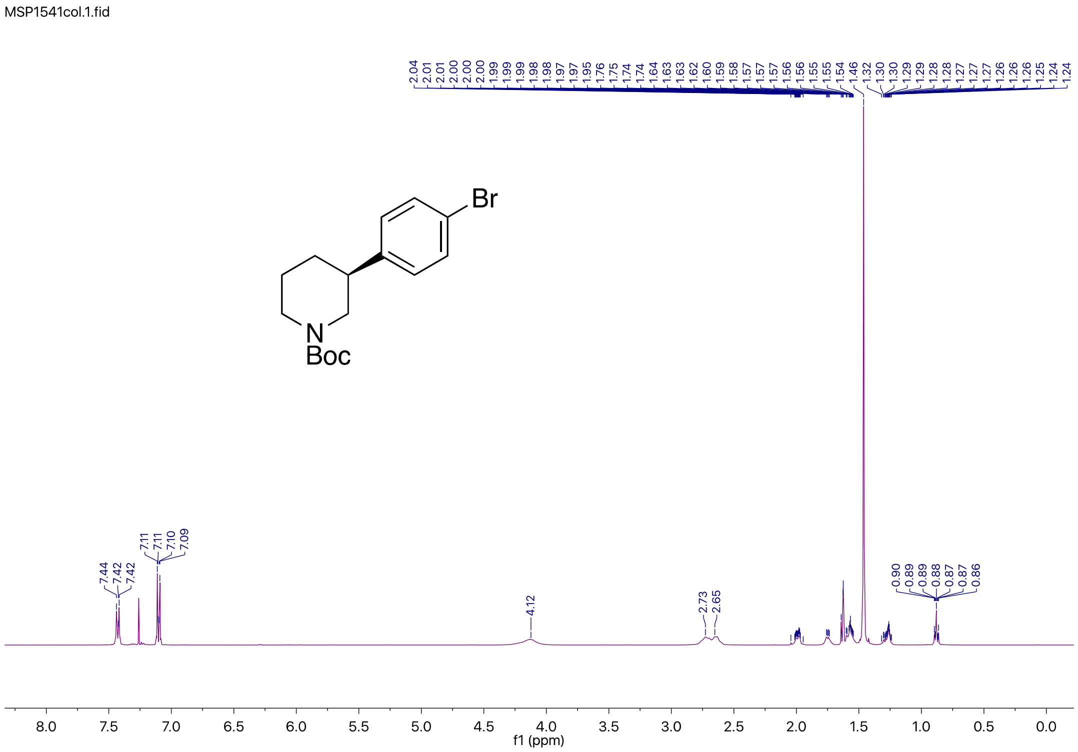

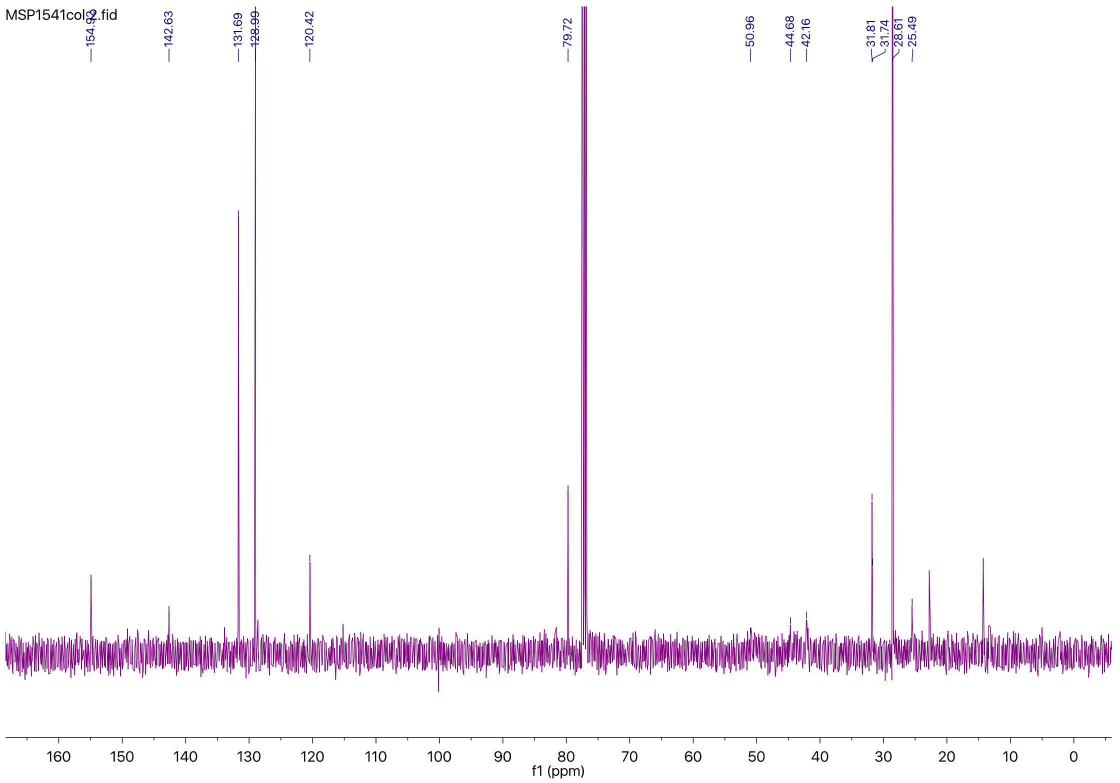
MSP1541col2.fid
154.92
142.63
131.69
128.99
120.42
79.72
50.96
44.68
42.16
31.81
31.74
28.61
25.49
160 150 140 130 120 110 100 90 80 70 60 50 40 30 20 10 0
f1 (ppm)

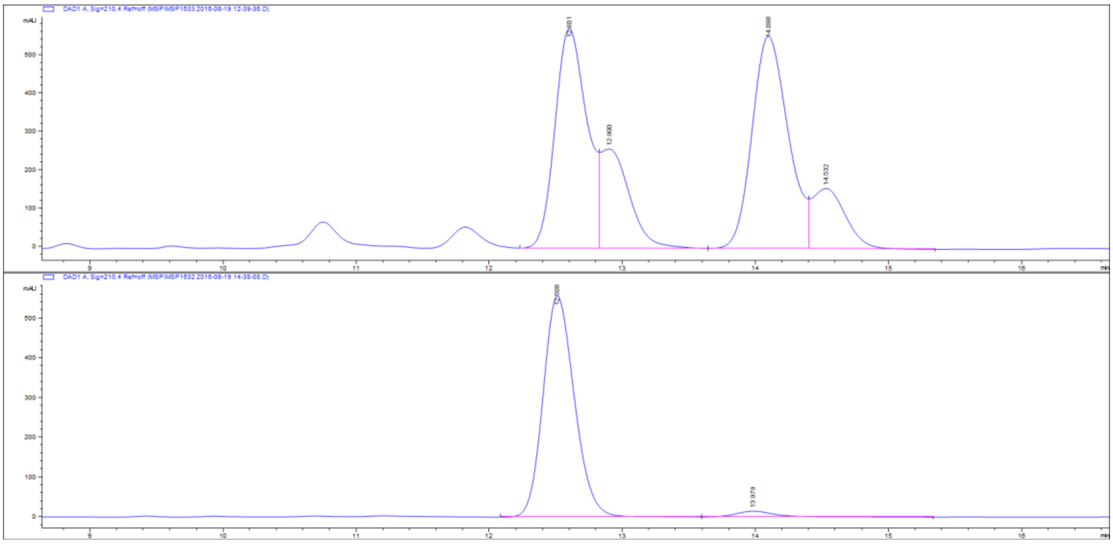

Supplementary figure 96: ^{1}H, ^{13}C-NMR spectra of compound **87**

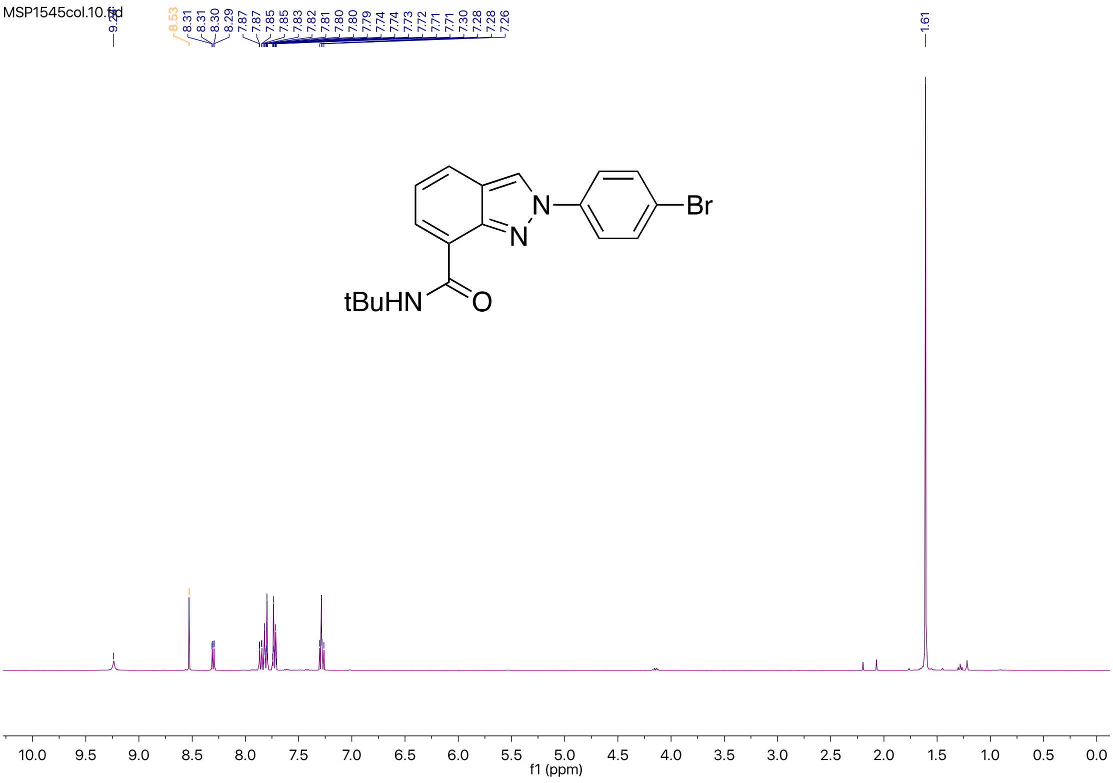

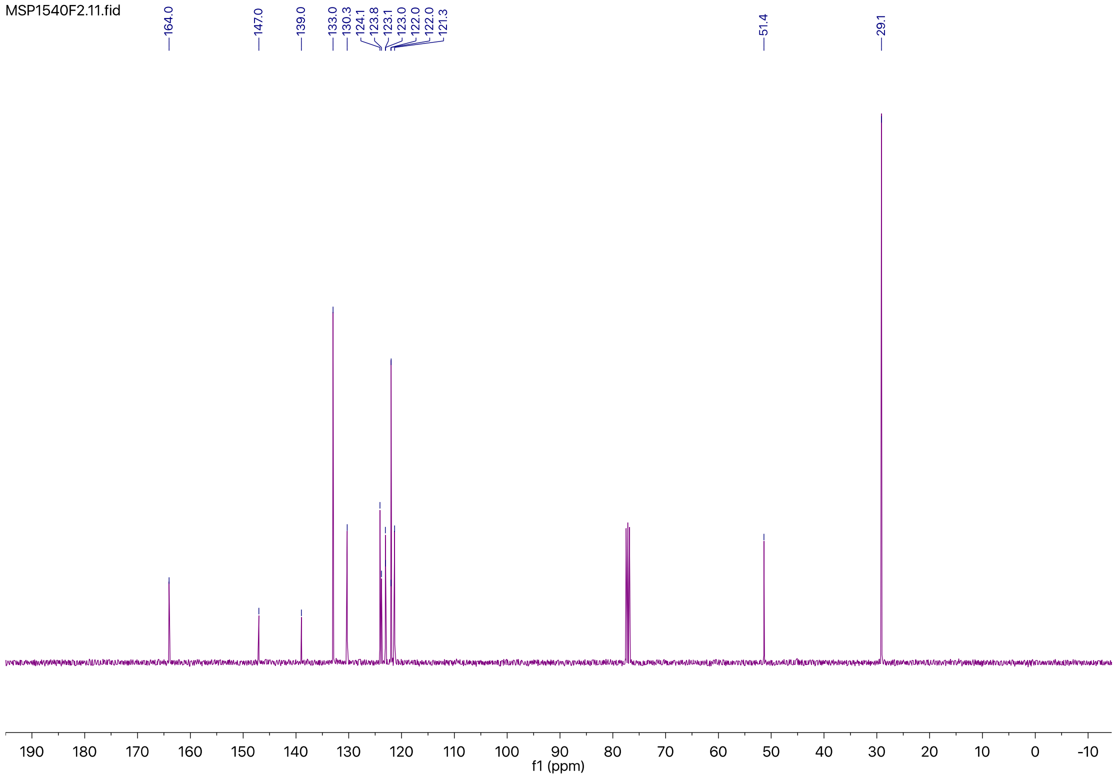
MSP1540F2.11.fid
164.0
147.0
139.0
133.0
130.3
124.1
123.8
123.1
123.0
122.0
122.0
121.3
51.4
29.1
190 180 170 160 150 140 130 120 110 100 90 80 70 60 50 40 30 20 10 0 -10
f1 (ppm)

Supplementary figure 97: ^{1}H, ^{13}C-NMR spectra of compound **88**

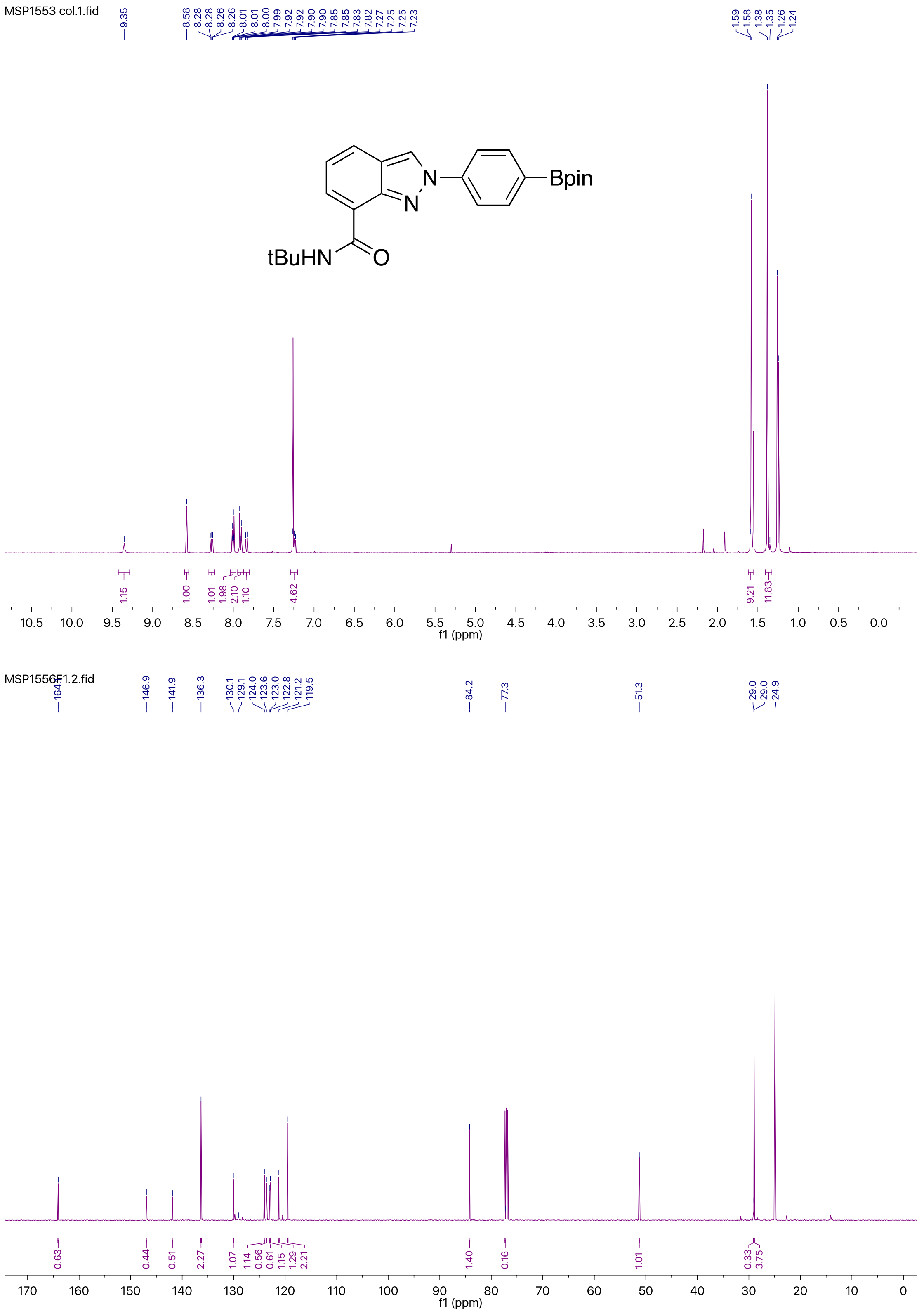

Supplementary figure 98: ^{1}H, ^{13}C-NMR spectra and HPLC traces of compound **89**

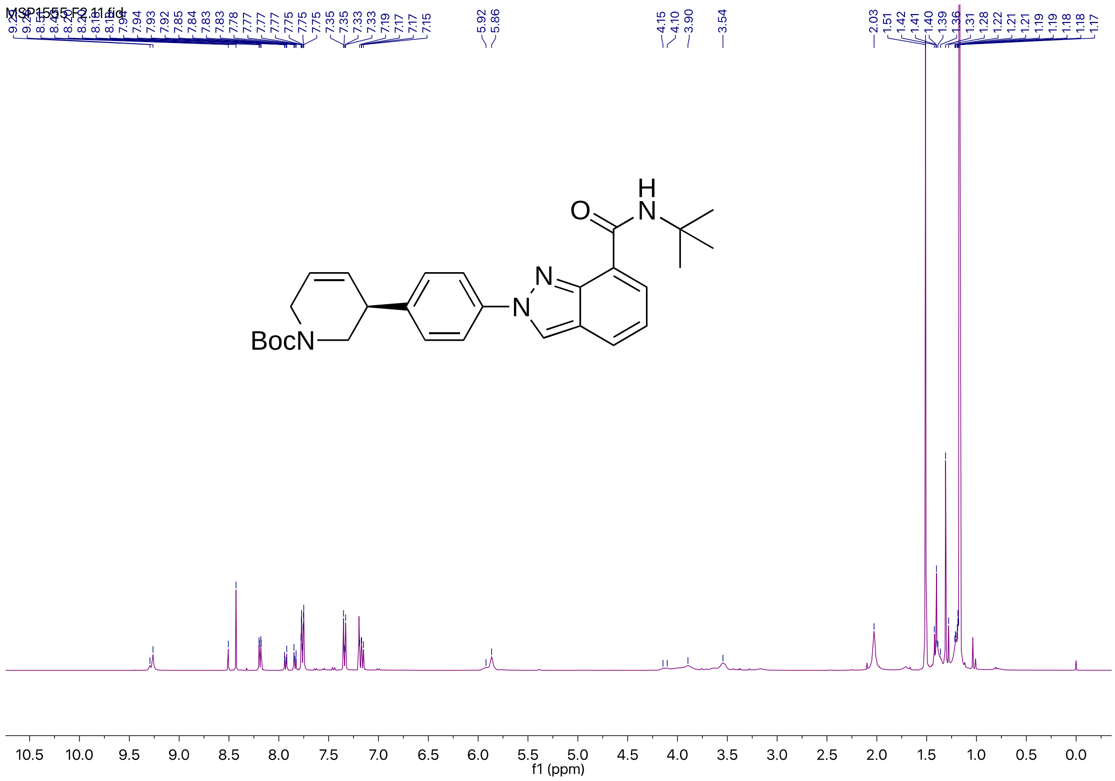

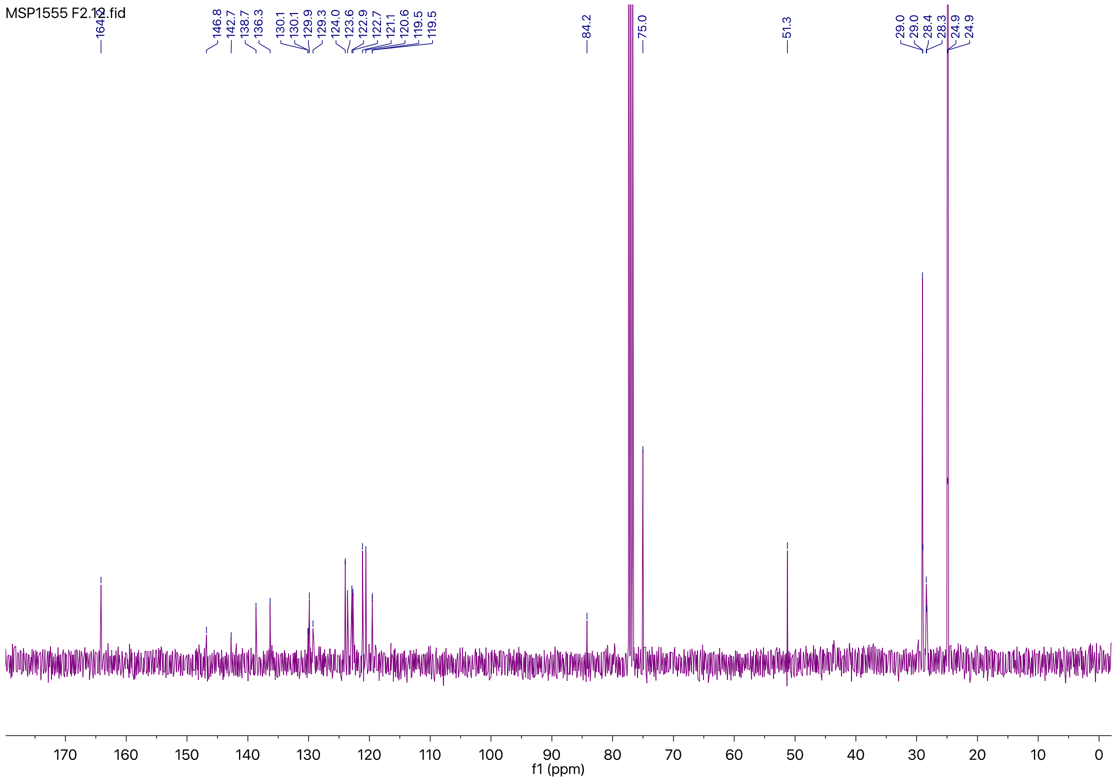

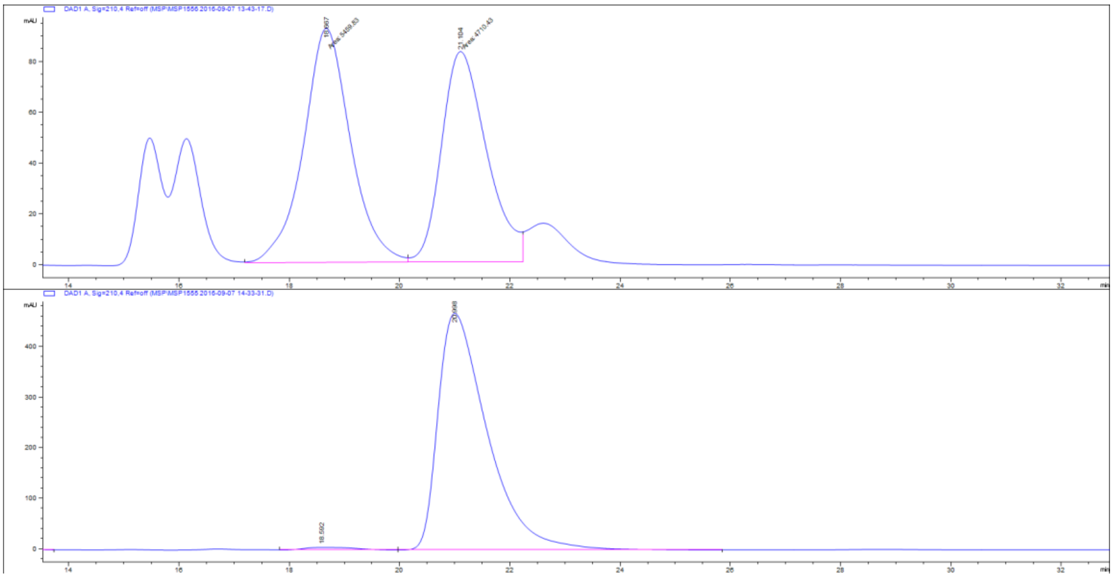

Supplementary figure 99: ^{1}H, ^{13}C-NMR spectra and HPLC traces of compound **90**

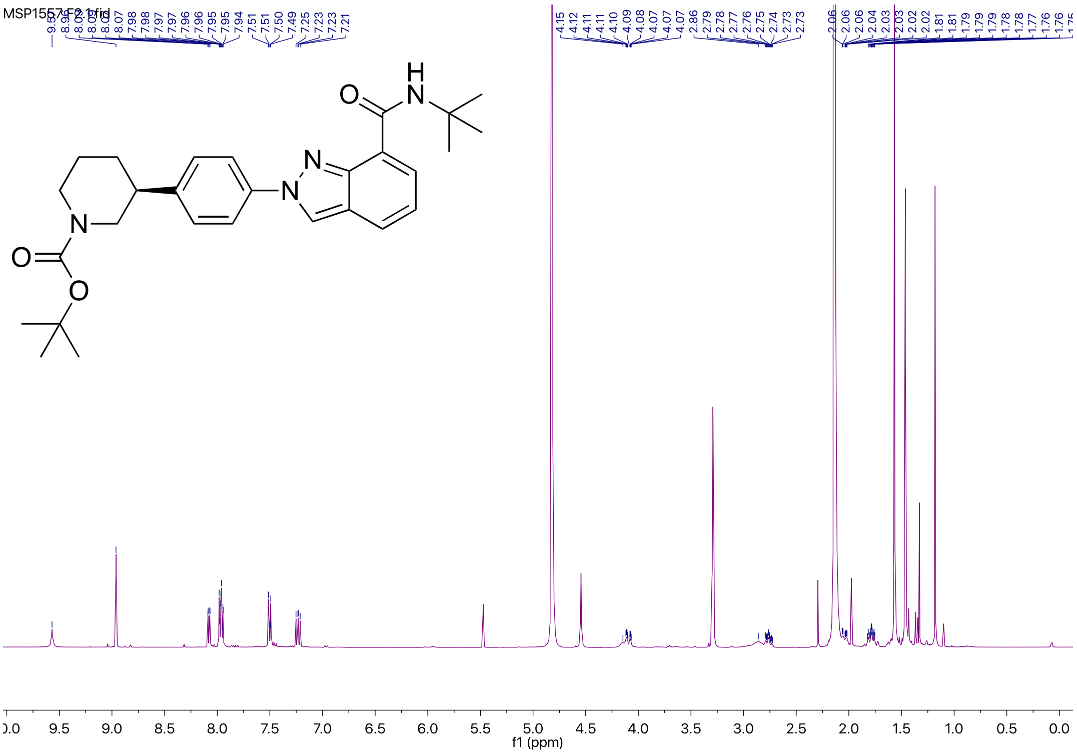

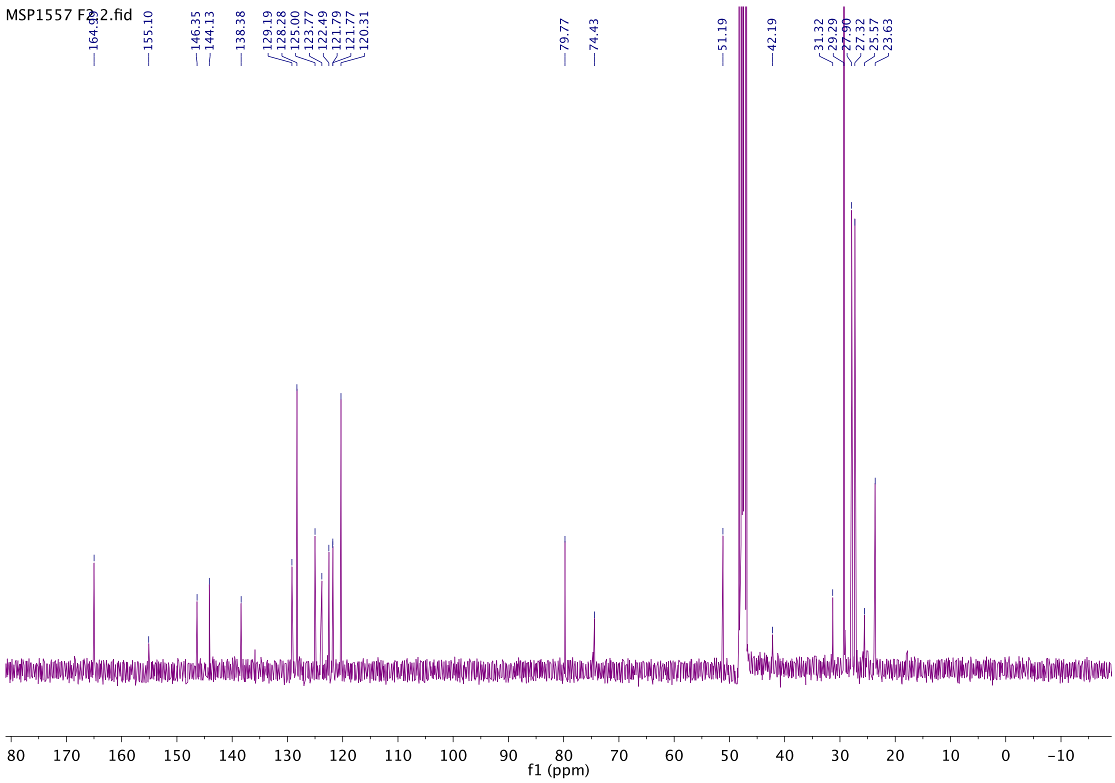

MSP1557 F2.2.fid
164.9?
155.10
146.35
144.13
138.38
129.19
128.28
125.00
123.77
122.49
121.79
121.77
120.31
79.77
74.43
51.19
42.19
31.32
29.29
27.90
27.32
25.57
23.63
80 170 160 150 140 130 120 110 100 90 80 70 60 50 40 30 20 10 0 -10
f1 (ppm)

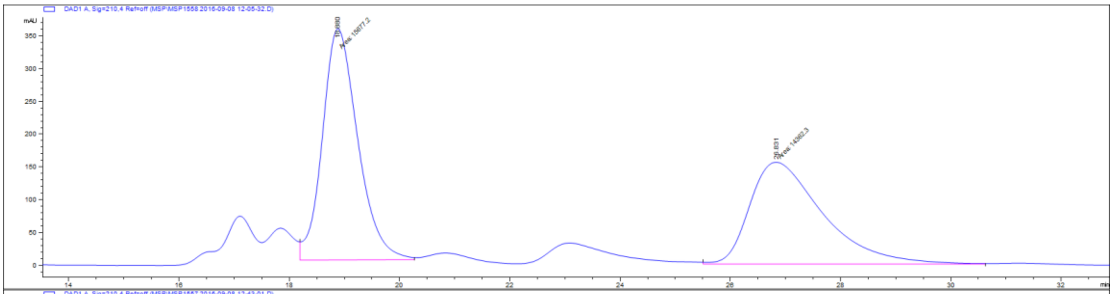
DAD1 A, Sig=210,4 Ref=off (MSP\MSP1558 2016-09-08 12-05-32.D)
mAU
Area: 15877.2
26.831
Area: 14362.3
min

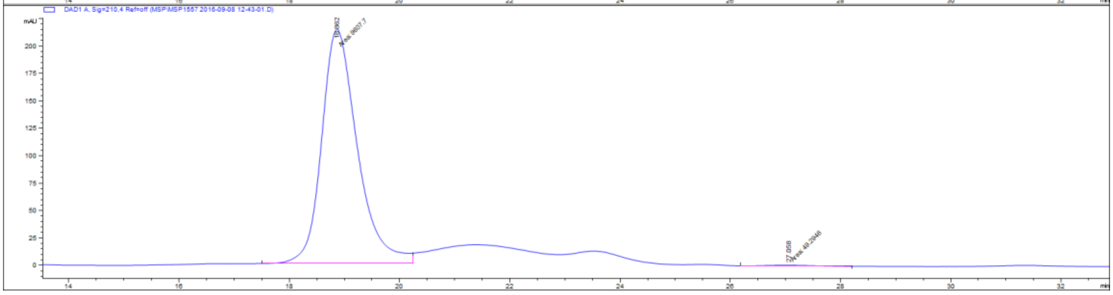
DAD1 A, Sig=210,4 Ref=off (MSP\MSP1557 2016-09-08 12-43-01.D)
mAU
Area: 9607.7
27.058
Area: 49.2946
min

Supplementary References

1. You, H., Rideau, E., Sidera, M. & Fletcher, S. P. Non-stabilized nucleophiles in Cu-catalysed dynamic kinetic asymmetric allylic alkylation. *Nature* **517,** 351–355 (2015).
2. Katcher, M. H. & Doyle, A. G. Palladium-Catalyzed Asymmetric Synthesis of Allylic Fluorides. *J. Am. Chem. Soc.* **132,** 17402–17404 (2010).
3. Takahata, H., Suto, Y., Kato, E., Yoshimura, Y. & Ouchi, H. A New Preparation of HomochiralN-Protected 5-Hydroxy-3- piperidenes, Promising Chiral Building Blocks, by Palladium- Catalyzed Deracemization of Their Alkyl Carbonates. *Adv. Synth. Catal.* **349,** 685–693 (2007).
4. Bouillon, A. *et al.* Synthesis of novel halopyridinylboronic acids and esters. Part 4: Halopyridin-2-yl-boronic acids and esters are stable, crystalline partners for classical Suzuki cross-coupling. *Tetrahedron* **59,** 10043–10049 (2003).
5. Sidera, M. & Fletcher, S. P. Rhodium-catalysed asymmetric allylic arylation of racemic halides with arylboronic acids. *Nat. Chem.* **7,** 935–939 (2015).
6. Amat, M. *et al.* Dynamic kinetic resolution of racemic gamma-aryl-delta-oxoesters. Enantioselective synthesis of 3-arylpiperidines. *J. Org. Chem.* **67,** 5343–51 (2002).
7. Kang, C.-Q., Cheng, Y.-Q., Guo, H.-Q., Qiu, X.-P. & Gao, L.-X. The natural alkaloid isoanabasine: synthesis from 2,3′-bipyridine, efficient resolution with BINOL, and assignment of absolute configuration by Mosher's method. *Tetrahedron: Asymmetry* **16,** 2141–2147 (2005).
8. Jones, P. *et al.* Discovery of 2-{4-[(3S)-piperidin-3-yl]phenyl}-2H-indazole-7-carboxamide (MK-4827): A novel oral poly(ADP-ribose)polymerase (PARP) inhibitor efficacious in BRCA-1 and -2 mutant tumors. *J. Med. Chem.* **52,** 7170–7185 (2009).
9. Chung, C. K. *et al.* Process development of C-N cross-coupling and enantioselective biocatalytic reactions for the asymmetric synthesis of niraparib. *Org. Process Res. Dev.* **18,** 215–227 (2014).
